# Repotrectinib in *NTRK* fusion–positive advanced solid tumors: a phase 1/2 trial

**DOI:** 10.1038/s41591-025-04079-7

**Published:** 2026-02-04

**Authors:** Benjamin Besse, Jessica J. Lin, Lyudmila Bazhenova, Koichi Goto, Adrianus Johannes de Langen, Dong-Wan Kim, Jürgen Wolf, Christoph Springfeld, Sanjay Popat, Darren W. T. Lim, Misako Nagasaka, Jung Yong Hong, Christina S. Baik, Alice Hervieu, Victor Moreno, Nong Yang, Kanthi Kollengode, Haisu Yang, Yuanfang Xu, Christophe Y. Calvet, Yong Yuan, Amy B. Hammell, Alexander Drilon, Benjamin J. Solomon

**Affiliations:** 1 Paris-Saclay University, Gustave Roussy Cancer Center, Villejuif, France; 2 Mass General Brigham Cancer Institute, Harvard Medical School, Boston, MA USA; 3 University of California, San Diego, Moores Cancer Center, La Jolla, CA USA; 4 National Cancer Center Hospital East, Kashiwa, Japan; 5 Netherlands Cancer Institute, Amsterdam, Netherlands; 6 Seoul National University Hospital, Seoul, South Korea; 7 Center for Integrated Oncology, University Hospital Cologne, Cologne, Germany; 8 Heidelberg University Hospital, National Center for Tumor Diseases, Department of Medical Oncology, Heidelberg, Germany; 9 The Royal Marsden NHS Foundation Trust, London, UK; 10 National Cancer Centre Singapore, Singapore, Singapore; 11 University of California, Irvine, School of Medicine, Orange, CA USA; 12 Samsung Medical Center, Sungkyunkwan University School of Medicine, Seoul, Korea; 13 University of Washington School of Medicine, Fred Hutchinson Cancer Center, Seattle, WA USA; 14 Centre Georges-François Leclerc, Dijon, France; 15 START MADRID-FJD, Hospital Fundación Jiménez Díaz, Madrid, Spain; 16 The Second People’s Hospital of Hunan Province, Hunan, China; 17 Bristol Myers Squibb, Princeton, NJ USA; 18 Memorial Sloan Kettering Cancer Center, Weill Cornell Medical College, New York, NY USA; 19 Peter MacCallum Cancer Centre, Melbourne, Victoria Australia

**Keywords:** Diseases, Non-small-cell lung cancer

## Abstract

Early-generation TRK tyrosine kinase inhibitors (TKIs) approved for treating *NTRK* fusion–positive (*NTRK*^+^) solid tumors provide clinical benefit; however, resistance emerges. Repotrectinib is a next-generation ROS1/TRK TKI with a compact macrocyclic structure designed to improve durability of response. TRIDENT-1 is a registrational phase 1/2 trial assessing repotrectinib, a next-generation ROS1/TRK TKI, in adults with advanced solid tumors, including *NTRK*^+^ disease. The primary endpoint was confirmed objective response; secondary endpoints included duration of response (DOR), progression-free survival (PFS), overall survival and safety. Median follow-up ranged between 21.3 months and 25.7 months. In the TKI-naive cohort (*n* = 51; 95% confidence interval (CI)), the response rate was 59% (44–72); the median DOR was not estimable (NE); and the median PFS was 30.3 months (9.0–NE). In the TKI-pretreated cohort (*n* = 69; 95% CI), the response rate was 48% (36–60); the median DOR was 9.8 months (7.4–13.0); and the median PFS was 7.4 months (3.9–9.7). Of 30 TKI-pretreated patients with *NTRK* solvent front mutations, 16 had a response (53%; 95% CI: 34–72). Intracranial responses were observed in two of three TKI-naive patients and in four of six TKI-pretreated patients with measurable intracranial disease at baseline. Among all treated patients (*n* = 565), the most common any-grade treatment-related adverse event (TRAE) was dizziness (57%); most TRAEs were low grade; and 4% discontinued repotrectinib due to a TRAE. Here repotrectinib demonstrated durable systemic and intracranial responses with generally low-grade adverse events in patients with *NTRK*^+^ solid tumors, including those with previous TRK TKI treatment and solvent front mutations. These results support the use of repotrectinib to treat patients with *NTRK*^+^ solid tumors. ClinicalTrials.gov identifier: NCT03093116.

## Abstract (web summary)

In the phase 1/2 TRIDENT-1 trial, treatment of patients with *NTRK* fusion–positive advanced solid tumors with the tyrosine kinase inhibitor repotrectinib—selective for ROS1, TRKA−C and ALK—was safe and resulted in durable systemic and intracranial clinical response.

## Main

*NTRK1/NTRK2/NTRK3* fusions (*NTRK*) drive oncogenesis through aberrant tropomyosin receptor kinase (TRK) protein signaling^1^ in up to 0.7% of solid tumors, although prevalence varies greatly by tumor type^2,3^. *NTRK* fusions are common in secretory carcinoma of the breast (91%) and in mammary analog secretory carcinoma of the salivary gland (83–90%) among adult patients^3^; by contrast, the frequency of *NTRK* fusions in non-small cell lung cancer (NSCLC) is 0.2%^4^. The use of early-generation TRK TKIs larotrectinib and entrectinib resulted in meaningful clinical benefit in patients with *NTRK*^+^ solid tumors^5,6^; however, durability of responses to these agents is limited by acquired resistance, including on-target *NTRK* mutations^7,8^. On-target mutations may occur in the following regions: the solvent front (for example, TRKA G595R, TRKB G639R and TRKC G623R), the xDFG motif (for example, TRKA G667C, TRKB G709C and TRKC G696A) and the gatekeeper residue. These mutations alter the TRK kinase domain conformation, causing interference with TRK inhibitor binding. Bypass resistance or off-target resistance mechanisms have also been described and include activation of non-TRK oncoproteins, such as activating BRAF or KRAS mutations^9^.

A TRK TKI that can maintain durable response among patients with or without previous TRK TKI treatment and address acquired resistance mutations would provide meaningful benefit to patients with *NTRK*^+^ solid tumors. With its compact macrocyclic structure that circumvents steric hindrance^10^, repotrectinib potently inhibits wild-type TRKA/TRKB/TRKC fusion proteins (half maximal inhibitory concentration (IC_50_) < 0.2 nmol l^−1^) and those with resistance mutations (IC_50_ ≤2.6 nmol l^−1^)^8^. Repotrectinib is a next-generation TRK and ROS1 TKI, approved in the United States for treatment of adult patients with *ROS1* fusion–positive (*ROS1*^+^) locally advanced or metastatic NSCLC and for treatment of adult or pediatric patients (12 years and older) with *NTRK*^+^ locally advanced or metastatic solid tumors^11,12^. Repotrectinib was recently added to the National Comprehensive Cancer Network Clinical Practice Guidelines in Oncology (NCCN Guidelines) as a preferred first-line treatment option for patients with *NTRK*^+^ NSCLC^13^ and as a treatment option in various *NTRK*^+^ solid tumors^14–16^, including soft tissue sarcoma^17^ and cancers of the breast^18^, rectum^19^ and colon^20^.

TRIDENT-1 is an ongoing international, registrational phase 1/2 trial evaluating repotrectinib in adult patients with *ROS1*^+^ and *NTRK*^+^ tumors^21^. We report the efficacy outcomes of repotrectinib in patients with *NTRK*^+^ solid tumors, including patients with *NTRK* solvent front mutations, and its safety in all patients regardless of tumor or fusion type.

## Results

### Patients and treatment

**Fig. 1:**
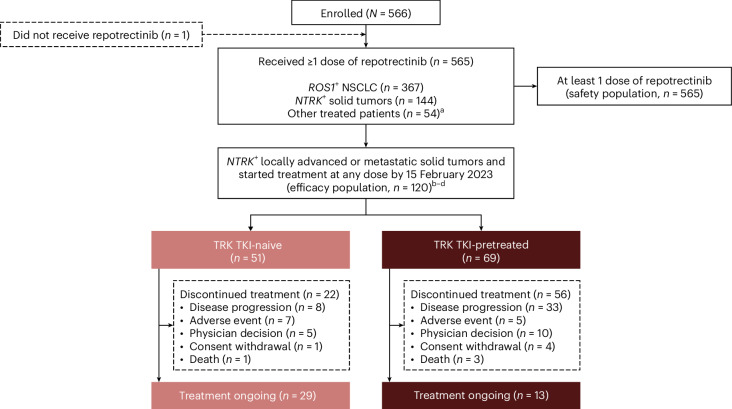
CONSORT diagram. ^a^Other treated patients include patients with *ROS1*^+^ non-NSCLC, *ALK* gene fusions and any gene fusions with discordant results between local FISH test and central laboratory test. ^b^At data cutoff, 42 patients remained on treatment (29 in the TRK TKI-naive cohort and 13 in the TRK TKI-pretreated cohort). The most common reason for discontinued treatment was disease progression (41 patients). ^c^Nine *NTRK*^+^ patients in phase 1 (five in the TRK TKI-naive cohort and four in the TRK TKI-pretreated cohort). ^d^Efficacy analysis set includes treated patients with at least 6 months of follow-up after the first post-baseline scan.

**Extended Data Fig. 1:**
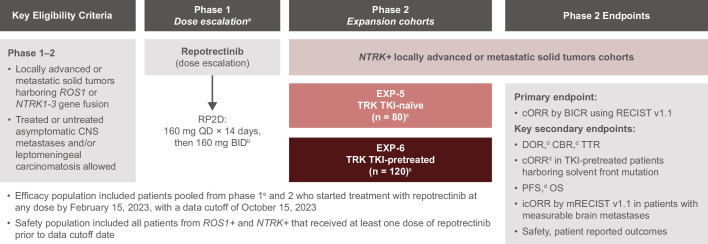
Overview of the Phase 1–2 TRIDENT-1 Study Design: *NTRK*+ Cohorts. Abbreviations: blinded independent central review (BICR), twice daily (BID), clinical benefit rate (CBR), central nervous system (CNS), confirmed objective response rate (cORR), duration of response (DOR), intracranial objective response rate (icORR), modified RECIST (mRECIST), *NTRK* fusion–positive (*NTRK*+), once daily (QD), response evaluation criteria in solid tumors (RECIST), *ROS1* fusion–positive (*ROS1*+), tyrosine kinase inhibitor (TKI), tropomyosin receptor kinase (TRK), time to response (TTR). ^a^Phase 1 primary endpoints: DLT, MTD, RP2D. ^b^Based on tolerability. ^c^Ns for expansion cohort indicate enrollment targets. ^d^By RECIST v1.1. ^e^Patients from phase 1 received 40 mg QD to 160 mg QD and 160 mg BID.

**Extended Data Fig. 2:**
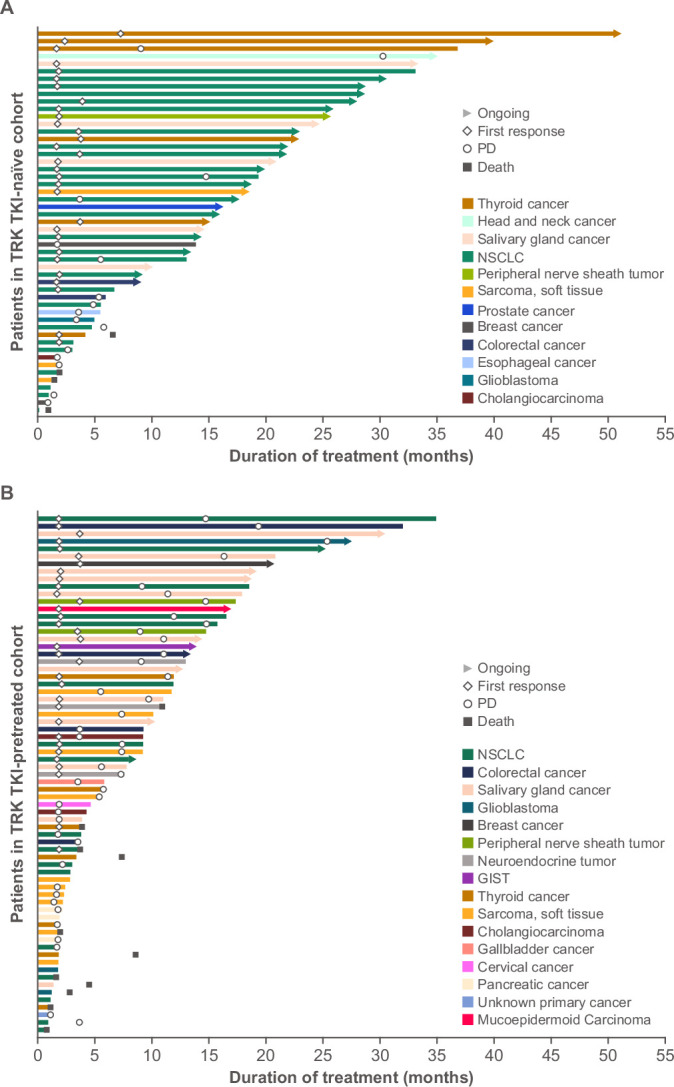
Duration of Repotrectinib Treatment in Patients with *NTRK*+ Locally Advanced or Metastatic Solid Tumors. TRK TKI-naïve cohort (**A**) and TRK TKI-pretreated cohort (**B**). Abbreviations: gastrointestinal stromal tumor (GIST), non-small cell lung cancer (NSCLC), *NTRK* fusion–positive (*NTRK*+), progressive disease (PD), tyrosine kinase inhibitor (TKI), tropomyosin receptor kinase (TRK).

From 27 February 2017 through 15 October 2023, 144 patients with *NTRK*^+^ solid tumors were enrolled in TRIDENT-1 (Extended Data Fig. 1). Of the 135 patients in phase 2, 112 (83%) increased dose from 160 mg once daily to 160 mg twice daily at day 15 per protocol. The efficacy population included 120 patients with *NTRK*^+^ solid tumors who started treatment with repotrectinib at any dose by 15 February 2023 (phase 1, *n* = 9; phase 2, *n* = 111) (Fig. 1). At data cutoff, 42 patients (35%) were still receiving treatment across the *NTRK*^+^ cohorts (29 TKI-naive; 13 TKI-pretreated). The median duration of treatment was 33.1 months (range, 0.1–51.2+) for TKI-naive and 7.6 months (range, 0.6–32.0) for TKI-pretreated cohorts. The most common reason for treatment discontinuation was disease progression (*n* = 41; 34%). Extended Data Fig. 2 shows the duration of treatment. Supplementary Table 1 summarizes subsequent therapies.

### Activity

#### Efficacy population

The efficacy population included 51 TKI-naive patients and 69 TKI-pretreated patients with sufficient follow-up for analysis (that is, at least 6 months of follow-up for tumor assessment after first post-baseline scan) (Table 1). Among 18 *NTRK*^+^ tumor types reported, NSCLC was the most common at 53% and 25% in the TKI-naive and TKI-pretreated cohorts, respectively, followed by thyroid cancer (12% and 10%), salivary gland cancer (10% and 17%) and soft tissue sarcoma (6% and 14%) (Supplementary Fig. 1). A total of 12 and 16 unique cancer types were treated in the TKI-naive and TKI-pretreated cohorts, respectively. Patients with asymptomatic leptomeningeal carcinomatosis were eligible, although none enrolled. The median age was 61 years (range, 25–84) in the TKI-naive cohort and 56 years (range, 18–81) in the TKI-pretreated cohort; approximately half were women (53% and 48%, respectively). Per blinded independent central review (BICR), intracranial disease at baseline was present in 20% and 23% of patients, respectively (Table 1).

**Table 1:** Characteristics of patients at baseline (efficacy population)^a^

Characteristic	TRK TKI-naive (*N* = 51)^b^	TRK TKI-pretreated (*N* = 69)^c^
Age		
Median (range) — years	61 (25–84)	56 (18–81)
Distribution — no. (%)		
≥18 to <65 years	30 (59)	44 (64)
≥65 years	21 (41)	25 (36)
Female sex — no. (%)	27 (53)	33 (48)
Ethnicity — no. (%)^d^		
Hispanic or Latino	2 (4)	1 (1)
Not Hispanic or Latino	46 (90)	64 (93)
Missing	3 (6)	4 (6)
Geographic region — no. (%)		
United States	8 (16)	24 (35)
Asia	23 (45)	15 (22)
Other^e^	20 (39)	30 (43)
ECOG performance status — no. (%)^f^		
0	23 (45)	27 (39)
1	28 (55)	42 (61)
Stage 4 metastatic disease at study entry — no. (%)	49 (96)	63 (91)
Missing^g^	0	1 (1)
Intracranial disease — no. (%)^h^		
Yes	10 (20)	16 (23)
No	41 (80)	53 (77)
No. of previous lines of systemic therapy — no. (%)^i^		
0	18 (35)	0
1	18 (35)	18 (26)
2	12 (24)	23 (33)
≥3	3 (6)	28 (41)
No. of previous lines of chemotherapy with or without immunotherapy — no. (%)^j^		
0	22 (43)	26 (38)
1	21 (41)	29 (42)
2	6 (12)	13 (19)
≥3	2 (4)	1 (1)
*NTRK* gene fusion — no. (%)^k^		
*NTRK1*	25 (49)	22 (32)
*NTRK2*	2 (4)	4 (6)
*NTRK3*	24 (47)	43 (62)

^a^ The efficacy population included patients with *NTRK*^+^ locally advanced or metastatic solid tumors. Percentages may not total 100 because of rounding. NA denotes not applicable.

^b^ The TRK TKI-naive cohort included five patients from phase 1 and 46 patients from phase 2.

^c^ The TRK TKI-pretreated cohort included four patients from phase 1 and 65 patients from phase 2.

^d^ Percentages may not total 100 based on reported data.

^e^ Other regions included Australia, Canada and Europe.

^f^ Eastern Cooperative Oncology Group (ECOG) performance status ranges from 0 to 5, with 0 indicating no symptoms and higher scores indicating greater disability.

^g^ At time of diagnosis, data on disease stage were missing for two patients (4%) from the TRK TKI-naive cohort and for five patients (7%) from the TRK TKI-pretreated cohort.

^h^ Intracranial disease at baseline was confirmed by BICR.

^i^ In the TRK TKI-naive and TRK TKI-pretreated cohorts, 11 patients (22%) and 12 patients (17%) received one previous line of immunotherapy with/without chemotherapy, respectively.

^j^ Nine patients from the TRK TKI-naive cohort received immunotherapy-based regimens as last treatment prior to repotrectinib. Among those nine patients, eight had NSCLC and one had breast cancer.

^k^ *NTRK1*−*3* gene fusions were identified by tissue-based local testing using next-generation sequencing, quantitative polymerase chain reaction or FISH with prospective confirmation by a central diagnostic laboratory.

#### TRK TKI-naive patients

**Fig. 2:**
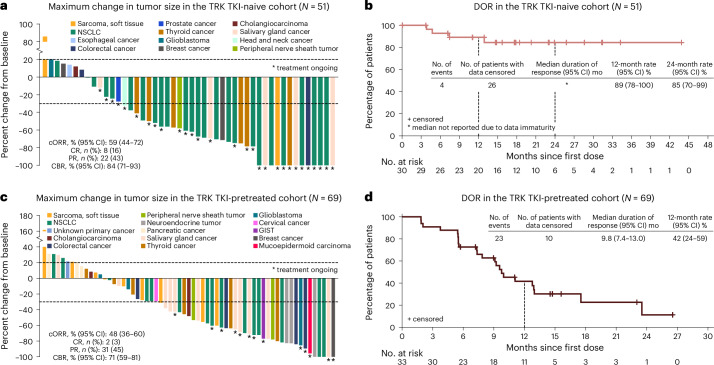
Efficacy outcomes of repotrectinib in the efficacy population. Shown are the change in the tumor burden (**a**) and DOR (**b**) in 51 TKI-naive patients (five patients from phase 1 and 46 patients from phase 2) and the change in the tumor burden (**c**) and DOR (**d**) in 69 TKI-pretreated patients (four patients from phase 1 and 65 patients from phase 2). In **a** and **c**, the waterfall plots include only patients with baseline and post-baseline target lesion measurements at baseline and during follow-up; dashed lines indicate a reduction of 30% or an increase of 20% from baseline in the tumor size, as assessed according to RECIST version 1.1. Clinical benefit rate (CBR) refers to complete response plus partial response and stable disease. cORR, confirmed objective response rate; CR, complete response; mo, months; PR, partial response.

**Extended Data Fig. 3:**
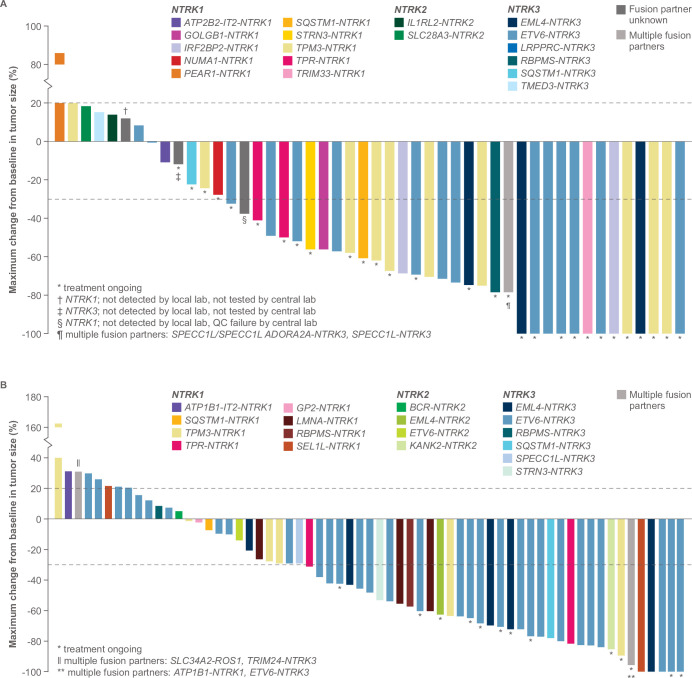
Change in Tumor Burden in Patients with *NTRK*+ Locally Advanced or Metastatic Solid Tumors by *NTRK* Gene and Fusion Partner. TRK TKI-naïve cohort (**A**) and TRK TKI-pretreated cohort (**B**). Waterfall plots only include patients with baseline and post-baseline target lesion measurements. Abbreviations: *NTRK* fusion–positive (*NTRK*+), tyrosine kinase inhibitor (TKI), tropomyosin receptor kinase (TRK).

**Extended Data Fig. 4:**
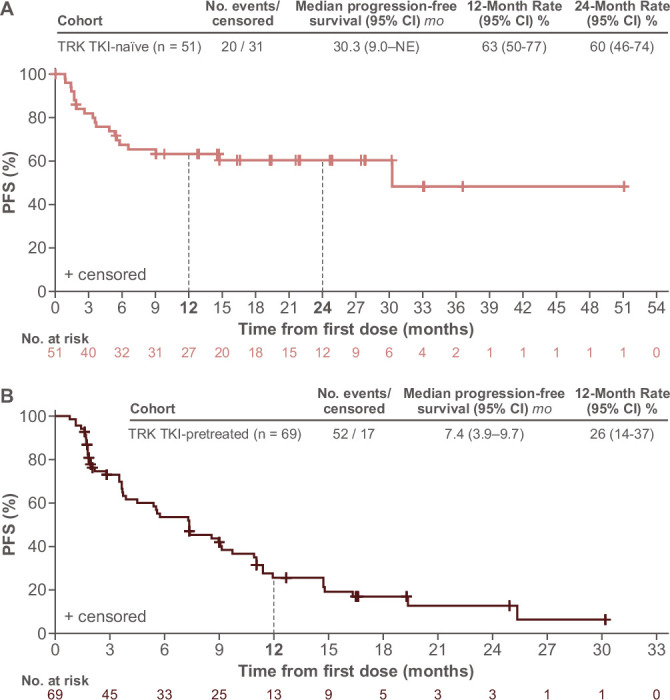
Progression-Free Survival in Patients with *NTRK*+ Locally Advanced or Metastatic Solid Tumors. TRK TKI-naïve cohort (**A**) and TRK TKI-pretreated cohort (**B**). Abbreviations: not evaluable (NE), *NTRK* fusion–positive (*NTRK*+), progression-free survival (PFS), tyrosine kinase inhibitor (TKI), tropomyosin receptor kinase (TRK).

**Extended Data Fig. 5:**
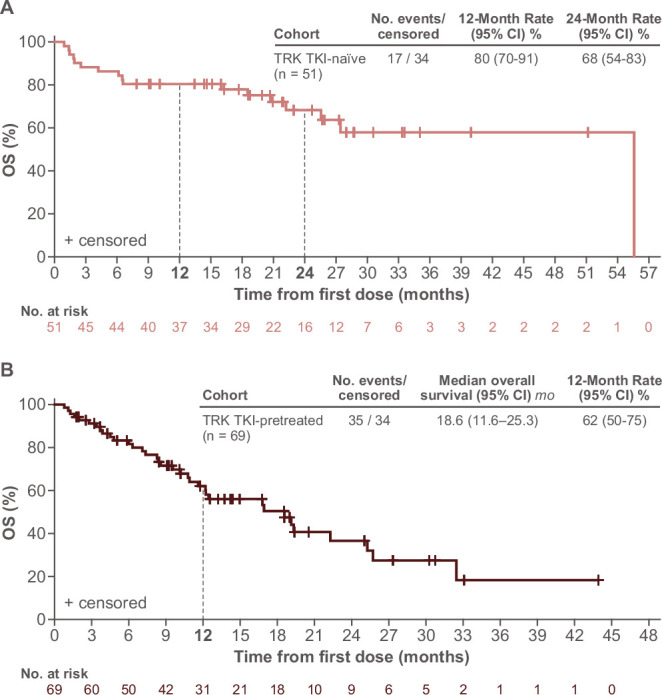
Overall Survival in Patients with *NTRK*+ Locally Advanced or Metastatic Solid Tumors. TRK TKI-naïve cohort (**A**) and TRK TKI-pretreated cohort (**B**). Abbreviations: *NTRK* fusion–positive (*NTRK*+), overall survival (OS), tyrosine kinase inhibitor (TKI), tropomyosin receptor kinase (TRK).

In the TKI-naive cohort, median follow-up was 25.7 months (range, 8.7–74.5). Thirty patients had confirmed response (59%; 95% CI: 44–72), eight (16%) had complete response and 22 (43%) had partial response (Table 2 and Fig. 2a), with median time to response of 1.8 months (1.6–7.3). Responses were observed regardless of tumor type, *NTRK* gene or fusion partner (Extended Data Fig. 3a). Median DOR was NE, and an estimated 85% of responders (95% CI: 70–99) had response lasting at least 24 months (Fig. 2b). The median PFS was 30.3 months (95% CI: 9.0–NE), and an estimated 60% of patients (95% CI: 46–74) were alive without progression for at least 24 months (Extended Data Fig. 4a). Estimated 24-month overall survival rate was 68% (95% CI: 54-83) (Extended Data Fig. 5a); overall survival was considered immature. Of 18 patients without prior systemic therapy, 61% (95% CI: 36–83) had a response (Supplementary Table 2). Among 27 patients who had *NTRK*^+^ NSCLC, 63% (95% CI: 42–81) had a response (Supplementary Table 3). Efficacy outcomes by sex are shown in Supplementary Table 4.

**Table 2:** Repotrectinib efficacy outcomes (efficacy population)

Variable	TRK TKI-naive (*N* = 51)	TRK TKI-pretreated (*N* = 69)
Confirmed objective response^a,b^		
No. of patients with response	30	33
Percentage of patients with response (95% CI)	59 (44–72)	48 (36–60)
Median time to response (range) — months	1.8 (1.6–7.3)	1.9 (1.7–3.7)
Median duration of response (95% CI) — months	NE (NE–NE)	9.8 (7.4–13.0)
Best overall response — no. (%)^a^		
Complete response	8 (16)	2 (3)
Partial response	22 (43)	31 (45)
Stable disease	13 (25)	16 (23)
Progressive disease	5 (10)	13 (19)
Not evaluable	0	3 (4)
Missing^c^	3 (6)	4 (6)
Clinical benefit^d^		
No. of patients with clinical benefit	43	49
Percentage of patients with benefit (95% CI)	84 (71–93)	71 (59–81)
Median PFS (95% CI) — months	30.3 (9.0–NE)	7.4 (3.9–9.7)
Median overall survival (95% CI) — months	–^e^	18.6 (11.6–25.3)
Intracranial objective response^f^		
No. of patients with measurable intracranial disease at baseline	3	6^g^
No. of patients with response	2^h^	4
Percentage of patients with response (95% CI)	67 (9–99)	67 (22–96)
Complete response — no. (%)	2 (67)	0
Partial response — no. (%)	0	4 (67)
Range of intracranial duration of response — months	17.5–24.0	5.5–10.6
Intracranial PFS in patients without baseline intracranial disease^I,j^		
No. of patients without intracranial disease at baseline^k^	41	53
Median intracranial PFS (95% CI) — months	NE (30.3–NE)	7.4 (4.5–11.0)

^a^ Objective response (complete or partial response) was assessed by BICR according to RECIST version 1.1.

^b^ Among nine patients who received immunotherapy-based regimens prior to repotrectinib, eight were evaluable and five had partial response to repotrectinib.

^c^ For all patients with missing overall response data, the missingness was due to lack of post-baseline tumor assessment; these patients were assumed to be non-responders.

^d^ Clinical benefit, defined as a best overall response of confirmed complete response, confirmed partial response or stable disease as assessed by BICR according to RECIST version 1.1, was a prespecified secondary endpoint.

^e^ Median overall survival for TRK TKI-naive cohort is not reported due to data immaturity.

^f^ Intracranial response was assessed by BICR according to modified RECIST version 1.1.

^g^ All patients in the TRK TKI-pretreated cohort with measurable intracranial disease at baseline received TKI as their most recent prior therapy, and three patients (two with partial response and one with missing response status) had prior treatment for CNS metastases (two with radiotherapy and one with intracranial surgery). Three of the responders had documented intracranial progression after treatment with repotrectinib.

^h^ One of the two responders in the TRK TKI-naive cohort had prior brain radiation, and neither responder had documented intracranial progression after treatment with repotrectinib.

^i^ Intracranial PFS in patients without baseline intracranial disease was assessed by BICR according to modified RECIST version 1.1.

^j^ Among patients with NSCLC in the TRK TKI-naive cohort, six had intracranial disease at baseline and 21 did not; no patient had documented intracranial progression. Among patients with NSCLC in the TRK TKI-pretreated cohort, five had intracranial disease at baseline and 11 did not; one patient (with intracranial disease at baseline) had documented intracranial progression.

^k^ Among patients without intracranial disease at baseline, no intracranial progression events were reported.

#### TRK TKI-pretreated patients

In the TKI-pretreated cohort, 63 patients (91%) received TKI as their most recent prior therapy, of whom 56 (89%) discontinued due to disease progression. At a median follow-up of 21.3 months (range, 8.4–79.2), 33 of 69 patients (48%; 95% CI: 36–60) had a confirmed response, two (3%) had complete response and 31 (45%) had partial response (Table 2 and Fig. 2c). The median time to response was 1.9 months (range, 1.7–3.7), and the median DOR was 9.8 months (95% CI, 7.4–13.0). An estimated 42% of responders (95% CI: 24–59) had responses lasting at least 12 months (Fig. 2d). Responses were observed regardless of tumor type, *NTRK* gene or fusion partner (Extended Data Fig. 3b). The median PFS was 7.4 months (95% CI: 3.9–9.7), and an estimated 26% of patients (95% CI: 14–37) were alive and without progression for at least 12 months (Extended Data Fig. 4b). The median overall survival was 18.6 months (95% CI: 11.6–25.3); the estimated 12-month overall survival rate was 62% (95% CI: 50–75) (Extended Data Fig. 5b). Efficacy outcomes by sex are shown in Supplementary Table 4. Among TKI-pretreated patients, 46% received prior entrectinib and 52% received larotrectinib. Confirmed response occurred in 18 of 32 patients (56%) who previously received entrectinib and in 14 of 36 patients (39%) who previously received larotrectinib (Supplementary Table 5). A total of 63 patients (91%) received TKI as their most recent prior therapy (Supplementary Table 6). Overall, 17 patients had *NTRK*^+^ NSCLC; 53% (95% CI: 28–77) had a response.

Results were also assessed in a subgroup of patients with extended follow-up (at least 19 months from treatment initiation) (Supplementary Tables 7 and 8). Outcomes were similar to the efficacy population.

### Intracranial activity

**Extended Data Fig. 6:**
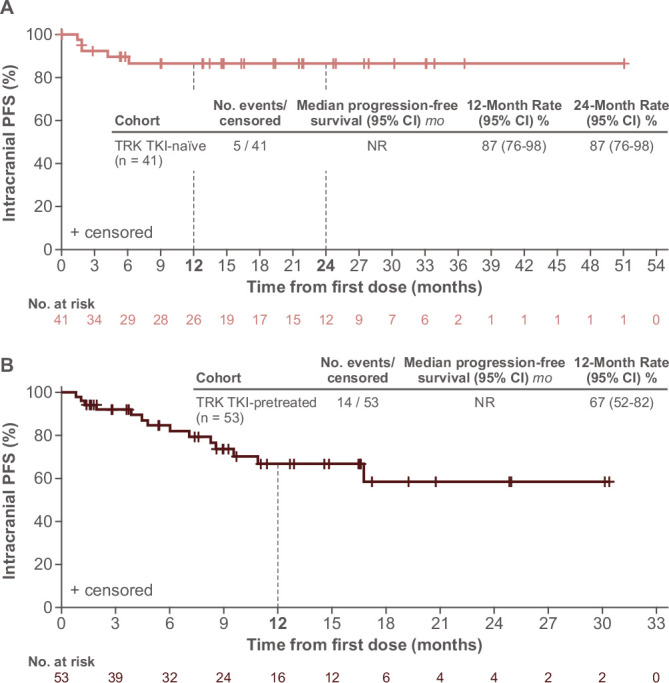
Intracranial Progression-Free Survival in Patients with *NTRK*+ Locally Advanced or Metastatic Solid Tumors Without Intracranial Disease at Baseline. TRK TKI-naïve cohort (**A**) and TRK TKI-pretreated cohort (**B**). NR indicates that the median was not reached. Abbreviations: not reached (NR), *NTRK* fusion–positive (*NTRK*+), progression-free survival (PFS), tyrosine kinase inhibitor (TKI), tropomyosin receptor kinase (TRK).

Systemic (intracranial and extracranial) efficacy outcomes in patients with and without baseline intracranial disease confirmed by BICR was observed (Supplementary Table 9). Among patients from phase 2 with measurable intracranial disease at baseline, intracranial response occurred in two of three patients in the TKI-naive cohort and in four of six patients in the TKI-pretreated cohort (Table 2). Range for intracranial DOR was 17.5–24.0 months and 5.5–10.6 months in these cohorts, respectively, and both responders (100%) in the TKI-naive cohort had intracranial response for at least 12 months. Among patients without intracranial disease at baseline, intracranial PFS (defined as time from administration of first dose to development of new brain lesions as first progression assessed by BICR or death) was 87% at 24 months in the TKI-naive cohort and 67% at 12 months in the TKI-pretreated cohort (Extended Data Fig. 6).

### Solvent front mutations

**Fig. 3:**
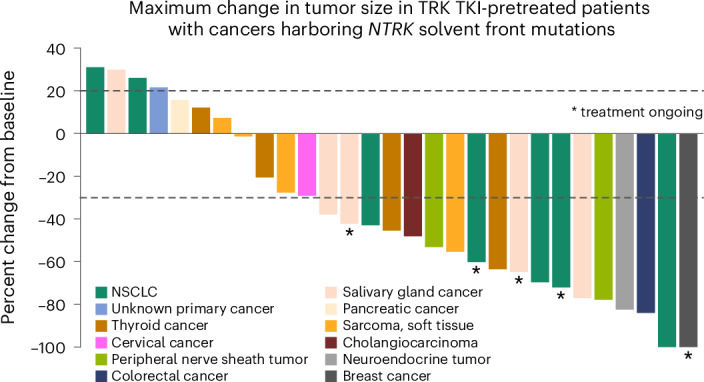
Efficacy outcomes in TRK TKI-pretreated patients with *NTRK* solvent front mutations. Shown are changes in tumor burden in 30 TRK TKI-pretreated patients with *NTRK* solvent front mutations. Asterisks indicate that treatment is ongoing, and dashed lines indicate a reduction of 30% or an increase of 20% from baseline in the tumor size, as assessed according to RECIST version 1.1.

**Extended Data Fig. 7:**
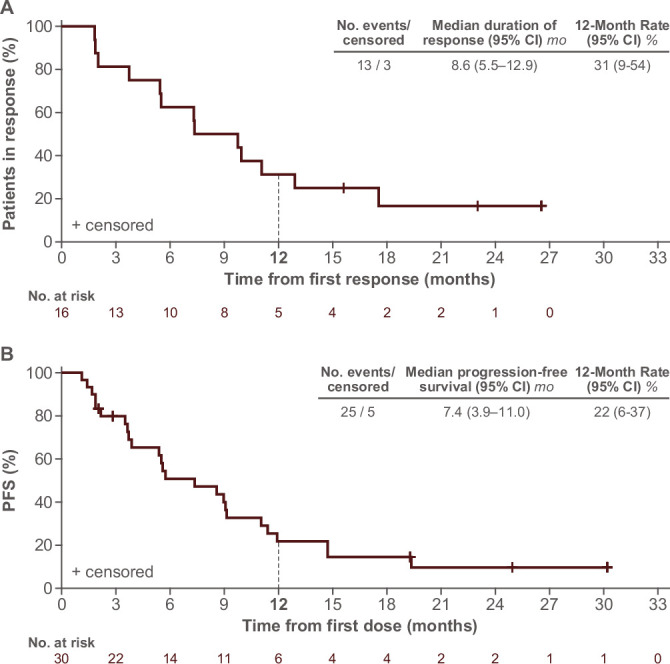
Duration of Response and Progression-Free Survival in Patients with *NTRK*+ Locally Advanced or Metastatic Solid Tumors and *NTRK* Solvent Front Mutation at Baseline. Duration of response (**A**) and progression-free survival (**B**). Abbreviations: *NTRK* fusion–positive (*NTRK*+), progression-free survival (PFS).

Among the 69 TKI-pretreated patients, 30 (43%) had *NTRK* solvent front mutations at baseline (Supplementary Table 10). Of these, 16 (53%; 95% CI: 34–72) had confirmed response (Fig. 3 and Supplementary Table 11); the median DOR was 8.6 months (95% CI: 5.5–12.9), and the median PFS was 7.4 months (95% CI: 3.9–11.0) (Extended Data Fig. 7). Among the 37 patients (54%) without solvent front mutations, 17 (46%, 95% CI: 30–63) had confirmed response.

### Repotrectinib resistance

An exploratory analysis was conducted to examine resistance mutations developed by patients with disease progression on repotrectinib. Among 28 patients with paired baseline and end-of-treatment samples, 20 discontinued repotrectinib due to disease progression or death (two TKI-naive, 18 TKI-pretreated). No emergent *NTRK* mutations were detected in the two TKI-naive patients, and three emergent *NTRK3* solvent front mutations were detected in the 18 TKI-pretreated patients (Supplementary Table 12). Notably, five patients who had mutations at baseline had no mutations detected at end of treatment.

Of the 18 patients with progressive disease as best overall response, seven had *NTRK* mutations at screening, all of which were absent at end of treatment (Supplementary Table 13). Additionally, activation of *MAPK*/*PI3K* pathways at baseline and the presence of *p53* mutations/deletions at baseline were observed in some of these patients. Future analysis of resistance mechanisms may be evaluated based on analyses of circulating tumor DNA (ctDNA) samples collected at baseline, on treatment and after progression.

### Safety

Among 565 patients with any tumor or fusion type who received at least one dose of repotrectinib, the most common any-grade TRAEs were dizziness (57%), dysgeusia (50%) and paresthesia (30%) (Table 3 and Supplementary Table 14). Most TRAEs were grade 1 or grade 2 (71%). Grade 3 or higher TRAEs occurred in 162 patients (29%); the most common were anemia (4%), dizziness (3%) and increased blood creatine phosphokinase level (3%).

**Table 3:** Adverse events in patients who received at least one dose of repotrectinib^a^

Event	All patients treated with at least one dose of repotrectinib (*N* = 565)^b^				*NTRK*^+^ cohorts (*N* = 144)			
	Treatment-emergent		Treatment-related		Treatment-emergent		Treatment-related	
	Any grade	Grade ≥3	Any grade	Grade ≥3	Any grade	Grade ≥3	Any grade	Grade ≥3
	Number of patients (percent)							
Any event	562 (99)	323 (57)	535 (95)	162 (29)	143 (99)	83 (58)	139 (97)	49 (34)
Event occurring in ≥15% of patients								
Dizziness	356 (63)	17 (3)	324 (57)	17 (3)	90 (63)	7 (5)	83 (58)	7 (5)
Dysgeusia	296 (52)	0	283 (50)	0	81 (56)	0	78 (54)	0
Constipation	222 (39)	1 (<1)	146 (26)	0	59 (41)	1 (1)	39 (27)	0
Anemia	215 (38)	50 (9)	148 (26)	22 (4)	59 (41)	16 (11)	43 (30)	9 (6)
Paresthesia	192 (34)	3 (1)	168 (30)	3 (1)	51 (35)	1 (1)	44 (31)	1 (1)
Dyspnea	177 (31)	38 (7)^c^	0	0	44 (31)	6 (4)	0	0
Fatigue	140 (25)	7 (1)	93 (16)	4 (1)	42 (29)	3 (2)	28 (19)	3 (2)
Increased alanine aminotransferase level	125 (22)	11 (2)	97 (17)	7 (1)	27 (19)	2 (1)	20 (14)	2 (1)
Ataxia	124 (22)	2 (<1)	119 (21)	1 (<1)	34 (24)	0	33 (23)	0
Muscular weakness	122 (22)	11 (2)	85 (15)	7 (1)	28 (19)	3 (2)	23 (16)	2 (1)
Increased aspartate aminotransferase level	118 (21)	15 (3)	98 (17)	7 (1)	27 (19)	6 (4)	21 (15)	3 (2)
Nausea	117 (21)	7 (1)	68 (12)	2 (<1)	31 (22)	2 (1)	18 (13)	1 (1)
Headache	113 (20)	2 (<1)	0	0	28 (19)	1 (1)	0	0
Cough	107 (19)	1 (<1)	0	0	29 (20)	1 (1)	0	0
Increased blood creatine phosphokinase level	99 (18)	19 (3)	89 (16)	17 (3)	29 (20)	4 (3)	25 (17)	4 (3)
Arthralgia	86 (15)	2 (<1)	0	0	18 (13)	0	0	0
Diarrhea	85 (15)	5 (1)	0	0	33 (23)	4 (3)	0	0
Event that led to treatment discontinuation	61 (11)	NA	23 (4)	NA	14 (10)	NA	5 (3)	NA
Event that led to dose reduction	216 (38)	NA	195 (35)	NA	65 (45)	NA	63 (44)	NA
Event that led to dose interruption	291 (52)	NA	197 (35)	NA	76 (53)	NA	61 (42)	NA
Any serious event	230 (41)	NA	48 (8)	NA	56 (39)	NA	18 (13)	NA
Death	35 (6)	NA	2 (<1)	NA	8 (6)	NA	1 (1)	NA

NA denotes not applicable.

^a^ Adverse events were categorized according to preferred terms of the Medical Dictionary for Regulatory Activities version 26.1 and were graded according to the National Cancer Institute Common Terminology Criteria for Adverse Events version 4.03.

^b^ Within the safety population, 24 patients were exposed to immunotherapy as last treatment prior to repotrectinib. Among those, adverse events leading to dose reduction occurred in 11 patients (46%), and grade 3−4 TRAEs occurred in 13 patients (54%). Treatment discontinuation due to adverse event occurred in one patient (4%). One patient died from unexplained death that was not related to treatment.

^c^ Two patients (<1%) had grade 5 dyspnea not related to repotrectinib.

Treatment-emergent adverse events (TEAEs) led to dose reduction in 216 patients (38%), dose interruption in 291 patients (52%) and treatment discontinuation in 61 patients (11%) (Table 3). Among patients who escalated to 160 mg twice a day and had subsequent dose reduction due to TEAE within 3 months, an exploratory analysis demonstrated that median DOR in the TKI-naive and TKI-pretreated cohorts was not reached and 13.0 months (95% CI: 3.8–NE), respectively (Supplementary Fig. 2 and Supplementary Methods). The most common TEAEs that led to treatment discontinuation were dyspnea, pneumonitis and muscular weakness (1% each). No patients discontinued treatment due to dizziness. Serious TEAEs occurred in 230 patients (41%), and fatal TEAEs occurred in 35 patients (6%). Fatal TRAEs occurred in two patients, including sudden death in one patient from the TKI-pretreated *ROS1*^+^ NSCLC cohort and cardiorespiratory arrest in one patient from the TKI-pretreated *NTRK*^+^ solid tumor cohort. Safety outcomes were generally similar among all treated patients with *NTRK*^+^ solid tumors; however, dizziness, dysgeusia and ataxia appeared to occur more frequently in TKI-naive patients compared to TKI-pretreated patients (Supplementary Table 15).

### Patient-reported outcomes

**Extended Data Fig. 8:**
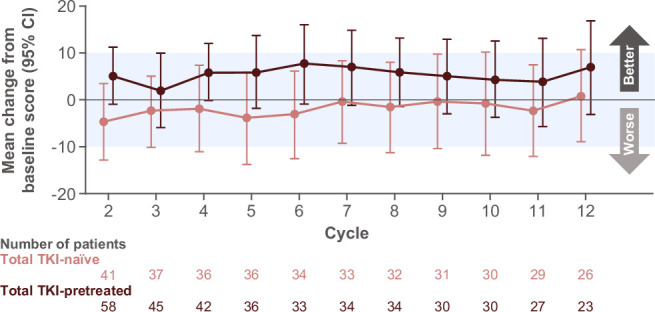
Mean Score Change from Baseline of EORTC QLQ-C30 Global Health Status/Quality of Life Score at Each Cycle in Patients in TRK TKI-Naïve and TRK TKI-Pretreated Cohorts. Abbreviations: European Organisation for Research and Treatment of Cancer (EORTC), tyrosine kinase inhibitor (TKI), tropomyosin receptor kinase (TRK).

A total of 111 patients with *NTRK*^+^ solid tumors in phase 2 had European Organisation for Research and Treatment of Cancer Quality of Life Questionnaire–Core 30 (EORTC QLQ-C30) assessments (46 TKI-naive and 65 TKI-pretreated), of whom ≥89% completed each assessment through cycle 12 (Extended Data Fig. 8), and 62–97% completed assessments between cycles 13 and 17. Among TKI-naive patients reporting data, mean global health status score at baseline was 67.4 (s.d. 24.2); 65% of patients had a stable score (<10-point increase or decrease from baseline) or improved score (≥10-point increase from baseline) at cycle 12 and cycle 17. Among TKI-pretreated patients reporting data, mean global health status score at baseline was 65.5 (s.d. 21.2); 78% and 69% of patients had a stable or improved score at cycle 12 and cycle 17, respectively.

## Discussion

In this phase 1/2 trial, repotrectinib showed clinical activity in patients with a diverse variety of *NTRK*^+^ solid tumors, with responses observed regardless of tumor type, *NTRK* gene, intracranial disease status, prior treatment and *NTRK* resistance mutation status. Among TKI-naive patients, 59% had confirmed response, and responses were durable, as 85% of responders had responses lasting at least 24 months, and 60% were progression free for at least 24 months.

Other TRK inhibitors also reported activity in *NTRK*^+^ solid tumors with similar response rates (larotrectinib, 66%; entrectinib, 62%)^5,6^. Median PFS with repotrectinib (30.3 months) was similar to larotrectinib (30.8 months), and that of entrectinib was reported as 15.7 months. However, cross-trial comparisons should be interpreted with caution due to differences between trials, such as tumor type distribution and inclusion of pediatric patients. Although data are limited on how response rates vary between tumor types, certain tumors in adults, such as secretory carcinomas of the breast and salivary gland, may respond particularly well to TRK inhibitors compared to other tumor types^22,23^. Among pediatric patients, infantile fibrosarcoma has also been associated with dramatic responses with TRK inhibitors; for repotrectinib, pediatric patients are being evaluated separately (CARE; NCT04094610)^24,25^.

TRIDENT-1 is the first trial to demonstrate clinical efficacy outcomes in a TKI-pretreated population, with nearly half of the TKI-pretreated patients experiencing a confirmed response. Responses occurred in patients regardless of which TKI (larotrectinib or entrectinib) they received previously, and the median PFS was 7.4 months. Approximately half of the TKI-pretreated patients had on-target mutations associated with resistance to current TRK TKIs, with 44% having solvent front mutations at baseline. The response rate in patients with solvent front mutations was 53%, indicating the clinical relevance of the preclinical activity of repotrectinib against these mutations^8^.

The brain is a common site of disease progression, including in *NTRK*^+^ solid tumors^26,27^. As previously reported, repotrectinib was designed for enhanced intracranial activity and has shown antitumor activity in the brain in a patient-derived intracranial model^21^. In patients with *NTRK*^+^ solid tumors, repotrectinib was active regardless of the presence of baseline intracranial disease in both TKI-naive and TKI-pretreated cohorts, and evidence of durable intracranial responses was observed. Patients treated with repotrectinib who were without baseline intracranial disease showed no documented development of brain lesions.

Repotrectinib-related adverse events were mostly grade 1 or grade 2 (71%); dizziness was most common (57%). As TRKA/TRKB/TRKC receptors are known to be involved in the development and maintenance of the nervous system, neurologic adverse events such as dizziness are expected of TRK inhibition and were also observed in patients treated with larotrectinib and entrectinib^28,29^. TRAEs rarely led to treatment discontinuation (4%) in TRIDENT-1, and discontinuation because of dizziness was not reported. There did not appear to be a negative impact on DOR in patients with dose reduction due to an adverse event. Serious and fatal TEAEs occurred in 41% and 6% of treated patients, respectively. The safety profile of repotrectinib was consistent between patients with *NTRK*^+^ solid tumors and the overall safety population, including patients with *ROS1*^+^ NSCLC, as reported previously^21^.

This trial is limited by its single-arm design and small sample size, owing to the rare patient population with *NTRK*^+^ solid tumors. Additionally, NSCLC was the most frequent tumor type in the *NTRK*^+^ cohorts (37%), which is in line with real-world data^2^; however, responses were observed across other tumor types. Enrollment for the *NTRK*^+^ cohorts is ongoing, and time-to-event efficacy endpoints and safety will continue to be assessed for long-term outcomes.

In conclusion, repotrectinib, a next-generation TKI, showed durable clinical activity in adult patients with *NTRK*^+^ solid tumors, including in patients with previous TKI treatment, with or without *NTRK* solvent front mutations, across multiple *NTRK* genes and fusion partners and in patients with or without intracranial disease. Repotrectinib was mainly associated with low-grade adverse events, consistent with previous reports. This evidence supports repotrectinib as a new treatment option for patients with *NTRK*^+^ solid tumors.

Looking forward, studies with longer follow-up and larger patient populations will help understand how response durability translates to overall survival as well as ascertain the ideal treatment sequence (for example, via matching-adjusted indirect comparison analyses) for individual tumor types for which the treatment landscapes vary. Translational analyses will be important to further understand on-target and off-target resistance mechanisms to determine appropriate mitigation strategies, such as combination therapies or other novel TRK inhibitors. ctDNA surveillance could help identify early progression and the need for treatment escalation. Other useful studies would be the utility of TRK inhibitors in the early-stage setting and the benefit/risk in pediatric patients (<12 years) with alternative drug formulations.

## Methods

### Trial design and treatment

From the phase 1 component of TRIDENT-1 (NCT03093116), the recommended phase 2 dose (RP2D) of repotrectinib was determined as 160 mg once daily for 14 days, followed by 160 mg twice daily^21^. Phase 2 was conducted at 152 sites across 19 countries. Patients enrolled in six cohorts by tumor molecular characteristics and treatment history. Two cohorts consisted of patients with *NTRK*^+^ solid tumors and included patients without previous TRK TKI treatment (TKI-naive cohort) and patients treated with one or two previous TRK TKIs (TKI-pretreated cohort). The remaining four cohorts included patients with *ROS1*^+^ NSCLC and were previously described^21^. TRIDENT-1 trial design is provided in Extended Data Fig. 1; study protocol details were previously reported^21^.

### Trial endpoints and assessments

The phase 2 primary endpoint was confirmed objective response (complete response or partial response) as assessed by BICR according to Response Evaluation Criteria in Solid Tumors (RECIST) version 1.1. Key secondary endpoints included DOR; clinical benefit; PFS; overall survival; intracranial response in patients with measurable intracranial disease at baseline, as assessed by BICR according to modified RECIST (mRECIST) version 1.1 (ref. ^30^); safety as assessed with Common Terminology Criteria for Adverse Events version 4.03; and patient-reported outcomes as assessed with the EORTC QLQ-C30. Exploratory endpoints included potential prognostic utility of genomic alterations, emergence of repotrectinib resistance mutations and confirmed response by patient subgroup (demographic and baseline risk factors). Sex of patients was recorded by each study site.

### Patients

Eligible patients had tumors harboring an *NTRK* fusion and were at least 18 years of age in phase 1 or at least 12 years of age in phase 2. *NTRK* fusion was determined by local tissue-based testing with retrospective confirmation at a centralized diagnostic laboratory or determined locally by fluorescence in situ hybridization (FISH) with prospective confirmation by a central diagnostic laboratory before enrollment. Patients with asymptomatic central nervous system (CNS) metastases (treated or untreated) were allowed to enroll. Efficacy population included all patients from *NTRK*^+^ cohorts pooled from phase 1 and phase 2 with at least one measurable target lesion according to RECIST version 1.1 and prospectively confirmed by BICR, who started treatment with repotrectinib at any dose by 15 February 2023, with data cutoff of 15 October 2023. Biomarker assay methods used to identify *NTRK* resistance mutations are in the Supplementary Methods. Safety population included all patients treated prior to data cutoff of 15 October 2023 who received any dose of repotrectinib in phase 1 or phase 2, regardless of tumor or fusion type.

### Trial oversight

This trial was sponsored and designed by Turning Point Therapeutics, a wholly owned subsidiary of Bristol Myers Squibb. Input was provided by investigators with agreement to keep all trial aspects and outcomes confidential. The trial was conducted following US Food and Drug Administration regulations and the International Council for Harmonisation E6 guideline for Good Clinical Practice. Appropriate health authorities and institutional committees reviewed the protocol (Supplementary Methods)^21^. All patients provided written informed consent. The clinical safety committee (phase 1), the data and safety monitoring committee (phase 2) and Turning Point Therapeutics provided clinical trial oversight. All authors participated in drafting the manuscript, and medical writing support was funded by the sponsor. Authors verified data accuracy and comprehensiveness and fidelity of the trial to the protocol.

### Statistical analysis

For both overall and intracranial response data, the proportion of patients with confirmed response was reported with 95% CIs, calculated using the two-sided 95% Clopper–Pearson method. Time-to-event endpoints were reported using the Kaplan–Meier method, with 95% CIs determined using the Greenwood variance estimate. CIs should not be used in place of hypothesis testing; CI widths were not adjusted for multiple comparisons. Additional details are provided in the Supplementary Methods and were reported previously^21^.

The protocol-specified target sample sizes for the TKI-naive and TKI-pretreated cohorts were 55 and 40, respectively (see the Supplementary Methods for details). The target sample size of 55 patients for the TKI-naive cohort was not achieved within the reported cutoff date. We report herein patient populations of 51 and 69 for the TKI-naive and TKI-pretreated cohorts, respectively, with at least 6 months of follow-up for tumor assessment after first post-baseline scan, which is consistent with the registrational dataset agreed upon with regulatory agencies.

### Reporting summary

Further information on research design is available in the Nature Portfolio Reporting Summary linked to this article.

## Online content

Any methods, additional references, Nature Portfolio reporting summaries, source data, extended data, supplementary information, acknowledgements, peer review information; details of author contributions and competing interests; and statements of data and code availability are available at 10.1038/s41591-025-04079-7.

## Supplementary information

Supplementary Information List of investigators and study sites, Supplementary methods, Supplementary Figs. 1 and 2, Supplementary Tables 1−15, Supplementary references, Redacted protocol, Redacted protocol amendment and Redacted statistical analysis plan.

Reporting Summary

## Data Availability

Genomic data for this study are available at the European Genome-Phenome Archive (study, EGAS50000001572; dataset, EGAD50000002249). These and other data for this study may be requested in accordance with Bristol Myers Squibb’s processes to ensure compliance with patient privacy and regulatory requirements. In-scope proposals for data requests are sent to and reviewed by an independent review committee (IRC) at the Duke Clinical Research Institute at Duke University. Review by an IRC is conducted to ensure that proposals requesting patient-level data receive a complete, consistent and fair assessment, and they provide the final decision on the requests. The IRC consists of experts in three broadly defined areas of expertise, including clinical, statistical and bioethical/protection of human subjects. The IRC may also discuss the proposal with the study research team and additional experts if needed for the request. Proposals are evaluated based on scientific rationale and methodology, experience and relevant qualifications of the research team, presence of a robust statistical analysis plan and publication plan. Plans for addressing potential conflicts of interest should be addressed. The researcher(s) will be expected to sign the Vivli Data Use Agreement prior to release of data, and the deidentified and/or anonymized datasets will be available within the Vivli Research environment upon agreement. The policy on data sharing for Bristol Myers Squibb may be found at https://www.bms.com/researchers-and-partners/independent-research/data-sharing-request-process.html.

## References

[1] Cocco, E., Scaltriti, M. & Drilon, A. *NTRK* fusion-positive cancers and TRK inhibitor therapy. *Nat. Rev. Clin. Oncol.* **15**, 731–747 (2018). 30333516 10.1038/s41571-018-0113-0 PMC6419506

[2] Westphalen, C. B. et al. Genomic context of *NTRK1/2/3* fusion-positive tumours from a large real-world population. *NPJ Precis. Oncol.* **5**, 69 (2021). 34285332 10.1038/s41698-021-00206-y PMC8292342

[3] O’Haire, S. et al. Systematic review of NTRK 1/2/3 fusion prevalence pan-cancer and across solid tumours. *Sci. Rep.* **13**, 4116 (2023). 36914665 10.1038/s41598-023-31055-3 PMC10011574

[4] Overbeck, T. R. et al. *NTRK* gene fusions in non-small-cell lung cancer: real-world screening data of 1068 unselected patients. *Cancers (Basel)* **15**, 2966 (2023). 37296928 10.3390/cancers15112966 PMC10252111

[5] Drilon, A. et al. Efficacy and safety of larotrectinib in a pooled analysis of patients (Pts) with tropomyosin receptor kinase (TRK) fusion cancer. *Ann. Oncol.* **34**, S470 (2023).

[6] Lu, S. et al. Updated efficacy and safety data of entrectinib in patients (pts) with locally advanced/metastatic *NTRK* fusion-positive (fp) solid tumours. *Ann. Oncol.* **34**, S468–S469 (2023).

[7] Drilon, A. et al. What hides behind the MASC: clinical response and acquired resistance to entrectinib after *ETV6*-*NTRK3* identification in a mammary analogue secretory carcinoma (MASC). *Ann. Oncol.* **27**, 920–926 (2016). 26884591 10.1093/annonc/mdw042 PMC4843186

[8] Murray, B. W. et al. Molecular characteristics of repotrectinib that enable potent inhibition of TRK fusion proteins and resistant mutations. *Mol. Cancer Ther.* **20**, 2446–2456 (2021). 34625502 10.1158/1535-7163.MCT-21-0632 PMC9762329

[9] Harada, G. & Drilon, A. TRK inhibitor activity and resistance in TRK fusion-positive cancers in adults. *Cancer Genet.* **264-265**, 33–39 (2022). 35334340 10.1016/j.cancergen.2022.03.002 PMC9133157

[10] Drilon, A. et al. Repotrectinib (TPX-0005) is a next-generation ROS1/TRK/ALK inhibitor that potently inhibits ROS1/TRK/ALK solvent-front mutations. *Cancer Discov.* **8**, 1227–1236 (2018). 30093503 10.1158/2159-8290.CD-18-0484

[11] Augtyro (repotrectinib). Package insert. (Bristol Myers Squibb, 2024).

[12] US Food and Drug Administration. FDA grants accelerated approval to repotrectinib for adult and pediatric patients with NTRK gene fusion-positive solid tumors. https://www.fda.gov/drugs/resources-information-approved-drugs/fda-grants-accelerated-approval-repotrectinib-adult-and-pediatric-patients-ntrk-gene-fusion-positive (2024).

[13] Non-small cell lung cancer (version 7.2024). in *NCCN Clinical Practice Guidelines in Oncology* (National Comprehensive Cancer Network, 2024). 10.6004/jnccn.2204.0023 38754467

[14] Hepatocellular carcinoma (version 2.2024). in *NCCN Clinical Practice Guidelines in Oncology* (National Comprehensive Cancer Network, 2024).

[15] Small bowel adenocarcinoma (version 4.2024). in *NCCN Clinical Practice Guidelines in Oncology* (National Comprehensive Cancer Network, 2024). 10.6004/jnccn.2019.0043 PMC10191182 31487687

[16] Biliary tract cancers (version 3.2024). in *NCCN Clinical Practice Guidelines in Oncology* (National Comprehensive Cancer Network, 2024). 10.6004/jnccn.2025.0042 40930144

[17] Soft tissue sarcoma (version 2.2024) in *NCCN Clinical Practice Guidelines in Oncology* (National Comprehensive Cancer Network, 2024). 10.6004/jnccn.2016.0078 27283169

[18] Breast cancer (version 4.2024) in N*CCN Clinical Practice Guidelines in Oncology* (National Comprehensive Cancer Network, 2024). 10.6004/jnccn.2024.0035 39019058

[19] Rectal cancer (version 3.2024). in *NCCN Clinical Practice Guidelines in Oncology* (National Comprehensive Cancer Network, 2024).

[20] Colon cancer (version 4.2024). in *NCCN Clinical Practice Guidelines in Oncology* (National Comprehensive Cancer Network, 2024). 10.6004/jnccn.2024.0029 38862008

[21] Drilon, A. et al. Repotrectinib in *ROS1* fusion-positive non-small-cell lung cancer. *N. Engl. J. Med.* **390**, 118–131 (2024). 38197815 10.1056/NEJMoa2302299 PMC11702311

[22] Eisenhauer, E. A. et al. New response evaluation criteria in solid tumours: revised RECIST guideline (version 1.1). *Eur. J. Cancer* **45**, 228–247 (2009). 19097774 10.1016/j.ejca.2008.10.026

[23] Drilon, A. et al. Efficacy of larotrectinib in *TRK* fusion-positive cancers in adults and children. *N. Engl. J. Med.* **378**, 731–739 (2018). 29466156 10.1056/NEJMoa1714448 PMC5857389

[24] Hong, D. S. et al. Larotrectinib long-term efficacy and safety in adult patients (pts) with tropomyosin receptor kinase (TRK) fusion cancer. *J. Clin. Oncol.* **41**, 3141 (2023).

[25] Dubois, S. et al. A phase 1/2, open-label study of repotrectinib in pediatric and young adult patients with advanced or metastatic malignancies harboring ALK, ROS1, or NTRK1-3 alterations. *Pediatr. Blood Cancer* **68**, e29349 (2021).34662504

[26] Orbach, D. et al. Spotlight on the treatment of infantile fibrosarcoma in the era of neurotrophic tropomyosin receptor kinase inhibitors: international consensus and remaining controversies. *Eur. J. Cancer* **137**, 183–192 (2020). 32784118 10.1016/j.ejca.2020.06.028

[27] Schouten, L. J., Rutten, J., Huveneers, H. A. & Twijnstra, A. Incidence of brain metastases in a cohort of patients with carcinoma of the breast, colon, kidney, and lung and melanoma. *Cancer* **94**, 2698–2705 (2002). 12173339 10.1002/cncr.10541

[28] Villano, J. L. et al. Incidence of brain metastasis at initial presentation of lung cancer. *Neuro Oncol.* **17**, 122–128 (2015). 24891450 10.1093/neuonc/nou099 PMC4483041

[29] Demetri, G. D. et al. Updated integrated analysis of the efficacy and safety of entrectinib in patients with *NTRK* fusion-positive solid tumors. *Clin. Cancer Res.* **28**, 1302–1312 (2022). 35144967 10.1158/1078-0432.CCR-21-3597 PMC9365368

[30] Hong, D. S. et al. Larotrectinib in patients with TRK fusion-positive solid tumours: a pooled analysis of three phase 1/2 clinical trials. *Lancet Oncol.* **21**, 531–540 (2020). 32105622 10.1016/S1470-2045(19)30856-3 PMC7497841

