# Supplementary material for: Repotrectinib in NTRK fusion–positive advanced solid tumors: a phase 1/2 trial
Source: Nat Med. 2026 Feb 4;32(2):682–9. doi: 10.1038/s41591-025-04079-7 (PMC12920079; doi:10.1038/s41591-025-04079-7)
Supplement: Supplementary file 1 — List of investigators and study sites, Supplementary methods, Supplementary Figs. 1 and 2, Supplementary Tables 1−15, Supplementary references, Redacted protocol, Redacted protocol amendment and Redacted statistical analysis plan. [file 41591_2025_4079_MOESM1_ESM.pdf]

nature medicine

Supplementary information https://doi.org/10.1038/s41591-025-04079-7

Repotrectinib in *NTRK* fusion–positive advanced solid tumors: a phase 1/2 trial

In the format provided by the authors and unedited

SUPPLEMENTARY APPENDIX

Table of Contents

LIST OF INVESTIGATORS AND STUDY SITES 3

SUPPLEMENTARY METHODS 7

Institutional Review Boards (IRBs) and Committees that Reviewed the Study Protocol 7

Eligibility Criteria for *NTRK*+ Cohorts 8

Biomarker Assay Method 10

Statistical Analysis 10

Pooling Criteria for Efficacy Outcomes Analysis 11

Patient-Reported Outcomes 13

SUPPLEMENTARY FIGURES AND TABLES 14

Figure S1. Tumor Type Distribution in Patients With NTRK+ Locally Advanced or Metastatic Solid Tumors. 14

Figure S2. Duration of Response in Patients Who Escalated to 160 mg BID and Had Subsequent Dose Reduction Due to Adverse Event Within 3 Months (Exploratory Analysis). 15

Table S1. Subsequent Therapies (Efficacy Population). 17

Table S2. Repotrectinib Efficacy Outcomes in TRK TKI-Naïve Patients With or Without Prior Treatment (Efficacy Population). 18

Table S3. Responses by *NTRK*+ Tumor Types (Efficacy Population). 20

Table S4. Repotrectinib Efficacy Outcomes by Sex in TRK TKI-Naïve and TRK TKI-Pretreated Patients (Efficacy Population). 23

Table S5. Repotrectinib Efficacy Outcomes in TRK TKI-Pretreated Patients Who Had Received Prior Entrectinib or Larotrectinib Therapy (Efficacy Population). 25

Table S6. Repotrectinib Efficacy Outcomes in TRK TKI-Pretreated Patients Who Received TKI as Most Recent Prior Therapy According to BICR (Efficacy Population). 26

Table S7. Repotrectinib Efficacy Outcomes in TRK TKI-Naïve and TKI-Pretreated Patients With Extended Follow-Up Who Were Treated With at Least One Dose of Repotrectinib......... 27

Table S8. Repotrectinib Efficacy Outcomes in TRK TKI-Naïve and TKI-Pretreated Patients With Extended Follow-Up Treated at the RP2D. 29

Table S9. Systemic Efficacy Outcomes of Repotrectinib in TRK TKI-Naïve and TRK TKI-Pretreated Patients With or Without Baseline Intracranial Disease According to BICR (Efficacy Population). 31

Table S10. *NTRK* Solvent Front Mutations at Baseline (Efficacy Population). 33

Table S11. Repotrectinib Efficacy Outcomes in TRK TKI-Pretreated Patients With *NTRK*+ Locally Advanced or Metastatic Solid Tumors and *NTRK* Solvent Front Mutation at Baseline (Efficacy Population). 34

Table S12. Emergent *NTRK* Solvent Front Mutations in Patients Who Discontinued Repotrectinib Due to Disease Progression or Death (Efficacy Population). 36

Table S13. Characterization of Patients With Disease Progression as Best Overall Response. 37

Table S14. Characteristics of Patients at Baseline (Safety Population). 40

Table S15. Adverse Events by TKI-Naïve and TKI-Pretreated Cohort.. 42

REFERENCES 45

LIST OF INVESTIGATORS AND STUDY SITES

Country	Investigators	Institution
Australia	Kao, Steven	Chris O'Brien Lifehouse
	Karapetis, Christos	Flinders Medical Centre
	Solomon, Benjamin	Peter MacCallum Cancer Centre
Belgium	Dooms, Christopher	Universitair Ziekenhuis Leuven
	Prenen, Hans	University Hospital Antwerp
Canada	Cheema, Parneet	William Osler Health System
	Chu, Quincy	Cross Cancer Institute
	Liu, Geoffrey	Princess Margaret Cancer Centre
	Wheatley-Price, Paul	The Ottawa Hospital
China	Cheng, Ying	Jilin Cancer Hospital/Medical Oncology Department
	Dong, Xiaorong	Union Hospital Affiliated with Tongji Medical College of Huazhong University of Science and Technology
	Fang, Jian	Beijing Cancer Hospital/Oncology Department
	He, Yong	Daping Hospital, The Third Affiliated Hospital of Third Military Medical University/Cancer Center
	Hu, Chunhong	The Second Xiangya Hospital of Central South University/Oncology Department
	Hu, Xiufeng	Henan Cancer Hospital/The 1st Pneumonology Department
	Jiang, Liyan	Shanghai Chest Hospital/Department of Respiratory Medicine
	Li, Junling	Cancer Hospital Chinese Academy of Medical Sciences/Cancer Institute
	Lin, Gen	Fujian Provincial Cancer Hospital/Thoracic Oncology Department
	Lu, Shun	Shanghai Chest Hospital/Oncology Department
	Luo, Feng	West China Hospital, Sichuan University/Lung Cancer Center
	Miao, Liyun	Nanjing Drum Tower Hospital/Oncology Department
	Shi, Huaqiu	The First Affiliated Hospital of Gannan Medical University/Oncology Department
	Su, Haichuan	Tangdu Hospital, Air Force Medical University/ Oncology Department
	Sun, Meili	Jinan Central Hospital/Medical Oncology Department
	Wang, Xiang	Xuzhou Central Hospital/Oncology Department
	Wu, Jingxun	The First Affiliated Hospital of Xiamen University/Medical Oncology Department
	Yang, Jinji	Guangdong Provincial People Hospital/Department 1 of Lung Disease
	Yang, Nong	Hunan Cancer Hospital/Medical Oncology Department
	Yao, Wenxiu	Sichuan Cancer Hospital/Medical Oncology Department

	Ying, Kejing	Sir Run Run Shaw Hospital, Zhejiang University School of Medicine/Oncology Department
	Yu, Yan	The Affiliated Tumor Hospital of Harbin Medical University/Medical Oncology Department
	Zhang, Yiping	Zhejiang Cancer Hospital/Thoracic Oncology Department
	Zhou, Jianying	The First Affiliated Hospital Zhejiang University School of Medicine/Respiratory Medicine Department
Denmark	Rohrberg, Kristoffer	Rigshospitalet, University Hospital of Copenhagen
France	Besse, Benjamin	Institut Gustave Roussy
	Greillier, Laurent	Hôpital de la Timone
	Hervieu, Alice	Centre Georges François Leclerc
	Isambert, Nicolas	Centre Hospitalier Universitaire de Poitiers
	Moro-Sibilot, Denis	Centre Hospitalier Universitaire Grenoble Alpes (Chuga)
Germany	Grohe, Christian	Evangelische Lungenklinik Berlin
	Springfeld, Christoph	University Hospital Heidelberg
	Wermke, Martin	University Clinic Carl Gustav Carus
	Wolf, Jürgen	Centrum für Integrierte Onkologie - Universitaetsklinikum Koeln
Hong Kong	Ho, James	Queen Mary Hospital
	Li, Jacky	Hong Kong United Oncology Centre
	Loong, Herbert	Prince of Wales Hospital
Hungary	Muller, Veronika	Semmelweis Egyetem
Italy	Bearz, Alessandra	Centro di Riferimento Oncologico
	Bracarda, Sergio	Azienda Ospedaliera Santa Maria di Terni
	Cappuzzo, Federico	IRCCS Istituto Regina Elena Oncologia Medica 1
	Pagano, Maria	Arcispedale Santa Maria Nuova
	Prelaj, Arsela	Fondazione IRCCS Istituto Nazionale dei Tumori
Japan	Goto, Koichi	National Cancer Center Hospital East
	Goto, Yasushi	National Cancer Center Hospital
	Kato, Terufumi	Kanagawa Cancer Center
	Nogami, Naoyuki	Ehime University Hospital
	Sakakibara, Jun	Hokkaido University Hospital
	Tamiya, Motohiro	Osaka International Cancer Institute
Korea	Cho, Byoung Chul	Yonsei University Health System, Severance Hospital
	Hong, Jung Yong	Samsung Medical Center
	Kang, Jin-Hyoung	Seoul Saint Mary's Hospital
	Kim, Dong-Wan	Seoul National University Hospital
	Kim, Sang-We	Asan Medical Center
	Kim, Young-Chul	Chonnam National University Hwasun Hospital
	Lee, Ki Hyeong	Chungbuk National University Hospital

Netherlands	de Langen, Joop	Netherlands Cancer Institute
	van der Wekken, Anthonie	Universitair Medisch Centrum Groningen
Poland	Dziadziuszko, Rafal	Ośrodek Badań Klinicznych Wczesnych Faz, Uniwersyteckie Centrum Kliniczne
	Kowalski, Dariusz	Klinika Nowotworow Pluca i Klatki Piersiowej - Narodowy Instytut Onkologii im. Marii Skłodowskiej-Curie
	Krawczyk, Pawel	Instytut Genetyki i Immunologii GENIM Sp. Z.o.o
Singapore	Lim, Darren	National Cancer Centre Singapore
	Soo, Ross Andrew	National University Hospital
Spain	Aguilar, Andres	Hospital Universitario Dexeus - Grupo Quironsalud
	Felip, Enriqueta	Hospital Universitario Vall d'Hebron
	Grande, Enrique	Fundación MD Anderson Cancer Center España
	Guerrero, Angel	Instituto Valenciano de Oncología
	Moreno, Irene	Hospital HM Sanchinarro
	Moreno, Victor	START Madrid-FJD
	Paz-Ares, Luis	Hospital Universitario 12 de Octubre
Taiwan	Yang, Chih-Hsin	National Taiwan University Hospital
	Yen, Chia-Jui	National Cheng Kung University Hospital
United Kingdom	Fontana, Elisa	Sarah Cannon Research Institute
	Krebs, Matthew	The Christie NHS
	Pinato, David	Imperial College London - Hammersmith
	Popat, Sanjay	The Royal Marsden - NHS Foundation Trust
United States of America	Adhami, Faisal	TriHealth Cancer Institute
	Anderson, Ian	St. Joseph Heritage Healthcare
	Anderson, Peter	Cleveland Clinic
	Awad, Mark	Dana-Farber Cancer Institute
	Baik, Christina	University of Washington, Seattle
	Bauman, Jessica	Fox Chase Cancer Center
	Bazhenova, Lyudmila	UC San Diego Moores Cancer Center
	Bestvina, Christine	University of Chicago
	Camidge, Ross	University of Colorado Denver
	Creelan, Benjamin	Moffitt Cancer Center
	Drilon, Alexander	Memorial Sloan Kettering Cancer Center
	Dudek, Arkadiusz	Regions Hospital Cancer Care Center
	Elamin, Yasir	MD Anderson Cancer Center
	Gadgeel, Shirish	Henry Ford Health System
	Gerstner, Greg	Illinois Cancer Care
	Kasbari, Samer	Southeastern Medical Oncology Center
	Kazmi, Syed	University of Texas Southwestern Medical Center

	Lammers, Philip	Baptist Memorial Hospital
	Levy, Benjamin	Sidney Kimmel Comprehensive Cancer Center at Johns Hopkins
	Lin, Jessica	Massachusetts General Hospital
	Liu, Stephen	Georgetown University Hospital
	Nagasaka, Misako	University of California Irvine Medical Center
	Nikolinakos, Petros	University Cancer & Blood Center
	Owen, Dwight	The Ohio State University Wexner Medical Center
	Pippas, Andrew	Columbus Regional Research Institute
	Raez, Luis	Memorial Healthcare System
	Scilla, Katherine	University of Maryland School of Medicine
	Shirinian, Mihran	Glendale Adventist Health
	Spira, Alexander	Virginia Cancer Specialists
	Tchekmedyian, Nishan	City of Hope, Long Beach (Elm)
	Uprety, Dipesh	Karmanos Cancer Institute
	Van Tine, Brian	Washington University in St. Louis
	Velcheti, Vamsidhar	NYU Perlmutter Cancer Center
	Weiss, Matthias	ThedaCare

SUPPLEMENTARY METHODS

Institutional Review Boards (IRBs) and Committees that Reviewed the Study Protocol

UC Irvine IRB, Memorial Sloan Kettering IRB, WCG IRB (previously WIRB), Dana-Farber Cancer Institute IRB, University of Michigan Medical School IRB, IRB of the Cleveland Clinic, Johns Hopkins Medicine IRB, Advarra IRB, UC San Diego Human Research Protections Program, NYU School of Medicine IRB, The University of Toledo - Human Research Protection Program - Cancer Biomedical IRB, The University of Chicago IRB, UT Southwestern Medical Center IRB, HealthPartners Institute IRB, MD Anderson Cancer Center Office of Protocol Research (OPR), University Health Network Research Ethics Board, HREBA Cancer Committee, William Osler Health System Research Ethics Board, Ottawa Health Science Network Research Ethics Board (OHSN-REB), UBC BC Cancer Research Ethics Board, Seoul National University Hospital, Yonsei Severence Hospital, Samsung Medical Center, Seoul St. Mary's Hospital IRB, Asan Medical Center IRB, Chungbuk National University Hospital, Konkuk University Medical Center IRB, Chonnam National University Hwasun Hospital, Peter MacCallum Cancer Centre, Ceim Instituto de Investigación Sanitaria La Fe, Comite de Protection des Personnes Sud Mediterranee III, Comitato Etico Territoriale Lombardia 4, London - Fulham Research Ethics Committee, Medisch Ethische Toetsingscommissie UMCG, Niezależna Komisja Bioetyczna do Spraw Badań Naukowych przy Gdańskim Uniwersytecie Medycznym, Ethikkommission der Medizinischen Fakultät der Universität zu Köln (Lead), Ethik-Kommission der Medizinischen Fakultät Heidelberg, Ethikkommission der Technischen Universität Dresden, Landesamt für Gesundheit und Soziales, (CEC) Ethics Committee Research UZ/KU Leuven, (LEC) Ethisch comité UZA/UA Antwerpen, De Videnskabsetiske Komiteer for Region Hovedstaden, Medical Research Council Ethics Committee for Clinical Pharmacology, Research Ethics Committee D of National Taiwan University Hospital, MacKay Memorial Hospital Institutional Review Board, National Cheng Kung University Hospital IRB,

DSRB, IRB of the University of Hong Kong/Hospital Authority Hong Kong West Cluster, Kowloon West Cluster Research Ethics Committee (KWC-REC), Joint Chinese University of Hong Kong - New Territories East Cluster Clinical Research Ethics Committee, and Hong Kong Oncology Study Group Research Ethics Committee.

Eligibility Criteria for *NTRK*+ Cohorts

Patients were considered eligible for inclusion in the *NTRK*+ locally advanced or metastatic solid tumor phase 2 expansion cohorts of the study if they met all the following criteria:

1. Locally advanced (defined as stage III when patient is not a candidate for surgery, radiation, or multi-modality therapy) or metastatic (defined as stage IV per the American Joint Committee on Cancer Eighth Edition Cancer Staging Manual guidelines) solid tumor harboring *NTRK1-3* gene fusions
2. Documented *NTRK1-3* gene fusion determined by any of the following tissue-based local testing:
    a. Next generation sequencing (NGS) or quantitative polymerase chain reaction
        i. Adequate tumor tissue needed to be retrospectively confirmed by a Sponsor-approved central diagnostic laboratory test
        ii. Partner of the fusion target gene needed to be identified if NGS was used
        iii. Retrospective confirmation by a Sponsor-approved central diagnostic laboratory was not required if *NTRK1-3* gene fusion was determined by the repotrectinib clinical trial assay
    b. FISH test and prospective confirmation of *NTRK1-3* gene fusion by a Sponsor-approved central diagnostic laboratory test prior to enrollment
3. Had the following performance score based on age at the time of enrollment:
    a. ECOG performance score of 0 to 1 for patients 18 years of age or older
    b. Karnofsky score of at least 50 for patients 16 to 18 years of age

    c. Lansky score of at least 50 for patients less than 16 years of age
4. At least 12 years of age (or as required by local regulations)
5. Provided written informed consent
6. At least one measurable target lesion by RECIST v1.1 prospectively confirmed by BICR, including CNS-only measurable disease of at least 10 mm according to RECIST v1.1
7. Patients with *NTRK*+ locally advanced or metastatic solid tumors were assigned into 2 distinct expansion cohorts provided all inclusion and exclusion criteria were met
    a. Cohort 5: TRK TKI-naïve
        i. No prior exposure to TRK TKI was allowed
        ii. Any number of prior lines of chemotherapy or immunotherapy were allowed
    b. Cohort 6: TRK TKI-pretreated
        i. Disease progression or intolerant to 1 or 2 prior TRK TKIs
        ii. TRK TKIs used in prior lines of treatment are limited to: entrectinib, larotrectinib, selitrectinib (LOXO-195), and cabozantinib. Other prior TRK TKIs that are not listed may be allowed after discussion with the Sponsor Medical Monitor. Note: Any previous exposure to a TRK TKI is considered as one prior line of TKI treatment, e.g., if 2 different TRK TKIs are used or the same TRK TKI was used before and after a chemotherapy or other systemic therapy, it is considered as 2 prior TKIs and the patient would be eligible
        iii. Any number of prior lines of chemotherapy or immunotherapy were allowed
8. Wash-out time related to prior therapies before starting repotrectinib treatment consistent with phase 1

9. Criteria for asymptomatic CNS metastases, treated or untreated, and/or asymptomatic leptomeningeal carcinomatosis consistent with phase 1

Biomarker Assay Method

NTRK resistance mutations were identified by either local tissue-based or central plasma-based NGS using Guardant360 CDx, or GeneseeqLite (for subjects enrolled in China) circulating tumor DNA (ctDNA)–based assays.

Statistical Analysis

Data were collected from patients by study investigators and study staff at each study site. Clinical data from TRIDENT-1 were collected using Medidata Classic Rave 2023.2.0. Outcomes were analyzed descriptively, and plots were generated and analyzed using SAS 9.4.

For each extension cohort, treatment outcomes and safety were evaluated in all patients who received at least 1 dose of repotrectinib. Two-sided exact 95% binomial CIs were computed for binary endpoints (eg, ORR). Time-to-event endpoints (eg, PFS), analyzed using Kaplan–Meier methods, are expressed as medians and milestone timepoint estimates with corresponding 95% CIs. Sample size calculations, prespecified subgroup analyses, and definitions for time-to-event outcomes are described in the Statistical Analysis Protocol (SAP).

For phase 2, 55 TRK TKI-naïve patients with *NTRK*+ (*NTRK1*, *NTRK2*, and *NTRK3*) solid tumors were to be enrolled in the TKI-naïve expansion cohort. If the ORR is ≤35%, repotrectinib is not considered effective. If 27 out of 55 patients have a confirmed objective response (ORR, 49.1%; 95% CI, 35.4–62.9) where the lower limit of the 95% CI is >35%, repotrectinib will be considered efficacious in this expansion cohort. After enrollment of 55 patients as specified for the primary analysis, an additional 25 patients are to be enrolled for a total of approximately 80 subjects in the TKI-naïve cohort.

For the TRK TKI-pretreated cohort, 40 TRK TKI-pretreated patients with *NTRK*+ solid tumors were to be enrolled with 1 or 2 prior TRK TKI treatments. In this cohort, if the ORR is ≤10%, then it is assumed that repotrectinib is not effective. If 9 out of 40 patients have a confirmed objective response (ORR, 22.5%; 95% CI, 10.8–38.5) where the lower limit of 95% CI is >10%, repotrectinib will be considered efficacious in this cohort. After enrollment of 40 patients in this cohort for the primary analysis, an additional 80 patients are to be enrolled, for a total of approximately 120 patients in the TKI-pretreated cohort.

In an exploratory analysis, duration of response (DOR) was assessed in patients who initially dose escalated to BID and subsequently had a dose reduction due to an adverse event (AE; **Fig. S10**). The calculations were performed using a 3-month landmark analysis to mitigate immortal time bias. Results should be interpreted with caution due to the exploratory nature of this analysis, which may be impacted by confounding factors.

Pooling Criteria for Efficacy Outcomes Analysis

The efficacy outcomes analysis for the TRK TKI-naïve solid tumor cohort included all patients with *NTRK*+ metastatic/locally advanced solid tumors in the phase 1 and phase 2 portions of the TRIDENT-1 study who met the following criteria:

- Confirmation by central laboratory (Almac) test for *NTRK* rearrangement per the requirement in each phase of the TRIDENT-1 study protocol
    - In phase 1, central confirmation of gene fusion status was not required for enrollment. However, patients with *NTRK*+ solid tumors based on local FISH test were retested retrospectively by central laboratory. Only subjects with confirmed *NTRK* positivity by central laboratory were included in the integrated efficacy outcomes analysis, in addition to patients with *NTRK*+ locally advanced or metastatic solid tumors tested locally by NGS or qPCR.

    - In phase 2, subjects entering with local results by FISH must have had gene fusion status confirmed prospectively prior to enrollment
- No prior exposure to a TRK TKI
- Started treatment at least 8 months prior to data cutoff date (6 months of follow-up for tumor assessment after first post-baseline scan)

The efficacy outcomes analysis for the TRK TKI-pretreated solid tumor cohort included all patients with *NTRK*+ locally advanced or metastatic solid tumors in the phase 1 and phase 2 portions of the TRIDENT-1 study who met the following criteria:

- Confirmation by central laboratory (Almac) test for *NTRK* rearrangement per the requirement in each phase of the TRIDENT-1 study protocol
    - In phase 1, central confirmation of gene fusion status was not required for enrollment. However, patients with *NTRK*+ solid tumors based on local FISH test were retested retrospectively by central laboratory. Only patients with confirmed *NTRK* positivity by central laboratory were included in the integrated efficacy outcomes analysis, in addition to patients with *NTRK*+ locally advanced or metastatic solid tumors tested locally by NGS or qPCR
    - In phase 2, subjects entering with local results by FISH must have had gene fusion status confirmed prospectively prior to enrollment
- Disease progression or intolerant to at least 1 prior line of a TRK TKI treatment, including entrectinib, larotrectinib, selitrectinib (LOXO-195), and cabozantinib. Other prior TRK TKIs that are not listed may be allowed after discussion with the Sponsor Medical Monitor.
- Started treatment at least 8 months prior to data cutoff date (6 months of follow-up for tumor assessment after first post-baseline scan)

Patient-Reported Outcomes

Patient-reported outcomes were assessed using the European Organisation for Research and Treatment Cancer Quality of Life Questionnaire–Core 30 (EORTC QLQ-C30). The EORTC QLQ-C30 is a 30-item questionnaire that incorporate of 9 multi-item scales: 5 functional scales (Physical, Role, Cognitive, Emotional, and Social), 3 symptom scales (Fatigue, Pain, and Nausea and Vomiting), and a Global Health Status/Quality of Life (GHS/QOL) scale. Several single-item symptom measures are also included (Dyspnea, Insomnia, Appetite, Constipation, Diarrhea, and Financial Impact).[1] Patients assessed how true each statement in the questionnaire was for them on a 4-point scale (1 = not at all, 2 = a little, 3 = quite a bit, 4 = very much), whereas GHS/QOL was assessed using a 7-point Likert scale (ranging from “poor” to “excellent”) for 2 items. The scales and items are linearly transformed to a 0 to 100 score according to the specifications in the questionnaire scoring manual. For GHS/QOL and functional scales, higher scores represent higher or better levels of functioning or quality of life (QOL); for symptom scales and items, higher scores represent increased levels of symptoms burden and worse outcomes. A 10-point change in an item or domain score between baseline and each follow-up assessment (on Day 1 of each 28-day treatment cycle and within 7 days after treatment discontinuation) is considered a clinically meaningful within-patient change.[2]

SUPPLEMENTARY FIGURES AND TABLES

Figure S1. Tumor Type Distribution in Patients With NTRK+ Locally Advanced or Metastatic Solid Tumors. TRK TKI-naïve cohort (A) and TRK TKI-pretreated cohort (B).[a]

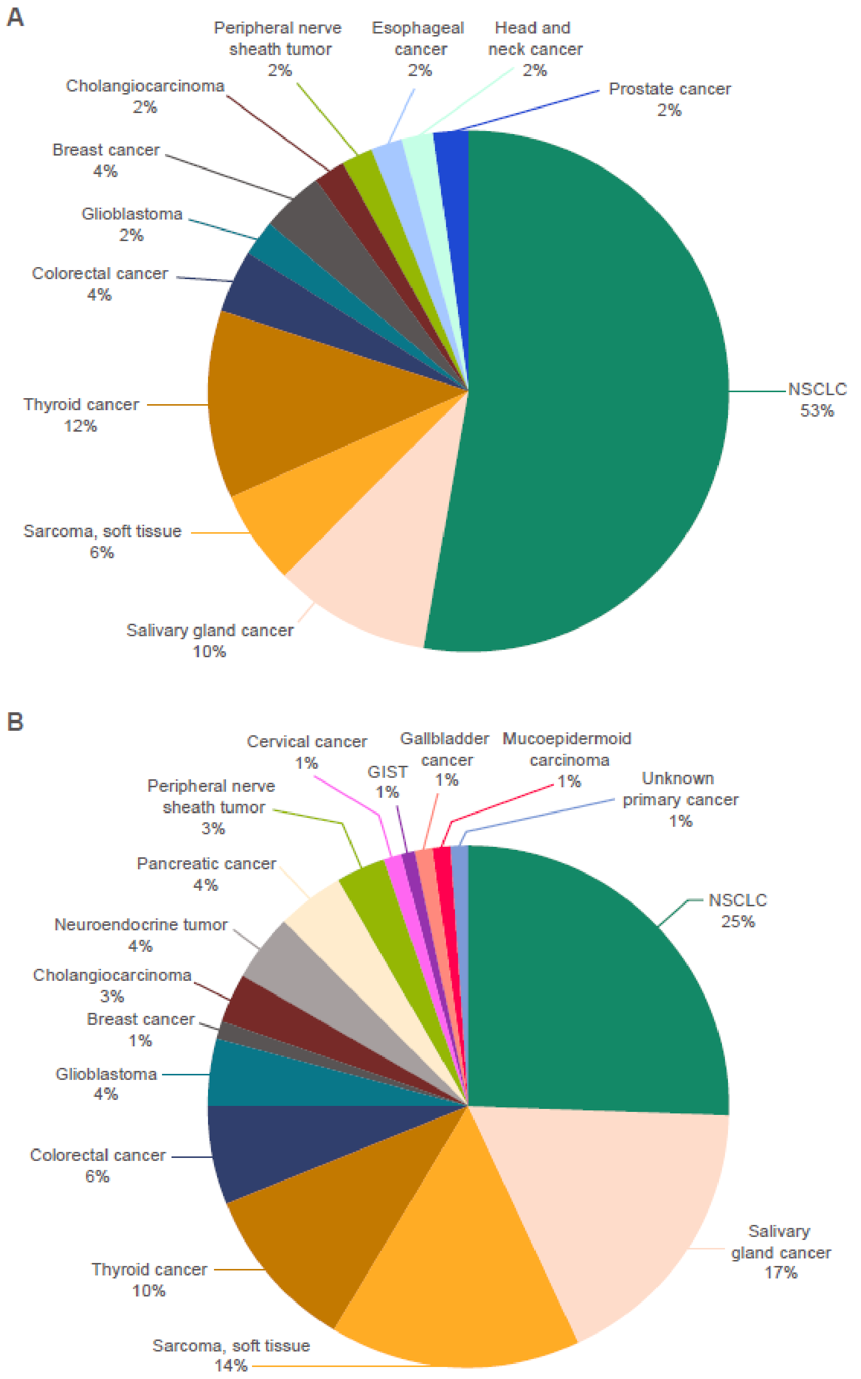

[a]Patients with asymptomatic leptomeningeal carcinomatosis were eligible, though none enrolled.

Figure S2. Duration of Response in Patients Who Escalated to 160 mg BID and Had Subsequent Dose Reduction Due to Adverse Event Within 3 Months (Exploratory Analysis).[a] TRK TKI-naïve cohort (A) and TRK TKI-pretreated cohort (B). NR indicates that the median was not reached.

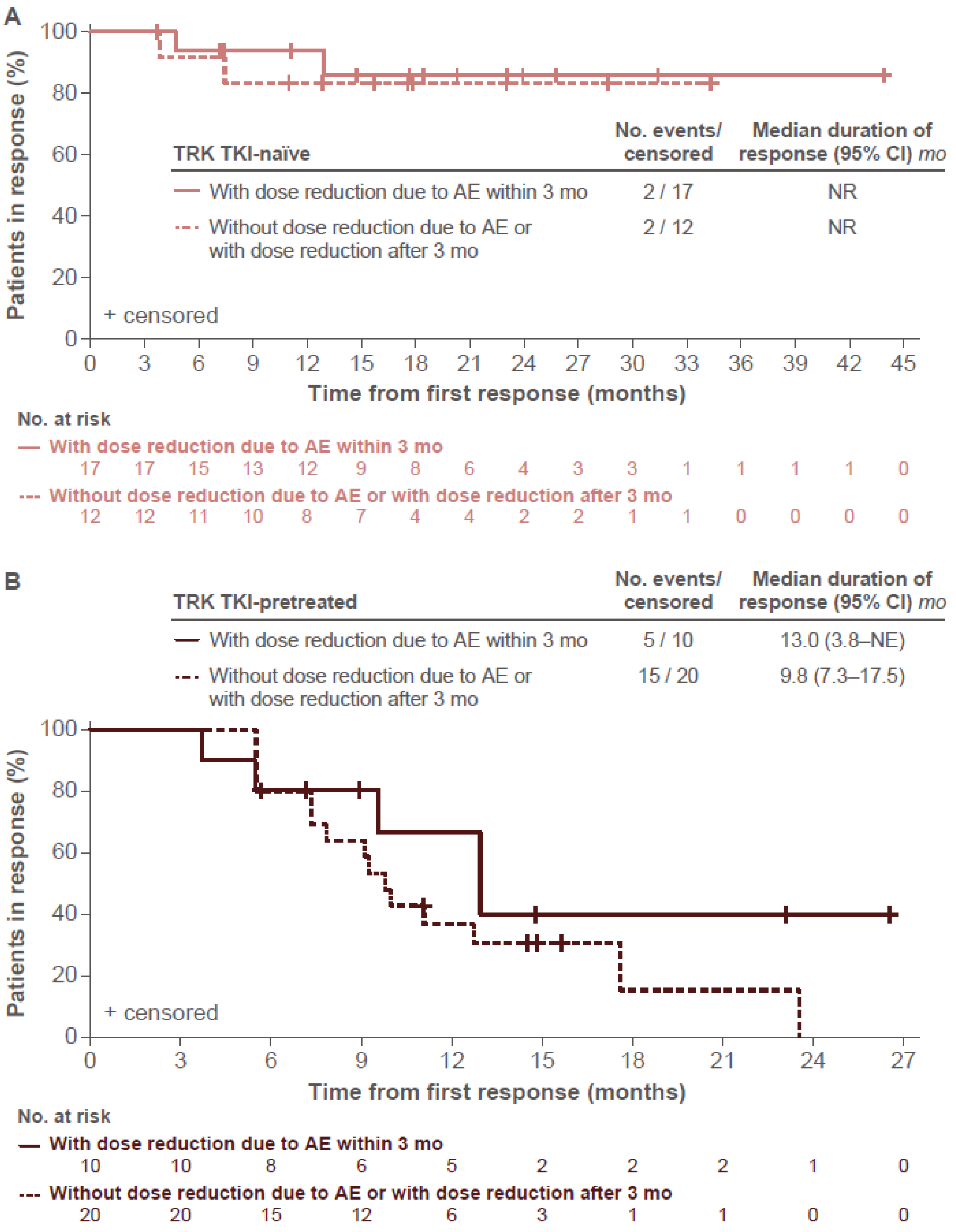

[a]The calculations were performed using a 3-month landmark analysis to mitigate immortal time bias. Results should be interpreted with caution due to the exploratory nature of this analysis, which may be impacted by confounding factors.

Table S1. Subsequent Therapies (Efficacy Population).

	**TRK TKI-naïve (n = 51)**	**TRK TKI-pretreated (n = 69)**
**Patients who discontinued repotrectinib — no. (%)**	22 (43)	56 (81)
**Type of first subsequent anticancer therapy — no. (%)[a]**		
TRK TKI – single agent	4 (18)	6 (11)
TRK TKI with chemotherapy	0	0
Chemotherapy alone	1 (5)	13 (23)
Immunotherapy alone	3 (14)	1 (2)
Other targeted therapy	3 (14)	1 (2)
Not reported[b]	10 (45)	33 (59)

[a]Percentages are based on number of patients who discontinued repotrectinib treatment. Each patient only counted once.

[b]Includes patients who did not receive subsequent therapy or with unknown status.

Table S2. Repotrectinib Efficacy Outcomes in TRK TKI-Naïve Patients With or Without Prior Treatment (Efficacy Population).

	TKI-naïve with no prior treatment (n = 18)	TKI-naïve with prior treatment (n = 33)
**Confirmed objective response rate[a]**		
No. patients in response	11	19
Percent (95% CI)	61 (36–83)	58 (39–75)
**Best overall response — no. (%)[a]**		
Complete response	4 (22)	4 (12)
Partial response	7 (39)	15 (45)
Stable disease	6 (33)	7 (21)
Progressive disease	0	5 (15)
Not evaluable	0	0
**Median duration of response (95% CI) — mo[a]**	NE (12.9–NE)	NE (NE–NE)
12-month duration of response — % (95% CI)	90 (71–100)	89 (74–100)
Events — no.	2	2
Censored — no. (%)	9 (82)	17 (90)
**Median progression-free survival (95% CI) — mo[a]**	NE (5.5–NE)	30.3 (6.6–NE)
12-month progression-free survival — % (95% CI)	65 (42–88)	62 (45–79)
Events — no.	7	13

Censored — no. (%)	11 (61)	20 (61)

[a]Per RECIST v1.1

Table S3. Responses by *NTRK*+ Tumor Types (Efficacy Population).

	TRK TKI-naïve (n = 51)	TRK TKI-pretreated (n = 69)
**NSCLC**		
No. patients	27	17
Objective response rate — no.[a]	17	9
Percent (95% CI)	63 (42–81)	53 (28–77)
**Thyroid cancer**		
No. patients	6	7
Objective response rate — no.[a]	6	2
Percent (95% CI)	100 (54–100)	29 (4–71)
**Salivary gland cancer**		
No. patients	5	12
Objective response rate — no.[a]	4	9
Percent (95% CI)	80 (28–100)	75 (43–94)
**Soft tissue sarcoma**		
No. patients	3	10
Objective response rate — no.[a]	1	1
Percent (95% CI)	33 (1–91)	10 (0–44)
**Breast cancer**		
No. patients	2	1
Objective response rate — no.[a]	0	1
Percent (95% CI)	0 (0–84)	100 (2–100)
**Colorectal cancer**		
No. patients	2	4

Objective response rate — no.[a]	1	2
Percent (95% CI)	50 (1–99)	50 (7–93)
**Cholangiocarcinoma**		
No. patients	1	2
Objective response rate — no.[a]	0	1
Percent (95% CI)	0 (0–98)	50 (1–99)
**Glioblastoma**		
No. patients	1	3
Objective response rate — no.[a]	0	1
Percent (95% CI)	0 (0–98)	33 (1–91)
**Peripheral nerve sheath tumor**		
No. patients	1	2
Objective response rate — no.[a]	1	2
Percent (95% CI)	100 (2–100)	100 (16–100)
**Neuroendocrine tumor**		
No. patients	0	3
Objective response rate — no.[a]	0	3
Percent (95% CI)	NA	100 (29–100)
**Pancreatic cancer**		
No. patients	0	3
Objective response rate — no.[a]	0	0
Percent (95% CI)	NA	0 (0–71)
**Other[b]**		
No. patients	3	5
Objective response rate — no.[a]	0	2

Percent (95% CI)	0 (0–71)	40 (5–85)

[a]By RECIST v1.1.

[b]Other tumors included cervical cancer, esophageal cancer, gastrointestinal stromal tumor, gallbladder cancer, head and neck cancer, mucoepidermoid carcinoma, prostate cancer, and unknown primary cancer.

Table S4. Repotrectinib Efficacy Outcomes by Sex in TRK TKI-Naïve and TRK TKI-Pretreated Patients (Efficacy Population).

	TRK TKI-naïve		TRK TKI-pretreated	
	Male (n = 24)	Female (n = 27)	Male (n = 36)	Female (n = 33)
**Confirmed objective response rate[a]**				
No. patients in response	13	17	19	14
Percent (95% CI)	54 (33–74)	63 (42–81)	53 (36–70)	42 (26–61)
**Best overall response — no. (%)[a]**				
Complete response	3 (13)	5 (19)	2 (6)	0
Partial response	10 (42)	12 (44)	17 (47)	14 (42)
Stable disease	8 (33)	5 (19)	6 (17)	10 (30)
Progressive disease	1 (4)	4 (15)	5 (14)	8 (24)
Not evaluable	0	0	3 (8)	0
**Median duration of response (95% CI) — mo[a]**	NE (12.9–NE)	NE (NE–NE)	9.8 (7.4–NE)	9.6 (5.5–17.5)
12-month duration of response — % (95% CI)	92 (76–100)	88 (71–100)	49 (26–73)	30 (4–56)

Events — no.	2	2	13	10
Censored — no. (%)	11 (85)	15 (88)	6 (32)	4 (29)
**Median progression-free survival (95% CI) — mo[a]**	30.3 (5.5–NE)	NE (6.6–NE)	8.6 (5.4–11.4)	5.8 (3.5–9.1)
12-month progression-free survival — % (95% CI)	65 (45–85)	62 (44–81)	31 (15–47)	20 (5–35)
Events — no.	10	10	27	25
Censored — no. (%)	14 (58)	17 (63)	9 (25)	8 (24)
**Median overall-free survival (95% CI) — mo**	-[b]	-[b]	25.7 (10.8–NE)	16.8 (11.6–19.1)
12-month overall-free survival — % (95% CI)	83 (68–98)	78 (62–94)	60 (43–77)	64 (46–82)
Events — no.	9	8	15	20
Censored — no. (%)	15 (63)	19 (70)	21 (58)	13 (39)

[a]Per BICR.

[b]Median overall survival for TRK TKI-naïve cohort is not reported due to data immaturity.

Table S5. Repotrectinib Efficacy Outcomes in TRK TKI-Pretreated Patients Who Had Received Prior Entrectinib or Larotrectinib Therapy (Efficacy Population).

	Prior entrectinib (n = 32)	Prior larotrectinib (n = 36)
**Confirmed objective response rate**[a]		
No. patients in response	18	14
Percent (95% CI)	56 (38–74)	39 (23–57)
**Best overall response — no. (%)**[a]		
Complete response	0	2 (6)
Partial response	18 (56)	12 (33)
Stable disease	6 (19)	11 (31)
Progressive disease	7 (22)	6 (17)
Not evaluable	0	3 (8)
**Median duration of response (95% CI) — mo**[a]	12.8 (9.2–17.5)	6.4 (5.5–NE)
12-month duration of response — % (95% CI)	52 (28–76)	33 (8–59)
Events — no.	12	10
Censored — no. (%)	6 (33)	4 (29)
**Median progression-free survival (95% CI) — mo**[a]	9.0 (5.8–11.9)	5.5 (3.7–9.0)
12-month progression-free survival — % (95% CI)	30 (14–47)	23 (7–38)
Events — no.	25	26
Censored — no. (%)	7 (22)	10 (28)

[a]By RECIST v1.1.

Table S6. Repotrectinib Efficacy Outcomes in TRK TKI-Pretreated Patients Who Received TKI as Most Recent Prior Therapy According to BICR (Efficacy Population).

	**TRK TKI-pretreated patients who received TKI as most recent prior therapy (n = 63)[a,b]**
**Best overall response — no. (%)**	
Complete response	1 (2)
Partial response	29 (46)
Stable disease	14 (22)
Progressive disease	12 (19)
Not evaluable	3 (5)
**TKI as most recent prior therapy — no. (%)**	
Entrectinib	25 (40)
Larotrectinib	28 (44)
Other[c]	10 (14)
**Median time since end of most recent prior TKI therapy — days (range)**	19 (2–1156)

[a]Of the 56 patients who discontinued their most recent prior TKI due to disease progression, one patient (2%) had complete response to repotrectinib, 26 (46%) had partial response, 11 (20%) had stable disease, 11 (20%) had progressive disease, and 3 (5%) were not evaluable.

[b]Among patients whose most recent prior therapy was TKI, the best responses to this TKI therapy were 3 patients (5%) with complete response, 35 (56%) with partial response, 10 (16%) with stable disease, and 9 (14%) with progressive disease.

[c]Other previous TKIs included selitrectinib (n = 6, 10%), cabozantinib (n = 1, 2%), PBI-200 (n = 1, 2%), taletrectinib (AB-106, n = 1, 2%), and VC004 with TRK activity (n = 1, 2%).

Table S7. Repotrectinib Efficacy Outcomes in TRK TKI-Naïve and TKI-Pretreated Patients With Extended Follow-Up Who Were Treated With at Least One Dose of Repotrectinib.[a]

	TRK TKI-naïve (n = 40)	TRK TKI-pretreated (n = 48)
**Confirmed objective response rate[b]**		
No. patients in response	23	24
Percent (95% CI)	58 (41–73)	50 (35–65)
**Best overall response — no. (%)[b]**		
Complete response	6 (15)	1 (2)
Partial response	17 (43)	23 (48)
Stable disease	9 (23)	12 (25)
Progressive disease	5 (13)	8 (17)
Not evaluable	0	2 (4)
**Median time to response (range) — mo**	1.8 (1.6–7.3)	1.9 (1.7–3.7)
**Median duration of response (95% CI) — mo[b]**	NE (NE–NE)	9.9 (7.4-–13.0)
12-month duration of response — % (95% CI)	86 (72–100)	42 (22–61)
24-month duration of response — % (95% CI)	82 (66–98)	–
Events — no.	4	19
Censored — no. (%)	19 (83)	5 (21)
**Median progression-free survival (95% CI) — mo[b]**	30.3 (5.5–NE)	7.4 (3.9–9.7)

12-month progression-free survival — % (95% CI)	56 (40–72)	25 (13–38)
24-month progression-free survival — % (95% CI)	53 (38–69)	–
Events — no.	19	39
Censored — no. (%)	21 (53)	9 (19)
**Median overall survival (95% CI) — mo**	–[c]	16.8 (9.6–22.3)
12-month overall survival — % (95% CI)	75 (62–88)	59 (45–74)
24-month overall survival — % (95% CI)	64 (48–79)	–
Events — no.	17	30
Censored — no. (%)	23 (58)	18 (38)

[a]Patients were treated prior to April 19, 2022. Median follow-up was 27.7 months for the TKI-naïve cohort and 29.9 months for the TKI-pretreated cohort.

[b]Per RECIST v1.1.

[c]Median overall survival for TRK TKI-naïve cohort is not reported due to data immaturity.

Table S8. Repotrectinib Efficacy Outcomes in TRK TKI-Naïve and TKI-Pretreated Patients With Extended Follow-Up Treated at the RP2D.[a,b]

	TRK TKI-naïve (n = 35)	TRK TKI-pretreated (n = 44)
**Confirmed objective response rate[c]**		
No. patients in response	21	23
Percent (95% CI)	60 (42–76)	52 (37–68)
**Best overall response — no. (%)[c]**		
Complete response	6 (17)	1 (2)
Partial response	15 (43)	22 (50)
Stable disease	8 (23)	10 (23)
Progressive disease	4 (11)	7 (16)
Not evaluable	0	2 (4)
**Median time to response (range) — mo**	1.8 (1.6–3.9)	1.9 (1.8–3.7)
**Median duration of response (95% CI) — mo**	NE (NE–NE)	10.0 (7.4–13.0)
12-month duration of response — % (95% CI)	90 (77–100)	44 (23–64)
24-month duration of response — % (95% CI)	85 (69–100)	–
Events — no.	3	18
Censored — no. (%)	18 (86)	5 (22)
**Median progression-free survival (95% CI) — mo**	30.3 (5.8–NE)	7.4 (3.9–9.7)

12-month progression-free survival — % (95% CI)	61 (45–78)	27 (14–41)
24-month progression-free survival — % (95% CI)	58 (42–75)	–
Events — no.	15	36
Censored — no. (%)	20 (57)	8 (18)
**Median overall survival (95% CI) — mo**	–[d]	16.8 (9.6–22.3)
12-month overall survival — % (95% CI)	77 (63–91)	58 (43–73)
24-month overall survival — % (95% CI)	67 (50–83)	–
Events — no.	13	30
Censored — no. (%)	22 (63)	14 (32)

[a]Patients were treated prior to April 19, 2022.

[b]The RP2D was repotrectinib 160 mg once daily for 14 days, then 160 mg twice daily.

[c]Per RECIST v1.1.

[d]Median overall survival for TRK TKI-naïve cohort is not reported due to data immaturity.

Table S9. Systemic Efficacy Outcomes of Repotrectinib in TRK TKI-Naïve and TRK TKI-Pretreated Patients With or Without Baseline Intracranial Disease According to BICR (Efficacy Population).

	**Patients with intracranial disease at baseline**		**Patients without intracranial disease at baseline**	
	**TRK TKI-naïve (n = 10)**	**TRK TKI-pretreated (n = 16)**	**TRK TKI-naïve (n = 41)**	**TRK TKI-pretreated (n = 53)**
**Confirmed objective response rate**				
No. patients in response	5	7	25	26
Percent (95% CI)	50 (19–81)	44 (20–70)	61 (45–76)	49 (35–63)
**Best overall response — no. (%)**				
Complete response	0	0	8 (20)	2 (4)
Partial response	5 (50)	7 (44)	17 (41)	24 (45)
Stable disease	3 (30)	4 (25)	10 (24)	12 (23)
Progressive disease	1 (10)	3 (19)	4 (10)	10 (19)
Not evaluable	0	1 (6)	0	2 (4)
**Median duration of response (95% CI) — mo**	12.9 (3.8–NE)	9.2 (5.5–NE)	NE (NE–NE)	11.1 (7.9–17.5)

12-month duration of response — % (95% CI)	60 (17–100)	19 (0–52)	96 (87–100)	47 (27–67)
Events — no.	3	6	1	17
Censored — no. (%)	2 (40)	1 (14)	24 (96)	9 (35)
**Median progression-free survival (95% CI) — mo**	6.1 (3.4–NE)	7.4 (2.8–11.0)	NE (30.3–NE)	7.4 (4.5–11.0)
12-month progression-free survival — % (95% CI)	40 (10–70)	9 (0–26)	69 (55–84)	30 (17–43)
Events — no.	7	13	13	39
Censored — no. (%)	3 (30)	3 (19)	28 (68)	14 (26)

Table S10. *NTRK* Solvent Front Mutations at Baseline (Efficacy Population).

	***NTRK* solvent front mutations at baseline in TRK TKI-pretreated cohort (n = 30)[a,b]**
***NTRK1* solvent front mutation — no. (%)**	5 (17)
NTRK1 G595R — no. (%)	5 (17)
***NTRK3* solvent front mutation — no. (%)**	25 (83)
NTRK3 G623R — no. (%)	20 (67)
NTRK3 G623L — no. (%)	2 (7)
NTRK3 G623E — no. (%)	8 (27)
NTRK3 G623V — no. (%)	2 (7)
NTRK3 G623K — no. (%)	1 (3)

[a]Among 41 patients in the TRK TKI-naïve cohort with available samples, no patients had solvent front mutations observed at baseline.

[b]Multiple mutations may be detected in an individual patient.

Table S11. Repotrectinib Efficacy Outcomes in TRK TKI-Pretreated Patients With *NTRK*+ Locally Advanced or Metastatic Solid Tumors and *NTRK* Solvent Front Mutation at Baseline (Efficacy Population).[a]

	**With *NTRK* solvent front mutation (n = 30)[b]**	**Without *NTRK* solvent front mutation (n = 37)[b]**
**Confirmed objective response rate[c]**		
No. patients in response	16	17
Percent (95% CI)	53 (34–72)	46 (30–63)
**Best overall response — no. (%)[c]**		
Complete response	0	2 (5)
Partial response	16 (53)	15 (40)
Stable disease	8 (27)	7 (19)
Progressive disease	6 (20)	7 (19)
Not evaluable	0	2 (5)
**Median time to response (range) — mo**	1.9 (1.7–3.7)	1.8 (1.7–3.6)
**Median duration of response (95% CI) — mo[c]**	8.6 (5.5–12.9)	12.8 (9.1–NE)
12-month duration of response — % (95% CI)	31 (9–54)	53 (27–79)
Events — no.	13	10
Censored — no. (%)	3 (19)	7 (41)
**Median progression-free survival (95% CI) — mo[c]**	7.4 (3.9–11.0)	7.4 (3.6–11.4)

12-month progression-free survival — % (95% CI)	22 (6–37)	32 (14–48)
Events — no.	25	25
Censored — no. (%)	5 (17)	12 (32)

[a]Among 4 patients in the TRK TKI-pretreated cohort with gatekeeper mutations, objective response rate was 25% (95% CI, 1–81); 1 patient had a partial response, 2 had stable disease, and 1 had progressive disease.

[b]Excludes 2 patients with unknown solvent front mutation status and 2 patients with solvent front mutations at baseline identified after the current data cutoff of October 15, 2023.

[c]Per RECIST v1.1.

Table S12. Emergent *NTRK* Solvent Front Mutations in Patients Who Discontinued Repotrectinib Due to Disease Progression or Death (Efficacy Population).

	**Patients who discontinued repotrectinib due to disease progression or death (n = 20)**	
	**TRK TKI-naïve (n = 2)[a]**	**TRK TKI-pretreated (n = 18)**
**Emergent *NTRK* solvent front mutations — no. (%)**	0	3 (17)[b]
No mutation at baseline — no. (%)	–	2 (11)[c]
Mutation at baseline — no. (%)	–	1 (6)[d]

[a]In the TRK TKI-naïve cohort, there were 2 patients who did not have baseline samples reported but did have end-of-treatment samples with no mutations detected.

[b]All emergent *NTRK* solvent front mutations were *NTRK3*.

[c]Of the 2 patients who had no mutation at baseline, 1 developed G623E and the other developed G623R at end of treatment.

[d]One patient had G623K at baseline that was not detected at end of treatment although an emerging G623E was identified.

Table S13. Characterization of Patients With Disease Progression as Best Overall Response.

Tumor type	Days from first dose to progression	Number of lines of prior systemic therapy	Number of lines of TKI treatment	Prior TKI	Select ctDNA results at screening	Select ctDNA results at end of treatment
**TKI-naïve patients with progressive disease (n = 5, 10%)**						
Cholangiocarcinoma	52	1	0	-	NA	NA
Breast cancer	27	1	0	-	*NRAS* G12A, *TP53* deletion	NA
Breast cancer	52	4	0	-	No mutations identified	No mutations identified
NSCLC	43	1	0	-	NA	NA
Soft tissue sarcoma	57	1	0	-	NA	NA
**TKI-pretreated patients with progressive disease (n = 13, 19%)**						
NSCLC	66	3	1	Entrectinib	NA	NA
Soft tissue sarcoma	43	2	1	Entrectinib	*NTRK1* G595R, *PIK3CA* M1043I	No mutations identified
Soft tissue sarcoma	52	1	1	Larotrectinib	*NTRK1* G667C	NA
Pancreatic cancer	54	5	1	Larotrectinib	*KRAS* amp,	Failed testing

					ERBB2 amp, *CDK4* amp, *ARID1A* deletion, *ATM* D2785N/R3008C	
Pancreatic cancer	54	2	1	Larotrectinib	*KRAS* amp, *PIK3CA* E542Q	*KRAS* amp
Salivary gland cancer	57	3	2	Larotrectinib, selitrectinib	*NTRK3* G623R/V/*, G696A, F617L	NA
Unknown primary cancer	34	2	1	Entrectinib	*NTRK1* G595R, *KRAS* Q61K, *BRAF* V600E, *BRCA1* P1562L, *PTEN* R173H, *ERBB2* R47H	*TP53* deletion, *BRAF* V600E, *ERBB2* R47H
NSCLC	51	4	2	Entrectinib, selitrectinib	*NTRK3* G623E, *PIK3CA* E545K, *SMAD4* R420C,	*SMAD4* R420C, *TP53* V217E

					TP53 V217E	
Thyroid cancer	52	3	1	Larotrectinib	*ERBB2* P489L	*ERBB2* P489L
Cervical cancer	57	2	1	Larotrectinib	*NTRK3* G623R,<br>*CCND1* amp,<br>*FGFR2* I447M	NA
NSCLC	54	2	1	Entrectinib	*NTRK1* G667C	*TP53* S33F
Cholangiocarcinoma	55	2	1	Entrectinib	*IDH* R172K	NA
Soft tissue sarcoma	50	1	1	Entrectinib	*CDK4* amp	*TP53* deletion

NA indicates that sample was not available.

Table S14. Characteristics of Patients at Baseline (Safety Population).

Characteristic	Safety population (N = 565)[a]
**Age**	
Median (range) — yr	56 (18–93)
Distribution — no. (%)	
≥18 to <65 yr	425 (75)
≥65 yr	140 (25)
**Female sex — no. (%)**	328 (58)
**Ethnicity — no. (%)**	
Hispanic or Latino	16 (3)
Not Hispanic or Latino	536 (95)
**Geographic region — no. (%)**	
United States	175 (31)
Asia	204 (36)
Other[b]	186 (33)
**ECOG performance status — no. (%)[c]**	
0	202 (36)
1	362 (64)
**Stage 4 metastatic disease — no. (%)**	539 (95)
**Intracranial Disease — no. (%)[d]**	
Yes	162 (29)
No[e]	403 (71)
**No. of previous lines of systemic therapy — no. (%)**	
0	107 (19)

1	189 (33)
2	157 (28)
≥3	112 (20)
**No. of previous lines of chemotherapy with or without immunotherapy — no. (%)**	
0	304 (54)
1	197 (35)
2	47 (8)
≥3	17 (3)
**No. of previous lines of TKI therapy — no. (%)**	
0	191 (34)
1	249 (44)
2	96 (17)
≥3	29 (5)

[a]The safety population consists of all patients treated with at least one dose repotrectinib, which includes 367 patients with *ROS1*+ NSCLC, 144 patients with *NTRK*+ solid tumors, and 54 other treated patients. Percentages may not total 100 because of rounding.

[b]Other regions included Australia, Canada, and Europe.

[c]Eastern Cooperative Oncology Group performance status ranges from 0 to 5, with 0 indicating no symptoms and higher scores indicating greater disability.

[d]Intracranial disease at baseline was confirmed by blinded independent central review.

[e]Includes patients in the safety population who did not have measurable or non-measurable lesions or were not yet evaluated by blinded independent central review.

Table S15. Adverse Events by TKI-Naïve and TKI-Pretreated Cohort.

	TKI-naïve cohort (n = 58)				TKI-pretreated cohort (n = 86)			
**Event**	**Treatment-emergent**		**Treatment-related**		**Treatment-emergent**		**Treatment-related**	
	**Any grade**	**Grade ≥3**	**Any grade**	**Grade ≥3**	**Any grade**	**Grade ≥3**	**Any grade**	**Grade ≥3**
	Number of patients (%)							
**Any event**	58 (100)	36 (62)	57 (98)	22 (38)	85 (99)	47 (55)	82 (95)	27 (31)
**Event occurring in ≥15% of patients**								
Dizziness	44 (76)	5 (9)	41 (71)	5 (9)	46 (53)	2 (2)	42 (49)	2 (2)
Dysgeusia	39 (67)	0	38 (66)	0	42 (49)	0	40 (47)	0
Constipation	27 (47)	1 (2)	23 (40)	0	32 (37)	0	16 (19)	0
Anemia	27 (47)	6 (10)	18 (31)	1 (2)	32 (37)	10 (12)	25 (29)	8 (9)
Paresthesia	24 (41)	1 (2)	22 (38)	1 (2)	27 (31)	0	22 (26)	0
Dyspnea	16 (28)	1 (2)	7 (12)	0	28 (33)	5 (6)	9 (10)	1 (1)
Fatigue	15 (26)	1 (2)	10 (17)	1 (2)	27 (31)	2 (2)	18 (21)	2 (2)
Increased alanine	13 (22)	2 (3)	9 (16)	2 (3)	14 (16)	0	11 (13)	0

aminotransferase level								
Ataxia	21 (36)	0	20 (34)	0	13 (15)	0	13 (15)	0
Muscular weakness	13 (22)	1 (2)	11 (19)	1 (2)	15 (17)	2 (2)	12 (14)	1 (1)
Increased aspartate aminotransferase level	11 (19)	4 (7)	9 (16)	3 (5)	16 (19)	2 (2)	12 (14)	0
Nausea	13 (22)	0	10 (17)	0	18 (21)	2 (2)	8 (9)	1 (1)
Headache	12 (21)	0	4 (7)	0	16 (19)	1 (1)	10 (12)	0
Cough	14 (24)	1 (2)	3 (5)	0	15 (17)	0	4 (5)	0
Increased blood creatine phosphokinase level	13 (22)	1 (2)	12 (21)	1 (2)	16 (19)	3 (3)	13 (15)	3 (3)
Arthralgia	10 (17)	0	0	0	8 (9)	0	3 (3)	0
Diarrhea	13 (22)	2 (3)	6 (10)	2 (3)	20 (23)	2 (2)	13 (15)	0

**Event that led to treatment discontinuation**	9 (16)	NA	3 (5)	NA	5 (6)	NA	2 (2)	NA
**Event that led to dose reduction**	35 (60)	NA	35 (60)	NA	30 (35)	NA	28 (33)	NA
**Event that led to dose interruption**	40 (69)	NA	34 (59)	NA	36 (42)	NA	27 (31)	NA
**Any serious event**	27 (47)	NA	8 (14)	NA	29 (34)	NA	10 (12)	NA
**Death**	3 (5)	NA	0	NA	5 (6)	NA	1 (1)	NA

REFERENCES

1. Aaronson NK, Ahmedzai S, Bergman B, et al. The European Organization for Research and Treatment of Cancer QLQ C30: a quality-of-life instrument for use in international clinical trials in oncology. J Natl Cancer Inst. 1993;85(5):365-76.
2. Osoba D, Rodrigues G, Myles J, Zee B, Pater J. Interpreting the significance of changes in health-related quality-of-life scores. J Clin Oncol. 1998 Jan;16(1):139-44.

A Phase 1/2, Open-Label, Multi-Center, First-in-Human Study of the Safety, Tolerability, Pharmacokinetics, and Anti-Tumor Activity of TPX-0005 in Patients with Advanced Solid Tumors Harboring *ALK*, *ROS1*, or *NTRK1-3* Rearrangements (TRIDENT-1)

**Protocol Number:**	TPX-0005-01
**Version (Date):**	1 (29 September 2016)
**Compound:**	TPX-0005
**Compound Name**	Not Applicable (N/A)
**United States (US) Investigational New Drug (IND) Number:**	130465
**European Clinical Trial Database (EudraCT) Number:**	2016-003616-13
**TP Therapeutics:**	TP Therapeutics, Inc.<br>10628 Science Center Dr #225,<br>San Diego, CA 92121

This document contains confidential information belonging to TP Therapeutics. Except as otherwise agreed to in writing, by accepting or reviewing this document, you agree to hold this information in confidence and not copy or disclose it to others (except where required by applicable law) or use it for unauthorized purposes. In the event of any actual or suspected breach of this obligation, TP Therapeutics must be promptly notified.

Approved v1.0

TP THERAPEUTICS APPROVALS

Protocol Number: TPX-0005-01

Version (Date): 1 (29 September 2016)

Protocol Title: A Phase 1/2, Open-Label, Multi-Center, First-in-Human Study of the Safety, Tolerability, Pharmacokinetics, and Anti-Tumor Activity of TPX-0005 in Patients with Advanced Solid Tumors Harboring *ALK*, *ROS1*, or *NTRK1-3* Rearrangements (TRIDENT-1)

[redacted]____________ Date: September 29th, 2016

____________ Date: September 29th, 2016

TP Therapeutics, Inc.

PROTOCOL AGREEMENT

I confirm that I have read this protocol. I will comply with the protocol and the principles of Good Clinical Practice (GCP), applicable local, state, and federal laws, as well as other applicable country laws and the appropriate International Council for Harmonisation guidance documents.

Protocol Number: TPX-0005-01

Version (Date): 1 (29 September 2016)

Protocol Title: A Phase 1/2, Open-Label, Multi-Center, First-in-Human Study of the Safety, Tolerability, Pharmacokinetics, and Anti-Tumor Activity of TPX-0005 in Patients with Advanced Solid Tumors Harboring *ALK*, *ROS1*, or *NTRK1-3* Rearrangements (TRIDENT-1)

____ Date:________________

Investigator Signature

____

Name and Title

____

Site #

____

Site Name

Approved v1.0

Table of Contents

TP Therapeutics Approvals 2
Protocol Agreement 3
List of Tables 13
List of Figures 14
List of Appendices 14
List of Abbreviations and Definition of Terms 15
1 INTRODUCTION 19
1.1 Oncogenic RTK Fusions as Driver Mutations in Solid Malignancies 19
1.1.1 ALK-positive (ALK+) Solid Malignancies 19
1.1.1.1 ALK+ NSCLC 19
1.1.1.2 ALK+ Non-NSCLC Solid Malignancies 20
1.1.2 *ROS1*-positive (*ROS1*+) Solid Malignancies 21
1.1.2.1 ROS1+ NSCLC 21
1.1.2.2 ROS1+ Non-NSCLC Solid Malignancies 21
1.1.3 *NTRK*-positive (*NTRK*+) Solid Malignancies 22
1.2 Disease Progression on TKIs in Oncogenic RTK Driven Solid Malignancies 24
1.2.1 Development of Acquired Resistant Mutations Including Solvent Front Mutations 24
1.2.2 Activation of Bypass Signaling Pathways (SRC, FAK, JAK/STAT) 26
1.2.3 Disease Progression in the Central Nervous System (CNS) 27
2 TPX-0005 28
2.1 *In vitro* and *In vivo* Inhibitory Activity of TPX-0005 28
2.1.1 Activity against Wild-type (WT) and Mutated ALK 28
2.1.2 Activity against WT and Mutated ROS1 29
2.1.3 Activity against Wild-type and Mutated TRK 29
2.1.4 Activity against Solvent Front Mutations of ALK, ROS1 and TRKA/B/C 30
2.1.5 Activity against SRC, FAK and JAK2 for Modulation of Bypass Signaling, EMT, Cancer Stemness and Metastasis 31
2.2 TPX-0005 Pre-Clinical Safety Data 32
[redacted] 33
34
34
2.3 Benefit to Risk Assessment 34
2.4 Safety Consideration in the Clinical Trial 35

[redacted] 35
37
3 STUDY DESIGN ... 38
3.1 Study Overview ... 38
3.2 Phase 1a Dose Escalation ... 38
3.2.1 Starting Level for Cycle 1 ... 38
3.2.2 Dose Escalation Levels ... 39
3.2.3 Criteria for Dose Escalation and Determination of MTD ... 39
3.2.3.1 Maximum Tolerated Dose (MTD) Definition ... 39
3.2.3.2 Dose-escalation Process ... 40
3.2.3.3 Dose Cohort Modification ... 40
3.2.3.4 DLT Definition ... 41
3.2.3.5 Follow-up Dose-Limiting Toxicities ... 42
3.2.3.5.1 Hematologic Toxicities ... 42
3.2.3.5.2 Renal Toxicity ... 43
3.2.3.5.3 Hepatic Toxicity ... 43
3.2.3.5.4 Non-laboratory Toxicity ... 43
3.2.3.6 Dose Modification and Dose Delay ... 43
[redacted] 44
3.2.3.8 Intra-patient Dose Escalation ... 44
3.3 Phase 1b Food Effect Sub-study ... 44
3.4 Phase 2 Dose Expansion ... 45
3.4.1 *ROS1*+ Solid Malignancy Expansion Cohorts ... 46
3.4.1.1 ROS1+ NSCLC Expansion Cohorts ... 46
3.4.1.2 ROS1+ Non-NSCLC Expansion Cohort ... 46
3.4.2 *ALK*+ Solid Malignancy Expansion Cohorts ... 46
3.4.2.1 ALK+ NSCLC Expansion Cohorts ... 46
3.4.2.2 ALK+ Non-NSCLC Expansion Cohort ... 47
3.4.3 *NTRK*+ Solid Malignancy Expansion Cohorts ... 47
4 STUDY OBJECTIVES ... 49
4.1 Phase 1 Objectives ... 49
4.1.1 Phase 1 Primary Objectives ... 49
4.1.2 Phase 1 Secondary Objectives ... 49
4.2 Phase 2 Objectives ... 49
4.2.1 Phase 2 Primary Objectives ... 49

4.2.2 Phase 2 Secondary Objectives 49
4.2.3 Phase 2 Exploratory Objectives 50
5 PATIENT SELECTION 51
5.1 Inclusion Criteria 51
5.2 Exclusion Criteria 55
6 STUDY TREATMENTS 57
6.1 TPX-0005 Drug Supply 57
6.1.1 Formulation and Packaging 57
6.1.2 Preparation and Dispensing 57
6.1.3 Administration 57
6.1.4 Storage and Accountability 58
6.1.4.1 Temperature Excursions 58
6.2 Dose Modifications 59
6.2.1 Recommendation for Dose Modifications 59
6.2.2 Dose Interruptions 59
6.2.3 Dose Reductions 60
6.2.4 Dose Modifications 61
6.3 Food Requirements 62
6.3.1 Non-Food Effect Cohort (Phase 1a and Phase 2) 62
6.3.2 Food Effect Cohort (Phase 1b) 62
6.4 Concomitant Medications 63
6.4.1 Cytochrome P450 Substrates 63
6.4.2 Other Anti-tumor or Investigational Drugs 64
6.4.3 Seizure Prophylaxis 64
6.4.4 Hematopoietic Growth Factors 65
6.4.5 Anti-diarrheal and Anti-emetic Therapy 65
6.4.6 Anti-inflammatory Therapy 65
6.4.7 Cutaneous Toxicity Therapy 65
6.4.8 Testosterone Replacement 66
6.5 Surgery 66
6.6 Palliative Radiation 66
6.7 Supportive Care 66
6.8 Life Style Guidelines 66
6.8.1 Contraception 66
6.8.2 Sunlight Exposure 67

6.8.3 Additional Lifestyle Guidance 67
7 STUDY PROCEDURES 68
7.1 Clinical Assessments 68
7.1.1 Demographics 68
7.1.2 Medical History 68
7.1.3 Physical Examination 68
7.1.4 Vital Signs 68
7.1.5 Performance Status 69
7.1.6 Adverse Events 69
7.1.7 Concomitant Medications and Treatments 69
7.2 Tumor Assessments 69
7.3 Tumor Procurement 70
7.3.1 Archival Tumor Tissue 70
7.3.2 *De Novo* Tumor Biopsy 70
7.3.3 Liquid Biopsies 71
7.4 Blinded Independent Central Review (BICR) 71
7.5 Clinical Laboratory Assessments 71
7.6 Electrocardiogram (ECG) 71
7.7 Echocardiogram 72
7.8 Pharmacokinetic (PK) Assessments 72
7.8.1 PK Collection Times Points 73
7.8.1.1 Blood for PK Analysis of TPX-0005 (Phase 1a Dose Escalation) 73
7.8.1.2 Blood for PK Analysis of TPX-0005 (Phase 1b Food Effect) 75
7.8.1.3 Blood for PK Analysis of TPX-0005 (Phase 2) 76
7.8.1.4 CSF for Analysis of TPX-0005 Concentration 77
7.9 Pharmacodynamic (PD) Assessment 78
7.9.1 *De Novo* Tumor Biopsy 78
7.10 Patient-Reported Outcomes (PRO) 78
8 STUDY ASSESSMENTS BY VISIT 79
8.1 Phase 1a Dose Escalation 79
8.1.1 Screening Phase 1a Dose Escalation (within 28 days of the first dose of TPX-0005) 79
8.1.2 Lead-in PK Cycle 0 Day -3 (Phase 1a Dose Escalation) 80
8.1.3 Cycle 1 Day 1 (Phase 1a Dose Escalation) 81

Approved v1.0

8.1.4 Cycle 1 Day 8 (Phase 1a Dose Escalation) 81
8.1.5 Cycle 1 Day 15 (Phase 1a Dose Escalation) 82
8.1.6 Cycle 2 Day 1 (Phase 1a Dose Escalation) 82
8.1.7 Cycle 2 Day 15 (Phase 1a Dose Escalation) 83
8.1.8 Cycle 3 Day 1 (Phase 1a Dose Escalation) 83
8.1.9 Cycle 4 Day 1 (Phase 1a Dose Escalation) 84
8.1.10 Every 4 Weeks from Cycle 5 Day 1 (Phase 1a Dose Escalation) 84
8.1.11 End of Treatment (Phase 1a Dose Escalation) (within 7 days post last dose of TPX-0005 and after decision to end treatment) 85
8.1.12 Safety Follow-up (Phase 1a Dose Escalation) (approximately 28 days post the last dose of TPX-0005) 85
8.1.13 Survival Follow-up (Phase 1a Dose Escalation) 85
8.2 Phase 1b Food Effect 86
8.2.1 Screening Phase 1b Food Effect (within 28 days of the first dose of TPX-0005) 86
8.2.2 Lead-in PK Cycle 0 Day -3 (Phase 1b Food Effect) 87
8.2.3 Cycle 1 Day 1 (Phase 1b Food Effect) 88
8.2.4 Cycle 1 Day 8 (Phase 1b Food Effect) 89
8.2.5 Cycle 1 Day 15 (Phase 1b Food Effect) 90
8.2.6 Cycle 2 Day 1 (Phase 1b Food Effect) 90
8.2.7 Cycle 2 Day 15 (Phase 1b Food Effect) 91
8.2.8 Cycle 3 Day 1 (Phase 1b Food Effect) 91
8.2.9 Cycle 4 Day 1 (Phase 1b Food Effect) 92
8.2.10 Every 4 Weeks from Cycle 5 Day 1 (Phase 1b Food Effect) 92
8.2.11 End of Treatment (Phase 1b Food Effect) (within 7 days post last dose of TPX-0005 and after decision to end treatment) 93
8.2.12 Safety Follow-up (Phase 1b Food Effect) (approximately 28 days post the last dose of TPX-0005) 93
8.2.13 Survival follow-up (Phase 1b Food Effect) 93
8.3 Phase 2 Dose Expansion 94
8.3.1 Screening Phase 2 Dose Expansion 94
8.3.2 Cycle 1 Day 1 (Phase 2 Dose Expansion) 95
8.3.3 Cycle 1 Day 8 (Phase 2 Dose Expansion) 96
8.3.4 Cycle 1 Day 15 (Phase 2 Dose Expansion) 96
8.3.5 Cycle 1 Day 21 (Phase 2 Dose Expansion) 97

Approved v1.0

8.3.6 Cycle 2 Day 1 (Phase 2 Dose Expansion) 97
8.3.7 Cycle 2 Day 15 (Phase 2 Dose Expansion) 98
8.3.8 Cycle 3 Day 1 (Phase 2 Dose Expansion) 98
8.3.9 Cycle 4 Day 1 and Each Subsequent Treatment Cycle (Phase 2 Dose Expansion) 99
8.3.10 End of Treatment (Phase 2 Dose Expansion) (within 7 days post last dose of TPX-0005 and after decision to end treatment) 99
8.3.11 Safety Follow-up (Phase 2 Dose Expansion) (approximately 28 days post the last dose of TPX-0005) 100
8.3.12 Survival Follow-up (Phase 2 Dose Expansion) 100
8.4 Study Calendars 101
9 SAFETY ASSESSMENTS 113
9.1 Adverse Events (AEs) 113
9.1.1 Serious Adverse Events (SAE) (Immediately Reportable to TP Therapeutics) 113
9.2 Methods and Timing for Capturing and Assessing Safety Parameters 114
9.2.1 Adverse Event Reporting Period 114
9.2.2 Eliciting Adverse Event Information 115
9.2.3 Assessment of Severity of Adverse Events 115
9.2.4 Assessment of Causality of Adverse Events 115
9.2.5 Procedures for Recording Adverse Events 116
9.2.5.1 Diagnosis versus Signs and Symptoms 116
9.2.5.2 Adverse Events That Are Secondary to Other Events 117
9.2.5.3 Persistent or Recurrent Adverse Events 117
9.2.5.4 Abnormal Laboratory Values 117
9.2.5.5 Abnormal Vital Sign Values 118
9.2.5.6 Abnormal Liver Function Tests 119
9.2.5.7 Deaths 120
9.2.5.8 Preexisting Medical Conditions 120
9.2.5.9 Lack of Efficacy or Worsening of the Malignancy under Study. 120
9.2.5.10 Hospitalization or Prolonged Hospitalization 121
9.2.5.11 Exposure During Pregnancy 121
9.2.5.12 Occupational Exposure 122
9.2.5.13 Adverse Events Associated with an Overdose or Incorrect Administration of Study Drug 123

Approved v1.0

9.3 Immediate Reporting Requirements from Investigator to TP Therapeutics 123
9.3.1 SAE Reporting 123
9.3.2 Reporting Requirements for Pregnancies 124
9.3.2.1 Pregnancies in Female Patients 124
9.3.2.2 Congenital Anomalies/Birth Defects and Abortions 124
9.4 Follow-Up of Patients after Adverse Events 124
9.4.1 Investigator Follow-Up 124
9.4.2 TP Therapeutics Follow-Up 124
9.5 Post-Study Adverse Events 125
9.6 Expedited Reporting to Health Authorities, Investigators, Institutional Review Boards, and Ethics Committees 125
10 PATIENT END OF TREATMENT 126
11 PROTOCOL VIOLATIONS 127
12 DATA MONITORING COMMITTEE 128
13 STATISTICAL METHODS AND CONSIDERATIONS 129
13.1 Analysis Populations (Sets) 129
13.1.1 Full Analysis Set 129
13.1.2 Safety Analysis Set 129
13.1.3 Per Protocol Analysis Set (Phase 1) 129
13.1.4 Per Protocol Analysis Set (Phase 2) 129
13.1.5 PK Parameter Analysis Set 130
13.2 Efficacy Analyses 130
13.2.1 Analysis of Primary Endpoint 130
13.2.1.1 Analysis of Primary Endpoint (Phase 1) 130
13.2.1.2 Analysis of Primary Endpoint (Phase 2) 130
13.2.2 Analysis of Secondary Endpoints 130
13.2.2.1 Analysis of Secondary Endpoints (Phase 1) 130
13.2.2.2 Analysis of Secondary Endpoints (Phase 2) 131
13.2.2.2.1 Duration of Response (DOR) 131
13.2.2.2.2 Time to Response (TTR) 131
13.2.2.2.3 Clinical Benefit Rate (CBR) 131
13.2.2.2.4 Intracranial Objective Response Rate (IC-ORR) 132
13.2.2.2.5 CNS Progression-Free Survival (CNS-PFS) 132
13.2.2.2.6 Progression-Free Survival 132
13.2.2.2.7 Overall Survival (OS) 132
13.2.3 Analysis of Planned Pooled Analysis 133

Approved v1.0

13.2.3.1 Analysis of Efficacy by Specific Gene Rearrangement (ALK, ROS1, NTRK1, NTRK2, NTRK3) 133
13.2.3.2 Analysis of Efficacy by Measurable CNS Lesions 133
13.2.4 Analysis of Planned Subset Analysis 133
13.2.4.1 Analysis of Efficacy and Safety by Specific NTRK Rearrangement (NTRK1, NTRK2, NTRK3)............................. 133
13.2.4.2 Analysis of Efficacy and Safety by Specific Fusion Partner Within Each Specific Gene Rearrangement (ALK, ROS1, NTRK1, NTRK2, NTRK3).. 133
13.3 Statistical Analysis Plan (SAP) and Sample Size Justification 133
13.3.1 Phase 1 Dose Escalation and Food Effect Cohorts 133
13.3.1.1 Phase 1a Dose Escalation.. 133
13.3.1.2 Phase 1b Food Effect Study. 134
13.3.2 *ROS1*-rearranged NSCLC Expansion Cohorts...134
13.3.2.1 ROS1-rearranged NSCLC Expansion Cohort (ROS1i-naïve [EXP-1]) 134
13.3.2.2 ROS1-rearranged NSCLC Expansion Cohort (crizotinib-refractory only [EXP-2]).. 135
13.3.2.3 ROS1-rearranged NSCLC Expansion Cohort (ROS1i-refractory, > 1 prior ROS1i, or 1 non-crizotinib-refractory [EXP-3])... 135
13.3.3 *ALK*-rearranged NSCLC Expansion Cohorts...135
13.3.3.1 ALK-rearranged NSCLC Expansion Cohort (Treatment naïve [EXP-4]) 136
[redacted] 136
13.3.3.3 ALK-rearranged NSCLC Expansion Cohort (ALKi-refractory: 4 or more ALKi [EXP-8]).. 137
13.3.4 *ALK*-rearranged Non-NSCLC, *ROS1*-rearranged Non-NSCLC, and *NTRK*-rearranged Solid Malignancy Expansion Cohorts......................137
13.3.4.1 ALK-rearranged Non-NSCLC, ROS1-rearranged Non-NSCLC, and NTRK-rearranged Solid Malignancy Expansion Cohort (TKI-naïve [EXP-9])................................... 138
13.3.4.2 ALK-rearranged Non-NSCLC, ROS1-rearranged Non-NSCLC, and NTRK-rearranged Solid Malignancy Expansion Cohort (TKI-refractory [EXP-10])........................... 138
13.4 Safety Analyses.. 138
13.4.1 Adverse Events 138
13.4.2 Clinical Laboratory Results..139

Approved v1.0

13.4.3 Vital Signs 139
13.4.4 Concomitant Medications/Treatment 139
13.4.5 ECG 139
13.4.6 Left Ventricular Ejection Fraction (LVEF) 140
13.5 Population Pharmacokinetic (popPK)....140
13.5.1 Japan Sub-Study 141
13.6 Patient-Reported Outcomes (PROs) 141
13.6.1 Administration of PROs 142
13.6.2 EORTC-QLQ-C30 143
13.6.2.1 Technical Summary 144
13.6.2.2 Linear Transformation 144
13.6.2.3 Missing Items for QLQ-C30 144
13.6.3 EORTC-QLQ-LC13 144
13.6.4 QOL Compliance Rates....145
13.6.5 PRO Endpoints 146
13.6.6 PRO Analyses....146
13.6.6.1 Change from Baseline Scores 146
13.6.6.2 Proportion of Patients Improved, Remained Stable, or Worsened 146
14 DATA COLLECTION, RETENTION AND MONITORING 147
14.1 Data Collection Instruments 147
14.2 Data Management Procedures 147
14.3 Data Quality Control and Reporting 147
14.4 Data Archival 147
14.5 Availability and Retention of Investigational Records 148
14.6 Monitoring 148
14.7 Patient Confidentiality 148
15 ADMINISTRATIVE, ETHICAL, REGULATORY CONSIDERATIONS 149
15.1 Protocol Amendments....149
15.2 Institutional Review Boards/Ethic Committees....149
15.3 Reporting of Safety Issues and Serious Breaches of the Protocol or ICH GCP 150
15.4 TP Therapeutics Discontinuation Criteria 150
15.5 Post-Trial Access 151
15.6 Publications....151
16 REFERENCES 152

List of Tables

Table 1 List of ALK Fusions in Non-NSCLC Solid Malignancies 20
Table 2 List of ROS1 Fusions in Non-NSCLC Solid Malignancies 22
Table 3 List of *NTRK* Fusions in Solid Malignancies 23
Table 4 ALK/ROS1/TRK Inhibitors and Clinical Resistant Mutations. 25
Table 5 The Activities of TPX-0005 against WT and Solvent Front Mutated ALK, ROS1 and TRKs 30
Table 6 Provisional Dose Levels 39
Table 7 Criteria for Defining Dose-limiting Toxicities 41
Table 8 Adverse Events Dose Modifications Table 61
Table 9 List of EIAEDs and non-EIAEDs 65
Table 10 Noncompartmental Pharmacokinetic Parameters 72
Table 11 Time Points of Blood Collection for TPX-0005 PK Assessment during Phase 1a Dose Escalation Phase 74
Table 12 Time Points of Blood Collection for TPX-0005 PK Assessment during Phase 1b Food Effect Study (FED Followed by FASTED) 75
Table 13 Time Points of Blood Collection for TPX-0005 PK Assessment during Phase 1b Food Effect study (FASTED Followed by FED) 76
Table 14 Time Points of Blood Collection of TPX-0005 PK Assessment during Phase 2 Expansion Cohort 77
Table 15 Study Calendar for Phase 1a Dose Escalation Portion of TPX-0005-01 101
Table 16 Study Calendar for Phase 1b Food Effect Portion of TPX-0005-01 105
Table 17 Study Calendar for Phase 2 Dose Expansion Portion of TPX-0005-01 109
Table 18 Grading of Severity of Adverse Events 115
Table 19 Causation Attribution Table 116
Table 20 Estimated ORRs and Related 95% Confidence Intervals in 50 patients 134
Table 21 Estimated ORR and Related 95% Confidence Interval (Clopper-Pearson) in 40 Patients 136
Table 22 Estimated ORR and Related 95% Confidence Interval (Clopper-Pearson) in 120 Patients 136
Table 23 Patient-Reported Outcome QOL Instruments 142

Approved v1.0

List of Figures

Figure 1 The anti-tumor efficacy of TPX-0005 in WT and solvent front mutated ALK, ROS1 and TRKA tumor models. 31
Figure 2 Schema of TPX-0005-01 Trial........ 48

List of Appendices

Appendix 1 Phase 1 Required Laboratory Assessments 160
Appendix 2 Phase 2 Required Laboratory Assessments 161
Appendix 3 Japanese Patient-Only Lead-In Cohort (LIC) Schedule of Assessments........ 162
Appendix 4 Safety Review Process for Japanese Only Patient LIC 166
Appendix 5 Patient-Reported Outcomes: EORTC-QLQ-C30 167
Appendix 6 Patient-Reported Outcomes: EORTC-QLQ-LC13 169
Appendix 7 Table of Medications That ARE Strong CYP3A4/5 Inhibitors or Inducers of CYP3A4/5 or Cause QTc Prolongation........ 170

List of Abbreviations and Definition of Terms

The following abbreviations and specialist terms are used in this study protocol.

**AE**	Adverse event
**AKT**	Protein kinase B
**ALCL**	Anaplastic large cell lymphoma
**ALK**	Anaplastic lymphoma kinase
**ALKi**	ALK inhibitor
**ALP**	Alkaline phosphatase
**ALT (SGPT)**	Alanine aminotransferase
**ANC**	Absolute neutrophil count
**ASCO**	American Society of Clinical Oncology
**AST (SGOT)**	Aspartate aminotransferase
**ATP**	Adenosine triphosphate
**AUC**	Area under curve
**$AUC_{(0-t)}$**	Area under plasma concentration-time curve from zero to time t [amount × time/volume]
**AUC_{inf}**	The AUC from time zero to infinity
**AUC_{last}**	The area under the curve (AUC) from time zero to the last quantifiable concentration point (t_{last})
**BBB**	Blood-brain barrier
[redacted]	
**BICR**	Blinded Independent Central Review
**BID**	Twice a day
**BSA**	Body surface area
**CBR**	Clinical Benefit Rate
**ccfDNA**	Circulating cell-free DNA
**CFR**	Code of Federal Regulations
**CI**	Confidence interval
**C_{ave}**	The average observed plasma, blood, serum, or other body fluid drug concentration
**C_{max}**	The maximum observed plasma, blood, serum, or other body fluid drug concentration
**C_{min}**	Observed concentration at the end of a dosing interval
**CNS**	Central nervous system
**CNS-PFS**	CNS Progression-Free Survival
**CPK**	Creatine phosphokinase
**CR**	Complete response
**CRF**	Clinical Research Form
**CSF**	Cerebral spinal fluid
**CT**	Computerized tomography
**C_{trough}**	Concentration at the end of dosage interval
**CYP450**	Cytochrome P450
**DLT**	Dose-limiting toxicity
**DMC**	Data Monitoring Committee
**DOR**	Duration of response
**ECG**	Electrocardiogram
**ECOG**	Eastern Cooperative Oncology Group
**EDC**	Electronic data capture
**EGFR**	Epidermal growth factor receptor

**EF score**	Emotional functioning score
**EIAED**	Enzyme-inducing anti-epileptic drug
**EIU**	Exposure in utero
**EMT**	Epithelial-mesenchymal transition
**EORTC**	European Organization for Research and Treatment of Cancer
**EOS**	End of study
**EOT**	End-of-treatment
**ERK**	Extracellular signal–regulated kinase
**EXP**	Expansion
**FAK**	focal adhesion kinase
**FAS**	Full Analysis Set
**FDA**	Food and Drug Administration
**FFPE**	Formalin-fixed paraffin-embedded
**FIH**	First-in-human
**FISH**	Fluorescence *in situ* hybridization
**FSH**	Follicle-stimulating hormone
**GCP**	Good Clinical Practice
**HEK**	Human embryonic kidney
**hERG**	Human ether-a-go-go-related gene
**Hgb**	Hemoglobin
[redacted]	
**HPF**	High power field
**HRQoL**	Health-related quality of life
**IC_{50}**	Half maximal inhibitory concentration
**ICF**	Informed consent form
**ICH**	International Council for Harmonisation
**ICMJE**	International Committee of Medical Journal Editors
**IC-ORR**	Intracranial objective response rate
**IFU**	Instructions for use
**IHC**	Immunohistochemistry
**IMT**	Inflammatory myofibroblastic tumor
**INR**	International normalized ratio
**IP**	investigational product
**IRB**	Institutional Review Board
**IV**	Intravaneous
**JAK**	Janus kinase
**LC-MS/MS**	Liquid chromatography-tandem mass spectroscopy
**LFT**	Liver function test
**LIC**	Lead-In cohort
**LVEF**	Left ventricular ejection fraction
**MASC**	Mammary-associated secretory adenocarcinoma
**MEC**	Molar extinction coefficient
**MedDRA**	Medical Dictionary for Regulatory Affairs
**mEq/L**	Milliequivalent per liter
**mIU**	Milli international unit
**MRI**	Magnetic resonance imaging

**MTD**	Maximum tolerated dose
**MW**	Molecular weight
**NA**	Not applicable
**NCI CTCAE**	National Cancer Institute Common Terminology Criteria for Adverse Events
**ND**	Not determined
**NGS**	Next-generation sequencing
[redacted]	
**NSCLC**	Non-small-cell lung cancer
**NTRK**	Neutrophin receptor kinase
**NTRK-1**	Neurotrophin receptor kinase 1
**NTRK-2**	Neurotrophin receptor kinase 2
**NTRK-3**	Neurotrophin receptor kinase 3
**ORR**	Objective response rate
**OS**	Overall survival
[redacted]	
**PD**	Pharmacodynamic
**PET**	Positron emission tomography
**PFS**	Progression-free survival
**P-gp**	P-glycoprotein
**PK**	Pharmacokinetics
**PO**	per os
**popPK**	Population pharmacokinetic
**PR**	Partial response
**PRBC**	Packed red blood cells
**PRO**	Patient-reported outcomes
**PS**	Performance Status
**PT**	Prothrombin time
**PTT**	Partial thromboplastin time
**QD**	Once a day
**QLQ**	Quality of life questionnaire
**QOL**	Quality of life
**QT**	ECG interval measured from the onset of the QRS complex to the end of the T wave
**QTc**	QT interval corrected for heart rate
**QTcB**	QT interval corrected for heart rate using Bazett's formula
**QTcF**	QT interval corrected for heart rate using Fridericia's formula
**R_{acc}**	Accumulation ratio calculated using AUC_{tau} values obtained from a dosing interval at steady-state divided by AUC_{tau} at Day 1 or PK lead-in phase
**RECIST**	Response Evaluation Criteria in Solid Tumors
**ROS1**	Receptor tyrosine kinase encoded by the *ROS1* gene
**ROS1i**	ROS1 inhibitor
**RP2D**	Recommended Phase 2 dose
**RR**	R wave-to-R wave (RR) interval
**RS**	Raw score
**RTK**	Receptor tyrosine kinase
**SAE**	Serious adverse event
**SAP**	Statistical analysis plan

Approved v1.0

**SCID**	Severe combined immunodeficiency
**SD**	Stable disease
**SGOT**	Serum glutamic oxaloacetic transaminase
**SGPT**	Serum glutamic pyruvic transaminase
**SRC**	Tyrosine kinase first identified in avian sarcoma virus
**STAT**	Signal transducer and activator of transcription
**SOC**	System organ class
**SOP**	Standard operating procedure
[redacted]	
**$t_{1/2}$**	Half-life
**$t_{½,acc}$**	Effective half-life can be calculated from R_{acc}
**TEAE**	Treatment-emergent adverse event
**TKI**	Tyrosine kinase inhibitor
**T_{max}**	The time to reach maximum (peak) plasma, blood, serum, or other body fluid drug concentration
**TNBC**	Triple-negative breast cancer
**TRK**	Tropomyosin receptor kinase
**TRKA**	Tropomyosin receptor kinase A
**TRKB**	Tropomyosin receptor kinase B
**TRKC**	Tropomyosin receptor kinase C
**TRKi**	Tropomyosin receptor kinase inhibitor
**TT**	Total testosterone
**TTR**	Time to response
**UGT**	Uridine 5'-diphospho-glucuronosyltransferase
**ULN**	Upper limit of normal
**UVB**	Ultra Violet –B
**Vd**	volume of distribution
**VEGF**	Vascular endothelial growth factor
**V_z/F**	The apparent volume of distribution during terminal phase (associated with λ_z)
**WBRT**	Whole brain radiation treatment
**WT**	Wild-type
**λ_z**	Smallest (slowest) disposition (hybrid) rate constant (time-1) may also be used for terminal elimination rate constant

Approved v1.0

1 INTRODUCTION

1.1 Oncogenic RTK Fusions as Driver Mutations in Solid Malignancies

There are 58 human receptor tyrosine kinases (RTKs) and can be divided into 20 subfamilies (Blume-Jensen 2001). Rearrangement in many of the RTKs that retains the intact kinase domain are now know to be actionable oncogenic driver mutations in epithelial malignancies (Shaw 2013a). In particular, non-small-cell lung cancer (NSCLC) harboring rearrangement in the anaplastic lymphoma kinase (ALK) gene serves as a paradigm for management of actionable oncogenic drivers.

1.1.1 ALK-positive (ALK+) Solid Malignancies

1.1.1.1 ALK+ NSCLC

Lung cancer is the leading cause of cancer diagnosis with an estimated of 1.82 million new lung cancer cases diagnosed in 2012 (Torre 2015). Additionally, lung cancer is the leading cause of cancer-related death globally, with an estimated 1.59 million patients will die of lung cancer (Torre 2015). Improvements in biologic understanding have led to the identification of multiple therapeutic molecular targets in NSCLC. Large-scale comprehensive molecular profiling studies have identified multiple actionable driver mutations in NSCLC including oncogenic fusions involving the RTKs ALK and ROS1 (Kris 2014; Barlesi 2016). Recently, uncommon oncogenic fusions involving the neurotrophin receptor kinase 1 (*NTRK1*) (Vaishnavi 2013) and neurotrophin receptor kinase 2 (*NTRK2*) rearrangements have been reported in NSCLC (Stransky 2014).

ALK is one of 58 human RTKs (Blume-Jensen 2001). *ALK* rearrangement (*ALK*+) in NSCLC was first reported in 2007 (Soda 2007; Rikova 2007) and the clinical benefit of targeting oncogenic ALK fusions has led to the approval of several ALK inhibitors in the United States: crizotinib (Xalkori®) on August, 26, 2011 (Malik 2014), ceritinib (Zykadia®) on April 29, 2015 (Khozin 2015), and alectinib (Alecensa® on December 11, 2015). Two large randomized studies have compared crizotinib to chemotherapy (Shaw 2013b; Solomon 2014). Crizotinib has achieved superior objective response rates (ORRs) of 65% – 74% and superior progression-free survival (PFS) (7.3 months – 10.9 months) in *ALK*+ NSCLC patients. Crizotinib achieved full regular approval based on the statistically superior PFS compared to single-agent chemotherapy as second-line treatment of *ALK*+ NSCLC patients after progression on first-line platinum based chemotherapy (Kazandjian 2014).

Ceritinib, a more potent second-generation ALK inhibitor, has demonstrated an ORR of 43% with a median duration of response (DOR) of 7 months by blinded independent review committee in *ALK*+ NSCLC patients who progressed or intolerant of crizotinib (Shaw 2014b; Khozin 2015). Alectinib, another second-generation ALK inhibitor, demonstrated ORRs of 50% and 48% and DOR of 11.2 months and 13.0 months in a global and North American Phase 2 study respectively in *ALK*+ NSCLC patients who progressed or intolerant of crizotinib (Ou 2016; Shaw 2016a). The clinical activity of brigatinib, another potent second-generation ALK inhibitor

Approved v1.0

was investigated in a randomized Phase 2 study comparing 2 dosing strategies. Brigatinib given orally at 90 mg once daily for the first 7 days and then escalated to 180 mg once daily was the superior arm and achieved an ORR of 54% (95% confidence interval [CI]: 43% – 65%) and median PFS of 12.9 months (95%CI: 11.1 months – not reached) (Kim 2016b).

1.1.1.2 ALK+ Non-NSCLC Solid Malignancies

Besides the rearrangement in anaplastic large cell lymphoma (ALCL) where ALK derived its namesake, *ALK* rearrangement has been identified in many epithelial malignancies (Table 1).

Table 1 List of ALK Fusions in Non-NSCLC Solid Malignancies

Tumor	ALK fusions	Reference
Bladder cancer	TPM1-ALK	Stransky 2014
Breast cancer	EML4-ALK	Lin 2009
Colorectal adenocarcinoma	EML4-ALK	Lin 2009
	C2orf44-ALK	Lipson 2012
	CAD-ALK	Lee 2015
	SPTBN1-ALK	Ying 2015
Inflammatory Myofibroblastic Tumor (IMT)	CLTC-ALK<br>FN1-ALK<br>EMl4-ALK<br>PRKAR1A-ALK<br>RANB2-ALK<br>SEC31A-ALK<br>TFG-ALK<br>TPM3-ALK<br>TPM4-ALK	Lovly 2014
Pigmented spindle cell nevus/Spitz nevus	DCTN1-ALK<br>TPM3-ALK	Wiesner 2014
Rectal adenocarcinoma	SMEK2-ALK	Stransky 2014
Renal carcinoma	VCL-ALK	Debelenko 2011
Renal medullary carcinoma	VCL-ALK	Mariño-Enríquez 2011
Thyroid cancer	GTF2IRD1-ALK	Stransky 2014
Thyroid cancer (anaplastic)	STRN-ALK	Perot 2014
Thyroid cancer (medullary)	GFPT1-ALK	Ji 2015
	EML4-ALK	Demeure 2014

Approved v1.0

1.1.2 *ROS1*-positive (*ROS1*+) Solid Malignancies

1.1.2.1 ROS1+ NSCLC

ROS1 is another one of the 58 human RTKs (Blume-Jensen 2001) and shares extensive amino acid homology with ALK in the kinase domain (Ou 2012). *ROS1* rearrangement in NSCLC was discovered in 2007 (Rikova 2007) and shares similar clinicopathologic characteristics with *ALK*-positive NSCLC (Bergethon 2012). Given the extensive homology between ALK and ROS1, crizotinib is a ROS1 inhibitor and has demonstrated significant clinical activity with an ORR of 73% and a median PFS of 19.2 months as a single agent in 50 *ROS1*+ NSCLC patients (Shaw 2014a). Independent radiologic review of the study revealed a confirmed ORR of 66% (95%CI: 51% – 79%) and DOR of 18.3 months (95%CI: 12.7 months – not reached) (Kazandjian 2016). Based on this significant clinical activity of crizotinib in *ROS1*+ NSCLC patients, crizotinib was approved by the US Food and Drug Administration (FDA) for the treatment of advanced and/or metastatic *ROS1*+ NSCLC patients on March 11, 2016 (Kazandjian 2016). Additionally, a Phase 2 study of crizotinib in *ROS1*+ NSCLC patients in Asia confirmed the high ORR of 69.3% (95% CI: 60.5% - 77.2%) that observed in the US study and the on-going median PFS of 13.4 months (5%CI: 10.3 months – not reached) (Goto 2016). More importantly, the ORR to crizotinib was independent of the prior number of chemotherapy regimens. The ORR was 75% (95%CI: 53.3%) in patients with no prior chemotherapy, 66% (95%CI: 51.7% – 78.5%) in patients who received 1 prior chemotherapy regimen, 63.3% (95%CI: 43.9% – 80.1%) in patients who received 2 prior chemotherapy regimens, and 80.8% (95%CI: 56.3% – 94.3%) in patients who received 3 or more prior chemotherapy regimens. Similar to the patients with *ALK*+ NSCLC, acquired resistant *ROS1* mutations developed in *ROS1*+ NSCLC patients during treatment with crizotinib. Solvent front mutations in *ROS1* such as G2032R (Awad 2013; Song 2015) and D2033N (Drilon 2016b) seemed to be the dominant acquired resistant mutations to crizotinib.

1.1.2.2 ROS1+ Non-NSCLC Solid Malignancies

Similar to *ALK* rearrangements, *ROS1* rearrangements have been identified in many non-NSCLC solid malignancies. Importantly, patients with *ROS1*-rearranged inflammatory myofibroblastic tumor (IMT) has responded to crizotinib (Lovly 2014). Listed below are the published ROS1 fusions in non-NSCLC solid malignancies (Table 2).

Table 2 List of ROS1 Fusions in Non-NSCLC Solid Malignancies

Tumor	ROS1 fusions	Reference
Angiosarcoma	CEP85L-ROS1	Giacomini 2013
Anaplastic large cell lymphoma	NFkB2-ROS1<br>NCOR-ROS1	Crescenzo 2015
Cholangiocarcinoma	GOPC-ROS1 (FIG-ROS1)	Gu 2011
Colorectal adenocarcinoma	SLC34A2-ROS1	Aisner 2014
Gastric adenocarcinoma	SLC34A2-ROS1	Lee 2013
Glioblastoma Multiforme	GOPC-ROS1 (FIG-ROS1)	Charest 2003
Inflammatory Myofibroblastic Tumor (IMT)	TFG-ROS1<br>YWHAE-ROS1	Lovly 2014
Pigmented spindle cell nevus/Spitz nevus	CLIP1-ROS1<br>ERC1-ROS1<br>HLA-A-ROS1<br>KIAA1598-ROS1<br>MYO5A-ROS1<br>PWWP2A-ROS1<br>ZCCHC8-ROS1	Wiesner 2014

1.1.3 *NTRK*-positive (*NTRK*+) Solid Malignancies

Tropomyosin receptor kinase (TRK) encoded by neurotrophin receptor kinase (*NTRK*) gene belongs to the TRK sub-family among the 58 members of the human RTK (Blume-Jensen 2001). There are 3 members of TRK family: TRKA, TRKB, and TRKC encoded by *NTRK1*, *NTRK2*, and *NTRK3*, respectively. The transforming activity of the TRKA kinase was first discovered as the transforming kinase partner to tropomyosin gene in a colorectal cancer tumor sample (Martin-Zanca 1986). Subsequently TRKA was identified to be the receptor for nerve growth factor (Kaplan 1991; Klein 1991). TRKB (Squinto 1991) and TRKC (Lamballe 1991) were discovered later. All 3 members share significant amino acid homology especially among the kinase domains (Barbacid 1995). Oncogenic rearrangements of *NTRK1*, *NTRK2*, and *NTRK3* that lead to function TRK fusion proteins have subsequently been identified in many solid malignancies (Vaishnavi 2015) (Table 3).

Approved v1.0

Table 3 List of *NTRK* Fusions in Solid Malignancies

Tumor type	*NTRK* fusion type	Reference
Colorectal adenocarcinoma	TPM3-NTRK1	Martin-Zanca 1986; Ardini 2014
Colorectal adenocarcinoma	TPR-NTRK1	Créancier 2015
	LMNA-NTRK1	Russo 2016
NSCLC	CD74-NTRK1	Vaishnavi 2013
	MPRIP-NTRK1	Vaishnavi 2013
	SQSTM1-NTRK1	Farago 2015
	TRIM24-NTRK2	Stransky2014
Mammary Analogue of Secretory Carcinoma (MASC) of the salivary gland	ETV6-NTRK3	Skálová 2010
Squamous cell head and neck cancer	PAN3-NTRK2	Stransky 2014
Spitzoid melanoma	LMNA-NTRK1	Wiesner 2014
	TP53-NTRK1	Wiesner 2014
GIST	ETV6-NTRK3	Brenca 2016
Intrahepatic Cholangiocarcinoma	RABGAP1L-NTRK1	Ross 2014
Soft tissue sarcoma	LMNA-NTRK1	Doebele 2015
	TPM3-NTRK3	Stransky 2014
	NTRK3-HOMER2	Doebele 2015
Inflammatory Myofibroblastic Tumor (IMT)	ETV6-NTRK3	Yamamoto 2016
Soft tissue fibrosarcoma (pediatric)	LMNA-NTRK1	Doebele 2015
	SQSTM1-NTRK1	Doebele 2015
Soft tissue schwannoma (pediatric)	TPM3-NTRK1	Doebele 2015
Soft tissue hemangioma (pediatric)	ETV6-NTRK3	Doebele 2015
Soft tissue solitary fibrous tumor (pediatric)	TFG-NTRK3	Doebele 2015
Infantile (congenital) fibrosarcoma (pediatric < 1 year)	ETV6-NTRK3	Knezevich 1998
Glioblastoma Multiforme	NFASC-NTRK1	Frattini 2013
	BCAN-NTRK1	Kim 2014
	CHTOP-NTRK1	Zheng 2014
	ARHGEF2-NTRK1	Zheng 2014
Low grade glioma	AFAP1-NTRK2	Stransky 2014
Pediatric non-brainstem High Grade Glioma	TPM3-NTRK1	Wu 2014
	BTBD1-NTRK1	Wu 2014
	VCL-NTRK2	Wu 2014
	AGBL4-NTRK2	Wu 2014
Papillary Thyroid	TPM3-NTRK1	Greco 2010
	TPR-NTRK1	Greco 2010
	TFG-NTRK1	Greco 2010
	PPL-NTRK1	Zheng 2014
Secretary breast carcinoma	ETV6-NTRK3	Tognon 2002

Approved v1.0

The use of tyrosine kinase inhibitors (TKIs) targeting TRK family kinases have led to clinical benefit in patients with solid malignancies harboring oncogenic *NTRK* fusions: *LMNA-NTRK1* in colorectal cancer and soft tissue sarcoma (Sartore-Bianchi 2016; Doebele 2015), *SQSTM-NTRK1* in NSCLC (Farago 2015), *ETV6-NTRK3* in mammary associated secretory carcinoma (MASC) (Drilon 2016a). Therefore, identifying and treating these diverse patients with oncogenic *NTRK* fusion genes will likely be clinically beneficial.

1.2 Disease Progression on TKIs in Oncogenic RTK Driven Solid Malignancies

Despite the established benefit of crizotinib and second-generation ALK inhibitors, median PFS of patients on front-line crizotinib in treatment-naïve advanced *ALK*+ NSCLC was about 10.9 months (Solomon 2014; Lu 2016). The median PFS of second-generation ALK inhibitors crizotinib-refractory *ALK*+ NSCLC ranged from 7.4 months for ceritinib (Shaw 2013b), 8.9 months for alectinib (Ou 2016) to 12.9 months for brigatinib (Kim 2016b) as resistance to various ALK inhibitors invariably develops. Generally, the mode of resistance can be broadly divided into 3 categories and can occur in combination of these 3 modes of progression. First is the development of brain metastases especially among patients on crizotinib. Second is the development of acquired ALK resistant mutations. Third is the activation of multiple bypass pathways.

1.2.1 Development of Acquired Resistant Mutations Including Solvent Front Mutations

ALK, ROS1 and TRK kinases prefer type I kinase inhibitor for favored interactions at the hinge, glycine-rich P-loop, and the solvent front exposure area. As shown in Table 4, all of the inhibitors have a red colored heteroatom interacting with the hinge. Most of the *N*-lobe mutations of ALK, including 1151Tins, C1156Y, I1171T/N/S, F1174L/C, V1180L and L1196M destabilize the inactive conformation and shift ALK in a more active conformation for tighter binding with ATP. The mutations at ALK G1202, D1203, S1206 and E1210 residues, referred together as solvent front mutations, are caused by steric interactions with the blue colored motif, a bulky feature of all ALK inhibitors, and have been identified to be acquired resistance mutations to ALK inhibitors . It is not surprising that ALK G1202R mutation renders a common resistance to all ALK inhibitors because of an aromatic ring (blue colored) just located over the G1202 residue. Although it is much smaller (MW 406.42) than most of ALK inhibitors, the third generation of ALK inhibitor lorlatinib still has a pyrazole aromatic ring (blue colored) located on the top of G1202 residue, leading to a much reduced activity against ALK G1202R mutation (>20 fold reduction over wild type) (Gainor 2016). Similar to ALK G1202R, the same glycine mutations of ROS1 G2032R (Awad 2013), TRKA G595R (Russo 2016), and TRKC G623R (Drilon 2016a) render resistances to ROS1 inhibitor crizotinib and TRK inhibitor entrectinib, respectively in the clinic.

Approved v1.0

Table 4 ALK/ROS1/TRK Inhibitors and Clinical Resistant Mutations.

Kinase Inhibitor	Structure	Molecule Weight (MW)	Primary Targets	Clinical Resistant Mutations
Crizotinib	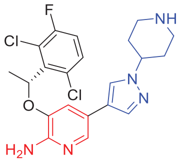	450.34	ALK, ROS1, MET	ALK[a]: 1151Tins, C1156Y, I1171T/N/S, L1196M, G1202R*, S1206Y/C, E1210K, G1269A<br><br>ROS1: D2032R*[b], D2033N*[c]
Ceritinib	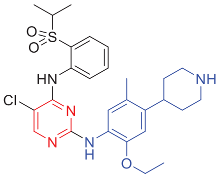	558.14	ALK, ROS1	ALK[a]: C1156Y, I1171T/N/S, F1174L/C, V1180L, L1196M, G1202R*, G1202del, D1203N<br><br>ROS1: no data reported
Alectinib	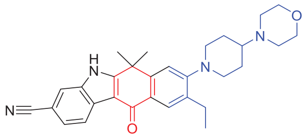	482.64	ALK	ALK[a]: I1171T/N/S, V1180L, L1196M, G1202R*
Brigatinib	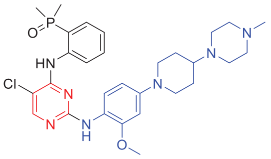	584.10	ALK, ROS1	ALK[a]: G1202R*, D1203N, S1206Y/C, E1210K<br><br>ROS1: no data reported
Lorlatinib	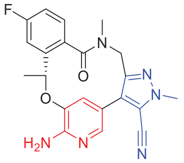	406.42	ALK, ROS1	ALK: L1198F[d]<br><br>ROS1: no data reported
Entrectinib	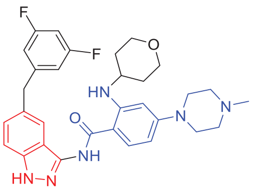	560.65	ALK, ROS1, pan-TRK	ALK: no data reported.<br><br>ROS1: no data reported.<br><br>TRKA: G595R*[e]<br>TRKC: G623R*[f]

*solvent front mutation; [a] (Gainor 2016); [b] (Awad 2013); [c] (Drilon 2016b); [d] (Shaw 2016b); [e] (Russo 2016); [f] (Drilon 2016a)

Overall, the current ALK, ROS1, and TRK inhibitors approved or in clinic trials are much bigger than adenosine triphosphate (ATP) non-phosphate motif, and are predicted to develop common and unique resistance mutations in the clinic. An inhibitor targeting the resistance-driven kinase active conformation and located completely inside ATP adenine binding pocket to avoid developing steric hindrance from resistance mutations is highly desired for systematically overcoming the acquired resistance mutation issues.

Approved v1.0

1.2.2 Activation of Bypass Signaling Pathways (SRC, FAK, JAK/STAT)

The tumor microenvironment is being increasingly recognized as a key factor in multiple stages of disease progression, particularly local resistance, immune-escaping, and distant metastasis. Excessive activation of the JAK family and their associated transcription factors STAT3 and STAT5 have been observed in many human malignancies in response to increased abundance of inflammatory cytokines in the tumor microenvironment prominently produced by infiltrating innate immune cells (Buchert 2016). JAK/STAT3 signaling promotes cancer hallmarks in the tumor and its microenvironment, including proliferation, survival, angiogenesis, and tumor metabolism while suppressing anti-tumor immunity (Buchert 2016). It was reported that JAK/STAT3 signaling pathway was aberrantly increased in EGFR inhibitor-resistant EGFR-mutant NSCLC cells, and JAK2 inhibition overcomes acquired resistance to EGFR inhibitors (Gao 2016). Focal amplification of JAK2 gene was also observed in post chemotherapy triple-negative breast cancers in a group of 9p24-amplified tumors, suggesting a role in tumorigenicity and chemoresistance (Balko 2016). Therefore, pharmacologic inhibition of the JAK2 signaling pathway can be an important new therapeutic strategy to enhance anti-tumor activity and overcome treatment resistance.

Also, SRC kinase has been identified to contribute broadly to cancer treatment resistance including radiotherapy, chemotherapy, and targeted therapy (Zhang 2012). SRC/FAK signaling pathway plays important roles in regulating anti-tumor immunity (Serrels 2015), cancer stem-like properties (Thakur 2015), and epithelial-mesenchymal transition (EMT) (Wilson 2014). SRC family kinases can promote mitogenic signaling from growth factor receptors in a number of ways, including initiation of signaling pathways required for DNA synthesis, control of receptor turnover, actin cytoskeleton rearrangements and motility, and survival (Bromann 2004). Recent findings have demonstrated that integrins participate in the regulation of cancer stem-cell biology and are required for cancer progression, metastasis, and drug resistance *via* SRC/FAK signaling (Seguin 2015). The integrin β1/SRC/AKT-driven bypass signaling is a key mediator of acquired resistance to EGFR-targeted anticancer drugs (Kanda 2013). The EGFR-SRC family kinase-STAT3 signaling pathway was upregulated in BRAF inhibitor-resistant cells and tumors with intrinsic or acquired resistance to vemurafenib (Girotti 2013). BRAF and EGFR or SFK inhibition blocked proliferation and invasion of these resistant tumors. It was found that the increased SRC activation conferred considerable trastuzumab resistance in breast cancer cells and correlated with trastuzumab resistance in patients (Zhang 2011). Similarly, SRC activation was observed in lapatinib-resistant breast cancer cell lines. The selective small-molecule SRC inhibitor saracatinib combined with lapatinib synergistically inhibited the proliferation, migration, and invasion of lapatinib-resistant cells (Formisano 2014). In addition, it was found that the selective SRC inhibitor saracatinib can restore sensitivity to ALK inhibitor in 6 of 9 patient-derived resistant ALK fusion positive NSCLC cell lines (Crystal 2014), suggesting a therapeutic role of SRC in modulating ALK inhibitor resistance. SRC proteins were overexpressed in alectinib-resistance H3122 cells and the combination of alectinib and saracatinib demonstrated significant treatment effect in *in vitro* and *in vivo* studies using tumor-bearing mouse model (Yoshida 2016). Overall, SRC activation plays an important role in the development of targeted therapy resistance, and targeting SRC signaling pathway may represent a valid strategy overcoming bypass signaling and EMT.

Approved v1.0

1.2.3 Disease Progression in the Central Nervous System (CNS)

There is a higher incidence of central nervous system (CNS) metastases (27%) in advanced treatment-naïve *ALK*+ NSCLC patients (Solomon 2014; Lu 2016) as compared to the incidence of CNS metastasis (13%-15%) in advanced treatment-naïve epidermal growth factor receptor (*EGFR*+) NSCLC patients (Rosell 2012; Park 2016; Schuler 2016). Furthermore, isolated CNS progression accounted for approximately 40% to 50% as the site of progression for *ALK*+ NSCLC patients (Ou 2014; Khozin 2015). Indeed, the incidence of CNS metastases increased with disease progression with successive treatment (Ou 2016; Shaw 2013a; Shaw 2014b, Shaw 2016a; Kim 2016a).

While crizotinib has demonstrated intracranial activity of approximately 18% in *ALK*+ NSCLC patients with untreated brain metastasis in a large-scale retrospective analysis of *ALK*+ NSCLC patients treated with crizotinib (Costa 2015), the second-generation ALK inhibitors such as alectinib, ceritinib, and brigatinib has demonstrated much higher intracranial activity (Ou 2016; Shaw 2013a; Shaw 2014b, Shaw 2016a; Kim 2016a; Kim 2016b). Additionally, while crizotinib may delay the time to treatment progression in *ALK*+ NSCLC patients with treated CNS metastases when compared to platinum/pemetrexed chemotherapy, it did not seem to prolong the time to treatment progression when compared to platinum/pemetrexed in *ALK*+ NSCLC patients without brain metastases at the time of treatment (Solomon 2016).

The incidence and pattern of CNS progression in *ROS1*+ NSCLC patients and in patients with solid malignancies harboring *NTRK* rearrangement is not well studied and documented given the lower incidence of these patients. However, it is more likely than not that the pattern of CNS progression will be similar to *ALK*+ NSCLC patients. Thus development of ROS1 and TRK inhibitors should also have activity against metastases in the CNS.

A key event of brain metastasis is the migration of cancer cells through the blood-brain barrier (BBB). It was reported that SRC was hyperactivated in brain-seeking breast cancer cells derived from human cell lines or from patients' brain metastases (Zhang 2013). Activating SRC signaling is important for promoting tumor cell extravasation into the brain parenchyma *via* permeabilization of BBB. FAK is enriched in brain blood vessels compared to surrounding cell types, phosphorylated in response to vascular endothelial growth factor (VEGF), and forms signaling complexes with SRC and integrins in VEGF-induced signaling (Lee 2010). It was found that the brain vascular permeability was proportionally increased with brain tumor burden, and FAK was a mediator of tumor-induced vascular permeability in the brain (Lee 2010). The conditional endothelial-specific deletion of FAK in the tumor-bearing mice resulted in reduced vascular permeability, partial restoration of cell-cell junction proteins in the tumor vessels and astrocyte-endothelial interactions in tumors, leading to reduced tumor growth in brain (Lee 2010). Therefore, targeting SRC/FAK signaling will be a good strategy to restore BBB structure/function in cancer to overcome/prevent CNS metastasis.

Approved v1.0

2 TPX-0005

TPX-0005 is designed to simultaneously overcome multiple resistance mechanisms by a combination of careful chemical design to create a compact molecule that is not prone to mutations and a polypharmacology profile that addresses the critical signaling pathways in drug resistance. TPX-0005 is a potent small-molecule inhibitor of ALK, ROS1, and TRK family with a rigid three-dimensional macrocyclic structure and much smaller size than current ALK, ROS1 and TRK family inhibitors. TPX-0005 is completely located inside adenine binding boundary **WITHOUT** a blue colored solvent exposure motif as illustrated in Table 4, leading to efficiently binding with the active kinase conformation and circumventing the steric interference from clinical resistant mutations, especially the gatekeeper and solvent front mutations of ALK, ROS1 and TRK family kinases. At the clinically relevant concentrations, TPX-0005 also inhibits JAK2, SRC and FAK that are important targets in modulating multiple resistance mechanisms, including bypass signaling, EMT, cancer stemness, and metastasis. Therefore, the unique compact macrocyclic structure and polypharmacology profile of TPX-0005 will provide opportunities in clinic to overcome multiple resistance mechanisms, including a broad spectrum of acquired mutations, bypass signaling, EMT, cancer stemness, and metastasis, for the treatment of patients with advanced solid tumors harboring *ALK*, *ROS1*, or *NTRK1-3* rearrangements.

2.1 *In vitro* and *In vivo* Inhibitory Activity of TPX-0005

2.1.1 Activity against Wild-type (WT) and Mutated ALK

Designed to equally inhibit wild-type (WT) and most mutated ALKs, TPX-0005 was found to inhibit the recombinant kinase activities of ALK (the half maximal inhibitory concentration IC_{50} 1.04 nM) and its mutants, including ALK G1202R, L1196M, F1174L, F1174S, C1156Y, S1206R, L1152R, R1275Q, 1151T Ins, T1151M, and G1269A with IC_{50} values of 1.21, 1.08, 1.46, 1.02, 0.932, 0.525, 1.23, 2.79, 2.16, 0.491, and 5.50 nM, respectively. In Karpas299 cell based assays, TPX-0005 suppressed phosphorylation of NPM-ALK (IC_{50} < 3 nM) and its downstream signaling effectors STAT3, AKT and ERK, leading to anti-proliferation at an IC_{50} of 23.7 nM. TPX-0005 suppressed ALK phosphorylation with IC_{50} values around 30 nM in the engineered NIH3T3 and Ba/F3 cells expressing EML4-ALK v1 or EML4-ALK v1 G1202R. Furthermore, TPX-0005 inhibited cell proliferation of engineered Ba/F3 cells expressing EML4-ALK v1 (IC_{50} 21.1 nM) or its clinical mutant, e.g. EML4-ALK v1 G1202R, L1196M, F1174C, C1156Y and L1152P with IC_{50} values of 20.5, 74, 54.2, 24, and 60.1 nM, respectively.

Consistently, TPX-0005 demonstrated marked tumor growth inhibition (94%) in *in vivo* Karpas299 tumor model with a trough free plasma concentration of 19 nM, corresponding to ~90% inhibition of ALK phosphorylation, achieved at a BID (twice a day) dose of 50 mg/kg. Marked tumor growth inhibition was achieved by TPX-0005 in NIH3T3 EML4-ALK v1 WT (141%) tumor model and Ba/F3 tumor models expressing EML4-ALK v1 WT (154%) or G1202R (99%) with a BID dose of 75 mg/kg without overt toxicity, corresponding to a free plasma concentration at the end of dosage interval (C_{trough}) of 13.3 nM. Overall, TPX-0005 demonstrated effective inhibition against both WT and clinically mutated ALKs, especially the solvent front mutation ALK G1202R in *in vitro* and *in vivo* ALK assays and tumor models. A free plasma C_{trough} of 13.3 nM is able to achieve tumor regression and complete tumor growth inhibition in Ba/F3 EML4-ALK v1 WT and G1202R tumor models, respectively.

Approved v1.0

2.1.2 Activity against WT and Mutated ROS1

TPX-0005 was found to potently inhibit the kinase activities of ROS1 (IC_{50} 0.0706 nM) and its solvent front mutation G2032R (IC_{50} 0.46 nM) in biochemical assay. TPX-0005 effectively suppressed the phosphorylation of CD74-ROS1, and its mutants G2032R, L2026M, D2033N with estimated IC_{50} values of <1, 3, 10, and 1 nM, respectively, in engineered NIH3T3 cell based assays. Meanwhile, TPX-0005 inhibited cell proliferation of engineered Ba/F3 cells expressing SDC4-ROS1, SDC4-ROS1 G2032R, CD74-ROS1, CD74-ROS1 G2032R, CD74-ROS1 L2026M, or CD74-ROS1 D2033N with IC_{50} values of <0.2, 3, <0.2, 8.4, 10, and 0.15 nM, respectively.

In *in vivo* tumor models, a BID dose of 15 mg/kg of TPX-0005 resulted in a free trough plasma concentration of 12.7 nM, leading to a complete tumor regression in Ba/F3 CD74-ROS1 xenograft tumor model, and 97% inhibition of CD74-ROS1 G2032R phosphorylation and 99% inhibition of tumor growth in Ba/F3 CD74-ROS1 G2032R xenograft tumor model. A BID dose of 75 mg/kg resulted in a complete tumor regression in Ba/F3 CD74-ROS1 G2032R tumor model without causing animal toxicity. Overall, TPX-0005 is a potent inhibitor against ROS1 fusions and the corresponding clinical resistance mutations, especially the solvent front G2032R mutation. A free plasma C_{trough} of 12.7 nM is able to achieve complete tumor regression and tumor growth inhibition in Ba/F3 CD74-ROS1 WT and G2032R tumor models, respectively.

2.1.3 Activity against Wild-type and Mutated TRK

TPX-0005 had a similar binding affinity with TRKA, TRKB, and TRKC with K_d values of 0.019, 0.054 and 0.088 nM, respectively. TPX-0005 potently inhibited the enzymatic kinase activities of TRKA (IC_{50} 0.826 nM), TRKB (IC_{50} 0.0517 nM) and TRKC (IC_{50} 0.0956 nM).

Correspondently, TPX-0005 suppressed the phosphorylation of fusion TRKs and their solvent front mutants with IC_{50} values in a range of 0.01 to 3 nM in KM12 and engineered NIH3T3 cells. TPX-0005 inhibited cell proliferation of KM12 cells (IC_{50} 0.2 nM) and engineered Ba/F3 stable cells expressing fusion TRKs or solvent front mutants, e.g. LMNA-TRKA G595R (IC_{50} 0.4 nM), TEL-TRKB (also called EVT6-TRKB) G639R (IC_{50} 0.6 nM) and TEL-TRKC (ETV6-TRKC) G623R (IC_{50} 0.39 nM).

Meanwhile, TPX-0005 demonstrated a dose-dependent inhibition of tumor growth in the KM12 tumor model, with a free trough plasma concentration of 6.9 nM corresponding to >90% tumor growth inhibition at a BID dose of 3 mg/kg. Additionally, a BID dose of 15 mg/kg of TPX-0005 produced a free trough plasma concentration of 22.7 nM, leading to 128% and 97% tumor growth inhibition in NIH3T3 LMNA-TRKA WT and G595R mutant xenograft tumor models, respectively. A BID dose of 60 mg/kg of TPX-0005 led to tumor regression in NIH3T3 LMNA-TRKA G595R tumor model without causing animal toxicity. Overall, TPX-0005 is a potent pan-TRK inhibitor and markedly inhibits solvent front mutations in *in vitro* cellular assays and *in vivo* tumor models. A free plasma C_{trough} of 22.7 nM is able to achieve tumor regression and 97% tumor growth inhibition in NIH3T3 LMNA-TRKA WT and G595R tumor models, respectively.

2.1.4 Activity against Solvent Front Mutations of ALK, ROS1 and TRKA/B/C

TPX-0005 is designed to inhibit both WT and mutant kinases, especially solvent front mutations. The activities of TPX-0005 against WT and solvent front mutated ALK, ROS1 and TRKs are summaried in Table 5 and Figure 1. TPX-0005 demonstrated potent inhibition in *in vitro* cellular assays and in *in vivo* tumor models against the solvent front mutations of ALK, ROS1 and TRKs without a significant potency shifts when compared to the WT kinases.

Table 5 The Activities of TPX-0005 against WT and Solvent Front Mutated ALK, ROS1 and TRKs

Target	Auto-phosphorylation IC_{50} (nM)	Anti-proliferation IC_{50} (nM)	TGI%* (Tumor model)	Free C_{trough} (nM)
	NIH3T3 Cells	Ba/F3 Cells		
EML4-ALK v1 WT	30	21.1	154% (Ba/F3, SCID mice)	13
EML4-ALKv1 G1202R	30	20.5	99% (Ba/F3, SCID mice)	13
SDC4-ROS1 WT	ND	<0.2	139% (NIH3T3, nude mice)	23
SDC4-ROS1 G2032R	ND	3	ND	ND
CD74-ROS1 WT	<1	<0.2	200% (Ba/F3, SCID mice)	13
CD74-ROS1 G2032R	3	8.4	200% (Ba/F3, SCID mice)	13
CD74-ROS1 D2033N	1	0.15	ND	ND
LMNA-TRKA WT	<0.01	<0.2	128% (NIH3T3, nude mice)	23
LMNA-TRKA G595R	0.1	0.4	97% (NIH3T3, nude mice)	23
TEL-TRKB WT	0.1	<0.2	ND	ND
TEL-TRKB G639R	3	0.6	ND	ND
TEL-TRKC WT	ND	<0.2	ND	ND
TEL-TRKC G623R	ND	0.39	ND	ND

*TGI% >100% means tumor regression and 200% means complete tumor regression. ND = not determined. SCID = severe combined immune deficiency

Approved v1.0

Figure 1 The anti-tumor efficacy of TPX-0005 in WT and solvent front mutated ALK, ROS1 and TRKA tumor models.

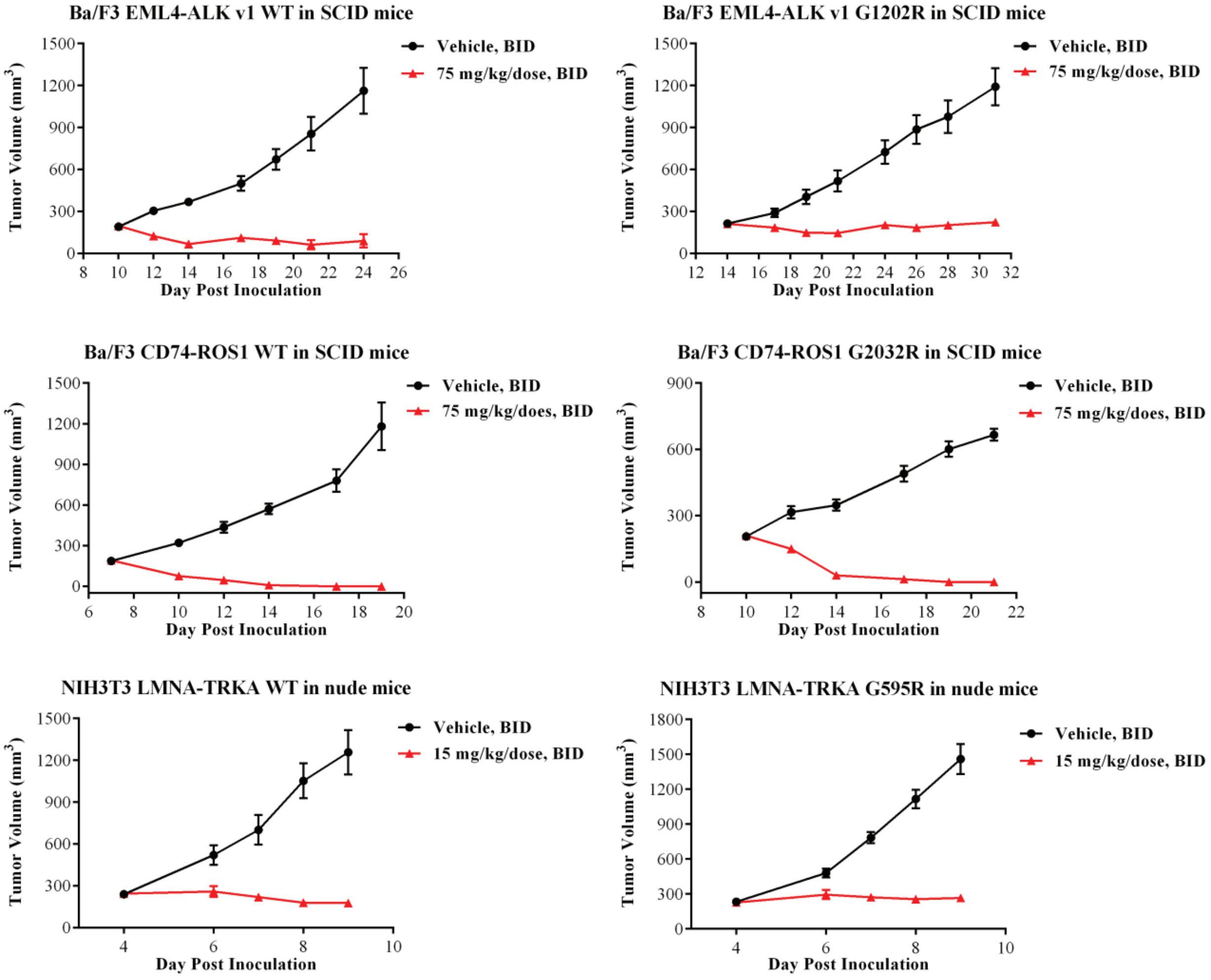

2.1.5 Activity against SRC, FAK and JAK2 for Modulation of Bypass Signaling, EMT, Cancer Stemness and Metastasis

Kinase selectivity of TPX-0005 over 456 human kinases (WT and mutants) was evaluated in kinase binding assay using the KINOMEscan® profiling platform, and the hits were further determined for IC_{50} values in enzymatic kinase inhibition assays.

It was found that TPX-0005 also inhibited JAK2, SRC and FAK with IC_{50} values of 1.04, 5.29 and 6.96 nM, respectively in enzymatic kinase inhibition assays. TPX-0005 inhibited JAK2 signaling (pSTAT5 IC_{50} 139 nM) and cell proliferation (IC_{50} 169 nM) of SET2 cells harboring oncogenic JAK2 V617F mutation.

H2228 lung cancer cell line endogenously expresses EML4-ALK v3 and was found to be resistant to ALK inhibitor TAE684 (Koivunen 2008). Upregulation of EGFR (Isozaki 2016), mesenchymal marker vimentin (Voena 2016), and cancer stem-like marker CD44 (Jokinen 2014)

Approved v1.0

was reported in H2228 cells, likely leading to intrinsic resistance to ALK inhibitors. In the anti-proliferation assays, both crizotinib and ceritinib were found to be resistant in H2228 cells with IC_{50} values around 1000 nM, consistent with the literature reports. However, TPX-0005 was able to overcome the intrinsic resistance with an IC_{50} of 100 nM. Additonally, TPX-0005 suppressed the phosphorylation of SRC, FAK and paxillin with IC_{50} values in a range of 80 nM - 100 nM, and downregulated the expression of EGFR, CD44, and vimentin with IC_{50} values around 100 nM in H2228 cells.

In addition, TPX-0005 inhibited the phosphorylation of the oncogenic transcription/translation factor YB-1 with an IC_{50} value around 100 nM in H2228 cells. YB-1 is involved in many aspects of gene expression control that lead to tumor cell growth and drug resistance (Lasham 2013), including modulation of EGFR upregulation (Stratford 2007), EMT (Castellana 2015), and cancer stemness (Kang 2013). Therefore, it was postulated that inhibition of SRC/FAK by TPX-0005 suppressed the phosphorylation of YB-1, leading to the downregulation of EGFR, CD44 and vimentin, and eventually to anti-proliferation effect on H2228 cells. TPX-0005 demonstrated *in vitro* anti-metastatic activity by inhibiting cell migration in both H2228 cells and HT1080 human fibrosarcoma cells.

Overall, TPX-0005 demonstrated inhibition of JAK2, SRC and FAK in cellular assays, and has the potential in overcoming resistance based on the mechanisms of bypass signaling, EMT, cancer stemness, and metastasis.

2.2 TPX-0005 Pre-Clinical Safety Data

Toxicology studies in 2 mammalian species (rat and monkey) have been conducted to support the safety of this first-in-human (FIH) clinical trial. Please refer to TPX-0005-01 Investigator's Brochure (IB) for detailed description.

[redacted]

Approved v1.0

[redacted]

[redacted]

2.3 Benefit to Risk Assessment

The nonclinical safety profile of TPX-0005 has been extensively evaluated in the rats and monkeys to support progression into clinical trials in advanced cancer indications. [redacted] TPX-0005 is also a potential phototoxicant based on Ultra Violet –B (UVB)-absorbing properties. The nonclinical safety findings related to TPX-0005 administration can be

monitored and are considered clinically manageable or acceptable risks in the intended patient population.

The primary pharmacodynamics data demonstrated that TPX-0005 potently inhibited the wild type and mutated ALK, ROS1 and TRKs. The cellular anti-phosphorylation IC_{50} values and the anti-proliferation IC_{50} values ranged from <1 nM for WT and solvent front mutated fusion TRKs and WT fusion ROS1s, <10 nM for solvent muted fusion ROS1s, to <30 nM for WT and solvent front mutated EML4-ALKs. A free plasma $C_{trough} \leq 23$ nM can lead to either complete tumor regression or >90% tumor growth inhibition in tumors driven by fusion ALK, ROS1, TRKs, or their solvent front mutants. The anti-tumor efficacy of TPX-0005 was dose-dependent and demonstrated strong correlations to inhibition of ALK, ROS1 and TRK phosphorylation, and there was no body weight loss observed in any of the *in vivo* efficacy studies. Collectively, the data indicate that TPX-0005 is a potent inhibitor of WT and mutated ALK, ROS1, and TRK family kinase. The small and rigid three-dimensional macrocyclic structure allows TPX-0005 target active kinase conformation and systematically overcome a broad spectrum of clinical resistance mutations in an structurally expected manner, especially the solvent front mutations ALK G1202R, ROS1 G2032R, TRKA G595R, and TRKC G623R. Additionally, the potent kinase inhibitory activities against JAK2/SRC/FAK provide a unique polypharmacology profile to TPX-0005 for combatting multiple resistance mechanisms simultaneously. Therefore, the benefit to risk assessment is in favor of studying TPX-0005 in patients with advanced solid tumors harboring *ALK*, *ROS1* or *NTRK1-3* rearrangements.

2.4 Safety Consideration in the Clinical Trial

As TPX-0005 absorbs light in the 290-700 nm range (λmax = 290 nm) and the Molar Extinction Coefficient (MEC) value calculated for this absorption maximum (7100 L $mol^{-1}cm^{-1}$) exceeds the 1000 L $mol^{-1}cm^{-1}$ value stipulated in the International Council for Harmonisation (ICH) S10 Tripartite Guideline[1], TPX-0005 is considered to have sufficient photoreactive potential to result in direct phototoxicity. Therefore, additional *in vitro* and/or *in vivo* studies to assess the photosafety risk of TPX-0005 will be conducted per ICH S10 guidance[1] as TPX-0005 proceeds through clinical development. Until the photosafety of TPX-0005 has been adequately demonstrated, photosafety risk in the clinical setting will be managed by the use of light protective measures. Phase 1/2 volunteers should be instructed to avoid extensive sun exposure, phototherapy, or use of a tanning salon within a prudent amount of time prior to and following trial participation.

[redacted]

Approved v1.0

[redacted]

[redacted]

3 STUDY DESIGN

3.1 Study Overview

This is a Phase 1/2, open-label, multi-center, multiple-dose, dose-escalation, safety, PK, pharmacodynamic (PD), and anti-cancer efficacy exploration study of TPX-0005 as a single agent in patients with advanced *ALK+*, *ROS1+, NTRK1+, NTRK2+,* or *NTRK3+* solid malignancies. This clinical study will consist of 2 parts, Phase 1 and Phase 2.

3.2 Phase 1a Dose Escalation

The Phase 1a portion of the study will estimate the maximum tolerated dose (MTD) for single-agent TPX-0005 in dose escalation cohorts in patients with advanced *ALK+, ROS1+, NTRK1+, NTRK2+*, or *NTRK3+* solid malignancies.

To understand the single-dose PK of TPX-0005, a lead-in period preceding the continuous daily dosing will be conducted. A **SINGLE** lead-in dose of TPX-0005 will be given on Day -3. No trial drug will be administered during the interval between the lead-in single dose and Day 1 of the first cycle. The timing, dosage regimen, and PK time points for lead-in period and subsequent doses may be modified based on the emerging PK data.

3.2.1 Starting Level for Cycle 1

The starting dose for TPX-0005 in the first-in-patient trial in cancer patients has been determined to be 40 mg once daily, [redacted] The provisional dose levels of TPX-0005 are shown in Table 6.

The first cycle of treatment for the intense PK determination will be 21 days. Subsequent cycles will be 28 days.

Approved v1.0

3.2.2 Dose Escalation Levels

Table 6 Provisional Dose Levels

Dose Level	Proposed Dose Level (mg/day)[a]	Increment from Previous Dose (%)
-1[b]	20 mg/day	(50% decrease)
1	40 mg/day	Starting Dose
2	80 mg/day	100%
3	160 mg/day	100%
4	240 mg/day	50%
5	320 mg/day	33%
6	400 mg/day	25%
7	500 mg/day[c]	25%

[a] It is possible for some dose levels to be skipped or additional dose levels to be added during the course of the study.
[b] Dose level -1 represents a dose that may be evaluated if dose level 1 is poorly tolerated. No dose de-escalation below this level is planned for this study. If dose level -1 is poorly tolerated, then the study will be terminated.
[c] TPX-0005 doses higher than 500 mg may be allowed depending on the observed safety, PK and PD.

Cycle 1 doses will be administered according to the dose escalation schedule listed in Table 6. Dose escalation will continue until dose-limiting toxicity (DLT) is reached or PK saturation is reached (Section 3.2.3.4). Patients will be dosed on a flat scale of mg/day and not by individual weight or body surface area.

All dose levels beyond cohort 1 may change with emerging safety and PK. The planned dose escalations will not exceed doubling of the dose in principle. Initially, QD dosing schedule will be used. As soon as clinical safety and PK/PD data become available, it may be preferable to give TPX-0005 BID schedule. If the totality of safety, PK, and other data indicate such schedule is deemed necessary, dose escalation may proceed with BID dosing schedule in a new cohort of patients. Evaluation of safety, PK, preliminary efficacy, biomarker data at the new BID dosing schedule will be performed.

3.2.3 Criteria for Dose Escalation and Determination of MTD

3.2.3.1 Maximum Tolerated Dose (MTD) Definition

For any given schedule, the MTD is the highest drug dosage not expected to cause DLT in more than 33% of the treated patients in the first 21 days of continuous TPX-0005 treatment under that schedule. Adverse events (AEs) and laboratory abnormalities considered to be DLTs are defined in Table 7.

Approved v1.0

3.2.3.2 Dose-escalation Process

The following provides a full description of the procedure for patient accrual and provisions for dose escalation/de-escalation decisions for the study

1. In the first cohort, a delay of at least 7 days will be mandatory between the administration of the first (single) dose to the first patient and administration of first (single) dose to subsequent patients (i.e., at least a 7-day gap between patients initiating study drug in all sites). Provided there are no safety concerns after completion of the first cohort, ensuing cohorts of patients will begin study drug as suitable patients are identified. If ambiguous findings occur after the first cohort, TP Therapeutics may choose to stagger the start of dosing in subsequent cohorts.
2. Patients will be enrolled to ensure a minimum of 3 and a maximum of 6 evaluable patients per cohort. Dose escalation and de-escalation will follow the scheme below, according to the following logic:
    - If no DLT is observed (for definition see Section 3.2.3.4) in a cohort of 3-6 evaluable patients then dose escalation may occur. Dose increases will be permitted after review of data from a minimum of 3 evaluable patients has been performed.
    - If one patient experiences a DLT in a group of 3 or more evaluable patients, then the cohort will be expanded to include 6 evaluable patients. If only one DLT is observed in the complete cohort of 6 evaluable patients, then dose escalation may occur.
    - If 2 or more patients experience a DLT in a group of up to 6 patients, irrespective of the number of patients enrolled, the dose will be considered not tolerated and recruitment to the cohort and dose escalation will cease. A lower intermediary dose (de-escalation) may be considered in order to better define the MTD.
3. A patient will be considered as evaluable for dose determination if they experience a DLT during cycle 1 or the preceding PK lead-in period, or meet the minimum treatment and safety evaluation requirements for the first cycle (for definition of the dose--determining set, see Section 9.1).

Dose administration (dose escalation and de-escalation) of the Phase 1 portion of the trial stops if: (1) there is evidence in the dose-exposure relationship of saturation of absorption; (2) at least 12 patients have been treated at a dose that is predicted to be the MTD or (3) all doses appear to be overly toxic and the MTD cannot be determined in the current trial.

Once the MTD of TPX-0005 has been estimated with confidence, in case fewer than 12 patients have been treated at the dose identified as MTD, enrollment will continue to reach at least 12 patients treated at the MTD to confirm the recommended Phase 2 dose (RP2D).

3.2.3.3 Dose Cohort Modification

Dose level decisions during the escalation period are not limited to the levels specified in Table 6. Provisional dose levels may be assigned to separate cohorts of patients based on the observed PK and AEs. Intermediate dose levels may be selected for subsequent new cohorts of patients.

Approved v1.0

The dose level may be lower or higher than a dose level associated with a designated cohort listed in Table 6. To further characterize the safety (e.g., suspected specific treatment-related adverse events) or PK/PD of TPX-0005, a dose level that is considered acceptably safe (i.e., shown to be lower than potential MTD) may be expanded. Dose escalation may be terminated at any time based on emerging safety concerns without establishing the MTD.

3.2.3.4 DLT Definition

Clinically relevant toxicities will be those assessed as unrelated to disease, disease progression, intercurrent disease, or concomitant medications. These will be evaluated according to National Cancer Institute Common Terminology Criteria for Adverse Events (NCI CTCAE) v4.03.

DLT is defined as an adverse event or abnormal laboratory value assessed as unrelated to disease progression, intercurrent illness, or concomitant medications that meets any of the follow criteria in Table 7.

For the purpose of dose escalation decisions, only DLTs occurring during the first cycle of treatment including PK lead-in period will be considered. Patients who discontinue treatment before completing Cycle 1 (i.e., the DLT evaluation time window) or receive less than 16 of the planned first 21 continuous TPX-0005 doses for reasons other than treatment-related toxicity (e.g., missed appointments, misplaced study drug supplies, development of coexisting medical condition rendering the patient unable to swallow medication, development of rapidly progressing disease) will be replaced for DLT evaluation but will remain in the overall safety and efficacy analyses.

Table 7 Criteria for Defining Dose-limiting Toxicities

**Toxicity**	**Any of the following criteria**
**Toxicities resulting in an excessive number of missed doses**	Inability to deliver > 75% of the planned doses in Cycle 1 treatment (i.e., at least 16 doses in a 21-day cycle) because of toxicity at least related to TPX-0005
**Hematology**	CTCAE grade ≥ 4 neutropenia (ANC < 0.5 × 10^9/L)
	CTCAE grade ≥ 4 thrombocytopenia (platelets < 25 × 10^9/L)
	CTCAE grade ≥ 3 anemia (Hgb < 8.0 g/dL)
	CTCAE grade ≥ 3 febrile neutropenia (defined as ANC < 1000/mm^3 with a single temperature of ≥ 38.3°C [≥ 101°F] or a sustained temperature of ≥ 38°C [≥ 100.4°F] for > 1 hour)
**Renal**	CTCAE grade ≥ 3 creatinine increase (> 3 × ULN)
**Hepatic**	CTCAE grade ≥ 3 total bilirubin elevation (> 3 × ULN)
	CTCAE grade ≥ 2 total bilirubin elevation (> 1.5 × ULN) **AND** CTCAE grade ≥ 2 ALT or AST elevation (> 3 × ULN)
	CTCAE grade ≥ 3 ALT elevation (> 5 × ULN)
	CTCAE grade ≥ 3 AST elevation (> 5 × ULN)

Toxicity	Any of the following criteria
**Pancreatic**	CTCAE grade ≥ 2 pancreatitis
	CTCAE grade ≥ 3 amylase or lipase elevation
**Cardiac**	CTCAE grade ≥ 3
**Dermatologic**	CTCAE grade ≥ 2 phototoxicity
**Other adverse events**	CTCAE grade ≥ 3 vomiting or nausea despite optimal anti-emetic therapy
	CTCAE grade ≥ 3 diarrhea despite optimal anti-diarrhea treatment
	Any CTCAE grade ≥ 3 AE, except for the exclusions noted below
	In view of the Investigators and TP Therapeutics any other unacceptable toxicity encountered
**Exceptions to DLT criteria**	CTCAE grade 3 or 4 elevations in alkaline phosphatase
	CTCAE grade 3 or 4 electrolytes abnormalities that are adequately managed by IV or PO supplementations
	< 7 days of CTCAE grade 3 fatigue

NCI CTCAE version 4.03 will be used for all grading.
Patients may receive supportive care (e.g., PRBCs) as per local institution guidelines
Optimal therapy for vomiting or diarrhea will be based on institutional guidelines, with consideration of the prohibited medications in this protocol.

3.2.3.5 Follow-up Dose-Limiting Toxicities

When treatment is interrupted or permanently discontinued due to an AE or abnormal laboratory value, the patient/subject must be followed at least once a week for 4 weeks, and subsequently at a maximum interval of 4 weeks, until resolution or stabilization of the event, whichever comes first. If a patient requires a dose delay due to study drug-related toxicity of > 28 days from the intended day of the scheduled dose, then the patient must be discontinued from the study. However, the patient will be followed up for toxicity as previously described. All patients will be followed for AEs and serious AEs (SAEs) for 28 days following the last dose of TPX-0005.

3.2.3.5.1 Hematologic Toxicities

If CTCAE grade ≥ 3 neutropenia, CTCAE grade ≥ 3 thrombocytopenia, or CTCAE grade ≥ 3 anemia have been demonstrated, these parameters must be repeated at least twice a week until resolution to CTCAE grade ≤ 1 neutropenia, CTCAE grade ≤ 1 thrombocytopenia, or CTCAE grade ≤ 1 anemia, and then at least weekly until either resolution to allow for re-treatment or until stabilization.

Approved v1.0

3.2.3.5.2 Renal Toxicity

If serum creatinine > 3 × upper limit of normal (ULN) has been demonstrated, this parameter must be repeated at least twice a week until resolution to CTCAE grade ≤ 1, and then at least weekly until either resolution to allow for initiation of re-treatment or until stabilization.

3.2.3.5.3 Hepatic Toxicity

If total bilirubin >3 × ULN or CTCAE grade ≥ 3 aspartate aminotransferase (AST) (also called serum glutamic oxaloacetic transaminase [SGOT])/ Alanine aminotransferase (ALT) (also called serum glutamic pyruvic transaminase [SGPT])/alkaline phosphatase (ALP) has been demonstrated, these parameters must be repeated at least twice a week until resolution to CTCAE grade ≤ 1 (or grade ≤ 2 if liver metastasis present), and then at least weekly until either resolution to allow for initiation of re-treatment or until stabilization.

Patients with total bilirubin > ULN (any duration) should have fractionation of bilirubin into total/direct or indirect/direct components and any additional work-up as clinically indicated by these results. Follow-up of hyperbilirubinemia should proceed as per the guidelines above, irrespective of the results of fractionation.

3.2.3.5.4 Non-laboratory Toxicity

Patients who experience non-laboratory DLTs who are not described in the above sections must be evaluated at least once a week following demonstration of the toxicity until resolution of the toxicity to allow for re-treatment, or stabilization of the toxicity.

3.2.3.6 Dose Modification and Dose Delay

For patients who experience DLT with TPX-0005, dose adjustments are permitted if it is considered in the best interest of the patient to continue therapy after discussing with the Medical Monitor and TP Therapeutics.

Patients who cannot be re-treated with TPX-0005 should have weekly follow-ups that include a physical examination, vital signs including weight, Eastern Cooperative Oncology Group (ECOG) performance status (PS), electrocardiograms (ECGs), and assessment of AEs and concomitant medication. Following a DLT or toxicity, hematology, renal, and liver function tests should be performed as appropriate.

If a patient requires a dose delay of > 28 days from the intended day of the next scheduled dose of TPX-0005, then the patient must be discontinued from the study. Patients, who discontinue from the study for a study drug-related AE or an abnormal laboratory value must be followed as described in Section 9.4.

For each patient, a maximum of 2 dose reductions will be allowed after which the patient should be discontinued from the study. For each patient, once a dose level reduction has occurred, the dose level may not be re-escalated during subsequent treatment cycles with TPX-0005. Dose reduction for TPX-0005 means treatment at a lower TPX-0005 dose level. Planned dose levels

for TPX-0005 are listed in Table 6. All interruptions or changes to study drug administration must be recorded on the study clinical report form (CRF).

[redacted]

3.2.3.8 *Intra-patient Dose Escalation*

Intra-patient dose escalation is not permitted during the first 4 cycles of treatment if there is no documented disease progression by response evaluation criteria in solid tumors (RECIST) version 1.1. After the fourth cycle is completed, individual patients may be considered for treatment at a dose of TPX-0005 higher than the dose to which they were initially assigned. In order for a patient to be treated at a higher dose of TPX-0005, he or she must have received the lower dose for at least 4 cycles of therapy without a drug-related toxicity of CTCAE grade ≥ 2. Observation of CTCAE grade ≤ 3 laboratory abnormalities not requiring dose modifications (as identified in Table 7) would be permitted. Moreover, the new, higher dose with which the patient is to be treated must be a dose that has completed evaluation where at least 3 patients have completed Cycle 1 with TPX-0005 without having a DLT and that does not exceed the MTD or the RP2D.

For patients who had documented disease progression, intra-patient dose escalation is permitted if he or she has completed Cycle 1 without any DLT and satisfies all the other conditions listed above.

Any further increases after the initial intra-patient dose escalation are subject to the same rules as for the initial intra-patient escalation. Consultation with TP Therapeutics must occur prior to any intra-patient dose escalation decision. These changes must be recorded on the study CRF.

3.3 Phase 1b Food Effect Sub-study

The effect of food on the pharmacokinetics of TPX-0005 will be evaluated in a food effect sub-study. The testing order for fed versus fasted conditions will be as follows: the first half of the patients to participate in this sub-study will be tested under fed followed by fasted conditions, the next half of the patients will be tested under fasted followed by fed conditions. Patients who have had a gastrectomy or have dietary or other restrictions that preclude a 10-hour overnight fast (water permitted) or consumption of the required high-fat, high-calorie meal will not participate in this sub-study.

Approved v1.0

The effect of a high-fat, high-calorie breakfast on TPX-0005 pharmacokinetics will be studied. The food effect sub-study will commence at the dose level where preliminary anti-tumor activity (partial response) was observed by the Investigator. The food effect sub-study will then continue at subsequent dose escalation/de-escalation levels. A total of 6 patients will be enrolled at each dose level. Additionally, a total of 12 patients will be enrolled at the presumed RP2D.

Each patient will serve as his/her own control. In half of the patients per dose level, TPX-0005 will be administered in the morning under either "fed" followed by "fasted" conditions on Cycle 0 Day -3 and on Cycle 1 Day 1, respectively. The other half of the patients per dose level TPX-0005 will be administered in the morning under either "fasted" followed by "fed" conditions on Cycle 0 Day -3 and on Cycle 1 Day 1, respectively. No trial drug will be administered during the interval after Cycle 0 Day -3 and before Cycle 1 Day 1.

3.4 Phase 2 Dose Expansion

The Phase 2 portion of the study will be with single-agent TPX-0005 at the identified MTD/RP2D and will enroll patients with advanced *ALK+*, *ROS1+*, *NTRK1+*, *NTRK2+*, or *NTRK3+* solid malignancies (primarily NSCLC patients). The Phase 2 will evaluate the anti-cancer activity of single-agent TPX-0005 in multiple sub-populations of patients with advanced *ALK+*, *ROS1+*, *NTRK1+*, *NTRK2+*, or *NTRK3+* solid malignancies and will further define the safety and PK profiles of single-agent TPX-0005 at the RP2D.

To evaluate the safety and tolerability of TPX-0005 in Japanese patients, a Japanese patient-only lead-in cohort (LIC) will be enrolled to evaluate TPX-0005 safety and PK in Japanese patients treated at a previously tested dose in Phase 1. The LIC will be conducted at Japanese sites concurrently with the Phase 2 portion of the study but will be considered separate from Phase 2 enrollment. Patients enrolled into the LIC will follow the same eligibility criteria, study procedures (unless otherwise specified), and patient withdrawal criteria as outlined in Phase 2. The TPX-0005 starting dose for the LIC will be communicated by letters to the site Investigators after identification of the RP2D in Phase 1. Initially up to 3 patients will be enrolled and treated. If a DLT is observed in 1 of the initial 3 treated patients, then 3 additional patients will be enrolled and treated. Patients who discontinue treatment before completing Cycle 1 (i.e., the DLT observation period) or receive less than 16 of the planned 21 TPX-0005 doses for reasons other than treatment-related toxicity (e.g., missed appointments, misplaced study drug supplies, development of coexisting medical condition rendering the patient unable to swallow medication, development of rapidly progressing disease) will be replaced for DLT evaluation but will remain in the overall safety and efficacy analyses. If ≤ 33% patients experience DLT (0/3 or 1/6), then the tested dose would be considered tolerable in Japanese patients, and the Phase 2 portion of the study will be opened to Japan sites. If > 33% patients experience DLT, then Japan sites will not join the Phase 2 study and a lower dose cohort may be explored if deemed necessary. Additional patients may be included for further safety and tolerability assessments in the LIC as appropriate.

The total enrollment of Phase 2 will range from approximately 357 patients to 404 patients. The patients will be accrued into 10 different expansion cohorts according to tumor of origin, prior treatment, and *ALK*, *ROS1*, or *NTRK* rearrangement (see Figure 2).

Approved v1.0

3.4.1 *ROS1*+ Solid Malignancy Expansion Cohorts

The *ROS1*+ expansion cohorts will enroll either ROS1i-naïve or ROS1i-refractory patients.

3.4.1.1 ROS1+ NSCLC Expansion Cohorts

There are 3 *ROS1*+ NSCLC expansion cohorts. For the ROS1i-naïve *ROS1*+ NSCLC expansion cohort ([EXP]-1), there is no limit to the number of prior regimens of cytotoxic chemotherapy or immunotherapy. There are 2 ROS1i-refractory *ROS1*+ NSCLC expansion cohorts. The first ROS1i-refractory *ROS1*+ NSCLC will enroll patients who are crizotinib-refractory only (EXP-2). The second ROS1i-refractory *ROS1*+ NSCLC expansion cohort will enroll patients who are refractory to 1 prior ROS1i that is not crizotinib or *ROS1*+ NSCLC patients who are refractory to 2 or more ROS1i (EXP-3). For the ROS1i-refractory *ROS1*+ NSCLC expansion cohorts (EXP-2, EXP-3), there is no limit to the number of prior regimens of cytotoxic chemotherapy or immunotherapy.

3.4.1.2 ROS1+ Non-NSCLC Expansion Cohort

Any patients with non-NSCLC solid malignancies harboring *ROS1* rearrangement will be allowed to enroll into *ROS1*+ non-NSCLC expansion cohort. For the ROS1i-naïve *ROS1*+ non-NSCLC expansion cohort, there is no limit to the number of prior regimens of cytotoxic chemotherapy or immunotherapy (EXP-9). This expansion cohort (EXP-9) will also enroll ALK inhibitor (ALKi)-naïve *ALK*+ non-NSCLC patients and TRKi-naïve *NTRK*+ solid malignancy patients. For the ROS1i-refractory *ROS1*+ non-NSCLC expansion cohort (EXP-10), there is no limit to the number of prior regimens of cytotoxic chemotherapy or immunotherapy. This expansion cohort (EXP-10) will also enroll ALKi-refractory *ALK*+ non-NSCLC patients and TRKi-refractory *NTRK*+ solid malignancy patients. This expansion cohort (EXP-10) will have no limit on the prior TKI specific to the particular *ALK*, *ROS1*, or *NTRK* rearrangements.

3.4.2 *ALK*+ Solid Malignancy Expansion Cohorts

The *ALK*+ expansion cohorts will enroll ALKi-naïve or ALKi-refractory patients.

3.4.2.1 ALK+ NSCLC Expansion Cohorts

For the ALKi-naïve NSCLC expansion cohort (EXP-4), these patients had to be treatment-naïve (no prior cytotoxic chemotherapy, immunotherapy, or TKI allowed) for their advanced/recurrent *ALK*+ NSCLC.

There are 4 expansion cohorts for the ALKi-refractory *ALK*+ NSCLC patients. These ALKi-refractory *ALK*+ NSCLC patients will be assigned to the respective ALKi-refractory expansion cohort depending on the number of prior ALKi therapy (EXP-5: 1 prior ALKi; EXP-6: 2 prior ALKi; EXP-7: 3 prior ALKi; EXP-8: more than 3 prior ALKi). Prior ALKi therapy is defined as actual ALKi received not as line of ALKi. For example, an *ALK*+ NSCLC patient who received crizotinib, ceritinib, and then crizotinib sequentially will count as 2 prior ALKi therapy and not 3 prior therapies.

3.4.2.2 *ALK+ Non-NSCLC Expansion Cohort*

Any patients with non-NSCLC solid malignancies harboring *ALK* rearrangement will be allowed to enroll into *ALK*+ non-NSCLC expansion cohort. Additionally, *ALK*-amplified neuroblastoma or neuroblastoma harboring known activating *ALK* mutations will be eligible to enroll (Bresler 2014).

For the ALKi-naïve *ALK*+ non-NSCLC expansion cohort (EXP-9), there is no limit to the number of prior regimens of cytotoxic chemotherapy or immunotherapy. For the ALKi-refractory *ALK*+ non-NSCLC expansion cohort (EXP-10), there is no limit to the number of prior regimens of cytotoxic chemotherapy or immunotherapy or number of prior ALK inhibitors (see Section 3.4.1.2).

3.4.3 *NTRK*+ Solid Malignancy Expansion Cohorts

Each of the *NTRK*+ expansion cohort will enroll TRKi-naïve and TRKi-refractory patients. For the TRKi-naïve *NTRK*+ expansion cohort (EXP-9), there is no limit to the number of prior regimens of cytotoxic chemotherapy. These patients will be enrolled into the same TKI-naïve expansion cohort (EXP-9) as ALKi-naïve *ALK*+ non-NSCLC solid malignancy patients and ROS1i-naïve *ROS1*+ non-NSCLC solid malignancy patients.

For the TRKi-refractory *NTRK*+ expansion cohort (EXP-10), there is no limit to the number of prior regimens of cytotoxic chemotherapy AND prior TRK inhibitors. These patients will be enrolled into the same TKI-refractory expansion cohort (EXP-10) as ALKi-refractory *ALK*+ non-NSCLC solid malignancy patients and ROS1i-refractory *ROS1*+ non-NSCLC solid malignancy patients.

Given the rarity of *NTRK*+ solid malignancies of a particular organ type, the statistical calculation and enrollment goal of the TRKi-naïve and TRKi-refractory expansion cohorts will be the same respectively independent of organ of origin. If TPX-0005 shows promising clinical efficacy in one particular *NTRK*+ tumor organ type, there is potential to expand that particular *NTRK*+ organ type as determined by TP Therapeutics.

Approved v1.0

Figure 2 Schema of TPX-0005-01 Trial

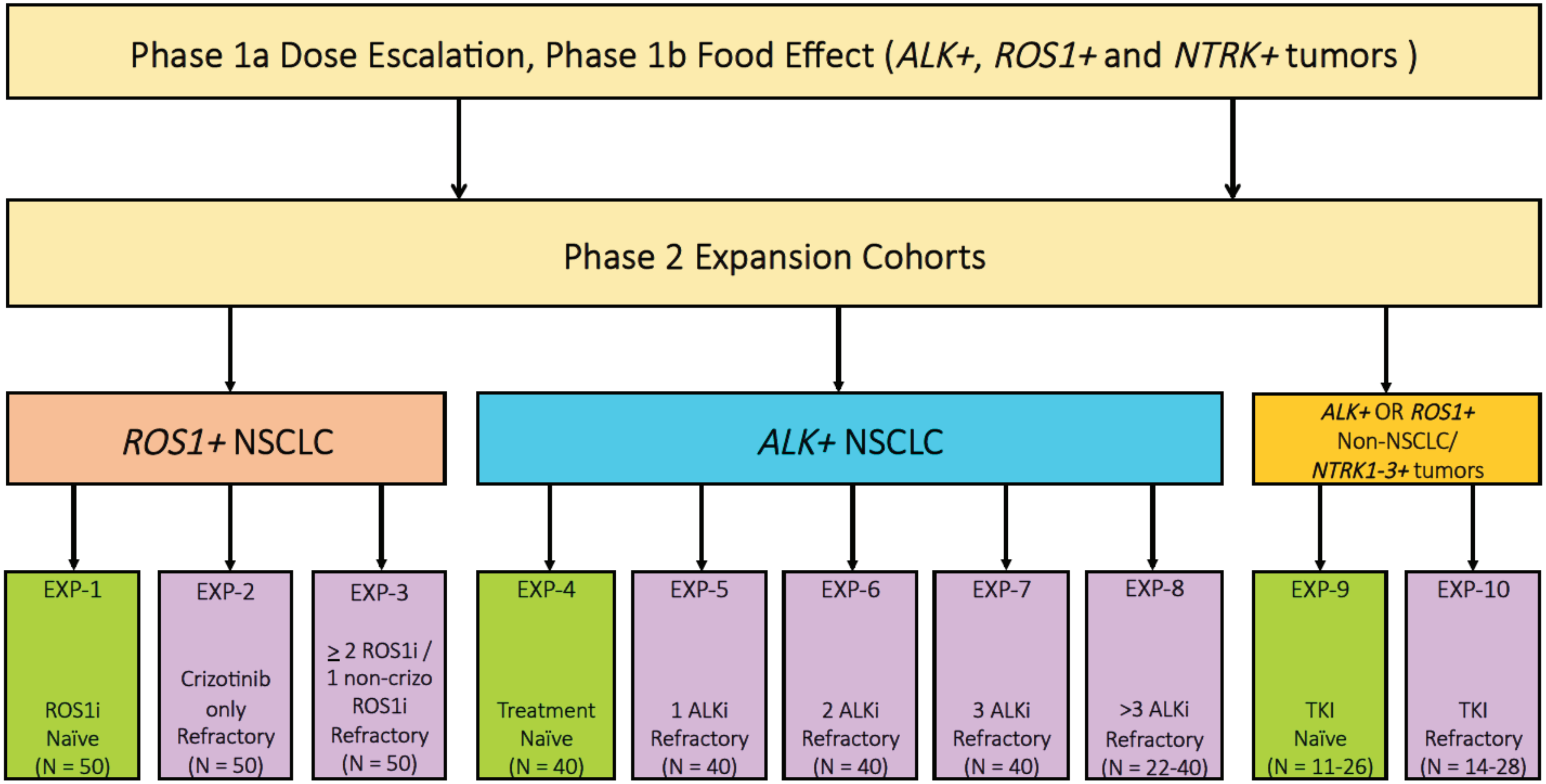

4 STUDY OBJECTIVES

4.1 Phase 1 Objectives

4.1.1 Phase 1 Primary Objectives

- To determine the first cycle DLTs of TPX-0005 given to adult patients with solid malignancies harboring an *ALK*, *ROS1*, *NTRK1*, *NTRK2*, or *NTRK3* gene rearrangement.
- To determine the MTD of TPX-0005 in adult patients with solid malignancies harboring an *ALK*, *ROS1*, *NTRK1*, *NTRK2*, or *NTRK3* gene rearrangement.
- To determine the biologically effective and R2PD of TPX-0005 for adult patients with solid malignancies harboring an *ALK*, *ROS1*, *NTRK1*, *NTRK2*, or *NTRK3* gene rearrangement.

4.1.2 Phase 1 Secondary Objectives

- To evaluate the safety and tolerability of TPX-0005 at various doses in patients with solid tumors that harbor an *ALK, ROS1*, *NTRK1*, *NTRK2*, or *NTRK3* gene rearrangement.
- To determine the effect of food on the PK of TPX-0005.
- To determine the preliminary objective response rate (ORR) of TPX-0005, in patients with solid tumors that harbor an *ALK, ROS1*, *NTRK1*, *NTRK2*, or *NTRK3* gene rearrangement.

4.2 Phase 2 Objectives

4.2.1 Phase 2 Primary Objectives

- To determine the ORR of TPX-0005 in each patient population expansion cohort of solid tumors that harbor an *ALK, ROS1, NTRK1*, *NTRK2*, or *NTRK3* gene rearrangement.

4.2.2 Phase 2 Secondary Objectives

- To determine the DOR, time to response (TTR), and clinical benefit rate (CBR) of TPX-0005, as assessed by BICR, in each patient population expansion cohort of solid tumors that harbor an *ALK, ROS1, NTRK1*, *NTRK2*, or *NTRK3* gene rearrangement.
- To estimate the PFS and overall survival (OS) of patients with solid tumors that harbor an *ALK, ROS1, NTRK1*, *NTRK2*, or *NTRK3* gene rearrangement treated with TPX-0005.
- To evaluate the safety and tolerability of TPX-0005 when administered at the RP2D in patients with solid tumors that harbor an *ALK, ROS1, NTRK1*, *NTRK2*, or *NTRK3* gene rearrangement.

Approved v1.0

- To determine the intracranial objective response rate (IC-ORR) of TPX-0005 and CNS progression-free survival (CNS-PFS) in patients presenting with measurable brain metastases at baseline, using RECIST version 1.1 assessment.
- To assess the population pharmacokinetics of TPX-0005 and to explore correlations between PK, response, and/or safety findings in patients with an *ALK, ROS1, NTRK1*, *NTRK2*, or *NTRK3* gene rearrangements
- To assess treatment-related symptoms and general health status using validated instruments of patient-reported outcomes (EORTC-QLQ-C30 and LC-13 when applicable) in patients treated with TPX-0005

4.2.3 Phase 2 Exploratory Objectives

- To assess for any potential differences in clinicopathologic presentation and response to TPX-0005 among various organ-specific solid malignancies harboring the same *ALK, ROS1, NTRK1, NTRK2,* or *NTRK3* gene rearrangements
- To assess for any potential differences in clinicopathologic presentation and response to TPX-0005 among the various fusion partner variants of *ALK, ROS1, NTRK1, NTRK2,* or *NTRK3* gene rearrangements among all patients and patients with specific organ-specific solid malignancy
- To assess the potential role of Bcl-2-like protein 11 deletion polymorphism as a primary resistance mechanism to TPX-0005 in TKI-naïve patients with *ALK, ROS1, NTRK1, NTRK2,* or *NTRK3* gene rearrangements
- To investigate the potential mechanisms of responsiveness and resistance to TPX-0005

5 PATIENT SELECTION

Both the inclusion and exclusion criteria are for Phase 1 and 2 unless otherwise stated.

5.1 Inclusion Criteria

1. Histologically or cytologically confirmed diagnosis of locally advanced or metastatic solid tumor (including non-Hodgkin Lymphoma) (Stage IV, American Joint Committee on Cancer v.7) that harbors an *ALK, ROS1, NTRK1, NTRK2,* or *NTRK3* gene rearrangement as by:

   - Any nucleic acid-based diagnostic testing method (e.g., next-generation sequencing [NGS], Sanger sequencing, reverse transcription-polymerase chain reaction) performed at a local clinical laboratory improvement amendments-certified or equivalently accredited diagnostic laboratory. All tests must have been performed according to the product's instructions for use (IFU).
   - Break-apart fluorescence *in situ* hybridization (FISH) is allowed for diagnosis of *ALK*, *ROS1*, *NTRK1*, *NTRK2*, and *NTRK3* rearrangements. For *ALK* rearrangement detection in NSCLC, the FISH test has to be performed using the FDA-approved Abbott Molecular's Vysis® ALK Break-apart FISH Probe Kit. All tests must have been performed according to the product's IFU.
   - Immunohistochemistry (IHC) detection of *ROS1*, *NTRK1*, *NTRK2*, and *NTRK3* rearrangement will not directly qualify patients. Archival tumor samples of these IHC-positive patients (ROS1, TRKA, TRKB, TRKC) will have to be sent to the TP Therapeutics's designated central laboratory for confirmation of *ROS1*, *NTRK1*, *NTRK2*, and *NTRK3* rearrangement prior to enrollment onto the trial. IHC detection of *ALK* rearrangement is allowed if performed using the Ventana ALK (D5F3) companion diagnostic assay. All tests must have been performed according to the product's IFU.
   - For NSCLC patients with *ALK* rearrangement not detected by an FDA-approved test, patient can still be consented and screening process started if the Investigator believes that *ALK* rearrangement detected by an FDA-approved test can be obtained within the 28 days screening period and submitted for eligibility determination after discussion with TP Therapeutics. For *ALK*+ NSCLC patients enrolled into the **Phase 1a Dose Escalation and Phase 1b Food Effect portion** of the study, the period for confirmation of the *ALK* rearrangement by an FDA-approved test can be extended until the end of Cycle 1 of the treatment schedule.
   - For patients enrolled with TP Therapeutics-approved non–FDA-approved tests, central laboratory confirmation of the specific gene alteration is not required before start of TPX-0005 treatment as long as adequate archival tissue is sent to the TP Therapeutics designated central laboratory.

Approved v1.0

- Special population:
    - ❖ Patients with *ALK*-amplified neuroblastoma are eligible to be enrolled into the *ALK*+ non-NSCLC expansion cohorts. *ALK* amplification will be defined as ALK/CEP2 FISH ratio > 2.2.
    - ❖ Patients with neuroblastoma harboring known activating *ALK* mutations. Any nucleic acid-based diagnostic testing method will be allowed. All tests must have been performed according to the product's IFU.

2. All patients must have archival tissue sample available and collected prior to enrollment. Formalin-fixed paraffin-embedded (FFPE) tissue block(s) from initial diagnosis that contain sufficient tissue to generate at least 10 (preferably 15), 5-micron thick unstained slides will be collected. If no FFPE block is available, then at least 10 (preferably 15) unbaked, 5-micron thick unstained slides containing FFPE tumor tissue must be provided. Specimens will be sent to the TP Therapeutics-designated central laboratories for *ALK*, *ROS1*, or *NTRK* rearrangement status confirmation.
3. ECOG PS 0–1.
4. Age ≥18 (or age ≥ 20 of age as required by local regulation).
5. Willing and able to provide written institutional review board (IRB)/institutional ethics committee-approved Informed Consent.
6. At least 1 measurable target lesion according to RECIST version 1.1. CNS-only measurable disease as defined by RECIST version 1.1 is allowed.
7. Prior cytotoxic chemotherapy for advanced or metastatic disease is allowed (except for the treatment-naïve *ALK*+ NSCLC expansion cohort). At the time of starting treatment with TPX-0005, at least 14 days must have elapsed after discontinuation of prior cytotoxic chemotherapy (or at least 42 days for prior nitrosoureas, mitomycin C, and liposomal doxorubicin) and all side effects from prior treatments must have resolved to grade ≤ 1 (NCI CTCAE Version 4.03) with the exception of alopecia.
8. Prior immunotherapy (e.g., anti-PD-1, anti-PDL1, anti-TIM3, anti-OX40) is allowed (except for the treatment-naïve *ALK*+ NSCLC expansion cohort). At the time of starting treatment with TPX-0005, at least 14 days must have elapsed after discontinuation of prior immunotherapy treatment and all immune-related side effects from prior treatments must have resolved to grade ≤ 1.
9. **For the Phase 1 dose escalation and food effect cohorts,** patients with solid tumors harboring *ALK*, *ROS1*, *NTRK1*, *NTRK2*, or *NTRK3* rearrangements are eligible. There is no limit to the number of prior chemotherapy, immunotherapy, or TKI regimens.
10. **For the Phase 2 dose expansion cohorts**, patients with solid tumors harboring *ALK*, *ROS1*, *NTRK1*, *NTRK2*, or *NTRK3* rearrangements will be assigned into 10 distinct expansion (EXP) cohorts provided all inclusion and exclusion criteria are met. The number of prior TKIs is defined as different TKI and not lines of therapy. For example, a patient who progresses on crizotinib, ceritinib, and then crizotinib will be considered as

Approved v1.0

disease progression on 2 prior ALKi and not 3 prior ALKi. Additionally, patients do not have to progress immediately on a TKI prior to enrollment into TPX-0005.

***ROS1*+ NSCLC**

- **EXP-1:** *ROS1+* NSCLC. No prior ROS1i allowed. Any prior lines of chemotherapy or immunotherapy are allowed.
- **EXP-2**: *ROS1+* NSCLC. Disease progression on crizotinib only. Any prior lines of chemotherapy or immunotherapy are allowed.
- **EXP-3:** *ROS1+* NSCLC. Disease progression on any 2 or more ROS1i OR disease progression on 1 non-crizotinib ROS1i. Prior lines of chemotherapy or immunotherapy allowed.

***ALK*+ NSCLC**

- **EXP-4:** *ALK+* NSCLC. No prior cytotoxic chemotherapy, immunotherapy, or TKI treatment for advanced metastatic or recurrent disease.
- **EXP-5:** *ALK+* NSCLC. Disease progression on any 1 ALKi only. Prior lines of chemotherapy or immunotherapy allowed.
- **EXP-6:** *ALK+* NSCLC. Disease progression on any 2 different ALKi only. Prior lines of chemotherapy or immunotherapy allowed.
- **EXP-7:** *ALK+* NSCLC. Disease progression on any 3 different ALKi only. Prior lines of chemotherapy or immunotherapy allowed.
- **EXP-8:** *ALK+* NSCLC. Disease progression on any 4 or more different ALKi. Prior lines of chemotherapy or immunotherapy allowed.

***ALK*+ non-NSCLC, *ROS1*+ non-NSCLC, or *NTRK*+ solid tumors**

- **EXP-9:** *ALK+* non-NSCLC, *ROS1+* non-NSCLC, or *NTRK+* solid tumors. No prior TKI for the corresponding gene rearrangement. For example, for a *ROS1+* patient, no prior ROS1i is allowed. Prior lines of chemotherapy or immunotherapy allowed.
- **EXP-10:** *ALK+* non-NSCLC, *ROS1+* non-NSCLC, or *NTRK+* solid tumors. Disease progression on prior TKI corresponding to the particular gene rearrangement. For example, a *NTRK+* patient must have disease progression on an TRKi. There is no limit on the number of prior TKI. Prior lines of chemotherapy or immunotherapy allowed.

11. At least 7 days must have elapsed since completion of treatment with the last ALKi, ROS1i, or TRKi prior to starting treatment with TPX-0005 for patients enrolling into the TKI-refractory expansion cohorts. All side effects from prior treatments with ALKi, ROS1i, and TRKi must have resolved to grade ≤ 1 prior to starting treatment with TPX-0005; however, the most immediate treatment prior to enrollment does not have to be a TKI.

- Prior ALKi allowed include crizotinib, ceritinib, alectinib, brigatinib, lorlatinib, ASP3026, TSR- 011, X-396
- Prior ROS1i allowed include crizotinib, ceritinib, lorlatinib, brigatinib, DS6051b, ASP3026, cabozantinib
- Prior TRKi allowed include entrectinib, LOXO-101, DS6051b. **Crizotinib is not considered a TRKi for the purpose of this trial**
- Other prior ALKi, ROS1i, and TRKi not listed above may be allowed after discussion with TP Therapeutics

12. Patients with asymptomatic CNS metastases (treated or untreated) and/or asymptomatic leptomeningeal carcinomatosis are eligible to enroll if they satisfy the following criteria:
    - Patients requiring steroids at a stable or decreasing dose (≤ 12 mg/day dexamethasone or equivalent) for at least 14 days are eligible. Patients on stable doses of levetiracetam (same dose for 14 days) are eligible to be enrolled.
    - A minimum of 14 days must have elapsed from the completion of whole brain radiation treatment (WBRT) before the start of treatment with TPX-0005, and all side effects (with the exception of alopecia) from WBRT are resolved to CTCAE grade ≤ 1.
    - A minimum of 7 days must have elapsed from the completion of stereotactic radiosurgery before the start of treatment with TPX-0005, and all side effects (with the exception of alopecia) from stereotactic radiosurgery are resolved to CTCAE grade ≤ 1.
13. Baseline laboratory values fulfilling the following requirements:

Absolute neutrophils count (ANC)	1500/mm^3 (1.5 × 10^9/L)
Platelets (PLTs)	100,000/mm^3 (100 × 10^9/L)
Hemoglobin	> 9.0 g/dL transfusions are allowed
Serum creatinine or creatinine clearance*	Within normal limits or > 40 mL/min
Total serum bilirubin	< 1.5 × ULN
Liver transaminases (ASTs/ALTs)	< 2.5 × ULN; < 5 × ULN if liver metastasis are present
Alkaline phosphatase (ALP)	< 2.5 × ULN; < 5 × ULN if liver and/or bone metastasis are present
Serum calcium, magnesium, and potassium	Normal or CTCAE grade ≤ 1 with or without supplementation.

AST/ALT = aspartate aminotransferase/alanine aminotransferase, ULN = upper limit of normal

* Calculated by Cockcroft and Gault's formula: (140 – age [yr]) × body weight [Kg] × 1.23 × (0.85 if female) / serum creatinine [µmol/L].

14. Females of childbearing potential must have a negative serum pregnancy test during screening and be neither breast feeding nor intending to become pregnant during study participation. Females of childbearing potential must agree to avoid pregnancy during the study and agree to the use of 2 effective contraceptive methods (hormonal or barrier method of birth control, or abstinence) prior to study entry, for the duration of study

Approved v1.0

participation, and in the following 90 days after discontinuation of study treatment (see Section 6.8.1). Men with partner(s) of childbearing potential must take appropriate precautions to avoid fathering a child from screening until 28 days after discontinuation of study treatment and to use appropriate barrier contraception or abstinence.

Japan Sites Only (Phase 2 Dose Expansion): A female patient who is not of childbearing potential is one that (i.e., meets at least one of the following criteria):

- Has undergone a documented hysterectomy and/or bilateral oophorectomy,
- Has medically confirmed ovarian failure, or
- Has achieved postmenopausal status, defined as cessation of regular menses for at least 12 consecutive months with no alternative pathological or physiological cause (which may be confirmed with a serum follicle-stimulating hormone [FSH] level within the laboratory's reference range for postmenopausal women).

15. Capability to swallow capsules intact (without chewing, crushing, or opening).
16. Life expectancy ≥ 3 months.
17. Willingness and ability to comply with scheduled visits, treatment plan, laboratory tests, and other study procedures.

5.2 Exclusion Criteria

1. Concurrent participation in another therapeutic clinical trial.
2. Symptomatic brain metastases or leptomeningeal involvement.
3. History of previous cancer, except for squamous cell or basal-cell carcinoma of the skin, or any *in situ* carcinoma that has been completely resected, requiring therapy within the previous 2 years.
4. Incomplete recovery from any surgery prior to treatment.
5. Any of the following in the past 6 months: myocardial infarction, unstable angina, coronary/peripheral artery bypass graft, symptomatic congestive heart failure, cerebrovascular accident or transient ischemic attack, pulmonary embolism, deep vein thrombosis, symptomatic bradycardia, requirement for anti-arrhythmic medication.
6. Any of the following cardiac criteria:
    - Mean resting corrected QT interval (ECG interval measured from the onset of the QRS complex to the end of the T wave) for heart rate (QTc) > 470 msec obtained from 3 ECGs, using the screening clinic ECG machine-derived QTc value
    - Any clinically important abnormalities in rhythm, conduction or morphology of resting ECG (e.g., complete left bundle branch block, third degree heart block, second degree heart block, PR interval > 250 msec)
    - Any factors that increase the risk of QTc prolongation or risk of arrhythmic events such as heart failure, hypokalemia, congenital long QT syndrome, family history of long QT syndrome, or any concomitant medication known to prolong the QT interval

Approved v1.0

7. Known active infections (bacterial, fungal, viral including human immunodeficiency virus positivity).
8. Gastrointestinal disease (e.g., Crohn's disease, ulcerative colitis, short gut syndrome) or other malabsorption syndromes that would impact on drug absorption.
9. Peripheral neuropathy CTCAE grade ≥ 2.
10. History of extensive, disseminated, bilateral, or presence of CTCAE grade 3 or 4 interstitial fibrosis or interstitial lung disease including a history of pneumonitis, hypersensitivity pneumonitis, interstitial pneumonia, interstitial lung disease, obliterative bronchiolitis, and pulmonary fibrosis. Patients with history of prior radiation pneumonitis are not excluded.
11. Other severe acute or chronic medical or psychiatric condition or laboratory abnormality that may increase the risk associated with study participation or study drug administration, or that may interfere with the interpretation of study results and, in the judgment of the Investigator, would make the patient inappropriate for entry into this study, or could compromise protocol objectives in the opinion of the Investigator and/or TP Therapeutics.

Approved v1.0

6 STUDY TREATMENTS

6.1 TPX-0005 Drug Supply

6.1.1 Formulation and Packaging

TPX-0005 will be supplied for oral administration as 10-mg and 40-mg capsules in bottles containing 30 capsules of a single strength. Capsules have different sizes according to different strengths (10 mg = size 4; 40 mg = size 0). Study medication will be supplied by TP Therapeutics.

6.1.2 Preparation and Dispensing

Only qualified personnel who are familiar with procedures that minimize undue exposure of the study drug to them and to the environment should undertake the preparation, handling, and safe disposal of chemotherapeutic agents.

TPX-0005 will be provided in bottles containing 10-mg and 40-mg capsules, individually. Each bottle will contain 30 capsules enough medication for 28 days of dosing. Site personnel must ensure that patients clearly understand the directions for self-medication. Patients should be given sufficient supply to last until their next study visit. TPX-0005 will be dispensed at the beginning of each treatment cycle (or as otherwise indicated). Patients should be instructed to keep their medication in the bottles provided and not transfer it to any other container.

6.1.3 Administration

All patients will receive single-agent TPX-0005.

The starting dose for TPX-0005 in Phase 1 will be 40 mg. TPX-0005 will be administered once daily in 28-day cycles with the exception of Cycle 1, which is 21 days.

Patients in Phase 2 will receive TPX-0005 as a single agent once daily in 28-day cycles at the RP2D determined in the Phase 1.

Administration will be performed on an outpatient basis. TPX-0005 should be taken with at least 8 oz (240 mL) of water on an empty stomach (i.e., patient should refrain from food and beverages [except water] for at least 1 hour prior to dosing and for at least 2 hours after dosing). See Section 6.3 for dosing instructions in food effect cohort. Patients should be instructed to take their medication at approximately the same time each day and to not take more than the prescribed dose at anytime. However, a variance of up to 12 hours is allowed for any given dose, rather than miss a day's dose. If a patient misses a daily dose, they must be instructed not to "make it up" the next day. If a patient vomits at anytime after taking a dose, they must be instructed not to "make it up," but to resume subsequent doses the next day as prescribed. If a patient inadvertently takes 1 extra dose during a day, the patient should not take the next dose of TPX-0005. Patients should also be instructed to swallow the trial medication whole and not chew

Approved v1.0

the capsule prior to swallowing. No capsule should be ingested if it is broken, cracked, or otherwise not intact. Doses may be modified according to Table 6.

Patients may continue TPX-0005 treatment after objective progression of disease is determined if the patient is continuing to experience clinical benefit, in the opinion of the Investigator, and after discussion with TP Therapeutics.

The study Investigator may implement TPX-0005 dose suspension and/or reduction in order to ensure patient safety at any time in the course of the study (see Section 6.2).

At Day 1 of each cycle visit, and at the end of the treatment study, all unused or partially used bottles must be returned by patients to the Investigator and TP Therapeutics will provide instructions as to disposition of any unused TPX-0005. If TP Therapeutics authorizes destruction at the study site, the Investigator must ensure that the materials are destroyed in compliance with applicable environmental regulations, institutional policy, and any special instructions provided by TP Therapeutics. Destruction must be adequately documented.

6.1.4 Storage and Accountability

TPX-0005 capsules should be stored at 15°C to 30°C (59°F to 86°F). Medication should be kept in a secured locked area at the study site in accordance with applicable regulatory requirements. Patients should be instructed to keep their medication in its original container and stored at 15°C to 30°C (59°F to 86°F). Returned medication should be stored separately from medication that is yet to be dispensed.

Investigators and site staff are reminded to document storage room temperatures daily (i.e., manual or electronic systems that alert of any excursions) and ensure that thermometers are working correctly as required for proper storage of TPX-0005. Any temperature excursions should be reported to TP Therapeutics.

The investigational product (IP) must be stored as indicated. Deviations from the storage requirements, including any actions taken, must be documented and reported to TP Therapeutics. Once a deviation is identified, the IP must be quarantined and not used until TP Therapeutics provides documentation of permission to use the IP. The temperature of all locations where IP are stored should be monitored continuously and verified as appropriate per the site processes, preferably using a thermometer that measures minimum and maximum temperatures. Storage temperature should be recorded and monitored consistently by the site personnel. If a continuous measuring instrument/device is not available, daily temperature (and as applicable, humidity) recordings must be taken to ensure compliance with TP Therapeutics requirements and/or site standard operating procedures (SOPs), where applicable. If the site has its own SOPs, the most conservative standard will be applied, so that TP Therapeutics requirements are always met.

6.1.4.1 Temperature Excursions

If a temperature excursion occurs, the site should immediately contact their study monitor to alert them and escalate to TP Therapeutics for evaluation and disposition. Once a deviation is identified, the IP must be quarantined and not used until TP Therapeutics provides

Approved v1.0

documentation of permission to use the IP. When reporting an excursion, the following information, at a minimum, should be included:

- Protocol number
- Site number
- Length of time for the excursion
- Minimum/maximum temperatures for the excursion
- Impacted container numbers and/or lot numbers
- Confirmation of whether impacted containers were dispensed/administered to patients
- Copy of temperature monitoring log if available

The site should not use the supplies until a disposition is received from TP Therapeutics *via* the study management. The IP should continue to be stored in its appropriate location pending the disposition from TP Therapeutics. If the materials are rejected, TP Therapeutics will initiate a replacement shipment to the site.

6.2 Dose Modifications

6.2.1 Recommendation for Dose Modifications

Every effort should be made to administer the study treatment on the planned dose and schedule.

In the event of significant toxicity dosing may be withheld and/or reduced as described in the Table 8. In the event of multiple toxicities, dose modification should be based on the worst toxicity observed. Patients are to be instructed to notify Investigators at the first occurrence of any adverse symptom.

Dose modifications of an oral medication given continuously may occur in 2 ways:

- Within a cycle: dosing interruption until adequate recovery and dose reduction, if required, during a given treatment cycle; this may persist delaying the start of a new cycle.
- In the next cycle: dose reduction may be required in a subsequent cycle based on toxicity experienced in the previous cycle.

6.2.2 Dose Interruptions

Appropriate follow-up assessments should be done until adequate recovery occurs as assessed by the Investigator. Criteria required before treatment can resume are described in the dose modification table (see Table 8).

Doses may be held as needed until toxicity resolution. Depending on when the adverse event resolved, a treatment interruption may lead to the patient missing all subsequent planned doses within that same cycle or even to delay the initiation of the subsequent cycle.

Approved v1.0

If the adverse event that led to the treatment interruption recovers within the same cycle, then re-dosing in that cycle is allowed. Doses omitted for toxicity are not replaced within the same cycle. The need for a dose reduction at the time of treatment resumption should be based on the criteria defined in Table 8 unless expressly agreed otherwise following discussion between the Investigator and TP Therapeutics.

In the event of a treatment interruption for reasons other than treatment-related toxicity (e.g., elective surgery) lasting > 14 days, treatment resumption will be decided in consultation with TP Therapeutics.

Patients not recovering from TPX-0005-related toxicity within 28 days since the last dose should discontinue TPX-0005 treatment.

If a treatment interruption continues beyond Day 28 of the current cycle, then the day when treatment is restarted will be counted as Day 1 of the next cycle. Every effort should be made to maintain the tumor assessments scheduling as described in the Study Calendars (Section 8.4) (i.e., every 2 cycles [± 1 week] versus the date of Cycle 1 Day 1).

6.2.3 Dose Reductions

Following dosing interruption or cycle delay due to toxicity, the TPX-0005 dose may need to be reduced when treatment is resumed.

In cases where no specific dose adjustment requirements for CTCAE grade 2 treatment-related toxicity, Investigators should always manage their patients according to their medical judgment, which may include dose reduction or interruption based on the particular clinical circumstances in consultation with the Medical Monitor and TP Therapeutics.

Patients experiencing recurrent and intolerable CTCAE grade 2 toxicity may resume dosing at the next lower dose level once recovery to CTCAE grade ≤1 or baseline is achieved.

Dose reduction of TPX-0005 by 1 and, if needed, 2 dose levels (Table 8) will be allowed depending on the type and severity of toxicity encountered. Patients requiring more than 2 dose reductions will be discontinued from the treatment and entered into the follow-up phase, unless otherwise agreed between the Investigator and TP Therapeutics. All dose modifications/adjustments must be clearly documented in the patient's source notes and CRF.

Once a dose has been reduced for a given patient, all subsequent cycles should be administered at that dose level, unless further dose reduction is required. **Dose re-escalation is not allowed**.

Patients experiencing a DLT may resume dosing at the next lower dose level (if applicable) once adequate recovery is achieved.

Approved v1.0

6.2.4 Dose Modifications

Patients will be monitored closely for toxicity, and the dose of TPX-0005 may be adjusted as indicated in Table 8.

Table 8 Adverse Events Dose Modifications Table

Toxicity	CTCAE Grade 1**	CTCAE Grade 2**	CTCAE Grade 3	CTCAE Grade 4
Hematologic	Continue at same dose level	Continue at same dose level	Withhold dose until toxicity is grade ≤ 2, or has returned to baseline, then resume treatment at the same dose level or reduce by 1 dose level as per the Investigator's discretion<br><br>Grade 3 lymphopenia without other dose-limiting events (e.g., opportunistic infection) may continue study treatment without interruption	Withhold dose until toxicity is grade ≤ 2, or has returned to baseline, reduce the dose by 1 dose level and resume treatment<br><br>Grade 4 lymphopenia without other dose-limiting events (e.g., opportunistic infection) may continue study treatment without interruption
Non-Hematologic	Continue at same dose level	Continue at same dose level.<br><br>For prolonged or intolerable CNS toxicity, withhold dose until toxicity is grade ≤ 1 or has returned to baseline, then reduce by 1 dose level and resume treatment	Withhold dose until toxicity is grade ≤ 1 or has returned to baseline, then reduce by 1 dose level and resume treatment	Withhold dose until toxicity is grade ≤ 1 or has returned to baseline, then reduce by 1 dose level and resume treatment; or discontinue treatment as per the Investigator's discretion
Pneumonitis (in the absence of disease progression, pulmonary embolism, positive cultures or radiation effect)	Asymptomatic, radiographic findings only: No need for dose adjustment. Initiate appropriate monitoring.<br><br>Symptomatic: Withhold current dose until toxicity has returned to baseline. Rule out infection and consider initiating	Withhold current dose until toxicity has returned to baseline. Rule out infection and consider initiating treatment with corticosteroids. Then resume treatment at 1 dose level lower.<br><br>Discontinue permanently if pneumonitis recurs or if failure to	Discontinue treatment permanently	Discontinue treatment permanently

Toxicity	CTCAE Grade 1**	CTCAE Grade 2**	CTCAE Grade 3	CTCAE Grade 4
	treatment with corticosteroids. Then resume treatment at the same dose. Discontinue permanently if pneumonitis recurs or if failure to recover after 6 weeks of study treatment hold and steroid treatment.	recover after 6 weeks of study treatment hold and steroid treatment.		
Prolonged QTc	Assess electrolytes and concomitant medications<br><br>Correct any electrolyte abnormalities, or hypoxia<br><br>Continue at the same dose level	Assess electrolytes and concomitant medications<br><br>Correct any electrolyte abnormalities, or hypoxia<br><br>Continue at the same dose level	Withhold dose<br><br>Assess electrolytes and concomitant medications<br><br>Correct any electrolyte abnormalities, or hypoxia<br><br>Upon recovery to grade ≤ 1: if no other cause for QTc prolongation is found or is considered drug-related resume treatment at 1 dose level lower	Discontinue treatment permanently

**In cases where no specific dose adjustments for CTCAE grade 1 or grade 2 treatment-related toxicity are provided, Investigators should always manage their patients according to their medical judgment, which may include dose reduction or interruption based on the particular clinical circumstances.

6.3 Food Requirements

6.3.1 Non-Food Effect Cohort (Phase 1a and Phase 2)

Oral TPX-0005 will be administered QD with at least 8 oz (240 mL) of water on an empty stomach. No food or liquids other than water will be consumed for 1 hour before and 2 hours following each dose throughout the study. These fasting requirements may be removed (via a letter to the Investigators) if the data from the food effect sub-study indicate that there is no effect of food on the PK of TPX-0005.

6.3.2 Food Effect Cohort (Phase 1b)

A food effect sub-study will be conducted to evaluate the effect of a high-fat, high-calorie breakfast on TPX-0005 pharmacokinetics at the dose level where preliminary anti-tumor activity

Approved v1.0

(partial response) was observed by the Investigator. Each patient will serve as his/her own control.

For those patients scheduled to receive the "fed" treatment first, a test breakfast meal (described below) will be provided and must be consumed over 30 minutes on Cycle 0 Day -3. Study drug will be administered with approximately 8 oz (240 mL) of water 30 minutes after the start of the meal. No additional food will be allowed until at least 4 hours post-dose. Patients will then take TPX-0005 under the "fasted" condition (see below) on Cycle 1 Day 1.

For patients scheduled to receive the "fasted" treatment first, study drug will be administered with 8 oz (240 mL) of water on Cycle 0 Day -3. No food will be allowed for an additional 4 hours post-dose. For either treatment day, water will be allowed *ad libitum* except for 1 hour before and 1 hour after drug administration. Patients will then take TPX-0005 under the "fed" condition (see below) on Cycle 1 Day 1.

For all patients participating in the food effect sub-study, there will be no TPX-0005 doses administered after Cycle 0 Day -3 and before Cycle 1 Day 1. For all patients participating in the food effect sub-study, TPX-0005 will be administered following an overnight fast of at least 10 hours after Cycle 1 Day 1.

The test meal to be consumed will be a high-fat (approximately 50% of total caloric content of the meal) and high-calorie (approximately 800 to 1000 calories) meal. This test meal should derive approximately 150, 250, and 500-600 calories from protein, carbohydrate, and fat, respectively. An example test meal would be 2 eggs fried in butter, 2 strips of bacon (may be replaced with ham and cheese of similar caloric content), 2 slices of toast with butter, 4 oz of hash brown potatoes, and 8 oz of whole-fat milk. Substitutions to this test meal can be made after discussion with TP Therapeutics, as long as the meal provides a similar amount of calories from protein, carbohydrate, and fat and has comparable meal volume and viscosity (if substitutions are made, the contents of the meal will be documented by a dietitian or designee to confirm it matches the FDA requirements for protein, carbohydrate, and fat described above). However, it is understood that some patients may not be able to consume the entire meal. Study staff should record the percent of the test meal breakfast and the time it takes to be consumed.

6.4 Concomitant Medications

Drug-interaction studies with TPX-0005 have not been conducted. All concomitant medications, blood products, as well as non-drug interventions (e.g., paracentesis) received by patients from screening until the end-of-treatment visit will be recorded on the CRF. A list of concomitant medications is to be provided to TP Therapeutics or their designee prior to study enrollment.

6.4.1 Cytochrome P450 Substrates

A study with recombinant human cytochrome P450 isoforms (CYP450) suggests that TPX-0005 is metabolized mainly by CYP3A4 isoform. *In vitro* studies indicated that TPX-0005 does not inhibit or induce the activity of CYP3A4. Please refer to IB for detailed description.

Approved v1.0

- Inhibition of CYP3A4 isoenzymes may increase TPX-0005 exposure leading to a potential increase in toxicities. The use of known strong/moderate inhibitors are strongly discouraged and only used if deemed in the patients' best interest, by the treating Investigator, from 10 days prior to the first dose of TPX-0005 until study treatment discontinuation.
  - Strong CYP3A4 inhibitors: grapefruit juice or grapefruit/grapefruit-related citrus fruits (e.g., Seville oranges, pomelos), ketoconazole, miconazole, itraconazole, voriconazole, posaconazole, clarithromycin, telithromycin, indinavir, saquinavir, ritonavir, nelfinavir, amprenavir, fosamprenavir, nefazodone, lopinavir, troleandomycin, mibefradil, conivaptan
  - Moderate CYP3A4 inhibitors: erythromycin, verapamil, atazanavir, fluconazole, darunavir, diltiazem, delavirdine, aprepitant, imatinib, tofisopam, ciprofloxacin, cimetidine.
- TPX-0005 metabolism may be induced when taking strong CYP3A4 inducers resulting in reduced plasma concentrations. Therefore, coadministration of TPX-0005 in combination with those listed below and other strong CYP3A4 inducers is strongly discouraged from 10 days prior to the first dose of TPX-0005 until study treatment discontinuation.
  - Strong CYP34A inducers: phenobarbital, rifampin, phenytoin, carbamazepine, rifabutin, rifapentin, St. John's Wort.

6.4.2 Other Anti-tumor or Investigational Drugs

No additional systemic anti-tumor therapy will be permitted while patients are receiving study therapy. Additionally, the concurrent use of select herbal supplements is not permitted.

Bisphosphonate therapy for metastatic bone disease is permitted. Bisphosphonate therapy should be given as per local medical practice.

6.4.3 Seizure Prophylaxis

Moderate inducers of CYP3A, CYP3A4, or CYP3A4/5, such as dexamethasone or other glucocorticoids, may be used at the discretion of the Investigator.

Seizure prophylaxis with non–enzyme-inducing anti-epileptic drugs (non-EIAEDs) is allowed during the study for patients with controlled asymptomatic CNS involvement. EIAEDs are not allowed. Table 9 lists EIAEDs and non-EIAEDs.

Approved v1.0

Table 9 List of EIAEDs and non-EIAEDs

EIAEDs	non-EIAEDs
Carbamazepine<br>Oxcarbazepine<br>Phenytoin<br>Fosphenytoin<br>Phenobarbital<br>Primidone	Levetiracetam<br>Valproic acid<br>Lacosamide<br>Gabapentin<br>Topiramate<br>Lamotrigine<br>Tiagabine<br>Zonisamide<br>Clonazepam<br>Clonozam<br>Pregabalin

6.4.4 Hematopoietic Growth Factors

Primary prophylactic use of granulocyte-colony stimulating factors is not permitted during the first cycle but they may be used to treat treatment-emergent neutropenia as indicated by the current American Society of Clinical Oncology (ASCO) guidelines.

Erythropoietin may be used at the Investigator's discretion for the supportive treatment of anemia.

6.4.5 Anti-diarrheal and Anti-emetic Therapy

Primary prophylaxis of diarrhea, nausea, and vomiting is not permitted in the first cycle in both Phase 1 and Phase 2 portions of the study.

Primary prophylaxis in subsequent cycles is at the Investigator's discretion. The choice of the prophylactic drug is up to the Investigator with TP Therapeutics approval and assuming there is no known or expected drug-drug interaction. If so it must be approved by TP Therapeutics.

6.4.6 Anti-inflammatory Therapy

Anti-inflammatory or narcotic analgesic may be offered as needed assuming there is no known or expected drug-drug interaction.

6.4.7 Cutaneous Toxicity Therapy

Prompt medical intervention is recommended at the first sign of appearance of cutaneous toxicity including topical or oral corticosteroids if required according to Investigator's judgment, assuming there is no known or expected drug-drug interaction.

Approved v1.0

6.4.8 Testosterone Replacement

Testosterone replacement therapy is only allowed in the presence of signs and symptoms clearly attributable to hypogonadism in consultation with an endocrinologist, who should also exclude any potential confounding effects of elevated prolactin and/or estradiol, or a significant recent change in corticosteroid dose, before doing so.

6.5 Surgery

Caution is advised on theoretical grounds for any surgical procedures during the study. The appropriate interval of time passed between surgery and TPX-0005 required to minimize the risk of impaired wound healing and bleeding has not been determined. Stopping TPX-0005 is recommended for at least 2 days prior to surgery. Postoperatively, the decision to reinitiate TPX-0005 treatment should be based on a clinical assessment of satisfactory wound healing and recovery from surgery.

6.6 Palliative Radiation

Palliative radiotherapy on study is permitted for the treatment of painful bony lesions providing the lesions were known at the time of study entry and the Investigator clearly indicates that the need for palliative radiotherapy is not indicative of disease progression. In view of the current lack of data about the interaction of TPX-0005 with radiotherapy, TPX-0005 treatment should be interrupted during palliative radiotherapy, stopping 1 day before palliative radiotherapy and resuming treatment 1 day after completion of palliative radiotherapy and recovery from any acute radiation toxicities to baseline.

6.7 Supportive Care

Palliative and supportive care for disease-related symptoms may be administered at the Investigator's discretion and according to available ASCO guidelines.

6.8 Life Style Guidelines

6.8.1 Contraception

In this study, patients of childbearing potential will receive TPX-0005, a compound for which the teratogenic risk is currently unknown. Two methods of highly effective contraception must be used throughout the study and continued for 90 days after the last dose. The Investigator, in consultation with the patient and his/her partner(s), will select 2 appropriate methods of contraception for the individual patient from the permitted list of contraception methods, and instruct the patient in their consistent and correct use. The Investigator, at each study visit, will discuss with the patient the need to use highly effective contraception consistently and correctly and document such conversation in the patient chart. In addition, the Investigator will instruct the

patient to call immediately if a selected birth control method is discontinued or if pregnancy is known or suspected.

Highly effective methods of contraception are those that, alone or in combination, result in a failure rate of less than 1% per year when used consistently and correctly (i.e., perfect use) and include the following:

1. Established use of oral, inserted, injected, or implanted hormonal methods of contraception are allowed provided the patient remains on the same treatment throughout the entire study and has been using that hormonal contraceptive for an adequate period of time to ensure effectiveness.
2. Correctly placed copper containing intrauterine device.
3. Male condom or female condom used WITH a spermicide (i.e., foam, gel, film, cream, suppository).
4. Male sterilization with appropriately confirmed absence of sperm in the post-vasectomy ejaculate.
5. Bilateral tubal ligation or bilateral salpingectomy.

6.8.2 Sunlight Exposure

Patients will be advised to report any reaction to sun-exposed skin. In addition, special precautions will be taken to limit any potential photo irritation effect, by minimizing the patients' exposure to light including high intensity UVB sources such as tanning beds, tanning booths, and sunlamps. Patients should be advised to apply sunscreen and wear appropriate clothing to minimize exposure to the sun during treatment with TPX-0005.

6.8.3 Additional Lifestyle Guidance

Patients will be advised to avoid eating or drinking grapefruit/grapefruit-related citrus fruits (e.g., Seville oranges, pomelos).

Approved v1.0

7 STUDY PROCEDURES

7.1 Clinical Assessments

The schedule of assessments representing the required testing procedures to be performed during the study is diagrammed in the Study Calendars (Section 8.4).

Prior to conducting any study-related activities, written informed consent and any other authorizations must be signed and dated by the patient or patient's legal representative. Every effort should be made to ensure that the protocol-required tests and procedures are completed as described. However, it is anticipated that from time to time there may be circumstances outside of the control of the Investigator that may make it unfeasible to perform the test. In those cases, the Investigator should take all steps necessary to ensure the safety and wellbeing of the patient. When a protocol-required test cannot be performed, the Investigator will document the reason for this and any corrective and preventive actions that he/she has taken to ensure that normal processes are adhered to as soon as possible.

The TP Therapeutics study team or their designee should be informed of these incidents in a timely fashion. Unless indicated otherwise, scheduled clinic visits should occur within ± 2 days of the specified dates; where this is not possible because of extenuating circumstances (e.g., holidays), the visit should take place as close to this window as possible.

7.1.1 Demographics

Demographic information (e.g., date of birth and race) will be recorded at screening, as allowed per local country privacy law regulations.

7.1.2 Medical History

Relevant medical history, including history of current disease, other pertinent clinical conditions, and information regarding underlying diseases will be recorded at screening.

7.1.3 Physical Examination

A complete physical examination will be performed by either the Investigator or a sub-Investigator according to the Study Calendar. New abnormal physical examination findings must be documented and followed by the Investigator, sub-Investigator, or other qualified staff at the next scheduled visit or earlier as clinically indicated.

7.1.4 Vital Signs

Body temperature, blood pressure, heart rate, respiratory rate, and pain level (0-10) will be performed at Screening and at every clinic visit. Blood pressure and heart rate can be assessed either in the supine or seated position. Height will be recorded at screening only. Body weight will be recorded at every clinic visit.

Approved v1.0

7.1.5 Performance Status

The ECOG PS scale will be assessed at Screening and at every clinic visit (Study Calendars).

7.1.6 Adverse Events

Assessment of adverse events will include type, incidence, severity (graded by the NCI CTCAE, v4.03), timing, seriousness, and relatedness. Adverse events will be assessed at every clinic visit.

7.1.7 Concomitant Medications and Treatments

All concomitant medication and concurrent treatments (e.g., radiation therapy) will be documented at Screening and at every clinic visit. The indication for administration and dates of medication or treatment will be captured.

7.2 Tumor Assessments

Tumor assessments will be performed at Screening, at the end of Cycle 2 (7 weeks from the initial TPX-0005 treatment ± 2 days), every 2 cycles (± 7 days) up to Cycle 18 and then every 3 cycles (± 7 days) up to Cycle 36 and then every 4 cycles (± 7 days) thereafter until documented progression of disease regardless of treatment delays resulting from toxicity, and at the End of Treatment (EOT) if more than 4 weeks have passed since the last imaging assessment). Care must be taken in scheduling disease assessments to prevent introduction of bias based on-treatment delays.

Imaging studies will include a computerized tomography (CT) or magnetic resonance imaging (MRI) scan of the brain, chest, abdomen, pelvis (depending on tumor type), plus bone scan (if applicable).

- At Screening, a CT/MRI of the brain should be obtained to rule out newly diagnosed, untreated brain metastases or to document stability of previously treated brain metastases. Regardless of whether brain metastases are documented at Screening or not, CT/MRI brain scans should be performed at every on-study assessment.
- For patients diagnosed with primary brain tumors, only the CT/MRI of the brain will be required at Screening and during the study.
- At Screening, a bone scan should be obtained if bone metastases are suspected. For patients with bone metastases at baseline, a bone scan should be performed every 3 cycles.

Radiographic confirmation of objective tumor response or disease progression will be based on RECIST version 1.1 and assessed both locally and by blinded independent central review (BICR). The same imaging modality should be used at Screening and on study.

IV contrast is required when not medically contraindicated. Patients who have a contraindication to IV contrast may have MRI exams of the brain, abdomen, and pelvis performed in lieu of CTs and a non-contrast CT of the chest. Positron emission tomography (PET)/computerized

tomography (CT) may be used to document baseline and new disease, but the CT portion of a PET/CT may not be used in lieu of a diagnostic CT, unless it is performed with IV contrast.

For patients with brain metastases, radiographic confirmation of intracranial objective tumor response or disease progression will be based on RECIST version 1.1 and assessed both locally and by BICR.

Additional requirements are provided in the Imaging Manual.

7.3 Tumor Procurement

7.3.1 Archival Tumor Tissue

These samples are mandatory for all patients enrolled in the study. (FFPE tissue block[s]) from initial diagnosis that contain sufficient tissue to generate at least 10 (preferably 15) unbaked, 5-micron thick unstained slides will be collected. If no FFPE block is available, then at least 10 (preferably 15) unbaked, 5-micron thick unstained slides containing FFPE tumor tissue must be provided. The archived tumor tissue may be obtained and analyzed outside the 28-day screening window. In cases where archived tumor tissue is not available, a *de novo* biopsy should be obtained for this purpose.

Samples will be sent to the TP Therapeutics-designated central laboratories for determination of the presence of *ALK, ROS1, NTRK1, NTRK2,* or *NTRK3* rearrangement. Additionally, samples will be used for assessment of biomarkers potentially associated with sensitivity and/or resistance to TPX-0005 (mutations in *ALK, ROS1, NTRK1, NTRK2,* or *NTRK3*, mutations, mutations/copy number variation of various genes, expression and/or phosphorylation of various proteins, etc.). Tissue samples from all patients will be used for additional biomarker analyses. Details for handling of these samples including processing, storage, and shipment will be provided in the Laboratory Manual.

7.3.2 *De Novo* Tumor Biopsy

These samples will be mandatory for all patients enrolled in the Phase 1 Dose Escalation and Food Effect cohorts and Phase 2 if archival tumor tissue is not available. This *de novo* biopsy must be taken no more than 28 days prior to starting study treatment. The *de novo* biopsy will consist of an incisional or excisional biopsy, or a core needle biopsy, of a primary or metastatic lesion. Pleural effusion cell pellets may substitute for a tumor core biopsy, as appropriate. Fine needle aspiration biopsies are not acceptable. The tumor tissue will be processed as specified in the Laboratory Manual.

Additional, optional *de novo* tumor specimen collection at screening and at the time of progression is encouraged. If present, pleural effusion cell pellets may substitute for tumor core biopsy. As noted above, a *de novo* biopsy should be obtained in cases where archived tumor tissue is not available.

Approved v1.0

7.3.3 Liquid Biopsies

In all patients, 20 mL of blood will be collected at the screening period and at the time of progression OR at the end of study (EOS) to assess ccfDNA (circulating cell-free DNA). ccfDNA will undergo NGS in order to identify relevant molecular alterations that may predict activity of TPX-0005 and to gain insights into potential mechanisms of resistance such as mutational status of the *ALK*, *ROS1, NTRK1, NTRK2*, and *NTRK3* genes among others.

7.4 Blinded Independent Central Review (BICR)

All scans will be submitted to a third-party core imaging laboratory for independent review of tumor response and disease progression during the study according to an Imaging Review Charter to be prepared by the core imaging laboratory in consultation with TP Therapeutics.

It is important to the integrity of the study that all imaging studies are forwarded to the core imaging laboratory in a timely manner throughout the study, preferably within 1 week of collection. Further details can be found in the Imaging Manual.

7.5 Clinical Laboratory Assessments

Appendix 1 and Appendix 2 list all of the specific laboratory tests that should be performed for the Phase 1 and Phase 2 portions of the study. Table 15, Table 16, and Table 17 are Study Calendars that list all timing of the laboratory tests to be performed.

Investigators can also perform additional local laboratory tests for the purpose of planning treatment administration, dose modification, or following adverse events.

7.6 Electrocardiogram (ECG)

Triplicate 12-lead (with a 10-second rhythm strip) tracing will be used for all ECGs. It is preferable that the machine used has a capacity to calculate the standard intervals automatically. At each time point (see Study Calendars, Section 8.4), 3 consecutive ECGs will be performed at approximately 2 minutes apart to determine the mean QTc interval. If the mean QTc is prolonged (> 500 msec [i.e., CTCAE grade 3]), then the ECGs should be re-evaluated by a qualified person at the site for confirmation as soon as the finding is made, including verification that the machine reading is accurate. If manual reading verifies a QTc of > 500 msec, immediate correction for reversible causes (including electrolyte abnormalities, hypoxia, and concomitant medications for drugs with the potential to prolong the QTc interval) should be performed. Study drug will be held until the QTc interval decreases to ≤ 500 msec. Patients will then re-start the study drug at the next lowest dose level. If the QTc interval has still not decreased to < 500 msec after 2 weeks, or if at any time a patient has a QTc interval > 515 msec or becomes symptomatic, the patient will be removed from the study. Additional triplicate ECGs may be performed as clinically indicated.

Prior to concluding that an episode of prolongation of the QTc interval is due to study drug, thorough consideration should be given to potential precipitating factors (e.g., change in-patient

Approved v1.0

clinical condition, effect of concurrent medication, electrolyte disturbance) and possible evaluation by specialist. If patient experiences a cardiac or neurologic AE (specifically syncope, dizziness, seizures, or stroke), an ECG (triplicate) should be obtained at the time of the event.

When matched with PK sampling, the ECG must be carried out before each PK sample drawing such that the PK sample is collected at the nominal time (i.e., the timing of the PK collections over rides the timing of the ECG collections).

7.7 Echocardiogram

In order to monitor potential left ventricular ejection fraction dysfunction, an echocardiogram will be performed at the screening period and repeated every 3 cycles thereafter.

7.8 Pharmacokinetic (PK) Assessments

Blood samples for PK analysis of TPX-0005 will be collected during the study from patients receiving study medication. Table 10 lists noncompartmental PK parameters that will be calculated from the individual plasma concentration-versus-time profiles [redacted]. The individual and mean plasma concentration-versus-time profiles will be displayed graphically. In addition, a population PK model may be developed to characterize inter-patient variability and covariate effect by pooling all available data using [redacted].

Blood sampling schedules for PK assessment can be found in Table 11, Table 12, Table 13, Table 14 and in Study Calendars (Table 15, Table 16, and Table 17) for dose escalation and dose expansion, respectively.

Table 10 Noncompartmental Pharmacokinetic Parameters

AUC_{last}	The area under the curve (AUC) from time zero to the last quantifiable concentration point (t_{last})
AUC_{inf}	The AUC from time zero to infinity
AUC_{tau}	The AUC calculated to the end of the dosing interval, tau
AUC%Extrap	The percent extrapolated area under the curve to infinity. This is calculated as ratio of the extrapolated area (C_{last}/λ_z) to the total AUC_{inf}
C_{min}	Observed concentration at the end of a dosing interval (taken directly before next administration)
C_{max}	The maximum observed plasma, blood, serum, or other body fluid drug concentration
T_{max}	The time to reach maximum (peak) plasma, blood, serum, or other body fluid drug concentration
λ_z	Smallest (slowest) disposition (hybrid) rate constant (time-1) may also be used for terminal elimination rate constant
$t_{½}$	The elimination half-life associated with the terminal slope (λ_z) of a semi logarithmic concentration-time curve (time).
$t_{½,acc}$	Effective half-life can be calculated from R_{acc}
CL/F	Apparent total body clearance of drug from the plasma
V_z/F	The apparent volume of distribution during terminal phase (associated with λ_z)
Racc	Accumulation ratio calculated using AUC_{tau} values obtained from a dosing interval at steady-state divided by AUC_{tau} at Day 1 or PK lead-in phase

If the clinical safety, PK, and other data become available and indicate that an alternative dosing schedule such as BID is deemed necessary, dose escalation may then proceed with an alternative dosing schedule.

The collection of plasma with exact dates and clock times of drug administration and sample collection will be recorded on the appropriate CRF.

All blood samples will be taken by either direct venipuncture or venous catheter or indwelling cannula inserted in a forearm vein. Samples should be processed and labeled as detailed in the Laboratory Manual. Plasma fractions will be split in 2 aliquots and all samples should be stored frozen at~ -70°C within 90 minutes of venipuncture at the site until sample shipment.

7.8.1 PK Collection Times Points

PK blood samples will be collected in both Phase 1a and 1b (Table 11, Table 12 and Table 13) and Phase 2 (Table 14).

7.8.1.1 Blood for PK Analysis of TPX-0005 (Phase 1a Dose Escalation)

For the determination of TPX-0005 during the Phase 1a Dose Escalation portion, 5.0 mL of whole blood will be drawn for each time point. For patients treated in the escalation phase (Table 11), 2 full PK profiles will be collected during the PK lead-in period (Cycle 1 Day -3) and Cycle 1 Day 15 and abbreviated PK profiles on Day 1 of Cycles 2-4. There is an additional pre-dose PK draw on Cycle 1 Day 8. A total of approximately 120 mL of blood will be collected for each patient during the Phase 1a Dose Escalation phase.

Approved v1.0

Table 11 Time Points of Blood Collection for TPX-0005 PK Assessment during Phase 1a Dose Escalation Phase

PK Collection Number	Sample Number	Cycle	Day	Scheduled Time Points Relative to Previous Dose (hours)	Description
1	1	PK lead-in	-3	0 hr[a]	Pre-dose
1	2	PK lead-in	-3	1 hr	Post-dose
1	3	PK lead-in	-3	2 hr	Post-dose
1	4	PK lead-in	-3	4 hr	Post-dose
1	5	PK lead-in	-3	6 hr	Post-dose
1	6	PK lead-in	-3	8 hr	Post-dose
1	7	PK lead-in	-2	24 hr	Post-dose
1	8	PK lead-in	-1	48 hr	Post-dose
2	9	1	1	72 hr	72 hr post-dose of PK lead-in and Cycle 1 Day 1 pre-dose
2	10	1	1	4 hr	Post-dose
3	11	1	8	0 hr[a]	Pre-dose
4	12	1	15	0 hr[a]	Pre-dose
4	13	1	15	1 hr	Post-dose
4	14	1	15	2 hr	Post-dose
4	15	1	15	4 hr	Post-dose
4	16	1	15	6 hr	Post-dose
4	17	1	15	8 hr	Post-dose
4	18	1	16	24 hr	Pre-dose
5	19	2	1	0 hr[a]	Pre-dose
5	20	2	1	4 hr	Post-dose
6	21	3	1	0 hr[a]	Pre-dose
6	22	3	1	4 hr	Post-dose
7	23	4	1	0 hr[a]	Pre-dose
7	24	4	1	4 hr	Post-dose
	1001+ [b]	NA	NA	Unscheduled[b]	Unspecified

All measurement times are relative to dose of TPX-0005 unless otherwise specified

[a] Take PK sample immediately prior to the administration of TPX-0005

[b] Sample numbers for any unscheduled blood collection for TPX-0005 will start with 1001

7.8.1.2 Blood for PK Analysis of TPX-0005 (Phase 1b Food Effect)

The Phase 1b Food Effect will perform according to Section 3.3. Each patient will serve as his/her own control.

For the determination of TPX-0005 during the Phase 1b Dose Escalation portion, 5.0 mL of whole blood will be drawn for each time point. For patients treated in the Food Effect phase, 2 full PK profiles will be collected during the PK lead-in period and Cycle 1 Day 1 and abbreviated PK profiles on Cycle 1 Day 15 and on Day 1 of Cycles 2-4. There is an additional pre-dose PK draw on Cycle 1 Day 8. A total of approximately 115 mL blood will be collected for each patient during Phase 1b Dose Escalation phase (Table 12 and Table 13)

Table 12 Time Points of Blood Collection for TPX-0005 PK Assessment during Phase 1b Food Effect Study (FED Followed by FASTED)

PK Collection Number	Sample Number	Cycle	Day	Scheduled Time Points Relative to Previous Dose (hours)	Description
10	1	PK lead-in	-3	0 hr[a]	Pre-dose (Fed)
10	2	PK lead-in	-3	1 hr	Post-dose (Fed)
10	3	PK lead-in	-3	2 hr	Post-dose (Fed)
10	4	PK lead-in	-3	4 hr	Post-dose (Fed)
10	5	PK lead-in	-3	6 hr	Post-dose (Fed)
10	6	PK lead-in	-3	8 hr	Post-dose (Fed)
20	7	PK lead-in	-2	24 hr	Post-dose (Fed)
30	8	1	1	0 hr[a]	Pre-dose (Fasted)
30	9	1	1	1 hr	Post-dose (Fasted)
30	10	1	1	2 hr	Post-dose (Fasted)
30	11	1	1	4 hr	Post-dose (Fasted)
30	12	1	1	6 hr	Post-dose (Fasted)
30	13	1	1	8 hr	Post-dose (Fasted)
40	14	1	2	24 hr	Cycle 1 Day 1 post-dose (Fasted)
50	15	1	8	0 hr[a]	Pre-dose
60	16	1	15	0 hr[a]	Pre-dose
60	17	1	15	4 hr	Post-dose
70	18	2	1	0 hr[a]	Pre-dose
70	19	2	1	4 hr	Post-dose
80	20	3	1	0 hr[a]	Pre-dose
80	21	3	1	4 hr	Post-dose
90	22	4	1	0 hr[a]	Pre-dose
90	23	4	1	4 hr	Post-dose
	1001+ [b]	NA	NA	Unscheduled[b]	Unspecified

All measurement times are relative to dose of TPX-0005 unless otherwise specified
[a] Take PK sample immediately prior to the administration of TPX-0005
[b] Sample numbers for any unscheduled blood collection for TPX-0005 will start with 1001

Approved v1.0

Table 13 Time Points of Blood Collection for TPX-0005 PK Assessment during Phase 1b Food Effect study (FASTED Followed by FED)

PK Collection Number	Sample Number	Cycle	Day	Scheduled Time Points Relative to Previous Dose (hours)	Description
10	1	PK lead-in	-3	0 hr[a]	Pre-dose (Fasted)
10	2	PK lead-in	-3	1 hr	Post-dose ((Fasted)
10	3	PK lead-in	-3	2 hr	Post-dose (Fasted)
10	4	PK lead-in	-3	4 hr	Post-dose (Fasted)
10	5	PK lead-in	-3	6 hr	Post-dose (Fasted)
10	6	PK lead-in	-3	8 hr	Post-dose (Fasted)
20	7	PK lead-in	-2	24 hr	Post-dose (Fasted)
30	8	1	1	0 hr[a]	Pre-dose (Fed)
30	9	1	1	1 hr	Post-dose (Fed)
30	10	1	1	2 hr	Post-dose (Fed)
30	11	1	1	4 hr	Post-dose (Fed)
30	12	1	1	6 hr	Post-dose (Fed)
30	13	1	1	8 hr	Post-dose (Fed)
40	14	1	2	24 hr	Cycle 1 Day 1 post-dose (Fed)
50	15	1	8	0 hr[a]	Pre-dose
60	16	1	15	0 hr[a]	Pre-dose
60	17	1	15	4 hr	Post-dose
70	18	2	1	0 hr[a]	Pre-dose
70	19	2	1	4 hr	Post-dose
80	20	3	1	0 hr[a]	Pre-dose
80	21	3	1	4 hr	Post-dose
90	22	4	1	0 hr[a]	Pre-dose
90	23	4	1	4 hr	Post-dose
	1001+ [b]	NA	NA	Unscheduled[b]	Unspecified

All measurement times are relative to dose of TPX-0005 unless otherwise specified
[a] Take PK sample immediately prior to the administration of TPX-0005
[b] Sample numbers for any unscheduled blood collection for TPX-0005 will start with 1001

7.8.1.3 Blood for PK Analysis of TPX-0005 (Phase 2)

For patients enrolled in the Phase 2 Dose Expansion phase, 5 abbreviated PK profiles will be collected on Cycle 1 Day 1, Cycle 1 Day 15, and Cycles 2-4 Day 1 (Table 14). At each time point, 5.0 mL of whole blood will be drawn. A total of approximately 50 mL blood will be collected for each patient enrolled in the dose expansion phase.

Table 14 Time Points of Blood Collection of TPX-0005 PK Assessment during Phase 2 Expansion Cohort

PK Collection Number	Sample Number	Cycle	Day	Scheduled Time Points Relative to Previous Dose (hours)	Description
101	201	1	1	0 hr[a]	Pre-dose
101	202	1	1	4 hr	Post-dose
102	203	1	15	0 hr[a]	Pre-dose
102	204	1	15	4 hr	Post-dose
103	205	2	1	0 hr[a]	Pre-dose
103	206	2	1	4 hr	Post-dose
104	207	3	1	0 hr[a]	Pre-dose
104	208	3	1	4 hr	Post-dose
105	209	4	1	0 hr[a]	Pre-dose
105	210	4	1	4 hr	Post-dose
	2001+[b]	NA	NA	Unscheduled[b]	Unspecified

All measurement times are relative to dose of TPX-0005 unless otherwise specified
[a] Take PK sample immediately prior to the administration of TPX-0005
[b] Sample numbers for any unscheduled blood collection for TPX-0005 will start with 2001

PK sampling schedule may be modified based on emerging PK data. In addition to samples collected at the scheduled times, an additional blood sample should be collected from patients experiencing unexpected and/or serious AEs and the date and time documented in the CRF.

All efforts will be made to obtain the PK samples at the scheduled nominal time relative to dosing. However, samples obtained within 10% of the nominal time (e.g., within 6 minutes of a 60-minute sample, within 12 minutes of a 120-minute samples) will be considered protocol compliant, and the exact time of the sample collection noted on the CRF. If a scheduled blood sample collection cannot be completed for any reason, the missed sample time may be re-scheduled with agreement of clinical Investigators, patient, and TP Therapeutics.

PK samples will be assayed for TPX-0005 using a validated analytical method in compliance with TP Therapeutics standard operating procedures. Details regarding the collection, processing, storage, and shipping of the blood samples will be provided in the Study Manual.

7.8.1.4 CSF for Analysis of TPX-0005 Concentration

If a patient is required to undergo a lumber puncture while on trial for disease-related issues, an additional ~1 mL sample of CSF should be collected for determining TPX-0005 concentrations any time at steady state. If this sample is collected, a plasma PK sample should also be collected at approximately the same time. Detailed collection procedures will be provided in the laboratory manual.

It is recommended that patients do not undergo lumbar puncture solely for the purpose of analysis of the TPX-0005 concentration in the CSF.

Approved v1.0

7.9 Pharmacodynamic (PD) Assessment

7.9.1 *De Novo* Tumor Biopsy

One optional fresh tumor tissue re-biopsy is recommended between Cycle 1 Day 8 and Cycle 1 Day 15 during the Phase 1b Food Effect and Phase 2 Dose Expansion to assess the PD effect of TPX-0005. The biopsy should obtain enough amount of tumor to generate at least 10 (preferably 15) unbaked, 5-micron thick unstained slides containing FFPE tumor tissue. Specimens will be sent to the TP Therapeutics-designated central laboratories for PD study. Patients enrolled in the Phase 1a Dose Escalation part of the study will not undergo this optional fresh tumor tissue re-biopsy.

7.10 Patient-Reported Outcomes (PRO)

For NSCLC patients enrolled into Phase 2 Dose Expansion, at screening, prior to the first dose of TPX-0005 on Cycle 1 Day 1, pre-dose on Day 1 of each subsequent treatment cycle thereafter, and at the End of Treatment, patients will be required to complete the following self-administered quality of life (QOL) instruments: the European Organization for Research and Treatment of Cancer (EORTC) Core Quality of Life Questionnaire (QLQ-C30) (Appendix 5) and EORTC-Lung Cancer Quality of Life Questionnaire (QLQ-LC13) (Appendix 6) to assess health status.

Non-NSCLC patients will only be required to complete the EORTC-QLQ-C30 module (Appendix 5).

Approved v1.0

8 STUDY ASSESSMENTS BY VISIT

8.1 Phase 1a Dose Escalation

8.1.1 Screening Phase 1a Dose Escalation (within 28 days of the first dose of TPX-0005)

- Obtain Informed Consent
- Eligibility checklist (see complete list in Sections 5.1 and 5.2)
- Perform molecular testing as per Section 5.1 and Table 15
  - ❖ Molecular testing to determine eligibility can be performed in advance with no time limit (e.g., the patient can be tested while being treated on another anticancer therapy)
  - ❖ Source documents (molecular pathology report detailing the specific test that detect the *ALK*, *ROS1*, or *NTRK* rearrangement, or *ALK* amplification or mutations in neuroblastoma) should be submitted for TP Therapeutics approval of eligibility
  - ❖ Submitted archival tissues to TP Therapeutics's designated central laboratory to confirm the specific molecular alteration
  - ❖ An optional tumor tissue re-biopsy (for patients who have failed prior TKIs) during the screening period to investigate resistance mechanism(s) is recommended but not mandatory
- Obtain detailed treatment history
  - ❖ Number of prior TKIs
  - ❖ Duration of treatment of each prior TKI
  - ❖ Investigator assessment of best response to each prior TKI
  - ❖ Number of prior chemotherapy regimens
  - ❖ Duration of treatment of each chemotherapy regimen
  - ❖ Investigator assessment of best response to each prior chemotherapy regimen
  - ❖ Number of prior immunotherapy regimens
  - ❖ Duration of treatment of each immunotherapy regimen
  - ❖ Investigator assessment of best response to each prior immunotherapy regimen
  - ❖ For patients with prior treated brain metastases, record the method of radiation: whole brain radiation, stereotactic radiosurgery, or both. Record also the dates of the radiation treatment(s), total dose delivered to each course of treatment, and date of last radiation to the start of TPX-0005 treatment
- Obtain detailed medical history
- Perform a complete physical examination

Approved v1.0

- Perform and record vital signs, height, body weight, pain level (0-10), and ECOG PS
- Review concomitant medications
- **Female patients with childbearing potential:** Perform a serum pregnancy test
- Collect blood and urine for clinical laboratory assessments and tumor markers
- Collect blood for ccfDNA
- Obtain echocardiogram for baseline LVEF assessment
- Perform and record triplicate ECGs:
  - ❖ **All** patients: Triplicate 12-lead ECGs obtain approximately 2 minutes apart
- Obtain cardiac troponin-I test
- Obtain baseline tumor assessment including CT chest, abdomen, and pelvis, MRI of the brain and bone scan
- Patient registration

8.1.2 Lead-in PK Cycle 0 Day -3 (Phase 1a Dose Escalation)

- Perform a complete physical examination
- Perform and record vital signs, body weight, pain level (0-10), and ECOG PS
- Review medical history
- Review concomitant medications
- **Female patients with childbearing potential:** Perform a serum pregnancy test
- Collect blood and urine for clinical laboratory assessments
- **All patients**: Collect a baseline pre-dose PK sample
- Perform and record pre-dose ECGs:
  - ❖ **All** patients: Triplicate 12-lead ECGs approximately 2 minutes apart
- Administer first dose of TPX-0005 on an empty stomach (1 hour before dosing and 2 hours after dosing)
- **ALL patients**: Collect PK samples at 1, 2, and 4 hours (± 5 minutes), 6 and 8 hours (± 15 minutes), 24 hours (± 1 hour), and 48 hours (± 2 hours) post-dose***. Prior to blood sample collection, record triplicate 12-lead ECGs approximately 2 minutes apart.***
- Record any adverse events

8.1.3 Cycle 1 Day 1 (Phase 1a Dose Escalation)

- Perform a complete physical examination
- Perform and record vital signs, body weight, pain level (0-10), and ECOG PS
- Review medical history
- Review concomitant medications
- Record any adverse events
- Collect blood and urine for clinical laboratory assessments
- **All patients**: Collect a baseline pre-dose PK sample
- Perform and record pre-dose ECGs:
    - ❖ **All patients**: triplicate 12-lead ECGs approximately 2 minutes apart
- Administer TPX-0005 on an empty stomach (1 hour before dosing and 2 hours after dosing)
- **All patients**: At 4 hours (± 15 minutes) post-dose, record triplicate 12-lead ECGs approximately 2 minutes apart and collect a corresponding PK blood sample
- Dispense study medication for the next cycle (21 days) of treatment

8.1.4 Cycle 1 Day 8 (Phase 1a Dose Escalation)

- Perform a complete physical examination
- Perform and record vital signs, body weight, pain level (0-10), and ECOG PS
- Review medical history
- Assess study drug compliance
- Review concomitant medications
- Record any adverse events
- Collect blood and urine for clinical laboratory assessments
- **All patients**: Collect a baseline pre-dose PK sample
- Perform and record pre-dose ECGs:
    - ❖ **All patients**: triplicate 12-lead ECGs approximately 2 minutes apart
- Administer TPX-0005 on an empty stomach (1 hour before dosing and 2 hours after dosing)

Approved v1.0

8.1.5 Cycle 1 Day 15 (Phase 1a Dose Escalation)

- Perform a complete physical examination
- Perform and record vital signs, body weight, pain level (0-10), and ECOG PS
- Review medical history
- Assess study drug compliance
- Review concomitant medications
- Record any adverse events
- Collect blood and urine for clinical laboratory assessments
- **All patients**: Collect a baseline pre-dose PK sample
- Perform and record pre-dose ECGs:
  - ❖ **All patients**: Triplicate 12-lead ECGs approximately 2 minutes apart
- Administer TPX-0005 on an empty stomach (1 hour before dosing and 2 hours after dosing)
- **ALL patients**: Collect PK samples at 1, 2, and 4 hours (± 5 minutes), 6 and 8 hours (± 15 minutes), and 24 hours (± 1 hour) post-dose. ***Prior to blood sample collection, record triplicate 12-lead ECGs approximately 2 minutes apart.***

8.1.6 Cycle 2 Day 1 (Phase 1a Dose Escalation)

- Perform a complete physical examination
- Perform and record vital signs, body weight, pain level (0-10), and ECOG PS
- Review medical history
- Assess study drug compliance
- Review concomitant medications
- Record any adverse events
- **Female patients with childbearing potential:** Perform a serum pregnancy test
- Collect blood and urine for clinical laboratory assessments
- **All patients**: Collect a baseline pre-dose PK sample
- Perform and record pre-dose ECGs:
  - ❖ **All patients**: triplicate 12-lead ECGs approximately 2 minutes apart
- Administer TPX-0005 on an empty stomach (1 hour before dosing and 2 hours after dosing)
- **All patients**: At 4 hours (± 15 minutes) post-dose, record triplicate 12-lead ECGs approximately 2 minutes apart and collect a corresponding PK blood sample
- Dispense study medication for the next cycle (28 days) of treatment

Approved v1.0

8.1.7 Cycle 2 Day 15 (Phase 1a Dose Escalation)

- Perform a complete physical examination
- Perform and record vital signs, body weight, pain level (0-10), and ECOG PS
- Review medical history
- Assess study drug compliance
- Review concomitant medications
- Record any adverse events
- Collect blood for clinical laboratory assessments

8.1.8 Cycle 3 Day 1 (Phase 1a Dose Escalation)

- Perform a complete physical examination
- Perform and record vital signs, body weight, pain level (0-10), and ECOG PS
- Review medical history
- Assess study drug compliance
- Review concomitant medications
- Record any adverse events
- **Female patients with childbearing potential:** Perform a serum pregnancy test
- Collect blood and urine for clinical laboratory assessments
- Assess radiographic response to TPX-0005
- **All patients**: Collect a baseline pre-dose PK sample
- Perform and record pre-dose ECGs:
  - ❖ **All patients**: triplicate 12-lead ECGs approximately 2 minutes apart
- Administer TPX-0005 on an empty stomach (1 hour before dosing and 2 hours after dosing)
- **All patients**: At 4 hours (± 15 minutes) post-dose, record triplicate 12-lead ECGs approximately 2 minutes apart and collect a corresponding PK blood sample
- Dispense study medication for the next cycle (28 days) of treatment

Approved v1.0

8.1.9 Cycle 4 Day 1 (Phase 1a Dose Escalation)

- Perform a complete physical examination
- Perform and record vital signs, body weight, pain level (0-10), and ECOG PS
- Review medical history
- Assess study drug compliance
- Review concomitant medications
- Record any adverse events
- **Female patients with childbearing potential:** Perform a serum pregnancy test
- Obtain echocardiogram for LVEF assessment (and beginning of every 3 cycles, Cycle 4, 7, 10, 13)
- Collect blood and urine for clinical laboratory assessments
- **All patients**: Collect a baseline pre-dose PK sample
- Perform and record pre-dose ECGs:
  - ❖ **All patients**: triplicate 12-lead ECGs approximately 2 minutes apart
- Administer TPX-0005 on an empty stomach (1 hour before dosing and 2 hours after dosing)
- **All patients**: At 4 hours (± 15 minutes) post-dose, record triplicate 12-lead ECGs approximately 2 minutes apart and collect a corresponding PK blood sample
- Dispense study medication for the next cycle (28 days) of treatment

8.1.10 Every 4 Weeks from Cycle 5 Day 1 (Phase 1a Dose Escalation)

- Perform a complete physical examination
- Perform and record vital signs, body weight, pain level (0-10), and ECOG PS
- Review medical history
- Assess study drug compliance
- Review concomitant medications
- Record any adverse events
- **Female patients with childbearing potential:** Perform a serum pregnancy test
- Collect blood and urine for clinical laboratory assessments
- Assess radiographic response to study drug (at the beginning of every odd number of cycles)
- Obtain echocardiogram for LVEF assessment (at the beginning of every 3 cycles, Cycle 7, 10, 13)
- Dispense study medication for the next cycle (28 days) of treatment

Approved v1.0

8.1.11 End of Treatment (Phase 1a Dose Escalation) (within 7 days post last dose of TPX-0005 and after decision to end treatment)

- **Female patients with childbearing potential:** Perform a serum pregnancy test
- Collect blood and urine for clinical laboratory assessments
- Collect blood for ccfDNA
- Perform and record triplicate ECGs:
  - ❖ **All** patients: Triplicate 12-lead ECGs obtain approximately 2 minutes apart
- Obtain echocardiogram for LVEF assessment
- Assess study drug compliance (optional if performed at the time of the last dose)
- The following assessments are optional if performed within the past week:
  - ❖ Review concomitant medications
  - ❖ Record any adverse events
  - ❖ Review medical history
  - ❖ Perform complete physical examination
  - ❖ Perform and record vital signs, body weight, pain level (0-10), and ECOG PS
  - ❖ Perform tumor imaging, submit scans for BICR within 1 week, and assess locally as per RECIST version 1.1 (optional if performed within the past 4 weeks)

8.1.12 Safety Follow-up (Phase 1a Dose Escalation) (approximately 28 days post the last dose of TPX-0005)

- Review concomitant medications
- Record any adverse events
- Review medical history
- Perform complete physical examination
- Perform and record vital signs, body weight, pain level (0-10), and ECOG PS, as clinically indicated
- Collect blood and urine for clinical laboratory assessments, as clinically indicated

8.1.13 Survival Follow-up (Phase 1a Dose Escalation)

- For patients discontinuing the study treatment due to documented radiographic progression, obtain survival status *via* phone call or medical chart review, including information about subsequent anticancer therapies (including best response) every 3 months until death, loss of follow-up, or withdrawal of consent, whichever comes first.
- For patients discontinuing the study treatment prior to documented radiographic progression, tumor assessments should continue on schedule approximately every 2 cycle

until radiographic evidence of disease progression, the start of a subsequent anticancer therapy, or decision to no longer treat (e.g., supportive care only), whichever is first. At that time, survival status (and subsequent anticancer therapy information, including best response, if appropriate) will be collected every 3 months until death, loss of follow-up, or withdrawal of consent, whichever comes first.

8.2 Phase 1b Food Effect

8.2.1 Screening Phase 1b Food Effect (within 28 days of the first dose of TPX-0005)

- Obtain Informed Consent
- Eligibility checklist (See complete list in Sections 5.1 and 5.2)
- Perform molecular testing as per Section 5.1 and Table 16
  - ❖ Molecular testing to determine eligibility can be performed in advance with no time limit (e.g., the patient can be tested while being treated on another anticancer therapy)
  - ❖ Source documents (molecular pathology report detailing the specific test that detect the *ALK*, *ROS1*, or *NTRK* rearrangement, or *ALK* amplification or mutations in neuroblastoma) should be submitted for TP Therapeutics approval of eligibility
  - ❖ Submitted archival tissues to TP Therapeutics's designated central laboratory to confirm the specific molecular alteration
  - ❖ An optional tumor tissue re-biopsy (for patients who have failed prior TKIs) during the screening period to investigate resistance mechanism(s) is recommended but not mandatory
- Obtain detailed treatment history
  - ❖ Number of prior TKIs
  - ❖ Duration of treatment of each prior TKI
  - ❖ Investigator assessment of best response to each prior TKI
  - ❖ Number of prior chemotherapy regimens
  - ❖ Duration of treatment of each chemotherapy regimen
  - ❖ Investigator assessment of best response to each prior chemotherapy regimen
  - ❖ Number of prior immunotherapy regimens
  - ❖ Duration of treatment of each immunotherapy regimen
  - ❖ Investigator assessment of best response to each prior immunotherapy regimen
  - ❖ For patients with prior treated brain metastases, record the method of radiation: whole brain radiation, stereotactic radiosurgery, or both. Record also the dates of the radiation treatment(s), total dose delivered to each completed course of treatment, and date of last radiation to the start of TPX-0005 treatment
- Obtain detailed medical history

Approved v1.0

- Perform a complete physical examination
- Perform and record vital signs, height, body weight, pain level (0-10), and ECOG PS
- Review concomitant medications
- **Female patients with childbearing potential:** Perform a serum pregnancy test
- Collect blood and urine for clinical laboratory assessments and tumor markers
- Collect blood for ccfDNA
- Perform and record triplicate ECGs:
  - ❖ **All** patients: Triplicate 12-lead ECGs obtain approximately 2 minutes apart
- Obtain echocardiogram for baseline LVEF assessment
- Obtain cardiac troponin-I test
- Obtain baseline tumor assessment including CT chest, abdomen and pelvis, MRI of the brain and bone scan
- Patient registration

8.2.2 Lead-in PK Cycle 0 Day -3 (Phase 1b Food Effect)

- **Fasted Treatments:** Following an overnight fast of at least 10 hours, TPX-0005 should be administered with 8 oz (240 mL) of water. No food should be allowed for at least 4 hours post-dose. Water can be allowed as desired except for one hour before and after drug administration.
- **Fed Treatments:** Following an overnight fast of at least 10 hours, patients should start the recommended meal 30 minutes prior to administration of the drug product. Study subjects should eat this meal in 30 minutes or less; however, TPX-0005 should be administered 30 minutes after start of the meal. TPX-0005 should be administered with 8 oz (240 mL) of water. No food should be allowed for at least 4 hours post-dose. Water can be allowed as desired except for one hour before and after drug administration. Subjects should receive standardized meals scheduled at the same time [Guidance for Industry Food Effect Bioavailability and Fed Bioequivalence Studies. http://www.fda.gov/downloads/RegulatoryInformation/Guidances/UCM126833.pdf.].
- For patients starting with the **Fed** state, a high-fat (approximately 50 percent of total caloric content of the meal) and high-calorie (approximately 800 to 1000 calories) meal is recommended as a test meal. This test meal should derive approximately 150, 250, and 500-600 calories from protein, carbohydrate, and fat, respectively.
  - ❖ An example test meal would be 2 eggs fried in butter, 2 strips of bacon, 2 slices of toast with butter, 4 ounces of hash brown potatoes and 8 ounces of whole milk. Substitutions in this test meal can be made as long as the meal provides a similar amount of calories from protein, carbohydrate, and fat and has comparable meal volume and viscosity. The caloric breakdown of the test meal should be provided in the CRF.

Approved v1.0

- Patients assigned the Fed treatment on PK Lead-in Cycle will follow the Fasted treatment procedure on Cycle 1 Day 1. Patients assigned the Fasted treatment on PK Lead-in Cycle will follow the Fed treatment procedure on Cycle 1 Day 1.
- Review medical history
- Perform a complete physical examination
- Perform and record vital signs, body weight, pain level (0-10), and ECOG PS
- Review concomitant medications
- Record any adverse events
- **Female patients with childbearing potential:** Perform a serum pregnancy test
- Collect blood and urine for clinical laboratory assessments and tumor markers
- **All patients**: Collect a baseline pre-dose PK sample
- Perform and record pre-dose ECGs:
  - ❖ **All** patients: Triplicate 12-lead ECGs approximately 2 minutes apart
- Administer first dose of TPX-0005
- **ALL patients**: Collect PK samples at 1, 2, and 4 hours (±5 minutes), 6 and 8 hours (± 15 minutes), and 24 hours (± 1 hour) post-dose***. Prior to blood sample collection, record triplicate 12-lead ECGs approximately 2 minutes apart.***

8.2.3 Cycle 1 Day 1 (Phase 1b Food Effect)

- **Fasted Treatments:** Following an overnight fast of at least 10 hours, TPX-0005 should be administered with 8 oz (240 mL) of water. No food should be allowed for at least 4 hours post-dose. Water can be allowed as desired except for 1 hour before and after drug administration. Patient should receive standardized meals scheduled at the same time in each period of the study.
- **Fed Treatments:** Following an overnight fast of at least 10 hours, patients should start the recommended meal 30 minutes prior to administration of the drug product. Study subjects should eat this meal in 30 minutes or less; however, TPX-0005 should be administered 30 minutes after start of the meal. TPX-0005 should be administered with 8 oz (240 mL) of water. No food should be allowed for at least 4 hours post-dose. Water can be allowed as desired except for 1 hour before and after drug administration. Subjects should receive standardized meals scheduled at the same time in each period of the study [Guidance for Industry Food Effect Bioavailability and Fed Bioequivalence Studies. http://www.fda.gov/downloads/ Regulatory Information/Guidances/UCM126833.pdf.].
- For patients starting with the **Fed** state, a high-fat (approximately 50% of total caloric content of the meal) and high-calorie (approximately 800 to 1000 calories) meal is recommended as a test meal. This test meal should derive approximately 150, 250, and 500-600 calories from protein, carbohydrate, and fat, respectively.

Approved v1.0

- ❖ An example test meal would be 2 eggs fried in butter, 2 strips of bacon, 2 slices of toast with butter, 4 oz of hash brown potatoes and 8 oz of whole-fat milk. Substitutions in this test meal can be made as long as the meal provides a similar amount of calories from protein, carbohydrate, and fat and has comparable meal volume and viscosity. The caloric breakdown of the test meal should be provided in the CRF.

- Patients assigned the Fed treatment on PK Lead Cycle will follow the Fasted treatment procedure on Cycle 1 Day 1. Patients assigned the Fasted treatment on PK Lead Cycle will follow the Fed treatment procedure on Cycle 1 Day 1.
- Perform a complete physical examination
- Perform and record vital signs, body weight, pain level (0-10), and ECOG PS
- Review medical history
- Review concomitant medications
- Record any adverse events
- Collect blood and urine for clinical laboratory assessments
- **All patients**: Collect a baseline pre-dose PK sample
- Perform and record pre-dose ECGs:
  - ❖ **All patients**: triplicate 12-lead ECGs approximately 2 minutes apart
- Administer TPX-0005 according to either Fasted or Fed instructions.
- **ALL patients**: Collect PK samples at 1, 2, and 4 hours (± 5 minutes), 6 and 8 hours (± 15 minutes), and 24 hours (± 1 hour) post-dose***. Prior to blood sample collection, record triplicate 12-lead ECGs approximately 2 minutes apart.***
- Dispense study medication for the next cycle (21 days) of treatment

8.2.4 Cycle 1 Day 8 (Phase 1b Food Effect)

- Perform a complete physical examination
- Perform and record vital signs, body weight, pain level (0-10), and ECOG PS
- Review medical history
- Assess study drug compliance
- Review concomitant medications
- Record any adverse events
- Collect blood and urine for clinical laboratory assessments
- **All patients**: Collect a baseline pre-dose PK sample
- Perform and record pre-dose ECGs:
  - ❖ **All patients**: triplicate 12-lead ECGs approximately 2 minutes apart

- Administer TPX-0005 on an empty stomach (1 hour before dosing and 2 hours after dosing)
- One optional fresh tumor biopsy is recommended between Cycle 1 Day 8 and Cycle 1 Day 15

8.2.5 Cycle 1 Day 15 (Phase 1b Food Effect)

- Perform a complete physical examination
- Perform and record vital signs, body weight, pain level (0-10), and ECOG PS
- Review medical history
- Assess study drug compliance
- Review concomitant medications
- Record any adverse events
- Collect blood and urine for clinical laboratory assessments
- **All patients**: Collect a baseline pre-dose PK sample
- Perform and record pre-dose ECGs:
  - ❖ **All patients**: Triplicate 12-lead ECGs approximately 2 minutes apart
- Administer TPX-0005 on an empty stomach (1 hour before dosing and 2 hours after dosing)
- **All patients**: At 4 hours (± 15 minutes) post-dose, record triplicate 12-lead ECGs approximately 2 minutes apart and collect a corresponding PK blood sample

8.2.6 Cycle 2 Day 1 (Phase 1b Food Effect)

- Perform a complete physical examination
- Perform and record vital signs, body weight, pain level (0-10), and ECOG PS
- Review medical history
- Assess study drug compliance
- Review concomitant medications
- Record any adverse events
- **Female patients with childbearing potential:** Perform a serum pregnancy test
- Collect blood and urine for clinical laboratory assessments
- **All patients**: Collect a baseline pre-dose PK sample
- Perform and record pre-dose ECGs:
  - ❖ **All patients**: triplicate 12-lead ECGs approximately 2 minutes apart

Approved v1.0

- Administer TPX-0005 on an empty stomach (1 hour before dosing and 2 hours after dosing)
- **All patients**: At 4 hours (± 15 minutes) post-dose, record triplicate 12-lead ECGs approximately 2 minutes apart and collect a corresponding PK blood sample
- Dispense study medication for the next cycle (28 days) of treatment

8.2.7 Cycle 2 Day 15 (Phase 1b Food Effect)

- Perform a complete physical examination
- Perform and record vital signs, body weight, pain level (0-10), and ECOG PS
- Review medical history
- Assess study drug compliance
- Review concomitant medications
- Record any adverse events
- Collect blood for clinical laboratory assessments and tumor markers

8.2.8 Cycle 3 Day 1 (Phase 1b Food Effect)

- Perform a complete physical examination
- Perform and record vital signs, body weight, pain level (0-10), and ECOG PS
- Review medical history
- Assess study drug compliance
- Review concomitant medications
- Record any adverse events
- **Female patients with childbearing potential:** Perform a serum pregnancy test
- Collect blood and urine for clinical laboratory assessments
- Assess radiographic response to TPX-0005
- **All patients**: Collect a baseline pre-dose PK sample
- Perform and record pre-dose ECGs:
  - ❖ **All patients**: Triplicate 12-lead ECGs approximately 2 minutes apart
- Administer TPX-0005 on an empty stomach (1 hour before dosing and 2 hours after dosing)
- **All patients**: At 4 hours (+/- 15 minutes) post-dose, record triplicate 12-lead ECGs approximately 2 minutes apart and collect a corresponding PK blood sample
- Dispense study medication for the next cycle (28 days) of treatment

Approved v1.0

8.2.9 Cycle 4 Day 1 (Phase 1b Food Effect)

- Perform a complete physical examination
- Perform and record vital signs, body weight, pain level (0-10), and ECOG PS
- Review medical history
- Assess study drug compliance
- Review concomitant medications
- Record any adverse events
- **Female patients with childbearing potential:** Perform a serum pregnancy test
- Obtain echocardiogram for LVEF assessment (and beginning of every 3 cycles, Cycle 4, 7, 10, 13)
- Collect blood and urine for clinical laboratory assessments
- **All patients**: Collect a baseline pre-dose PK sample
- Perform and record pre-dose ECGs:
    - ❖ **All patients**: Triplicate 12-lead ECGs approximately 2 minutes apart
- Administer TPX-0005 on an empty stomach (1 hour before dosing and 2 hours after dosing)
- **All patients**: At 4 hours (± 15 minutes) post-dose, record triplicate 12-lead ECGs approximately 2 minutes apart and collect a corresponding PK blood sample
- Dispense study medication for the next cycle (28 days) of treatment

8.2.10 Every 4 Weeks from Cycle 5 Day 1 (Phase 1b Food Effect)

- Perform a complete physical examination
- Perform and record vital signs, body weight, pain level (0-10), and ECOG PS
- Review medical history
- Assess study drug compliance
- Review concomitant medications
- Record any adverse events
- **Female patients with childbearing potential:** Perform a serum pregnancy test
- Collect blood and urine for clinical laboratory assessments and tumor markers
- Assess radiographic response to study drug (at the beginning of every odd number of cycles)
- Obtain echocardiogram for LVEF assessment (at the beginning of every 3 cycles, Cycle 7, 10, 13)
- Dispense study medication for the next cycle (28 days) of treatment

8.2.11 End of Treatment (Phase 1b Food Effect) (within 7 days post last dose of TPX-0005 and after decision to end treatment)

- Perform tumor biopsy (if clinically feasible and patient has consented to the biopsy) and send tissue to TP Therapeutics's designated central laboratory
- Collect blood for ccfDNA
- **Female patients with childbearing potential:** Perform a serum pregnancy test
- Collect blood and urine for clinical laboratory assessments
- Perform and record triplicate ECGs:
  - ❖ **All** patients: Triplicate 12-lead ECGs obtain approximately 2 minutes apart
- Obtain echocardiogram for LVEF assessment
- Assess study drug compliance (optional if performed at the time of the last dose)
- The following assessments are optional if performed within the past week:
  - ❖ Review concomitant medications
  - ❖ Record any adverse events
  - ❖ Perform complete physical examination
  - ❖ Perform and record vital signs, body weight, pain level (0-10), and ECOG PS
  - ❖ Perform tumor imaging, submit scans for BICR within 1 week, and assess locally as per RECIST version 1.1 (optional if performed within the past 4 weeks)

8.2.12 Safety Follow-up (Phase 1b Food Effect) (approximately 28 days post the last dose of TPX-0005)

- Review concomitant medications
- Record any adverse events
- Review medical history
- Perform complete physical examination
- Perform and record vital signs, body weight, pain level (0-10), and ECOG PS, as clinically indicated
- Collect blood and urine for clinical laboratory assessments, as clinically indicated

8.2.13 Survival follow-up (Phase 1b Food Effect)

- For patients discontinuing the study treatment due to documented radiographic progression, obtain survival status via phone call or medical chart review, including information about subsequent anticancer therapies (including best response) every 3 months until death, loss of follow-up, or withdrawal of consent, whichever comes first.

Approved v1.0

- For patients discontinuing the study treatment prior to documented radiographic progression, tumor assessments should continue on schedule approximately every 2 cycles until radiographic evidence of disease progression, the start of a subsequent anticancer therapy, or decision to no longer treat (e.g., supportive care only), whichever is first. At that time, survival status (and subsequent anticancer therapy information, including best response, if appropriate) will be collected every 3 months until death, loss of follow-up, or withdrawal of consent, whichever comes first.

8.3 Phase 2 Dose Expansion

8.3.1 Screening Phase 2 Dose Expansion

- Obtain Informed Consent
- Eligibility checklist (see complete list in Sections 5.1 and 5.2)
- Perform molecular testing as per Section 5.1 and Table 17
  - ❖ Molecular testing to determine eligibility can be performed in advance with no time limit (e.g., the patient can be tested while being treated on another anticancer therapy)
  - ❖ Source documents (molecular pathology report detailing the specific test that detect the *ALK*, *ROS1*, or *NTRK* rearrangement, or *ALK* amplification or mutations in neuroblastoma) should be submitted for TP Therapeutics approval of eligibility
  - ❖ Submitted archival tissues to TP Therapeutics's designated central laboratory to confirm the specific molecular alteration
  - ❖ An optional tumor tissue re-biopsy (for patients who have failed prior TKIs) during the screening period to investigate resistance mechanism(s) is recommended but not mandatory
- Obtain detailed treatment history
  - ❖ Number of prior TKIs
  - ❖ Duration of treatment of each prior TKI
  - ❖ Investigator assessment of best response to each prior TKI
  - ❖ Number of prior chemotherapy regimens
  - ❖ Duration of treatment of each chemotherapy regimen
  - ❖ Investigator assessment of best response to each prior chemotherapy regimen
  - ❖ Number of prior immunotherapy regimens
  - ❖ Duration of treatment of each immunotherapy regimen
  - ❖ Investigator assessment of best response to each prior immunotherapy regimen
  - ❖ For patients with prior treated brain metastases, record the method of radiation: whole brain radiation, stereotactic radiosurgery, or both. Record also the dates of the radiation treatment(s), total dose each completed course of treatment delivered, and date of last radiation to the start of TPX-0005 treatment
- Obtain detailed medical history

Approved v1.0

- Perform a complete physical examination
- Perform and record vital signs, height, body weight, pain level (0-10), and ECOG PS
- Review concomitant medications
- **Female patients with childbearing potential:** Perform a serum pregnancy test
- Collect blood and urine for clinical laboratory assessments
- Collect blood for ccfDNA
- Perform and record triplicate ECGs:
  - ❖ **All** patients: Triplicate 12-lead ECGs obtain approximately 2 minutes apart
- Obtain echocardiogram for baseline LVEF assessment
- Obtain cardiac troponin-I test
- Obtain baseline tumor assessment including CT chest, abdomen and pelvis, MRI of the brain and bone scan.
- Administer QOL questionnaires:
  - ❖ EORTC-QLQ-C30 (all patients)
  - ❖ EORTC-QLQ-LC13 (NSCLC patients only)
- Patient registration and assignment to one of the 10 expansion cohorts

8.3.2 Cycle 1 Day 1 (Phase 2 Dose Expansion)

- Administer QOL questionnaires:
  - ❖ EORTC-QLQ-C30 (all patients)
  - ❖ EORTC-QLQ-LC13 (NSCLC patients only)
- Review medical history
- Review concomitant medications
- Record any adverse events
- Perform a complete physical examination
- Perform and record vital signs, body weight, pain level (0-10), and ECOG PS
- **Female patients with childbearing potential:** Perform a serum pregnancy test
- Collect blood and urine for clinical laboratory assessments (***optional if performed within the past 7 days***)
- Collect a baseline pre-dose PK sample
- Perform and record pre-dose ECGs:
  - ❖ **All patients**: triplicate 12-lead ECGs approximately 2 minutes apart

Approved v1.0

- Administer first dose of TPX-0005 on an empty stomach (1 hour before dosing and 2 hours after dosing)
- At 4 hours (± 15 minutes) post-dose, record triplicate 12-lead ECGs approximately 2 minutes apart and collect a corresponding PK blood sample
- Dispense TPX-0005 for the next cycle (28 days) of treatment

8.3.3 Cycle 1 Day 8 (Phase 2 Dose Expansion)

- Perform a complete physical examination
- Perform and record vital signs, body weight, pain level (0-10), and ECOG PS
- Review medical history
- Assess study drug compliance
- Review concomitant medications
- Record any adverse events
- Collect blood and urine for clinical laboratory assessments

8.3.4 Cycle 1 Day 15 (Phase 2 Dose Expansion)

- Perform a complete physical examination
- Perform and record vital signs, body weight, pain level (0-10), and ECOG PS
- Review medical history
- Assess study drug compliance
- Review concomitant medications
- Record any adverse events
- Collect blood and urine for clinical laboratory assessments
- Collect a baseline pre-dose PK sample
- Perform and record pre-dose ECGs:
  - ❖ **All patients**: triplicate 12-lead ECGs approximately 2 minutes apart
- Administer TPX-0005 on an empty stomach (1 hour before dosing and 2 hours after dosing)
- At 4 hours (± 15 minutes) post-dose, record triplicate 12-lead ECGs approximately 2 minutes apart and collect a corresponding PK blood sample

Approved v1.0

8.3.5 Cycle 1 Day 21 (Phase 2 Dose Expansion)

- Perform a complete physical examination
- Perform and record vital signs, body weight, pain level (0-10), and ECOG PS
- Review medical history
- Assess study drug compliance
- Review concomitant medications
- Record any adverse events
- Collect blood and urine for clinical laboratory assessments

8.3.6 Cycle 2 Day 1 (Phase 2 Dose Expansion)

- Administer QOL questionnaires:
  - ❖ EORTC-QLQ-C30 (all patients)
  - ❖ EORTC-QLQ-LC13 (NSCLC patients only)
- Perform a complete physical examination
- Perform and record vital signs, body weight, pain level (0-10), and ECOG PS
- Review medical history
- Assess study drug compliance
- Review concomitant medications
- Record any adverse events
- **Female patients with childbearing potential:** Perform a serum pregnancy test
- Collect blood and urine for clinical laboratory assessments
- Collect a baseline pre-dose PK sample
- Perform and record pre-dose ECGs:
  - ❖ **All patients**: triplicate 12-lead ECGs approximately 2 minutes apart
- Administer TPX-0005 on an empty stomach (1 hour before dosing and 2 hours after dosing)
- At 4 hours (± 15 minutes) post-dose, record triplicate 12-lead ECGs approximately 2 minutes apart and collect a corresponding PK blood sample
- Dispense study medication for the next cycle (28 days) of treatment

8.3.7 Cycle 2 Day 15 (Phase 2 Dose Expansion)

- Perform a complete physical examination
- Perform and record vital signs, body weight, pain level (0-10), and ECOG PS
- Review medical history
- Assess study drug compliance
- Review concomitant medications
- Record any adverse events
- Collect blood for clinical laboratory assessments

8.3.8 Cycle 3 Day 1 (Phase 2 Dose Expansion)

- Administer QoL questionnaires:
  - ❖ EORTC-QLQ-C30 (all patients)
  - ❖ EORTC-QLQ-LC13 (NSCLC patients only)
- Perform a complete physical examination
- Perform and record vital signs, body weight, pain level (0-10), and ECOG PS
- Review medical history
- Assess study drug compliance
- Review concomitant medications
- Record any adverse events
- **Female patients with childbearing potential:** Perform a serum pregnancy test
- Collect blood and urine for clinical laboratory assessments
- Assess radiographic response to study drug (**at the beginning of every odd cycle of treatment OR at the end of every even cycle of treatment**)
- Collect a baseline pre-dose PK sample
- Perform and record pre-dose ECGs:
  - ❖ **All patients**: triplicate 12-lead ECGs approximately 2 minutes apart
- Administer TPX-0005 on an empty stomach (1 hour before dosing and 2 hours after dosing)
- At 4 hours (± 15 minutes) post-dose, record triplicate 12-lead ECGs approximately 2 minutes apart and collect a corresponding PK blood sample
- Dispense study medication for the next cycle (28 days) of treatment

8.3.9 Cycle 4 Day 1 and Each Subsequent Treatment Cycle (Phase 2 Dose Expansion)

- Administer quality of life questionnaires:
  - ❖ QLQ-C30 (all patients)
  - ❖ QLQ-LC13 (NSCLC patients only)
- Perform a complete physical examination
- Perform and record vital signs, body weight, pain level (0-10), and ECOG PS
- Review medical history
- Assess study drug compliance
- Review concomitant medications
- Record any adverse events
- **Female patients with childbearing potential:** Perform a serum pregnancy test
- Obtain echocardiogram for LVEF assessment (and beginning of every 3 cycles, Cycle 4, 7, 10, 13)
- Collect blood and urine for clinical laboratory assessments
- Collect a baseline pre-dose PK sample
- Perform and record pre-dose ECGs:
  - ❖ **All patients**: triplicate 12-lead ECGs approximately 2 minutes apart
- Administer TPX-0005 on an empty stomach (1 hour before dosing and 2 hours after dosing)
- At 4 hours (± 15 minutes) post-dose, record triplicate 12-lead ECGs approximately 2 minutes apart and collect a corresponding PK blood sample
- Dispense study medication for the next cycle (28 days) of treatment

8.3.10 End of Treatment (Phase 2 Dose Expansion) (within 7 days post last dose of TPX-0005 and after decision to end treatment)

- Perform tumor biopsy (if clinically feasible and patient has consented to the biopsy) and send tissue to TP Therapeutics's designated central laboratory
- Collect blood for ccfDNA
- **Female patients with childbearing potential:** Perform a serum pregnancy test.
- Collect blood and urine for clinical laboratory assessments
- Perform and record triplicate ECGs:
  - ❖ **All patients**: triplicate 12-lead ECGs approximately 2 minutes apart

- Obtain echocardiogram for LVEF assessment
- Administer quality of life questionnaires (optional if performed within the past 2 weeks)
  - ❖ QLQ-C30 (all patients)
  - ❖ QLQ-LC13 (NSCLC patients only)
- Assess study drug compliance (optional if performed at the time of the last dose)
- The following assessments are optional if performed within the past week:
  - ❖ Review concomitant medications
  - ❖ Record any adverse events
  - ❖ Review medical history
  - ❖ Perform complete physical examination
  - ❖ Perform and record vital signs, body weight, pain level (0-10), and ECOG PS
  - ❖ Perform tumor imaging, submit scans for BICR within 1 week, and assess locally as per RECIST version 1.1 (optional if performed within the past 4 weeks)

8.3.11 Safety Follow-up (Phase 2 Dose Expansion) (approximately 28 days post the last dose of TPX-0005)

- Review concomitant medications
- Record any adverse events
- Review medical history
- Perform complete physical examination
- Perform and record vital signs, body weight, pain level (0-10), and ECOG PS, as clinically indicated
- Collect blood and urine for clinical laboratory assessments, as clinically indicated

8.3.12 Survival Follow-up (Phase 2 Dose Expansion)

- For patients discontinuing the study treatment due to documented radiographic progression, obtain survival status via phone call or medical chart review, including information about subsequent anticancer therapies (including best response) every 3 months until death, loss of follow-up, or withdrawal of consent, whichever comes first.
- For patients discontinuing the study treatment prior to documented radiographic progression, tumor assessments should continue on schedule approximately every 2 cycles until radiographic evidence of disease progression, the start of a subsequent anticancer therapy, or decision to no longer treat (e.g., supportive care only), whichever is first. At that time, survival status (and subsequent anticancer therapy information, including best response, if appropriate) will be collected every 3 months until death, loss of follow-up, or withdrawal of consent, whichever comes first.

Approved v1.0

8.4 Study Calendars

Table 15 Study Calendar for Phase 1a Dose Escalation Portion of TPX-0005-01

	Screening	Lead-in PK/Cycle 0 (3 days)	Cycle 1 (21-day cycle)			Cycle 2 (28-day cycle)		Cycle 3 and Cycle 4	Cycle 5 and Beyond	End of Treatment	Safety Follow-up	Long-Term Follow-up
**Protocol Activity**	**Days -28 to -1**	**Day -3**	**Day 1**	**Day 8**	**Day 15**	**Day 1**	**Day 15**	**Day 1**	**Day 1**			
Visit Window[1]	NA			±1	±1	±2	±2	±2	±2	±7	+7	
Informed Consent[2]	X											
Tumor Molecular Alteration[3]	X											
Tumor Treatment History[4]	X											
Medical History	X	X	X	X	X	X	X	X	X	X	X	
Physical Examination	X	X	X	X	X	X	X	X	X	X	X	
ECOG Performance Status	X	X	X	X	X	X	X	X	X	X	X	
Height	X											
Weight	X	X	X	X	X	X	X	X	X	X	X	
Vital Signs[5]	X	X	X	X	X	X	X	X	X	X	X	
Dispense TPX-0005			X			X		X	X			
TPX-0005 Compliance				X	X	X	X	X	X	X		
Laboratory												
Complete Blood Count with Differential[6]	X	X	X	X	X	X	X	X		X	X	
Reticulocyte Count (%)	X	X	X			X		X	X	X	X	
Complete Metabolic Panel[7]	X	X	X	X	X	X	X	X	X	X	X	
Coagulation[8]	X	X	X	X	X	X		X	X	X	X	
Endocrine (for male patients only)[9]	X	X				X		X	X	X	X	

	Screening	Lead-in PK/Cycle 0 (3 days)	Cycle 1 (21-day cycle)			Cycle 2 (28-day cycle)		Cycle 3 and Cycle 4	Cycle 5 and Beyond	End of Treatment	Safety Follow-up	Long-Term Follow-up
**Protocol Activity**	**Days -28 to -1**	**Day -3**	**Day 1**	**Day 8**	**Day 15**	**Day 1**	**Day 15**	**Day 1**	**Day 1**			
Pregnancy Test (serum)[10]	X	X				X		X	X	X	X	
Urine Analysis[11]	X	X	X	X	X	X		X	X	X	X	
Blood Specimens for ccfDNA Enrichment[12]	X									X		
Cardiac Safety Monitoring												
Triplicate 12-lead ECG[13]	X	X	X	X	X	X		X		X		
Echocardiogram for LVEF Assessment[14]	X							X	X	X		
Cardiac Troponin-I[15]	X											
Pharmacokinetics												
Plasma for Full PK[16]		X			X							
Plasma for Abbreviated PK[17]			X	X		X		X				
CSF (Optional)[18]						X						
Tumor Assessment												
CT or MRI (chest/abdomen/pelvis)[19]	X							X	X			
MRI of Brain[20]	X							X	X			
Bone Scan[21]	X							X	X			
Other Clinical Assessments												
Adverse Events		X	X	X	X	X	X	X	X	X	X	
Concomitant Medications[22]	X	X	X	X	X	X	X	X	X	X	X	
Survival Follow-Up												X

[1] **Visit Window:** The 28-day screening period starts on the day informed consent is signed by the patient. The safety visit should be within 28 days from the last dose of TPX-0005.

[2] **Informed Consent:** Must be obtained before undergoing any protocol-specific procedures.

[3] **Tumor Molecular Alteration:** Confirmation of *ALK+, ROS1+*, or *NTRK+* rearrangement (submit molecular pathology report-source document) and submit archival tumor tissue (please refer to Section 5.1).

[4] **Tumor Treatment History:** Documentation of disease progression on prior tyrosine kinase inhibitor (TKIs), duration of treatment with each prior TKI, prior chemotherapy regimens and duration of each chemotherapy regimens, prior immunotherapy regimens and duration of each immunotherapy regimens (if applicable), prior radiation to brain (if brain metastases present, and methods of radiation: whole brain radiation, stereotactic radiosurgery).

[5] **Vital Signs:** Body temperature, blood pressure, heart rate, respiratory rate, pain level (0-10).

[6] **Complete Blood Count with Differential:** White blood cell count, hemoglobin, platelet, absolute neutrophils, absolute lymphocytes, absolute monocytes, absolute eosinophils, and absolute basophils.

[7] **Complete Chemistry Panel:** Sodium, potassium, chloride, bicarbonate, blood urea nitrogen, creatinine, magnesium, phosphorus, calcium, uric acid, total protein, albumin, lactate dehydrogenase, aspartate aminotransferase, alanine aminotransferase, total bilirubin, alkaline phosphatase.

[8] **Coagulation:** Prothrombin time/international normalized ratio, partial thromboplastin time.

[9] **Endocrine (for male patients only):** Total testosterone, sex hormone-binding globulin, follicle-stimulating hormone, luteinizing hormone, prolactin. These laboratories should be drawn between 8 am to 11 am in the morning.

[10] **Serum Pregnancy Test:** For female patients of childbearing potential, a serum pregnancy test, with sensitivity of at least 25 mIU/mL, will be performed on 2 occasions prior to starting study therapy: once at the start of screening and once at the baseline visit, immediately before investigational product administration. Only patients with negative serum pregnancy tests are allowed to enroll. Serum pregnancy test will also be performed at the beginning of each cycle of visit.

[11] **Urine Analysis:** Urinalysis includes dipstick analysis (protein, glucose, ketones, blood, and specific gravity). A microscopic (white blood cells/high power field [HPF], red blood cells/HPF, and any additional findings) exam need only be performed if the urinalysis result is abnormal. More frequent and complete examinations may be performed at the Investigator's discretion if medically indicated; results should be recorded on unscheduled visit CRFs.

[12] **Blood Specimens for ccfDNA Enrichment (20 mL blood specimen):** Blood will be collected across 2 10 ml Streck Cell-Free DNA Blood Collection Tubes (for ccfDNA analysis) prior to the first dose of TPX-0005 and at EOT for exploratory ccfDNA molecular profiling analyses. Details for handling of these specimens including processing, storage, and shipment will be provided in the Study Manual.

[13] **Triplicate 12-lead ECGs:** At each time point, 3 consecutive 12-lead ECGs will be performed approximately 2 minutes apart to determine mean QTc interval. Triplicate 12-lead ECGs will all be time matched with PK with the exception of during the screening period and at the EOT. Triplicate ECGs will be collected as follows:

a) At Screening.
b) Day -3 (Lead-in/Cycle 0) after single-dose administration at pre-dose (0 hour), at 1, 2, 4, 6, 8, 24, and 48 hours post-dose.
c) Cycle 1 Day 1, at pre-dose (0 hour) and 4 hours post-dose.
d) Cycle 1 Day 8, at pre-dose (0 hour).
e) Cycle 1 Day 15 after single-dose administration at pre-dose (0 hour), at 1, 2, 4, 6, 8, and 24 hours post-dose.
f) Cycles 2-4, Day 1 at pre-dose (0 hour) and 4 hours post-dose.

In addition to these time points, ECGs should be repeated as clinically indicated. Additional ECG time points may be included based on the emerging data. Interpretation of the tracing will be made by a central ECG laboratory. Each ECG tracing should be labeled with the study number, patient initials, patient number, date, and kept in the source documents at the study site. Only clinically significant abnormalities will be recorded in the AE CRF page. Clinically significant abnormalities at screening/baseline should be

Approved v1.0

recorded on the relevant medical history/current medical conditions CRF page. Clinically significant findings must be discussed with TP Therapeutics and Medical Monitor prior to enrolling the patient in the study.

14 **Echocardiography for LVEF Assessments:** Echocardiography to be performed at screening, and after every 3 cycles of treatment (± 7 days) (Cycles 4, 7, 10, 13 and so on), and at the EOT visit (± 7 days).

15 **Cardiac Troponin-I:** Cardiac troponin-I will be done at baseline then repeated and assessed whenever the ECG morphology demonstrates possible myocardial ischemia or infarction. If troponin CTCAE grade ≥ 3 is seen, this parameter must be repeated twice a week until resolution to CTCAE grade ≤ 1.

16 **Plasma for Full PK:** Timing for full PK: Lead-in PK pre-dose (time 0), 1, 2, 4, 6, 8, 24, and 48 hours post-dose. Cycle 1 Day 15 pre-dose (time 0), 1, 2, 4, 6, 8, and 24 hours post-dose.

17 **Plasma for Abbreviated PK:** Timing for abbreviated PK: at pre-dose (0 hour) and 4 hours post-dose on Cycle 1 Day 1 and Cycles 2-4 Day 1, and at pre-dose (0 hour) on Cycle 1 Day 8.

18 **Cerebral Spinal Fluid (CSF) Collection (optional):** If a patient undergoes a lumber puncture, a sample of CSF should be collected for exploratory analysis of TPX-0005 concentration, if possible. If a CSF sample is collected, a blood sample for PK analysis should also be collected at approximately the same time as the CSF sample.

19 **CT or MRI Chest/Abdomen/Pelvis Tumor Assessment:** Tumor assessments will include all known or suspected disease sites. For all tumor assessments, the method of assessment that was used at baseline should be the same method used throughout the study. For patients who are without documented disease progression, CT or MRI scans to be done at every 2 cycles (after 7 weeks for the first assessment and then every 2 cycles up to Cycle 18 and then every 3 cycles up to Cycle 36 and then every 4 cycles thereafter until documented progression of disease). Patient responses will be confirmed > 4 weeks later after the initial documentation of response by the Investigator. For patients who have documented disease progression but are continuing TPX-0005 post-RECIST progression, CT or MRI scans are to be done according to local institutional standard of care. Every effort should be made to maintain the assessment scheduling relative to Cycle 1 Day 1 especially if there are dosing cycle interruptions due to toxicities. For all patients, copies of radiologic images must be available for independent central radiology review as determined by TP Therapeutics.

20 **MRI of Brain:** Gadolinium contrast-enhanced MRI must be used for assessment of CNS lesions at baseline with contingent slices of 1 mm for lesions 5 to 10 mm in size, 5 mm for lesions greater than 10 mm. For the Phase 1a Dose Escalation portion of the study, only patients with documented baseline CNS metastases will be assessed by MRI of the brain at every 2 cycles (after 7 weeks for the first assessment and then every 2 cycles up to Cycle 18 and then every 3 cycles up to Cycle 36 and then every 4 cycles thereafter until documented progression of disease). Patient responses will be confirmed > 4 weeks later after the initial documentation of response by the Investigator. For patients who have documented disease progression but are continuing TPX-0005 post-RECIST progression, MRI scans of the brain are to be done according to local institutional standard of care.

21 **Bone Scans:** Bone scans (or bone MRI if preferred by Investigator) will be performed at baseline for all patients and repeated every 3 cycles while on study only if evidence of bone metastases is observed at baseline.

22 **Concomitant Medications and Non-Drug Supportive Interventions:** All concomitant medications and non-drug supportive interventions should be recorded in the CRF.

Approved v1.0

Table 16 Study Calendar for Phase 1b Food Effect Portion of TPX-0005-01

	**Screening**	**Lead-in PK/Cycle 0 (3 days)**	**Cycle 1 (21-day cycle)**			**Cycle 2 (28-day cycle)**		**Cycle 3 and Cycle 4**	**Cycle 5 and Beyond**	**End of Treatment**	**Safety Follow-up**	**Long-Term Follow-up**
**Protocol Activity**	**Days -28 to -1**	**Day -3**	**Day 1**	**Day 8**	**Day 15**	**Day 1**	**Day 15**	**Day 1**	**Day 1**			
Visit Window[1]	NA			±1	±1	±2	±2	±2	±2	±7	+7	
Informed Consent[2]	X											
Tumor Molecular Alteration[3]	X											
Tumor Treatment History[4]	X											
Medical History	X	X	X	X	X	X	X	X	X	X	X	
Physical Examination	X	X	X	X	X	X	X	X	X	X	X	
ECOG Performance Status	X	X	X	X	X	X	X	X	X	X	X	
Height	X											
Weight	X	X	X	X	X	X	X	X	X	X	X	
Vital Signs[5]	X	X	X	X	X	X	X	X	X	X	X	
Dispense TPX-0005			X			X		X	X			
TPX-0005 Compliance				X	X	X	X	X	X	X		
Laboratory												
Complete Blood Count with Differential[6]	X	X	X	X	X	X	X	X	X	X	X	
Reticulocyte Count (%)	X	X	X			X		X	X	X	X	
Complete Metabolic Panel[7]	X	X	X	X	X	X	X	X	X	X	X	
Coagulation[8]	X	X	X	X	X	X		X	X	X	X	
Endocrine (for male patients only)[9]	X		X			X		X	X	X	X	
Pregnancy Test (serum)[10]	X	X				X		X	X	X	X	
Urine Analysis[11]	X	X	X	X	X	X		X	X	X	X	

	Screening	Lead-in PK/Cycle 0 (3 days)	Cycle 1 (21-day cycle)			Cycle 2 (28-day cycle)		Cycle 3 and Cycle 4	Cycle 5 and Beyond	End of Treatment	Safety Follow-up	Long-Term Follow-up
**Protocol Activity**	**Days -28 to -1**	**Day -3**	**Day 1**	**Day 8**	**Day 15**	**Day 1**	**Day 15**	**Day 1**	**Day 1**			
Blood Specimens for ccfDNA Enrichment[12]	X									X		
Cardiac Safety Monitoring												
Triplicate 12-lead ECG[13]	X	X	X	X	X	X		X		X		
Echocardiogram for LVEF Assessment[14]	X							X	X	X		
Cardiac Troponin-I[15]	X											
Pharmacokinetics												
Plasma for Full PK[16]		X	X									
Plasma for Abbreviated PK[17]				X	X	X		X				
CSF (optional)[18]						X						
Tumor Assessment												
CT or MRI (Chest/Abdomen/Pelvis)[19]	X							X	X			
MRI of Brain[20]	X							X	X			
Bone Scan[21]	X							X	X			
Other Clinical Assessments												
Adverse Events		X	X	X	X	X	X	X	X	X	X	
Pharmacodynamic (PD) Assessment[22]				X								
Concomitant Medications[23]	X	X	X	X	X	X	X	X	X	X	X	
Survival Follow-Up												X

[1] **Visit Window:** The 28-day screening period starts on the day informed consent is signed by the patient. The safety visit should be within 28 days from the last dose of TPX-0005.

[2] **Informed Consent:** Must be obtained before undergoing any protocol-specific procedures.

[3] **Tumor Molecular Alteration:** Confirmation of *ALK+, ROS1+, or NTRK+* rearrangement (submit molecular pathology report-source document) and submit archival tumor tissue (please refer to Section 5.1).

[4] **Tumor Treatment History:** Documentation of disease progression on prior tyrosine kinase inhibitor (TKIs), duration of treatment with each prior TKI, prior chemotherapy regimens and duration of each chemotherapy regimens, prior immunotherapy regimens and duration of each immunotherapy regimens (if applicable), prior radiation to brain (if brain metastases present, and methods of radiation: whole brain radiation, stereotactic radiosurgery).

[5] **Vital Signs:** Body temperature, blood pressure, heart rate, respiratory rate, pain level (0-10).

[6] **Complete Blood Count with Differential:** White blood cell count, hemoglobin, platelet, absolute neutrophils, absolute lymphocytes, absolute monocytes, absolute eosinophils, and absolute basophils.

[7] **Complete Chemistry Panel:** Sodium, potassium, chloride, bicarbonate, blood urea nitrogen, creatinine, magnesium, phosphorus, calcium, uric acid, total protein, albumin, lactate dehydrogenase, aspartate aminotransferase, alanine aminotransferase, total bilirubin, alkaline phosphatase.

[8] **Coagulation:** Prothrombin time/international normalized ratio, partial thromboplastin time.

[9] **Endocrine (for male patients only):** Total testosterone, sex hormone-binding globulin, follicle-stimulating hormone, luteinizing hormone, prolactin. These laboratories should be drawn between 8 am to 11 am in the morning.

[10] **Serum Pregnancy Test:** For female patients of childbearing potential, a serum pregnancy test, with sensitivity of at least 25 mIU/mL, will be performed on 2 occasions prior to starting study therapy: once at the start of screening and once at the baseline visit, immediately before investigational product administration. Only patients with negative serum pregnancy tests are allowed to enroll. Serum pregnancy test will also be performed at the beginning of each cycle of visit.

[11] **Urine Analysis:** Urinalysis includes dipstick analysis (protein, glucose, ketones, blood, and specific gravity). A microscopic (white blood cells/high power field [HPF], red blood cells/HPF, and any additional findings) exam need only be performed if the urinalysis result is abnormal. More frequent and complete examinations may be performed at the Investigator's discretion if medically indicated; results should be recorded on unscheduled visit CRFs.

[12] **Blood Specimens for ccfDNA Enrichment (20 mL blood specimen):** Blood will be collected across 2 10-ml Streck Cell-Free DNA Blood Collection Tubes (for ccfDNA analysis) prior to the first dose of TPX-0005 and at the EOT for ccfDNA enumeration and exploratory molecular profiling analyses. Details for handling of these specimens including processing, storage, and shipment will be provided in the Study Manual.

[13] **Triplicate 12-lead ECGs:** At each time point, 3 consecutive 12-lead ECGs will be performed approximately 2 minutes apart to determine mean QTc interval. Triplicate 12-lead ECGs will all be time matched with PK with the exception of during the screening period and at EOT. Triplicate ECGs will be collected as follows:

a) At Screening.
b) Day -3 (Lead-in/Cycle 0) after single-dose administration at pre-dose (0 hour), at 1, 2, 4, 6, 8, and 24 hours post-dose.
c) Cycle 1 Day 1, after single-dose administration at pre-dose (0 hour), at 1, 2, 4, 6, 8, and 24, hours post-dose.
d) Cycle 1 Day 8, at pre-dose (0 hour).
e) Cycle 1 Day 15, Cycles 2-4, Day 1 at pre-dose (0 hour) and 4 hours post-dose.

In addition to these time points, ECGs should be repeated as clinically indicated. Additional ECG time points may be included based on the emerging data. Interpretation of the tracing will be made by a central ECG laboratory. Each ECG tracing should be labeled with the study number, patient initials, patient number, date, and kept in the source documents at the study site. Only clinically significant abnormalities will be recorded in the AE CRF page. Clinically significant abnormalities at screening/baseline should be recorded on the relevant medical history/current medical conditions CRF page. Clinically significant findings must be discussed with TP Therapeutics and Medical Monitor prior to enrolling the patient in the study.

Approved v1.0

[14] **Echocardiography for LVEF Assessments:** Echocardiography to be performed at screening, and after every 3 cycles of treatment (± 7 days) (Cycles 4, 7, 10, 13 and so on), and at the EOT visit (± 7 days).

[15] **Cardiac Troponin-I:** Cardiac troponin-I will be done at baseline then repeated and assessed whenever the ECG morphology demonstrates possible myocardial ischemia or infarction. If troponin CTCAE grade ≥ 3 is seen, this parameter must be repeated twice a week until resolution to CTCAE grade ≤ 1.

[16] **Plasma for Full PK:** Timing for full PK: Lead-in PK pre-dose (0 hour), 1, 2, 4, 6, 8, and 24 hours post-dose. Cycle 1 Day 1 pre-dose (time 0), 1, 2, 4, 6, 8, and 24 hours post-dose.

[17] **Plasma for Abbreviated PK:** Timing for abbreviated PK: at pre-dose (0 hour) and 4 hours post-dose on Cycle 1 Day 15 and Cycles 2-4 Day 1, and pre-dose (0 hour) on Cycle 1 Day 8.

[18] **Cerebral Spinal Fluid (CSF) Collection (optional):** If a patient undergoes a lumber puncture, a sample of CSF should be collected for exploratory analysis of TPX-0005 concentration, if possible. If a CSF sample is collected, a blood sample for PK analysis should also be collected at approximately the same time as the CSF sample.

[19] **CT or MRI Chest/Abdomen/Pelvis Tumor Assessment:** Tumor assessments will include all known or suspected disease sites. For all tumor assessments, the method of assessment that was used at baseline should be the same method used throughout the study. For patients who are without documented disease progression, CT or MRI scans to be done at every 2 cycles (after 7 weeks for the first assessment and then every 2 cycles up to Cycle 18 and then every 3 cycles up to Cycle 36 and then every 4 cycles thereafter until documented progression of disease). Patient responses will be confirmed > 4 weeks later after the initial documentation of response by the Investigator. For patients who have documented disease progression but are continuing TPX-0005 post-RECIST progression, CT or MRI scans are to be done according to local institutional standard of care. Every effort should be made to maintain the assessment scheduling relative to Cycle 1 Day 1 especially if there are dosing cycle interruptions due to toxicities. For all patients, copies of radiologic images must be available for independent central radiology review as determined by TP Therapeutics.

[20] **MRI of Brain:** Gadolinium contrast-enhanced MRI must be used for assessment of CNS lesions at baseline with contingent slices of 1 mm for lesions 5 to 10 mm in size, 5 mm for lesions greater than 10 mm. For the Phase 1b food effect portion of the study, only patients with documented baseline CNS metastases will be assessed by MRI of the brain at every 2 cycles (after 7 weeks for the first assessment and then every 2 cycles up to Cycle 18 and then every 3 cycles up to Cycle 36 and then every 4 cycles thereafter until documented progression of disease). Patient responses will be confirmed > 4 weeks later after the initial documentation of response by the Investigator. For patients who have documented disease progression but are still continuing TPX-0005 post-RECIST progression, MRI scans of the brain are to be done according to local institutional standard of care.

[21] **Bone Scans:** Bone scans (or bone MRI if preferred by Investigator) will be performed at baseline for all patients and repeated every 3 cycles while on study only if evidence of bone metastases is observed at baseline.

[22] **Pharmacodynamic (PD) Assessment:** One optional fresh tumor tissue re-biopsy is recommended between Cycle 1 Day 8 and Cycle 1 Day 15 to assess the PD effect of TPX-0005. The biopsy should obtain enough amount of tumor to generate at least 10 (preferably 15) unbaked, 5-micron thick unstained slides containing FFPE tumor tissue. Specimens will be sent to the TP Therapeutics-designated central laboratories for PD study.

[23] **Concomitant Medications and Non-Drug Supportive Interventions:** All concomitant medications and non-drug supportive interventions should be recorded in the CRF.

Approved v1.0

Table 17 Study Calendar for Phase 2 Dose Expansion Portion of TPX-0005-01

	**Screening**	**Cycle 1 (28-day cycle)**				**Cycle 2 (28-day cycle)**		**Cycle 3 and Cycle 4**	**Cycle 5 and beyond**	**End of Treatment**	**Safety Follow-Up**	**Long-Term Follow-Up**
**Protocol Activity**	**Days -28 to -1**	**Day 1**	**Day 8**	**Day 15**	**Day 21**	**Day 1**	**Day 15**	**Day 1**	**Day 1**			
Visit Window[1]	NA		±1	±1	±1	±2	±2	±2	±2	±7	+7	
Informed Consent[2]	X											
Tumor Molecular Alteration[3]	X											
Tumor Treatment History[4]	X											
Medical History	X	X	X	X	X	X	X	X	X	X	X	
Physical Examination	X	X	X	X	X	X	X	X	X	X	X	
ECOG Performance Status	X	X	X	X	X	X	X	X	X	X	X	
Height	X											
Weight	X	X	X	X	X	X	X	X	X	X	X	
Vital Signs[5]	X	X	X	X	X	X	X	X	X	X	X	
Dispense TPX-0005		X				X		X	X			
TPX-0005 Compliance			X	X	X	X	X	X	X	X		
Laboratory												
Complete Blood Count with Differential[6]	X	X	X	X	X	X	X	X	X	X	X	
Reticulocyte Count (%)	X	X				X		X	X	X	X	
Complete Metabolic Panel[7]	X	X	X	X	X	X	X	X	X	X	X	
Coagulation[8]	X	X		X		X		X	X	X	X	
Endocrine (for male patients only)[9]	X	X				X		X	X	X	X	
Pregnancy Test (serum)[10]	X	X				X		X	X	X	X	
Urine Analysis[11]	X	X	X	X	X	X		X	X	X	X	

<table>
<tr><th></th><th>Screening</th><th colspan="4">Cycle 1
(28-day cycle)</th><th colspan="2">Cycle 2
(28-day cycle)</th><th>Cycle 3
and
Cycle 4</th><th>Cycle 5
and
beyond</th><th>End of
Treatment</th><th>Safety
Follow-Up</th><th>Long-Term
Follow-Up</th></tr>
<tr><th>Protocol Activity</th><th>Days
-28 to -1</th><th>Day 1</th><th>Day 8</th><th>Day 15</th><th>Day 21</th><th>Day 1</th><th>Day 15</th><th>Day 1</th><th>Day 1</th><th></th><th></th><th></th></tr>
<tr><td>Blood Specimens for ccfDNA Enrichment[12]</td><td>X</td><td></td><td></td><td></td><td></td><td></td><td></td><td></td><td></td><td>X</td><td></td><td></td></tr>
<tr><td>Cardiac Safety Monitoring</td><td></td><td></td><td></td><td></td><td></td><td></td><td></td><td></td><td></td><td></td><td></td><td></td></tr>
<tr><td>Triplicate 12-lead ECG[13]</td><td>X</td><td>X</td><td></td><td>X</td><td></td><td>X</td><td></td><td>X</td><td></td><td>X</td><td></td><td></td></tr>
<tr><td>Echocardiogram for LVEF Assessment[14]</td><td>X</td><td></td><td></td><td></td><td></td><td></td><td></td><td>X</td><td>X</td><td>X</td><td></td><td></td></tr>
<tr><td>Cardiac Troponin-I[15]</td><td>X</td><td></td><td></td><td></td><td></td><td></td><td></td><td></td><td></td><td></td><td></td><td></td></tr>
<tr><td>Pharmacokinetics</td><td></td><td></td><td></td><td></td><td></td><td></td><td></td><td></td><td></td><td></td><td></td><td></td></tr>
<tr><td>Plasma for Abbreviated PK[16]</td><td></td><td>X</td><td></td><td>X</td><td></td><td>X</td><td></td><td>X</td><td></td><td></td><td></td><td></td></tr>
<tr><td>CSF (optional)[17]</td><td></td><td colspan="8">X</td><td></td><td></td><td></td></tr>
<tr><td>Tumor Assessment</td><td></td><td></td><td></td><td></td><td></td><td></td><td></td><td></td><td></td><td></td><td></td><td></td></tr>
<tr><td>CT or MRI (Chest/Abdomen/Pelvis)[18]</td><td>X</td><td></td><td></td><td></td><td></td><td></td><td></td><td>X</td><td>X</td><td></td><td></td><td></td></tr>
<tr><td>MRI of Brain[19]</td><td>X</td><td></td><td></td><td></td><td></td><td></td><td></td><td>X</td><td>X</td><td></td><td></td><td></td></tr>
<tr><td>Bone Scan[20]</td><td>X</td><td></td><td></td><td></td><td></td><td></td><td></td><td>X</td><td>X</td><td></td><td></td><td></td></tr>
<tr><td>Other Clinical Assessments</td><td></td><td></td><td></td><td></td><td></td><td></td><td></td><td></td><td></td><td></td><td></td><td></td></tr>
<tr><td>Adverse Events</td><td></td><td>X</td><td>X</td><td>X</td><td>X</td><td>X</td><td>X</td><td>X</td><td>X</td><td>X</td><td>X</td><td></td></tr>
<tr><td>Pharmacodynamic (PD) Assessment[21]</td><td></td><td></td><td colspan="2">X</td><td></td><td></td><td></td><td></td><td></td><td></td><td></td><td></td></tr>
<tr><td>EORTC-QLQ-C30 and EORTC-QLQ-LC-13[22]</td><td>X</td><td>X</td><td></td><td></td><td></td><td>X</td><td></td><td>X</td><td>X</td><td>X</td><td></td><td></td></tr>
<tr><td>Concomitant Medications[23]</td><td>X</td><td>X</td><td>X</td><td>X</td><td>X</td><td>X</td><td>X</td><td>X</td><td>X</td><td>X</td><td>X</td><td></td></tr>
<tr><td>Survival Follow-Up</td><td></td><td></td><td></td><td></td><td></td><td></td><td></td><td></td><td></td><td></td><td></td><td>X</td></tr>
</table>

[1] **Visit Window:** The 28-day screening period starts on the day informed consent is signed by the patient. The safety visit should be within 28 days from the last dose of TPX-0005.

[2] **Informed Consent:** Must be obtained before undergoing any protocol-specific procedures.

[3] **Tumor Molecular Alteration:** Confirmation of *ALK+, ROS1+, or NTRK+* rearrangement (submit molecular pathology report-source document) and submit archival tumor tissue (please refer to Section 5.1).

[4] **Tumor Treatment History:** Documentation of disease progression on prior tyrosine kinase inhibitor (TKIs), duration of treatment with each prior TKI, prior chemotherapy regimens and duration of each chemotherapy regimens, prior immunotherapy regimens and duration of each immunotherapy regimens (if applicable), prior radiation to brain (if brain metastases present, and methods of radiation: whole brain radiation, stereotactic radiosurgery).

[5] **Vital Signs:** Body temperature, blood pressure, heart rate, respiratory rate, pain level (0-10)

[6] **Complete Blood Count with Differential:** White blood cell count, hemoglobin, platelet, absolute neutrophils, absolute lymphocytes, absolute monocytes, absolute eosinophils, and absolute basophils.

[7] **Complete Chemistry Panel:** Sodium, potassium, chloride, bicarbonate, blood urea nitrogen, creatinine, magnesium, phosphorus, calcium, uric acid, total protein, albumin, lactate dehydrogenase, aspartate aminotransferase, alanine aminotransferase, total bilirubin, alkaline phosphatase.

[8] **Coagulation:** Prothrombin time/international normalized ratio, partial thromboplastin time.

[9] **Endocrine (for male patients only):** Total testosterone, sex hormone-binding globulin, follicle-stimulating hormone, luteinizing hormone, prolactin. These laboratories should be drawn between 8 am to 11 am in the morning.

[10] **Serum Pregnancy Test:** For female patients of childbearing potential, a serum pregnancy test, with sensitivity of at least 25 mIU/mL, will be performed on 2 occasions prior to starting study therapy: once at the start of screening and once at the baseline visit, immediately before investigational product administration. Only patients with negative serum pregnancy tests are allowed to enroll. Serum pregnancy test will also be performed at the beginning of each cycle of visit.

[11] **Urine Analysis:** Urinalysis includes dipstick analysis (protein, glucose, ketones, blood, and specific gravity). A microscopic (WBC/HPF, RBC/HPF, and any additional findings) exam need only be performed if the urinalysis result is abnormal. More frequent and complete examinations may be performed at the Investigator's discretion if medically indicated; results should be recorded on unscheduled visit CRFs.

[12] **Blood Specimens for ccfDNA Enrichment (20 mL blood specimen):** Blood will be collected across 2 10 ml Streck Cell-Free DNA Blood Collection Tubes (for ccfDNA analysis) prior to the first dose of TPX-0005 and at EOT for exploratory ccfDNA molecular profiling analyses. Details for handling of these specimens including processing, storage, and shipment will be provided in the Study Manual.

[13] **Triplicate 12-lead ECGs:** At each time point, 3 consecutive 12-lead ECGs will be performed approximately 2 minutes apart to determine mean QTc interval. Triplicate 12-lead ECGs will all be time matched with PK with the exception of during the screening period and at EOT. Triplicate ECGs will be collected as follows:

a) At Screening,
b) Cycle 1 Day 1, at pre-dose (0 hour) and 4 hours post-dose.
c) Cycle 1 Day 15 at pre-dose (0 hour) and 4 hours post-dose
d) Cycles 2-4, Day 1 at pre-dose (0 hour) and 4 hours post-dose

In addition to these time points, ECGs should be repeated as clinically indicated. Additional ECG time points may be included based on the emerging data. Interpretation of the tracing will be made by a central ECG laboratory. Each ECG tracing should be labeled with the study number, patient initials, patient number, date, and kept in the source documents at the study site. Only clinically significant abnormalities will be recorded in the AE CRF page. Clinically significant abnormalities at screening/baseline should be recorded on the relevant medical history/current medical conditions CRF page. Clinically significant findings must be discussed with TP Therapeutics and Medical Monitor prior to enrolling the patient in the study.

[14] **Echocardiography for LVEF Assessments:** Echocardiography to be performed at screening, and after every 3 cycles of treatment (± 7 days) (Cycles 4, 7, 10, 13 and so on), and at the EOT visit (± 7 days).

[15] **Cardiac Troponin-I:** Cardiac troponin-I will be done at baseline then repeated and assessed whenever the ECG morphology demonstrates possible myocardial ischemia or infarction. If troponin CTCAE grade ≥ 3 is seen, this parameter must be repeated twice a week until resolution to CTCAE grade ≤ 1.

[16] **Plasma for Abbreviated PK:** Abbreviated PK will be obtained on Cycle 1 Day 1, Cycle 1 Day 15, and Cycles 2-4 Day 1. Timing for abbreviated PK: at pre-dose (0 hour) and hours post-dose.

[17] **Cerebral Spinal Fluid (CSF) Collection (optional):** If a patient undergoes a lumber puncture, a sample of CSF should be collected for exploratory analysis of TPX-0005 concentration, if possible. If a CSF sample is collected, a blood sample for PK analysis should also be collected at approximately the same time as the CSF sample.

[18] **CT or MRI Chest/Abdomen/Pelvis Tumor Assessment:** Tumor assessments will include all known or suspected disease sites. For all tumor assessments, the method of assessment that was used at baseline should be the same method used throughout the study. For patients who are without documented disease progression, CT or MRI scans to be done every 2 cycles up to Cycle 18 and then every 3 cycles up to Cycle 36 and then every 4 cycles thereafter until documented progression of disease. Patient responses will be confirmed > 4 weeks later after the initial documentation of response by the Investigator. For patients who have documented disease progression but are continuing TPX-0005 post-RECIST progression, CT or MRI scans are to be done according to local institutional standard of care. Every effort should be made to maintain the assessment scheduling relative to Cycle 1 Day 1 especially if there are dosing cycle interruptions due to toxicities. For all patients, copies of radiologic images must be available for independent central radiology review as determined by TP Therapeutics.

[19] **MRI of Brain:** Gadolinium contrast-enhanced MRI must be used for assessment of CNS lesions at baseline with contingent slices of 1 mm for lesions 5 to 10 mm in size, 5 mm for lesions greater than 10 mm. For the Phase 2 dose expansion portion of the study, all patients (with or without documented baseline CNS metastases) will be assessed by MRI of the brain every 2 cycles up to Cycle 18 and then every 3 cycles up to Cycle 36 and then every 4 cycles there after until documented progression of disease. Patient responses will be confirmed > 4 weeks later after the initial documentation of response by the Investigator. For patients who have documented disease progression but are continuing TPX-0005 post-RECIST progression, MRI scans of the brain are to be done according to local institutional standard of care.

[20] **Bone Scans:** Bone scans (or bone MRI if preferred by Investigator) will be performed at baseline for all patients and repeated every 3 cycles while on study only if evidence of bone metastases is observed at baseline.

[21] **Pharmacodynamic (PD) Assessment:** One optional fresh tumor tissue re-biopsy is recommended between Cycle 1 Day 8 and Cycle 1 Day 15 to assess the PD effect of TPX-0005. The biopsy should obtain enough amount of tumor to generate at least 10 (preferably 15) unbaked, 5-micron thick unstained slides containing FFPE tumor tissue. Specimens will be sent to the TP Therapeutics-designated central laboratories for PD study.

[22] **EORTC-QLQ-C30 and QLQ-LC13:** Patients must complete all EORTC-QLQ-C30 and EORTC-QLQ-LC13 self-assessment questionnaires in the clinic at the specified time points prior to dosing. At Cycle 1 Day 1, site staff (e.g., site coordinators) should instruct patients that the assessment should be completed without help from friends or family members and also recommend that this assessment be completed in the morning. All scheduled assessments of the EORTC-QLQ-C30 and EORTC-QLQ-LC13 cannot be taken home and must be completed in the clinic prior to any other study or medical procedure.

[23] **Concomitant Medications and Non-Drug Supportive Interventions:** All concomitant medications and non-drug supportive interventions should be recorded in the CRF.

9 SAFETY ASSESSMENTS

Safety assessments will consist of monitoring and recording adverse events, including serious adverse events, measurement of protocol-specified clinical laboratory assessments and vital signs, and other protocol-specified tests that are deemed critical to the safety evaluation of the study.

Certain types of events require immediate reporting to TP Therapeutics, as outlined in Section 9.3.

9.1 Adverse Events (AEs)

According to the ICH guideline for Good Clinical Practice, an adverse event is any untoward medical occurrence in a clinical investigation subject administered a medicinal product, regardless of causal attribution. An adverse event can therefore be any of the following:

- Any unfavorable and unintended sign (including an abnormal laboratory finding), symptom, or disease temporally associated with the use of a medicinal product, whether or not considered related to the medicinal product
- Any new disease or exacerbation of an existing disease (a worsening in the character, frequency, or severity of a known condition), except as described in Section 9.2.5.9
- Recurrence of an intermittent medical condition (e.g., headache) not present at baseline
- Any deterioration in a laboratory value or other clinical test (e.g., ECG, X-ray) that is associated with symptoms or leads to a change in study treatment or concomitant treatment or discontinuation from study drug
- Adverse events that are related to a protocol-mandated intervention, including those that occur prior to assignment of study treatment (e.g., screening invasive procedures such as biopsies)

9.1.1 Serious Adverse Events (SAE) (Immediately Reportable to TP Therapeutics)

An SAE is any adverse event that meets any of the following criteria:

- Fatal (i.e., the adverse event actually causes or leads to death)
- Life-threatening (i.e., the adverse event, in the view of the Investigator, places the patient at immediate risk of death)
  - ❖ This does not include any adverse event that, had it occurred in a more severe form or was allowed to continue, might have caused death
- Requires or prolongs in-patient hospitalization (see Section 9.2.5.10)
- Results in persistent or significant disability/incapacity (i.e., the adverse event results in substantial disruption of the patient's ability to conduct normal life functions)

- Congenital anomaly/birth defect in a neonate/infant born to a mother exposed to study drug
- Significant medical event in the Investigator's judgment (e.g., may jeopardize the patient or may require medical/surgical intervention to prevent one of the outcomes listed above)

The terms "severe" and "serious" are **not** synonymous. Severity refers to the intensity of an adverse event (e.g., rated as mild, moderate, or severe, or according to NCI CTCAE; see Section 9.2.3); the event itself may be of relatively minor medical significance (such as severe headache without any further findings).

Severity and seriousness need to be independently assessed for each adverse event recorded on the CRF.

Serious adverse events are required to be reported by the Investigator to TP Therapeutics immediately (i.e., no more than 24 hours after learning of the event; see Section 9.3 for reporting instructions).

9.2 Methods and Timing for Capturing and Assessing Safety Parameters

The Investigator is responsible for ensuring that all adverse events are recorded on the Adverse Event CRF and reported to TP Therapeutics.

For each adverse event recorded on the Adverse Event CRF, the Investigator will make an assessment of seriousness (see Section 9.1.1 for seriousness criteria), severity (see Section 9.2.3), and causality (see Section 9.2.4).

9.2.1 Adverse Event Reporting Period

Investigators will seek information on adverse events at each patient contact. All adverse events, whether reported by the patient or noted by study personnel, will be recorded in the patient’s medical record and on the Adverse Event CRF.

After informed consent for the clinical trial has been obtained **but prior to initiation of study drug**, only serious adverse events related to protocol-mandated assessments should be reported.

After initiation of study drug, all adverse events, regardless of relationship to study drug, will be reported until at least 28 days after the last dose of study treatment. Any SAEs occurring any time after the reporting period must be promptly reported if a causal relationship to the study drug is suspected.

Approved v1.0

9.2.2 Eliciting Adverse Event Information

A consistent methodology of non-directive questioning should be adopted for eliciting adverse event information at all patient evaluation time points. Examples of non-directive questions include the following:

"How have you been feeling since your last clinic visit?"
"Have you noticed any new or changed health problems since you were last here?"

9.2.3 Assessment of Severity of Adverse Events

The adverse event severity grading scale for the NCI CTCAE v4.03 will be used for assessing adverse event severity. Table 18 will be used for assessing severity for adverse events that are not specifically listed in the NCI CTCAE.

Table 18 Grading of Severity of Adverse Events

Grade	Severity
1	Mild; asymptomatic or mild symptoms; clinical or diagnostic observations only; or intervention not indicated
2	Moderate; minimal, local, or non-invasive intervention indicated; or limiting age-appropriate instrumental activities of daily living [a]
3	Severe or medically significant, but not immediately life-threatening; hospitalization or prolongation of hospitalization indicated; disabling; or limiting self-care activities of daily living [b, c]
4	Life-threatening consequences or urgent intervention indicated [d]
5	Death related to adverse event [d]

[a] Instrumental activities of daily living refer to preparing meals, shopping for groceries or clothes, using the telephone, managing money, etc.
[b] Examples of self-care activities of daily living include bathing, dressing and undressing, feeding oneself, using the toilet, and taking medications, as performed by patients who are not bedridden.
[c] If an event is assessed as a "significant medical event," it must be reported as a serious adverse event per the definition of serious adverse event.
[d] Grade 4 and 5 events must be reported as serious adverse events per the definition of serious adverse event.

9.2.4 Assessment of Causality of Adverse Events

Investigators should use their knowledge of the patient, the circumstances surrounding the event, and an evaluation of any potential alternative causes to determine whether or not an adverse event is considered to be related to the study drug, indicating "yes" or "no" accordingly. The following guidance should be taken into consideration (see also Table 19):

- Temporal relationship of event onset to the initiation of study drug
- Course of the event, considering especially the effects of dose reduction, discontinuation of study drug, or reintroduction of study drug (as applicable)

Approved v1.0

- Known association of the event with the study drug or with similar treatments
- Known association of the event with the disease under study
- Presence of risk factors in the patient or use of concomitant medications known to increase the occurrence of the event
- Presence of non-treatment-related factors that are known to be associated with the occurrence of the event

Table 19 Causation Attribution Table

Is the adverse event suspected to be caused by the study drug on the basis of facts, evidence, science-based rationales, and clinical judgment?	
YES	There is a plausible temporal relationship between the onset of the adverse event and administration of the study drug, and the adverse event cannot be readily explained by the patient's clinical state, intercurrent illness, or concomitant therapies; and/or the adverse event follows a known pattern of response to the study drug; and/or the adverse event abates or resolves upon discontinuation of the study drug or dose reduction and, if applicable, reappears upon re-challenge.
NO	Evidence exists that the adverse event has an etiology other than the study drug (e.g., preexisting medical condition, underlying disease, intercurrent illness, or concomitant medication); and/or the adverse event has no plausible temporal relationship to administration of the study drug (e.g., cancer diagnosed 2 days after first dose of study drug).

9.2.5 Procedures for Recording Adverse Events

Investigators should use correct medical terminology/concepts when recording adverse events on the Adverse Event CRF. Avoid colloquialisms and abbreviations. Only one adverse event term should be recorded in the event field on the Adverse Event CRF.

9.2.5.1 Diagnosis versus Signs and Symptoms

A diagnosis (if known) should be recorded on the Adverse Event CRF rather than individual signs and symptoms (e.g., record only liver failure or hepatitis rather than jaundice, asterixis, and elevated transaminases). However, if a constellation of signs and/or symptoms cannot be medically characterized as a single diagnosis or syndrome at the time of reporting, each individual event should be recorded on the Adverse Event CRF. If a diagnosis is subsequently established, all previously reported adverse events based on signs and symptoms should be nullified and replaced by one adverse event report based on the single diagnosis, with a starting date that corresponds to the starting date of the first symptom of the eventual diagnosis.

Approved v1.0

9.2.5.2 *Adverse Events That Are Secondary to Other Events*

In general, adverse events that are secondary to other events (e.g., cascade events or clinical sequelae) should be identified by their primary cause, with the exception of severe or serious secondary events. A medically significant secondary adverse event that is separated in time from the initiating event should be recorded as an independent event on the Adverse Event CRF. For example:

- If vomiting results in mild dehydration with no additional treatment in a healthy adult, then only vomiting should be reported on the CRF
- If vomiting results in severe dehydration, then both events should be reported separately on the CRF
- If a severe gastrointestinal hemorrhage leads to renal failure, then both events should be reported separately on the CRF
- If dizziness leads to a fall and consequent fracture, then all 3 events should be reported separately on the CRF
- If neutropenia is accompanied by an infection, then both events should be reported separately on the CRF

All adverse events should be recorded separately on the Adverse Event CRF if it is unclear as to whether the events are associated.

9.2.5.3 *Persistent or Recurrent Adverse Events*

A persistent adverse event is one that extends continuously, without resolution, across cycles. When this occurs, the initial severity (intensity or grade) of the event will be recorded at the time the event is first reported. If a persistent adverse event becomes more or less severe, the initial event should be closed and a new event with the new severity should be recorded on the Adverse Event CRF. Similarly, if the event becomes serious, the initial event should be closed and a new event recorded on the Adverse Event CRF, completing all data fields related to serious adverse events. The serious adverse event should be reported to TP Therapeutics immediately (i.e., no more than 24 hours after learning that the event became serious).

A recurrent adverse event is one that resolves between treatment cycles and subsequently recurs. Each recurrence of an adverse event should be recorded as a separate event on the Adverse Event CRF.

9.2.5.4 *Abnormal Laboratory Values*

Not every laboratory abnormality qualifies as an adverse event. A laboratory test result must be reported as an adverse event if it meets any of the following criteria:

- Accompanied by clinical symptoms
- Results in a change in study treatment (e.g., dosage modification, treatment interruption, or treatment discontinuation)

Approved v1.0

- Results in a medical intervention (e.g., potassium supplementation for hypokalemia) or a change in concomitant therapy
- Results in more frequent follow-up assessments or further diagnostic investigation
- Clinically significant in the Investigator's judgment

It is the Investigator's responsibility to review all laboratory findings. Medical and scientific judgment should be exercised in deciding whether an isolated laboratory abnormality should be classified as an adverse event.

If a clinically significant laboratory abnormality is a sign of a disease or syndrome (e.g., increased alkaline phosphatase and bilirubin at 5 × ULN associated with cholestasis), only the diagnosis (i.e., cholestasis) should be recorded on the Adverse Event CRF.

If a clinically significant laboratory abnormality is not a sign of a disease or syndrome, the abnormality itself should be recorded on the Adverse Event CRF, along with a descriptor indicating if the test result is above or below the normal range (e.g., "elevated potassium," as opposed to "abnormal potassium"). If the laboratory abnormality can be characterized by a precise clinical term per standard definitions, the clinical term should be recorded as the adverse event. For example, an elevated serum potassium level of 7.0 mEq/L should be recorded as "hyperkalemia."

Observations of the same clinically significant laboratory abnormality from visit to visit should not be repeatedly recorded on the Adverse Event CRF, unless the etiology changes. The initial severity of the event should be recorded, and the severity or seriousness should be updated as described in Section 9.2.5.3.

9.2.5.5 Abnormal Vital Sign Values

Not every vital sign abnormality qualifies as an adverse event. A vital sign result must be reported as an adverse event if it meets any of the following criteria:

- Accompanied by clinical symptoms
- Results in a change in study treatment (e.g., dosage modification, treatment interruption, or treatment discontinuation)
- Results in a medical intervention or a change in concomitant therapy
- Clinically significant in the Investigator's judgment

It is the Investigator's responsibility to review all vital sign findings. Medical and scientific judgment should be exercised in deciding whether an isolated vital sign abnormality should be classified as an adverse event.

If a clinically significant vital sign abnormality is a sign of a disease or syndrome (e.g., high blood pressure), only the diagnosis (i.e., hypertension) should be recorded on the Adverse Event CRF.

Approved v1.0

Observations of the same clinically significant vital sign abnormality from visit to visit should not be repeatedly recorded on the Adverse Event CRF, unless the etiology changes. The initial severity of the event should be recorded, and the severity or seriousness should be updated as described in Section 9.2.5.3.

9.2.5.6 Abnormal Liver Function Tests

Abnormal values in aspartate transaminase (AST) and/or alanine transaminase (ALT) levels concurrent with abnormal elevations in total bilirubin level that meet the criteria outlined below in the absence of other causes of liver injury are considered potential cases of drug-induced liver injury (potential Hy's Law cases) and should always be considered important medical events.

The threshold of laboratory abnormalities for a potential case of drug-induced liver injury depends on the patient's individual baseline values and underlying conditions. Patients who present with the following laboratory abnormalities should be evaluated further to definitively determine the etiology of the abnormal laboratory values:

- Patients with AST or ALT and total bilirubin baseline values within the normal range who subsequently present with AST or ALT values > 3× ULN concurrent with a total bilirubin value > 2 × ULN with no evidence of hemolysis and an alkaline phosphatase value ≤ 2 × ULN or not available.
- For patients with preexisting ALT **OR** AST **OR** total bilirubin values above the ULN, the following threshold values should be used in the definition mentioned above:
    - ❖ For patients with preexisting AST or ALT baseline values above the normal range, AST or ALT value ≥ 2 times the baseline values and ≥ 3 × ULN, or ≥ 8 × ULN (whichever is smaller).
- Concurrent with
    - ❖ For patients with preexisting values of total bilirubin above the normal range: Total bilirubin increased from baseline by an amount of ≥ 1 × ULN or if the value reaches ≥ 3 × ULN (whichever is smaller).

The patient should return to the investigational site and be evaluated as soon as possible, preferably within 48 hours from awareness of the abnormal results. This evaluation should include laboratory tests, detailed history and physical assessment. The possibility of hepatic neoplasia (primary or secondary) should be considered. In addition to repeating measurements of AST and ALT, laboratory tests should include albumin, creatine kinase, total bilirubin, direct and indirect bilirubin, gamma-glutamyl transferase, prothrombin time (PT)/international normalized ratio (INR), and alkaline phosphatase. A detailed history, including relevant information, such as review of ethanol, acetaminophen, recreational drug and supplement consumption, family history, occupational exposure, sexual history, travel history, history of contact with a jaundiced person, surgery, blood transfusion, history of liver or allergic disease, and work exposure, should be collected. Further testing for acute hepatitis A, B, or C infection and liver imaging (e.g., biliary tract) may be warranted. All cases confirmed on repeat testing as meeting the laboratory criteria defined above, with no other cause for liver function test (LFT) abnormalities identified

Approved v1.0

at the time should be considered potential Hy's Law cases irrespective of availability of all the results of the investigations performed to determine etiology of the abnormal LFTs. Such potential Hy's Law cases should be reported as SAEs.

9.2.5.7 Deaths

For the purposes of this study, deaths due to disease progression will not be considered a serious adverse event.

All deaths, regardless of relationship to study drug, must be recorded on the Death CRF and on the Adverse Event CRF only if it is considered related to an adverse event.

Death should be considered an outcome and not a distinct event. The event or condition that caused or contributed to the fatal outcome should be recorded as the single medical concept on the Adverse Event CRF. Generally, only one such event should be reported. The term "**sudden death**" should be used only for the occurrence of an abrupt and unexpected death due to presumed cardiac causes in a patient with or without preexisting heart disease, within 1 hour after the onset of acute symptoms or, in the case of an unwitnessed death, within 24 hours after the patient was last seen alive and stable. If the cause of death is unknown and cannot be ascertained at the time of reporting, "**unexplained death**" should be recorded on the Adverse Event CRF. If the cause of death later becomes available (e.g., after autopsy), "unexplained death" should be replaced by the established cause of death.

9.2.5.8 Preexisting Medical Conditions

A preexisting medical condition is one that is present at the screening visit for this study. Such conditions should be recorded on the Medical History CRF.

A preexisting medical condition should be recorded as an adverse event only if the frequency, severity, or character of the condition worsens during the study. When recording such events on the Adverse Event CRF, it is important to convey the concept that the preexisting condition has changed by including applicable descriptors (e.g., "worsening headaches").

9.2.5.9 Lack of Efficacy or Worsening of the Malignancy under Study.

Events that are clearly consistent with the expected pattern of progression of the underlying disease should not be recorded as adverse events. These data will be captured as efficacy assessment data only. In most cases, the expected pattern of progression will be reflected as objective radiographic progression according to RECIST criteria. In rare cases, the determination of disease progression will be based predominantly on symptomatic deterioration. However, every effort should be made to document progression through use of objective criteria. If there is any uncertainty as to whether an event is due to disease progression, it should be reported as an adverse event.

Approved v1.0

9.2.5.10 Hospitalization or Prolonged Hospitalization

Any adverse event that results in hospitalization (i.e., in-patient admission to a hospital) or prolonged hospitalization should be documented and reported as a serious adverse event, except as outlined below.

The following hospitalization scenarios are not considered to be adverse events:

- Hospitalization for respite care or social admissions (e.g., lack of housing, economic inadequacy, family circumstances)
- Hospitalization solely for coordination of care, including hospice arrangements
- Hospitalization due solely to progression of the underlying cancer
- Hospitalization that was necessary solely because of patient requirement for outpatient care outside of normal outpatient clinic operating hours
- Hospitalization for same day surgeries (as outpatient/same day/ambulatory procedures).
- Planned hospitalization required by the protocol (e.g., for study drug administration or insertion of access device for study drug administration)
- Hospitalization for a preexisting condition, provided that the hospitalization was planned prior to the study or was scheduled during the study when elective surgery became necessary because of the expected normal progression of the disease and the patient has not experienced an adverse event

9.2.5.11 Exposure During Pregnancy

For investigational products and for marketed products, an exposure during pregnancy occurs if:

- A female becomes, or is found to be, pregnant either while receiving or having been exposed (e.g., because of treatment or environmental exposure) to the investigational product; or the female becomes, or is found to be pregnant after discontinuing and/or being exposed to the investigational product. An example of environmental exposure would be a case involving direct contact with TPX-0005 product in a pregnant women (e.g., a nurse reports that she is pregnant and has been exposed to chemotherapeutic products).
- A male patient has been exposed (e.g., because of treatment or environmental exposure) to the investigational product prior to or around the time of conception and/or is exposed during his partner's pregnancy.

If a study patient or study patient's partner becomes or is found to be pregnant during the study patient's treatment with the investigational product, the investigator must submit this information to the TP Therapeutics Drug Safety Unit on a Serious Adverse Event Report Form and Exposure in Utero (EIU) Supplemental Form, regardless of whether an SAE has occurred. In addition, the investigator must submit information regarding environmental exposure to a TP Therapeutics product in a pregnant woman (e.g., a patient reports that she is pregnant and has been exposed to

a cytotoxic product by inhalation or spillage) using the EIU Form. This must be done irrespective of whether an AE has occurred and within 24 hours of awareness of the exposure. The information submitted should include the anticipated date of delivery (see below for information related to termination of pregnancy).

Follow-up is conducted to obtain general information on the pregnancy and its outcome for all EIU reports with an unknown outcome. The investigator will follow the pregnancy until completion or until pregnancy termination and notify TP Therapeutics of the outcome as a follow-up to the initial EIU Form. In the case of a live birth, the structural integrity of the neonate can be assessed at the time of birth. In the event of a termination, the reason(s) for the termination should be specified and, if clinically possible, the structural integrity of the terminated fetus should be assessed by gross visual inspection (unless pre-procedure test findings are conclusive for a congenital anomaly and the findings are reported).

If the outcome of the pregnancy meets the criteria for an SAE (i.e., ectopic pregnancy, spontaneous abortion, intrauterine fetal demise, neonatal death, or congenital anomaly [in a live born, a terminated fetus, an intrauterine fetal demise or a neonatal death]), the investigator should follow the procedures for reporting SAEs.

Additional information about pregnancy outcomes that are reported as SAEs follows:

- Spontaneous abortion includes miscarriage and missed abortion;
- Neonatal deaths that occur within 1 month of birth should be reported, without regard to causality, as SAEs. In addition, infant deaths after 1 month should be reported as SAEs when the Investigator assesses the infant death as related or possibly related to exposure to investigational product.

Additional information regarding the exposure during pregnancy may be requested by the Investigator. Further follow-up of birth outcomes will be handled on a case-by-case basis (e.g., follow-up on preterm infants to identify developmental delays). In the case of paternal exposure, the Investigator will provide the study patient with the Pregnant Partner Release of Information Form to deliver to his partner. The Investigator must document in the source documents that the patient was given the Pregnant Partner Release of Information Form to provide to his partner.

9.2.5.12 Occupational Exposure

An occupational exposure occurs when during the performance of job duties, a person (whether a healthcare professional or otherwise) gets in unplanned direct contact with the product, which may or may not lead to the occurrence of an adverse event.

An occupational exposure is reported to safety within 24 hours of Investigator’s awareness, using the SAE Report form, regardless of whether there is an associated AE/SAE. Since the information does not pertain to a patient enrolled in the study, the information is not reported on a CRF, however a copy of the completed SAE Report form is maintained in the study master file.

Approved v1.0

9.2.5.13 Adverse Events Associated with an Overdose or Incorrect Administration of Study Drug

An overdose is the accidental or intentional use of a drug in an amount higher than the dose being studied. An overdose or incorrect administration of study treatment is not itself an adverse event, but it may result in an adverse event. All adverse events associated with an overdose or incorrect administration of study drug should be recorded on the Adverse Event CRF. If the associated adverse event fulfills serious criteria, the event should be reported to TP Therapeutics immediately (i.e., no more than 24 hours after learning of the event).

9.3 Immediate Reporting Requirements from Investigator to TP Therapeutics

Certain events require immediate reporting to allow TP Therapeutics to take appropriate measures to address potential new risks in a clinical trial. The Investigator must report such events to TP Therapeutics immediately; under no circumstances should reporting take place more than 24 hours after the Investigator learns of the event. The following is a list of events that the Investigator must report to TP Therapeutics within 24 hours after learning of the event, regardless of relationship to study drug:

- Serious adverse events
- Pregnancies

The Investigator must report new significant follow-up information for these events to TP Therapeutics immediately (i.e., no more than 24 hours after becoming aware of the information). New significant information includes the following:

- New signs or symptoms or a change in the diagnosis
- Significant new diagnostic test results
- Change in causality based on new information
- Change in the event’s outcome, including recovery
- Additional narrative information on the clinical course of the event

Investigators must also comply with local requirements for reporting serious adverse events to the local health authority and IRB/EC.

9.3.1 SAE Reporting

After informed consent has been obtained but prior to initiation of study drug, only serious adverse events related to protocol-mandated procedures should be reported.

After initiation of study drug, serious adverse events will be reported until at least 28 days after the last dose of study drug.

Investigators should record all case details that can be gathered immediately (i.e., within 24 hours after learning of the event) on the SAE report form and submit the report via fax or email (please refer to the Study Manual).

Approved v1.0

9.3.2 Reporting Requirements for Pregnancies

9.3.2.1 Pregnancies in Female Patients

Female patients or partners of male patients of childbearing potential will be instructed to immediately inform the Investigator if they become pregnant during the study or within 3 months after the last dose of study drug. A Pregnancy report form should be completed by the Investigator no more than 24 hours after learning of the pregnancy and submitted using the same process as for SAEs.

The Investigator should discontinue study drug and counsel the patient or partner of a male patient, discussing the risks of the pregnancy and the possible effects on the fetus. Monitoring should continue until conclusion of the pregnancy. Any serious adverse events associated with the pregnancy (e.g., an event in the fetus, an event in the mother during or after the pregnancy, or a congenital anomaly/birth defect in the child) should be reported and submitted within 24 hours.

9.3.2.2 Congenital Anomalies/Birth Defects and Abortions

Any congenital anomaly/birth defect in a child born to a female patient or partner of a male patient exposed to study drug should be classified as a serious adverse event and reported as such. Any spontaneous abortion or miscarriage should be reported in the same fashion (therapeutic abortions are excluded from expedited reporting and would be captured as pregnancy outcome information).

9.4 Follow-Up of Patients after Adverse Events

9.4.1 Investigator Follow-Up

The Investigator should follow each adverse event until the event has resolved to baseline grade or better, the event is assessed as stable by the Investigator, the patient is lost to follow-up, or the patient withdraws consent. Every effort should be made to follow all serious adverse events considered to be related to study drug or trial-related procedures until a final outcome can be reported.

During the study period, resolution of adverse events (with dates) should be documented on the Adverse Event CRF and in the patient's medical record to facilitate source data verification.

All pregnancies reported during the study should be followed until pregnancy outcome.

9.4.2 TP Therapeutics Follow-Up

For serious adverse events and pregnancies, TP Therapeutics or a designee may follow-up by telephone, fax, electronic mail, and/or a monitoring visit to obtain additional case details and outcome information (e.g., from hospital discharge summaries, consultant reports, autopsy reports) in order to perform an independent medical assessment of the reported case.

Approved v1.0

9.5 Post-Study Adverse Events

TP Therapeutics should be notified if the Investigator becomes aware of any serious adverse event that occurs after the end of the adverse event reporting period (defined as 28 days after the last dose of study drug), if the event is believed to be related to the study drug.

The Investigator should report these events directly to TP Therapeutics or its designee using the SAE report form.

9.6 Expedited Reporting to Health Authorities, Investigators, Institutional Review Boards, and Ethics Committees

TP Therapeutics will promptly evaluate all serious adverse events against cumulative product experience to identify and expeditiously communicate possible new safety findings to Investigators, IRBs, ECs, and applicable health authorities based on applicable legislation.

To determine reporting requirements for single adverse event cases, TP Therapeutics will assess the expectedness of these events using the Investigator's Brochure.

TP Therapeutics will compare the severity of each event and the cumulative event frequency reported for the study with the severity and frequency reported in the applicable reference document.

Reporting requirements will also be based on the Investigator's assessment of causality and seriousness, with allowance for upgrading by TP Therapeutics as needed.

Approved v1.0

10 PATIENT END OF TREATMENT

A patient may be discontinued from study treatment at any time if the patient, the Investigator, or TP Therapeutics feels that it is not in the patient's best interest to continue on study. The following is a list of possible reasons for early discontinuation of study treatment:

- Disease progression (unless there is reasonable evidence of clinical benefit to justify continuation on treatment – which must be previously discussed with TP Therapeutics)
  - ❖ Patients should be highly encouraged to remain on study treatment until there is BICR-confirmed radiographic progression
  - ❖ Patient may be allowed to continue TPX-0005 beyond RECIST-defined progression if, in the clinical judgment of the Investigator, the patient continues to derive clinical benefit from TPX-0005 (with approval from TP Therapeutics)
  - ❖ There is no limit to the number of cycles of treatment with TPX-0005
- Any adverse event that cannot be adequately managed with dose modifications, including dose interruption > 28 days (unless there is reasonable evidence of clinical benefit to justify continuation on the protocol – which must be previously discussed with TP Therapeutics)
- Protocol violation requiring discontinuation of study treatment
- Patient is not compliant with study procedures
- Lost to follow-up
- Patient withdrawal of consent for further treatment
- TP Therapeutics' early termination of study. Reasons for terminating the study may include, but are not limited to, the following:
  - ❖ All enrolled patients have discontinued study treatment
  - ❖ The incidence or severity of adverse events in this or other studies indicates a potential health hazard to patients
  - ❖ Patient enrollment is unsatisfactory

Data to be collected for the end-of-treatment visit are described in Section 8.4.

Patients will be followed for at least 28 calendar days after the last dose of study drug. If a patient is withdrawn from treatment due to an adverse event, the patient will be followed until the adverse event has resolved or stabilized as per Section 9.4.

11 PROTOCOL VIOLATIONS

A protocol violation occurs when the patient or Investigator fails to adhere to significant protocol requirements affecting the inclusion, exclusion, patient safety, or primary endpoint criteria. Protocol violations for this study include, but are not limited to, the following:

- Failure to meet inclusion/exclusion criteria (no waivers will be granted to meet the eligibility criteria)
- Dose modifications (e.g., wrong treatment or incorrect dose) that are not within the protocol specifications
- Use of a prohibited concomitant medication
- Any other deviation that presents significant risk or safety concerns to the patient (e.g., pregnancy on study)

Failure to comply with Good Clinical Practice (GCP) guidelines will also result in a protocol violation. TP Therapeutics, in consultation with the Investigator, will determine if a protocol violation should result in withdrawal of a patient.

Approved v1.0

12 DATA MONITORING COMMITTEE

An independent third-party Data Monitoring Committee (DMC) will be established to ensure the overall integrity and conduct of the Phase 2 Dose Expansion portion of the study.

The DMC will review the progress of the study and cumulative safety and efficacy data on a periodic basis (e.g., a minimum of 2 review meetings per year). In addition to the formal meetings, listings of serious adverse events will be provided to the DMC on a quarterly basis.

Following each review meeting, the DMC will recommend to TP Therapeutics whether to continue the trial unchanged, modify the conduct of the study, terminate a study cohort early, or terminate the study early with the final decision by TP Therapeutics. Rules for early termination, modifications, and/or continuation of the study, as well as how those recommendations will be made to TP Therapeutics or Health Authorities will be outlined in a separate DMC Charter.

The DMC will be composed of 3 external members (2 physicians and 1 biostatistician) not associated with the conduct of the study. TP Therapeutics' biostatistician and data management team (or their designee will prepare and provide study data to the DMC). Complete details regarding the composition and governance of the DMC will be outlined in the DMC Charter. Periodic adverse event data review will also be performed by designated members of TP Therapeutics' study team or their designee. Any safety issues of concern identified by the study team will be promptly reported to the DMC as described in the DMC Charter.

Approved v1.0

13 STATISTICAL METHODS AND CONSIDERATIONS

Detailed methodology for summary and statistical analyses of the data collected in this trial will be documented in a Statistical Analysis Plan (SAP), which will be maintained by TP Therapeutics or their designee. This document may modify the initial analysis plans outlined in the protocol; however, any major modifications of the primary endpoint and/or its analysis will also be reflected in a protocol amendment.

13.1 Analysis Populations (Sets)

13.1.1 Full Analysis Set

The Full Analysis Set (FAS) consists of all patients who receive at least 1 full or partial dose of TPX-0005. Patients will be classified according to the assigned treatment. Patients who were screened but never started treatment will be listed. Screening failures will not be included in any of the summary tables.

13.1.2 Safety Analysis Set

The safety analysis set includes all enrolled patients who receive at least 1 dose of TPX-0005. Patients will be classified according to assigned treatment (dose level for Phase 1 and study cohort for Phase 2). Each patient will be classified into and analyzed consistently within one (and only one) treatment group.

13.1.3 Per Protocol Analysis Set (Phase 1)

- Evaluable for MTD: All enrolled patients who receive at least 75% of the planned TPX-0005 doses in the first cycle. Patients who receive less than 75% of the planned TPX-0005 doses in the first cycle due to DLT are also considered evaluable for MTD.
- Evaluable for Response: All enrolled patients who receive study treatment, have a baseline tumor assessment, and have at least 1 on-study tumor assessment will be considered evaluable for response. Patients who are treated and removed from study prior to on-study tumor assessments because of disease progression will be considered evaluable for efficacy and counted as failures.
- Evaluable for CNS Metastasis Response: A subset of the evaluable for response analysis set with CNS metastases at study entry.

13.1.4 Per Protocol Analysis Set (Phase 2)

- Evaluable for Response: For each subpopulation in Phase 2, all enrolled patients who receive study treatment, have a baseline tumor assessment, and have at least 1 on-study tumor assessment will be considered evaluable for response. Patients who are treated and removed from study prior to on-study tumor assessment because of disease progression will be considered evaluable for efficacy and counted as failures.

Approved v1.0

- Evaluable for CNS Metastasis Response: A subset of the evaluable for response patients with CNS metastases at study entry.

13.1.5 PK Parameter Analysis Set

The PK parameter analysis population is defined as all enrolled patients who receive at least 1 dose of study medication and have sufficient information to estimate at least 1 of the PK parameters of interest.

13.2 Efficacy Analyses

13.2.1 Analysis of Primary Endpoint

13.2.1.1 Analysis of Primary Endpoint (Phase 1)

The primary endpoint for the Phase 1 study is to determine the MTD and RP2D of TPX-0005.

13.2.1.2 Analysis of Primary Endpoint (Phase 2)

The primary endpoint for the Phase 2 study is ORR, as assessed by BICR using RECIST version 1.1, in each patient population expansion cohort of solid tumors that harbor an *ALK*, *ROS1*, *NTRK1*, *NTRK2*, or *NTRK3* gene rearrangement.

The ORR will be defined as the proportion of patients with confirmed complete response (CR) or partial response (PR); a confirmed response is a response that persists on repeat-imaging ≥ 4 weeks after initial documentation of response. Such patients with a confirmed objective response (CR or PR) will be referred to as responders. Non-responders will include the following:

- Patients without a confirmed objective response
- Patients without a post-baseline tumor assessment
- Patients who receive at least 1 dose of TPX-0005 and who discontinue for any reason prior to undergoing 1 post-baseline response evaluation

The ORR will be reported as the proportion of responders along with the corresponding 2-sided 95% Clopper-Pearson exact confidence interval (CI).

13.2.2 Analysis of Secondary Endpoints

13.2.2.1 Analysis of Secondary Endpoints (Phase 1)

The secondary endpoint for the phase 1 study is ORR, as assessed by BICR using RECIST version 1.1, in each patient population expansion cohort of solid tumors that harbor an *ALK*, *ROS1*, *NTRK1*, *NTRK2*, or *NTRK3* gene rearrangement.

Approved v1.0

The ORR will be defined as the proportion of patients with confirmed CR or PR; a confirmed response is a response that persists on repeat-imaging ≥ 4 weeks after initial documentation of response. Such patients with a confirmed objective response (CR or PR) will be referred to as responders. Non-responders will include the following:

- Patients without a confirmed objective response
- Patients without a post-baseline tumor assessment
- Patients who receive at least 1 dose of TPX-0005 and who discontinue for any reason prior to undergoing 1 post-baseline response evaluation

The ORR will be reported as the proportion of responders along with the corresponding 2-sided 95% Clopper-Pearson exact CI.

13.2.2.2 Analysis of Secondary Endpoints (Phase 2)

13.2.2.2.1 Duration of Response (DOR)

The DOR will be defined from the first date of objective response (either CR or PR) to first documentation of radiographic disease progression, as assessed by RECIST version 1.1. The DOR will be censored at the last tumor assessment date for patients without disease progression who have not died within 28 days of the last dose of study treatment.

The DOR will only be calculated for the subgroup of patients with a confirmed objective tumor response. The DOR will be summarized in the populations of patients with a confirmed CR or PR using the Kaplan-Meier method and will be displayed graphically where appropriate. The median event time (if appropriate) and 2-sided 95% CI for the median will be provided. Since the number of patients with a confirmed CR or PR may be small, the use of the Kaplan-Meier method may be limited and the DOR may be summarized using descriptive statistics.

13.2.2.2.2 Time to Response (TTR)

The TTR will be defined as the time from the first dose of TPX-0005 to the first documentation of objective response (either CR or PR), as assessed by RECIST version 1.1.

Kaplan-Meier methods will be used to estimate median TTR.

13.2.2.2.3 Clinical Benefit Rate (CBR)

The CBR will be defined as the proportion of patients with CR, PR, or stable disease (SD) at 6 months after the first dose of TPX-0005, as assessed by RECIST version 1.1.

Approved v1.0

13.2.2.2.4 Intracranial Objective Response Rate (IC-ORR)

Intracranial ORR is defined as the percent of patients with intracranial objective response relative to patients on the per protocol analysis set, evaluable for CNS metastasis response subset, and will be provided along with the corresponding 95% CI.

Intracranial ORR will be calculated pooling the patients from all of the dose escalation cohorts in the per protocol analysis set, evaluable for CNS metastasis response subset.

13.2.2.2.5 CNS Progression-Free Survival (CNS-PFS)

CNS progression-free survival (CNS-PFS) will be defined as the time from the first dose of TPX-0005 to first evidence of radiographic CNS disease progression according to RECIST version 1.1 or death due to any cause (whichever occurs first) + 1 day. Censoring rules will apply as per below.

13.2.2.2.6 Progression-Free Survival

Progression-free survival (PFS) will be defined as the time from the first dose of TPX-0005 to first documentation of radiographic disease progression or death due to any cause (whichever occurs first) + 1 day.

PFS data for patients without progression or death will be censored on the date of the last tumor assessment (or, if no tumor assessment was performed after the baseline visit, at the date of first dose of study drug + 1 day).

Estimates of the PFS curves using the Kaplan-Meier method will be presented. This method will be applied to derive the median event time and a CI for the median as well as the 1-year and 18-month survival probabilities with related CIs. The CIs will be 2-sided, have a stated coverage probability of 95%, and be calculated using normal approximation methods.

13.2.2.2.7 Overall Survival (OS)

OS is defined as the time from randomization to the date of death due to any cause. For patients still alive at the time of analysis, the OS time will be censored on the last date the patients were known to be alive.

Estimates of the OS curves using the Kaplan-Meier method will be presented. This method will be applied to derive the median event time and a CI for the median as well as the 1-year and 18-month survival probabilities with related CIs. The CIs will be 2-sided, have a stated coverage probability of 95%, and be calculated using normal approximation methods.

Approved v1.0

13.2.3 Analysis of Planned Pooled Analysis

13.2.3.1 Analysis of Efficacy by Specific Gene Rearrangement (ALK, ROS1, NTRK1, NTRK2, NTRK3)

The ORR, DOR, TTR, CBR, IC-ORR, CNS-PFS, PFS, and OS will be analyzed according to the specific gene rearrangement (*ALK*, *ROS1*, *NTRK1*, *NTRK2*, or *NTRK3*) and within each gene rearrangement by TKI-naïve or TKI-refractory.

13.2.3.2 Analysis of Efficacy by Measurable CNS Lesions

IC-ORR and CNS-PFS will be analyzed in all patients with measurable CNS lesions as defined by RECIST version 1.1.

13.2.4 Analysis of Planned Subset Analysis

13.2.4.1 Analysis of Efficacy and Safety by Specific NTRK Rearrangement (NTRK1, NTRK2, NTRK3)

The ORR, DOR, TTR, CBR, IC-ORR, CNS-PFS, PFS, and OS will be analyzed according to the specific *NTRK* gene rearrangement (*NTRK1*, *NTRK2*, or *NTRK3*) and within each gene rearrangement by TKI-naïve or TKI-refractory.

13.2.4.2 Analysis of Efficacy and Safety by Specific Fusion Partner Within Each Specific Gene Rearrangement (ALK, ROS1, NTRK1, NTRK2, NTRK3)

The ORR, DOR, TTR, CBR, IC-ORR, CNS-PFS, PFS, and OS will be analyzed according to the specific fusion partner (i.e., TPM3, CD74) and within each fusion partner by TKI-naïve or TKI-refractory.

13.3 Statistical Analysis Plan (SAP) and Sample Size Justification

13.3.1 Phase 1 Dose Escalation and Food Effect Cohorts

13.3.1.1 Phase 1a Dose Escalation

For the Dose Escalation phase of the study, cohorts of 3-6 evaluable patients will be required. The total number of patients will depend upon the number of dose escalations necessary.

Currently there are 7 dose escalations levels planned (40 mg, 80 mg, 160 mg, 240 mg, 320 mg, 400 mg, 500 mg). If MTD is not reached at the highest dose level, then assuming 6 patients at each of the 6 lower dose levels and a total of 12 patients in the highest dose level, a maximum of 48 patients are anticipated to be enrolled. However, if MTD is not reached at the highest dose

Approved v1.0

level (500 mg), then there is a possibility to increase enrollment to doses higher than 500 mg with approval from the Investigators and the TP Therapeutics.

13.3.1.2 Phase 1b Food Effect Study.

The food effect study will commence at the dose level where 1 patient experience a treatment-related CTCAE grade 2 AE OR a patient experiences PR as per Investigator. Six patients will be enrolled at that dose level and subsequent dose levels. An additional 12 patients will enroll into the food effect study at the RP2D. Thus the minimum number of patients to enroll into the food effect study will be 12 patients.

13.3.2 *ROS1*-rearranged NSCLC Expansion Cohorts

The *ROS1*-rearranged NSCLC Expansion cohorts will enroll primarily *ROS1*+ NSCLC patients who are either ROS1i-naïve or ROS1i-refractory.

There are 3 *ROS1*+ NSCLC expansion cohorts: one ROS1i-naïve *ROS1*+ NSCLC expansion cohort and 2 ROS1i-refractory *ROS1*+ NSCLC expansion cohorts. Among the 2 ROS1i-refractory *ROS1*+ NSCLC expansion cohorts, one allows only crizotinib-refractory *ROS1*+ NSCLC patients and the other expansion cohort allows *ROS1*+ NSCLC patients refractory to 2 or more ROS1i.

For the 3 *ROS1*+ NSCLC expansion cohorts, the goal of the primary analysis of objective response will be to estimate the ORR and their exact 95% CIs (Clopper-Pearson).

13.3.2.1 ROS1-rearranged NSCLC Expansion Cohort (ROS1i-naïve [EXP-1])

Table 20 below shows possible estimated ORR and 95% CIs for different level of responses in populations of 50 patients.

Table 20 Estimated ORRs and Related 95% Confidence Intervals in 50 patients

# Responses in 50 Patients	Overall Response Rate (95% CI)
20/50	40% (26.4 – 54.8)
**23/50**	**46% (31.8 – 60.7)**
25/50	50% (35.5 – 64.5)
**26/50**	**52% (37.4 – 66.3)**
30/50	60% (45.2 – 73.6)
**33/50**	**66% (51.2 – 78.8)**
35/50	70% (55.4 – 82.1)

Fifty eligible patients will be enrolled into the ROS1i-naïve *ROS1*+ NSCLC patient expansion cohort. For the ROS1i-naïve expansion cohort, if the ORR is 50% or less, then it is assumed that TPX-0005 is not effective in these ROS1i-naïve *ROS1*+ NSCLC patients. Using Table 20, if

33 patients out of 50 patients have a confirmed objective response (ORR = 66%; 95% CI: 51.2 – 78.8) where the lower limit of the 95% CI is > 50%, TPX-0005 is considered to be efficacious in this patient expansion cohort.

13.3.2.2 ROS1-rearranged NSCLC Expansion Cohort (crizotinib-refractory only [EXP-2])

Fifty eligible patients will be enrolled into the crizotinib-refractory only *ROS1+* NSCLC patient expansion cohort. For the crizotinib-refractory *ROS1+* NSCLC expansion cohort, if the ORR is 35% or less, then it is assumed that TPX-0005 is not effective in these crizotinib-refractory *ROS1+* NSCLC patients. Using Table 20 above, if 26 patients out of 50 patients have a confirmed objective response (ORR = 52%; 95% CI: 37.4 – 66.3) where the lower limit of the 95% CI is > 35%, TPX-0005 is considered to be efficacious in this crizotinib-refractory *ROS1+* NSCLC patient expansion cohort.

13.3.2.3 ROS1-rearranged NSCLC Expansion Cohort (ROS1i-refractory, > 1 prior ROS1i, or 1 non-crizotinib-refractory [EXP-3])

Fifty eligible patients will be enrolled into the > 1 prior ROS1i-refractory *ROS1+* NSCLC patient expansion cohort. For the > 1 prior ROS1i-refractory or 1 prior non-crizotinib ROS1i-refractory *ROS1+* NSCLC expansion cohort, if the ORR is 30% or less, then it is assumed that TPX-0005 is not effective in these > 1 prior ROS1i-refractory or 1 prior non-crizotinib ROS1i-refractory *ROS1+* NSCLC patients. Using Table 20 above, if 23 patients out of 50 patients have a confirmed objective response (ORR = 46%; 95% CI: 31.8 – 60.7) where the lower limit of the 95% CI is > 30%, TPX-0005 is considered to be efficacious in this patient expansion cohort. Additionally, there is a potential in the future to increase accrual to this ROS1i-refractory cohort analyzing 2 prior or 3 prior ROS1i specifically to confirm the observed clinical efficacy.

13.3.3 *ALK*-rearranged NSCLC Expansion Cohorts

The *ALK*-rearranged NSCLC Expansion cohorts will enroll *ALK+* NSCLC who are either ALKi-naïve or ALKi-refractory. There are 5 *ALK+* NSCLC expansion cohorts: ALKi-naïve, 1 prior ALKi-refractory, 2 prior ALKi-refractory, 3 prior ALKi-refractory, and 4 or more prior ALKi-refractory. For the ALKi-naïve expansion cohort, the patients have to be treatment-naïve for their advanced metastatic or recurrent *ALK+* NSCLC.

For 4 of the 5 *ALK+* NSCLC expansion cohorts (ALKI-naïve, 1 prior ALKi, 2 prior ALKi, 3 prior ALKi), the goal of the primary analysis of objective response will be to estimate the ORR and their exact 95% CIs (Clopper-Pearson). Each of these 4 expansion cohorts will enroll 40 eligible patients. For the > 3 prior ALKi expansion cohort, please see Section 13.3.3.3.

Approved v1.0

13.3.3.1 ALK-rearranged NSCLC Expansion Cohort (Treatment naïve [EXP-4])

Table 21 below shows the estimated ORR and 95% CIs for different level of responses in a population of 40 patients.

Table 21 Estimated ORR and Related 95% Confidence Interval (Clopper-Pearson) in 40 Patients

# Responses in 40 patients	Overall response rate (95% CI)
16/40	40% (24.9 – 56.7)
18/40	45% (29.3 – 61.5)
**19/40**	**47.5% (31.5 – 63.9)**
20/40	50% (33.8 – 66.2)
24/40	60% (43.3 – 751)
25/40	62.5% (45.8 – 77.3)
26/40	65% (48.3 – 79.3)
**28/40**	**70% (53.5 – 83.4)**
30/40	75% (58.8 – 87.3)

Forty eligible patients will be enrolled into the treatment-naïve *ALK*+ NSCLC patient expansion cohort. For the ALKi-naïve *ALK*+ NSCLC expansion cohort, if the ORR is 50% or less, then it is assumed that TPX-0005 is not effective in these ALKi-naïve *ALK*+ NSCLC patients. Using Table 21 above, if 28 patients out of 40 patients have a confirmed objective response (ORR = 70%; 95% CI: 53.5 – 83.4) where the lower limit of the 95% CI is > 50%, TPX-0005 is considered to be of clinical benefit in this patient expansion cohort. Enrollment will continue until all 40 patients are confirmed to harbor *ALK* rearrangement by the TP Therapeutics's chosen central laboratory.

[redacted]

Approved v1.0

[redacted]

13.3.3.3 ALK-rearranged NSCLC Expansion Cohort (ALKi-refractory: 4 or more ALKi [EXP-8])

In the 4 or more prior ALKI *ALK*+ NSCLC expansion cohort, patients will be enrolled under a Simon's 2-stage design (Simon 1989) to determine whether TPX-0005 has sufficient anticancer activity to warrant further development of that specific patient population.

For this 4 or more ALKi-refractory *ALK*+ NSCLC expansion cohort, a true response rate of 10% or less is considered insufficient to warrant further study, whereas a true response rate of 25% or more is considered worthy of further study. The number of patients evaluated in each stage and the minimum number of responders needed to continue to the next stage were determined based on the Minimax method with 80% power and 1-sided $\alpha = 0.05$.

Based on the above design considerations, initially 22 ALKi-refractory (> 3 ALKi) eligible patients will be accrued to the first stage. If ≤ 2 patients achieve an objective response, then enrollment in that expansion cohort will be terminated. If at least 3 patients achieve an objective response among the first 22 patients, then an additional 18 eligible patients will be accrued to the second stage (total 40 patients). If ≤ 7 patients achieve an objective response out of the total eligible 40 patients accrued, then no further evaluation of TPX-0005 in this > 3 ALKi-refractory *ALK*+ NSCLC cohort is warranted.

13.3.4 *ALK*-rearranged Non-NSCLC, *ROS1*-rearranged Non-NSCLC, and *NTRK*-rearranged Solid Malignancy Expansion Cohorts

Given the relative rarity of patients with either *ALK*+ non-NSCLC solid malignancies, *ROS1*+ non-NSCLC solid malignancies, or *NTRK*+ solid malignancies in general, these patients will be enrolled into 2 expansion cohorts: one TKI-naïve and one TKI-refractory. The ORR among these patients in each expansion cohort will be analyzed together using Simon's 2-stage design (Simon, 1989) to determine whether TPX-0005 has sufficient anticancer activity to warrant further development of that specific patient population. There is potential to expand the enrollment of patients harboring a specific rearrangement in a specific organ type if clinical efficacy is observed in either expansion cohort.

Approved v1.0

13.3.4.1 ALK-rearranged Non-NSCLC, ROS1-rearranged Non-NSCLC, and NTRK-rearranged Solid Malignancy Expansion Cohort (TKI-naïve [EXP-9])

For this TKI-naïve expansion cohort, a true response rate of 20% or less is considered insufficient to warrant further study, whereas a true response rate of 50% or more is considered worthy of further study. The number of patients evaluated in each stage and the minimum number of responders needed to continue to the next stage were determined based on the Minimax method with 90% power and 1-sided $\alpha = 0.025$.

Based on the above design considerations, 11 eligible TKI-naïve patients will be accrued to the first stage. If ≤ 2 patients achieve a confirmed objective response, then enrollment in that expansion cohort will be terminated. If at least 3 patients achieve a confirmed objective response among the first 11 patients, then an additional 15 eligible patients will be accrued to the second stage (total 26 patients). If ≤ 9 patients achieve a confirmed objective response out of the total 26 patients accrued, then no further evaluation of TPX-0005 in this TKI-naïve cohort is warranted.

13.3.4.2 ALK-rearranged Non-NSCLC, ROS1-rearranged Non-NSCLC, and NTRK-rearranged Solid Malignancy Expansion Cohort (TKI-refractory [EXP-10])

For this TKI-refractory expansion cohort, a true response rate of 10% or less is considered insufficient to warrant further study, whereas a true response rate of 35% or more is considered worthy of further study. The number of patients evaluated in each stage and the minimum number of responders needed to continue to the next stage were determined based on the Minimax method with 90% power and 1-sided $\alpha = 0.025$.

Based on the above design considerations, 14 eligible TKI-refractory patients will be accrued to the first stage. If ≤ 1 patient achieves a confirmed objective response, then enrollment in that expansion cohort will be terminated. If at least 2 patients achieve a confirmed objective response among the first 14 patients, then an additional 14 eligible patients will be accrued to the second stage (total 28 patients). If ≤ 6 patients achieve a confirmed objective response out of the total 28 patients accrued, then no further evaluation of TPX-0005 in this TKI-refractory cohort is warranted.

13.4 Safety Analyses

13.4.1 Adverse Events

Adverse events will be graded according to the NCI CTCAE v4.03 and coded to preferred term and system organ class (SOC) using the most recent version of MedDRA.

All AEs reported during the AE reporting period (inclusive of the 28-day post last dose of study drug period) will be considered as TEAEs.

Approved v1.0

For each patient population expansion cohort, incidence rates will be summarized with frequency and percentage by MedDRA SOC and preferred term, with all patients treated in that particular expansion cohort as the denominator, unless otherwise specified. In addition, AE incidence rates will also be summarized by severity and relationship to study drug. Treatment-related AEs are those judged by the Investigator to be at least possibly related to the study drug. Patients with multiple occurrences of events will only be counted once at the maximum severity to study drug for each preferred team, SOC, and overall. Deaths that occur within 28 days after the last dose of study drug are defined as on-study deaths.

Summary tables and individual patient listings will be prepared as per the Statistical Analysis Plan.

13.4.2 Clinical Laboratory Results

Normal ranges will be used to identify values that are outside the normal ranges and abnormal laboratory results will be graded according to the NCI CTCAE v4.03 Descriptive statistics will be provided for each test result and for the change from baseline by visit.

A shift summary of baseline grade by maximum post-baseline CTCAE grade will be presented, as appropriate. For each laboratory parameter, the baseline laboratory value will be defined as the last laboratory value collected on or prior to the date of the first dose of study drug.

Patients who develop toxicities of CTCAE grade ≥ 3 will be summarized. Laboratory test results not having CTCAE grade will also be summarized. Parameters that have criteria available for both low and high values (e.g., hypercalcemia versus hypocalcaemia) will be summarized for both criteria. Patients will only be counted once for each criterion.

13.4.3 Vital Signs

Each vital sign (body temperature, blood pressure [systolic and diastolic], respiration rate, and heart rate) will be summarized and presented by study visit. Patients with clinically significant abnormalities as compared to baseline will be listed.

13.4.4 Concomitant Medications/Treatment

All medications and/or treatments received during the protocol treatment period will be considered as concomitant medications and/or concomitant treatments and will be coded by the World Health Organization Drug Dictionary; patients who received concomitant medications and/or treatments will be listed.

13.4.5 ECG

The analysis of ECG results will be based on Safety Population patients with baseline and on-treatment ECG data. ECG collected prior to the first day of dosing will be considered the baseline ECG.

Approved v1.0

ECG measurements (an average of the triplicate measurements) will be used for the statistical analysis and all data presentations. Any data obtained from ECGs repeated for safety reasons after the nominal time points will not be averaged along with the preceding triplicates. Interval measurements from repeated ECGs will be included in the outlier analysis as individual values obtained at unscheduled time points.

QT intervals will be corrected for heart rate (QTc) using standard correction factors (i.e., Bazett's, Fridericia's, and possibly a study-specific factor). The adequacy of the correction method will be assessed graphically (plots of QT and QTc versus RR) and supplementary transformations may be considered, as appropriate. Data will be summarized and listed for QT, PR, QRS, QTcF, and QTcB by cycle, day, and dose. Individual QTc (all evaluated corrections) intervals will be listed by time and dose. Descriptive statistics (n, mean, median, standard deviation, minimum, and maximum) will be used to summarize the absolute QTc value and changes from baseline in QTc after treatment by cycle, day, dose, and by time point. For each patient and by treatment, the maximum change from baseline will be calculated as well as the maximum post-baseline value across time points. Outlier analysis of the QTc data will be conducted and summarized as follows:

- The number of patients with maximum change from baseline in QTc (< 30, 30-60, and > 60 msec)
- The number of patients with maximum post-dose (post-baseline) QTc (< 450, 450 to < 480, 480 to ≤ 500, and > 500 msec).

Shift tables will be provided for baseline vs worst on-study QTc (one or more correction method will be used) using maximum CTCAE Grade (V4.03), as well as tables of ECG abnormality at baseline (yes, no, not done: [n, %]). Patients experiencing clinically relevant morphological ECG changes will be summarized (including frequency and percentage). The effect of drug concentrations on QTc change from baseline will be explored graphically. Additional concentration-QTc analyses may be performed. Data may be pooled with other study results and/or explored further with PK/PD models.

13.4.6 Left Ventricular Ejection Fraction (LVEF)

For patients with MUGA scans or echocardiograms, individual LVEF proportion (%) and its changes from baseline will be summarized by time point. The number of patients and the percentage whose maximum decrease from baseline in LVEF is ≥ 20% will be calculated.

13.5 Population Pharmacokinetic (popPK)

Population PK analysis will utilize patient covariates (age, gender, ethnicity, smoking status, body surface area, body mass index) to identify sub-populations where possible. The relationship of exposure to TPX-0005 (and its potential metabolites) to measures of efficacy and adverse events will be modeled to the greatest extent possible.

Descriptive statistics, including mean and median values will be summarized. Concentrations below the lower limit of quantification of the assay will be excluded or assigned a numeric value

Approved v1.0

based on the lower reporting limit of the assay. Plasma levels of TPX-0005 (and its potential metabolites) will be listed by patient and summarized descriptively (mean, standard deviation, percent coefficient of variation, minimum, maximum). Individual and mean concentration-versus-time plots will be presented on both linear and logarithmic scales where possible. Non-compartmental, compartmental, and population analysis methods will be utilized, as applicable.

Samples may also be used for metabolite identification, but the results of such analyses will be used for exploratory purposes and will not be included in the clinical study report. Selected samples may also be analyzed for concentrations of concurrent medications, and these data may be used to assess drug-drug interactions.

13.5.1 Japan Sub-Study

TPX-0005 (and its potential metabolites) concentrations will be measured using a validated LC-MS/MS method; pharmacokinetic parameters will be calculated for each patient using non-compartmental methods. Plasma samples that are left over after the main analyses may be used for qualitative metabolite identification.

13.6 Patient-Reported Outcomes (PROs)

Patient-reported outcomes (PROs) will be administered during the Phase 2 Does Expansion portion of the study.

PROs is an umbrella term referring to all outcomes and symptoms that are directly reported by the patient. PROs have become a significant endpoint when evaluating effectiveness of treatments in clinical trials.

Patients will complete the self-administered quality of life (QOL) instruments listed below in Table 23 every 4 weeks: The patient should complete the questionnaires at the scheduled clinic visit at baseline (screening) and then every 4 weeks. The patient should also complete a questionnaire at progression and their treatment discontinuation visit. If any scheduled PRO assessment is not completed, the reason for non-completion should be recorded.

The following PROs will be administered: European Organization for Research and Treatment of Cancer (EORTC) QLQ-C30 and QLQ-LC-13 (for patients with either *ALK*-, *ROS1*-, or *NTRK*-rearranged NSCLC).

Approved v1.0

Table 23 Patient-Reported Outcome QOL Instruments

Instruments[1]	Definition
EORTC QLQ-C30 (Appendix 5)	Core Quality of Life Questionnaire:<br>• Comprises 30 questions assessing global QOL, functioning, and symptoms of both multi-item and single-item measures<br>• Developed to assess the quality of life of cancer patients (Aaronson 1993)<br>• Has been translated and validated into 81 languages and is used in more than 3,000 studies worldwide (http://groups.eortc.be/qol/eortc-qlq-c30)
EORTC QLQ-LC13 (Appendix 6)	Lung Cancer Module (NSCLC patients only)<br>• Comprises 13 questions assessing lung cancer-specific symptoms (Bergman 1994)

1 Developed by European Organization for Research and Treatment of Cancer (EORTC)

13.6.1 Administration of PROs

PROs will only be administered during the Phase 2 Dose Expansion portion of the study.

Questionnaires will be administered. The patient should complete the questionnaires at the scheduled clinic visit at screening, and every 4 weeks relative to the first dose of multiple dosing, discontinuation, and progression as specified in the Study Calendars (Table 17).

PROs will be filled out prior to any other site activities and encounters with physician. The patients will be instructed to complete the PRO independently. The site will have a designated quiet space for patients to use when completing the assessments. Each center should allocate responsibility for PRO assessment to a specified individual (e.g., a research nurse).

It is important that the value and relevance of QOL data are explained carefully to participating patients so that they are motivated to comply with data collection. The appointed individual should also stress that the information is confidential. Therefore, if the patient has any medical problems she should discuss them with the doctor or research nurse separately from their QoL assessment.

The instructions for completion of questionnaires are:

- It must be completed before any investigations or discussions about the status of the patient's disease with the clinic staff.
- The patient must complete it themselves without any intervention from family, friends, center staff, etc.
- The only exception to this is if the patient is blind or illiterate. In this case the questionnaire may be read to the patient verbatim; however, the reader must not aid in the interpretation of questions or in the selection of answers.

- Only one answer to every question should be checked.
- Study center personnel should not review the responses to the questionnaire with the patient or with any other center staff.

Following completion, the appointed individual may quickly scan the questionnaire visually for completeness and should confirm verbally with the patient that the questionnaire has been completed fully.

13.6.2 EORTC-QLQ-C30

The EORTC-QLQ-C30 was developed by the EORTC Quality of Life Group 1993. It consists of 30 items and measures cancer patients' functioning (QoL) and symptoms (Aaronson 1993).

Note that the term item refers to a question. For example, "Do you have any trouble doing strenuous activities, like carrying a heavy shopping bag or a suitcase?" refers to question 1 of the EORTC-QLQ-C30 questionnaire.

The following variables are to be calculated from the QLQ-C30 questionnaires:

- Global health status: Global health status
- Function scales: Physical functioning, Role functioning, Emotional functioning, Cognitive functioning, and Social functioning
- Symptoms: Fatigue, Nausea and vomiting, and Pain
- Single items: Dyspnea, Insomnia, Appetite loss, Constipation, Diarrhea, and Financial difficulties

Each of these variables is described as a domain in the EORTC scoring manual. The Global health status, Functional scales, and Symptom variables are multi-item domains (consist of more than 1 question). The single-item domains consist of only 1 question.

There are 28 questions whose options are: Not at all (score is 1), A little (score is 2), Quite a bit (score is 3), and Very much (score is 4). Question 29 and 30, which are for measuring global health status, are scored from 1 (very poor) to 7 (excellent).

Each of the multi-item domains includes a different set of items; no item occurs in more than one domain. Note that:

- A computed high score for a functional domain represents a high/healthy level of functioning, whereas a high raw score for a functional item represents a low/poor level of functioning;
- A computed/raw high score for the global health status/QOL represents a high QOL;
- A computed/raw high score for a symptom domain/item represents a high level of symptomatology/problems.

Note: No items are to be reversed for scoring.

The principle for scoring these scales is the same in all cases:

1. Estimate the average of the items that contribute to the scale; this is the raw score (RS).
2. Use a linear transformation to standardize the raw score, so that scores range from 0 to 100; a higher score represents a higher ("better") level of functioning, or a higher ("worse") level of symptoms.

13.6.2.1 Technical Summary

In practical items, if terms I1, I2… In are included in a scale, the procedure is as follows:

Raw score: calculate the raw score, Raw score = RS = (I1+ I2+…+In)/n

13.6.2.2 Linear Transformation

Apply the linear transformation to 0-100 to obtain the score S.

Functional scales: S = {1 – ((RS – 1) /range)}*100

Symptom scales/items: S = {(RS – 1) /range}*100

Global health score /QOL: S = {(RS – 1) /range}*100

Range is difference between the maximum possible value of RS and minimum possible value. The QLQ-C30 has been designed so that all items in any scale take the same range of values.

Examples:

Emotional functioning, Raw score = (Q21+ Q22+ Q23+ Q24)/4

EF score = {1 – ((RS – 1)/3)}*100

13.6.2.3 Missing Items for QLQ-C30

If at least half of the items (i.e., 3 of 6 items, or 3 of 5 items) from the domain have been answered, assume that the missing items have values equal to the average of those items that are present for that respondent. This is equivalent to the raw score being taken as the mean of the non-missing item values. As a result, none of the single-item measures can be imputed.

Example:

Emotional Functioning if Q23 is missing (i.e., 3 items are not missing)

Raw/Score = (Q21+Q22+Q24)/3
EF Score = {1 – ((RS – 1)/3)}*100

13.6.3 EORTC-QLQ-LC13

The EORTC-QLQ-LC13 is a complementary module measuring lung cancer-associated symptoms and side effects from conventional chemotherapy and radiotherapy (Bergman 1994).

Approved v1.0

The EORTC-QLQ-LC13 incorporates 1 multi-item domain to assess dyspnea, and a series of single items assessing pain, coughing, sore mouth, dysphagia, peripheral neuropathy, alopecia, and hemoptysis.

There are 12 questions whose options are: Not at all (score is 1), A little (score is 2), Quite a bit (score is 3), and Very much (score is 4).

A low score for a domain represents a high/healthy level.

Scoring procedure: The scoring approach for the EORTC-QLQ-LC13 is identical in principle to that for the symptom domains/single items of the EORTC-QLQ-C30.

Note: Imputation will be performed for EORTC-QLQ-LC13 for Dyspnea only if question 5 is not missing.

e.g. RS for dyspnea will be Q3 + Q5 if Q4 is missing

RS for dyspnea will be Q4 + Q5 if Q3 is missing

If Q5 is missing then dyspnea will not be derived, however Q3 and Q4 will be summarized

separately.

13.6.4 QOL Compliance Rates

Summary measures of overall compliance and compliance over time will be derived for EORTC-QLQ-C30 and EORTC-QLQ-LC13 scores. These will be based upon:

- Received forms = number of EORTC-QLQ-C30 and EORTC-QLQ-LC13 forms received back plus the number not received back where the reason was 'Subject too heavily affected by symptoms of disease under investigation'
- Expected forms = number of patients still on treatment at the specified assessment time excluding patients in countries with no available translation
- Evaluable forms = EORTC-QLQ-C30 and EORTC-QLQ-LC13 forms with at least 1 domain that can be determined

Thus the overall compliance rate is defined as number of patients with an evaluable baseline and at least 1 evaluable follow-up form (as defined above), divided by the number of patients expected to have completed at least a baseline EORTC-QLQ-C30 and EORTC-QLQ-LC13 form.

Compliance over time will be calculated separately for each visit, including baseline, as the number of patients with an evaluable form at the time point (as defined above), divided by number of patients still expected to complete forms. Similarly, the evaluability rate over time will be calculated separately for each visit, including baseline, as the number of evaluable forms (per definition above), divided by the number of received forms.

13.6.5 PRO Endpoints

All QOL instruments will be reported on a 0 to 100 scale.

For the EORTC-QLQ-C30 and EORTC-QLQ-LC13, respectively, each domain and item will be linearly transformed to standardize the raw score to a range from 0 to 100, with 100 representing the best possible function/QOL and highest symptom severity.

The following analysis variables will be summarized for all QOL instruments, domains, and single items:

- Mean change from baseline, by cycle and across cycle
- Proportion of patients with improved, stable, and worsened outcomes, by cycle and across cycle

A 10-point change from baseline in an item or domain is established to be clinically meaningful (Osoba 1998). Hence, for functioning domains and global QOL, a patient will be deemed:

- Improved, if the change from baseline is a 10-points or greater increase
- Worsened, if the change from baseline is 10-point or greater decrement
- Stable otherwise

For analysis of symptom domains and single items, the opposite is true:

- Improved, if the change from baseline is a 10-points or greater decrement
- Worsened, if the change from baseline is 10-point or greater increase
- Stable otherwise

13.6.6 PRO Analyses

For each patient population expansion cohort and using the PRO-evaluable population, the number and percentage of patients of the total number eligible at each cycle who completed each questionnaire will be summarized per time point. The questionnaire domains and items will be scored according to each questionnaire's respective scoring algorithm. Analyses will include the mean change from baseline scores across cycles and the proportion of patients with improved, stable, or worsened outcomes.

13.6.6.1 Change from Baseline Scores

Descriptive statistics (including 95% CIs) of raw and change from baseline scores will be performed by cycle and across cycle for the EORTC-QLQ-C30 and EORTC-QLQ-LC13 items. In addition, results will be depicted graphically.

13.6.6.2 Proportion of Patients Improved, Remained Stable, or Worsened

The proportion of patients who improved, remained stable, or worsened will be reported by cycle and across cycle.

Approved v1.0

14 DATA COLLECTION, RETENTION AND MONITORING

14.1 Data Collection Instruments

The Investigator will prepare and maintain adequate and accurate source documents designed to record all observations and other pertinent data for each patient treated with the study drug.

Study personnel at each site will enter data from source documents corresponding to a patient's visit into the protocol-specific electronic Case Report Form (CRF) when the information corresponding to that visit is available. Patients will not be identified by name in the study database or on any study documents to be collected by TP Therapeutics (or designee), but will be identified by a site number, patient number, and/or patient initials, as per local regulations.

If a correction is required for an CRF, the time and date stamps track the person entering or updating CRF data and creates an electronic audit trail.

The Investigator is responsible for reviewing all information collected on patients enrolled in this study for completeness and accuracy. A copy of the CRF will remain at the Investigator's site at the completion of the study.

14.2 Data Management Procedures

The data will be entered into a validated database. The TP Therapeutics- or designee-designated data management group will be responsible for data processing, in accordance with procedural documentation. Database lock will occur once quality assurance procedures have been completed.

All procedures for the handling and analysis of data will be conducted using good computing practices meeting FDA guidelines for the handling and analysis of data for clinical trials.

14.3 Data Quality Control and Reporting

After data have been entered into the study database, a system of computerized data validation checks will be implemented and applied to the database on a regular basis. Queries are entered, tracked, and resolved through the electronic data capture (EDC) system directly. The study database will be updated in accordance with the resolved queries. All changes to the study database will be documented.

14.4 Data Archival

The database is safeguarded against unauthorized access by established security procedures; appropriate backup copies of the database and related software files will be maintained. Databases are backed up by the database administrator in conjunction with any updates or changes to the database.

Approved v1.0

At critical junctures of the protocol (e.g., production of interim and final reports), data for analysis are locked and cleaned per established procedures.

14.5 Availability and Retention of Investigational Records

To enable evaluations and/or audits from regulatory authorities or TP Therapeutics, the Investigator agrees to keep records, including the identity of all participating patients (sufficient information to link records [e.g., CRFs and hospital records]), all original signed informed consent forms, copies of all CRFs, safety reporting forms, source documents, and detailed records of treatment disposition, and adequate documentation of relevant correspondence (e.g., letters, meeting minutes, telephone calls reports). The records should be retained by the Investigator according to ICH, local regulations, or as specified in the Clinical Trial Agreement, whichever is longer; but at a minimum, all study documentation must be retained for 2 years after the last marketing application approval in an ICH region or after at least 2 years have elapsed since formal discontinuation of clinical development of TPX-0005.

If the Investigator becomes unable for any reason to continue to retain study records for the required period (e.g., retirement, relocation), then TP Therapeutics should be prospectively notified. The study records must be transferred to a designee acceptable to TP Therapeutics, such as another Investigator, another institution, or to TP Therapeutics itself. The Investigator must obtain TP Therapeutics' written permission before disposing of any records, even if retention requirements have been met.

14.6 Monitoring

Monitoring visits will be conducted by authorized representatives of TP Therapeutics according to the US Code of Federal Regulations (CFR) Title 21 Parts 50, 56, and 312 and ICH Guidelines for GCP (E6)[3], as well as any applicable local, regional, and country laws. By signing this protocol, the Investigator grants permission to TP Therapeutics (or designee), the US FDA, other regulatory agencies, IRB/ECs, and respective national or local health authorities to conduct on-site monitoring and/or auditing of all appropriate study documentation.

14.7 Patient Confidentiality

In order to maintain patient confidentiality, only a site number, patient number, and/or patient initials will identify all study patients on CRFs and other documentation submitted to TP Therapeutics. Additional patient confidentiality issues (if applicable) are covered in the Clinical Trial Agreement.

[3] ICH E6(R1) Tripartite Guideline. Guideline for Good Clinical Practice. 10 June 1996.

Approved v1.0

15 ADMINISTRATIVE, ETHICAL, REGULATORY CONSIDERATIONS

This study will be conducted in accordance with the US Food and Drug Administration (FDA) regulations, the International Council for Harmonisation (ICH) E6 Guideline[3] for Good Clinical Practice (GCP), and applicable local, state, and federal laws, as well as other applicable country laws.

To maintain confidentiality, all laboratory specimens, evaluation forms, reports, and other records will be identified by a coded number and initials only. All study records will be kept in a locked file cabinet and code sheets linking a patient's name to a patient identification number will be stored separately in another locked file cabinet. Clinical information will not be released without written permission of the patient, except as necessary for inspection by the FDA or other regulatory agencies, IRB/ECs, and respective national or local health authorities. The Investigator must also comply with all applicable privacy regulations (e.g., Health Insurance Portability and Accountability Act of 1996).

15.1 Protocol Amendments

Any amendment to the protocol will be written by TP Therapeutics. Protocol amendments cannot be implemented without prior written IRB/EC approval except as necessary to eliminate immediate safety hazards to patients. A protocol amendment intended to eliminate an apparent immediate hazard to patients may be implemented immediately, provided the IRB/ECs are notified within 5 working days.

15.2 Institutional Review Boards/Ethic Committees

Informed consent will be obtained in accordance with ICH GCP, US Code of Federal Regulations for Protection of Human Subjects (21 CFR 50.25[a, b], CFR 50.27, and CFR Part 56, Subpart A), the Health Insurance Portability and Accountability Act (if applicable), and local regulations.

Either the local institution's or TP Therapeutics' designated central laboratory molecular testing consent form can be used to perform molecular testing in order to determine enrollment eligibility.

For all patients, TP Therapeutics' clinical trial informed consent form will be used.

TP Therapeutics or its designee must review and approve any proposed deviations from the sample ICFs or any alternate consent forms proposed by the site before IRB/EC submission. Patients must be re-consented to the most current version of the consent forms during their participation in the study. The final IRB/EC-approved consent forms must be provided to TP Therapeutics for regulatory purposes.

The consent forms must be signed by the patient or the patient's legal representative before his/her participation in the study. The case history for each patient shall document the informed consent process and that written informed consent was obtained prior to participation in the

Approved v1.0

study. A copy of each signed consent form must be provided to the patient or the patient's legal representative. If applicable, it will be provided in a certified translation of the local language.

All signed and dated consent forms must remain in each patient's study file and must be available for verification by study monitors at any time.

The informed consent form should be revised whenever there are changes to procedures outlined in the informed consent or when new information becomes available that may affect the willingness of the patient to participate.

For any updated or revised consent forms, the case history for each patient shall document the informed consent process and that written informed consent was obtained for the updated/revised consent form for continued participation in the study. The final revised IRB/EC-approved informed consent form must be provided to TP Therapeutics for regulatory purposes.

15.3 Reporting of Safety Issues and Serious Breaches of the Protocol or ICH GCP

In the event of any prohibition or restriction imposed (i.e., clinical hold) by an applicable Competent Authority in any area of the World, or if the Investigator is aware of any new information that might influence the evaluation of the benefits and risks of the IP, TP Therapeutics should be informed immediately.

In addition, the Investigator will inform TP Therapeutics immediately of any urgent safety measures taken by the Investigator to protect the study patients against any immediate hazard, and of any serious breaches of this protocol or of ICH GCP of which the Investigator becomes aware.

15.4 TP Therapeutics Discontinuation Criteria

Premature termination of this study may occur because of a regulatory authority decision, change in opinion of the IRB/EC, drug safety problems, or at the discretion of TP Therapeutics. In addition, TP Therapeutics retains the right to discontinue development of TPX-0005 at any time.

If a study is prematurely terminated or discontinued, then TP Therapeutics will promptly notify the Investigator. After notification, the Investigator must notify the respective IRB/EC, and contact all participating patients and the hospital pharmacy (if applicable) within a 4-week time period. As directed by TP Therapeutics, all study materials must be collected and all CRFs completed to the greatest extent possible.

Approved v1.0

15.5 Post-Trial Access

A patient will not be eligible to receive study drug after the end of the study if any of the following conditions are met:

- The study drug is commercially marketed in the patient's country and is reasonably accessible to the patient (e.g., is covered by the patient's insurance or wouldn't otherwise create a financial hardship for the patient)
- TP Therapeutics has discontinued development of the study drug or data suggest that the study drug is not effective
- TP Therapeutics has reasonable safety concerns regarding the study drug
- Provision of study drug is not permitted under the laws and regulations of the patient's country

15.6 Publications

Publication of study results is discussed in the Clinical Trial Agreement. Details regarding production of manuscripts and conference presentations will adhere to the International Committee of Medical Journal Editors (ICMJE) requirements for authorship and contributorship.

http://www.icmje.org/recommendations/browse/roles-and-responsibilities/defining-the-roleof-authors-and-contributors.html

Approved v1.0

16 REFERENCES

Aaronson NK, Ahmedzai S, Bergman B, Bullinger M, Cull A, Duez NJ, et al. The European Organization for Research and Treatment of Cancer QLQ-C30: a quality-of-life instrument for use in international clinical trials in oncology. J Natl Cancer Inst. 1993;85:365-76.

Aisner DL, Nguyen TT, Paskulin DD, Le AT, Haney J, Schulte N, et al. ROS1 and ALK fusions in colorectal cancer with evidence of intratumoral heterogeneity for molecular drivers. Mol Cancer Res. 2014;12:111-18.

Ardini E, Bosotti R, Borgia AL, De Ponti C, Somaschini A, Cammarota R, et al. The TPM3-NTRK1 rearrangement is a recurring event in colorectal carcinoma and is associated with tumor sensitivity to TRKA kinase inhibition. Mol Oncol. 2014;8:1495-507.

Awad MM, Katayama R, McTigue M, Liu W, Deng YL, Brooun A, et al. Acquired resistance to crizotinib from a mutation in CD74-ROS1. N Engl J Med. 2013;368:2395-401.

Balko JM, Schwarz LJ, Luo N, Estrada MV, Giltnane JM, Dávila-González D, et al. Triple-negative breast cancers with amplification of JAK2 at the 9p24 locus demonstrate JAK2-specific dependence. Sci Transl Med. 2016;8:334ra53.

Barbacid M. Structural and functional properties of the TRK family of neurotrophin receptors. Ann N Y Acad Sci. 1995;766:442–58.

Barlesi F, Mazieres J, Merlio JP, Debieuvre D, Mosser J, Lena H, et al. Routine molecular profiling of patients with advanced non-small-cell lung cancer: results of a 1-year nationwide programme of the French Cooperative Thoracic Intergroup (IFCT). Lancet. 2016;387:1415-26

Bergethon K, Shaw AT, Ou SH, Katayama R, Lovly CM, McDonald NT, et al. ROS1 rearrangements define a unique molecular class of lung cancers. J Clin Oncol. 2012;30:863-70.

Bergman B, Aaronson NK, Ahmedzai S, Kaasa S, Sullivan M. The EORTC QLQ-LC13: a modular supplement to the EORTC Core Quality of Life Questionnaire (QLQC30) for use in lung cancer clinical trials. EORTC Study Group on Quality of Life. Eur J Cancer. 1994;30A:635-42.

Blume-Jensen P, Hunter T. Oncogenic kinase signalling. Nature. 2001;411:355-65.

Brenca M, Rossi S, Polano M, Gasparotto D, Zanatta L, Racanelli D, et al. Transcriptome sequencing identifies ETV6-NTRK3 as a gene fusion involved in GIST. J Pathol. 2016;238:543-9.

Bresler SC, Weiser DA, Huwe PJ, Park JH, Krytska K, Ryles H, et al. ALK mutations confer differential oncogenic activation and sensitivity to ALK inhibition therapy in neuroblastoma. Cancer Cell. 2014;26:682-94.

Bromann PA, Korkaya H, Courtneidge SA. The interplay between Src family kinases and receptor tyrosine kinases. Oncogene. 2004;23:7957-68.

Approved v1.0

Buchert M, Burns CJ, Ernst M. Targeting JAK kinase in solid tumors: emerging opportunities and challenges. Oncogene. 2016;35:939-51.

Castellana B, Aasen T, Moreno-Bueno G, Dunn SE, Ramón y Cajal S. Interplay between YB-1 and IL-6 promotes the metastatic phenotype in breast cancer cells. Oncotarget. 2015;6:38239-56.

Charest A, Lane K, McMahon K, Park J, Preisinger E, Conroy H, et al. Fusion of FIG to the receptor tyrosine kinase ROS in a glioblastoma with an interstitial del(6)(q21q21). Gene Chromosomes Cancer. 2003;37:58-71.

Costa DB, Shaw AT, Ou SH, Solomon BJ, Riely GJ, Ahn MJ, et al. Clinical Experience With Crizotinib in Patients With Advanced ALK-Rearranged Non-Small-Cell Lung Cancer and Brain Metastases. J Clin Oncol. 2015;33:1881-8.

Créancier L, Vandenberghe I, Gomes B, Dejean C, Blanchet JC, Meilleroux J, et al. Chromosomal rearrangements involving the NTRK1 gene in colorectal carcinoma. Cancer Lett. 2015;365:107-11. doi: 10.1016/j.canlet.2015.05.013.

Crescenzo R, Abate F2, Lasorsa E, Tabbo' F, Gaudiano M, Chiesa N, et al. Convergent mutations and kinase fusions lead to oncogenic STAT3 activation in anaplastic large cell lymphoma. Cancer Cell. 2015;27(4):516-32

Crystal AS, Shaw AT, Sequist LV, Friboulet L, Niederst MJ, Lockerman EL, et al. Patient-derived models of acquired resistance can identify effective drug combinations for cancer. Science. 2014;346:1480-6.

Debelenko LV, Raimondi SC, Daw N, Shivakumar BR, Huang D, Nelson M, et al. Renal cell carcinoma with novel VCL-ALK fusion: new representative of ALK-associated tumor spectrum. Mod Pathol. 2011;24:430-42.

Demeure MJ, Aziz M, Rosenberg R, Gurley SD, Bussey KJ, Carpten JD. Whole-genome sequencing of an aggressive BRAF wild-type papillary thyroid cancer identified EML4-ALK translocation as a therapeutic target. World J Surg. 2014;38:1296-305.

Doebele RC, Davis LE, Vaishnavi A, Le AT, Estrada-Bernal A, Keysar S, et al. An Oncogenic NTRK Fusion in a Patient with Soft-Tissue Sarcoma with Response to the Tropomyosin-Related Kinase Inhibitor LOXO-101. Cancer Discov. 2015;5:1049-57.

Drilon A, Li G, Dogan S, Gounder M, Shen R, Arcila M, et al. What hides behind the MASC: clinical response and acquired resistance to entrectinib after ETV6-NTRK3 identification in a mammary analogue secretory carcinoma (MASC). Ann Oncol. 2016a;27:920-6.

Drilon A, Somwar R, Wagner JP, Vellore NA, Eide CA, Zabriskie MS, et al. A Novel Crizotinib-Resistant Solvent-Front Mutation Responsive to Cabozantinib Therapy in a Patient with ROS1-Rearranged Lung Cancer. Clin Cancer Res. 2016b;22:2351-8.

Farago AF, Le LP, Zheng Z, Muzikansky A, Drilon A, Patel M, et al. Durable Clinical Response to Entrectinib in NTRK1-Rearranged Non-Small Cell Lung Cancer. J Thorac Oncol. 2015;10:1670-4.

Approved v1.0

Formisano L, Nappi L, Rosa R, Marciano R, D'Amato C, D'Amato V, et al. Epidermal growth factor-receptor activation modulates Src-dependent resistance to lapatinib in breast cancer models. Breast Cancer Res. 2014;16:R45.

Frattini V, Trifonov V, Chan JM, Castano A, Lia M, Abate F, et al. The integrated landscape of driver genomic alterations in glioblastoma. Nat Genet. 2013;45:1141-9.

Gainor JF, Dardaei L, Yoda S, Friboulet L, Leshchiner I, Katayama R, et al. Molecular Mechanisms of Resistance to First- and Second-Generation ALK Inhibitors in ALK-Rearranged Lung Cancer. Cancer Discov. Cancer Discovery. Epub 2016 Jul 18.

Gao SP, Chang Q, Mao N, Daly LA, Vogel R, Chan T, et al. JAK2 inhibition sensitizes resistant EGFR-mutant lung adenocarcinoma to tyrosine kinase inhibitors. Sci Signal. 2016;9:ra33.

Giacomini CP, Sun S, Varma S, Shain AH, Giacomini MM, Balagtas J, et al. Breakpoint analysis of transcriptional and genomic profiles uncovers novel gene fusions spanning multiple human cancer types. PLoS Genet. 2013;9:e1003464.

Girotti MR, Pedersen M, Sanchez-Laorden B, Viros A, Turajlic S, Niculescu-Duvaz D, et al. Inhibiting EGF receptor or SRC family kinase signaling overcomes BRAF inhibitor resistance in melanoma. Cancer Discov. 2013;3:158-67.

Goto K, Yang JC-H, Kim D-W, Lu S, Seto T, Yang J-J, et al. Phase II study of crizotinib in East Asian patients (pts) with ROS1-positive advanced non-small cell lung cancer (NSCLC). J Clin Oncol, 2016;34 (suppl) Abstract 9022.

Greco A, Miranda C, Pierotti MA. Rearrangements of NTRK1 gene in papillary thyroid carcinoma. Mol Cell Endocrinol. 2010;321:44-9.

Gu TL, Deng X, Huang F, Tucker M, Crosby K, Rimkunas V, et al. Survey of tyrosine kinase signaling reveals ROS kinase fusions in human cholangiocarcinoma. PLoS One. 2011;6:e15640

Isozaki H, Ichihara E, Takigawa N, Ohashi K, Ochi N, Yasugi M, et al. Non-Small Cell Lung Cancer Cells Acquire Resistance to the ALK Inhibitor Alectinib by Activating Alternative Receptor Tyrosine Kinases. Cancer Res. 2016;76:1506-16.

Ji JH, Oh YL, Hong M, Yun JW, Lee HW, Kim D, et al. Identification of driving ALK fusion genes and genomic landscape of medullary thyroid cancer. PLoS Genet. 2015;11:e1005467.

Jokinen E, Laurila N, Koivunen P, Koivunen JP. Combining targeted drugs to overcome and prevent resistance of solid cancers with some stem-like cell features. Oncotarget. 2014;5:9295-307.

Kanda R, Kawahara A, Watari K, Murakami Y, Sonoda K, Maeda M, et al. Erlotinib resistance in lung cancer cells mediated by integrin β1/Src/Akt-driven bypass signaling. Cancer Res. 2013;73:6243-53.

Kang Y, Hu W, Ivan C, Dalton HJ, Miyake T, Pecot CV, et al. Role of focal adhesion kinase in regulating YB-1-mediated paclitaxel resistance in ovarian cancer. J Natl Cancer Inst. 2013;105:1485-95.

Approved v1.0

Kaplan DR, Hempstead BL, Martin-Zanca D, Chao MV, Parada LF. The trk protooncogene product: a signal transducing receptor for nerve growth factor. Science. 1991;252:554-8.

Kazandjian D, Blumenthal GM, Chen HY, He K, Patel M, Justice R, et al. FDA approval summary: crizotinib for the treatment of metastatic non-small cell lung cancer with anaplastic lymphoma kinase rearrangements. Oncologist. 2014;19:e5-11.

Kazandjian D, Blumenthal GM, Luo L, He K, Fran I, Lemery S, et al. Benefit-Risk Summary of Crizotinib for the Treatment of Patients with ROS1 Alteration-Positive, Metastatic Non-Small Cell Lung Cancer. Oncologist. 2016;21:974-80.

Khozin S, Blumenthal GM, Zhang L, Tang S, Brower M, Fox E, et al. FDA approval: ceritinib for the treatment of metastatic anaplastic lymphoma kinase-positive non-small cell lung cancer. Clin Cancer Res. 2015;21:2436-9.

Kim J, Lee Y, Cho HJ, Lee YE, An J, Cho GH, et al. NTRK1 fusion in glioblastoma multiforme. PLoS One. 2014;9(3):e91940.

Kim DW, Mehra R, Tan DS, Felip E, Chow LQ, Camidge DR, et al. Activity and safety of ceritinib in patients with ALK-rearranged non-small-cell lung cancer (ASCEND-1): updated results from the multicentre, open-label, phase 1 trial. Lancet Oncol. 2016a;17:452-63

Kim DW, Tiseo M, Ahn MJ, Reckamp KL, Hansen KH, Kim S-W, et al. Brigatinib (BRG) in patients (pts) with crizotinib (CRZ)-refractory ALK+ non-small cell lung cancer (NSCLC): First report of efficacy and safety from a pivotal randomized phase (ph) 2 trial (ALTA). J Clin Oncol. 2016b; 34 (suppl): Abstract 9007

Klein R, Jing SQ, Nanduri V, O'Rourke E, Barbacid M. The trk proto-oncogene encodes a receptor for nerve growth factor. Cell. 1991;65:189–97.

Knezevich SR, Garnett MJ, Pysher TJ, Beckwith JB, Grundy PE, Sorensen PH. ETV6-NTRK3 gene fusions and trisomy 11 establish a histogenetic link between mesoblastic nephroma and congenital fibrosarcoma. Cancer Res. 1998;58:5046-8.

Koivunen JP, Mermel C, Zejnullahu K, Murphy C, Lifshits E, Holmes AJ, et al. EML4-ALK fusion gene and efficacy of an ALK kinase inhibitor in lung cancer. Clin Cancer Res. 2008;14:4275-83.

Kris MG, Johnson BE, Berry LD, Kwiatkowski DJ, Iafrate AJ, Wistuba II, et al. Using multiplexed assays of oncogenic drivers in lung cancers to select targeted drugs. JAMA. 2014;311:1998-2006.

Lamballe F, Klein R, Barbacid M. trkC, a new member of the trk family of tyrosine protein kinases, is a receptor for neurotrophin-3. Cell. 1991;66:967–79.

Lasham A, Print CG, Woolley AG, Dunn SE, Braithwaite AW. YB-1: oncoprotein, prognostic marker and therapeutic target? Biochem J. 2013;449:11–23.

Approved v1.0

Lee J, Borboa AK, Chun HB, Baird A, Eliceiri BP. Conditional deletion of the focal adhesion kinase FAK alters remodeling of the blood-brain barrier in glioma. Cancer Res. 2010;70:10131-40.

Lee J, Lee SE, Kang SY, Do IG, Lee S, Ha SY, et al. Identification of ROS1 rearrangement in gastric adenocarcinoma. Cancer. 2013;119:1627-1635.

Lee J, Kim HC, Hong JY, Wang K, Kim SY, Jang J, et al. Detection of novel and potentially actionable anaplastic lymphoma kinase (ALK) rearrangement in colorectal adenocarcinoma by immunohistochemistry screening. Oncotarget. 2015;6:24320-32.

Lin E, Li L, Guan Y, Soriano R, Rivers CS, Mohan S, et al. Exon array profiling detects EML4-ALK fusion in breast, colorectal, and non-small cell lung cancers. Mol Cancer Res. 2009;7:1466-76.

Lipson D, Capelletti M, Yelensky R, Otto G, Parker A, Jarosz M, et al. Identification of new ALK and RET gene fusions from colorectal and lung cancer biopsies. Nat Med. 2012;18:382-4.

Lovly CM, Gupta A, Lipson D, Otto G, Brennan T, Chung CT, et al. Inflammatory myofibroblastic tumors harbor multiple potentially actionable kinase fusions. Cancer Discov. 2014;4(8):889-95.

Lu S, Mok T, Lu Y, Zhou J, Shi Y, Sriuranpong V, et al. Phase 3 study of first-line crizotinib vs pemetrexed−cisplatin/carboplatin in East Asian patients with ALK+ advanced non-squamous non-small cell lung cancer. J Clin Oncol. 2016; 34(suppl): Abstract 9058.

Malik SM, Maher VE, Bijwaard KE, Becker RL, Zhang L, Tang SW, S et al. U.S. Food and Drug Administration approval: crizotinib for treatment of advanced or metastatic non-small cell lung cancer that is anaplastic lymphoma kinase positive. Clin Cancer Res. 2014;20:2029-34.

Mariño-Enríquez A, Ou WB, Weldon CB, Fletcher JA, Pérez-Atayde AR. ALK rearrangement in sickle cell trait-associated renal medullary carcinoma. Genes Chromosomes Cancer. 2011;50:146-53.

Martin-Zanca D, Hughes SH, Barbacid M. A human oncogene formed by the fusion of truncated tropomyosin and protein tyrosine kinase sequences. Nature. 1986;319:743-8.

Osoba D, Zee B. Completion rates in health-related quality-of-life assessment: approach of the National Cancer Institute of Canada Clinical Trials Group. Stat Med. 1998;17:603-12.

Ou SH, Tan J, Yen Y, Soo RA. ROS1 as a 'druggable' receptor tyrosine kinase: lessons learned from inhibiting the ALK pathway. Expert Rev Anticancer Ther. 2012;12:447-56.

Ou SH, Ahn JS, De Petris L, Govindan R, Yang JC, Hughes B, et al. Alectinib in Crizotinib-Refractory ALK-Rearranged Non-Small-Cell Lung Cancer: A Phase II Global Study. J Clin Oncol. 2016;34:661-8.

Ou SH, Jänne PA, Bartlett CH, Tang Y, Kim DW, Otterson GA, et al. Clinical benefit of continuing ALK inhibition with crizotinib beyond initial disease progression in patients with advanced ALK-positive NSCLC. Ann Oncol. 2014;25:415-22.

Approved v1.0

Park K, Tan EH, O'Byrne K, Zhang L, Boyer M, Mok T, et al. Afatinib versus gefitinib as first-line treatment of patients with EGFR mutation-positive non-small-cell lung cancer (LUX-Lung 7): a phase 2B, open-label, randomised controlled trial. Lancet Oncol. 2016;17:577-89.

Perot G, Soubeyran I, Ribeiro A, Bonhomme B, Savagner F, Boutet-Bouzamondo N, et al. Identification of a recurrent STRN/ALK fusion in thyroid carcinomas. PLoS ONE. 2014;9:e87170.

Rikova K, Guo A, Zeng Q, Possemato A, Yu J, Haack H, et al. Global survey of phosphotyrosine signaling identifies oncogenic kinases in lung cancer. Cell. 2007;131:1190-203.

Rosell R, Carcereny E, Gervais R, Vergnenegre A, Massuti B, Felip E, et al. Erlotinib versus standard chemotherapy as first-line treatment for European patients with advanced EGFR mutation-positive non-small-cell lung cancer (EURTAC): a multicentre, open-label, randomised phase 3 trial. Lancet Oncol. 2012;13:239-46.

Ross JS, Wang K, Gay L, Al-Rohil R, Rand JV, Jones DM, et al. New routes to targeted therapy of intrahepatic cholangiocarcinomas revealed by next-generation sequencing. Oncologist. 2014;19:235-42.

Russo M, Misale S, Wei G, Siravegna G, Crisafulli G, Lazzari L, et al. Acquired Resistance to the TRK Inhibitor Entrectinib in Colorectal Cancer. Cancer Discov. 2016;6:36-44.

Sartore-Bianchi A, Ardini E, Bosotti R, Amatu A, Valtorta E, Somaschini A, et al. Sensitivity to Entrectinib Associated With a Novel LMNA-NTRK1 Gene Fusion in Metastatic Colorectal Cancer. J Natl Cancer Inst. 2016;108:pii:djv306.

Schuler M, Wu YL, Hirsh V, O'Byrne K, Yamamoto N, Mok T, et al. First-Line afatinib versus chemotherapy in patients with non-small cell lung cancer and common epidermal growth factor receptor gene mutations and brain metastases. J Thorac Oncol. 2016;11:380-90.

Seguin L, Desgrosellier JS, Weis SM, Cheresh DA. Integrins and cancer: regulators of cancer stemness, metastasis, and drug resistance. Trends Cell Biol. 2015;25:234-40.

Serrels A, Lund T, Serrels B, Byron A, McPherson RC, von Kriegsheim A, et al. Nuclear FAK controls chemokine transcription, Tregs, and evasion of anti-tumor immunity. Cell. 2015;163:160-73.

Shaw AT, Hsu PP, Awad MM, Engelman JA. Tyrosine kinase gene rearrangements in epithelial malignancies. Nat Rev Cancer. 2013a;13:772-87.

Shaw AT, Kim DW, Nakagawa K, Seto T, Crinó L, Ahn MJ, et al. Crizotinib versus chemotherapy in advanced ALK-positive lung cancer. N Engl J Med. 2013b;368:2385-94.

Shaw AT, Ou SH, Bang Y-J, Camidge DR, Solomon BJ, Salgia R, et al. Crizotinib in ROS1-rearranged non-small-cell lung cancer. N Engl J Med. 2014a;371:1963-71.

Shaw AT, Kim DW, Mehra R, Tan DS, Felip E, Chow LQ, Camidge DR, et al. Ceritinib in ALK-rearranged non-small-cell lung cancer. N Engl J Med. 2014b;370:1189-97.

Approved v1.0

Shaw AT, Gandhi L, Gadgeel S, Riely GJ, Cetnar J, West H, et al. Alectinib in ALK-positive, crizotinib-resistant, non-small-cell lung cancer: a single-group, multicentre, phase 2 trial. Lancet Oncol. 2016a;17:234-42.

Shaw AT, Friboulet L, Leshchiner I, Gainor JF, Bergqvist S, Brooun A, et al. Resensitization to Crizotinib by the Lorlatinib ALK Resistance Mutation L1198F. N Engl J Med. 2016b;374:54-61.

Simon R. Optimal two-stage designs for phase II clinical trials. Control Clin Trials. 1989;10:1-10.

Skálová A, Vanecek T, Sima R, Laco J, Weinreb I, Perez-Ordonez B, et al. Mammary analogue secretory carcinoma of salivary glands, containing the ETV6-NTRK3 fusion gene: a hitherto undescribed salivary gland tumor entity. Am J Surg Pathol. 2010;34:599-608.

Soda M, Choi YL, Enomoto M, Takada S, Yamashita Y, Ishikawa S, et al. Identification of the transforming EML4-ALK fusion gene in non-small-cell lung cancer. Nature. 2007;448:561-6.

Solomon BJ, Mok T, Kim DW, Wu YL, Nakagawa K, Mekhail T, et al. First-line crizotinib versus chemotherapy in ALK-positive lung cancer. N Engl J Med. 2014;371:2167-77.

Solomon BJ, Cappuzzo F, Felip E, Blackhall FH, Costa DB, Kim DW, et al. Intracranial efficacy of crizotinib versus chemotherapy in patients with advanced ALK-positive non-small-cell lung cancer: results from PROFILE 1014. J Clin Oncol. 2016;34(24):2858-65.

Song A, Kim TM, Kim DW, Kim S, Keam B, Lee SH, et al. Molecular Changes Associated with Acquired Resistance to Crizotinib in ROS1-Rearranged Non-Small Cell Lung Cancer. Clin Cancer Res. 2015;21:2379-87.

Squinto SP, Stitt TN, Aldrich TH, Davis S, Bianco SM, Radziejewski C, et al. trkB encodes a functional receptor for brain-derived neurotrophic factor and neurotrophin-3 but not nerve growth factor. Cell. 1991;65: 885–93.

Stransky N, Cerami E, Schalm S, Kim JL, Lengauer C. The landscape of kinase fusions in cancer. Nat Commun. 2014;5:4846.

Stratford AL, Habibi G, Astanehe A, Jiang H, Hu K, Park E, et al. Epidermal growth factor receptor (EGFR) is transcriptionally induced by the Y-box binding protein-1 (YB-1) and can be inhibited with Iressa in basal-like breast cancer, providing a potential target for therapy. Breast Cancer Res. 2007;9:R61.

Thakur R, Trivedi R, Rastogi N, Singh M, Mishra DP. Inhibition of STAT3, FAK and Src mediated signaling reduces cancer stem cell load, tumorigenic potential and metastasis in breast cancer. Sci Rep. 2015;5:10194.

Tognon C, Knezevich SR, Huntsman D, Roskelley CD, Melnyk N, Mathers JA, et al. Expression of the ETV6-NTRK3 gene fusion as a primary event in human secretory breast carcinoma. Cancer Cell. 2002;2:367-76.

Torre LA, Bray F, Siegel RL, Ferlay J, Lortet-Tieulent J, Jemal A.. Global caner statistics, 2012. CA Cancer J Clin; 2015;65:87-108.

Approved v1.0

Vaishnavi A, Capelletti M, Le AT, Kako S, Butaney M, Ercan D, et al. Oncogenic and drug-sensitive NTRK1 rearrangements in lung cancer. Nat Med. 2013;19:1469-72.

Vaishnavi A, Le AT, Doebele RC. TRKing down an old oncogene in a new era of targeted therapy. Cancer Discov. 2015;5:25-34.

Voena C, Varesio LM, Zhang L, Menotti M, Poggio T, Panizza E, et al. Oncogenic ALK regulates EMT in non-small cell lung carcinoma through repression of the epithelial splicing regulatory protein 1. Oncotarget. 2016;7:33316-30.

Wiesner T, He J, Yelensky R, Esteve-Puig R, Botton T, Yeh I, et al. Kinase fusions are frequent in Spitz tumours and spitzoid melanomas. Nat Commun. 2014;5:3116.

Wilson C, Nicholes K, Bustos D, Lin E, Song Q, Stephan JP, et al. Overcoming EMT-associated resistance to anti-cancer drugs via Src/FAK pathway inhibition. Oncotarget. 2014;5:7328-41.

Wu G, Diaz AK, Paugh BS, Rankin SL, Ju B, Li Y, et al. The genomic landscape of diffuse intrinsic pontine glioma and pediatric non-brainstem high-grade glioma. Nat Genet. 2014;46:444-50.

Yamamoto H, Yoshida A, Taguchi K, Kohashi K, Hatanaka Y, Yamashita A, et al. ALK, ROS1 and NTRK3 gene rearrangements in inflammatory myofibroblastic tumours. Histopathology. 2016;69:72-83.

Ying J, Lin C, Wu J, Guo L, Qiu T, Ling Y, et al. Anaplastic lymphoma kinase rearrangement in digestive tract cancer: Implication for targeted therapy in Chinese population. PLoS One. 2015;10:e0144731.

Yoshida R, Okumura S, Sasaki T, Osaki Y. Src mediates acquired resistance to ALK inhibitor in ALK-rearranged non-small cell lung cancers. Cancer Res. 2016;76(14 Suppl):Abstract nr 2121.

Zhang S, Huang WC, Li P, Guo H, Poh SB, Brady SW, et al. Combating trastuzumab resistance by targeting SRC, a common node downstream of multiple resistance pathways. Nat Med. 2011;17:461-9.

Zhang S, Yu D. Targeting Src family kinases in anti-cancer therapies: turning promise into triumph. Trends Pharmacol Sci. 2012;33:122-8.

Zhang S, Huang WC, Zhang L, Zhang C, Lowery FJ, Ding Z, et al. SRC family kinases as novel therapeutic targets to treat breast cancer brain metastases. Cancer Res. 2013;73:5764-74.

Zheng Z, Liebers M, Zhelyazkova B, Cao Y, Panditi D, Lynch KD, et al. Anchored multiplex PCR for targeted next-generation sequencing. Nat Med. 2014;20:1479-84.

Approved v1.0

APPENDIX 1 PHASE 1 REQUIRED LABORATORY ASSESSMENTS

Phase 1 Dose Escalation					
**Hematology**	**Chemistry***	**Coagulation**	**Urinalysis**	**Pregnancy Test**	**Hypogonadism blood sampling between 08:00-11:00 in MALE patients only**
Hemoglobin and Hematocrit	Alanine aminotransferase	Prothrombin time or international normalized ratio	Urine dipstick for urine protein	Serum from female patients of childbearing potential	Total testosterone
Platelets	Aspartate aminotransferase	Partial thromboplastin time	Urine dipstick for urine blood		Sex hormone-binding globulin
White blood cell count	Alkaline phosphatase		Urine dipstick for urine glucose		Luteinizing hormone
Absolute neutrophils	Sodium		Urine dipsticks for urine ketones		Follicle-stimulating hormone
Absolute lymphocytes	Potassium		Specific gravity		Prolactin
Absolute monocytes	Magnesium				
Absolute eosinophils	Magnesium				
Absolute basophils	Chloride				
Absolute reticulocytes	Total calcium				
	Total bilirubin				
	Blood urea nitrogen				
	Creatinine				
	Uric acid				
	Glucose (non-fasted)				
	Albumin				
	Phosphorus				
	Calcium				
	Lactate Dehydrogenase				
	Creatine phosphokinase				
	Cardiac troponin				

*For Hy's Law cases, in addition to repeating AST and ALT, laboratory tests should include albumin, creatine phosphokinase (CPK), total bilirubin, direct and indirect bilirubin, gamma-glutamyl transferase, prothrombin time (PT)/INR, and alkaline phosphatase

Approved v1.0

APPENDIX 2 PHASE 2 REQUIRED LABORATORY ASSESSMENTS

Phase 2 Dose Escalation					
**Hematology**	**Chemistry***	**Coagulation**	**Urinalysis**	**Pregnancy Test**	**Hypogonadism blood sampling between 08:00-11:00 in MALE patients only**
Hemoglobin	Alanine aminotransferase	PT or INR	Urine dipstick for urine protein	Serum from female patients of childbearing potential	Total testosterone (TT)
Platelets	Aspartate aminotransferase	PTT	Urine dipstick for urine blood		Sex hormone-binding globulin
White blood cell count	Alkaline phosphatase		Urine dipstick for urine glucose		Luteinizing hormone
Absolute neutrophils	Sodium		Urine dipsticks for urine ketones		Follicle-stimulating hormone (FSH)
Absolute lymphocytes	Potassium		Specific gravity		Prolactin
Absolute monocytes	Magnesium				
Absolute eosinophils	Magnesium				
Absolute basophils	Chloride				
Absolute reticulocytes	Total calcium				
	Total bilirubin				
	BUN or urea				
	Creatinine				
	Uric acid				
	Glucose (non-fasted)				
	Albumin				
	Phosphorus				
	Calcium				
	Lactate Dehydrogenase				
	creatine phosphokinase				
	Cardiac troponin				

*For Hy's Law cases, in addition to repeating AST and ALT, laboratory tests should include albumin, creatine phosphokinase (CPK), total bilirubin, direct and indirect bilirubin, gamma-glutamyl transferase, prothrombin time (PT)/INR, and alkaline phosphatase

Approved v1.0

APPENDIX 3 JAPANESE PATIENT-ONLY LEAD-IN COHORT (LIC) SCHEDULE OF ASSESSMENTS

Study Calendar for Japan Lead-In Cohort of TPX-0005-01

	**Screening**	**Lead-in PK/Cycle 0 (3 days)**	**Cycle 1 (21-day cycle)**			**Cycle 2 (28-day cycle)**		**Cycle 3 and Cycle 4**	**Cycle 5 and Beyond**	**End of Treatment**	**Safety Follow-up**	**Long-Term Follow-up**
**Protocol Activity**	**Days -28 to -1**	**Day -3**	**Day 1**	**Day 8**	**Day 15**	**Day 1**	**Day 15**	**Day 1**	**Day 1**			
Visit Window[1]	NA			±1	±1	±2	±2	±2	±2	±7	+7	
Informed Consent[2]	X											
Tumor Molecular Alteration[3]	X											
Tumor Treatment History[4]	X											
Medical History	X	X	X	X	X	X	X	X	X	X	X	
Physical Examination	X	X	X	X	X	X	X	X	X	X	X	
ECOG Performance Status	X	X	X	X	X	X	X	X	X	X	X	
Height	X											
Weight	X	X	X	X	X	X	X	X	X	X	X	
Vital Signs[5]	X	X	X	X	X	X	X	X	X	X	X	
Dispense TPX-0005			X			X		X	X			
TPX-0005 Compliance				X	X	X	X	X	X	X		
Laboratory												
Complete Blood Count with Differential[6]	X	X	X	X	X	X	X	X	X	X	X	
Reticulocyte Count (%)	X	X	X			X		X	X	X	X	
Complete Metabolic Panel[7]	X	X	X	X	X	X	X	X	X	X	X	
Coagulation[8]	X	X	X	X	X	X		X	X	X	X	
Endocrine (for male patients only)[9]	X	X				X		X	X	X	X	
Pregnancy Test (serum)[10]	X	X				X		X	X	X	X	

	**Screening**	**Lead-in PK/Cycle 0 (3 days)**	**Cycle 1 (21-day cycle)**			**Cycle 2 (28-day cycle)**		**Cycle 3 and Cycle 4**	**Cycle 5 and Beyond**	**End of Treatment**	**Safety Follow-up**	**Long-Term Follow-up**
**Protocol Activity**	**Days -28 to -1**	**Day -3**	**Day 1**	**Day 8**	**Day 15**	**Day 1**	**Day 15**	**Day 1**	**Day 1**			
Urine Analysis[11]	X	X	X	X	X	X		X	X	X	X	
Blood Specimens for ccfDNA Enrichment[12]	X									X		
Cardiac Safety Monitoring												
Triplicate 12-lead ECG[13]	X	X	X	X	X	X		X		X		
Echocardiogram for LVEF Assessment[14]	X							X	X	X		
Cardiac Troponin-I[15]	X											
Pharmacokinetics												
Plasma for Full PK[16]		X			X							
Plasma for Abbreviated PK[17]			X	X		X		X				
CSF (Optional)[18]						X						
Tumor Assessment												
CT or MRI (chest/abdomen/pelvis)[19]	X							X	X			
MRI of Brain[20]	X							X	X			
Bone Scan[21]	X							X	X			
Other Clinical Assessments												
Adverse Events		X	X	X	X	X	X	X	X	X	X	
Concomitant Medications[22]	X	X	X	X	X	X	X	X	X	X	X	
Survival Follow-Up												X

[1] **Visit Window:** The 28-day screening period starts on the day informed consent is signed by the patient. The safety visit should be within 28 days from the last dose of TPX-0005.

[2] **Informed Consent:** Must be obtained before undergoing any protocol-specific procedures.

[3] **Tumor Molecular Alteration:** Confirmation of *ALK+, ROS1+*, or *NTRK+* rearrangement (submit molecular pathology report-source document) and submit archival tumor tissue (please refer to Section 5.1).

[4] **Tumor Treatment History:** Documentation of disease progression on prior tyrosine kinase inhibitor (TKIs), duration of treatment with each prior TKI, prior chemotherapy regimens and duration of each chemotherapy regimens, prior immunotherapy regimens and duration of each immunotherapy regimens (if applicable), prior radiation to brain (if brain metastases present, and methods of radiation: whole brain radiation, stereotactic radiosurgery).

[5] **Vital Signs:** Body temperature, blood pressure, heart rate, respiratory rate, pain level (0-10).

[6] **Complete Blood Count with Differential:** White blood cell count, hemoglobin, platelet, absolute neutrophils, absolute lymphocytes, absolute monocytes, absolute eosinophils, and absolute basophils.

[7] **Complete Chemistry Panel:** Sodium, potassium, chloride, bicarbonate, blood urea nitrogen, creatinine, magnesium, phosphorus, calcium, uric acid, total protein, albumin, lactate dehydrogenase, aspartate aminotransferase, alanine aminotransferase, total bilirubin, alkaline phosphatase.

[8] **Coagulation:** Prothrombin time/international normalized ratio, partial thromboplastin time.

[9] **Endocrine (for male patients only):** Total testosterone, sex hormone-binding globulin, follicle-stimulating hormone, luteinizing hormone, prolactin. These laboratories should be drawn between 8 am to 11 am in the morning.

[10] **Serum Pregnancy Test:** For female patients of childbearing potential, a serum pregnancy test, with sensitivity of at least 25 mIU/mL, will be performed on 2 occasions prior to starting study therapy: once at the start of screening and once at the baseline visit, immediately before investigational product administration. Only patients with negative serum pregnancy tests are allowed to enroll. Serum pregnancy test will also be performed at the beginning of each cycle of visit.

[11] **Urine Analysis:** Urinalysis includes dipstick analysis (protein, glucose, ketones, blood, and specific gravity). A microscopic (white blood cells/high power field [HPF], red blood cells/HPF, and any additional findings) exam need only be performed if the urinalysis result is abnormal. More frequent and complete examinations may be performed at the Investigator's discretion if medically indicated; results should be recorded on unscheduled visit CRFs.

[12] **Blood Specimens for ccfDNA Enrichment (20-mL blood specimen):** Blood will be collected across 2 10 ml Streck Cell-Free DNA Blood Collection Tubes (for ccfDNA analysis) prior to the first dose of TPX-0005 and at EOT for exploratory ccfDNA molecular profiling analyses. Details for handling of these specimens including processing, storage, and shipment will be provided in the Study Manual.

[13] **Triplicate 12-lead ECGs:** At each time point, 3 consecutive 12-lead ECGs will be performed approximately 2 minutes apart to determine mean QTc interval. Triplicate 12-lead ECGs will all be time matched with PK with the exception of during the screening period and at the EOT. Triplicate ECGs will be collected as follows:

a) At Screening,
b) Day -3 (Lead-in/Cycle 0) after single-dose administration at pre-dose (0 hour), at 1, 2, 4, 6, 8, 24, and 48 hours post-dose.
c) Cycle 1 Day 1, at pre-dose (0 hour) and 4 hours post-dose.
d) Cycle 1 Day 8, at pre-dose (0 hour)
e) Cycle 1 Day 15 after single-dose administration at pre-dose (0 hour), at 1, 2, 4, 6, 8, and 24 hours post-dose.
f) Cycles 2-4, Day 1 at pre-dose (0 hour) and 4 hours post-dose.

In addition to these time points, ECGs should be repeated as clinically indicated. Additional ECG time points may be included based on the emerging data. Interpretation of the tracing will be made by a central ECG laboratory. Each ECG tracing should be labeled with the study number, patient initials, patient number, date, and kept in the source documents at the study site. Only clinically significant abnormalities will be recorded in the AE CRF page. Clinically significant abnormalities at screening/baseline should be recorded on the relevant medical history/current medical conditions CRF page. Clinically significant findings must be discussed with TP Therapeutics and Medical Monitor prior to enrolling the patient in the study.

[14] **Echocardiography for LVEF Assessments:** Echocardiography to be performed at screening, and after every 3 cycles of treatment (± 7 days) (Cycles 4, 7, 10, 13 and so on), and at the EOT visit (± 7 days).

[15] **Cardiac Troponin-I:** Cardiac troponin-I will be done at baseline then repeated and assessed whenever the ECG morphology demonstrates possible myocardial ischemia or infarction. If troponin CTCAE grade ≥ 3 is seen, this parameter must be repeated twice a week until resolution to CTCAE grade ≤ 1.

[16] **Plasma for Full PK:** Timing for full PK: Lead-in PK pre-dose (time 0), 1, 2, 4, 6, 8, 24, and 48 hours post-dose. Cycle 1 Day 15 pre-dose (time 0), 1, 2, 4, 6, 8, 24, and 48 hours post-dose.

[17] **Plasma for Abbreviated PK:** Timing for abbreviated PK: at pre-dose (0 hour) and 4 hours post-dose on Cycle 1 Day 1 and Cycles 2-4 Day 1, and at pre-dose (0 hour) on Cycle 1 Day 8.

[18] **Cerebral Spinal Fluid (CSF) Collection (optional):** If a patient undergoes a lumber puncture, a sample of CSF should be collected for exploratory analysis of TPX-0005 concentration, if possible. If a CSF sample is collected, a blood sample for PK analysis should also be collected at approximately the same time as the CSF sample.

[19] **CT or MRI Chest/Abdomen/Pelvis Tumor Assessment:** Tumor assessments will include all known or suspected disease sites. For all tumor assessments, the method of assessment that was used at baseline should be the same method used throughout the study. For patients who are without documented disease progression, CT or MRI scans to be done at every 2 cycles (after 7 weeks for the first assessment and then every 2 cycles up to Cycle 18 and then every 3 cycles up to Cycle 36 and then every 4 cycles thereafter until documented progression of disease). Patient responses will be confirmed > 4 weeks later after the initial documentation of response by the Investigator. For patients who have documented disease progression but are continuing TPX-0005 post-RECIST progression, CT or MRI scans are to be done according to local institutional standard of care. Every effort should be made to maintain the assessment scheduling relative to Cycle 1 Day 1 especially if there are dosing cycle interruptions due to toxicities. For all patients, copies of radiologic images must be available for independent central radiology review as determined by TP Therapeutics.

[20] **MRI of Brain:** Gadolinium contrast-enhanced MRI must be used for assessment of CNS lesions at baseline with contingent slices of 1 mm for lesions 5 to 10 mm in size, 5 mm for lesions greater than 10 mm. For the Phase 1a Dose Escalation portion of the study, only patients with documented baseline CNS metastases will be assessed by MRI of the brain at every 2 cycles (after 7 weeks for the first assessment and then every 2 cycles up to Cycle 18 and then every 3 cycles up to Cycle 36 and then every 4 cycles thereafter until documented progression of disease). Patient responses will be confirmed > 4 weeks later after the initial documentation of response by the Investigator. For patients who have documented disease progression but are continuing TPX-0005 post-RECIST progression, MRI scans of the brain are to be done according to local institutional standard of care.

[21] **Bone Scans:** Bone scans (or bone MRI if preferred by Investigator) will be performed at baseline for all patients and repeated every 3 cycles while on study only if evidence of bone metastases is observed at baseline.

[22] **Concomitant Medications and Non-Drug Supportive Interventions:** All concomitant medications and non-drug supportive interventions should be recorded in the CRF.

APPENDIX 4 SAFETY REVIEW PROCESS FOR JAPANESE ONLY PATIENT LIC

Following 1 cycle (21 days) of treatment, a safety review will be performed by Japanese Investigators and TP Therapeutics to determine Japan site participation in Phase 2. Emerging Phase 1 safety/PK data in Western patients will also be used as a reference.

Approved v1.0

APPENDIX 5 PATIENT-REPORTED OUTCOMES: EORTC-QLQ-C30

[redacted]

[redacted]

APPENDIX 6 PATIENT-REPORTED OUTCOMES: EORTC-QLQ-LC13

[redacted]

APPENDIX 7 TABLE OF MEDICATIONS THAT ARE STRONG CYP3A4/5 INHIBITORS OR INDUCERS OF CYP3A4/5 OR CAUSE QTC PROLONGATION

Strong CYP3A4/5 inhibitors	Strong CYP3A4/5 inducers
Macrolide antibiotics: clarithromycin and telithromycin	Carbamazepine
	Efavirenz
Anti-fungal: itraconazole, ketoconazole, voriconazole, posaconazole	Phenobarbital
	Phenytoin
Anti-virals: lopinavir, indinavir, nelfinavir, ritonavir, saquinavir, amprenavir	Rifampin
	Rifabutin
Conivaptan	Rifapentin
Mibefradil	St John's wort
Nefazodone	
Troleandomycin	
Miscellaneous: Grapefruit juice	

Medications that causes QTc prolongation

Amiodarone	Astemizole	Azithromycin	Chlorpromazine
Disopyramide	Terfenadine	Clarithromycin	Haloperidol
Dofetilide	Chloroquine	Erhthromycin	Mesoridazine
Flecainide	Halofantrine	Moxifloxacin	Pimozide
Ibutilide	Probucol	Sparfloxacin	Thioridazine
Procainamide	Pentamidine	Domperidone	Levomethadyl
Quinidine	Bepridil	Droperidol	Methadone
Sotalol	Arsenic trioxide	Vavdetanib	Citalopram
cisapride			

Approved v1.0

A PHASE 1/2, OPEN-LABEL, MULTI-CENTER, FIRST-IN-HUMAN STUDY OF THE SAFETY, TOLERABILITY, PHARMACOKINETICS, AND ANTI-TUMOR ACTIVITY OF TPX-0005 IN PATIENTS WITH ADVANCED SOLID TUMORS HARBORING ALK, ROS1, OR NTRK1-3 REARRANGEMENTS (TRIDENT-1)

Protocol Number:	TPX-0005-01 (CA1271024)
Version (Date):	16.0 (02-Jan-2024)
Amendment (Date):	Amendment 15 (02-Jan-2024)
Compound:	TPX-0005, BMS-986472
Compound Name	Repotrectinib
United States (US) Investigational New Drug (IND) Number:	130465
European Clinical Trial Database (EudraCT) Number:	2016-003616-13
Turning Point Therapeutics:	Turning Point Therapeutics, Inc.<br>(a wholly owned subsidiary of Bristol Myers Squibb Company)<br><br>Route 206 & Province Line Road<br>Princeton, NJ 08543 USA

This document contains confidential information belonging to Turning Point Therapeutics, Inc. (a wholly owned subsidiary of Bristol Myers Squibb Company). Except as otherwise agreed to in writing, by accepting or reviewing this document, you agree to hold this information in confidence and not copy or disclose it to others (except where required by applicable law) or use it for unauthorized purposes. In the event of any actual or suspected breach of this obligation, Turning Point Therapeutics, Inc. must be promptly notified.

SPONSOR APPROVALS

Protocol Number:	TPX-0005-01 (CA127-1024)
Version (Date):	16.0 (02 Jan 2024)
Protocol Title:	A Phase 1/2, Open-Label, Multi-Center, First-in-Human Study of the Safety, Tolerability, Pharmacokinetics, and Anti-Tumor Activity of TPX-0005 in Patients with Advanced Solid Tumors Harboring ALK, ROS1, or NTRK1-3 Rearrangements (TRIDENT-1)

See Electronic Signature and Date on File in Electronic Document Management System Date:________________

[redacted]

Bristol Myers Squibb Company

PROTOCOL AGREEMENT

I confirm that I have read this protocol. I will comply with the protocol and the principles of Good Clinical Practice (GCP), applicable local, state, and federal laws, as well as other applicable country laws and the appropriate International Council for Harmonisation guidance documents.

Protocol Number:	TPX-0005-01 (CA1271024)
Version (Date):	16.0 (02 Jan 2024)
Protocol Title:	A Phase 1/2, Open-Label, Multi-Center, First-in-Human Study of the Safety, Tolerability, Pharmacokinetics, and Anti-Tumor Activity of TPX-0005 in Patients with Advanced Solid Tumors Harboring *ALK*, *ROS1*, or *NTRK1-3* Rearrangements (TRIDENT-1)

____ Date:____________________

Investigator Signature

____

Name and Title

____

Site #

____

Site Name

Approved v2.0 930210036 2.0

PROTOCOL SYNOPSIS

**Title**	A Phase 1/2 Open-Label, Multi-Center, First-in-Human Study of the Safety, Tolerability, Pharmacokinetics, and Anti-Tumor Activity of TPX-0005 in Patients with Advanced Solid Tumors Harboring ALK, ROS1, or NTRK1-3 Rearrangements (TRIDENT-1)
**Sponsor**	Turning Point Therapeutics, Inc. (a wholly owned subsidiary of Bristol Myers Squibb Company)
**Development Phase**	1/2
**Primary Objectives**	Phase 1 Dose Escalation<br>• To determine the first cycle dose-limiting toxicities (DLTs) of repotrectinib given to adult subjects with advanced solid malignancies harboring an ALK, ROS1, NTRK1, NTRK2, or NTRK3 gene rearrangement.<br>• To determine the maximum tolerated dose (MTD) of repotrectinib in adult subjects with advanced solid malignancies harboring an ALK, ROS1, NTRK1, NTRK2, or NTRK3 gene rearrangement.<br>• To determine the biologically effective dose and recommended Phase 2 dose (RP2D) of repotrectinib for adult subjects with advanced solid malignancies harboring an ALK, ROS1, NTRK1, NTRK2, or NTRK3 gene rearrangement.<br>Phase 2 Study<br>• To determine the confirmed objective response rate (ORR) as assessed by Blinded Independent Central Review (BICR) of repotrectinib in each subject population expansion cohort of advanced solid tumors that harbor a ROS1, NTRK1, NTRK2, or NTRK3 gene rearrangement.
**Secondary Objectives**	Phase 1 Dose Escalation<br>• To evaluate the safety and tolerability of repotrectinib at various doses in subjects with advanced solid tumors that harbor an ALK, ROS1, NTRK1, NTRK2, or NTRK3 gene rearrangement.<br>• To evaluate the single- and multiple-dose pharmacokinetic (PK) profiles of repotrectinib.<br>• To determine the effect of food on the pharmacokinetics (PK) of repotrectinib.<br>• To determine the preliminary objective response rate (ORR) by BICR and the clinical benefit rate (CBR) of repotrectinib, in subjects with advanced solid tumors that harbor an ALK, ROS1, NTRK1, NTRK2, or NTRK3 gene rearrangement.<br>• To evaluate the potential of repotrectinib to induce cytochrome P450 3A (CYP3A) using midazolam as a probe substrate.

	Phase 2 Study<br>• To determine the duration of response (DOR), time to response (TTR), and clinical benefit rate (CBR) of repotrectinib, as assessed by BICR, in each subject population expansion cohort of advanced solid tumors that harbor a ROS1, NTRK1, NTRK2, or NTRK3 gene rearrangement.<br>• To estimate the progression-free survival (PFS) and overall survival (OS) of subjects treated with repotrectinib with advanced solid tumors that harbor a ROS1, NTRK1, NTRK2, or NTRK3 gene rearrangement.<br>• To evaluate the safety and tolerability of repotrectinib when administered at the RP2D in subjects with advanced solid tumors that harbor a ROS1, NTRK1, NTRK2, or NTRK3 gene rearrangement.<br>• To determine the intracranial objective response rate (IC-ORR) of repotrectinib and Central Nervous System PFS (CNS-PFS) in subjects presenting with measurable brain metastases at baseline, using modified Response Evaluation Criteria in Solid Tumors (RECIST) Version 1.1 assessment.<br>• To confirm PK of repotrectinib at the RP2D.<br>• To assess treatment-related symptoms and general health status using validated instruments of subject-reported outcomes (EORTC-QLQ-C30 and LC-13 when applicable) in subjects treated with repotrectinib.
**Exploratory Objectives**	Phase 1<br>• To evaluate the pharmacodynamic (PD) effects of repotrectinib in subjects with advanced solid tumors that harbor an ALK, ROS1, NTRK1, NTRK2, or NTRK3 gene fusion.<br>• To explore the potential prognostic utility of genomic alterations and characterize intrinsic or acquired resistance to repotrectinib using liquid biopsy and tissue samples.<br>Phase 2<br>• To explore the potential prognostic utility of genomic alterations and characterize intrinsic or acquired resistance to repotrectinib using liquid biopsy and tissue samples.<br>• To explore association between ORR by subgroups including demographic and baseline risk factors in each expansion cohort.
**Study Design**	Phase 1 Study: (Phase 1a dose escalation, Phase 1b food-effect sub-study, Phase 1c dose escalation with food), and Midazolam Drug-Drug Interaction (DDI) sub-study<br>The starting daily dose level in the dose escalation segment was 40 mg QD. A total of 6 dose levels were evaluated in the initial Phase 1a study where subjects were to take repotrectinib while fasted with a lead-in dose at Day -3. In addition, the effect of high -fat, high -calorie food on the

single dose PK of repotrectinib (Phase 1b) was evaluated in the first 3 dose levels (Dose Level 1-3). Based on the findings from the Phase 1b study and the overall preliminary PK and clinical data, further dosing of repotrectinib continuously with food will be evaluated in the Phase 1c study (dosing cohorts outlined below) that includes up to 5 additional dosing cohorts. The escalation of each dose level will be based on the overall PK, and preliminary safety and efficacy data seen within each cohort. The cohorts in Phase 1c will follow a standard 3 + 3 design and dosing within a cohort will not continue if the dose is determined to have exceeded the MTD based on DLTs. The Phase 1c study will begin with dose level 7 (120 mg QD with food). During the DLT observation period (Cycle 1), if none of the first 3 subjects enrolled experiences a DLT, the dose of repotrectinib will be escalated by one dose level. If 1 one of the first 3 subjects experiences a DLT, up to 3 additional subjects will be enrolled at that dose level. If there are no additional DLTs (ie, ≤1 of 6 subjects with a DLT), then dose escalation will proceed. Assuming Dose Levels 7 and 8 are cleared without exceeding the MTD, patients will then be enrolled into Dose Level 9, which will start initially with a once daily (QD) dose of 160 mg for one week and, if no DLTs within the first seven days of QD dosing are observed, then patients will have their doses escalated to the 160 mg twice daily (BID) schedule. The rationale for this schedule is based on preliminary PK data obtained within the Phase 1a/1b portions of the study showing a high Cmax with the lead-in dose and a possible metabolic induction effect with repotrectinib leading to Cmax and AUC reductions at steady-state (Cycle 1, Day 15 [C1D15]). In correlation with this observation, the onset of the reported adverse events (AEs) of grade 2 and grade 3 dizziness occurred within the first few days of dosing, and the symptoms of dizziness were more tolerable during the following weeks of dosing after the metabolic induction effect occurred. Therefore, the incremental dose increases of 160 mg QD, followed by 160 mg BID starting one week later, potentially can avoid the high Cmax while maintaining the preferred exposure levels. If Dose Level 9 exceeds the MTD, patients will be enrolled into either: (1) Dose Level 10 (120 mg QD with food for 7 days followed by 120 mg BID with food) or (2) Dose Level 11 (120 mg QD with food for 7 days followed by 240 mg QD). Subject enrollment to Dose Levels 10 and 11 will occur simultaneously.

Dose Levels (QD and BID dosing): Phase 1a and Phase 1b

Dose Level	Proposed Dose Level (QD)	Increment from Previous Dose (%)
-1	20 mg QD	(50% decrease)
1	40 mg QD	Starting Dose
2	80 mg QD	100%

3	160 mg QD	100%
4	240 mg QD	50%

Dose Level	Proposed Dose Level (BID)	Increment from Previous Dose of 240 mg QD (%)
5	160 mg BID	33%
6	200 mg BID	25%

Phase 1c Dose Levels (QD and BID dosing with food)

Dose Level	Proposed Dose Levels and Schedules
-1	80 mg QD with food
7	120 mg QD with food
8	160 mg QD with food
9	160 mg QD with food for 7 days followed by 160 mg BID with food IF there are no DLTs within the first 7 days while on QD dosing
10*	120 mg QD with food for 7 days followed by 120 mg BID with food IF there are no DLTs within the first 7 days while on QD dosing
11*	120 mg QD with food for 7 days followed by 240 mg QD with food if there are no DLTs within the first 7 days

* If Dose Level 9 exceeds the MTD, subjects will be enrolled into Dose Levels 10 and 11 simultaneously

Criteria for Dose Escalation and Determination of MTD (Phase 1)

For any given schedule, the MTD is the highest dose level of repotrectinib expected to cause a DLT in fewer than 33% of the treated subjects in the first treatment cycle.

To support subject safety at each dose level, a delay of at least 7 days will be mandatory between the administration of the first dose to the first subject and administration of the first dose to subsequent subjects (ie, at least a 7-day gap between the first and second subjects initiating study drug in all sites).

Previously in Cohorts 1-6, up to 6 subjects were evaluated prior to dose escalation. Going forward and starting with Cohort 7, a standard 3 + 3 design will be followed with requirement of up to 3 additional subjects total if a DLT is observed in the first 3 subjects.

Expansion of up to 6 subjects is also allowed if 0 of 3 subjects are reported with a DLT to further explore efficacy ONLY after a dose level is cleared consistent with “backfilling” a cohort ONLY if the cohort has

shown preliminary efficacy so that subjects are not exposed to potentially subtherapeutic doses. For example, if no DLTs are reported in the first 3 subjects treated at the Cohort 7 dose level (120 mg QD with food), and dose escalation proceeds into Cohort 8 (160 mg QD with food), yet at least one response out of the 3 subjects treated within Cohort 7 was observed the protocol will allow the cohort expansion of up to 6 potential subjects within Cohort 7. This expansion is being explored based on the anti-tumor activity that has already been shown with doses at or below Dose Level 7 (120 mg QD with food).

During the DLT observation period (Cycle 1), if none of the first 3 subjects enrolled are reported with a DLT, the dose of repotrectinib will be escalated by one dose level in the subsequent cohort. If 1 subject in the first 3 subjects are reported with a DLT, up to 3 additional subjects will be enrolled at that dose level. If there are no additional DLTs (ie, ≤1 of 6 subjects with a DLT), then dose escalation will proceed. If there are 2 subjects reported with DLTs among the first 3 subjects enrolled, or there is a second DLT in up to 6 subjects enrolled at any dose level, the MTD will have been exceeded and no additional subjects will be started at that dose. The MTD will then be established at the prior dose level. If the prior dose cohort only has 3 enrolled subjects, the cohort will be expanded to up to 6 total subjects. If there are ≤1 of 6 subjects with a DLT at that dose level, this dose will be considered the MTD of repotrectinib.

To fully characterize the safety or PK/PD of repotrectinib, subjects who are discontinued from treatment before completing the DLT observation period (Cycle 1) due to disease progression or other event unrelated to repotrectinib, or who did not complete the PK evaluations within Cycle 1, will be replaced.

Dose escalation may be terminated at any time based on emerging safety concerns without establishing the MTD.

Dose escalations within the Phase 1 portion of the study will stop if: (1) there is evidence in the dose-exposure relationship of saturation of absorption; (2) at least 6 subjects have been treated at a dose that is considered the RP2D, or (3) all doses exceed the MTD.

Once the MTD of repotrectinib has been determined, the RP2D will be determined at or below the MTD. At least 6 subjects will be treated at the RP2D prior to proceeding into the Phase 2 portion of the study.

Criteria for Defining Dose-limiting Toxicities

Category	Criteria
Toxicities resulting in an excessive number of missed doses	Inability to deliver > 75% of the planned doses in Cycle 1 treatment (ie, at least 16 doses in a 21-day cycle) because of toxicity at least related to repotrectinib

Hematologic toxicities	• CTCAE grade ≥ 4 neutropenia (absolute neutrophil count [ANC] < 0.5 × 109/L)<br>• CTCAE grade ≥ 4 thrombocytopenia (platelets < 25 × 109/L)<br>• CTCAE grade ≥ 4 anemia not explained by underlying disease or condition<br>• CTCAE grade ≥ 3 febrile neutropenia (defined as ANC < 1000/mm3 with a single temperature of ≥ 38.3°C [≥ 101°F] or a sustained temperature of ≥ 38°C [≥ 100.4°F] for > 1 hour)
Renal	• CTCAE grade ≥ 3 creatinine increase (> 3 × upper limit of normal [ULN])
Hepatic	• CTCAE grade ≥ 3 total bilirubin elevation (> 3 × ULN)<br>• CTCAE grade ≥ 2 total bilirubin elevation (> 1.5 × ULN) AND CTCAE grade ≥ 2 ALT or AST elevation (> 3 × ULN)<br>• CTCAE grade ≥ 3 ALT elevation (> 5 × ULN)<br>• CTCAE grade ≥ 3 AST elevation (> 5 × ULN)
Pancreatic	• CTCAE grade ≥ 2 pancreatitis<br>• CTCAE grade ≥ 3 amylase or lipase elevation
Cardiac	• CTCAE grade ≥ 3
Other adverse events	• CTCAE grade ≥ 3 vomiting or nausea despite optimal anti-emetic therapy<br>• CTCAE grade ≥ 3 diarrhea despite optimal anti-diarrhea treatment<br>• Any CTCAE grade ≥ 3 AE, except for the exclusions noted below<br>• In view of the Investigators and the Sponsor any other unacceptable toxicity encountered
Exceptions to DLT criteria	• CTCAE grade 3 or 4 elevations in alkaline phosphatase<br>• CTCAE grade 3 or 4 electrolytes abnormalities that are adequately managed by IV or PO supplementations<br>• CTCAE grade 3 fatigue < 7 days<br>• CTCAE grade 2 dizziness that can be managed with dose interruptions and/or dose reductions

Subjects are eligible for DLT evaluation if they are reported with a DLT after at least one dose of study drug, or are not reported with a DLT having taken a minimum of 75% of doses expected during the first cycle of treatment. Subjects who do not fulfill these requirements and who discontinue study participation prior to completing the DLT assessment period will be replaced.

Dose Modifications: Intra-subject dose escalation/reduction & re-escalation criteria (Phase 1)

Intra-subject dose escalation is permitted on study ONLY after a subject has completed the third cycle of repotrectinib unless there is documented disease progression by RECIST (Version 1.1) after the DLT observation window has passed for that subject. After the third cycle is completed, individual subjects may be considered for treatment at a dose of repotrectinib higher than the dose that is initially assigned. In order for a

	subject to be eligible for intra-subject dose escalation and treated at a higher dose of repotrectinib, he or she must have received the lower dose for at least 3 cycles of therapy without a drug-related toxicity of CTCAE grade ≥ 2 or have documented disease progression after completing the DLT observation period (Cycle 1) without a DLT. Observation of CTCAE grade ≤ 3 laboratory abnormalities not requiring dose modifications (as identified in the table of Criteria for Defining Dose-limiting Toxicities) would be permitted. Moreover, the new, higher dose with which the subject is to be treated must be a dose that has completed evaluation where at least 3 subjects have completed Cycle 1 with repotrectinib without having a reported DLT and that does not exceed the MTD or the RP2D. Any further increases after the initial intra-subject dose escalation are subject to the same rules as for the initial intra-subject escalation. Consultation with the Sponsor's Medical Monitor must occur prior to any intra-subject dose escalation decision.<br>For subjects who undergo a dose reduction for an AE other than a protocol defined DLT, a subsequent re-escalation of the dose may be considered appropriate if deemed safe by the Investigator and after consultation with the Sponsor's Medical Monitor.<br>Subjects enrolled into Dose Level 9 starting at 160 mg QD, may be escalated to 160 mg BID provided no DLTs are reported within the first seven days while on the QD schedule. If the escalated dose of 160 mg BID is not well tolerated, the Investigator may choose to decrease the dose to a lower level after consultation with the Sponsor's Medical Monitor.<br>Midazolam DDI sub-study (Phase 1)<br>To evaluate the potential effect of repotrectinib on CYP3A induction, a midazolam (MDZ) DDI sub-study will be conducted in 6 PK evaluable subjects after RP2D determination. All subjects will be administered a single 5 mg oral dose of MDZ alone on Day -2 under fasted conditions (no food 8 hours before through 2 hours after dosing of MDZ), and then starting on Cycle 1 Day 1, subjects will begin daily treatment of 160 mg repotrectinib QD followed by 160 mg BID on Cycle 1 Day 15. On Cycle 1 Day 22, another single dose of 5 mg MDZ will be administered under fasted conditions (no food 8 hours before through 2 hours after dosing of MDZ) concurrently with repotrectinib (morning dose). Serial plasma samples will be collected for up to 24 hours following both MDZ dosing periods to assess PK. Once the DDI assessment periods end, subjects will continue the study treatment until progression of disease as determined by BICR, unacceptable toxicity, or consent withdrawal. Repotrectinib can be taken without regard to food between Cycle 1 Day 1 to Cycle 1 Day 21, as well as after completion of the DDI assessment.<br>Please see subject eligibility criteria for midazolam DDI sub-study in APPENDIX 7.<br>Recommended Phase 2 Dose (RP2D)

	The RP2D is the dose chosen for further study in Phase 2 based on the Phase 1 results. The RP2D will be the MTD unless one or more of the following suggest an alternate dose below the MTD would be preferable: (a) clinically significant anti-tumor effect (complete response, partial response or prolonged stable disease) occurs below the MTD, in which case a clinically active dose level may be selected as the RP2D; (b) the MTD is not achieved, in which case the highest dose level administered may become the RP2D; or (c) toxicities reported beyond the DLT observation period (Cycle 1) require selecting the RP2D below the MTD level.<br>Once the MTD of repotrectinib has been determined, if fewer than 6 subjects have been treated at the dose identified as the RP2D, enrollment will continue until at least 6 subjects are treated at the RP2D, which may be at or below the MTD.<br>Based on safety, PK and preliminary efficacy data obtained in Phase 1, the RP2D of repotrectinib was determined to be:<br>• 160 mg QD for the first 14 days, may increase to 160 mg BID. Repotrectinib can be taken with or without food.<br>• (Subjects must meet all below criteria while on 160 mg QD prior to dose increasing to 160 mg BID: No grade ≥3 treatment-related AE, unmanageable grade ≥2 dizziness, ataxia or paresthesia; or grade ≥3 clinically significant lab abnormalities).<br>Phase 2 Study<br>The Phase 2 portion of the study will be with single-agent repotrectinib at the identified RP2D and will enroll subjects with ROS1+, NTRK1+, NTRK2+, or NTRK3+ advanced solid malignancies.<br>The Phase 2 segment of this study will consist of 6 subject expansion cohorts (EXP) (described in the Inclusion Criteria). All subjects in the Phase 2 portion of this trial will receive repotrectinib orally once daily followed by twice daily (starting with 160 mg QD for the first 14 days, may increase to 160 mg BID based on subject's tolerability assessment at C1D15 in repeated 4-week cycles.<br>On Study Assessments (Phase 1 and Phase 2)<br>Tumor assessments will be performed at Screening, at the end of Cycle 2 (within the Phase 1a study this was approximately 7 weeks from the initial repotrectinib treatment ± 2 days; and in Phase 1c given the duration of Cycle 1 is 4 weeks, it will correspond to 8 weeks from the initial repotrectinib treatment ± 7 days), every 2 cycles (± 7 days) up to the end of Cycle 18 and then every 3 cycles (± 7 days) up to the end of Cycle 36 and then every 4 cycles (± 7 days) thereafter until documented progression of disease regardless of treatment delays resulting from toxicity, and at the End of Treatment (EOT) if more than 4 weeks have passed since the last imaging assessment). At EOT visit, the subject must undergo an EOT tumor assessment evaluation (CT or MRI; MRI of

	the Brain and Bone Scan, if applicable) PRIOR to treatment discontinuation to evaluate for radiologic disease progression if treatment is being discontinued for a reason other than BICR-confirmed radiographic disease progression. After treatment discontinuation, tumor assessments should continue at the current scan interval at the time of treatment discontinuation until a subject begins a new course of cancer therapy or withdraws consent if there is no BICR-confirmed radiologic progression at the time of treatment discontinuation.<br>A safety follow-up visit will be conducted within 28 days of last dose of repotrectinib. Additionally, each subject will be contacted by telephone approximately every 3 months following study discontinuation until death, loss to follow-up, or withdrawal of consent in order to assess disease progression and survival status.<br>For Phase 2, all efforts should be made to obtain the EOT radiographic scan before treatment discontinuation, and where possible inform and discuss with the Sponsor's Medical Monitor when considering treatment discontinuation. Disease progression must be determined by BICR. For subjects in Phase 2 with CNS disease who have been on study for at least 2 cycles of treatment with a best response of stable disease (SD) per RECIST Version 1.1 AND without treatment-related grade ≥ 2 AEs, dose escalation to 160 mg BID will be allowed as per Investigator's discretion and after discussion with the Sponsor's Medical Monitor.<br>Subjects may continue repotrectinib treatment after clinical or radiographic progression if he or she is continuing to experience clinical benefit, in the opinion of the Investigator, and after discussion with the Sponsor Medical Monitor.<br>Safety will be monitored via laboratory assessments, physical examinations, electrocardiograms (ECG), vital signs, and AEs. Study assessments for the Phase 1 and Phase 2 portion will be performed as per the Study Calendars. In Phase 2, a Data Monitoring Committee (DMC) will be established to monitor safety and conduct benefit-risk assessment on a routine basis that will be outlined in a separate DMC charter.<br>If a subject is not eligible for participation following the 28-day screening period, a subject may be re-screened.
**Number of Subjects**	Phase 1a Dose Escalation: 12-48<br>Phase 1b Food Effect: 12-48<br>Phase 1c Dose Escalation with food: approximately 30<br>Midazolam DDI sub-study: 6 PK evaluable<br>Phase 2: Approximately 630
**Enrollment Criteria**	For country-specific requirements, please refer to APPENDIX 8.<br><br>Inclusion criteria for Phase 1:

	1) Histologically or cytologically confirmed diagnosis of locally advanced, or metastatic solid tumor (including primary CNS tumors) (Stage IV, American Joint Committee on Cancer Version 7) that harbors an ALK, ROS1, NTRK1, NTRK2, or NTRK3 gene rearrangement as by:<br>a) All diagnostic tests must be conducted in a Clinical Laboratory Improvement Amendments (CLIA) lab or equivalent.<br>b) Any nucleic acid-based diagnostic testing method (eg, next-generation sequencing [NGS], Sanger sequencing, reverse transcription-polymerase chain reaction) performed at a local clinical laboratory improvement amendments-certified (CLIA-certified) or equivalently accredited diagnostic laboratory. All tests must have been performed according to the product's instructions for use (IFU).<br>c) Break-apart fluorescence in situ hybridization (FISH) is allowed for diagnosis of ALK, ROS1, NTRK1, NTRK2, and NTRK3 rearrangements. For ALK rearrangement detection in non-small-cell lung cancer (NSCLC), the FISH test has to be performed using the FDA-approved Abbott Molecular's Vysis® ALK Break-apart FISH Probe Kit. All tests must have been performed according to the product's IFU.<br>d) Immunohistochemistry (IHC) detection of ROS1, NTRK1, NTRK2, and NTRK3 rearrangement will not directly qualify subjects. Archival tumor samples of these IHC positive subjects (ROS1, TRKA, TRKB, and TRKC) will have to be sent to the Sponsor's designated central laboratory for confirmation of ROS1, NTRK1, NTRK2, and NTRK3 rearrangement prior to enrollment onto the trial. IHC detection of ALK rearrangement in NSCLC is allowed if performed using the Ventana ALK (D5F3) companion diagnostic assay. All tests must have been performed according to the product's IFU.<br>e) For subjects enrolled per the diagnostic tests outlined above and approved by the Sponsor, central laboratory confirmation of the specific gene alteration is not required before the start of repotrectinib treatment as long as adequate archival tissue is sent to the Sponsor's designated central laboratory.<br>2) All subjects must have archival or fresh tissue samples available and/or collected prior to enrollment. Formalin-fixed paraffin-embedded (FFPE) tissue block(s) from initial diagnosis that contain sufficient tissue to generate at least 10 (preferably 15), 5-micron thick unstained slides will be collected. If no FFPE block is available, then at least 10 (preferably 15) unbaked, 5-micron thick unstained slides with a minimum of 10% (preferably 20%) tumor content and 1 Hematoxylin and eosin stain (H&E) slide must be provided.

	Specimens will be sent to the Sponsor-designated central laboratories for ALK, ROS1, or NTRK rearrangement status confirmation.<br>3) Eastern Cooperative Oncology Group (ECOG) Performance Status (PS) 0-1<br>4) Age ≥18 (or age ≥ 20 of age as required by local regulation).<br>5) Willing and able to provide written institutional review board (IRB)/institutional ethics committee-approved Informed Consent.<br>6) At least 1 measurable target lesion according to RECIST (Version 1.1). CNS-only measurable disease as defined by RECIST (Version 1.1) is allowed.<br>7) Prior cytotoxic chemotherapy for advanced or metastatic disease is allowed. At the time of starting treatment with repotrectinib, at least 14 days or 5 half-lives (whichever is shorter) must have elapsed after discontinuation of prior cytotoxic chemotherapy (or at least 42 days for prior nitrosoureas, mitomycin C, and liposomal doxorubicin) and all side effects from prior treatments must have resolved to grade ≤ 1 (NCI CTCAE Version 4.03) with the exception of alopecia.<br>8) Prior immunotherapy (eg, anti-PD-1, anti-PDL1, anti-TIM3, and anti-OX40) is allowed. At the time of starting treatment with repotrectinib, at least 14 days must have elapsed after discontinuation of prior immunotherapy treatment and all immune-related side effects from prior treatments must have resolved to grade ≤ 1.<br>9) Subjects with advanced solid tumors harboring ALK, ROS1, NTRK1, NTRK2, or NTRK3 rearrangements are eligible. There is no limit to the number of prior chemotherapy, immunotherapy, or TKI regimens.<br>10) At least 7 days or 5 half-lives (whichever is shorter) must have elapsed since completion of treatment with the last ALK inhibitor (ALKi), ROS1 inhibitor (ROSi), or TRK inhibitor (TRKi) prior to starting treatment with repotrectinib for subjects enrolling into the TKI-pretreated expansion cohorts. In the midazolam DDI sub-study, if a subject received prior treatment of a TKI that is a moderate/strong inducer or inhibitor of CYP3A, such as lorlatinib, the required wash-out period will be at least 14 days to allow complete wash out of its induction or inhibition effects. All side effects from prior treatments with ALKi, ROS1i, and TRKi must have resolved to grade ≤ 1 prior to starting treatment with repotrectinib; however, the most immediate treatment prior to enrollment does not have to be a TKI.<br>a) Prior ALKi allowed include crizotinib, ceritinib, alectinib, brigatinib, lorlatinib, ensartinib, ASP3026, TSR- 011<br>b) Prior ROS1i allowed include crizotinib, ceritinib, lorlatinib, brigatinib, ensartinib, DS6051b, ASP3026, cabozantinib<br>c) Prior TRKi allowed include entrectinib, larotrectinib, LOXO-195, DS6051b

d) Other prior ALKi, ROS1i, and TRKi not listed above may be allowed after discussion with the Sponsor

11) Subjects with asymptomatic CNS metastases (treated or untreated) and/or asymptomatic leptomeningeal carcinomatosis are eligible to enroll if they satisfy the following criteria:

a) Subjects requiring steroids at a stable or decreasing dose (≤ 12 mg/day dexamethasone or equivalent) for at least 14 days are eligible. Subjects on stable doses of levetiracetam (same dose for 14 days) are eligible to be enrolled.

b) A minimum of 14 days must have elapsed from the completion of whole brain radiation treatment (WBRT) before the start of treatment with repotrectinib, and all side effects (with the exception of alopecia) from WBRT are resolved to CTCAE grade ≤ 1.

c) A minimum of 7 days must have elapsed from the completion of stereotactic radiosurgery before the start of treatment with repotrectinib, and all side effects (with the exception of alopecia) from stereotactic radiosurgery are resolved to CTCAE grade ≤ 1.

12) Baseline laboratory values fulfilling the following requirements

Absolute Neutrophils Count (ANC)	≥1,500/mm3 (1.5 x 109/L)
Platelets (PLT)	≥100,000/mm3 (100 x 109/L)
Hemoglobin	≥ 9.0 g/dL transfusions are allowed
Serum Creatinine or Creatinine Clearance*	Within Normal Limits or > 40 mL/min
Total Serum Bilirubin	<1.5 x ULN
Liver Transaminases (AST/ALT)	<2.5 x ULN; < 5 x ULN if liver metastases are present
Alkaline Phosphatase (ALP)	<2.5 x ULN; < 5 x ULN if liver and/or bone metastasis are present.
Serum calcium, magnesium and potassium	Normal or ≤ CTCAE grade 1 with or without supplementation.

Abbreviations: AST/ALT = aspartate aminotransferase/alanine aminotransferase, ULN = upper limit of normal

* calculated by Cockcroft and Gault's formula: (140 - age [yr]) x body weight [Kg] x 1.23 x (0.85 if female) / serum creatinine [µmol/L].

13) Reproductive Status: For female and male participants, see Section 5.1 for the full list of criteria.

14) Capability to swallow capsules intact (without chewing, crushing, or opening).

15) Life expectancy ≥ 3 months.

	16) Willingness and ability to comply with scheduled visits, treatment plan, laboratory tests, and other study procedures.<br>Inclusion criteria for Phase 2:<br>For country-specific requirements, please refer to APPENDIX 8.<br>1) Histologically or cytologically confirmed diagnosis of locally advanced, or metastatic solid tumor (including primary CNS tumors) that harbors a ROS1 or NTRK1-3 gene fusion. Note: Locally advanced disease is defined as Stage III when subject is not a candidate for surgery, radiation, or multi-modality therapy and metastatic disease is defined as Stage IV per the American Joint Committee on Cancer Eighth Edition Cancer Staging Manual guidelines (Rami-Porta 2017).<br>2) Subject must have a documented ROS1 or NTRK1-3 gene fusion determined by tissue-based local testing using either:<br>a) a next-generation sequencing (NGS) or quantitative polymerase chain reaction (qPCR) test will be accepted to determine molecular eligibility.<br>i) Adequate tumor tissue needs to be sent to the Sponsor designated central diagnostic laboratory for retrospective confirmation by a central diagnostic laboratory test selected by the Sponsor. In cases where archived tumor tissue is not available, a fresh biopsy should be obtained at Screening or as soon as possible after enrollment. See the Study Laboratory Manual for details.<br>ii) If NGS was used, the partner of the fusion target gene needs to be identified and reported.<br>iii) Retrospective confirmation by a central diagnostic laboratory test selected by the Sponsor is not required if ROS1 or NTRK1-3 gene fusion were determined by the Repotrectinib clinical trial assay (CTA).<br>OR<br>b) a fluorescence in situ hybridization (FISH) test AND prospective confirmation of fusion status by a central diagnostic laboratory test selected by the Sponsor PRIOR to enrollment will be accepted to determine molecular eligibility.<br>i) Adequate tumor tissue must be sent to the Sponsor designated central diagnostic laboratory for prospective confirmation by a central diagnostic laboratory test selected by the Sponsor PRIOR to enrollment. In cases where archived tumor tissue is not available, a fresh biopsy should be obtained for this purpose. See the Study Laboratory Manual for details.

	All tests need to be performed in a Clinical Laboratory Improvement Amendments (CLIA) laboratory or equivalently accredited diagnostic laboratory.<br>3) Subjects must have completed a performance score as follows based on age at time of enrollment:<br>a) Eastern Cooperative Oncology Group (ECOG) PS 0–1 (≥18 years)<br>b) Karnofsky score of at least 50 (16 to <18 years)<br>c) Lansky score of at least 50 (<16 years)<br>Subjects who are unable to walk because of paralysis or tumor pain, but who are in a wheelchair, will be considered ambulatory for the purpose of assessing the performance score.<br>4) Age ≥12 (or as required by local regulations).<br>5) Willing and able to provide written institutional review board (IRB)/institutional ethics committee-approved Informed Consent, or an Informed Consent signed by a parent or legal guardian accompanied by an Assent signed by the participant as required by local regulations.<br>6) At least 1 measurable target lesion according to RECIST (Version 1.1) prospectively confirmed by Blinded Independent Central Radiology Review (BICR), selected by the Sponsor, PRIOR to enrollment. Subjects with CNS-only measurable target lesion ≥10 mm as defined by RECIST (Version 1.1) are eligible.<br>7) Subjects with advanced solid tumors harboring ROS1, NTRK1, NTRK2, or NTRK3 rearrangement will be assigned into 6 distinct expansion (EXP) cohorts provided all inclusion and exclusion criteria are met:<br>a) EXP-1: ROS1 TKI-naïve ROS1+ NSCLC (n=110).<br>i) No prior exposure to a ROS1 TKI is allowed.<br>ii) Up to one prior line of chemotherapy OR immunotherapy is allowed (chemo- or immunotherapy-based combination regimen is considered as one line of treatment).<br>b) EXP-2: 1 Prior ROS1 TKI AND 1 Platinum-based Chemotherapy ROS1+ NSCLC (n=120).<br>i) Disease progression or intolerant to one prior line of a ROS1 TKI.<br>ii) ROS1 TKIs used in a prior line of treatment are limited to crizotinib, ceritinib, entrectinib, or lorlatinib. Note: Any previous exposure to a ROS1 TKI is considered as one prior line of TKI treatment (eg, if the same ROS1 TKI was given before and after a chemotherapy or other systemic therapy, it is considered as 2 prior TKIs and the subject would not be eligible for EXP-2).

	iii) In addition, the subject must have received one prior line of platinum-based chemotherapy OR one prior line of platinum-based chemotherapy in combination with immunotherapy, before or after a ROS1 TKI (Note: subject is not eligible if he/she has been treated with more than one line of chemotherapy OR has received immunotherapy alone).<br>c) EXP-3: 2 Prior ROS1 TKIs and NO Chemotherapy or Immunotherapy ROS1+ NSCLC (n=80).<br>i) Disease progression or intolerant to 2 prior lines of a ROS1 TKI treatment.<br>ii) ROS1 TKIs used in prior lines of treatment are limited to crizotinib, ceritinib, entrectinib, lorlatinib, brigatinib, ensartinib, or cabozantinib. Other prior ROS1 TKI agents that are not listed may be allowed after discussion with the Sponsor Medical Monitor. Note: Any previous exposure to a ROS1 TKI is considered as one prior line of TKI treatment (eg, if 2 different ROS1 TKIs are utilized, or the same ROS1 TKI was given before and after a chemotherapy or other systemic therapy, it is considered as 2 prior TKIs and the subject would be eligible).<br>iii) No prior lines of chemotherapy or immunotherapy are allowed<br>d) EXP-4: 1 Prior ROS1 TKI and NO Chemotherapy or Immunotherapy ROS1+ NSCLC (n=120).<br>i) Disease progression or intolerant to one prior line of a ROS1 TKI.<br>ii) ROS1 TKIs used in a prior line of treatment are limited to crizotinib, ceritinib, entrectinib, or lorlatinib. Note: Any previous exposure to a ROS1 TKI is considered as one prior line of TKI treatment (eg, if the same ROS1 TKI was given before and after a chemotherapy or other systemic therapy, it is considered as 2 prior TKIs and the subject would not be eligible for EXP-4).<br>iii) Note: No prior lines of chemotherapy or immunotherapy are allowed<br>e) EXP-5: TRK TKI-naïve NTRK+ solid tumors (n= approximately 80).<br>i) No prior exposure to a TRK TKI is allowed.<br>ii) Any number of prior lines of chemo or immunotherapy is allowed.<br>iii) As of global protocol amendment Version 15.0 (or equivalent version for respective countries), subjects with NSCLC are not allowed.

f) EXP-6: TRK TKI-pretreated NTRK+ solid tumors (n= approximately 120).

   i) Disease progression or intolerant to 1 or 2 prior TRK TKIs.

   ii) TRK TKIs used in prior lines of treatment are limited to entrectinib, larotrectinib, selitrectinib (LOXO-195), and cabozantinib. Other prior TRK TKIs that are not listed may be allowed after discussion with the Sponsor Medical Monitor. Note: Any previous exposure of a TRK TKI is considered as one prior line of TKI treatment, (eg, if 2 different TRK TKIs are utilized or the same TRK TKI was used before and after a chemo- or other systemic therapy, it is counted as 2 prior TKIs and the subject would be eligible).

   iii) Any number of prior lines of chemo- or immunotherapy are allowed.

   iv) As of global protocol amendment Version 15.0 (or equivalent version for respective countries), subjects with NSCLC are not allowed.

8) Required wash-out time that is related to prior therapies before starting repotrectinib treatment:

   a) If the immediate prior treatment was a ROS1 or TRK TKI: 7 days or 5 half-lives (whichever is shorter) must have elapsed since completion of treatment with the last TKI for subjects enrolling into the pretreated expansion cohorts (EXP-2, -3, -4, and -6). All side effects from prior treatments with a ROS1 or TRK TKI must have resolved to grade ≤ 1 prior to starting treatment with repotrectinib.

   b) At least 14 days or 5 half-lives (whichever is shorter) must have elapsed after discontinuation of prior systemic chemotherapy (or at least 42 days for prior nitrosoureas and mitomycin C) and all side effects from prior treatments must have resolved to grade ≤1 with the exception of alopecia.

   c) At least 14 days must have elapsed after discontinuation of prior immunotherapy and all immune-related side effects from prior immunotherapy must have resolved to grade ≤1.

9) Subjects with asymptomatic CNS metastases (treated or untreated) and/or asymptomatic leptomeningeal carcinomatosis are eligible to enroll if they satisfy the following criteria:

   a) Subjects requiring steroids at a stable or decreasing dose (≤ 12 mg/day dexamethasone or equivalent) for at least 14 days.

   b) Subjects on stable doses of levetiracetam (same dose for 14 days).

   c) A minimum of 14 days must have elapsed from the completion of whole brain radiation treatment (WBRT) before the start of

treatment with repotrectinib, and all side effects (with the exception of alopecia) from WBRT are resolved to grade ≤1.

d) A minimum of 7 days must have elapsed from the completion of stereotactic radiosurgery before the start of treatment with repotrectinib, and all side effects (with the exception of alopecia) from stereotactic radiosurgery are resolved to grade ≤1.

10) Baseline laboratory values fulfilling the following requirements:

Absolute Neutrophils Count (ANC)	≥1,500/mm3 (1.5 x 109/L)
Platelets (PLT)	≥100,000/mm3 (100 x 109/L) independent of platelets transfusion support for at least 7 days prior to dosing
Hemoglobin	≥ 9.0 g/dL independent of transfusion support for at least 7 days prior to dosing
Creatinine Clearance*	> 40 mL/min
Total Serum Bilirubin	<1.5 x ULN
Liver Transaminases (AST/ALT)	<2.5 x ULN; < 5 x ULN if liver metastases are present
Alkaline Phosphatase (ALP)	<2.5 x ULN; < 5 x ULN if liver and/or bone metastasis are present.
Serum calcium, magnesium and potassium	Normal or CTCAE grade ≤ 1 with or without supplementation.

AST/ALT = aspartate aminotransferase/alanine aminotransferase, ULN = upper limit of normal

* calculated by Cockcroft and Gault's formula: (140 - age [yr]) x body weight [Kg] x 1.23 x (0.85 if female) /serum creatinine [µmol/L].

11) Reproductive status: For female and male participants, see Section 5.2 for the full list of criteria.

12) Ability to swallow capsules intact (without chewing, crushing, or opening).

13) Life expectancy ≥ 3 months.

14) Willingness and ability to comply with scheduled visits, treatment plan, laboratory tests, and other study procedures.

Exclusion criteria for Phase 1 and Phase 2:

For country-specific requirements, please refer to APPENDIX 8.

1) Concurrent participation in another therapeutic clinical trial.

2) Symptomatic brain metastases or leptomeningeal involvement.

3) History of previous cancer requiring therapy within the previous 2 years, except for squamous cell or basal-cell carcinoma of the skin, or any in situ carcinoma that has been completely resected.
4) Major surgery within 4 weeks of start of repotrectinib treatment. Radiation therapy (except palliative to relieve bone pain) within 2 weeks of study entry. Palliative radiation (≤10 fractions) must have been completed at least 48 hours prior to study entry.
5) Clinically significant cardiovascular disease (either active or within 6 months prior to enrollment): myocardial infarction, unstable angina, coronary/peripheral artery bypass graft, symptomatic congestive heart failure (New York Heart Association Classification Class ≥ II), cerebrovascular accident or transient ischemic attack, symptomatic bradycardia, requirement for anti-arrhythmic medication. Ongoing cardiac dysrhythmias of CTCAE grade ≥2.
6) Any of the following cardiac criteria:
   a) Mean resting corrected QT interval (ECG interval measured from the onset of the QRS complex to the end of the T wave) for heart rate (QTc) > 470 msec obtained from 3 ECGs, using the screening clinic ECG machine-derived QTc value
   b) Any clinically important abnormalities in rhythm, conduction or morphology of resting ECG (eg, complete left bundle branch block, third degree heart block, second degree heart block, PR interval > 250 msec)
   c) Any factors that increase the risk of QTc prolongation or risk of arrhythmic events such as heart failure, hypokalemia, congenital long QT syndrome, family history of long QT syndrome, or any concomitant medication known to prolong the QT interval (APPENDIX 6)
7) Known active infections requiring ongoing treatment (bacterial, fungal, viral including HIV positivity).
8) Gastrointestinal disease (eg, Crohn's disease, ulcerative colitis, or short gut syndrome) or other malabsorption syndromes that would impact on drug absorption.
9) Peripheral neuropathy, paresthesia, dizziness, dysgeusia, muscle weakness, ataxia grade ≥2.
10) History of extensive, disseminated, bilateral, or presence of CTCAE grade 3 or 4 interstitial fibrosis or interstitial lung disease including a history of pneumonitis, hypersensitivity pneumonitis, interstitial pneumonia, interstitial lung disease, obliterative bronchiolitis, and pulmonary fibrosis. Subjects with history of prior radiation pneumonitis are not excluded.
11) Other severe acute or chronic medical or psychiatric condition or laboratory abnormality that may increase the risk associated with study participation or study drug administration, or that may interfere

	with the interpretation of study results and, in the judgment of the Investigator, would make the subject inappropriate for entry into this study, or could compromise protocol objectives in the opinion of the Investigator and/or the Sponsor.<br>12) Current use or anticipated need for drugs that are known to be strong CYP3A inhibitors or inducers as listed in APPENDIX 6.<br>13) Hypersensitivity to the active substance or to any of the excipients.<br>14) **Additional exclusion criteria for subjects participating in the midazolam DDI sub-study**: in addition to the strong CYP3A inhibitors or inducers listed in APPENDIX 6, subjects should not be taking any moderate inhibitors or inducers of CYP3A (moderate CYP3A inhibitors eg: erythromycin, verapamil, atazanavir, fluconazole, darunavir, diltiazem, delavirdine, aprepitant, imatinib, tofisopam, ciprofloxacin, cimetidine; moderate CYP3A inducers eg:bosentan, efavirenz, etravirine, modafinil) within 2 weeks of the lead-in midazolam dosing and until the DDI assessment portion is completed on Cycle 1 Day 23. Please refer to midazolam product package insert for complete information.
**Dose and Route of Administration**	Repotrectinib will be supplied as 40 mg capsules. Repotrectinib will be taken orally once or twice daily depending on the cohort in Phase 1 and at the RP2D for Phase 2 with or without food.<br>The RP2D for Phase 2 is:<br>• 160 mg QD for the first 14 days, may increase to 160 mg BID. Repotrectinib can be taken with or without food.<br>(Subjects must meet all below criteria while on 160 mg QD prior to dose increasing to 160 mg BID: No grade ≥3 treatment-related AE, unmanageable grade ≥2 dizziness, ataxia or paresthesia; or grade ≥3 clinically significant lab abnormalities).<br>For the midazolam DDI sub-study, midazolam will be supplied as an oral syrup (2 mg/mL). Midazolam will be administered at a dose of 5 mg during the DDI assessment periods on Cycle 1 Day -2, and Cycle 1 Day 22.
**Safety Assessments**	Subjects will be assessed for AEs at each clinic visit while on the study. Adverse events will be graded according to the CTCAE v4.03. Type, incidence, severity, timing, seriousness, and relatedness of AEs and laboratory abnormalities will be reported.
**Data Monitoring Committee**	For Phase 1 portion of the study, safety will be overseen by the Clinical Safety Committee (CSC) comprised of all Investigators and the Medical Monitor of the Study.<br>For the Phase 2 portion of the study, an independent third-party DMC consisting of two medical oncologists and one statistician will monitor the safety for the study. The DMC will meet on a regular basis to

	monitor the overall safety and to conduct an overall benefit-risk assessment. The membership and governance of the DMC will be outlined in a separate charter that will define the rules for early termination, modification and continuation of the study, as well as how those recommendations will be made to the Sponsor.<br>Periodic AE data review will also be performed by designated members of the Sponsor study team. Any safety issues of concern identified by the study team will be promptly reported to the DMC as described in the DMC Charter.
**Pharmacokinetics (PK) Assessments**	PK samples will be obtained from all subjects to determine the systemic exposure to repotrectinib and PK parameters. Analysis of PK samples will be performed at a Good Laboratory Practice (GLP) compliant bioanalytical laboratory designated by the Sponsor. Further information is in Section 7.7.<br>PK assessments for the midazolam DDI sub-study can be found in APPENDIX 7.
**Pharmacodynamics Assessments**	In all subjects, blood samples will be collected during screening, at Cycle 3 Day 1 (in conjunction with tumor assessment evaluation), and either at the time of radiographic disease progression or at the EOT (whichever occurs earlier). Circulating cell -free DNA (ccfDNA) will be isolated from blood to identify genomic alterations in oncogenes and tumor suppressor genes that may predict activity of repotrectinib and to gain insights into potential mechanisms of resistance using next -generation DNA and RNA sequencing and related techniques.
**Endpoints**	Primary Endpoint for Phase 1:<br>MTD and RP2D<br>Secondary Endpoint for Phase 1:<br>ORR assessed by BICR using RECIST Version 1.1<br>Primary Endpoint for Phase 2:<br>Objective Response Rate (ORR) assessed by BICR using RECIST Version 1.1.<br>Secondary Endpoints for Phase 2:<br>• Duration of response (DOR), time to response (TTR), and clinical benefit rate (CBR)<br>• Intracranial tumor response in subjects with measurable brain metastases, as determined by BICR using modified RECIST Version 1.1<br>• CNS progression-free survival (CNS-PFS) in subjects with measurable brain metastases using modified RECIST Version 1.1<br>• Progression-free survival (PFS), and OS<br>• Type, incidence, severity, timing, seriousness, and relatedness of AEs and laboratory abnormalities

	• Pharmacokinetic parameters of repotrectinib<br>• Pharmacokinetic parameters of midazolam (midazolam DDI sub-study only)<br>• Changes in Quality of life and health status during repotrectinib treatment
**Efficacy Assessments**	Subjects will be evaluated for efficacy endpoints ORR, DOR, PFS, per RECIST Version 1.1 that are based on radiologic assessments of disease.<br>The primary endpoint for the Phase 2 study is ORR, as assessed by BICR using RECIST Version 1.1, in each expansion cohort in subjects with advanced solid tumors that harbor a ROS1, NTRK1, NTRK2, or NTRK3 gene rearrangement.
**Duration of Subject Participation and Duration of Study**	Subjects will remain on study treatment until BICR-confirmed radiographic disease progression, development of unacceptable toxicity, or withdrawal of consent.<br>Subjects discontinuing study treatment will enter the survival follow-up period and remain on study until death, loss of follow-up, or withdrawal of consent, whichever comes first. After treatment discontinuation, tumor assessments should continue at the current scan interval at the time of treatment discontinuation until a subject begins a new course of cancer therapy or withdraws consent if there was no BICR-confirmed radiologic progression at the time of treatment discontinuation.<br>If the study is not terminated beforehand per the recommendation of the DMC, the end of trial in all participating countries will be defined as the time at which the secondary endpoint of OS has been met in each expansion cohort. At that time, the protocol may be amended to minimize the number of protocol assessments to only the collection of safety data for those subjects remaining on study.
**Statistical Analysis**	Detailed methodology for summary and statistical analyses of the data collected in this trial will be documented in a Statistical Analysis Plan (SAP). Any major changes in primary endpoint will be documented in the protocol amendment.<br>Sample Size Determination<br>Phase 1 Study<br>The Phase 1 portion is a dose escalation study to determine the MTD or RP2D dose, which consists of 3 stages, plus a DDI sub-study:<br>Phase 1a Dose Escalation<br>For the Dose Escalation phase of the study, cohorts of 3-6 evaluable subjects will be required. The total number of subjects will depend upon the number of dose escalations necessary.<br>Six total dose levels were evaluated (40 mg, 80 mg, 160 mg, 240 mg, 320 mg, and 400 mg/day). If MTD is not reached at the highest dose

level, then assuming 6 subjects at each of the 6 dose levels and expansion to 12 subjects was permitted at the potential RP2D, a maximum of 42 subjects are anticipated to be enrolled.

Phase 1b Food Effect Study

The food effect study commenced at the 40 mg QD dose level where a subject experienced a PR as per Investigator. Six subjects were enrolled at that dose level and subsequent dose levels. An additional 6 subjects enrolled into the food effect study at the RP2D which was assumed to be 160 mg QD. Thus, the minimum number of subjects to enroll into the food effect study will be 12 subjects.

Phase 1c Dose Escalation with Food

For the Phase 1c dose escalation with food phase of the study, cohorts of 3-6 evaluable subjects will be required. The total number of subjects will depend upon the number of DLTs (if any), and if preliminary efficacy is seen within that cohort.

Up to 5 total dose levels will be evaluated. Assuming a maximum of 6 patients at each of the 5 dose levels, approximately 30 patients are anticipated to be enrolled.

DDI Sub-study

To evaluate the potential effect of repotrectinib on CYP3A induction, a MDZ DDI sub-study will be conducted in 6 PK evaluable subjects after RP2D determination.

All subjects will be administered a single 5 mg oral dose of MDZ on Day -2 under fasted conditions (no food 8 hours before through 2 hours after dosing of MDZ), and then starting on Cycle 1 Day 1, subjects will begin daily treatment of 160 mg repotrectinib QD followed by 160 mg BID on Cycle 1 Day 15. On Cycle 1 Day 22, another single dose of 5 mg MDZ will be administered under fasted conditions (no food 8 hours before through 2 hours after dosing of MDZ) concurrently with repotrectinib (morning dose). Serial plasma samples will be collected for up to 24 hours following both MDZ dosing periods to assess PK. Once the DDI assessment periods end, subjects will continue the study treatment until progression of disease, unacceptable toxicity, or consent withdrawal.

Phase 2 Study

The primary objective for each of the six Phase 2 expansion cohorts is to determine the ORR by BICR and their corresponding exact 95% confidence intervals. Confidence intervals will be used for inference purposes.

Below is a summary of approximate subject numbers required for each cohort:

Cohort	Sample Size (N)

EXP-1 ROS1 TKI-Naïve ROS1+ NSCLC	55
EXP-2 1 Prior ROS1 TKI AND 1 Platinum-based Chemotherapy ROS1+ NSCLC	60
EXP-3 2 Prior ROS1 TKIs and NO Chemotherapy or Immunotherapy ROS1+ NSCLC	40
EXP-4 1 Prior ROS1 TKI and NO Chemotherapy or Immunotherapy ROS1+ NSCLC	60
EXP-5 TRK TKI-Naïve NTRK+ Solid Tumors	55
EXP-6 TRK TKI-Pretreated NTRK+ Advanced Solid Tumor	40

Fifty-five subjects were planned to be enrolled into the EXP-1 cohort to show efficacy in this cohort. After enrollment of 55 subjects in the EXP-1 cohort as specified above for the primary analysis, an additional 55 subjects are to be enrolled for a total of 110 subjects in EXP-1.

Sixty subjects were planned to be enrolled into the EXP-2 cohort to show efficacy in this cohort. After enrollment of 60 subjects in the EXP-2 cohort as specified above for the primary analysis, an additional 60 subjects are to be enrolled for a total of 120 subjects in EXP-2.

Forty subjects were planned to be enrolled into the EXP-3 cohort to show efficacy in this cohort. After enrollment of 40 subjects in the EXP-3 cohort as specified above for the primary analysis, an additional 40 subjects are to be enrolled for a total of 80 subjects in EXP-3.

Sixty subjects were planned to be enrolled into the EXP-4 cohort to show efficacy in this cohort. After enrollment of 60 subjects in the EXP-4 cohort as specified above for the primary analysis, an additional 60 subjects are to be enrolled for a total of 120 subjects in EXP-4.

Fifty-five subjects were planned to be enrolled into the EXP-5 cohort to show efficacy in this cohort. After enrollment of 55 subjects in the EXP-5 cohort as specified above for the primary analysis, an additional 25 subjects are to be enrolled for a total of approximately 80 subjects in EXP-5.

Forty subjects were planned to be enrolled into the EXP-6 cohort to show efficacy in this cohort. After enrollment of 40 subjects in the EXP-6 cohort as specified above for the primary analysis, an additional 80 subjects are to be enrolled for a total of approximately 120 subjects in EXP-6.

LIST OF ABBREVIATIONS AND DEFINITION OF TERMS

The following abbreviations and specialist terms are used in this study protocol.

Abbreviation	Definition
AE	Adverse event
AKT	Protein kinase B
ALCL	Anaplastic large cell lymphoma
ALK	Anaplastic lymphoma kinase
ALKi	ALK inhibitor
ALP	Alkaline phosphatase
ALT (SGPT)	Alanine aminotransferase
ANC	Absolute neutrophil count
ASCO	American Society of Clinical Oncology
AST (SGOT)	Aspartate aminotransferase
ATP	Adenosine triphosphate
AUC	Area under curve
$AUC_{(0-t)}$	Area under plasma concentration-time curve from zero to time t
AUC_{inf}	The AUC from time zero to infinity
AUC_{last}	The area under the curve (AUC) from time zero to the last quantifiable concentration point (t_{last})
BBB	Blood-brain barrier
BCRP	Breast cancer resistance protein
BCVA	Best-corrected visual acuity
BICR	Blinded Independent Central Review
BID	Twice a day
BSA	Body surface area
C	Cycle
CBR	Clinical Benefit Rate
ccfDNA	Circulating cell-free DNA
CFR	Code of Federal Regulations
CI	Confidence interval
C_{last}	The observed plasma drug concentration at the last sample collection point
C_{max}	The maximum observed plasma drug concentration
C_{trough}	Observed plasma drug concentration at the end of a dosing interval
CNS	Central nervous system
CNS-PFS	CNS Progression-Free Survival
CPK	Creatine phosphokinase
CR	Complete response
CRF	Clinical Research Form
CSC	Clinical Safety Committee
CSF	Cerebral spinal fluid
CT	Computerized tomography
CTCAE	Common Terminology Criteria for Adverse Events
CYP450	Cytochrome P450
D	Day
DLT	Dose-limiting toxicity
DMC	Data Monitoring Committee

Abbreviation	Definition
DOR	Duration of response
EC	Ethics Committee
ECG	Electrocardiogram
ECOG	Eastern Cooperative Oncology Group
EDC	Electronic data capture
EGFR	Epidermal growth factor receptor
EF score	Emotional functioning score
EIAED	Enzyme-inducing anti-epileptic drug
EMT	Epithelial-mesenchymal transition
EORTC	European Organization for Research and Treatment of Cancer
EOS	End of study
EOT	End of treatment
ERK	Extracellular signal–regulated kinase
EXP	Expansion
FAK	focal adhesion kinase
FAS	Full Analysis Set
FDA	Food and Drug Administration
FFPE	Formalin-fixed paraffin-embedded
FIH	First-in-human
FISH	Fluorescence in situ hybridization
FSH	Follicle-stimulating hormone
GCP	Good Clinical Practice
HNSTD	Highest non-severe toxic dose
HPF	High power field
IC_{50}	Half maximal inhibitory concentration
ICF	Informed consent form
ICH	International Council for Harmonisation
ICMJE	International Committee of Medical Journal Editors
IC-ORR	Intracranial objective response rate
ICR	Institute of Cancer Research
IFU	Instructions for use
IHC	Immunohistochemistry
IMT	Inflammatory myofibroblastic tumor
INR	International normalized ratio
IP	investigational product
IRB	Institutional Review Board
IV	Intravenous
JAK	Janus kinase
LFT	Liver function test
LIC	Lead-In cohort
LVEF	Left ventricular ejection fraction
MASC	Mammary-associated secretory adenocarcinoma
MEC	Molar extinction coefficient
MedDRA	Medical Dictionary for Regulatory Affairs
mEq/L	Milliequivalent per liter
mIU	Milli international unit

Abbreviation	Definition
MRI	Magnetic resonance imaging
MTD	Maximum tolerated dose
MUGA	multigated acquisition
MW	Molecular weight
NA	Not applicable
ND	Not determined
NGS	Next-generation sequencing
NOAEL	No -observed adverse effect level
NSCLC	Non-small-cell lung cancer
NTRK	Neurotrophin receptor kinase
NTRK-1	Neurotrophin receptor kinase 1
NTRK-2	Neurotrophin receptor kinase 2
NTRK-3	Neurotrophin receptor kinase 3
ORR	Objective response rate
OS	Overall survival
PD	Pharmacodynamic
PET	Positron emission tomography
PFS	Progression-free survival
P-gp	P-glycoprotein
PK	Pharmacokinetics
PLT	Platelet
PMDA	Pharmaceuticals and Medical Devices Agency
PO	Orally (By mouth)
PR	Partial response
PRBC	Packed red blood cells
PRO	Patient-reported outcomes
PS	Performance Status
PT	Prothrombin time
PTT	Partial thromboplastin time
QD	Once a day
QLQ	Quality of life questionnaire
QOL	Quality of life
QT	ECG interval measured from the onset of the QRS complex to the end of the T wave
QTc	QT interval corrected for heart rate
QTcB	QT interval corrected for heart rate using Bazett's formula
QTcF	QT interval corrected for heart rate using Fridericia's formula
R_{acc}	Accumulation ratio calculated using AUC_{tau} values at steady-state divided by AUC_{tau} after single dosing
RECIST	Response Evaluation Criteria in Solid Tumors
ROS1	Receptor tyrosine kinase encoded by the *ROS1* gene
ROS1i	ROS1 inhibitor
RP2D	Recommended Phase 2 dose
RR	R wave-to-R wave (RR) interval
RS	Raw score
RTK	Receptor tyrosine kinase
SAE	Serious adverse event
SAP	Statistical analysis plan

Abbreviation	Definition
SCID	Severe combined immunodeficiency
SD	Stable disease
SGOT	Serum glutamic oxaloacetic transaminase
SGPT	Serum glutamic pyruvic transaminase
SRC	Tyrosine kinase first identified in avian sarcoma virus
STAT	Signal transducer and activator of transcription
SOP	Standard operating procedure
SOC	System organ class
STD10	Severely toxic dose in 10% of animals
$t_{1/2}$	Elimination half-life
TEAE	Treatment-emergent adverse event
TKI	Tyrosine kinase inhibitor
T_{max}	The time to reach maximum (peak) plasma drug concentration
TRAE	Treatment-related adverse event
TRK	Tropomyosin receptor kinase
TRKA	Tropomyosin receptor kinase A
TRKB	Tropomyosin receptor kinase B
TRKC	Tropomyosin receptor kinase C
TRKi	Tropomyosin receptor kinase inhibitor
TT	Total testosterone
TTR	Time to response
UGT	Uridine 5'-diphospho-glucuronosyltransferase
ULN	Upper limit of normal
UVB	Ultra Violet –B
Vd	volume of distribution
VEGF	Vascular endothelial growth factor
V_z/F	The apparent volume of distribution during terminal phase
WBRT	Whole brain radiation treatment
WOCBP	Women of childbearing potential
WT	Wild-type
λ_z	Smallest (slowest) disposition (hybrid) rate constant (time-1) may also be used for terminal elimination rate constant

TABLE OF CONTENTS

TITLE PAGE 1
PROTOCOL SYNOPSIS 4
LIST OF ABBREVIATIONS AND DEFINITION OF TERMS 27
TABLE OF CONTENTS 31
LIST OF TABLES 39
LIST OF FIGURES 41
1 INTRODUCTION 42
1.1 Oncogenic RTK Fusions as Driver Mutations in Solid Malignancies 42
1.1.1 ALK-positive (ALK+) Solid Malignancies 42
1.1.1.1 ALK+ NSCLC 42
1.1.1.2 ALK+ Non-NSCLC Solid Malignancies 43
1.1.2 ROS1-positive (ROS1+) Solid Malignancies 44
1.1.2.1 ROS1+ NSCLC 44
1.1.2.2 ROS1+ Non-NSCLC Solid Malignancies 44
1.1.3 NTRK-positive (NTRK+) Solid Malignancies 45
1.2 Disease Progression on TKIs in Oncogenic RTK Driven Solid Malignancies 47
1.2.1 Development of Acquired Resistant Mutations Including Solvent Front Mutations 48
1.2.2 Activation of Bypass Signaling Pathways (SRC, FAK, JAK/STAT) 50
1.2.3 Disease Progression in the Central Nervous System (CNS) 51
2 REPOTRECTINIB 52
2.1 In vitro and *In vivo* Inhibitory Activity of Repotrectinib 52
2.1.1 Activity Against Wild-type (WT) and Mutated ALK 52
2.1.2 Activity Against WT and Mutated ROS1 53
2.1.3 Activity Against Wild-type and Mutated TRK 53
2.1.4 Activity Against Solvent Front Mutations of ALK, ROS1 and TRKA/B/C 54
2.1.5 Activity Against SRC, FAK and JAK2 for Modulation of Bypass Signaling, EMT, Cancer Stemness and Metastasis 55
2.2 Repotrectinib Pre-Clinical Safety Data 56
2.3 Benefit to Risk Ratio Assessment for First-in-Human Study 57
2.4 Safety Consideration in the Clinical Trial 58
2.5 Repotrectinib Pharmacokinetics in Animals and Projection of Human Pharmacokinetics 58
2.6 Rationale for Selection of the Starting Dose 59
2.7 Preliminary Safety and Efficacy Data from the TPX-0005-01 Study 60
2.8 Rationale for the Recommended Phase 2 Dose Selection 60
2.9 Preliminary Pharmacokinetic Data 61
2.9.1 Study TPX-0005-09: Mass Balance, Pharmacokinetics, and Metabolism of Repotrectinib in Healthy Male Subjects 63
3 STUDY DESIGN 63
3.1 Study Overview 63
3.2 Phase 1a and Phase 1c Dose Escalation 64
3.2.1 Starting Dose Level for Dose Escalation Phases: Phase 1a and Phase 1c 64
3.2.2 Dose Escalation Levels 65
3.2.3 Criteria for Dose Escalation and Determination of MTD 66

3.2.3.1 Maximum Tolerated Dose (MTD) Definition ... 66
3.2.3.2 Dose escalation and MTD Determination Process: (Phase 1a and Phase 1c) ... 67
3.2.3.3 DLT Definition ... 68
3.2.3.4 Follow-up After a Dose-Limiting Toxicity ... 70
3.2.3.5 Dose Modifications: Intra-subject dose escalation/reductions, and re-escalation criteria (for Phase 1 only) ... 71
3.2.3.6 Recommended Phase 2 Dose (RP2D) Definition ... 72
3.3 Repotrectinib Phase 1b Food Effect Subgroup ... 73
3.4 Midazolam Drug-Drug Interaction Sub-study ... 73
3.5 Phase 2 Study ... 74
3.5.1 ROS1+ Solid Malignancy Expansion Cohorts ... 74
3.5.1.1 ROS1+ NSCLC Expansion Cohorts ... 74
3.5.2 NTRK+ Solid Malignancy Expansion Cohorts ... 75
3.6 End of Study Definition ... 78
4 STUDY OBJECTIVES ... 78
4.1 Phase 1 Objectives ... 78
4.1.1 Phase 1 Primary Objectives ... 78
4.1.2 Phase 1 Secondary Objectives ... 78
4.1.3 Phase 1 Exploratory Objectives ... 78
4.2 Phase 2 Objectives ... 79
4.2.1 Phase 2 Primary Objectives ... 79
4.2.2 Phase 2 Secondary Objectives ... 79
4.2.3 Phase 2 Exploratory Objectives ... 79
5 SUBJECT SELECTION ... 79
5.1 Inclusion Criteria for Phase 1 ... 79
5.2 Inclusion Criteria for Phase 2 ... 83
5.3 Exclusion Criteria for Phase 1 and Phase 2 ... 89
6 STUDY TREATMENTS ... 90
6.1 Repotrectinib Drug Supply ... 90
6.1.1 Formulation and Packaging ... 90
6.1.2 Preparation and Dispensing ... 90
6.1.3 Administration ... 90
6.1.3.1 Dose Administration (Phase 1) ... 90
6.1.3.2 Dose Administration (Phase 2) ... 92
6.1.4 Storage and Accountability ... 93
6.1.4.1 Temperature Excursions ... 93
6.2 Dose Modifications ... 94
6.2.1 Recommendation for Dose Modifications ... 94
6.2.2 Dose Interruptions ... 94
6.2.3 Dose Reductions ... 95
6.2.3.1 Dose Modification for Phase 1: ... 95
6.2.4 Dose Modification for Phase 2: ... 96
6.3 Concomitant Medications ... 99
6.3.1 Strong CYP3A4 Inhibitors and Inducers and Sensitive Substrates of CYP3A4/CYP2B6 ... 99
6.3.1.1 Medications Known to be Strong CYP3A4 Inhibitors and Inducers ... 99

6.3.1.2 Medications Known to be Sensitive CYP3A4 and CYP2B6 Substrates 99
6.3.2 Medications Known to Prolong the QT Interval 100
6.3.3 Other Anti-tumor or Investigational Drugs 101
6.3.4 Seizure Prophylaxis 101
6.3.5 Hematopoietic Growth Factors 101
6.3.6 Anti-diarrheal and Anti-emetic Therapy 102
6.3.7 Anti-inflammatory Therapy 102
6.3.8 Cutaneous Toxicity Therapy 102
6.3.9 Testosterone Replacement 102
6.3.10 Dizziness Prophylaxis 102
6.4 Other Study Restrictions 102
6.4.1 Surgery 102
6.4.2 Palliative Radiation 102
6.4.3 Supportive Care 103
6.4.4 Lifestyle Guidelines: Contraception 103
7 STUDY PROCEDURES 103
7.1 Clinical Assessments 103
7.1.1 Main Informed Consent 103
7.1.2 Pre-screening Informed Consent (Phase 2 only) 103
7.1.3 Demographics 103
7.1.4 Medical History 104
7.1.5 Physical Examination 104
7.1.5.1 Neurological Examination 104
7.1.6 Vital Signs 104
7.1.7 Performance Status 104
7.1.8 Laboratory Assessments 104
7.1.9 Adverse Events 105
7.1.10 Concomitant Medications and Treatments 105
7.1.11 Subject Dosing Diary 105
7.1.12 Ophthalmologic Examination 105
7.2 Tumor Assessments 105
7.2.1 Blinded Independent Central Review (BICR) 107
7.3 Tumor Procurement 107
7.3.1 Archival Tumor Tissue 107
7.3.2 Fresh Tumor Biopsy 108
7.3.3 Liquid Biopsies 108
7.4 Clinical Laboratory Assessments 109
7.5 Electrocardiogram (ECG) 109
7.6 Echocardiogram/MUGA Scan 109
7.7 Pharmacokinetic (PK) Assessments 109
7.7.1 PK Collection Times Points 111
7.7.1.1 Blood for PK Analysis of Repotrectinib (Phase 1a Dose Escalation) 111
7.7.1.2 Blood for PK Analysis of Repotrectinib (Phase 1b Food Effect) 112
7.7.1.3 Blood for PK Analysis of Repotrectinib (Phase 1c Dose Escalation) 116
7.7.1.4 Blood for PK Analysis of Midazolam and Repotrectinib (Midazolam DDI Sub-study) 117

7.7.1.5 *Blood for PK Analysis of Repotrectinib (Phase 2)* 117
7.8 Pharmacodynamic (PD) Assessment 120
7.8.1 *On-treatment Tumor Biopsy* 120
7.9 Patient-Reported Outcomes (PRO) 120
8 STUDY ASSESSMENTS BY VISIT 120
8.1 Phase 1a Dose Escalation 120
8.1.1 *Screening Phase 1a Dose Escalation (within 28 days of the first dose of repotrectinib)* 120
8.1.2 *Lead-in PK Cycle 0 Day -3 (Phase 1a Dose Escalation)* 121
8.1.3 *Cycle 1 Day 1 (Phase 1a Dose Escalation)* 122
8.1.4 *Cycle 1 Day 8 (Phase 1a Dose Escalation)* 122
8.1.5 *Cycle 1 Day 15 (Phase 1a Dose Escalation)* 123
8.1.6 *Cycle 2 Day 1 (Phase 1a Dose Escalation)* 123
8.1.7 *Cycle 2 Day 15 (Phase 1a Dose Escalation)* 123
8.1.8 *Cycle 3 Day 1 (Phase 1a Dose Escalation)* 124
8.1.9 *Cycle 4 Day 1 (Phase 1a Dose Escalation)* 124
8.1.10 *Cycle 5 and Every 4 Weeks Thereafter at Each Cycle Day 1 Visit (Phase 1a Dose Escalation)* 125
8.1.11 *End of Treatment (Phase 1a Dose Escalation) (within 7 days post last dose of repotrectinib and after decision to end treatment)* 125
8.1.12 *Safety Follow-up (Phase 1a Dose Escalation) (approximately 28 days post the last dose of repotrectinib)* 126
8.1.13 *Survival Follow-up (Phase 1a Dose Escalation)* 126
8.2 Phase 1b Food Effect 126
8.2.1 *Screening Phase 1b Food Effect (within 28 days of the first dose of repotrectinib)* 126
8.2.2 *Lead-in PK Cycle 0 Day -7 (Phase 1b Food Effect): applicable to Cohorts 1-3 only* 128
8.2.3 *Cycle 1 Day 1 (Phase 1b Food Effect): applicable to Cohorts 1-3 only* 129
8.2.4 *Cycle 1 Day 8 (Phase 1b Food Effect): applicable to Cohorts 1-3 only* 130
8.2.5 *Cycle 1 Day 15 (Phase 1b Food Effect): applicable to Cohorts 1-3 only* 130
8.2.6 *Cycle 2 Day 1 (Phase 1b Food Effect): applicable to Cohorts 1-3 only* 131
8.2.7 *Cycle 2 Day 15 (Phase 1b Food Effect): applicable to Cohorts 1-3 only* 131
8.2.8 *Cycle 3 Day 1 (Phase 1b Food Effect): applicable to Cohorts 1-3 only* 131
8.2.9 *Cycle 4 Day 1 (Phase 1b Food Effect): applicable to Cohorts 1-3 only* 132
8.2.10 *Cycle 5 and Every 4 Weeks Thereafter at Each Cycle Day 1 Visit (Phase 1b Food Effect): applicable to Cohorts 1-3 only* 132
8.2.11 *End of Treatment (Phase 1b Food Effect) (within 7 days post last dose of repotrectinib and after decision to end treatment)* 133
8.2.12 *Safety Follow-up (Phase 1b Food Effect) (approximately 28 days post the last dose of repotrectinib)* 133
8.2.13 *Survival follow-up (Phase 1b Food Effect)* 134
8.3 Phase 1c Study (Cohort 7-9) 134
8.3.1 *Screening Phase 1c Dose Escalation (within 28 days of the first dose of repotrectinib)* 134

8.3.2 *Cycle 1 Day 1 (Phase 1c Dose Escalation)* 135
8.3.3 *Cycle 1 Day 8 (Phase 1c Dose Escalation)* 136
8.3.4 *Cycle 1 Day 15 (Phase 1c Dose Escalation)* 136
8.3.5 *Cycle 1 Day 22 (Phase 1c Dose Escalation)* 136
8.3.6 *Cycle 2 Day 1 (Phase 1c Dose Escalation)* 137
8.3.7 *Cycle 2 Day 15 (Phase 1c Dose Escalation)* 137
8.3.8 *Cycle 3 Day 1 (Phase 1c Dose Escalation)* 137
8.3.9 *Cycle 4 Day 1 (Phase 1c Dose Escalation)* 138
8.3.10 *Cycle 5 and Every 4 Weeks Thereafter at Each Cycle Day 1 Visit (Phase 1c Dose Escalation)* 139
8.3.11 *End of Treatment (Phase 1c Dose Escalation) (within 7 days post last dose of repotrectinib and after decision to end treatment)* 139
8.3.12 *Safety Follow-up (Phase 1c Dose Escalation) (approximately 28 days post the last dose of repotrectinib)* 140
8.3.13 *Survival Follow-up (Phase 1c Dose Escalation)* 140
8.4 Phase 2 Study 140
8.4.1 *Screening Phase 2 Study* 140
8.4.2 *Re-Screening* 142
8.4.3 *Cycle 1 Day 1 (Phase 2 Study)* 143
8.4.4 *Cycle 1 Day 8 (Phase 2 Study)* 143
8.4.5 *Cycle 1 Day 15 (Phase 2 Study)* 143
8.4.6 *Cycle 1 Day 22 (Phase 2 Study)* 144
8.4.7 *Cycle 2 Day 1 (Phase 2 Study)* 144
8.4.8 *Cycle 2 Day 15 (Phase 2 Study)* 145
8.4.9 *Cycle 3 Day 1 (Phase 2 Study)* 145
8.4.10 *Cycle 4 Day 1 (Phase 2 Study)* 146
8.4.11 *Cycle 5 and Every 4 Weeks Thereafter at Each Cycle Day 1 Visit (Phase 2 Study)* 146
8.4.12 *End of Treatment (Phase 2 Study) (within 7 days post last dose of repotrectinib and after decision to end treatment)* 147
8.4.13 *Safety Follow-up (Phase 2 Study) (approximately 28 days post the last dose of repotrectinib)* 148
8.4.14 *Survival Follow-up (Phase 2 Study)* 148
8.5 Study Calendars 149
9 SAFETY ASSESSMENTS 178
9.1 Adverse Events 178
9.1.1 *Serious Adverse Events (Immediately Reportable to the Sponsor)* 178
9.2 Methods and Timing for Capturing and Assessing Safety Parameters 179
9.2.1 *Adverse Event Reporting Period* 179
9.2.2 *Eliciting Adverse Event Information* 179
9.2.3 *Assessment of Severity of Adverse Events* 179
9.2.4 *Assessment of Causality of Adverse Events* 180
9.2.5 *Procedures for Recording Adverse Events* 181
9.2.5.1 *Diagnosis versus Signs and Symptoms* 181
9.2.5.2 *Adverse Events That Are Secondary to Other Events* 181
9.2.5.3 *Persistent or Recurrent Adverse Events* 182

9.2.5.4 *Abnormal Laboratory Values* ... 182
9.2.5.5 *Abnormal Vital Sign Values* ... 183
9.2.5.6 *Abnormal Liver Function Tests* ... 183
9.2.5.7 *Deaths* ... 184
9.2.5.8 *Preexisting Medical Conditions* ... 185
9.2.5.9 *Lack of Efficacy or Worsening of the Malignancy under Study* ... 185
9.2.5.10 *Hospitalization or Prolonged Hospitalization* ... 185
9.2.5.11 *Occupational Exposure* ... 186
9.2.5.12 *Adverse Events Associated with an Overdose or Incorrect Administration of Study Drug* ... 186
9.3 Immediate Reporting Requirements from Investigator to the Sponsor ... 186
9.3.1 *SAE Reporting* ... 187
9.3.2 *Pregnancy* ... 187
9.4 Follow-Up of Subjects After Adverse Events ... 187
9.4.1 *Investigator Follow-Up* ... 187
9.4.2 *Sponsor Follow-Up* ... 188
9.5 Post-Study Adverse Events ... 188
9.6 Expedited Reporting to Health Authorities, Investigators, Institutional Review Boards, and Ethics Committees ... 188
10 SUBJECT END OF TREATMENT ... 188
11 PROTOCOL DEVIATIONS ... 189
12 DATA MONITORING COMMITTEE ... 190
13 STATISTICAL METHODS AND CONSIDERATIONS ... 190
13.1 Analysis Populations (Sets) ... 190
13.1.1 *Full Analysis Set* ... 190
13.1.2 *Safety Analysis Set* ... 191
13.1.3 *Per Protocol Analysis Set (Phase 1)* ... 191
13.1.4 *PK Concentration Analysis Set* ... 191
13.1.4.1 *Repotrectinib* ... 191
13.1.4.2 *Midazolam (MDZ DDI sub-study only)* ... 191
13.1.5 *PK Parameter Analysis Set* ... 191
13.1.5.1 *Repotrectinib* ... 191
13.1.5.2 *Midazolam (MDZ DDI sub-study only)* ... 192
13.2 Efficacy Analyses ... 192
13.2.1 *Analysis of Primary Endpoint* ... 192
13.2.1.1 *Analysis of Primary Endpoint (Phase 1)* ... 192
13.2.1.2 *Analysis of Primary Endpoint (Phase 2)* ... 192
13.2.2 *Analysis of Secondary Endpoints* ... 193
13.2.2.1 *Analysis of Secondary Endpoints (Phase 1)* ... 193
13.2.2.2 *Analysis of Secondary Endpoints (Phase 2)* ... 193
13.3 Statistical Analysis Plan (SAP) and Sample Size Justification ... 195
13.3.1 *Phase 1 Dose Escalation and Food Effect Cohorts* ... 195
13.3.1.1 *Phase 1a Dose Escalation* ... 195
13.3.1.2 *Phase 1b Food Effect Study* ... 195
13.3.1.3 *Phase 1c Dose Escalation with Food* ... 195
13.3.2 *Phase 2 Study* ... 195

13.3.2.1 EXP-1 ROS1 TKI-Naïve ROS1+ NSCLC 195
13.3.2.2 EXP-2 1 Prior ROS1 TKI AND 1 Platinum-based Chemotherapy ROS1+ NSCLC 196
13.3.2.3 EXP-3 2 Prior ROS1 TKI and NO Chemotherapy or Immunotherapy ROS1+ NSCLC 197
13.3.2.4 EXP-4 1 Prior ROS1 TKI and NO Chemotherapy or Immunotherapy ROS1+ NSCLC 197
13.3.2.5 EXP-5 TRK TKI-Naïve NTRK+ Solid Tumors 198
13.3.2.6 EXP-6 TRK TKI-Pretreated NTRK+ Advanced Solid Tumors 198
13.4 Safety Analyses 199
13.4.1 Extent of Exposure 199
13.4.2 Adverse Events 199
13.4.3 Clinical Laboratory Results 200
13.4.4 Vital Signs 200
13.4.5 Concomitant Medications/Treatment 200
13.4.6 ECG 200
13.4.7 Left Ventricular Ejection Fraction (LVEF) 201
13.4.8 Ophthalmologic Assessment 201
13.5 Analysis of Pharmacokinetics 201
13.5.1 Single- and Multiple-Dose Repotrectinib Pharmacokinetic Analysis 201
13.5.1.1 Effect of Food on Repotrectinib Pharmacokinetics 202
13.5.1.2 Effect of Repotrectinib on MDZ Pharmacokinetics 202
13.5.1.3 Population Pharmacokinetic Analysis or PK/PD Modeling 202
13.6 Subject-Reported Outcomes (PROs) 202
13.6.1 Administration of PROs 203
13.6.2 EORTC-QLQ-C30 204
13.6.2.1 Technical Summary 205
13.6.2.2 Linear Transformation 205
13.6.2.3 Missing Items for QLQ-C30 205
13.6.3 EORTC-QLQ-LC13 205
13.6.4 QOL Compliance Rates 206
13.6.5 PRO Endpoints 206
13.6.6 PRO Analyses 207
13.6.6.1 Change from Baseline Scores 207
13.6.6.2 Proportion of Subjects Improved, Remained Stable, or Worsened 207
13.7 Interim Analysis 207
14 DATA COLLECTION, RETENTION AND MONITORING 208
14.1 Data Collection Instruments 208
14.2 Data Management Procedures 208
14.3 Data Quality Control and Reporting 208
14.4 Data Archival 208
14.5 Availability and Retention of Investigational Records 208
14.6 Monitoring 209
14.7 Subject Confidentiality 209
15 ADMINISTRATIVE, ETHICAL, REGULATORY CONSIDERATIONS 210
15.1 Protocol Amendments 210

15.2 Institutional Review Boards/Ethic Committees 210
15.3 Reporting of Safety Issues and Serious Breaches of the Protocol or ICH GCP 211
15.4 Sponsor Discontinuation Criteria 211
15.5 Post-Trial Access 211
15.6 Publications 212
16 REFERENCES 212
APPENDIX 1 CONTRACEPTIVE GUIDELINES 220
APPENDIX 2 PHASE 1 REQUIRED LABORATORY ASSESSMENTS 223
APPENDIX 3 PHASE 2 REQUIRED LABORATORY ASSESSMENTS 225
APPENDIX 4 SUBJECT-REPORTED OUTCOMES: EORTC-QLQ-C30..................227
APPENDIX 5 SUBJECT-REPORTED OUTCOMES: EORTC-QLQ-LC13 229
APPENDIX 6 TABLE OF MEDICATIONS THAT ARE STRONG CYP3A INHIBITORS OR INDUCERS OF CYP3A OR CAUSE QTC PROLONGATION...230
APPENDIX 7 MIDAZOLAM DRUG-DRUG INTERACTION SUB-STUDY............232
APPENDIX 8 COUNTRY SPECIFIC REQUIRMENTS / DIFFERENCES 249
APPENDIX 9 SUMMARY OF CHANGES 254

LIST OF TABLES

Table 1: List of ALK Fusions in Non-NSCLC Solid Malignancies 43
Table 2: List of ROS1 Fusions in Non-NSCLC Solid Malignancies 45
Table 3: List of NTRK Fusions in Solid Malignancies 46
Table 4: ALK/ROS1/TRK Inhibitors and Clinical Resistant Mutations 48
Table 5: The Activities of Repotrectinib against WT and Solvent Front Mutated ALK, ROS1 and TRKs 54
Table 6: Summary of Geometric Mean (%CV) Pharmacokinetic Parameters of Repotrectinib After a Single Oral Dose of Repotrectinib Under Fasted Conditions 61
Table 7: Summary of Geometric Mean (%CV) Pharmacokinetic Parameters of Repotrectinib After Single Ascending Oral Doses of Repotrectinib Under Fasted Conditions on Day 15 (Steady-State) 62
Table 8: Provisional Dose Levels (QD under fasted condition): Phase 1a and Phase 1b 65
Table 9: Provisional Dose Levels (BID under fasted condition): Phase 1a 66
Table 10: Provisional Dose Levels (QD & BID with food): Phase 1c 66
Table 11: Criteria for Defining Dose-limiting Toxicities 69
Table 12: Recommended Dose Modifications for Phase 1 71
Table 13: Recommended Dose Reduction Guidance for Phase 2 96
Table 14: Dose Modifications for Adverse Events (CTCAE version 4.03) 97
Table 15: List of Enzyme-inducing Anti-epileptic Drugs and Non-enzyme-inducing Anti-epileptic Drugs 101
Table 16: Noncompartmental Pharmacokinetic Parameters 110
Table 17: Time Points of Blood Collection for Repotrectinib PK Assessment during Phase 1a Dose Escalation Phase 111
Table 18: Time Points of Blood Collection for Repotrectinib PK Assessment during Phase 1b Food Effect Study (FED Followed by FASTED) 113
Table 19: Time Points of Blood Collection for Repotrectinib PK Assessment during Phase 1b Food Effect Study (FASTED Followed by FED) 114
Table 20: Time Points of Blood Collection for Repotrectinib PK Assessment during Phase 1c Dose Escalation Phase 116
Table 21: Time Points of Blood Collection of Repotrectinib PK Assessment during Phase 2 (Adult Subjects age 18 Years and Older) 118
Table 22: Time Points of Blood Collection of Repotrectinib PK Assessment during Phase 2 (Adolescent Subjects Age 12-17 Years) 118

Table 23: Study Calendar for Phase 1a Dose Escalation Portion of TPX-0005-01 149
Table 24: Study Calendar for Phase 1b Food Effect Portion of TPX-0005-01 (Applicable for Cohorts 1-3 ONLY) 156
Table 25: Study Calendar for Phase 1c Study of TPX-0005-01 163
Table 26: Study Calendar for Phase 2 Study of TPX-0005-01 170
Table 27: Grading of Severity of Adverse Events 180
Table 28: Causation Attribution 181
Table 29: Estimated ORRs and 95% Confidence Intervals Using Sample Size N = 55 for EXP-1 196
Table 30: Estimated ORRs and 95% Confidence Intervals Using Sample Size N = 60 for EXP-2 196
Table 31: Estimated ORRs and 95% Confidence Intervals Using Sample Size N = 40 for EXP-3 197
Table 32: Estimated ORRs and 95% Confidence Intervals Using Sample Size N = 60 for EXP-4 197
Table 33: Estimated ORRs and 95% Confidence Intervals Using Sample Size N = 55 for EXP-5 198
Table 34: Estimated ORRs and 95% Confidence Intervals using Sample Size N = 40 for EXP-6 199
Table 35: Subject-Reported Outcome QOL Instruments 203
Table 36: Time Points of Blood Collection for Midazolam Pharmacokinectic Assessment 233
Table 37: Time Points of Blood Collection for Repotrectinib PK Assessment (Midazolam DDI Sub-study) 234
Table 38: Study Calendar for Midazolam Drug-Drug Interaction Sub-study 242

LIST OF FIGURES

Figure 1: The Anti-tumor Efficacy of Repotrectinib in WT and Solvent Front Mutated ALK, ROS1 and TRKA Tumor Models 55

Figure 2: Schema of TPX-0005-01 Study 77

1 INTRODUCTION

1.1 Oncogenic RTK Fusions as Driver Mutations in Solid Malignancies

There are 58 human receptor tyrosine kinases (RTKs) that can be divided into 20 subfamilies (Blume-Jensen 2001). Rearrangement in many of the RTKs that retains the intact kinase domain are now known to be actionable oncogenic driver mutations in epithelial malignancies (Shaw 2013a). In particular, non-small-cell lung cancer (NSCLC) harboring rearrangement in the anaplastic lymphoma kinase (ALK) gene serves as a paradigm for management of actionable oncogenic drivers.

1.1.1 ALK-positive (ALK+) Solid Malignancies

1.1.1.1 ALK+ NSCLC

Lung cancer is the leading cause of cancer diagnosis with an estimated of 1.82 million new lung cancer cases diagnosed in 2012 (Torre 2015). Additionally, lung cancer is the leading cause of cancer-related death globally, with an estimated 1.59 million patients who will die of lung cancer per estimates for the year 2012 (Torre 2015). Improvements in biologic understanding have led to the identification of multiple therapeutic molecular targets in NSCLC. Large-scale comprehensive molecular profiling studies have identified multiple actionable driver mutations in NSCLC including oncogenic fusions involving the RTKs ALK and ROS1 (Barlesi 2016; Kris 2014). Recently, uncommon oncogenic fusions involving the neurotrophin receptor kinase 1 (*NTRK1*) (Vaishnavi 2013) and neurotrophin receptor kinase 2 (*NTRK2*) rearrangements have been reported in NSCLC (Stransky 2014).

ALK is one of 58 human RTKs (Blume-Jensen 2001). *ALK* rearrangement (*ALK*+) in NSCLC was first reported in 2007 (Rikova 2007; Soda 2007) and the clinical benefit of targeting oncogenic ALK fusions has led to the approval of several ALK inhibitors in the United States: crizotinib (Xalkori®) on August, 26, 2011 (Malik 2014), ceritinib (Zykadia®) on April 29, 2015 (Khozin 2015), alectinib (Alecensa® on December 11, 2015) and brigatinib (Alunbrig® on May 19, 2017). Two large randomized studies have compared crizotinib to chemotherapy (Shaw 2013b; Solomon 2014). In previously untreated *ALK*-positive metastatic NSCLC patients, crizotinib has achieved superior objective response rates (ORRs) of 74% compared to 45% and superior progression-free survival (PFS) (10.9 months compared to 7.3 months; HR 0.45 (95% confidence interval (CI): 0.35, 060); p-value < 0.001). Crizotinib achieved full approval as second-line treatment of *ALK*+ metastatic NSCLC patients after progression on first-line platinum-based chemotherapy based on the statistically superior PFS compared to single-agent chemotherapy of 7.7 months compared to 3.0 months; HR 0.49 (95% CI: 0.37, 0.64); p-value < 0.001 (Kazandjian 2014).

Ceritinib, a more potent second-generation ALK inhibitor, has demonstrated an ORR of 43% with a median duration of response (DOR) of 7 months by blinded independent review committee (BICR) in *ALK*+ metastatic NSCLC patients who progressed or were intolerant of crizotinib (Khozin 2015; Shaw 2014a). Alectinib, another second-generation ALK inhibitor, demonstrated ORRs of 50% and 48% and DOR of 11.2 months and 13.0 months in both a global and a North American Phase 2 study respectively in *ALK*+ NSCLC patients who progressed or intolerant of crizotinib (Ou 2016; Shaw 2016a). The clinical activity of brigatinib, another potent second-

generation ALK inhibitor was investigated in a randomized Phase 2 study comparing 2 dosing strategies (90 mg once daily compared to 180 mg once daily following a 7-day lead-in at 90 mg once daily) in *ALK+* locally advanced or metastatic NSCLC patients who progressed on or were intolerant of crizotinib. Brigatinib given orally at 90 mg once daily for the first 7 days and then escalated to 180 mg once daily was the superior arm and achieved an ORR of 54% (95% CI: 43% – 62%) and DOR of 13.8 months (9.3-not estimable) (Kim 2016a). Despite the multiple available therapies for *ALK+* NSCLC, resistant mutations develop and remain an unmet medical need to address.

1.1.1.2 ALK+ Non-NSCLC Solid Malignancies

Besides the rearrangement in anaplastic large cell lymphoma (ALCL) where ALK derived its namesake, *ALK* rearrangement has been identified in many epithelial malignancies (Table 1).

Table 1: List of ALK Fusions in Non-NSCLC Solid Malignancies

Tumor	ALK fusions	Reference
Bladder cancer	TPM1-ALK	Stransky 2014
Breast cancer	EML4-ALK	Lin 2009
Colorectal adenocarcinoma	EML4-ALK	Lin 2009
	C2orf44-ALK	Lipson 2012
	CAD-ALK	Lee 2015
	SPTBN1-ALK	Ying 2015
Inflammatory Myofibroblastic Tumor (IMT)	CLTC-ALK<br>FN1-ALK<br>EML4-ALK<br>PRKAR1A-ALK<br>RANB2-ALK<br>SEC31A-ALK<br>TFG-ALK<br>TPM3-ALK<br>TPM4-ALK	Lovly 2014
Pigmented spindle cell nevus/Spitz nevus	DCTN1-ALK<br>TPM3-ALK	Wiesner 2014
Rectal adenocarcinoma	SMEK2-ALK	Stransky 2014
Renal carcinoma	VCL-ALK	Debelenko 2011
Renal medullary carcinoma	VCL-ALK	Mariño-Enriquez 2011
Thyroid cancer	GTF2IRD1-ALK	Stransky 2014

Table 1: List of ALK Fusions in Non-NSCLC Solid Malignancies

Tumor	ALK fusions	Reference
Thyroid cancer (anaplastic)	STRN-ALK	Perot 2014
Thyroid cancer (medullary)	GFPT1-ALK	Ji 2015
	EML4-ALK	Demeure 2014

1.1.2 ROS1-positive (ROS1+) Solid Malignancies

1.1.2.1 ROS1+ NSCLC

Crizotinib (Xalkori®) and entrectinib (Rozlytrek™) are Food and Drug Administration (FDA) approved kinase inhibitors indicated for patients with metastatic *ROS1+* NSCLC. ROS1 is another one of the 58 human RTKs (Blume-Jensen 2001) and shares extensive amino acid homology with ALK in the kinase domain (Ou 2012). *ROS1* rearrangement in NSCLC was discovered in 2007 (Rikova 2007) and shares similar clinicopathologic characteristics with *ALK*-positive NSCLC (Bergethon 2012). Given the extensive homology between ALK and ROS1, crizotinib is a ROS1 inhibitor and has demonstrated significant clinical activity with an ORR of 73% and a median PFS of 19.2 months as a single agent in 50 *ROS1+* metastatic NSCLC patients (Shaw 2014b). Independent radiologic review of the study revealed a confirmed ORR of 66% and DOR of 18.3 months (Kazandjian 2016). Based on this significant clinical activity of crizotinib in *ROS1+* NSCLC patients, crizotinib was approved by the US FDA for the treatment of metastatic *ROS1+* NSCLC patients on March 11, 2016 (Kazandjian 2016). Additionally, a Phase 2 study of crizotinib in *ROS1+* NSCLC patients in Asia confirmed the high ORR of 69.3% observed in the US study and the median PFS of 13.4 months (Goto 2016). More importantly, the ORR to crizotinib was independent of the prior number of chemotherapy regimens. The ORR was 75% in patients with no prior chemotherapy, 66% in patients who received 1 prior chemotherapy regimen, 63.3% in patients who received 2 prior chemotherapy regimens, and 80.8% in patients who received 3 or more prior chemotherapy regimens. Similar to the patients with *ALK+* NSCLC, acquired resistant *ROS1* mutations developed in *ROS1+* NSCLC patients during treatment with crizotinib. Solvent front mutations in *ROS1* such as G2032R (Awad 2013; Song 2015) and D2033N (Drilon 2016a) seemed to be the dominant acquired resistant mutations to crizotinib.

Entrectinib was evaluated in 51 patients with *ROS1+* NSCLC, Eastern Cooperative Oncology Group (ECOG) performance status (PS) ≤ 2, measurable disease per RECIST Version 1.1, ≥ 12 months of follow-up from first post-treatment tumor assessment, and no prior therapy with a ROS1 inhibitor. Objective response rate, as assessed by BICR, was 78% and the percent of patients with a duration of response (DOR) of ≥ 9 months, ≥12 months and ≥18 months was 70%, 55%, and 30%, respectively (ROZLYTREK™ USPI, 2019).

1.1.2.2 ROS1+ Non-NSCLC Solid Malignancies

Similar to *ALK* rearrangements, *ROS1* rearrangements have been identified in many non-NSCLC solid malignancies. Importantly, patients with *ROS1*-rearranged inflammatory myofibroblastic

tumor (IMT) have shown responses to crizotinib, and yet remain an area of unmet medical need (Lovly 2014). Listed below are the published ROS1 fusions in non-NSCLC solid malignancies (Table 2).

Table 2: List of ROS1 Fusions in Non-NSCLC Solid Malignancies

Tumor	ROS1 fusions	Reference
Angiosarcoma	CEP85L-ROS1	Giacomini 2013
Anaplastic large cell lymphoma	NFkB2-ROS1<br>NCOR-ROS1	Crescenzo 2015
Cholangiocarcinoma	GOPC-ROS1 (FIG-ROS1)	Gu 2011
Colorectal adenocarcinoma	SLC34A2-ROS1	Aisner 2014
Gastric adenocarcinoma	SLC34A2-ROS1	Lee 2013
Glioblastoma Multiforme	GOPC-ROS1 (FIG-ROS1)	Charest 2003
Inflammatory Myofibroblastic Tumor (IMT)	TFG-ROS1<br>YWHAE-ROS1	Lovly 2014
Pigmented spindle cell nevus/Spitz nevus	CLIP1-ROS1<br>ERC1-ROS1<br>HLA-A-ROS1<br>KIAA1598-ROS1<br>MYO5A-ROS1<br>PWWP2A-ROS1<br>ZCCHC8-ROS1	Wiesner 2014

1.1.3 NTRK-positive (NTRK+) Solid Malignancies

Tropomyosin receptor kinase (TRK) encoded by neurotrophin receptor kinase (*NTRK*) gene belongs to the TRK sub-family among the 58 members of the human RTK (Blume-Jensen 2001). There are 3 members of TRK family: TRKA, TRKB, and TRKC encoded by *NTRK1*, *NTRK2*, and *NTRK3*, respectively. The transforming activity of the TRKA kinase was first discovered as the transforming kinase partner to tropomyosin gene in a colorectal cancer tumor sample (Martin-Zanca 1986). Subsequently TRKA was identified to be the receptor for nerve growth factor (Kaplan 1991; Klein 1991). TRKB (Squinto 1991) and TRKC (Lamballe 1991) were discovered later. All 3 members share significant amino acid homology especially among the kinase domains (Barbacid 1995). Oncogenic rearrangements of *NTRK1*, *NTRK2*, and *NTRK3* that lead to function TRK fusion proteins have subsequently been identified in many solid malignancies (Vaishnavi 2015) (Table 3).

Table 3: List of NTRK Fusions in Solid Malignancies

Tumor type	*NTRK* fusion type	Reference
Colorectal adenocarcinoma	TPM3-NTRK1	Martin-Zanca 1986; Ardini 2014
Colorectal adenocarcinoma	TPR-NTRK1	Créancier 2015
	LMNA-NTRK1	Russo 2016
NSCLC	CD74-NTRK1	Vaishnavi 2013
	MPRIP-NTRK1	Vaishnavi 2013
	SQSTM1-NTRK1	Farago 2015
	TRIM24-NTRK2	Stransky 2014
Mammary Analogue of Secretory Carcinoma (MASC) of the salivary gland	ETV6-NTRK3	Skálová 2010
Squamous cell head and neck cancer	PAN3-NTRK2	Stransky 2014
Spitzoid melanoma	LMNA-NTRK1	Wiesner 2014
	TP53-NTRK1	Wiesner 2014
GIST	ETV6-NTRK3	Brenca 2016
Intrahepatic Cholangiocarcinoma	RABGAP1L-NTRK1	Ross 2014
Soft tissue sarcoma	LMNA-NTRK1	Doebele 2015
	TPM3-NTRK3	Stransky 2014
	NTRK3-HOMER2	Doebele 2015
Inflammatory Myofibroblastic Tumor (IMT)	ETV6-NTRK3	Yamamoto 2016
Soft tissue fibrosarcoma (pediatric)	LMNA-NTRK1	Doebele 2015
	SQSTM1-NTRK1	Doebele 2015
Soft tissue schwannoma (pediatric)	TPM3-NTRK1	Doebele 2015
Soft tissue hemangioma (pediatric)	ETV6-NTRK3	Doebele 2015
Soft tissue solitary fibrous tumor (pediatric)	TFG-NTRK3	Doebele 2015
Infantile (congenital) fibrosarcoma (pediatric < 1 year)	ETV6-NTRK3	Knezevich 1998
Glioblastoma Multiforme	NFASC-NTRK1	Frattini 2013
	BCAN-NTRK1	Kim 2014
	CHTOP-NTRK1	Zheng 2014
	ARHGEF2-NTRK1	Zheng 2014

Table 3: List of NTRK Fusions in Solid Malignancies

Tumor type	*NTRK* fusion type	Reference
Low grade glioma	AFAP1-NTRK2	Stransky 2014
Pediatric non-brainstem High Grade Glioma	TPM3-NTRK1	Wu 2014
	BTBD1-NTRK1	Wu 2014
	VCL-NTRK2	Wu 2014
	AGBL4-NTRK2	Wu 2014
Papillary Thyroid	TPM3-NTRK1	Greco 2010
	TPR-NTRK1	Greco 2010
	TFG-NTRK1	Greco 2010
	PPL-NTRK1	Zheng 2014
Secretary breast carcinoma	ETV6-NTRK3	Tognon 2002

The use of tyrosine kinase inhibitors (TKIs) targeting TRK family kinases has led to clinical benefit in patients with solid malignancies harboring oncogenic *NTRK* fusions: *LMNA-NTRK1* in colorectal cancer and soft tissue sarcoma (Doebele 2015; Sartore-Bianchi 2016), *SQSTM-NTRK1* in NSCLC (Farago 2015), *ETV6-NTRK3* in mammary-associated secretory carcinoma (MASC) (Drilon 2016b). Additionally, larotrectinib showed clinical activity in 55 *TRK* fusion positive patients (both adults and pediatric) with a confirmed ORR of 76% and median DOR and PFS not yet reached (Drilon 2018). Unfortunately, patient samples obtained at time of progression showed acquired drug resistant mutations, including solvent front mutations. Therefore, identifying and treating patients with oncogenic *NTRK* fusion genes, and those that acquire resistant mutations is important going forward. Further, in 54 adult patients with solid tumors with an *NTRK* gene fusion, entrectinib demonstrated clinical activity with an ORR of 57% and the percent of patients with a DOR of ≥ 6 months, 9 months and 12 months of 68%, 61% and 45%, respectively (ROZLYTREK™ USPI, 2019).

1.2 Disease Progression on TKIs in Oncogenic RTK Driven Solid Malignancies

Despite the established benefit of crizotinib and second-generation ALK inhibitors, median PFS in treatment-naïve advanced *ALK*+ NSCLC patients treated with front-line crizotinib was approximately 10.9 months (Lu 2016; Solomon 2014). Unfortunately, resistance to various ALK inhibitors invariably develops, and the median PFS of second-generation ALK inhibitors crizotinib-refractory *ALK*+ NSCLC patients ranged from 7.4 months for ceritinib (Shaw 2013b), 8.9 months for alectinib (Ou 2016) to 12.9 months for brigatinib (Kim 2016a).Generally, the mode of resistance can be broadly divided into 3 categories. First is the development of brain metastases especially among patients on crizotinib. Second is the development of acquired ALK resistant

mutations. Third is the activation of multiple bypass pathways. All three of which can occur in combination.

1.2.1 Development of Acquired Resistant Mutations Including Solvent Front Mutations

ALK, ROS1 and TRK kinases prefer type I kinase inhibitor for favored interactions at the hinge, glycine-rich P-loop, and the solvent front exposure area. As shown in Table 4, all of the inhibitors have a red colored heteroatom interacting with the hinge. Most of the *N*-lobe mutations of ALK, including 1151Tins, C1156Y, I1171T/N/S, F1174L/C, V1180L and L1196M destabilize the inactive conformation and shift ALK to a more active conformation for tighter binding with ATP. The mutations at ALK G1202, D1203, S1206 and E1210 residues, referred together as solvent front mutations, are caused by steric interactions with the blue colored motif, a bulky feature of all ALK inhibitors, and have been identified to be acquired resistance mutations to ALK inhibitors. It is not surprising that ALK G1202R mutation renders a common resistance to all ALK inhibitors because of an aromatic ring (blue colored) just located over the G1202 residue. Although it is much smaller (MW 406.42) than most of ALK inhibitors, the third generation of ALK inhibitor lorlatinib still has a pyrazole aromatic ring (blue colored) located on the top of G1202 residue, leading to a much reduced activity against ALK G1202R mutation (>20 fold reduction over wild type) (Gainor 2016). Similar to ALK G1202R, the same glycine mutations of ROS1 G2032R (Awad 2013), TRKA G595R (Russo 2016), and TRKC G623R (Drilon 2016b) render resistances to ROS1 inhibitor crizotinib and TRK inhibitor entrectinib, respectively in the clinic.

Table 4: ALK/ROS1/TRK Inhibitors and Clinical Resistant Mutations

Kinase Inhibitor	Structure	Molecule Weight (MW)	Primary Targets	Clinical Resistant Mutations
Crizotinib	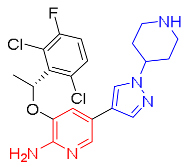	450.34	ALK, ROS1, MET	ALK[a]: 1151Tins, C1156Y, I1171T/N/S, L1196M, G1202R*, S1206Y/C, E1210K, G1269A<br><br>ROS1: G2032R*[b], D2033N*[c]
Ceritinib	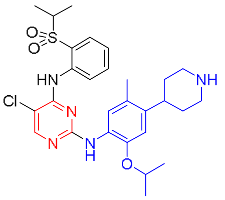	558.14	ALK, ROS1	ALK[a]: C1156Y, I1171T/N/S, F1174L/C, V1180L, L1196M, G1202R*, G1202del, D1203N<br><br>ROS1: no data reported
Alectinib	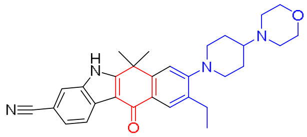	482.64	ALK	ALK[a]: I1171T/N/S, V1180L, L1196M, G1202R*

Table 4: ALK/ROS1/TRK Inhibitors and Clinical Resistant Mutations

Kinase Inhibitor	Structure	Molecule Weight (MW)	Primary Targets	Clinical Resistant Mutations
Brigatinib	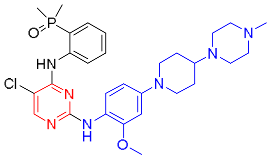	584.10	ALK, ROS1	ALK[a]: G1202R*, D1203N, S1206Y/C, E1210K<br><br>ROS1: no data reported
Lorlatinib	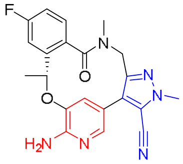	406.42	ALK, ROS1	ALK: L1198F[d]<br><br>ROS1: no data reported
Entrectinib	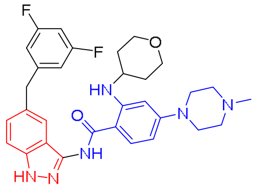	560.65	ALK, ROS1, pan-TRK	ALK: no data reported.<br><br>ROS1: no data reported.<br><br>TRKA: G595R*[e]<br>TRKC: G623R*[f]
Larotrectinib	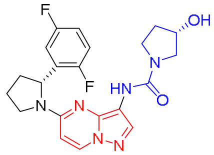	428.44	Pan-TRK	TRKA: G595R[g]<br>TRKC: G623R[g]

* solvent front mutation

a Gainor 2016

b Awad 2013

c Drilon 2016a

d Shaw 2016b

e Russo 2016

f Drilon 2016b

g Drilon 2018

Overall, the current ALK, ROS1, and TRK inhibitors approved or in clinical trials are much bigger than the adenosine triphosphate (ATP) non-phosphate motif, and are predicted to develop common and unique resistance mutations in the clinic. An inhibitor targeting the resistance-driven kinase active conformation and located completely inside ATP adenine binding pocket to avoid developing steric hindrance from resistance mutations is highly desired for systematically overcoming the acquired resistance mutation issues.

1.2.2 Activation of Bypass Signaling Pathways (SRC, FAK, JAK/STAT)

The tumor microenvironment is being increasingly recognized as a key factor in multiple stages of disease progression, particularly local resistance, immune-escaping, and distant metastasis. Excessive activation of the JAK family and their associated transcription factors STAT3 and STAT5 have been observed in many human malignancies in response to increased abundance of inflammatory cytokines in the tumor microenvironment prominently produced by infiltrating innate immune cells (Buchert 2016). JAK/STAT3 signaling promotes cancer hallmarks in the tumor and its microenvironment, including proliferation, survival, angiogenesis, and tumor metabolism while suppressing anti-tumor immunity (Buchert 2016). It was reported that JAK/STAT3 signaling pathway was aberrantly increased in epidermal growth factor receptor (EGFR) inhibitor-resistant EGFR-mutant NSCLC cells, and JAK2 inhibition overcomes acquired resistance to EGFR inhibitors (Gao 2016). Focal amplification of JAK2 gene was also observed in post chemotherapy triple-negative breast cancers in a group of 9p24-amplified tumors, suggesting a role in tumorigenicity and chemoresistance (Balko 2016). Therefore, pharmacologic inhibition of the JAK2 signaling pathway can be an important new therapeutic strategy to enhance anti-tumor activity and overcome treatment resistance.

Also, SRC kinase has been identified to contribute broadly to cancer treatment resistance including radiotherapy, chemotherapy, and targeted therapy (Zhang 2012). SRC/FAK signaling pathway plays important roles in regulating anti-tumor immunity (Serrels 2015), cancer stem-like properties (Thakur 2015), and epithelial-mesenchymal transition (EMT) (Wilson 2014). SRC family kinases can promote mitogenic signaling from growth factor receptors in a number of ways, including initiation of signaling pathways required for DNA synthesis, control of receptor turnover, actin cytoskeleton rearrangements and motility, and survival (Bromann 2004). Recent findings have demonstrated that integrins participate in the regulation of cancer stem-cell biology and are required for cancer progression, metastasis, and drug resistance *via* SRC/FAK signaling (Seguin 2015). The integrin β1/SRC/AKT-driven bypass signaling is a key mediator of acquired resistance to EGFR-targeted anticancer drugs (Kanda 2013). The EGFR-SRC family kinase-STAT3 signaling pathway was upregulated in BRAF inhibitor-resistant cells and tumors with intrinsic or acquired resistance to vemurafenib (Girotti 2013). BRAF and EGFR or SFK inhibition blocked proliferation and invasion of these resistant tumors. It was found that the increased SRC activation conferred considerable trastuzumab resistance in breast cancer cells and correlated with trastuzumab resistance in patients (Zhang 2011). Similarly, SRC activation was observed in lapatinib-resistant breast cancer cell lines. The selective small-molecule SRC inhibitor saracatinib combined with lapatinib synergistically inhibited the proliferation, migration, and invasion of lapatinib-resistant cells (Formisano 2014). In addition, it was found that the selective SRC inhibitor saracatinib can restore sensitivity to ALK inhibitor in 6 of 9 patient-derived resistant ALK fusion positive NSCLC cell lines (Crystal 2014), suggesting a therapeutic role of SRC in modulating ALK inhibitor resistance. SRC proteins were overexpressed in alectinib-resistance H3122 cells and the combination of alectinib and saracatinib demonstrated significant treatment effect in in vitro and *in vivo* studies using tumor-bearing mouse model (Yoshida 2016). Overall, SRC activation plays an important role in the development of targeted therapy resistance, and

targeting SRC signaling pathway may represent a valid strategy overcoming bypass signaling and EMT.

1.2.3 Disease Progression in the Central Nervous System (CNS)

There is a higher incidence of central nervous system (CNS) metastases (27%) in advanced treatment-naïve *ALK*+ NSCLC patients (Lu 2016; Solomon 2014) as compared to the incidence of CNS metastasis (13%-15%) in advanced treatment-naïve epidermal growth factor receptor (*EGFR*+) NSCLC patients (Park 2016; Rosell 2012; Schuler 2016). Furthermore, isolated CNS progression accounted for approximately 40% to 50% as the site of progression for *ALK*+ NSCLC patients (Khozin 2015; Ou 2014). Indeed, the incidence of CNS metastases increased with disease progression with successive treatment (Kim 2016b; Ou 2016; Shaw 2013a; Shaw 2014a; Shaw 2016a).

While crizotinib has demonstrated intracranial activity of approximately 18% in *ALK*+ NSCLC patients with untreated brain metastasis in a large-scale retrospective analysis of *ALK*+ NSCLC patients treated with crizotinib (Costa 2015), the second-generation ALK inhibitors such as alectinib, ceritinib, and brigatinib have demonstrated much higher intracranial activity (Kim 2016b; Kim 2016a; Ou 2016; Shaw 2013a; Shaw 2014a, Shaw 2016a). Additionally, while crizotinib may improve progression-free survival in *ALK*+ NSCLC patients with treated CNS metastases when compared to platinum/pemetrexed chemotherapy, it did not prolong intracranial time to tumor progression when compared to platinum/pemetrexed in *ALK*+ NSCLC patients with or without treated brain metastases (Solomon 2016).

The incidence and pattern of CNS progression in *ROS1*+ NSCLC patients and in patients with solid malignancies harboring *NTRK* rearrangement is not well studied and documented given the lower incidence of these patients. However, it is more likely than not that the pattern of CNS progression will be similar to *ALK*+ NSCLC patients. Thus, development of ROS1 and TRK inhibitors should also have activity against metastases in the CNS.

A key event of brain metastasis is the migration of cancer cells through the blood-brain barrier (BBB). It was reported that SRC was hyperactivated in brain-seeking breast cancer cells derived from human cell lines or from patients' brain metastases (Zhang 2013). Activating SRC signaling is important for promoting tumor cell extravasation into the brain parenchyma *via* permeabilization of BBB. FAK is enriched in brain blood vessels compared to surrounding cell types, phosphorylated in response to vascular endothelial growth factor (VEGF), and forms signaling complexes with SRC and integrins in VEGF-induced signaling (Lee 2010). It was found that the brain vascular permeability was proportionally increased with brain tumor burden, and FAK was a mediator of tumor-induced vascular permeability in the brain (Lee 2010). The conditional endothelial-specific deletion of FAK in the tumor-bearing mice resulted in reduced vascular permeability, partial restoration of cell-cell junction proteins in the tumor vessels and astrocyte-endothelial interactions in tumors, leading to reduced tumor growth in brain (Lee 2010). Therefore, targeting SRC/FAK signaling will be a good strategy to restore BBB structure/function in cancer to overcome/prevent CNS metastasis.

2 REPOTRECTINIB

Repotrectinib is a potent small-molecule inhibitor of ALK, ROS1, and TRK family with a rigid three-dimensional macrocyclic structure and much smaller size than current ALK, ROS1 and TRK family inhibitors. Repotrectinib is completely located inside adenine binding boundary without a blue colored solvent exposure motif as illustrated in Table 4, leading to efficiently binding with the active kinase conformation and circumventing the steric interference from clinical resistant mutations, especially the gatekeeper and solvent front mutations of ALK, ROS1 and TRK family kinases. At the clinically relevant concentrations, repotrectinib also inhibits JAK2, SRC and FAK which are important targets in modulating multiple resistance mechanisms, including bypass signaling, EMT, cancer stemness, and metastasis. Therefore, the unique compact macrocyclic structure and polypharmacology profile of repotrectinib will potentially provide opportunities to overcome multiple resistance mechanisms, including a broad spectrum of acquired mutations, bypass signaling, EMT, cancer stemness, and metastasis, for the treatment of subjects with advanced solid tumors harboring *ALK*, *ROS1*, or *NTRK1-3* rearrangements.

2.1 In vitro and *In vivo* Inhibitory Activity of Repotrectinib

2.1.1 Activity Against Wild-type (WT) and Mutated ALK

Designed to equally inhibit wild-type (WT) and most mutated ALKs, repotrectinib was found to inhibit the recombinant kinase activities of ALK (the half maximal inhibitory concentration IC_{50} 1.04 nM) and its mutants, including ALK G1202R, L1196M, F1174L, F1174S, C1156Y, S1206R, L1152R, R1275Q, 1151T Ins, T1151M, and G1269A with IC_{50} values of 1.21, 1.08, 1.46, 1.02, 0.932, 0.525, 1.23, 2.79, 2.16, 0.491, and 5.50 nM, respectively. In Karpas299 cell-based assays, repotrectinib suppressed phosphorylation of NPM-ALK (IC_{50} < 3 nM) and its downstream signaling effectors STAT3, AKT and ERK, leading to anti-proliferation at an IC_{50} of 23.7 nM. Repotrectinib suppressed ALK phosphorylation with IC_{50} values around 30 nM in the engineered NIH3T3 and Ba/F3 cells expressing EML4-ALK v1 or EML4-ALK v1 G1202R. Furthermore, repotrectinib inhibited cell proliferation of engineered Ba/F3 cells expressing EML4-ALK v1 (IC_{50} 21.1 nM) or its clinical mutant, eg, EML4-ALK v1 G1202R, L1196M, F1174C, C1156Y and L1152P with IC_{50} values of 20.5, 74, 54.2, 24, and 60.1 nM, respectively.

Consistently, repotrectinib demonstrated marked tumor growth inhibition (94%) in the *in vivo* Karpas299 tumor model with a trough free plasma concentration of 19 nM, corresponding to ~90% inhibition of ALK phosphorylation, achieved at a BID (twice a day) dose of 50 mg/kg. Marked tumor growth inhibition was achieved by repotrectinib in NIH3T3 EML4-ALK v1 WT (141%) tumor model and Ba/F3 tumor models expressing EML4-ALK v1 WT (154%) or G1202R (99%) with a BID dose of 75 mg/kg without overt toxicity, corresponding to a free plasma concentration at the end of dosage interval ($C_{trough)}$ of 13.3 nM. Overall, repotrectinib demonstrated effective inhibition against both WT and clinically mutated ALKs, especially the solvent front mutation ALK G1202R in in vitro and *in vivo* ALK assays and tumor models. A free plasma C_{trough} of 13.3 nM is able to achieve tumor regression and complete tumor growth inhibition in Ba/F3 EML4-ALK v1 WT and G1202R tumor models, respectively.

2.1.2 Activity Against WT and Mutated ROS1

Repotrectinib was found to potently inhibit the kinase activities of ROS1 (IC_{50} 0.0706 nM) and its solvent front mutation G2032R (IC_{50} 0.46 nM) in biochemical assay. Repotrectinib effectively suppressed the phosphorylation of CD74-ROS1, and its mutants G2032R, L2026M, D2033N with estimated IC_{50} values of <1, 3, 10, and 1 nM, respectively, in engineered NIH3T3 cell-based assays. Meanwhile, repotrectinib inhibited cell proliferation of engineered Ba/F3 cells expressing SDC4-ROS1, SDC4-ROS1 G2032R, CD74-ROS1, CD74-ROS1 G2032R, CD74-ROS1 L2026M, or CD74-ROS1 D2033N with IC_{50} values of <0.2, 3, <0.2, 8.4, 10, and 0.15 nM, respectively.

In *in vivo* tumor models, a BID dose of 15 mg/kg of repotrectinib resulted in a free trough plasma concentration of 12.7 nM, leading to a complete tumor regression in Ba/F3 CD74-ROS1 xenograft tumor model, and 97% inhibition of CD74-ROS1 G2032R phosphorylation and 99% inhibition of tumor growth in Ba/F3 CD74-ROS1 G2032R xenograft tumor model. A BID dose of 75 mg/kg resulted in a complete tumor regression in Ba/F3 CD74-ROS1 G2032R tumor model without causing animal toxicity. Overall, repotrectinib is a potent inhibitor against ROS1 fusions and the corresponding clinical resistance mutations, especially the solvent front G2032R mutation. A free plasma C_{trough} of 12.7 nM is able to achieve complete tumor regression and tumor growth inhibition in Ba/F3 CD74-ROS1 WT and G2032R tumor models, respectively.

2.1.3 Activity Against Wild-type and Mutated TRK

Repotrectinib had a similar binding affinity with TRKA, TRKB, and TRKC with K_d values of 0.019, 0.054 and 0.088 nM, respectively. Repotrectinib potently inhibited the enzymatic kinase activities of TRKA (IC_{50} 0.826 nM), TRKB (IC_{50} 0.0517 nM) and TRKC (IC_{50} 0.0956 nM).

Correspondently, repotrectinib suppressed the phosphorylation of fusion TRKs and their solvent front mutants with IC_{50} values in a range of 0.01 to 3 nM in KM12 and engineered NIH3T3 cells. Repotrectinib inhibited cell proliferation of KM12 cells (IC_{50} 0.2 nM) and engineered Ba/F3 stable cells expressing fusion TRKs or solvent front mutants, eg, LMNA-TRKA G595R (IC_{50} 0.4 nM), TEL-TRKB (also called EVT6-TRKB) G639R (IC_{50} 0.6 nM) and TEL-TRKC (ETV6-TRKC) G623R (IC_{50} 0.39 nM).

Meanwhile, repotrectinib demonstrated a dose-dependent inhibition of tumor growth in the KM12 tumor model, with a free trough plasma concentration of 6.9 nM corresponding to >90% tumor growth inhibition at a BID dose of 3 mg/kg. Additionally, a BID dose of 15 mg/kg of repotrectinib produced a free trough plasma concentration of 22.7 nM, leading to 128% and 97% tumor growth inhibition in NIH3T3 LMNA-TRKA WT and G595R mutant xenograft tumor models, respectively. A BID dose of 60 mg/kg of repotrectinib led to tumor regression in NIH3T3 LMNA-TRKA G595R tumor model without causing animal toxicity. Overall, repotrectinib is a potent pan-TRK inhibitor and markedly inhibits solvent front mutations in in vitro cellular assays and *in vivo* tumor models. A free plasma C_{trough} of 22.7 nM is able to achieve tumor regression and 97% tumor growth inhibition in NIH3T3 LMNA-TRKA WT and G595R tumor models, respectively.

2.1.4 Activity Against Solvent Front Mutations of ALK, ROS1 and TRKA/B/C

Repotrectinib is designed to inhibit both WT and mutant kinases, especially solvent front mutations. The activities of repotrectinib against WT and solvent front mutated ALK, ROS1 and TRKs are summarized in Table 5 and Figure 1. Repotrectinib demonstrated potent inhibition in in vitro cellular assays and in *in vivo* tumor models against the solvent front mutations of ALK, ROS1 and TRKs without a significant potency shifts when compared to the WT kinases.

Table 5: The Activities of Repotrectinib against WT and Solvent Front Mutated ALK, ROS1 and TRKs

Target	Auto-phosphorylation IC_{50} (nM)	Anti-proliferation IC_{50} (nM)	TGI%* (Tumor model)	Free C_{trough} (nM)
	NIH3T3 Cells	Ba/F3 Cells		
EML4-ALK v1 WT	30	21.1	154% (Ba/F3, SCID mice)	13
EML4-ALKv1 G1202R	30	20.5	99% (Ba/F3, SCID mice)	13
SDC4-ROS1 WT	ND	<0.2	139% (NIH3T3, nude mice)	23
SDC4-ROS1 G2032R	ND	3	ND	ND
CD74-ROS1 WT	<1	<0.2	200% (Ba/F3, SCID mice)	13
CD74-ROS1 G2032R	3	8.4	200% (Ba/F3, SCID mice)	13
CD74-ROS1 D2033N	1	0.15	ND	ND
LMNA-TRKA WT	<0.01	<0.2	128% (NIH3T3, nude mice)	23
LMNA-TRKA G595R	0.1	0.4	97% (NIH3T3, nude mice)	23
TEL-TRKB WT	0.1	<0.2	ND	ND
TEL-TRKB G639R	3	0.6	ND	ND
TEL-TRKC WT	ND	<0.2	ND	ND
TEL-TRKC G623R	ND	0.39	ND	ND

Abbreviations: ND = not determined. SCID = severe combined immune deficiency
* TGI% >100% means tumor regression and 200% means complete tumor regression.

Figure 1: The Anti-tumor Efficacy of Repotrectinib in WT and Solvent Front Mutated ALK, ROS1 and TRKA Tumor Models

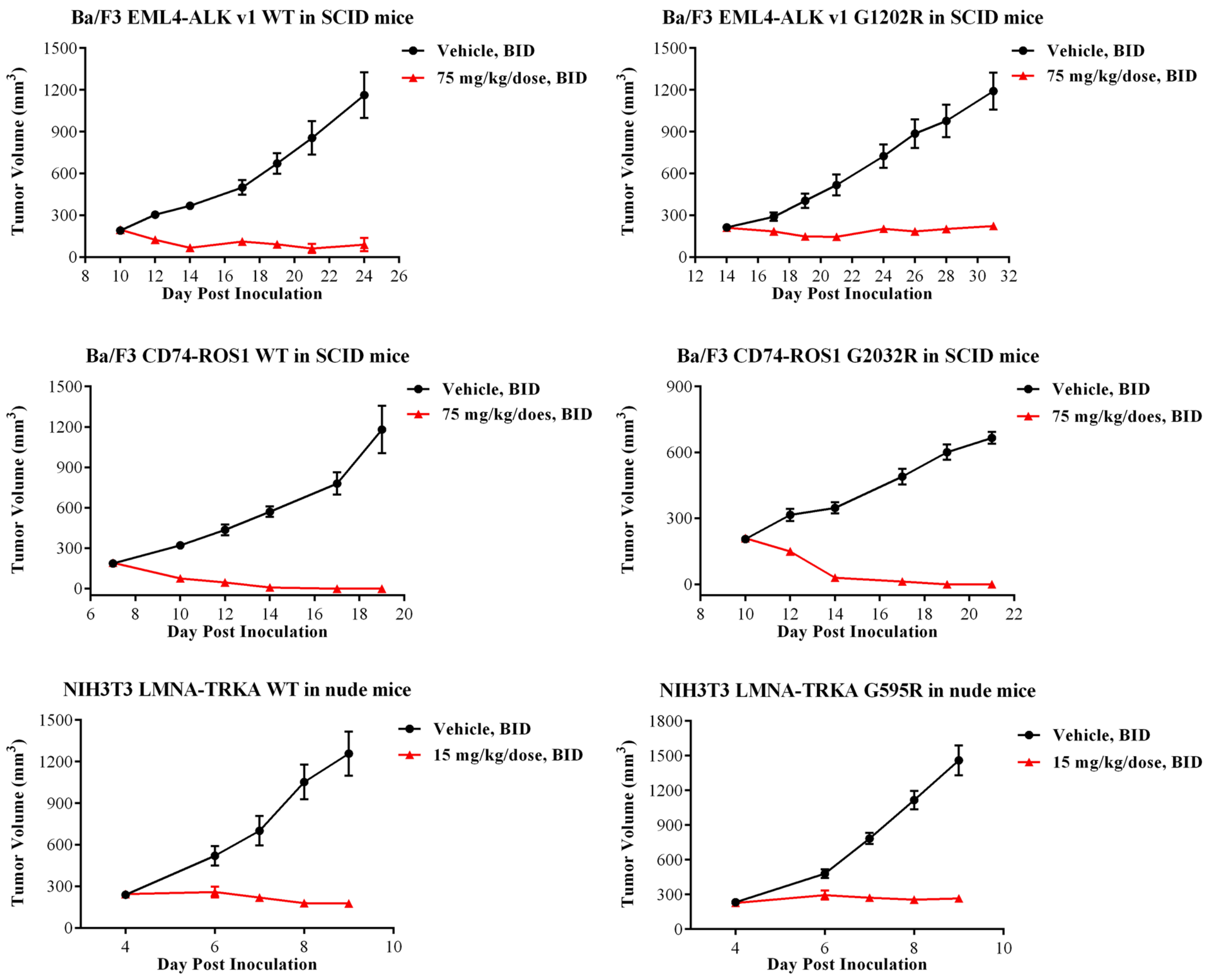

2.1.5 *Activity Against SRC, FAK and JAK2 for Modulation of Bypass Signaling, EMT, Cancer Stemness and Metastasis*

Kinase selectivity of repotrectinib over 456 human kinases (WT and mutants) was evaluated in kinase binding assay using the KINOMEscan® profiling platform, and the hits were further determined for IC_{50} values in enzymatic kinase inhibition assays.

It was found that repotrectinib also inhibited JAK2, SRC and FAK with IC_{50} values of 1.04, 5.29 and 6.96 nM, respectively in enzymatic kinase inhibition assays. Repotrectinib inhibited JAK2 signaling (pSTAT5 IC_{50} 139 nM) and cell proliferation (IC_{50} 169 nM) of SET2 cells harboring oncogenic JAK2 V617F mutation.

H2228 lung cancer cell line endogenously expresses EML4-ALK v3 and was found to be resistant to ALK inhibitor TAE684 (Koivunen 2008). Upregulation of EGFR (Isozaki 2016), mesenchymal marker vimentin (Voena 2016), and cancer stem-like marker CD44 (Jokinen 2014) was reported

in H2228 cells, likely leading to intrinsic resistance to ALK inhibitors. In the anti-proliferation assays, both crizotinib and ceritinib were found to be resistant in H2228 cells with IC_{50} values around 1000 nM, consistent with the literature reports. However, repotrectinib was able to overcome the intrinsic resistance with an IC_{50} of 100 nM. Additionally, repotrectinib suppressed the phosphorylation of SRC, FAK and paxillin with IC_{50} values in a range of 80 nM -100 nM, and downregulated the expression of EGFR, CD44, and vimentin with IC_{50} values around 100 nM in H2228 cells.

In addition, repotrectinib inhibited the phosphorylation of the oncogenic transcription/translation factor YB-1 with an IC_{50} value around 100 nM in H2228 cells. YB-1 is involved in many aspects of gene expression control that lead to tumor cell growth and drug resistance (Lasham 2013), including modulation of EGFR upregulation (Stratford 2007), EMT (Castellana 2015), and cancer stemness (Kang 2013). Therefore, it was postulated that inhibition of SRC/FAK by repotrectinib suppressed the phosphorylation of YB-1, leading to the downregulation of EGFR, CD44 and vimentin, and eventually to anti-proliferation effect on H2228 cells. repotrectinib demonstrated in vitro anti-metastatic activity by inhibiting cell migration in both H2228 cells and HT1080 human fibrosarcoma cells.

Overall, repotrectinib demonstrated inhibition of JAK2, SRC and FAK in cellular assays, and has the potential in overcoming resistance based on the mechanisms of bypass signaling, EMT, cancer stemness, and metastasis.

2.2 Repotrectinib Pre-Clinical Safety Data

Repotrectinib has been evaluated in a comprehensive nonclinical toxicology program consistent with the guidance provided in the ICH S9 Guideline and the Questions and Answers, ICH S9 Guideline. The rat and monkey were selected as the appropriate species for toxicity studies based on similar metabolic profiles to human and based on the ability to achieve adequate systemic exposures based on tolerability. Furthermore, the protein sequences of ROS1, ALK and NTRK1-3 are well conserved across rat, monkey, and human. Please refer to TPX-0005 version 13 Investigator's Brochure for detailed information.

Following repeat-dose oral administration of repotrectinib daily for 7, 28, or 91 days in Sprague Dawley rats and cynomolgus monkeys, the main toxicities observed were skin ulceration and CNS effects in rats, bone marrow effects (ie, hypocellularity, increased megakaryocytes) in rats and monkeys and GI tract effects (ie, emesis, watery feces, and minimal subacute/chronic inflammation and/or minimal to mild mucosal gland hyperplasia in the large intestines) in monkeys. The skin findings in rats were considered secondary to TRK inhibition resulting in loss of sensation and bodily harm. These findings were mostly reversible during recovery. There were no repotrectinib-related cardiovascular (including QT effects) or respiratory effects. CNS symptoms including tremors and ataxia were observed in the rats in repeat-dose toxicology studies. While no skin abnormalities were noted in the patients, peripheral nervous system (PNS), CNS, bone marrow, and GI tract adverse events (AEs) were noted at the intended drug exposure levels in clinical trials with repotrectinib.

Repotrectinib was not mutagenic nor clastogenic, but it was considered aneugenic in both in vitro and in vivo micronucleus assays. In a preliminary embryo-fetal development toxicity study in pregnant rats, fetal external malformation of malrotated hindlimbs was observed at estimated exposures approximately 3-fold higher than the clinical exposures at 160 mg BID based on AUC. Similar to adult rats, juvenile rats had CNS and bone marrow toxicity findings; however, CNS effects in juvenile rats at PND 13-14 were much more severe than what was observed in adult rats at similar exposures and resulted in acute mortality and early euthanasia in the dose-range finding study. Decreased effects on growth (decreased body weight, food consumption, and femur length) were observed in the definitive study, but no effects on age of attainment of balanopreputial separation or vaginal patency nor any effects on auditory startle responsiveness, motor activity, and learning and memory were observed. An in vivo phototoxicity study in rats showed no evidence of cutaneous or ocular phototoxicity, suggesting repotrectinib does not have phototoxicity potential.

2.3 Benefit to Risk Ratio Assessment for First-in-Human Study

The nonclinical safety profile of repotrectinib has been extensively evaluated in the rats and monkeys to support progression into clinical trials in advanced cancer indications. The primary target organ effects were observed on the rat skin, hematopoietic, and monkey thymus. The reversibility of the target organ toxicities was established following a 28-day treatment-free recovery period. Repotrectinib is also a potential phototoxicant based on Ultra Violet –B (UVB)-absorbing properties. However, based on results from a multiple-dose phototoxicity study conducted in pigmented rats, no direct phototoxicity is anticipated in subjects undergoing repotrectinib treatment. The nonclinical safety findings related to repotrectinib administration can be monitored and are considered clinically manageable or acceptable risks in the intended subject population.

The primary pharmacodynamics data demonstrated that repotrectinib potently inhibited the WT and mutated ALK, ROS1 and TRKs. The cellular anti-phosphorylation IC_{50} values and the anti-proliferation IC_{50} values ranged from < 1 nM for WT and solvent front mutated fusion TRKs and WT fusion ROS1s, < 10 nM for solvent muted fusion ROS1s, to < 30 nM for WT and solvent front mutated EML4-ALKs. A free plasma $C_{trough} \leq 23$ nM can lead to either complete tumor regression or > 90% tumor growth inhibition in tumors driven by fusion ALK, ROS1, TRKs, or their solvent front mutants. The anti-tumor efficacy of repotrectinib was dose-dependent and demonstrated strong correlations to inhibition of ALK, ROS1 and TRK phosphorylation, and there was no body weight loss observed in any of the *in vivo* efficacy studies. Collectively, the data indicate that repotrectinib is a potent inhibitor of WT and mutated ALK, ROS1, and TRK family kinase. The small and rigid three-dimensional macrocyclic structure allows repotrectinib target active kinase conformation and systematically overcome a broad spectrum of clinical resistance mutations in an structurally expected manner, especially the solvent front mutations ALK G1202R, ROS1 G2032R, TRKA G595R, and TRKC G623R. Additionally, the potent kinase inhibitory activities against JAK2/SRC/FAK provide a unique polypharmacology profile to repotrectinib for combating multiple resistance mechanisms simultaneously. Therefore, the benefit to risk

assessment is in favor of studying repotrectinib in subjects with advanced solid tumors harboring *ALK*, *ROS1* or *NTRK1-3* rearrangements.

2.4 Safety Consideration in the Clinical Trial

As repotrectinib absorbs light in the 290-700 nm range (λ_{max} = 290 nm) and the Molar Extinction Coefficient (MEC) value calculated for this absorption maximum (7100 L $mol^{-1}cm^{-1}$) exceeds the 1000 L $mol^{-1}cm^{-1}$ value stipulated in the International Council for Harmonisation (ICH) S10 Tripartite Guideline (ICH Tripartite Guideline, 2013). A further phototoxicity study was conducted in pigmented rats with multiple doses. The results from the *in vivo* multi-dose phototoxicity study indicated that no direct phototoxicity is anticipated from repotrectinib treatment. Therefore, subjects undergoing repotrectinib therapy do not need to take extensive measures to avoid sun exposure.

2.5 Repotrectinib Pharmacokinetics in Animals and Projection of Human Pharmacokinetics

In single-dose intravenous (IV) studies, mean plasma elimination half-life (t½) was approximately 2.4 hours in fasted male Institute of Cancer Research (ICR) mice, 11.9 hours in fasted male Sprague Dawley (SD) rats, 7.1 hours in fasted female SD rats, 9.7 hours in fasted male beagle dogs and 10.7 hours in fasted female cynomolgus monkeys. The total plasma clearance was low to moderate, ranging from 10% (female monkey) to 41% (male rat) of hepatic blood flow and the apparent volume of distribution (Vd) was greater than total body water in all species tested. In single-dose PO studies, the oral bioavailability of repotrectinib ranged from 13.5% (monkey) to 61% (female rat) in fasted mice, rats, dogs, and monkeys. A gender difference was observed in rats with female rats having higher exposure and oral availability.

In oral repeat-dose studies of rats and monkeys for up to 28 days, the systemic exposures (AUC_{0-24h}) to repotrectinib generally increased in a less than dose-proportional manner at dose levels above 20 mg/kg. Approximate 2-fold AUC_{0-24h} accumulation was observed following 28-day repeated dose for male rats at 30 mg/kg and female rats at 6 and 20 mg/kg, while <2-fold at 10 and 30 mg/kg and no accumulation at 100 mg/kg for monkeys, respectively. The clinical capsule formulation led to 40% increases in C_{max} and AUC values compared to the suspension formulation used in toxicity studies at comparable doses in monkey crossover PK studies.

After single and 7-day repeated oral dosing of repotrectinib to female CD-1 mice, brain tissue-to-plasma ratios were approximately 5.2% and 3.8%, respectively.

Repotrectinib was highly bound to plasma protein ranging from 92.07% (dog), 92.65% (monkey), 94.92% (rat), 95.41% (human) to 95.77% (mouse). Following incubation of repotrectinib in whole human blood, the human blood-to-plasma concentration ratio of repotrectinib was 0.56, suggesting some compartmentalization of the compound in blood cells.

Repotrectinib was mainly metabolized by CYP3A4, and did not inhibit CYP1A2, CYP2B6, CYP2D6 or CYP3A4 (midazolam) with IC_{50} values >50 µM. Repotrectinib weakly inhibited CYP2C8, CYP2C9, CYP2C19 and CYP3A4 (testosterone) at high concentrations (IC_{50} values ≥5.72 µM), and exhibited minimal potential for time-dependent inhibition of CYP isozymes.

Treatment of cultured human hepatocytes with up to 75 µM repotrectinib had little or no effect on CYP1A2 mRNA expression; however, concentration-dependent increases in CYP2B6 and CYP3A4 mRNA were observed, suggesting that repotrectinib may induce CYP2B6 and CYP3A4 isoenzymes.

Repotrectinib inhibited uridine 5'-diphospho-glucuronosyltransferase 1A1 (UGT1A1) (IC_{50} = 1.60 µM), but not the other UGT isoforms tested. Therefore, caution may need to be exercised when concurrently administering repotrectinib with compounds that are metabolized by UGT1A1.

Repotrectinib was a substrate for P-glycoprotein (P-gp) and likely a substrate for breast cancer resistance protein (BCRP). Therefore, caution may need to be exercised when concurrently administering repotrectinib with known inhibitors of P-gp or BCRP. Repotrectinib inhibited P-gp- and BCRP-mediated transport of probe substrates at high concentrations (IC_{50} values of 16.7 and 6.9 µM, respectively). In the assessment of potential inhibition of human solute carrier transporters OATP1B1, OATP1B3, OAT1, OAT3, OCT2, MATE1 and MATE2-K, repotrectinib inhibited OATP1B1, OAT3, MATE1 and MATE2-K with IC_{50} values of 2.35, 15.2, 4.13 and 0.726 µM.

2.6 Rationale for Selection of the Starting Dose

The results of the 28-day definitive toxicity studies of rats and monkeys were used to calculate a reasonably safe clinical starting dose for repotrectinib in the proposed Phase 1/2 trial. The approach used is consistent with guidance provided in the ICH S9 guideline (ICH Tripartite Guideline 2013) and involves setting the starting dose at 1/10th of the severely toxic dose in 10% of animals (STD10) in rodents. If the nonrodent is the more appropriate species, then 1/6th of the HNSTD is considered an appropriate starting dose.

The doses tested in the 28-day toxicology study in the male/female rats were 30/6, 100/20, and 300/60 mg/kg once a day (QD) orally. The STD10 in male/female rats was determined to be 100/60 mg/kg/day respectively. For the purposes of calculating a reasonably safe clinical starting dose for repotrectinib, the more conservative 60 mg/kg/day female STD10 value was used. This value was converted to a body surface area mg/m^2/day value using the standard rat km value of 6 to arrive at a dose of 360 mg/m^2/day. Applying a safety factor of 10, the starting dose in humans would be 1/10th of the STD10 in rats or 1/10th of 360 mg/m^2/day = 36.0 mg/m^2/day. Since 1/10th of the STD10 in rat is below the HNSTD in the monkey (30 mg/kg or 360 mg/m^2), this dose is expected to be well tolerated by the monkey. Therefore, the rat STD10 was the primary reference species for the starting dose calculation. No supportive data are available at this stage that would suggest body surface area (BSA) dosing would reduce human inter-subject variability in pharmacokinetics (PK). Thus, based on a BSA of 1.62 m^2 (reference body weight of 60-kg subject), the recommended safe flat starting dose of repotrectinib is 58.3 mg/day. Since the clinical capsule formulation gave 40% increase on C_{max} and AUC in monkey crossover PK studies compared with the suspension formulation used for toxicity studies, the starting dose with the capsule formulation was calculated to 58.32/1.4=41.65 mg, round to 40 mg/day as the starting dose.

At the starting dose of 40 mg dose once daily, the projected human unbound exposure (AUC_{0-24h}, 87.2 h*ng/mL) and unbound C_{max} (5.0 ng/mL) is ~7.5- and 15-fold lower than the unbound exposure (AUC_{0-24h}, 655.7 h*ng/ml) and unbound C_{max} (76.1 ng/mL) observed at female rat NOAEL dose (20 mg/kg) on Day 28 and provides a good safety margin.

2.7 Preliminary Safety and Efficacy Data from the TPX-0005-01 Study

The preliminary safety and efficacy data from the TPX-0005-01 (TRIDENT-1) study is available in the Investigator's Brochure.

2.8 Rationale for the Recommended Phase 2 Dose Selection

The selection of the Recommended Phase 2 Dose (RP2D) was based on the observed safety, preliminary efficacy data obtained from Phase 1 based on the data cutoff of 04 March 2019, available pharmacokinetic data, as well as the results of preliminary population PK modeling and exploratory exposure response (ER) analyses.

Assessment of AEs among 83 subjects treated as of the data cutoff 4 March 2019 showed most AEs were grade 1 or grade 2 and managed without dose modifications. Dizziness, often reported during the first 1-2 weeks of repotrectinib treatment and considered TRK inhibition related, was limited to grade 1 and 2 in the majority of subjects and was manageable. There were only 3 (3.6%) subjects among total 83 treated reported with dose reductions or dose interruptions due to development of reported dizziness (1 subject at 240 mg QD [grade 3]) and 2 subjects at 160 mg BID (1 with grade 2 and 1 with grade 3). No DLTs were reported in a total of 23 subjects who received repotrectinib treatment at the 160 mg QD dose in Phase 1.

Clinical activity of repotrectinib has been demonstrated in subjects with *ROS1*+ or *TRK*+ solid tumors in Phase 1. A sustained pharmacodynamic coverage on the ROS1 G2032R SFM and other potential acquired mutations by 160 mg BID may further enhance clinical activity (eg, deeper response and/or prolonged duration of response).

Repotrectinib exhibited rapid absorption under fasted conditions as peak plasma concentrations (C_{max}) generally achieved at approximately 2-4 hours post-dose. PK exposure as measured by C_{max} and AUC generally increased in a dose-related manner. Mean estimates of terminal half-life ranged from 14.6 to 25.9 hours. After multiple once daily doses of repotrectinib values of C_{max} and AUC at steady-state were lower than those observed after a single dose at the same administered doses suggesting potential auto-induction of metabolic enzyme(s). A modest food effect on PK was observed; food appeared to prolong repotrectinib absorption and increased systemic exposure but may not be of clinically significance.

A preliminary population PK model was developed based on currently available PK data. Model-based PK simulations suggest that although repotrectinib exhibits time-dependent PK, steady-state PK can be attained following 7 days of once daily dosing, and approximately 25% increase in steady-state AUC is expected under fed condition, which is unlikely to be clinically significant. At 160 mg QD, the median steady-state C_{trough} is expected to exceed the IC_{90} for solvent front mutated ROS1 G2032R, and over 95% of the subjects are expected to have steady-state C_{trough} exceeding the IC_{90} for WT ROS1. For the dosing regimen of 160 mg QD for at least 7 days in

Cycle 1 followed by 160 mg BID, over 95% of the subjects are expected to have steady-state C_{trough} exceeding the IC_{90} for solvent front mutated ROS1 G2032R.

Taken together, based on the preliminary safety, clinical activity, available pharmacokinetic data, as well as the results of preliminary population PK modeling and exploratory exposure response (ER) analyses obtained from Phase 1 study, RP2D for Phase 2 study is selected as:

- 160 mg QD for the first 14 days, may increase to 160 mg BID. Repotrectinib can be taken with or without food.

(Subjects must meet all below criteria while on 160 mg QD prior to dose increasing to 160 mg BID: No grade ≥ 3 treatment-related AE, unmanageable grade ≥ 2 dizziness, ataxia or paresthesia; or grade ≥ 3 clinically significant lab abnormalities).

2.9 Preliminary Pharmacokinetic Data

Single- and multiple-dose PK of repotrectinib was evaluated in the dose escalation portion of the ongoing TRIDENT-1 study. Following oral administration of ascending single doses of repotrectinib ranging from 40 mg to 240 mg QD, repotrectinib exhibited rapid absorption with C_{max} occurring at approximately 2-4 hours post-dose under fasted conditions, and repotrectinib concentrations declined over time with mean terminal half-life values ranging from 14.6 to 25.9 hours. Systemic exposure, as measured by C_{max} and AUC, generally increased in a dose-related manner over the dose range evaluated (Table 6).

Table 6: Summary of Geometric Mean (%CV) Pharmacokinetic Parameters of Repotrectinib After a Single Oral Dose of Repotrectinib Under Fasted Conditions

Parameter	40 mg QD	80 mg QD	160 mg QD	240 mg QD	160 mg BID[c]	200 mg BID[c]
N (Total)	12	11	21	7	10	1
N (1a)<br>N (1b)	5<br>7	5<br>6	6<br>15	7<br>0	10<br>0	1<br>0
C_{max} (ng/mL)	279 (35)	347 (33)	683 (50)	1414 (45)	720 (46)	273
T_{max}[a] (h)	2 (1-4)	2 (1-8)	2 (1-6)	2 (1-4)	4 (1-8)	2
$t_{1/2}$ (h)	23.8 (42)	25.9 (20)	18.0 (37)[b]	14.6 (44)	16.4 (37)	31.2
AUC_{last} (ng•h/mL)	3915 (32)	5213 (53)	9090 (57)	21019 (85)	10464 (42)	4839
AUC_{inf} (ng•h/mL)	4498 (30)	5901 (55)	9922 (56)[b]	22095 (82)	11050 (43)	5999
CL/F (L/h)	8.9 (30)	13.6 (55)	16.1 (56)[b]	10.9 (82)	14.5 (43)	33.3

Table 6: Summary of Geometric Mean (%CV) Pharmacokinetic Parameters of Repotrectinib After a Single Oral Dose of Repotrectinib Under Fasted Conditions

Parameter	40 mg QD	80 mg QD	160 mg QD	240 mg QD	160 mg BID[c]	200 mg BID[c]
V_Z/F (L)	306 (51)	506 (51)	418 (71) b	229 (151)	343 (66)	1501

Abbreviations: BID = twice daily; QD = once daily.

a. Median (range)

b N=20

c Subjects in the 160 mg BID and 200 mg BID dose cohorts only received one repotrectinib dose during the PK lead-in to allow for single dose PK evaluation.

The multiple-dose PK of repotrectinib under fasted conditions was evaluated in subjects who participated in the Phase 1a portion of the ongoing TRIDENT-1 study. The multiple-dose PK parameters on Cycle 1 Day 15 following once daily or twice daily doses of repotrectinib are summarized in Table 7. Following once daily doses of repotrectinib, the C_{max} and AUC values generally increased with increasing doses. Accumulation (R_{acc}) of repotrectinib at steady-state (Cycle 1 Day 15) were lower than anticipated based on repotrectinib half-life and dosing regimen, and accumulation ratios were lower at higher doses (>80 mg QD), probably related to the net auto-induction, which becomes more pronounced with increasing repotrectinib doses.

Table 7: Summary of Geometric Mean (%CV) Pharmacokinetic Parameters of Repotrectinib After Single Ascending Oral Doses of Repotrectinib Under Fasted Conditions on Day 15 (Steady-State)

Parameter	40 mg QD	80 mg QD	160 mg QD	240 mg QD	160 mg BID	200 mg BID [c]
N	6	5	6	6	8	2
C_{max} (ng/mL)	351 (41)	437 (31)	482 (46)	725 (29)	560 (45)	411, 330
t_{max}[a] (h)	1 (1-2)	2 (1-2)	2 (1-4)	2 (2-6)	2 (1-8)	1, 2
AUC_{24} (ng•h/mL)	2372 (42)	2966 (29)	3966 (49)	6281 (20)	6663 (42)	5711, 3198
CL/F (L/h)	16.9 (42)	27.0 (32)	40.3 (49)	38.2 (20)	48.0 (42)	70.0, 125.1
R_{acc}	1.12 (47)[b]	0.99 (78)	0.49 (58)[b]	0.47 (72)	0.63 (43)	1.72[d]

Abbreviations: BID = twice daily; QD = once daily.

[a] Median (range)

[b] N=5

[c] N=2, Individual data listed

[d] N=1

The effect of food on repotrectinib PK was evaluated in the Phase 1b portion of the ongoing TRIDENT-1 study. Consumption of the standard high-fat, high-calorie meal resulted in increases of AUC at doses of 40 mg, 80 mg, and 160 mg QD, with geometric mean AUC ratios ranging from 1.04 to 1.31. Under fed condition, the time to maximum concentration (T_{max}) was delayed by approximately 2 hours. The geometric mean C_{max} ratios (fed/fasted) were 0.99, 0.86, and 1.4 for the doses of 40 mg, 80 mg, and 160 mg, respectively.

2.9.1 Study TPX-0005-09: Mass Balance, Pharmacokinetics, and Metabolism of Repotrectinib in Healthy Male Subjects

TPX-0005-09 was conducted as a 2-part, 2-treatment, fixed-sequence study in 7 healthy male volunteers to investigate the mass balance, PK, and metabolism of a single oral dose and PK of an intravenous tracer of [^{14}C]repotrectinib in healthy male subjects. The treatments were administered after an overnight fast of at least 10 hours. Below is a summary of key findings. Refer to the Investigator's Brochure for further details.

Following concomitant administration of a single oral dose of 160-mg repotrectinib capsule formulation and intravenous administration of tracer [^{14}C]repotrectinib in study TPX-0005-09 (Period 1), the geometric mean absolute bioavailability of repotrectinib based on $AUC_{0-\infty}$ was approximately 45.7%. The geometric means $t_{1/2}$ of the IV formulation and the oral capsule dose were 32.5 and 36.2 hours, respectively.

Following oral administration of a 160 mg of [^{14}C]-repotrectinib (100 µCi) dose (TPX-0005-09, Period 2), the mean total recovery of radioactivity in excreta was 93.7% with radioactivity recoveries in feces and urine of 88.8% and 4.84%, respectively. No metabolite exceeded 10% of total circulating drug-related radioactivity. All detected metabolites had been previously detected in rat and/or cynomolgus monkey following oral administration; therefore, there were no unique human metabolites.

3 STUDY DESIGN

3.1 Study Overview

This is a Phase 1/2, open-label, multi-center, multiple-dose, dose escalation, safety, PK, pharmacodynamic (PD), and anticancer efficacy exploration study of repotrectinib as a single agent in subjects with *ALK*+, *ROS1+, NTRK1+, NTRK2+,* or *NTRK3*+ advanced solid malignancies. This clinical study will consist of 2 parts, Phase 1 and Phase 2.

Throughout the Phase 1 Study, oversight will be provided by the Clinical Safety Committee (CSC) comprised of all Investigators and the Medical Monitor. The CSC will:

- Review all relevant safety findings and confirm all DLTs;

- Determine all cohort dose escalation and de-escalation decisions during the Dose Escalation Phase for Phase 1a, Phase1b, and Phase 1c;
- Determine recommended phase 2 dose (RP2D);

Within the Phase 2 Study, oversight will be provided by the Sponsor and the DMC.

3.2 Phase 1a and Phase 1c Dose Escalation

The Phase 1a portion of the study will estimate the maximum tolerated dose (MTD) for single agent repotrectinib in dose escalation cohorts in subjects with advanced *ALK+, ROS1+, NTRK1+, NTRK2+*, or *NTRK3+* solid malignancies.

To understand the single-dose PK of repotrectinib, a lead-in period preceding the continuous daily dosing was conducted in Cohorts 1-6. A single lead-in dose of repotrectinib was given on Day -3. No study drug was administered during the interval between the lead-in single dose and Day 1 of the first cycle.

For the twice daily (BID) regimen, the lead-in dose was the same dose under study given once a day (for example, the lead-in dose for the 80 mg BID dose level was 80 mg given 3 days before BID dosing begins), and the BID dosing started at Day 1 of the first cycle.

For Phase 1c, there will be no lead-in dose and repotrectinib will be given with food continuously from Cycle 1 Day 1.

Any changes to the dose or schedule and/or PK time points based on the emerging PK data will ONLY be implemented after a formal protocol amendment is submitted to the appropriate health authorities for review and has been approved by the institutional review boards (IRBs)/Ethics Committees (ECs).

3.2.1 Starting Dose Level for Dose Escalation Phases: Phase 1a and Phase 1c

The starting dose for repotrectinib in the first-in-human trial has been determined to be 40 mg once daily, based on information derived from the 28-day repeat-dose definitive toxicology studies in rats and monkeys (see Section 2.6).

The first cycle of treatment for the intense PK determination was initially 21 days for the Phase 1a Study (Cohorts 1-6). The lead-in period will no longer be required for Phase 1c and the duration of Cycle 1 in Cohorts 7 and beyond will be 28 days. Subsequent cycles will also be 28 days.

The initial dose levels of repotrectinib are shown in Table 8 and Table 9.

As of 04 March 2019, a total of 83 adult subjects have been enrolled in this ongoing Study of repotrectinib from both the Dose Escalation Phase (Phase 1a) the Food Effect Sub-Study (Phase 1b) and 11 subjects in Phase 1c. Doses and schedules tested include the following: 40 mg QD, 80 mg QD, 160 mg QD, 240 mg QD (Cohorts 1-4), 160 mg BID and 200 mg BID (Cohorts 5 & 6) in Phase 1a and 40 mg QD, 80 mg QD and 160 mg QD in Phase 1b, 120 mg QD, 160 mg QD and 160 mg QD for 7 days followed by 160 mg BID administered with food continuously. In each cohort, 6 evaluable subjects were enrolled. In some cohorts, replacement of subjects occurred due

to inadequate PK collection or to further understand steady-state exposure levels in Korean compared to US based subjects.

Doses up to 240 mg QD were well tolerated and 2 subsequent BID cohorts (160 mg BID and 200 mg BID) were then explored. Given that 3 DLTs occurred out of 12 enrolled subjects (25%) in the 160 mg BID cohort, it was conservatively determined that 160 mg BID exceeded the MTD.

Based on the findings from the Phase 1b study and the overall preliminary pharmacokinetic (PK) and clinical data, further dosing of repotrectinib continuously with food will be evaluated in the Phase 1c study (dosing cohorts outlined below) that includes up to 5 additional dosing cohorts. The escalation of each dose level will be based on the overall PK, and preliminary safety and efficacy data seen within each cohort. The cohorts in Phase 1c will follow a standard 3 + 3 design and dosing within a cohort will not continue if the dose is determined to have exceeded the MTD based on DLTs. The Phase 1c study will begin with dose level 7 (120 mg QD with food). During the DLT observation period (Cycle 1), if none of the first 3 subjects enrolled experiences a DLT, the dose of repotrectinib will be escalated by one dose level. If 1 subject in the first 3 subjects experiences a DLT, up to 3 additional subjects will be enrolled at that dose level. If there are no additional DLTs (ie, ≤1 of 6 subjects with a DLT), then dose escalation will proceed. Assuming Dose Levels 7 and 8 are cleared without exceeding the MTD, subjects will then be enrolled into Dose Level 9, which will start initially with a once daily (QD) dose of 160 mg for one week and, if no DLTs within the first seven days of QD dosing are observed, then subjects will have their doses escalated to the 160 mg twice daily (BID) schedule. If Dose Level 9 exceeds the MTD, patients will be simultaneously enrolled into Dose Levels 10 and 11.

Any changes to the dose or schedule based on emerging data will ONLY be implemented after a formal protocol amendment is submitted to the appropriate health authorities for review and has been approved by the institutional review boards (IRBs)/Ethics Committees (ECs).

3.2.2 Dose Escalation Levels

Table 8: Provisional Dose Levels (QD under fasted condition): Phase 1a and Phase 1b

Dose Level	Cohort	Proposed Dose Level (QD)	Increment from Previous Dose (%)
-1[a]		20 mg QD	(50% decrease)
1	1	40 mg QD	Starting Dose
2	2	80 mg QD	100%
3	3	160 mg QD	100%
4	4	240 mg QD	50%

Abbreviation: QD, once daily

[a] Dose level -1 represents a dose that may be evaluated if Dose Level 1 is poorly tolerated. No dose de-escalation below this level is planned for this study. If Dose Level -1 is poorly tolerated, then the study will be terminated.

Table 9: Provisional Dose Levels (BID under fasted condition): Phase 1a

Dose Level	Cohort	Proposed Dose Level (BID)	Increment from Previous Dose of 240 mg QD (%)
5	5	160 mg BID	33%
6	6	200 mg BID	25%

Table 10: Provisional Dose Levels (QD & BID with food): Phase 1c

Dose Level	Proposed Dose Levels and Schedules
-1	80 mg QD with food
7	120 mg QD with food
8	160 mg QD with food
9	160 mg QD with food for 7 days followed by 160 mg BID with food IF there are no DLTs within the first 7 days while on QD dosing
10*	120 mg QD with food for 7 days followed by 120 mg BID with food IF there are no DLTs within the first 7 days while on QD dosing
11*	120 mg QD with food for 7 days followed by 240 mg QD with food if there are no DLTs within the first 7 days

Abbreviations: BID, twice daily; DLT, dose-limiting toxicity; MTD, maximum tolerated dose; QD, once daily

* If Dose Level 9 exceeds the MTD, subjects will be enrolled into Dose Levels 10 and 11 simultaneously

Dose escalation will continue until the MTD is identified or PK saturation is reached if that occurs at a dose level less than the MTD (Section 3.2.3.3). Subjects will be dosed on a flat scale of mg/day and not by individual weight or BSA.

The planned dose escalations will not exceed doubling of the dose in principle. Initially, QD dosing schedule was utilized and yet the BID schedule based on emerging PK data was expected to decrease the C_{max} and increase the plasma trough concentrations and therefore was evaluated per Table 9 and will be further evaluated per Table 10.

3.2.3 Criteria for Dose Escalation and Determination of MTD

3.2.3.1 Maximum Tolerated Dose (MTD) Definition

For any given schedule, the MTD is the highest dose level of repotrectinib expected to cause a DLT in fewer than 33% of the treated subjects in the first repotrectinib treatment cycle or the dose level that has no more than 1 DLT in up to six treated subjects in the first cycle including the lead-in dose period (Phase 1a). For the Phase 1c portion of the study (Cohorts 7-10), given the lead-in dose at day -3 is no longer given, the duration of cycle 1 will be 28 days and therefore the DLT observation period will be 28 days.

The definition of MTD applies to both the QD and BID dosing schedules. Adverse events (AEs) and laboratory abnormalities considered to be DLTs are defined in Table 11.

3.2.3.2 Dose escalation and MTD Determination Process: (Phase 1a and Phase 1c)

The following provides a full description of the subject accrual process, dose escalation process, and determination of the MTD for the study:

1) To support subject safety at each dose level, a delay of at least 7 days will be mandatory between the administration of the first dose to the first subject and administration of the first dose to subsequent subjects (ie, at least a 7-day gap between the first and second subjects initiating study drug in all sites).
   a) Previously in Cohorts 1-6 of Phase 1a/b, up to 6 subjects were evaluated prior to dose escalation. Going forward and starting with Cohort 7, a standard 3 + 3 design will be followed with requirement of up to 3 additional subjects total if a DLT is reported in the first 3 subjects.
   b) Expansion of up to 6 subjects is also allowed if 0 of 3 subjects is reported with a DLT to further explore efficacy ONLY after a dose level is cleared, consistent with "backfilling" a cohort, ONLY if the cohort has shown preliminary efficacy so that subjects are not exposed to potentially subtherapeutic doses. For example, if no DLTs are reported in the first 3 subjects treated at the Cohort 7 dose level (120 mg QD with food), and dose escalation proceeds into Cohort 8 (160 mg QD with food), yet at least one response out of the 3 subjects treated within Cohort 7 was observed, the protocol will allow cohort expansion of up to 6 potential subjects within Cohort 7. This potential expansion is being explored based on the anti-tumor activity that has already been shown with doses above and below Dose Level 7 (120 mg QD).
   c) During the DLT observation period (Cycle 1), if none of the first 3 subjects enrolled are reported with a DLT, the dose of repotrectinib will be escalated by one dose level in each cohort. If 1 subject in the first 3 subjects is reported with a DLT, up to 3 additional subjects will be enrolled at that dose level. If there are no additional DLTs (ie, ≤1 of 6 subjects with a DLT), then dose escalation will proceed. If there are 2 subjects reported with DLTs among the first 3 subjects enrolled, or there is a second DLT in up to 6 subjects enrolled at any dose level, the MTD will have been exceeded and no additional subjects will be started at that dose. The MTD will then be established at the prior dose level. If the prior dose cohort only has 3 enrolled subjects, the cohort will be expanded to up to 6 total subjects. If there are ≤1 of 6 subjects reported with a DLT at that dose level, this dose will be considered the MTD of repotrectinib.
2) Provided there are not 2 DLTs that have already been reported at any given dose, subjects will be enrolled to a minimum of 3 and a maximum of 6 evaluable subjects per cohort. Dose escalation and dose reduction will follow the scheme below:
   a) If no DLT is reported (for definition see Section 3.2.3.3) in a cohort of 3 evaluable subjects, then dose escalation may occur. Dose increases will be permitted after review of data from a minimum of 3 evaluable subjects. Following the escalation from the current dose cohort to the next higher cohort, a total of up to six subjects may be enrolled in the previous cleared cohort to facilitate collection of more safety and PK data provided the benefit to risk ratio is positive ie, no concerning (for example, dose-limiting) safety findings and at least one

response has been seen at the previous dose so that subjects will not potentially be given subtherapeutic doses.

b) If one subject is reported with a DLT in a group of 3 evaluable subjects, then the cohort will be expanded to include up to 6 evaluable subjects. If only one DLT is reported in a cohort of up to 6 evaluable subjects, then dose escalation may occur.

c) If 2 or more subjects are reported with a DLT in a group of up to 6 subjects, irrespective of the number of subjects enrolled in that cohort, the dose will be considered to have exceeded the MTD and recruitment to that dose cohort and dose escalation will cease. A lower dose will be evaluated to better define the MTD.

3) The DLT observation period includes the preceding PK lead-in period and Cycle 1 in Phase 1a and Phase 1b. In the absence of the lead-in period in Phase 1c, the DLT period will be 28 days.

To fully characterize the safety or PK/PD of repotrectinib, subjects that are discontinued from treatment before completing the DLT observation period (Cycle 1) due to disease progression or other event unrelated to repotrectinib, or who did not complete the PK evaluations within Cycle 1, will be replaced.

Dose escalation may be terminated at any time based on emerging safety concerns without establishing the MTD.

All dose escalation within the Phase 1 portion of the study will stop if: (1) there is evidence in the dose-exposure relationship of saturation of absorption; (2) at least 6 subjects have been treated at a dose that is considered the recommended Phase 2 dose (RP2D) that will be used within the Phase 2 portion of the study; (3) all doses exceed the MTD.

Once the MTD of repotrectinib has been determined, the RP2D will be determined at or below the MTD. At least 6 subjects will be treated at the RP2D prior to proceeding into the Phase 2 portion of the study.

3.2.3.3 DLT Definition

Clinically relevant toxicities will be those assessed as unrelated to disease, disease progression, intercurrent disease, or concomitant medications. These will be evaluated according to National Cancer Institute Common Terminology Criteria for Adverse Events (CTCAE) v4.03.

DLT is defined as an adverse event or abnormal laboratory value assessed as unrelated to disease progression, intercurrent illness, or concomitant medications that meets any of the following criteria in Table 11.

For the purpose of dose escalation decisions, only DLTs reported within the first cycle (DLT observation period) will be considered. Within Cohorts 1-6, the DLT observation period was defined as starting on day -3 in Phase 1a or day -7 in Phase 1b and included the 21-day duration of Cycle 1. In the Phase 1c Study from Cohort 7 and beyond, the first lead-in dose will be at Cycle 1 Day 1 and the DLT observation period will be within Cycle 1 which is 28 days in duration.

Subjects are eligible for DLT evaluation if they are reported with a DLT after at least one dose of study drug, or are not reported with a DLT having taken a minimum of 75% of doses expected

during the first cycle of treatment. Subjects who do not fulfill these requirements and who discontinue study participation prior to completing the DLT assessment period will be replaced for DLT evaluation but will remain in the overall safety and efficacy analyses.

Table 11: Criteria for Defining Dose-limiting Toxicities

Toxicity	Any of the following criteria
**Toxicities resulting in an excessive number of missed doses**	Inability to deliver > 75% of the planned doses in Cycle 1 treatment because of toxicity related to repotrectinib
**Hematology**	CTCAE grade ≥ 4 neutropenia (ANC < 0.5×10^9/L)
	CTCAE grade ≥ 4 thrombocytopenia (platelets < 25×10^9/L)
	CTCAE grade ≥ 4 anemia not explained by underlying disease or condition
	CTCAE grade ≥ 3 febrile neutropenia (defined as ANC < 1000/mm^3 with a single temperature of ≥ 38.3°C [≥ 101°F] or a sustained temperature of ≥ 38°C [≥ 100.4°F] for > 1 hour)
**Renal**	CTCAE grade ≥ 3 creatinine increase (> 3 × ULN)
**Hepatic**	CTCAE grade ≥ 3 total bilirubin elevation (> 3 × ULN)
	CTCAE grade ≥ 2 total bilirubin elevation (> 1.5 × ULN) AND CTCAE grade ≥ 2 ALT or AST elevation (> 3 × ULN)
	CTCAE grade ≥ 3 ALT elevation (> 5 × ULN)
	CTCAE grade ≥ 3 AST elevation (> 5 × ULN)
**Pancreatic**	CTCAE grade ≥ 2 pancreatitis
	CTCAE grade ≥ 3 amylase or lipase elevation
**Cardiac**	CTCAE grade ≥ 3
**Other adverse events**	CTCAE grade ≥ 3 vomiting or nausea despite optimal anti-emetic therapy
	CTCAE grade ≥ 3 diarrhea despite optimal anti-diarrhea treatment
	Any CTCAE grade ≥ 3 AE, except for the exceptions noted below
	In view of the Investigators and the Sponsor any other unacceptable toxicity encountered
**Exceptions to DLT criteria**	CTCAE grade 3 or 4 elevations in alkaline phosphatase
	CTCAE grade 3 or 4 electrolytes abnormalities that are adequately managed by IV or PO supplementations
	CTCAE grade 3 fatigue < 7 days

Table 11: Criteria for Defining Dose-limiting Toxicities

Toxicity	Any of the following criteria
	CTCAE grade 2 dizziness that can be managed with dose interruptions and/or dose reductions

Abbreviations: ANC, absolute neutrophil count; CTCAE, Common Terminology Criteria for Adverse Events; DLT, dose-limiting toxicity; IV, intravenous; PO, per os; PRBC, packed red blood cells; ULN, upper limit of normal

CTCAE version 4.03 will be used for all grading.

Subjects may receive supportive care (eg, PRBCs) as per local institution guidelines

Optimal therapy for vomiting or diarrhea will be based on institutional guidelines, with consideration of the prohibited medications in this protocol.

3.2.3.4 Follow-up After a Dose-Limiting Toxicity

When treatment is interrupted due to a DLT, the subject must be followed at least once a week for 4 weeks, and subsequently at a maximum interval of 4 weeks, until resolution or stabilization of the event, whichever comes first.

All subjects will be followed for AEs and serious AEs (SAEs) for 28 days following the last dose of repotrectinib.

Hematologic Toxicities

If CTCAE grade ≥ 3 neutropenia, CTCAE grade ≥ 3 thrombocytopenia, or CTCAE grade ≥ 4 anemia (not due to underlying disease) have been demonstrated, these parameters must be repeated at least twice a week until resolution to CTCAE grade ≤ 2 neutropenia, CTCAE grade ≤ 2 thrombocytopenia, or CTCAE grade ≤ 2 anemia, and then at least weekly until either resolution to allow for re-treatment or until stabilization.

Renal Toxicity

If serum creatinine > 3 × upper limit of normal (ULN) has been demonstrated, this parameter must be repeated at least twice a week until resolution to CTCAE grade ≤ 1, and then at least weekly until either resolution to allow for initiation of re-treatment or until stabilization.

Hepatic Toxicity

If total bilirubin >3 × ULN or CTCAE grade ≥ 3 aspartate aminotransferase (AST) (also called serum glutamic oxaloacetic transaminase [SGOT])/ Alanine aminotransferase (ALT) (also called serum glutamic pyruvic transaminase [SGPT])/alkaline phosphatase (ALP) has been demonstrated, these parameters must be repeated at least twice a week until resolution to CTCAE grade ≤ 1 (or grade ≤ 2 if liver metastasis present), and then at least weekly until either resolution to allow for initiation of re-treatment or until stabilization.

Subjects with total bilirubin > ULN (any duration) should have fractionation of bilirubin into total/direct or indirect/direct components and any additional work-up as clinically indicated by these results. Follow-up of hyperbilirubinemia should proceed as per the guidelines above, irrespective of the results of fractionation.

Non-laboratory Toxicity

Subjects with a non-laboratory DLT not described in the sections above must be evaluated at least weekly following presentation of the DLT until resolution to allow for retreatment, or stabilization of the toxicity.

3.2.3.5 Dose Modifications: Intra-subject dose escalation/reductions, and re-escalation criteria (for Phase 1 only)

For subjects who experience a DLT with repotrectinib, dose adjustments are permitted if it is considered in the best interest of the subject to continue therapy after discussing with the Medical Monitor and the Sponsor.

Subjects who cannot be re-treated with repotrectinib should have weekly follow-ups that include a physical examination, vital signs including weight, ECOG PS, ECGs, and assessment of AEs and concomitant medication. Following a DLT or toxicity, hematology, renal, and liver function tests should be performed as appropriate.

If a subject requires a dose delay of > 28 days from the intended day of the next scheduled dose of repotrectinib, then the subject must be discontinued from the study. Subjects, who discontinue from the study for a study drug-related AE or an abnormal laboratory value must be followed as described in Section 9.4.

For each subject, a maximum of 2 dose reductions will be allowed, after which the subject should be discontinued from the study. Once a subject has undergone a dose level reduction, the dose level may not be re-escalated during subsequent treatment cycles with repotrectinib. Dose reduction for repotrectinib means treatment at a lower repotrectinib dose level. Planned dose levels for repotrectinib are listed in Table 8, Table 9, and Table 10. All interruptions or changes to study drug administration must be recorded on the study case report form (CRF).

The dose levels in Table 8, Table 9, and Table 10 will be followed in all dose reduction decisions. For example, subjects currently being treated at 120 mg QD should dose reduce one dose level to 80 mg QD. If a subject is being treated at 160 mg QD and requires dose reduction, they should dose reduce to 120 mg QD initially and then 80 mg QD if needed.

Subjects currently on the BID schedule may have a dose reduction to a QD schedule provided the total daily dose is lower than their current total daily dose, ie, 80 mg BID may dose reduce to 40 mg BID or 120 mg QD, whereas subjects on 120 mg BID may dose reduce first to either 160 mg QD or 80 mg BID and then subsequently to 120 mg QD (Table 12).

Table 12: Recommended Dose Modifications for Phase 1

Current Dose level	Dose Level -1	Dose Level -2
120 mg QD	80 mg QD	40 mg QD
160 mg QD	120 mg QD	80 mg QD

Table 12: Recommended Dose Modifications for Phase 1

Current Dose level	Dose Level -1	Dose Level -2
240 mg QD	160 mg QD	80 mg QD
120 mg BID	160 mg QD OR 80 mg BID	120 mg QD
160 mg BID	160 mg QD OR 120 mg BID	120 mg QD

Abbreviations: BID, twice daily; QD, once daily

For dose modifications in Phase 2, please refer to Section 6.2.

Intra-subject dose escalation is permitted on study yet ONLY after a subject has completed the third cycle of repotrectinib unless there is documented disease progression by RECIST Version 1.1 after the DLT observation window has passed for that subject. After the third cycle is completed, individual subjects may be considered for treatment at a dose of repotrectinib higher than the dose that is initially assigned. In order for a subject to be eligible for intra-subject dose escalation and treated at a higher dose of repotrectinib, he or she must have received the lower dose for at least 3 cycles of therapy without a drug-related toxicity of CTCAE grade ≥ 2 or have documented disease progression after completing the DLT observation period (Cycle 1) without a DLT. Observation of CTCAE grade ≤ 3 laboratory abnormalities not requiring dose modifications (as identified in Table 11) would be permitted. Moreover, the new, higher dose with which the subject is to be treated must be a dose that has completed evaluation where at least 3 subjects have completed Cycle 1 with repotrectinib without having a DLT and that does not exceed the MTD or the RP2D. Any further increases after the initial intra-subject dose escalation are subject to the same rules as for the initial intra-subject escalation. Consultation with the Sponsor's Medical Monitor must occur prior to any intra-subject dose escalation decision.

3.2.3.6 Recommended Phase 2 Dose (RP2D) Definition

The RP2D is the dose chosen for further study in Phase 2 based on the Phase 1 results. The RP2D will be the MTD unless one or more of the following suggest an alternate dose below the MTD would be preferable: (a) clinically significant anti-tumor effect (complete response, partial response or prolonged stable disease) occurs below the MTD, in which case a clinically active dose level may be selected as the RP2D; (b) the MTD is not achieved, in which case the highest dose level administered may become the RP2D; or (c) toxicities observed beyond the DLT observation period (Cycle 1) require selecting the RP2D below the MTD level.

Once the MTD of repotrectinib has been determined, if fewer than 6 subjects have been treated at the dose identified as the RP2D, enrollment will continue until at least 6 subjects are treated at the RP2D, which may be at or below the MTD.

The rationale for the Recommended Phase 2 Dose Selection is described in Section 2.8.

Based on the preliminary clinical pharmacology, safety, and efficacy obtained from Phase 1 study, RP2D for Phase 2 study is selected as:

- 160 mg QD for the first 14 days, may increase to 160 mg BID. Repotrectinib can be taken with or without food.
- (Subjects must meet all below criteria while on 160 mg QD prior to dose increasing to 160 mg BID: No grade ≥3 treatment-related AE, unmanageable grade ≥2 dizziness, ataxia or paresthesia; or grade ≥3 clinically significant lab abnormalities).

3.3 Repotrectinib Phase 1b Food Effect Subgroup

The effect of food on the pharmacokinetics of repotrectinib was evaluated in a food effect sub-study up through the 160 mg QD dose level. Preliminary findings are outlined in Section 2.9.

The testing order for fed versus fasted conditions was as follows: the first half of the subjects to participate in this sub-study were tested under fed followed by fasted conditions, the next half of the subjects were tested under fasted followed by fed conditions. Subjects who had a gastrectomy or have dietary or other restrictions that preclude a 10-hour overnight fast (water permitted) or consumption of the required high-fat, high-calorie meal did not participate in this sub-study.

The effect of a high-fat, high-calorie breakfast on repotrectinib pharmacokinetics was studied. The food effect sub-study commenced at the 40 mg QD dose level where preliminary anti-tumor activity (partial response) was observed by the Investigator. The food effect sub-study then continued at subsequent dose escalation/de-escalation levels.

Each subject served as his/her own control. In half of the subjects per dose level, repotrectinib was administered in the morning under either “fed” followed by “fasted” conditions on Cycle 0 Day -7 and on Cycle 1 Day 1, respectively. The other half of the subjects per dose level repotrectinib was administered in the morning under either “fasted” followed by “fed” conditions on Cycle 0 Day -7 and on Cycle 1 Day 1, respectively. No trial drug was administered during the interval after Cycle 0 Day -7 and before Cycle 1 Day 1. The subjects skipped dosing on Cycle 1 Day 2 and Day 3. The third dose was administered at Cycle 1 Day 4. After Cycle 1 Day 4 all subjects continued to receive repotrectinib under fasted conditions.

A total of 6 subjects were enrolled at each dose level. Additionally, a total of 12 subjects were to be enrolled at the presumed RP2D. Based on the finding of grade 3 dizziness in a subject treated at the 240 mg QD dose level and knowing the effect of food increased the C_{max}, it was decided that further dose escalation should cease, and that the 160 mg QD should be expanded to 12 total subjects to evaluate the food effect sub-study.

3.4 Midazolam Drug-Drug Interaction Sub-study

After the RP2D of repotrectinib is identified, the midazolam interaction sub-study will be evaluated at the RP2D level. To evaluate the potential effect of repotrectinib on CYP3A induction, a midazolam (MDZ) DDI sub-study will be conducted in 6 PK evaluable subjects after RP2D determination. All subjects participating in this sub-study will be administered a single 5 mg oral dose of MDZ alone on Day -2 under fasted conditions (no food 8 hours before through 2 hours after dosing of MDZ), and serial plasma samples will be collected for up to 24 hours to assess midazolam PK. Starting on Cycle 1 Day 1, subjects will begin daily treatment of 160 mg

repotrectinib QD followed by 160 mg BID on Cycle 1 Day 15. On Cycle 1 Day 22, another single dose of 5 mg MDZ will be administered under fasted conditions (no food 8 hours before through 2 hours after dosing of MDZ) concurrently with repotrectinib (morning dose). Serial plasma samples will again be collected for up to 24 hours to assess the induction potential of repotrectinib using midazolam as a probe substrate for CYP3A. A comparison of midazolam's PK profiles on Day -2 and Cycle 1 Day 22 will then allow an assessment of the potential of repotrectinib to induce CYP3A. Repotrectinib full PK will be assessed using serial plasma samples collected on Cycle 1 Day 1 and Day 22, and abbreviated PK samples will be collected on Cycle 1 Day 15 and Day 1 of Cycles 2, 3 and 4 (see APPENDIX 7). Once the DDI assessment periods end, subjects will continue the study treatment until progression of disease as determined by the Investigator, unacceptable toxicity, or consent withdrawal. Repotrectinib can be taken without regard to food between Cycle 1 Day 1 to Cycle 1 Day 21, as well as after completion of the DDI assessment.

3.5 Phase 2 Study

The Phase 2 portion of the study will evaluate the anticancer activity of single-agent repotrectinib at the identified RP2D as listed below:

- 160 mg QD for the first 14 days, may increase to 160 mg BID. Repotrectinib can be taken with or without food.
- (Subjects must meet all below criteria while on 160 mg QD prior to dose increasing to 160 mg BID: No grade ≥3 treatment-related AE, unmanageable grade ≥2 dizziness, ataxia or paresthesia; or grade ≥3 clinically significant lab abnormalities).

Multiple sub-populations of subjects with *ROS1+*, *NTRK1+*, *NTRK2+*, or *NTRK3+* advanced solid malignancies will be enrolled. The Phase 2 segment of the study is designed to further define the safety and PK profiles of single-agent repotrectinib at the RP2D. All subjects in the Phase 2 portion of this trial will receive repotrectinib regardless of food intake orally either once daily or twice daily depending on the RP2D and schedule for 28 consecutive days in repeated 4-week cycles.

The total enrollment of Phase 2 will be approximately 630 subjects. The subjects will be accrued into 6 expansion cohorts according to tumor of origin, prior treatment, and *ROS1* or *NTRK* rearrangement (see Figure 2).

3.5.1 ROS1+ Solid Malignancy Expansion Cohorts

The *ROS1+* expansion cohorts will enroll either ROS1 TKI-naïve or ROS1 TKI pretreated subjects.

3.5.1.1 ROS1+ NSCLC Expansion Cohorts

There are 4 *ROS1+* NSCLC expansion cohorts.

- **EXP-1: ROS1 TKI-naïve ROS*1*+ NSCLC (n=110).**
  - No prior exposure to a ROS1 TKI is allowed.

  - Up to one prior line of chemotherapy or immunotherapy is allowed (chemo- or immunotherapy-based combination regimen is considered as one line of treatment).
- **EXP-2: 1 Prior ROS1 TKI AND 1 Platinum-based Chemotherapy *ROS1*+ NSCLC (n=120).**
  - Disease progression or intolerant to one prior line of a ROS1 TKI.
  - ROS1 TKIs used in a prior line of treatment are limited to crizotinib, ceritinib, entrectinib, or lorlatinib. Note: Any previous exposure to a ROS1 TKI is considered as one prior line of TKI treatment (eg, if the same ROS1 TKI was given before and after a chemotherapy or other systemic therapy, it is considered as 2 prior TKIs and the subject would not be eligible for EXP-2).
  - In addition, the subject must have received one prior line of platinum-based chemotherapy OR one prior line of platinum-based chemotherapy in combination with immunotherapy before or after a ROS1 TKI (Note: subject is not eligible if he/she has been treated with more than one line of chemotherapy OR has received immunotherapy alone).
- **EXP-3: 2 Prior ROS1 TKIs and NO Chemotherapy or Immunotherapy *ROS1*+ NSCLC (n=80).**
  - Disease progression or intolerant to 2 prior lines of a ROS1 TKI treatment.
  - ROS1 TKIs used in prior lines of treatment are limited to crizotinib, ceritinib, entrectinib, lorlatinib, brigatinib, ensartinib, or cabozantinib. Other prior ROS1TKI agents that are not listed may be allowed after discussion with the Sponsor Medical Monitor. Note: Any previous exposure to a ROS1 TKI is considered as one prior line of TKI treatment (eg, if 2 different ROS1 TKIs are utilized, or the same ROS1 TKI was given before and after a chemotherapy or other systemic therapy, it is considered as 2 prior TKIs and the subject would be eligible).
  - No prior lines of chemotherapy or immunotherapy are allowed.
- **EXP-4: 1 Prior ROS1 TKI and NO Chemotherapy or Immunotherapy *ROS1*+ NSCLC (n=120).**
  - Disease progression or intolerant to one prior line of a ROS1 TKI.
  - ROS1 TKIs used in a prior line of treatment are limited to crizotinib, ceritinib, entrectinib, or lorlatinib. Note: Any previous exposure to a ROS1 TKI is considered as one prior line of TKI treatment (eg, if the same ROS1 TKI was given before and after a chemotherapy or other systemic therapy, it is considered as 2 prior TKIs and the subject would not be eligible for EXP-4).
  - Note: No prior lines of chemotherapy or immunotherapy are allowed.

3.5.2 NTRK+ Solid Malignancy Expansion Cohorts

For country-specific requirements, please refer to APPENDIX 8.

NTRK+ expansion cohorts will enroll TRKi-naïve (EXP-5) and TRKi-pretreated (EXP-6) subjects respectively. For the TRKi-naïve *NTRK*+ expansion cohort (EXP-5), there is no limit to the number of prior regimens of cytotoxic chemotherapy or immunotherapy.

For the TRKi-pretreated *NTRK*+ expansion cohort (EXP-6), there is no limit to the number of prior regimens of cytotoxic chemotherapy or immunotherapy AND no more than 2 prior TRK inhibitors are allowed:

- **EXP-5 (TRK TKI-native, n=approximately 80)**: *NTRK*+ advanced solid tumors.
  - No prior exposure to TRK TKI is allowed. Prior lines of chemotherapy or immunotherapy allowed.
  - As of global protocol amendment Version 15.0 (or equivalent version for respective countries), subjects with NSCLC are not allowed.
- **EXP-6 (TRK TKI-pretreated, n=approximately 120)**: *NTRK*+ advanced solid tumors.
  - Disease progression or intolerant to 1 or 2 prior TRK TKIs.
  - Prior lines of chemotherapy or immunotherapy allowed.
  - TRK TKI used in prior lines of treatment are limited to: entrectinib, larotrectinib, selitrectinib (LOXO-195), and cabozantinib. Other prior TRK TKIs that are not listed may be allowed after discussion with the Sponsor. Note: Any previous exposure of a TRK TKI is considered as one prior line of TKI treatment, eg, if 2 different TRK TKIs are utilized or the same TRK TKI was used before and after a chemo- or other systemic therapy, it is considered as 2 prior TKIs and the subject would be eligible.
  - As of global protocol amendment Version 15.0 (or equivalent version for respective countries), subjects with NSCLC are not allowed.

Given the rarity of *NTRK*+ solid malignancies of a particular organ type, the statistical calculation and enrollment goal of the TRKi-naïve and TRKi-pretreated expansion cohorts will be the same respectively independent of organ of origin.

If repotrectinib shows promising clinical efficacy in one particular *NTRK*+ tumor organ type, there will be potential to expand that particular *NTRK*+ organ type ONLY after a formal protocol amendment has been submitted to the appropriate health authorities for review and no change will be implemented until approval by the individual IRBs has been received.

Figure 2: Schema of TPX-0005-01 Study

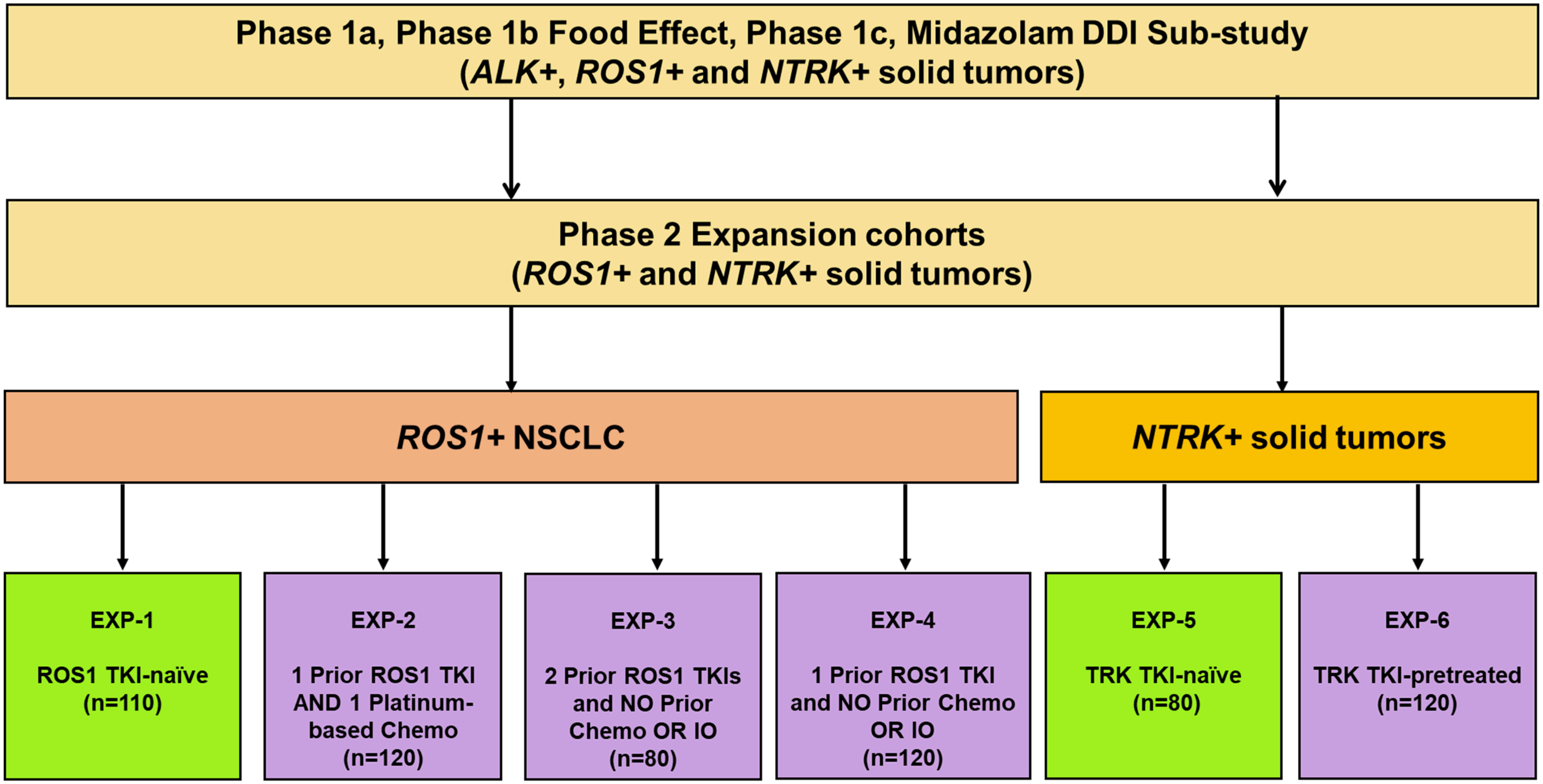

Approved v2.0 930210036 2.0

3.6 End of Study Definition

The end of study will be defined as the time at which the secondary endpoint of OS has been met in each expansion cohort. The DMC may recommend termination of the study prior to meeting the secondary endpoint (see Section 12). The Sponsor may end study at any time upon Sponsor discretion.

4 STUDY OBJECTIVES

4.1 Phase 1 Objectives

4.1.1 Phase 1 Primary Objectives

- To determine the first cycle DLTs of repotrectinib given to adult subjects with advanced solid malignancies harboring an *ALK*, *ROS1*, *NTRK1*, *NTRK2*, or *NTRK3* gene rearrangement.
- To determine the MTD of repotrectinib in adult subjects with advanced solid malignancies harboring an *ALK*, *ROS1*, *NTRK1*, *NTRK2*, or *NTRK3* gene rearrangement.
- To determine the biologically effective dose and RP2D of repotrectinib for adult subjects with advanced solid malignancies harboring an *ALK*, *ROS1*, *NTRK1*, *NTRK2*, or *NTRK3* gene rearrangement.

4.1.2 Phase 1 Secondary Objectives

- To evaluate the safety and tolerability of repotrectinib at various doses in subjects with advanced solid tumors that harbor an *ALK, ROS1*, *NTRK1*, *NTRK2*, or *NTRK3* gene rearrangement.
- To evaluate the single- and multiple-dose pharmacokinetic (PK) profiles of repotrectinib.
- To determine the effect of food on the PK of repotrectinib.
- To determine the preliminary objective response rate (ORR) by Blinded Independent Central Review (BICR) and clinical benefit rate (CBR) of repotrectinib, in subjects with advanced solid tumors that harbor an *ALK, ROS1*, *NTRK1*, *NTRK2*, or *NTRK3* gene rearrangement.
- To evaluate the potential of repotrectinib to induce CYP3A using midazolam as a probe substrate.

4.1.3 Phase 1 Exploratory Objectives

- To evaluate the pharmacodynamic (PD) effects of repotrectinib in subjects with advanced solid tumors that harbor an *ALK, ROS1, NTRK1, NTRK2, or NTRK3* gene fusion.
- To explore the potential prognostic utility of genomic alterations and characterize intrinsic or acquired resistance to repotrectinib using liquid biopsy and tissue samples.

4.2 Phase 2 Objectives

4.2.1 Phase 2 Primary Objectives

- To determine the confirmed ORR as assessed by BICR of repotrectinib in each subject population expansion cohort of solid tumors that harbor a *ROS1, NTRK1*, *NTRK2*, or *NTRK3* gene rearrangement.

4.2.2 Phase 2 Secondary Objectives

- To determine the DOR, time to response (TTR), and clinical benefit rate (CBR) of repotrectinib, as assessed by BICR, in each subject population expansion cohort of advanced solid tumors that harbor a *ROS1, NTRK1*, *NTRK2*, or *NTRK3* gene rearrangement.
- To estimate the PFS and overall survival (OS) of subjects treated with repotrectinib with advanced solid tumors that harbor a *ROS1, NTRK1*, *NTRK2*, or *NTRK3* gene rearrangement.
- To evaluate the safety and tolerability of repotrectinib when administered at the RP2D in subjects with advanced solid tumors that harbor a *ROS1, NTRK1*, *NTRK2*, or *NTRK3* gene rearrangement.
- To determine the intracranial objective response rate (IC-ORR) of repotrectinib and CNS -PFS in subjects presenting with measurable brain metastases at baseline, using modified RECIST Version 1.1 assessment.
- To confirm PK of repotrectinib at the RP2D.
- To assess treatment-related symptoms and general health status using validated instruments of subject-reported outcomes (EORTC-QLQ-C30 and LC-13 when applicable) in subjects treated with repotrectinib.

4.2.3 Phase 2 Exploratory Objectives

- To explore the potential prognostic utility of genomic alterations and characterize intrinsic or acquired resistance to repotrectinib using liquid biopsy and tissue samples.
- To explore association between ORR by subgroups including demographic and baseline risk factors in each expansion cohort.

5 SUBJECT SELECTION

For country-specific requirements to inclusion criteria, please refer to APPENDIX 8.

5.1 Inclusion Criteria for Phase 1

1) Histologically or cytologically confirmed diagnosis of locally advanced or metastatic solid tumor (including primary CNS tumors; Stage IV, American Joint Committee on Cancer Version 7) that harbors an *ALK, ROS1, NTRK1, NTRK2,* or *NTRK3* gene rearrangement as by:
   a) All diagnostic tests must be conducted in CLIA lab or equivalent.
   b) Any nucleic acid-based diagnostic testing method (eg, next-generation sequencing [NGS], Sanger sequencing, reverse transcription-polymerase chain reaction) performed at a local clinical laboratory improvement amendments-certified (CLIA-certified) or equivalently accredited diagnostic laboratory. In NGS test results, the fusion gene partner for *ALK,*

ROS1, NTRK1, NTRK2, or *NTRK3* gene rearrangement has to be identified and reported. All tests must have been performed according to the product's instructions for use (IFU).

c) Break-apart fluorescence in situ hybridization (FISH) is allowed for diagnosis of *ALK*, *ROS1*, *NTRK1*, *NTRK2*, and *NTRK3* rearrangements. For *ALK* rearrangement detection in NSCLC, the FISH test has to be performed using the FDA-approved Abbott Molecular's Vysis® ALK Break-apart FISH Probe Kit. All tests must have been performed according to the product's IFU.

d) Immunohistochemistry (IHC) detection of *ROS1*, *NTRK1*, *NTRK2*, and *NTRK3* rearrangement will not directly qualify subjects. Archival tumor samples of these IHC positive subjects (ROS1, TRKA, TRKB, TRKC) will have to be sent to the Sponsor's designated central laboratory for confirmation of *ROS1*, *NTRK1*, *NTRK2*, and *NTRK3* rearrangement prior to enrollment onto the trial. IHC detection of *ALK* rearrangement in NSCLC is allowed if performed using the Ventana ALK (D5F3) companion diagnostic assay. All tests must have been performed according to the product's IFU.

e) For subjects enrolled per the diagnostic tests outlined above and approved by the Sponsor, central laboratory confirmation of the specific gene alteration is not required before start of repotrectinib treatment as long as adequate archival tissue is sent to the Sponsor's designated central laboratory.

2) All subjects must have archival tissue sample or fresh tissue sample available and/or collected prior to enrollment. Formalin-fixed paraffin-embedded (FFPE) tissue block(s) from initial diagnosis that contain sufficient tissue to generate at least 10 (preferably 15), 5-micron thick unstained slides will be collected. If no FFPE block is available, then at least 10 (preferably 15) unbaked, 5-micron thick unstained slides with a minimum of 10% (preferably 20%) tumor content and 1 Hematoxylin and eosin stain (H&E) slide must be provided. Specimens will be sent to the the Sponsor-designated central laboratories for *ALK*, *ROS1*, or *NTRK* rearrangement status confirmation.

3) ECOG PS 0–1.

4) Age ≥18 (or age ≥ 20 of age as required by local regulation).

5) Willing and able to provide written institutional review board (IRB)/institutional ethics committee-approved Informed Consent.

6) At least 1 measurable target lesion according to RECIST version 1.1. CNS-only measurable disease as defined by RECIST version 1.1 is allowed.

7) Prior cytotoxic chemotherapy for advanced or metastatic disease is allowed. At the time of starting treatment with repotrectinib, at least 14 days or 5 half-lives (whichever is shorter) must have elapsed after discontinuation of prior cytotoxic chemotherapy (or at least 42 days for prior nitrosoureas, mitomycin C, and liposomal doxorubicin) and all side effects from prior treatments must have resolved to grade ≤ 1 (CTCAE Version 4.03) with the exception of alopecia.

8) Prior immunotherapy (eg, anti-PD-1, anti-PDL1, anti-TIM3, anti-OX40) is allowed (except for the treatment-naïve expansion cohort). At the time of starting treatment with repotrectinib, at least 14 days must have elapsed after discontinuation of prior immunotherapy treatment and all immune-related side effects from prior treatments must have resolved to grade ≤ 1.

9) Subjects with advanced solid tumors harboring *ALK*, *ROS1*, *NTRK1*, *NTRK2*, or *NTRK3* rearrangements are eligible. There is no limit to the number of prior chemotherapies, immunotherapy, or TKI regimens.
10) At least 7 days or 5 half-lives (whichever is shorter) must have elapsed since completion of treatment with the last ALKi, ROS1i, or TRKi prior to starting treatment with repotrectinib for subjects enrolling into the TKI-pretreated expansion cohorts. In the midazolam DDI sub-study, if a subject received prior treatment of a TKI that is a moderate/strong inducer or inhibitor of CYP3A, such as lorlatinib, the required wash-out period will be at least 14 days to allow complete wash out of its induction or inhibition effects. All side effects from prior treatments with ALKi, ROS1i, or TRKi must have resolved to grade ≤ 1 prior to starting treatment with repotrectinib; however, the most immediate treatment prior to enrollment does not have to be a TKI.
    a) Prior ALKi allowed include crizotinib, ceritinib, alectinib, brigatinib, lorlatinib, ensartinib and entrectinib
    b) Prior ROS1i allowed include crizotinib, ceritinib, lorlatinib, brigatinib, entrectinib, ensartinib, DS6051b, cabozantinib
    c) Prior TRKi allowed include entrectinib, larotrectinib, LOXO-195, DS6051b
    d) Other prior ALKi, ROS1i, and TRKi not listed above may be allowed after discussion with the Sponsor
11) Subjects with asymptomatic CNS metastases (treated or untreated) and/or asymptomatic leptomeningeal carcinomatosis are eligible to enroll if they satisfy the following criteria:
    a) Subjects requiring steroids at a stable or decreasing dose (≤ 12 mg/day dexamethasone or equivalent) for at least 14 days are eligible. Subjects on stable doses of levetiracetam (same dose for 14 days) are eligible to be enrolled.
    b) A minimum of 14 days must have elapsed from the completion of whole brain radiation treatment (WBRT) before the start of treatment with repotrectinib, and all side effects (with the exception of alopecia) from WBRT are resolved to CTCAE grade ≤ 1.
    c) A minimum of 7 days must have elapsed from the completion of stereotactic radiosurgery before the start of treatment with repotrectinib, and all side effects (with the exception of alopecia) from stereotactic radiosurgery are resolved to CTCAE grade ≤ 1.
12) Baseline laboratory values fulfilling the following requirements:

ANC	≥1500/mm^3 (1.5 × 10^9/L)
PLTs	≥100,000/mm^3 (100 × 10^9/L)
Hemoglobin	≥ 9.0 g/dL transfusions are allowed
Serum creatinine or creatinine clearance*	Within normal limits or > 40 mL/min
Total serum bilirubin	< 1.5 × ULN
Liver transaminases (ASTs/ALTs)	< 2.5 × ULN; < 5 × ULN if liver metastases are present
ALP	< 2.5 × ULN; < 5 × ULN if liver and/or bone metastasis are present
Serum calcium, magnesium, and potassium	Normal or CTCAE grade ≤ 1 with or without supplementation

Abbreviations: ALP, alkaline phosphatase; ANC, absolute neutrophils count; AST/ALT, aspartate aminotransferase/alanine aminotransferase; CTCAE, Common Terminology Criteria for Adverse Events; PLTs, platelets; ULN = upper limit of normal

* Calculated by Cockcroft and Gault's formula: (140 – age [yr]) × body weight [Kg] × 1.23 × (0.85 if female) / serum creatinine [µmol/L].

13) Reproductive Status

a) Investigators shall counsel women of childbearing potential (WOCBP) (as defined in Section 6.4.4) participants, and male participants who are sexually active with WOCBP, on the importance of pregnancy prevention, the implications of an unexpected pregnancy, and the potential of fetal toxicity occurring due to transmission of study intervention, present in seminal fluid, to a developing fetus, even if the participant has undergone a successful vasectomy or if the partner is pregnant or breastfeeding.

b) The investigator shall evaluate the effectiveness of the contraceptive method in relationship to the first dose of study intervention.

c) Local laws and regulations may require the use of alternative and/or additional contraception methods.

d) Female Participants:

i) Female participants must have documented proof that they are not of childbearing potential.

ii) Women who are not of childbearing potential (as defined in Section 6.4.4) are exempt from contraceptive requirements.

iii) WOCBP must have a negative highly sensitive serum or urine pregnancy test (minimum sensitivity 25 IU/L or equivalent units of human chorionic gonadotropin) within 24 hours prior to the start of study intervention. Cycle 1 Day 1 (C1D1) testing (urine or serum) is optional if screening serum test was done within 24 hours of C1D1.

iv) If a urine test cannot be confirmed as negative (eg, an ambiguous result), a serum pregnancy test is required. In such cases, the participant must be excluded from participation if the serum pregnancy result is positive.

v) Additional requirements for pregnancy testing during and after study intervention are in Table 25.

vi) The investigator is responsible for review of medical history, menstrual history, and recent sexual activity to potentially decrease the risk for inclusion of a woman with an undetected pregnancy.

vii) WOCBP and male participants who are sexually active with WOCBP must agree to follow instructions for method(s) of contraception as described below and included in the informed consent form (ICF).

viii) Due to a potential loss of effectiveness of hormonal contraceptives caused by interaction with study intervention, if WOCBP use hormonal contraceptives (including oral hormonal contraceptives), they must use either another form of non-hormonal highly effective contraception or a reliable barrier method (see Section 6.4.4). A female participant is eligible to participate if she is not pregnant or breastfeeding, and at least 1 of the following conditions applies:

(a) Is not a WOCBP

OR

(b) Is a WOCBP who agrees to avoid pregnancy during the study and using a contraceptive method that is highly effective (with a failure rate of < 1% per year), with user independent methods, as described in Section 6.4.4, during the intervention period and for at least 2 months post dose and agrees not to donate eggs (ova, oocytes) for the purpose of reproduction for the same period.

ix) Hormonal contraception must begin 7 days prior to the first dose of administration.

x) Additional pregnancy test (conducted locally) at 2 months post-last dose is highly recommended.

e) Male participants who are sexually active with WOCBP must agree to follow instructions for method(s) of contraception as described below and included in the ICF.

i) Azoospermic males are not exempt from contraceptive requirements and will be required to always use a latex or other synthetic condom during any sexual activity (eg, vaginal, anal, oral) with WOCBP, even if the participant has undergone a successful vasectomy or if the partner is pregnant or breastfeeding.

ii) Male participants are required to use a latex condom during the intervention period and for at least 4 months after the last dose of study intervention.

iii) Female partners of male participants should be advised to use a highly effective method of contraception during the study intervention period and for at least 4 months after the last dose of study intervention for the male participant.

iv) Male participants with a pregnant or breastfeeding partner must agree to remain abstinent from sexual activity or use a male latex condom during any sexual activity (eg, vaginal, anal, oral) during the intervention period and for at least 4 months after the last dose of study intervention.

v) Male participants must refrain from donating sperm during the intervention period and for at least 4 months after the last dose of study intervention.

vi) Breastfeeding partners of male participants should be advised to consult their health care provider about using appropriate highly effective contraception during the time the male participant is required to use latex condoms.

14) Capability to swallow capsules intact (without chewing, crushing, or opening).

15) Life expectancy ≥3 months.

16) Willingness and ability to comply with scheduled visits, treatment plan, laboratory tests, and other study procedures.

5.2 Inclusion Criteria for Phase 2

For country-specific requirements, please refer to APPENDIX 8.

1) Histologically or cytologically confirmed diagnosis of locally advanced, or metastatic solid tumor (including primary CNS tumors) that harbors a *ROS1* or *NTRK1-3* gene fusion. Note: Locally advanced disease is defined as Stage III when subject is not a candidate for surgery, radiation, or multi-modality therapy and metastatic disease is defined as Stage IV per the American Joint Committee on Cancer Eighth Edition Cancer Staging Manual guidelines (Rami-Porta 2017).

2) Subject must have a documented *ROS1* or *NTRK1-3* gene fusion determined by tissue-based local testing using either:
   a) a next-generation sequencing (NGS) or quantitative polymerase chain reaction (qPCR) test will be accepted to determine molecular eligibility.
      i) Adequate tumor tissue needs to be sent to the Sponsor designated central diagnostic laboratory for retrospective confirmation by a central diagnostic laboratory test selected by the Sponsor. In cases where archived tumor tissue is not available, a fresh biopsy should be obtained at Screening or as soon as possible after enrollment. See the Study Laboratory Manual for details.
      ii) If NGS was used, the partner of the fusion target gene needs to be identified and reported.
      iii) Retrospective confirmation by a central diagnostic laboratory test selected by the Sponsor is not required if ROS1 or NTRK1-3 gene fusion were determined by the Repotrectinib clinical trial assay (CTA).

      OR

   b) a FISH test AND prospective confirmation of fusion status by a central diagnostic laboratory test selected by the Sponsor PRIOR to enrollment will be accepted to determine molecular eligibility.
      i) Adequate tumor tissue must be sent to the Sponsor designated central diagnostic laboratory for prospective confirmation by a central diagnostic laboratory test selected by the Sponsor PRIOR to enrollment. In cases where archived tumor tissue is not available, a fresh biopsy should be obtained for this purpose. See the Study Laboratory Manual for details.

      All tests need to be performed in a Clinical Laboratory Improvement Amendments (CLIA) laboratory or equivalently accredited diagnostic laboratory.

3) Subjects must have a performance score as follows based on age at time of enrollment:
   a) Eastern Cooperative Oncology Group (ECOG) PS 0–1 (≥18 years)
   b) Karnofsky score of at least 50 (16 to <18 years)
   c) Lansky score of at least 50 (<16 years)

   Subjects who are unable to walk because of paralysis or tumor pain, but who are in a wheelchair, will be considered ambulatory for the purpose of assessing the performance score.

4) Age ≥12 (or as required by local regulations).
5) Willing and able to provide written institutional review board (IRB)/institutional ethics committee-approved Informed Consent, or an Informed Consent signed by a parent or legal guardian accompanied by an Assent signed by the participant as required by local regulations.
6) At least 1 measurable target lesion according to RECIST (Version 1.1) prospectively confirmed by Blinded Independent Central Radiology Review (BICR), selected by Sponsor, PRIOR to enrollment. Subjects with CNS-only measurable disease ≥10 mm as defined by RECIST (Version 1.1) are eligible.

7) Subjects with advanced solid tumors harboring ROS1, NTRK1, NTRK2, or NTRK3 rearrangement will be assigned into 6 distinct expansion (EXP) cohorts provided all inclusion and exclusion criteria are met.

- **EXP-1: ROS1 TKI-naïve *ROS1*+ NSCLC (n=110).**
  - No prior exposure to ROS1 TKI is allowed.
  - Up to one prior line of chemotherapy OR immunotherapy is allowed (chemo- or immunotherapy-based combination regimen is considered as one line of treatment).
- **EXP-2: 1 Prior ROS1 TKI AND 1 Platinum-based Chemotherapy *ROS1*+ NSCLC (n=120).**
  - Disease progression or intolerant to one prior line of ROS1 TKI.
  - ROS1 TKIs used in a prior line of treatment are limited to crizotinib, ceritinib, entrectinib, or lorlatinib. Note: Any previous exposure to a ROS1 TKI is considered as one prior line of TKI treatment (eg, if the same ROS1 TKI was given before and after a chemotherapy or other systemic therapy, it is considered as 2 prior TKIs and the subject would not be eligible for EXP-2).
  - In addition, the subject must have received one prior line of platinum-based chemotherapy OR one prior line of platinum-based chemotherapy in combination with immunotherapy before or after a ROS1 TKI (Note: subject is not eligible if he/she has been treated with more than one line of chemotherapy OR has received immunotherapy alone).
- **EXP-3: 2 Prior ROS1 TKIs and NO Chemotherapy or Immunotherapy *ROS1*+ NSCLC (n=80).**
  - Disease progression or intolerant to 2 prior lines of a ROS1 TKI treatment.
  - ROS1 TKI used in prior lines of treatment are limited to crizotinib, ceritinib, entrectinib, lorlatinib, brigatinib, ensartinib, or cabozantinib. Other prior ROS1 TKI agents that are not listed may be allowed after discussion with the Sponsor Medical Monitor. Note: Any previous exposure to a ROS1 TKI is considered as one prior line of TKI treatment (eg, if 2 different ROS1 TKIs are utilized, or the same ROS1 TKI was given before and after a chemotherapy or other systemic therapy, it is considered as 2 prior TKIs and the subject would be eligible).
  - No prior lines of chemotherapy or immunotherapy are allowed.
- **EXP-4: 1 Prior ROS1 TKI and NO Chemotherapy or Immunotherapy *ROS1*+ NSCLC (n=120).**
  - Disease progression or intolerant to one prior line of a ROS1 TKI.
  - ROS1 TKIs used in a prior line of treatment are limited to crizotinib, ceritinib, entrectinib, or lorlatinib. Note: Any previous exposure to a ROS1 TKI is considered as one prior line of TKI treatment (eg, if the same ROS1 TKI was given before and after a chemotherapy or other systemic therapy, it is considered as 2 prior TKIs and the subject would not be eligible for EXP-4).
  - Note: No prior lines of chemotherapy or immunotherapy are allowed.
- **EXP-5: TRK TKI-naïve *NTRK*+ solid tumors (n=approximately 80).**
  - No prior TRK TKI is allowed.

- Any number of prior lines on chemo or immunotherapy is allowed.
- As of global protocol amendment Version 15.0 (or equivalent version for respective countries), subjects with NSCLC are not allowed.

- **EXP-6: TRK TKI-pretreated *NTRK*+ solid tumors (n=approximately 120):**
  - Disease progression or intolerant to 1 or 2 prior TRK TKIs only.
  - TRK TKI used in prior lines or treatment are limited to entrectinib, larotrectinib, selitrectinib (LOXO-195), and cabozantinib. Other prior TRKi not listed above may be allowed after discussion with the Sponsor Medical Monitor. Note: Any previous exposure of a TRK TKI is considered as one prior line of TKI treatment, eg, if 2 different TRK TKIs are utilized or the same TRK TKI was used before and after a chemo- or other systemic therapy, it is considered as 2 prior TKIs and the patient would be eligible.
  - Any prior lines on chemo or immunotherapy allowed.
  - As of global protocol amendment Version 15.0 (or equivalent version for respective countries), subjects with NSCLC are not allowed.

8) Required wash-out time that is related to prior therapies before starting repotrectinib treatment:
   a) If the immediate prior treatment was a ROS1 or TRK TKI: 7 days or 5 half-lives (whichever is shorter) must have elapsed since completion of treatment with the last TKI for subjects enrolling into the pretreated expansion cohorts (EXP-2, -3, -4, and -6). All side effects from prior treatments with ROS1 or TRK TKI must have resolved to grade ≤1 prior to starting treatment with repotrectinib.
   b) At least 14 days or 5 half-lives (whichever is shorter) must have elapsed after discontinuation of prior systemic chemotherapy (or at least 42 days for prior nitrosoureas and mitomycin C) and all side effects from prior treatments must have resolved to grade ≤1 with the exception of alopecia.
   c) At least 14 days must have elapsed after discontinuation of prior immunotherapy and all immune-related side effects from prior immunotherapy must have resolved to grade ≤1.
9) Subjects with asymptomatic CNS metastases (treated or untreated) and/or asymptomatic leptomeningeal carcinomatosis are eligible to enroll if they satisfy the following criteria:
   a) Subjects requiring steroids at a stable or decreasing dose (≤ 12 mg/day dexamethasone or equivalent) for at least 14 days are eligible.
   b) Subjects on stable doses of levetiracetam (same dose for 14 days).
   c) A minimum of 14 days must have elapsed from the completion of whole brain radiation treatment (WBRT) before the start of treatment with repotrectinib, and all side effects (with the exception of alopecia) from WBRT are resolved to grade ≤ 1.
   d) A minimum of 7 days must have elapsed from the completion of stereotactic radiosurgery before the start of treatment with repotrectinib, and all side effects (with the exception of alopecia) from stereotactic radiosurgery are resolved to grade ≤ 1.
10) Baseline laboratory values fulfilling the following requirements:

ANC	≥1500/mm^3 (1.5 × 10^9/L)
PLTs	≥100,000/mm^3 (100 × 10^9/L) independent of platelets transfusion support for at least 7 days prior to dosing

Hemoglobin	≥ 9.0 g/dL independent of transfusion support for at least 7 days prior to dosing
Creatinine clearance*	> 40 mL/min
Total serum bilirubin	< 1.5 × ULN
Liver transaminases (ASTs/ALTs)	< 2.5 × ULN; < 5 × ULN if liver metastases are present
ALP	< 2.5 × ULN; < 5 × ULN if liver and/or bone metastasis are present
Serum calcium, magnesium, and potassium	Normal or CTCAE grade ≤ 1 with or without supplementation

Abbreviations: ALP, alkaline phosphatase; ANC, absolute neutrophils count; AST/ALT, aspartate aminotransferase/alanine aminotransferase; CTCAE, Common Terminology Criteria for Adverse Events; PLTs, platelets; ULN = upper limit of normal

* Calculated by Cockcroft and Gault's formula: (140 – age [yr]) × body weight [Kg] × 1.23 × (0.85 if female) / serum creatinine [µmol/L].

11) Reproductive status

a) Investigators shall counsel women of childbearing potential (WOCBP) (as defined in APPENDIX 1) participants, and male participants who are sexually active with WOCBP, on the importance of pregnancy prevention, the implications of an unexpected pregnancy, and the potential of fetal toxicity occurring due to transmission of study intervention, present in seminal fluid, to a developing fetus, even if the participant has undergone a successful vasectomy or if the partner is pregnant or breastfeeding.

b) Investigators shall evaluate the effectiveness of the contraceptive method in relationship to the first dose of study intervention.

c) Local laws and regulations may require the use of alternative and/or additional contraception methods.

d) Female Participants:

i) Female participants must have documented proof that they are not of childbearing potential.

ii) Women who are not of childbearing potential (as defined in Section 6.4.4) are exempt from contraceptive requirements.

iii) WOCBP must have a negative highly sensitive serum or urine pregnancy test (minimum sensitivity 25 IU/L or equivalent units of human chorionic gonadotropin) within 24 hours prior to the start of study intervention. Cycle 1 Day 1 (C1D1) testing (urine or serum) is optional if screening serum test was done within 24 hours of C1D1.

iv) If a urine test cannot be confirmed as negative (eg, an ambiguous result), a serum pregnancy test is required. In such cases, the participant must be excluded from participation if the serum pregnancy result is positive.

v) Additional requirements for pregnancy testing during and after study intervention are in Table 26.

vi) The investigator is responsible for review of medical history, menstrual history, and recent sexual activity to potentially decrease the risk for inclusion of a woman with an undetected pregnancy.

vii) WOCBP and male participants who are sexually active with WOCBP must agree to follow instructions for method(s) of contraception as described below and included in the informed consent form (ICF).

viii) Due to a potential loss of effectiveness of hormonal contraceptives caused by interaction with study intervention, if WOCBP use hormonal contraceptives (including oral hormonal contraceptives), they must use either another form of non-hormonal highly effective contraception or a reliable barrier method (see APPENDIX 1). A female participant is eligible to participate if she is not pregnant or breastfeeding, and at least 1 of the following conditions applies:

(a) Is not a WOCBP

OR

(b) Is a WOCBP who agrees to avoid pregnancy during the study and using a contraceptive method that is highly effective (with a failure rate of < 1% per year), with user independent methods, as described in APPENDIX 1), during the intervention period and for at least 2 months post dose and agrees not to donate eggs (ova, oocytes) for the purpose of reproduction for the same period.

ix) Hormonal contraception must begin 7 days prior to the first dose of administration.

x) Additional pregnancy test (conducted locally) at 2 months post-last dose is highly recommended.

e) Male participants who are sexually active with WOCBP must agree to follow instructions for method(s) of contraception as described below and included in the ICF:

i) Azoospermic males are not exempt from contraceptive requirements and will be required to always use a latex or other synthetic condom during any sexual activity (eg, vaginal, anal, oral) with WOCBP, even if the participant has undergone a successful vasectomy or if the partner is pregnant or breastfeeding.

ii) Male participants are required to use a latex condom during the intervention period and for at least 4 months after the last dose of study intervention.

iii) Female partners of male participants should be advised to use a highly effective method of contraception during the study intervention period and for at least 4 months after the last dose of study intervention for the male participant.

iv) Male participants with a pregnant or breastfeeding partner must agree to remain abstinent from sexual activity or use a male latex condom during any sexual activity (eg, vaginal, anal, oral) during the intervention period and for at least 4 months after the last dose of study intervention.

v) Male participants must refrain from donating sperm during the intervention period and for at least 4 months after the last dose of study intervention.

vi) Breastfeeding partners of male participants should be advised to consult their health care provider about using appropriate highly effective contraception during the time the male participant is required to use latex condoms.

12) Ability to swallow capsules intact (without chewing, crushing, or opening).

13) Life expectancy ≥ 3 months.

14) Willingness and ability to comply with scheduled visits, treatment plan, laboratory tests, and other study procedures.

5.3 Exclusion Criteria for Phase 1 and Phase 2

For country-specific requirements, please refer to APPENDIX 8.

1) Concurrent participation in another therapeutic clinical trial.
2) Symptomatic brain metastases or leptomeningeal involvement.
3) History of previous cancer requiring therapy within the previous 2 years, except for squamous cell or basal-cell carcinoma of the skin, or any in situ carcinoma that has been completely resected.
4) Major surgery within 4 weeks of start of repotrectinib treatment. Radiation therapy (except palliative to relieve bone pain) within 2 weeks of study entry. Palliative radiation (≤10 fractions) must have been completed at least 48 hours prior to study entry.
5) Clinically significant cardiovascular disease (either active or within 6 months prior to enrollment): myocardial infarction, unstable angina, coronary/peripheral artery bypass graft, symptomatic congestive heart failure (New York Heart Association Classification Class ≥ II), cerebrovascular accident or transient ischemic attack, symptomatic bradycardia, requirement for anti-arrhythmic medication. Ongoing cardiac dysrhythmias of CTCAE grade ≥2.
6) Any of the following cardiac criteria:
   a) Mean resting corrected QT interval (ECG interval measured from the onset of the QRS complex to the end of the T wave) for heart rate (QTcF) > 470 msec obtained from 3 ECGs, using the screening clinic ECG machine-derived QTc value
   b) Any clinically important abnormalities in rhythm, conduction or morphology of resting ECG (eg, complete left bundle branch block, third degree heart block, second degree heart block, PR interval > 250 msec)
   c) Any factors that increase the risk of QTc prolongation or risk of arrhythmic events such as heart failure, hypokalemia, congenital long QT syndrome, family history of long QT syndrome, or any concomitant medication known to prolong the QT interval (APPENDIX 6)
7) Known active infections requiring ongoing treatment (bacterial, fungal, viral including human immunodeficiency virus positivity).
8) Gastrointestinal disease (eg, Crohn's disease, ulcerative colitis, short gut syndrome) or other malabsorption syndromes that would impact on drug absorption.
9) Peripheral neuropathy, paresthesia, dizziness, dysgeusia, muscle weakness, ataxia grade ≥2.
10) History of extensive, disseminated, bilateral, or presence of CTCAE grade 3 or 4 interstitial fibrosis or interstitial lung disease including a history of pneumonitis, hypersensitivity pneumonitis, interstitial pneumonia, interstitial lung disease, obliterative bronchiolitis, and pulmonary fibrosis. Subjects with history of prior radiation pneumonitis are not excluded.
11) Other severe acute or chronic medical or psychiatric condition or laboratory abnormality that may increase the risk associated with study participation or study drug administration, or that may interfere with the interpretation of study results and, in the judgment of the Investigator, would make the subject inappropriate for entry into this study, or could compromise protocol objectives in the opinion of the Investigator and/or the Sponsor.
12) Current use or anticipated need for drugs that are known to be strong CYP3A inhibitors or inducers as listed in APPENDIX 6.

13) Hypersensitivity to the active substance or to any of the excipients.
14) **Additional exclusion criteria for subjects participating in the midazolam DDI sub-study**: in addition to the strong CYP3A inhibitors or inducers listed in APPENDIX 6, subjects should not be taking any moderate inhibitors or inducers of CYP3A (moderate CYP3A inhibitors eg: erythromycin, verapamil, atazanavir, fluconazole, darunavir, diltiazem, delavirdine, aprepitant, imatinib, tofisopam, ciprofloxacin, cimetidine; moderate CYP3A inducers eg: bosentan, efavirenz, etravirine, modafinil) within 2 weeks of the lead-in midazolam dosing and until the DDI assessment portions is completed on Cycle 1 Day 23. Please refer to midazolam product package insert for complete information.

6 STUDY TREATMENTS

6.1 Repotrectinib Drug Supply

6.1.1 Formulation and Packaging

Repotrectinib will be supplied for oral administration as 40-mg capsules in bottles containing 30 capsules. Study medication will be supplied by the Sponsor.

6.1.2 Preparation and Dispensing

Only qualified personnel who are familiar with procedures that minimize undue exposure of the study drug to them and to the environment should undertake the preparation, handling, and safe disposal of chemotherapeutic agents.

Repotrectinib will be provided in bottles containing 40-mg capsules, individually. Each bottle will contain 30 capsules. Site personnel must ensure that subjects clearly understand the directions for self-medication. Subjects should be given sufficient supply to last until their next study visit. Repotrectinib will be dispensed at the beginning of each treatment cycle (or as otherwise indicated). For the Phase 2 portion of the study, repotrectinib will be dispensed in Cycle 1 at Day 1 and Day 15, and then at the beginning of each subsequent treatment cycle thereafter. Subjects should be instructed to keep their medication in the bottles provided and not transfer it to any other container.

For the midazolam DDI sub-study, midazolam will be supplied as an oral syrup (2 mg/mL). Midazolam will be administered at dose of 5 mg during the DDI assessment periods on Cycle 1 Day -2, and Cycle 1 Day 22.

Drug accountability should be assessed at each study visit. The subject dosing diary and remaining study drug amount should be reviewed as referred in the Pharmacy Manual for Phase 1 and Phase 2 study.

6.1.3 Administration

All subjects will receive single-agent repotrectinib.

6.1.3.1 Dose Administration (Phase 1)

The starting dose for repotrectinib in Phase 1 will be 40 mg. Repotrectinib will be administered once or twice daily in 28-day cycles with the exception of Cycle 1, which was 21 days in Phase 1a

and Phase 1b and will be 28 days in Phase 1c. Repotrectinib doses that are to be administered twice daily (BID) for all study phases will be given approximately twelve hours apart.

Administration will be performed on an outpatient basis.

Initially in the Phase 1 dose escalation study (Cohorts 1-6), repotrectinib was taken with at least 8 oz (240 mL) of water on an empty stomach (ie, subject should refrain from food and beverages [except water] for at least 1 hour prior to dosing and for at least 2 hours after dosing). Subjects were instructed to take their medication at approximately the same time each day and to not take more than the prescribed dose at any time. However, a variance of up to 12 hours is allowed for any given dose, rather than miss a day's dose. If a subject misses a daily dose, they must be instructed not to "make it up" the next day. If a subject vomits at any time after taking a dose, they must be instructed not to "make it up," but to resume subsequent doses the next day as prescribed. If a subject inadvertently takes 1 extra dose during a day, the subject should not take the next dose of repotrectinib. Subjects should also be instructed to swallow the trial medication whole and not chew the capsule prior to swallowing. No capsule should be ingested if it is broken, cracked, or otherwise not intact. Doses may be modified according to Table 8, Table 9, and Table 10.

A food effect sub-study was conducted to evaluate the effect of a high-fat, high-calorie breakfast on repotrectinib pharmacokinetics at the dose level where preliminary anti-tumor activity (partial response) was observed by the Investigator. Each subject served as his/her own control.

For those subjects scheduled to receive the "fed" treatment first, a test breakfast meal (described below) was provided and consumed over 30 minutes on Cycle 0 Day -7. Study drug was administered with approximately 8 oz (240 mL) of water 30 minutes after the start of the meal. No additional food was allowed until at least 2 hours post-dose. Subjects then took repotrectinib under the "fasted" condition (see below) on Cycle 1 Day 1.

For subjects scheduled to receive the "fasted" treatment first, study drug was administered with 8 oz (240 mL) of water on Cycle 0 Day -7. No food was allowed 1 hour before the dose and for an additional 2 hours post-dose. For either treatment day, water was allowed *ad libitum* except for 1 hour before and 1 hour after drug administration. Subjects then took repotrectinib under the "fed" condition (see below) on Cycle 1 Day 1.

For all subjects participating in the food effect sub-study, there were no repotrectinib doses administered after Cycle 0 Day -7 and before Cycle 1 Day 1, and after Cycle 1 Day 1 and before Cycle 1 Day 4. For all subjects participating in the food effect sub-study, repotrectinib was administered at Cycle 1 Day 4 on an empty stomach. No food except water was allowed 1 hour before and 2 hours after drug administration. Water was allowed *ad libitum* except for 1 hour before and 1 hour after drug administration.

The test meal to be consumed was a high-fat (approximately 50% of total caloric content of the meal) and high-calorie (approximately 800 to 1000 calories) meal. This test meal should derive approximately 150, 250, and 500-600 calories from protein, carbohydrate, and fat, respectively. An example test meal would be 2 eggs fried in butter, 2 strips of bacon (may be replaced with ham and cheese of similar caloric content), 2 slices of toast with butter, 4 oz of hash brown potatoes,

and 8 oz of whole-fat milk. Substitutions to this test meal can be made after discussion with the Sponsor, as long as the meal provides a similar number of calories from protein, carbohydrate, and fat and has comparable meal volume and viscosity (if substitutions are made, the contents of the meal will be documented by a dietitian or designee to confirm it matches the FDA requirements for protein, carbohydrate, and fat described above). However, it is understood that some subjects may not be able to consume the entire meal. Study staff should record the percent of the test meal breakfast and the time it takes to be consumed.

Based on the results of the food effect sub-study (Section 3.3), further dosing in the Phase 1c study starting in Cohort 7 and above will be administered with food. There will be no further fasting restrictions with repotrectinib and study drug should be administered with at least 8 oz of water and with or after a standard meal (taken with food, or within one hour after food intake).

Subjects may continue repotrectinib treatment after objective progression of disease is determined if the subject is continuing to experience clinical benefit, in the opinion of the Investigator, and after discussion with the Sponsor.

The study Investigator may implement repotrectinib dose interruption and/or reduction in order to ensure subject safety at any time in the course of the study (see Section 6.2).

At Day 1 of each cycle visit, and at the end of the treatment study, all unused or partially used bottles must be returned by subjects to the Investigator and the Sponsor will provide instructions as to disposition of any unused repotrectinib. If the Sponsor authorizes destruction at the study site, the Investigator must ensure that the materials are destroyed in compliance with applicable environmental regulations, institutional policy, and any special instructions provided by the Sponsor. Destruction must be adequately documented.

Dose Administration for Midazolam DDI Sub-study

For the midazolam DDI sub-study, six PK evaluable subjects will receive a single 5 mg oral dose of midazolam (as an oral syrup) on Cycle 1 Day -2 (ie, two days before starting repotrectinib at the RP2D) under fasted conditions (no food 8 hours before through 2 hours after dosing of MDZ). Starting on Cycle 1 Day 1, subjects will begin daily treatment of 160 mg repotrectinib QD followed by 160 mg BID on Cycle 1 Day 15. On Cycle 1 Day 22, another single 5 mg oral dose of midazolam will be administered concurrently with repotrectinib (morning dose) under fasted condition (no food 8 hours before through 2 hours after dosing of MDZ). Repotrectinib can be taken without regard to food between Cycle 1 Day 1 to Cycle 1 Day 21, as well as after completion of the DDI assessment.

Midazolam will be supplied as an oral syrup (2 mg/mL). Qualified personnel will administer midazolam syrup (2.5 mL to provide 5 mg of midazolam) using an oral disposable syringe followed by 8 oz (240 ml) of ambient temperature water.

6.1.3.2 Dose Administration (Phase 2)

Subjects in Phase 2 will receive repotrectinib as a single agent at RP2D as defined below:

- 160 mg QD for the first 14 days, may increase to 160 mg BID. Repotrectinib can be taken with or without food.

(Subjects must meet all below criteria while on 160 mg QD prior to dose increasing to 160 mg BID: No grade ≥3 treatment-related AE, unmanageable grade ≥2 dizziness, ataxia or paresthesia; or grade ≥3 clinically significant lab abnormalities).

Subjects must have received repotrectinib at 160 mg QD for the first 14 days and tolerability must be evaluated by his/her treating physician on C1D15 prior to increasing dose to 160 mg BID.

Dosing should be at a consistent time each day; QD and BID dosing will be separated by approximately 24 hours (±2 hours) and 12 hours (±1 hours), respectively. The subject will keep a daily diary to record dosing compliance. Doses that are late by more than 12 hours should not be taken and recorded in the dosing diary as missed. Doses that are late by more than 6 hours should be skipped and recorded in the dosing diary as missed.

6.1.4 Storage and Accountability

Repotrectinib capsules should be stored under the United States Pharmacopeia (USP) controlled room temperature conditions at 20°C to 25°C (68°F to 77°F), with excursions permitted between 15°C to 30°C (59°F to 86°F). Medication should be kept in a secured locked area at the study site in accordance with applicable regulatory requirements. Subjects should be instructed to keep their medication in its original container and stored at 15°C to 30°C (59°F to 86°F). Returned medication should be stored separately from medication that is yet to be dispensed.

Investigators and site staff are reminded to document storage room temperatures daily (ie, manual or electronic systems that alert of any excursions) and ensure that thermometers are working correctly as required for proper storage of repotrectinib. Any temperature excursions, defined as storage conditions at lower than 15ºC or exceeding 30ºC (ie, should be reported to the Sponsor).

The study drug must be stored as indicated. Deviations from the storage requirements, including any actions taken, must be documented and reported to the Sponsor. Once a deviation is identified, the IP must be quarantined and not used until the Sponsor provides documentation of permission to use the IP. The temperature of all locations where IP are stored should be monitored continuously and verified as appropriate per the site processes, preferably using a thermometer that measures minimum and maximum temperatures. Storage temperature should be recorded and monitored consistently by the site personnel. If a continuous measuring instrument/device is not available, daily temperature (and as applicable, humidity) recordings must be taken to ensure compliance with the Sponsor requirements and/or site standard operating procedures (SOPs), where applicable. If the site has its own SOPs, the most conservative standard will be applied, so that the Sponsor requirements are always met.

6.1.4.1 Temperature Excursions

If a temperature excursion to lower than 15ºC or exceeding 30ºC (ie, lower than 59°F or exceeding 86°F) occurs, the site should immediately contact their study monitor to alert them and escalate to the Sponsor for evaluation and disposition. Once a deviation is identified, the IP must be

quarantined and not used until the Sponsor provides documentation of permission to use the IP. When reporting an excursion, the following information, at a minimum, should be included:

- Protocol number
- Site number
- Length of time for the excursion
- Minimum/maximum temperatures for the excursion
- Impacted container numbers or lot numbers
- Confirmation of whether impacted containers were dispensed/administered to subjects
- Copy of temperature monitoring log if available

The site should not use the supplies until a disposition is received from the Sponsor *via* the study management. The IP should continue to be stored in its appropriate location pending the disposition from the Sponsor. If the materials are rejected, the Sponsor will initiate a replacement shipment to the site.

6.2 Dose Modifications

6.2.1 Recommendation for Dose Modifications

For dose modifications due to a DLT in Cycle 1 (Phase 1), refer to Section 3.2.3.5.

Every effort should be made to administer the study treatment on the planned dose and schedule.

In the event of significant toxicity dosing may be withheld and/or reduced as described in Table 14. In the event of multiple toxicities, dose modification should be based on the worst toxicity observed. Subjects are to be instructed to notify Investigators at the first occurrence of any adverse symptom.

Dose modifications may occur in 2 ways:

- Within a cycle: dosing interruption until adequate recovery followed by dose reduction, if required, during a given treatment cycle; this may persist delaying the start of a new cycle.
- In the next cycle: dose reduction may be required in a subsequent cycle based on toxicity experienced in the previous cycle Table 14.

6.2.2 Dose Interruptions

Appropriate follow-up assessments should be done until adequate recovery occurs as assessed by the Investigator. Criteria required before treatment can resume are described in the dose modification table (see Table 14).

Doses may be held as needed until toxicity resolution. Depending on when the adverse event resolved, a treatment interruption may lead to the subject missing all subsequent planned doses within that same cycle or even to delay the initiation of the subsequent cycle.

If the adverse event that led to the treatment interruption recovers within the same cycle, then re-dosing in that cycle is allowed. Doses omitted for toxicity are not replaced within the same cycle.

The need for a dose reduction at the time of treatment resumption should be based on the criteria defined in Table 14 unless otherwise agreed to following discussion between the Investigator and the Sponsor Medical Monitor.

In the event of a treatment interruption for reasons other than treatment-related toxicity (eg, elective surgery) lasting ≥14 days, treatment resumption will be decided in consultation with the Sponsor Medical Monitor.

Subjects not recovering from repotrectinib-related toxicity within 28 days since the last dose will discontinue repotrectinib treatment.

All study visits (visit date calculations) are based off of the Cycle 1 Day 1 visit date, regardless of dose interruptions, and evaluations are to be performed according to the Study Calendars (Section 8.5). Every effort should be made to maintain the tumor assessments scheduling as described in the Study Calendars (Section 8.5) (ie, every 2 cycles [± 1 week] versus the date of Cycle 1 Day 1).

6.2.3 Dose Reductions

6.2.3.1 Dose Modification for Phase 1:

For dose reductions in Cycle 1 to manage a DLT refer to Section 3.2. Following dosing interruption or cycle delay due to toxicity, the repotrectinib dose may need to be reduced when treatment is resumed.

In cases where no specific dose adjustment requirements for CTCAE grade 2 treatment-related toxicity exist, Investigators should always manage their subjects according to their medical judgment, which may include dose reduction or interruption based on the particular clinical circumstances in consultation with the the Sponsor Medical Monitor.

Subjects experiencing recurrent and intolerable CTCAE grade 2 toxicity may resume dosing at the next lower dose level once recovery to CTCAE grade ≤1 or baseline is achieved.

For the Phase 1 portion of the study, Dose reduction of repotrectinib by 1 and, if needed, 2 dose levels (Table 8, Table 9, Table 10, and Table 14) will be allowed depending on the type and severity of toxicity encountered. As a guide, subjects currently being treated at 120 mg QD should dose reduce one dose level to 80 mg QD. If a subject is being treated at 160 mg QD and requires dose reduction, they should dose reduce to 120 mg QD initially and then 80 mg QD if needed.

Subjects currently on the BID schedule may have a dose reduction to a QD schedule provided the total daily dose is lower than their current total daily dose, ie, 80 mg BID may dose reduce to 40 mg BID or 120 mg QD, whereas subjects on 120 mg BID may dose reduce first to either 160 mg QD or 80 mg BID and then subsequently to 120 mg QD.

Subjects requiring more than 2 dose reductions will be discontinued from the treatment and entered into the follow-up phase, unless otherwise agreed between the Investigator and the Sponsor Medical Monitor. All dose modifications/adjustments must be clearly documented in the subject's source notes and CRF.

Once a dose has been reduced for a given subject, all subsequent cycles should be administered at that dose level, unless further dose reduction is required.

Dose re-escalation is not allowed in the Phase 1 study. Subjects experiencing a DLT may resume dosing at the next lower dose level (if applicable) once adequate recovery is achieved.

6.2.4 Dose Modification for Phase 2:

For Phase 2 portion of the study, recommended dose reduction guidance is summarized in Table 13.

Table 13: Recommended Dose Reduction Guidance for Phase 2

Dose level	160 mg QD	160 mg BID
First dose reduction	120 mg QD	120 mg BID
Second dose reduction	80 mg QD	80 mg BID
If any further dose reduction is needed	Discuss with Medical Monitor	Discuss with Medical Monitor

Abbreviations: BID, twice daily; QD, once daily

Subjects requiring more than 2 dose reductions should be discussed with a Sponsor Medical Monitor to determine if further dose reduction is permitted. All dose modifications/adjustments must be clearly documented in the subject's source notes and CRF.

If 160 mg QD was not tolerated during first 14 days of dosing, dose escalation to 160 mg BID is not permitted, see below.

- Subjects must meet all below criteria while on 160 mg QD dosing prior to starting 160 mg BID: No grade ≥3 treatment-related AE, unmanageable grade ≥2 dizziness, ataxia or paresthesia; or grade ≥3 clinically significant lab abnormalities.

Dose re-escalation back to the previous dose level is allowed per Investigator discretion and must be discussed with a Sponsor Medical Monitor for appropriate subject management.

Subjects may continue repotrectinib treatment after radiographic disease progression is confirmed by BICR if the subject is continuing to experience clinical benefit, in the opinion of the Investigator, and after discussion with the Sponsor's Medical Monitor.

Subjects will be monitored closely for toxicity, and the dose of repotrectinib may be adjusted as indicated in Table 14.

For country-specific requirements, please refer to APPENDIX 8.

Intra-patient dose escalation in Phase 2:

Dose Escalation for subjects with CNS disease: CNS metastases are common in many solid tumors, such as lung, breast, colon, kidney, and malignant melanoma. Specifically, among *ALK+* NSCLC patients previously treated with crizotinib, the frequency of CNS metastases is approximately 60% (Ou 2016) and, similarly, the incidence of CNS metastasis ranged from the mid 30% to mid 55% among *ROS1+* NSCLC patients who had progressed on crizotinib (Ou 2019). Recent studies with alectinib, a second-generation ALK inhibitor, suggest that dose intensification may be necessary to overcome incomplete ALK inhibition in the CNS and prolong the durability

of responses in patients with CNS metastases, particularly those with leptomeningeal carcinomatosis (Gainor 2015). As such, for patients with CNS disease who have been on study for at least 2 cycles of treatment (ie, 8 weeks) with a best response of stable disease (SD) per RECIST Version 1.1 **AND** without treatment-related Grade ≥ 2 AEs, dose escalation up to 160 mg BID (if the subject was on 160 mg QD) will be allowed as per Investigator's discretion and after discussion with the Sponsor's Medical Monitor.

Table 14: Dose Modifications for Adverse Events (CTCAE version 4.03)

Toxicity	CTCAE Grade 1**	CTCAE Grade 2**	CTCAE Grade 3	CTCAE Grade 4
Hematologic	Continue at same dose level	Continue at same dose level	Withhold dose until toxicity is grade ≤ 2, or has returned to baseline, then resume treatment at the same dose level or reduce by 1 dose level as per the Investigator's discretion<br><br>Grade 3 lymphopenia without other dose-limiting events (eg, opportunistic infection) may continue study treatment without interruption	Withhold dose until toxicity is grade ≤ 2, or has returned to baseline, reduce the dose by 1 dose level and resume treatment<br><br>Grade 4 lymphopenia without other dose-limiting events (eg, opportunistic infection) may continue study treatment without interruption
Non-Hematologic AEs excluding dizziness ataxia, and paresthesia	Continue at same dose level	Continue at same dose level.<br><br>For prolonged or intolerable CNS toxicity, withhold dose until toxicity is grade ≤ 1 or has returned to baseline, then reduce by 1 dose level and resume treatment	Withhold dose until toxicity is grade ≤ 1 or has returned to baseline, then reduce by 1 dose level and resume treatment	Withhold dose until toxicity is grade ≤ 1 or has returned to baseline, then reduce by 1 dose level and resume treatment; or discontinue treatment as per the Investigator's discretion
Dizziness ataxia, and paresthesia	Continue at same dose level	Withhold current dose until toxicity is grade ≤1 or has returned to baseline OR reduce dose by 1 dose level immediately.	Withhold dose until toxicity is grade ≤ 1 or has returned to baseline, then reduce by 1 dose level and resume treatment	Withhold dose until toxicity is grade ≤ 1 or has returned to baseline, then reduce by 1 dose level and resume treatment; or discontinue treatment as per the Investigator's discretion after

Table 14: Dose Modifications for Adverse Events (CTCAE version 4.03)

Toxicity	CTCAE Grade 1**	CTCAE Grade 2**	CTCAE Grade 3	CTCAE Grade 4
				discussion with Medical Monitor
Pneumonitis (in the absence of disease progression, pulmonary embolism, positive cultures or radiation effect)	Asymptomatic, radiographic findings only: No need for dose adjustment. Initiate appropriate monitoring.<br><br>Symptomatic: Withhold current dose until toxicity has returned to baseline. Rule out infection and consider initiating treatment with corticosteroids. Then resume treatment at the same dose. Discontinue permanently if pneumonitis recurs or if failure to recover after 6 weeks of study treatment hold and steroid treatment	Withhold current dose until toxicity has returned to baseline. Rule out infection and consider initiating treatment with corticosteroids. Then resume treatment at 1 dose level lower.<br><br>Discontinue permanently if pneumonitis recurs or if failure to recover after 6 weeks of study treatment hold and steroid treatment	Discontinue treatment	Discontinue treatment
Prolonged QTc	Assess electrolytes and concomitant medications<br><br>Correct any electrolyte abnormalities, or hypoxia<br><br>Continue at the same dose level	Assess electrolytes and concomitant medications<br><br>Correct any electrolyte abnormalities, or hypoxia<br><br>Continue at the same dose level	Withhold dose<br><br>Assess electrolytes and concomitant medications<br><br>Correct any electrolyte abnormalities, or hypoxia<br><br>Upon recovery to grade ≤ 1: if no other cause for QTc prolongation is found or is considered drug-related resume treatment at 1 dose level lower	Discontinue treatment

Abbreviations: AE, adverse event; CTCAE, Common Terminology Criteria for Adverse Events

** In cases where no specific dose adjustments for CTCAE grade 1 or grade 2 treatment-related toxicity are provided, Investigators should always manage their subjects according to their medical judgment, which may include dose reduction or interruption based on the particular clinical circumstances.

6.3 Concomitant Medications

Clinical drug interaction studies for repotrectinib have been conducted with CYP3A4 strong inhibitor itraconazole, strong inducer rifampin, and substrate midazolam. All concomitant medications, blood products, as well as non-drug interventions (eg, paracentesis) received by subjects from date of a signed informed consent until the end of treatment (EOT) visit will be recorded on the CRF. A list of concomitant medications is to be provided to the Sponsor or their designee prior to study enrollment.

Note on COVID-19 Vaccination: The use of vaccines is not a restriction for this study. Currently enrolled and potential adult clinical trial subjects are allowed to receive the COVID-19 vaccine with no impact on their eligibility or ability to remain on study treatment. This guidance will also apply to pediatric and adolescent patients once vaccine approval is available for these populations. In addition, there is not a required wash-out period for the vaccine. The risk-benefit for COVID-19 vaccination while on study should be determined by the treating physician. Administration of the COVID-19 vaccine will be recorded in the medical history or the concomitant medications CRF, as applicable, as well as any adverse events related to the vaccine.

6.3.1 Strong CYP3A4 Inhibitors and Inducers and Sensitive Substrates of CYP3A4/CYP2B6

6.3.1.1 Medications Known to be Strong CYP3A4 Inhibitors and Inducers

An *in vitro* metabolism study with a panel of recombinant human cytochrome P450 isoforms (CYP450) suggests that repotrectinib is metabolized mainly by the CYP3A4 isoform.

- Inhibition of CYP3A4 isoenzymes may increase repotrectinib exposure leading to a potential increase in toxicities. The concomitant use of known strong CYP3A inhibitors is prohibited (APPENDIX 6).
  - Strong CYP3A inhibitors: boceprevir, clarithromycin, cobicistat, danoprevir and ritonavir, elvitegravir and ritonavir, grapefruit juice or grapefruit/grapefruit-related citrus fruits (eg, Seville oranges, pomelos), idelalisib, indinavir and ritonavir, itraconazole, ketoconazole, lopinavir and ritonavir, nefazodone, nelfinavir, paritaprevir and ritonavir and (ombitasvir and/or dasabuvir), posaconazole, ritonavir, saquinavir and ritonavir, telaprevir, telithromycin, tipranavir and ritonavir, troleandomycin, voriconazole.
- Repotrectinib metabolism may be induced when taking strong CYP3A inducers resulting in reduced plasma concentrations. Therefore, coadministration of repotrectinib with strong CYP3A inducers is prohibited (APPENDIX 6).
  - Strong CYP3A inducers: apalutamide, enzalutamide, mitotane, rifampin, phenytoin, carbamazepine, St. John's Wort.

6.3.1.2 Medications Known to be Sensitive CYP3A4 and CYP2B6 Substrates

In vitro studies indicated that repotrectinib does not inhibit the activity of CYP3A4 and CYP2B6; however, *in vitro* hepatocyte induction studies suggest that repotrectinib may induce CYP3A4 and CYP2B6. Clinical PK data also suggest that repotrectinib may induce CYP3A4 activity. Please refer to the Investigator's Brochure for detailed description.

- Induction of CYP3A4 or CYP2B6 isoenzymes may decrease exposures of co-administered drugs that are predominantly metabolized by CYP3A4 or CYP2B6 isoenzymes leading to a potential decrease of efficacy of co-administered drugs. The use of known sensitive CYP3A4 or CYP2B6 substrates are to be avoided and only used if deemed in the subjects 'best interest', by the treating Investigator during study treatment.
  - Sensitive CYP3A substrates: alfentanil, avanafil, budesonide, buspirone, conivaptan, darifenacin, darunavir, dasatinib, dronedarone, ebastine, eletriptan, eplerenone, everolimus, felodipine, ibrutinib, indinavir, lomitapide, lovastatin, lurasidone, maraviroc, midazolam, naloxegol, nisoldipine, quetiapine, saquinavir, simvastatin, sildenafil, sirolimus, tacrolimus, ticagrelor, tipranavir, tolvaptan, triazolam, vardenafil.
  - Sensitive CYP2B6 substrates: bupropion, efavirenz.

Repotrectinib is a moderate inducer of CYP3A4 and may accelerate the metabolism of both the estrogenic and progestagenic components of hormonal contraceptives, resulting in decreased effectiveness. If WOCBP use hormonal contraceptives (including oral hormonal contraceptives), they must use either another form of non-hormonal highly effective contraceptives or a reliable barrier method.

Additionally, refer to APPENDIX 7, Section 7.2 for Concomitant Medications specific for subjects participating in the midazolam DDI sub-study.

6.3.2 Medications Known to Prolong the QT Interval

Repotrectinib had no clinically significant effects on QTc based on the results of the concentration-QTc analysis. The following drugs are associated with QT prolongation and are prohibited:

- **Antiarrhythmics:** Quinidine, Disopyramide, Procainamide, Sotalol, Amiodarone, Dofetilide, Ibutilide, and Flecainide.
- **Antimicrobials:** Moxifloxacin, Sparfloxacin, Erythromycin, Azithromycin, Clarithromycin, and Pentamidine.
- **Antipsychotics:** Haloperidol, Droperidol, Quetiapine, Thioridazine, Chlorpromazine, Mesoridazine, and Pimozide.
- **Antihistamines:** Terfenadine and Astemizole.
- Antidepressant: Citalopram.
- Antimalarial: Halofantrine
- Antilipemic: Probucol
- Antianginal: Bepridil
- **Antiemetics:** Domperidone and Chlorpromazine
- Anti-cancer: Vandetanib
- **Opioid agonist:** Levomethadyl acetate

Additionally, refer to APPENDIX 6 for common medications known to have QT prolongation. For further reference, please use www.crediblemeds.org.

6.3.3 Other Anti-tumor or Investigational Drugs

No additional systemic anti-tumor therapy will be permitted while subjects are receiving study therapy. Additionally, the concurrent use of select herbal supplements is not permitted.

Bisphosphonate therapy for metastatic bone disease is permitted. Bisphosphonate therapy should be given as per local medical practice.

6.3.4 Seizure Prophylaxis

Moderate inducers of CYP3A, such as dexamethasone or other glucocorticoids, may be used at the discretion of the Investigator.

Seizure prophylaxis with non–enzyme-inducing anti-epileptic drugs (non-EIAEDs) is allowed during the study for subjects with controlled asymptomatic CNS involvement. EIAEDs are not allowed. Table 15 lists EIAEDs and non-EIAEDs.

Table 15: List of Enzyme-inducing Anti-epileptic Drugs and Non-enzyme-inducing Anti-epileptic Drugs

EIAEDs	non-EIAEDs
Carbamazepine<br>Oxcarbazepine<br>Phenytoin<br>Fosphenytoin<br>Phenobarbital<br>Primidone	Levetiracetam<br>Valproic acid<br>Lacosamide<br>Gabapentin<br>Topiramate<br>Lamotrigine<br>Tiagabine<br>Zonisamide<br>Clonazepam<br>Clonozam<br>Pregabalin

Abbreviation: EIAED, enzyme-inducing anti-epileptic drug

6.3.5 Hematopoietic Growth Factors

Primary prophylactic use of granulocyte-colony stimulating factors is not permitted during the first cycle in Phase 1 but they may be used to treat treatment-emergent neutropenia or anemia in Phase 2 as indicated by the current American Society of Clinical Oncology (ASCO) guidelines. Patients who have been treated for >4 weeks with erythropoietin prior to starting on study may continue on their existing treatment.

Erythropoietin may be used at the Investigator’s discretion for the supportive treatment of anemia.

Red blood cell and platelet transfusions should be administered as warranted.

6.3.6 Anti-diarrheal and Anti-emetic Therapy

Primary prophylaxis of diarrhea, nausea, and vomiting is not permitted in the first cycle in Phase 1, but is permitted in subsequent cycles in Phase 1. Primary prophylaxis in Phase 2 is at the Investigator's discretion using institutional guidelines and/or published guidelines. The choice of the prophylactic drug is up to the Investigator with the Sponsor Medical Monitor approval and assuming there is no known or expected drug-drug interaction. If so it must be approved by the Sponsor Medical Monitor.

6.3.7 Anti-inflammatory Therapy

Anti-inflammatory or narcotic analgesic may be offered as needed assuming there is no known or expected drug-drug interaction.

6.3.8 Cutaneous Toxicity Therapy

Prompt medical intervention is recommended at the first sign of appearance of cutaneous toxicity including topical or oral corticosteroids if required according to Investigator's judgment, assuming there is no known or expected drug-drug interaction.

6.3.9 Testosterone Replacement

Testosterone replacement therapy is only allowed in the presence of signs and symptoms clearly attributable to hypogonadism in consultation with an endocrinologist, who should also exclude any potential confounding effects of elevated prolactin and/or estradiol, or a significant recent change in corticosteroid dose, before doing so.

6.3.10 Dizziness Prophylaxis

Anti-dizziness medication (such as meclizine or other antihistamines) is permitted in Phase 2 at the Investigator's discretion. The choice of the prophylactic drug is up to Investigator discretion. Please consult the Sponsor for potential drug-drug interaction concern.

6.4 Other Study Restrictions

6.4.1 Surgery

Caution is advised on theoretical grounds for any surgical procedures during the study. The appropriate interval of time passed between surgery and repotrectinib required to minimize the risk of impaired wound healing and bleeding has not been determined. Stopping repotrectinib is recommended for at least 2 days prior to surgery. Postoperatively, the decision to reinitiate repotrectinib treatment should be based on a clinical assessment of satisfactory wound healing and recovery from surgery.

6.4.2 Palliative Radiation

Palliative radiotherapy on study is permitted for the treatment of painful bony lesions providing the lesions were known at the time of study entry and the Investigator clearly indicates that the need for palliative radiotherapy is not indicative of disease progression. In view of the current lack of data about the interaction of repotrectinib with radiotherapy, repotrectinib treatment should be interrupted during palliative radiotherapy, stopping 1 day before palliative radiotherapy and

resuming treatment 1 day after completion of palliative radiotherapy and recovery from any acute radiation toxicities to baseline.

6.4.3 *Supportive Care*

Palliative and supportive care for disease-related symptoms may be administered at the Investigator's discretion and according to available ASCO guidelines.

6.4.4 *Lifestyle Guidelines: Contraception*

For more details on contraceptive guidelines, please see APPENDIX 1.

7 STUDY PROCEDURES

7.1 Clinical Assessments

The schedule of assessments representing the required testing procedures to be performed during the study is diagrammed in the Study Calendars (Section 8.5).

7.1.1 *Main Informed Consent*

Prior to conducting any study-related activities, written informed consent and any other authorizations must be signed and dated by the subject or subject's legal representative. Every effort should be made to ensure that the protocol-required tests and procedures are completed as described. However, it is anticipated that from time to time there may be circumstances outside of the control of the Investigator that may make it unfeasible to perform the test. In those cases, the Investigator should take all steps necessary to ensure the safety and wellbeing of the subject. When a protocol-required test cannot be performed, the Investigator will document the reason for this and any corrective and preventive actions that he/she has taken to ensure that normal processes are adhered to as soon as possible.

The Sponsor study team or their designee should be informed of these incidents in a timely fashion. Unless indicated otherwise, scheduled clinic visits should occur within ± 2 days of the specified dates; where this is not possible because of extenuating circumstances (eg, holidays), the visit should take place as close to this window as possible.

7.1.2 *Pre-screening Informed Consent (Phase 2 only)*

In accordance with local policies and institutional guidelines, subjects may provide consent for their tumor tissue to undergo tissue testing for molecular alterations required for enrollment for ROS1 and NTRK rearrangements on a separate pre-screening ICF. There is no time window associated with signing of the pre-screening ICF.

Once the subject signs the main study ICF, the subject can continue to undergo the remaining screening procedures. The 28-day screening window begins when the subject signs the main study ICF (ie, signature on the pre-screening ICF does not initiate the 28 days screening window).

7.1.3 *Demographics*

Demographic information (eg, date or year of birth and race) will be recorded at screening, as allowed per local country privacy law regulations.

7.1.4 Medical History

Relevant medical history, including history of current disease, other pertinent clinical conditions, and information regarding underlying diseases will be recorded at screening.

7.1.5 Physical Examination

A complete physical examination including neurological examination will be performed by either the Investigator or a sub-Investigator according to the Study Calendar. New abnormal physical examination findings must be documented and followed by the Investigator, sub-Investigator, or other qualified staff at the next scheduled visit or earlier as clinically indicated.

7.1.5.1 Neurological Examination

The neurological examination needs to be performed at Screening, Cycle 1 Day 1, and at all subsequent clinical visits when a physical examination is required, including the End of Treatment visit. The neurological examination will include assessments of the following symptoms: dizziness, dysgeusia, paresthesia, ataxia, headache, neuralgia, extrapyramidal disorder, memory impairment, concentration impairment, somnolence, and cognitive disturbance.

7.1.6 Vital Signs

Body temperature, blood pressure, heart rate, respiratory rate, and pain level (0-10) will be performed at Screening and at every clinic visit. Blood pressure and heart rate can be assessed either in the supine or seated position. Height will be recorded at screening only. Body weight will be recorded at every clinic visit.

7.1.7 Performance Status

Performance scales will be assessed at Screening and at every clinic visit. The scale used will depend on the age of the subjects, including Lansky (< 16 years), Karnofsky (≥ 16 to <18 years), and ECOG (≥18 years).

For country-specific requirements, please refer to APPENDIX 8.

7.1.8 Laboratory Assessments

In Phase 2, blood chemistry, hematology, coagulation parameters, and urine analysis will all be analyzed by the central laboratory, please see Study Manual.

Central laboratory assessments are listed below:

- Blood chemistry
- Hematology
- Coagulation
- Urine analysis

Central laboratory testing in Phase 2 is mandatory and must be performed. If both local and central laboratory results are available at screening, eligibility will be based on central laboratory assessments. However, if central laboratory results cannot be obtained, then local laboratory

results may be used to determine subject eligibility prior to enrollment. Note: even if local laboratory results are utilized for eligibility purposes, central laboratory results testing remains mandatory; samples must still be sent to the central laboratory.

The Investigator must evaluate all results outside the reference range and determine the clinical significance (clinically significant or not clinically significant). All results will be entered into the CRF.

Pregnancy test: Women of childbearing potential will have a serum pregnancy test performed at Screening, Day 1 of every cycle, at the End of Treatment, and as clinically indicated.

7.1.9 Adverse Events

Assessment of AEs will include type, incidence, severity (graded by the CTCAE, v4.03), timing, seriousness, and relatedness. Adverse events will be assessed at every clinic visit.

7.1.10 Concomitant Medications and Treatments

All concomitant medication and concurrent treatments (eg, radiation therapy) will be documented at Screening and at every clinic visit. The indication for administration and dates of medication or treatment will be captured.

7.1.11 Subject Dosing Diary

All subjects are required to complete dosing diary daily in conjunction with administration of repotrectinib. At C1D1 all subjects should be trained by the site personnel how to complete the diary and inform them that all efforts should be made to record the dosing information as accurate as possible. The diary needs to be returned to the clinic together with the remaining study drug at each study visit. Study drug compliance and accountability needs to be reviewed at each study visit by the site personnel.

7.1.12 Ophthalmologic Examination

The ophthalmologic examination needs to be performed at Cycle 1 Day 1 (C1D1) with a window of -7 days (baseline), Cycle 3 Day 1 (C3D1, ±2 days), annually thereafter while on treatment (±7 days, at the end of treatment (±7 days) and as clinical indicated. The following ophthalmologic tests are mandatory at each scheduled time point including 1) best-corrected visual acuity (BCVA), 2) refractive error associated with BCVA, 3) pupil size (pupillary diameter), 4) slit-lamp biomicroscopy of the anterior segment, 5) intraocular inflammation, 6) intraocular pressure, and 7) fundoscopy of the posterior segment. If a participant exhibits ocular or visual symptoms, other ophthalmologic evaluation will be considered while evaluating and treating the participant as clinically indicated according to local clinical practice guidelines. Visual disorder should be followed for up to 12 months if the symptoms persist or worsen in severity.

7.2 Tumor Assessments

Tumor assessments will be performed according to RECIST Version 1.1 guidelines (Eisenhauer 2009).

For Phase 1, tumor assessments will be performed at Screening, at the end of Cycle 2 (± 7 days),every 2 cycles (± 7 days) up to the end of Cycle 18 and then every 3 cycles (± 7 days) up to

the end Cycle 36 and then every 4 cycles (± 7 days) thereafter until documented progression of disease regardless of treatment delays resulting from toxicity, and at the EOT if more than 4 weeks have passed since the last imaging assessment.

For Phase 2, tumor assessments will be performed at Screening, C3D1 (± 7 days), every 2 cycles (± 7 days) up to Cycle 19 and then every 3 cycles (± 7 days) up to Cycle 37 and then every 4 cycles (± 7 days) thereafter until documented progression of disease regardless of treatment delays resulting from toxicity, and at the EOT if more than 4 weeks have passed since the last imaging assessment. If a CT scan of the chest, abdomen, pelvis has been performed within 30 days prior to Cycle 1 Day 1 the scan can be used for Screening.

Imaging studies will include a computerized tomography (CT) or magnetic resonance imaging (MRI) scan of the chest, abdomen, pelvis [CAP] (depending on tumor type), plus bone scan (if applicable) and gadolinium contrast-enhanced brain MRI.

- At Screening, a gadolinium contrast-enhanced MRI of the brain should be obtained to rule out newly diagnosed, untreated brain metastases or to document stability of previously treated brain metastases.
  - For the Phase 1 portion of the study, only subjects with documented baseline CNS metastases will be assessed by MRI of the brain at every 2 cycles for the first assessment and then every 2 cycles up to the end of Cycle 18 and then every 3 cycles up to the end of Cycle 36 and then every 4 cycles thereafter until documented progression of disease.
  - For the Phase 2 portion of the study, MRI brain scans should be performed *for all subjects* at every on study tumor assessment: at Screening, at C3D1(± 7 days), every 2 cycles (± 7 days) up to Cycle 19 and then every 3 cycles (± 7 days) up to Cycle 37 and then every 4 cycles (± 7 days) thereafter until documented progression of disease regardless of treatment delays resulting from toxicity, and at the EOT if more than 4 weeks have passed since the last imaging assessment. If an MRI of the brain has been performed within 30 days prior to Cycle 1 Day 1 the MRI can be used for Screening.
  - For subjects diagnosed with primary brain tumors, only the MRI of the brain will be required at Screening and during the study.

At Screening, a bone scan should be obtained if bone metastases are suspected. For subjects with bone metastases at baseline, a bone scan should only be repeated to check for residual disease when assessing for complete response unless the bone scan is used to determine RECIST Version 1.1 response assessment. The bone scan should be performed at the same time as the chest, abdomen and pelvis CT (CAP) scans. If a bone scan has been performed within 30 days prior to Cycle 1 Day 1 the scan can be used for Screening. Radiographic confirmation of objective tumor response or disease progression will be based on RECIST Version 1.1 and assessed by blinded independent central review (BICR). The same imaging modality should be used at Screening and on study.

For Phase 1 and Phase 2, assessment of overall response will be made using RECIST Version 1.1 criteria. A maximum of 5 target lesions will be selected for tumor assessment (whole body

including brain) using ≥ 10 mm lesion size. Modified RECIST Version 1.1 will be used for intracranial response assessment in the Phase 2 portion of the study.

Tumor assessments will include all known or suspected disease sites. CT scans of Chest Abdomen Pelvis [CAP] and MRI of the brain will be performed at screening and post-screening timepoints. Gadolinium contrast-enhanced MRI must be used to assess CNS lesions. When clinically contraindicated to conduct brain MRI, CT of brain with contrast may be applied to acquire brain images.

For intracranial response assessment in subjects with brain metastases at baseline, up to 5 CNS target lesions can be selected within the brain, and a CNS lesion selected as target lesion will have a diameter of minimum 5 mm using contrast enhancement. Same rules as for RECIST Version 1.1 will be applied to the brain to define CNS responses and progression based on assessment of CNS target lesions, non-target lesions, and new lesions.

IV contrast is required when not medically contraindicated. Subjects who have a contraindication to IV contrast may have MRI exams of the brain, abdomen, and pelvis performed in lieu of CTs and a non-contrast CT of the chest. Positron emission tomography (PET)/CT may be used to document baseline and new disease, but the CT portion of a PET/CT may not be used in lieu of a diagnostic CT, unless it is performed with IV contrast.

All efforts should be made to obtain the EOT radiographic scan before treatment discontinuation, and where possible inform the Sponsor's Medical Monitor when considering treatment discontinuation. After treatment discontinuation, tumor assessments should continue at the current scan interval at the time of treatment discontinuation until a subject begins a new course of cancer therapy or withdraws consent if there was no BICR-confirmed radiologic progression at the time of treatment discontinuation.

Care must be taken in scheduling disease assessments to prevent introduction of bias based on treatment delays.

Additional requirements are to be followed that are provided in the Imaging Manual and Image Acquisition Guideline (IAG).

7.2.1 Blinded Independent Central Review (BICR)

All scans will be submitted to a third-party central imaging laboratory for independent review of tumor response and disease progression during the study according to an Imaging Review Charter to be prepared by the core imaging laboratory in consultation with the Sponsor Medical Monitor.

It is important to the integrity of the study that all imaging studies are forwarded to the central imaging laboratory in a timely manner throughout the study, within 48 hours of collection whenever possible. Further details can be found in the Imaging Manual.

7.3 Tumor Procurement

7.3.1 Archival Tumor Tissue

Tumor samples are mandatory for all subjects enrolled in the study. Adequate tumor tissue needs to be sent to the Sponsors' designated central diagnostic laboratory for prospective confirmation

of *ROS1* or *NTRK* gene fusion status **PRIOR to enrollment onto Phase 2** if a FISH test was used for local testing. If an NGS or qPCR test was used for local testing, adequate tumor tissue needs to be sent to the Sponsors' designated central diagnostic laboratory for retrospective confirmation of the fusion status. In cases where archived tumor tissue is not available, a fresh biopsy should be obtained for this purpose.

Archived tumor tissue needs to be from an incisional or excisional biopsy, or a core needle biopsy, of a primary or metastatic lesion. Pleural effusion cell pellets, fine needle aspiration biopsies, other cytology samples, and bone biopsy are not acceptable.

FFPE tissue block[s] that contain sufficient tissue to generate at least 10 (preferably 15) unbaked, 4-5 µm thick unstained slides are required. If no FFPE block is available, then at least 10 (preferably 15) unbaked, 4-5 µm thick unstained slides with a minimum of 10% tumor content and 1 Hematoxylin and eosin stain (H&E) slide must be provided.

Details for handling of these samples including processing, storage, and shipment will be provided in the Laboratory Manual.

Additionally, tumor tissue samples from enrolled patients will be used for assessment of biomarkers potentially associated with sensitivity and/or resistance to repotrectinib (mutations in *ROS1, NTRK1, NTRK2,* or *NTRK3*, mutations/copy number variation of various genes, expression and/or phosphorylation of various proteins, etc.).

7.3.2 Fresh Tumor Biopsy

Tumor samples are mandatory for all subjects enrolled in the study. Adequate tumor tissue needs to be sent to the Sponsors' designated central diagnostic laboratory **PRIOR to enrollment onto Phase 2** if a FISH test was used for local testing. If an NGS or qPCR test was used for local testing, adequate tumor tissue needs to be sent to the Sponsors' designated central diagnostic laboratory for retrospective confirmation of the fusion status. In cases where archived tumor tissue is not available, a fresh biopsy should be obtained for this purpose. The fresh biopsy will consist of an incisional or excisional biopsy, or a core needle biopsy, of a primary or metastatic lesion and should obtain enough amount of tumor to generate at least 10 (preferably 15) unbaked, 4-5 µm thick unstained slides containing FFPE tumor tissue. Pleural effusion cell pellets, fine needle aspiration biopsies, other cytology based biopsies, and bone biopsy are not acceptable.

Details for handling of these specimens including processing, storage, and shipment will be provided in the Laboratory Manual.

7.3.3 Liquid Biopsies

In all subjects, blood will be collected during screening prior to the first dose of repotrectinib, at the Cycle 3 Day 1 visit (in conjunction with tumor assessment evaluation), and either at the time of tumor progression or EOT (whichever occurs earlier). Circulating cell -free DNA (ccfDNA) will be isolated from blood to identify genomic alterations in oncogenes and tumor suppressor genes that may predict activity of repotrectinib and to gain insights into potential mechanisms of resistance using next -generation DNA and RNA sequencing and related techniques. Furthermore, minor allele frequency (MAF) modulations of genomic alterations and their association with

response will be studied. Details for handling of these specimens including processing, storage, and shipment will be provided in the Study Manual.

7.4 Clinical Laboratory Assessments

APPENDIX 2 and APPENDIX 3 list all of the specific laboratory tests that should be performed for the Phase 1 and Phase 2 portions of the study. Table 23, Table 24, Table 25, and Table 26 are Study Calendars that list all timing of the laboratory tests to be performed.

Investigators can also perform additional local laboratory tests for the purpose of planning treatment administration, dose modification, or following AEs.

7.5 Electrocardiogram (ECG)

Triplicate 12-lead (with a 10-second rhythm strip) tracing will be used for all ECGs. It is preferable that the machine used has a capacity to calculate the standard intervals automatically. At each time point (see Study Calendars, Section 8.5), 3 consecutive ECGs will be performed at approximately 2 minutes apart to determine the mean QTc interval. If the mean QTc is prolonged (> 500 msec [ie, CTCAE grade 3]), then the ECGs should be re-evaluated by a qualified person at the site for confirmation as soon as the finding is made, including verification that the machine reading is accurate. If manual reading verifies a QTc of > 500 msec, immediate correction for reversible causes (including electrolyte abnormalities, hypoxia, and concomitant medications for drugs with the potential to prolong the QTc interval) should be performed. Study drug will be held until the QTc interval decreases to ≤ 500 msec. Subjects will then re-start the study drug at the next lower dose level. If the QTc interval has still not decreased to < 500 msec after 2 weeks, or if at any time a subject has a QTc interval > 515 msec or becomes symptomatic, the subject will be removed from the study. Additional triplicate ECGs may be performed as clinically indicated.

Prior to concluding that an episode of prolongation of the QTc interval is due to study drug, thorough consideration should be given to potential precipitating factors (eg, change in subject clinical condition, effect of concurrent medication, electrolyte disturbance) and possible evaluation by specialist. If subject experiences a cardiac or neurologic AE (specifically syncope, dizziness, seizures, or stroke), an ECG (triplicate) should be obtained at the time of the event.

When matched with PK sampling, the ECG must be carried out before each PK sample drawing such that the PK sample is collected at the nominal time (ie, the timing of the PK collections overrides the timing of the ECG collections).

7.6 Echocardiogram/MUGA Scan

In order to monitor potential left ventricular ejection fraction dysfunction, an echocardiogram or multigated acquisition (MUGA) scan will be performed at the screening period, and starting with Cycle 4, repeated every 3 cycles thereafter (eg, Cycles 7, 10, 13 and so on), and at the EOT. The same method of evaluation should be utilized throughout the study.

7.7 Pharmacokinetic (PK) Assessments

Blood samples for PK analysis of repotrectinib will be collected during the study from subjects receiving study medication. Table 16 lists noncompartmental PK parameters that will be

calculated from the individual plasma concentration-versus-time profiles using Phoenix® WinNonlin® 7.0 (Certara, Princeton, NJ). The individual and mean plasma concentration-versus-time profiles will be displayed graphically.

For the six subjects participating in the midazolam sub-study, blood samples for PK analysis of MDZ and 1-OH-MDZ will be collected serially for 24 hours on Cycle 1 Day -2 and Cycle 1 Day 22.

Blood sampling schedules for PK assessment can be found in Table 17, Table 18, Table 19, Table 20, Table 21, and in Study Calendars (Table 23, Table 24, Table 25, and Table 26) for dose escalation and dose expansion, respectively.

Table 16: Noncompartmental Pharmacokinetic Parameters

**AUC_{last}**	The area under the curve (AUC) from time zero to the last quantifiable concentration point (t_{last})
**AUC_{inf}**	The AUC from time zero to infinity
**AUC_{tau}**	The AUC calculated to the end of the dosing interval, tau
**AUC%Extrap**	The percent extrapolated area under the curve to infinity. This is calculated as ratio of the extrapolated area (C_{last}/λ_z) to the total AUC_{inf}
**C_{trough}**	Observed concentration at the end of a dosing interval (taken directly before next administration)
**C_{max}**	The maximum observed plasma, blood, serum, or other body fluid drug concentration
**T_{max}**	The time to reach maximum (peak) plasma, blood, serum, or other body fluid drug concentration
**λ_z**	Smallest (slowest) disposition (hybrid) rate constant (time-1) may also be used for terminal elimination rate constant
**$t_{½}$**	The elimination half-life associated with the terminal slope (λ_z) of a semi logarithmic concentration-time curve (time)
**CL/F**	Apparent total body clearance of drug from the plasma
**V_z/F**	The apparent volume of distribution during terminal phase (associated with λ_z)
**R_{acc}**	Accumulation ratio calculated using AUC_{tau} values obtained from a dosing interval at steady-state divided by AUC_{tau} at Day 1 or PK lead-in phase

If the clinical safety, PK, and other data become available and indicate that an alternative dosing schedule is deemed necessary, a formal protocol amendment with rationale to support the change in dosing schedule will be submitted to the appropriate health authorities for review and no changes will be implemented until the protocol amendment is approved by the appropriate IRBs.

The collection of plasma with exact dates and clock times of drug administration and sample collection will be recorded on the appropriate CRF.

All blood samples will be taken by either direct venipuncture or venous catheter or indwelling cannula inserted in a forearm vein. Samples should be processed and labeled as detailed in the Laboratory Manual. Plasma fractions will be split in 2 aliquots and all samples should be stored frozen at~ -70°C within 90 minutes of venipuncture at the site until sample shipment.

7.7.1 PK Collection Times Points

PK blood samples will be collected in Phase 1a, 1b, 1c, midazolam DDI sub-study and Phase 2. All reasonable efforts will be made to obtain the PK samples at the exact nominal time relative to dosing. However, samples obtained within 10% of the nominal time (eg, within 6 minutes of the 60-minute sample) from dosing will not be considered as a protocol deviation, as long as the exact time of the sample collection is noted on the source document and data collection tool (eg, CRF). For pre-dose samples, including the lead-in dose 72 hr. sample (considered as the pre-dose samples on Cycle 0 Day -3) in Phase 1a within Cohorts 1-6 (Table 17), these blood samples should be collected within 1 hour prior to dose administration.

7.7.1.1 Blood for PK Analysis of Repotrectinib (Phase 1a Dose Escalation)

For the determination of repotrectinib during the Phase 1a Dose Escalation portion, 5.0 mL of whole blood will be drawn for each time point. For subjects treated in the escalation phase 1a for both QD and BID dosing regimens (Table 17), 2 full PK profiles will be collected during the PK lead-in period (Cycle 0 Day -3) and Cycle 1 Day 15 and abbreviated PK profiles on Day 1 of Cycles 2-4. There is an additional pre-dose PK draw on Cycle 1 Day 8. A total of approximately 120 mL of blood will be collected for each subject during the Phase 1a Dose Escalation phase.

Table 17: Time Points of Blood Collection for Repotrectinib PK Assessment during Phase 1a Dose Escalation Phase

PK Collection Number	Sample Number	Cycle	Day	Scheduled Time Points Relative to Previous Dose (hours)	Description
1	1	PK lead-in	-3	0 hr[a]	Pre-dose
1	2	PK lead-in	-3	1 hr	Post-dose
1	3	PK lead-in	-3	2 hr	Post-dose
1	4	PK lead-in	-3	4 hr	Post-dose
1	5	PK lead-in	-3	6 hr	Post-dose
1	6	PK lead-in	-3	8 hr	Post-dose
1	7	PK lead-in	-2	24 hr	Post-dose
1	8	PK lead-in	-1	48 hr	Post-dose
2	9	1	1	72 hr	72 hr post-dose of PK lead-in and Cycle 1 Day 1 pre-dose
2	10	1	1	4 hr	Post-dose
3	11	1	8	0 hr[a]	Pre-dose
4	12	1	15	0 hr[a]	Pre-dose

Table 17: Time Points of Blood Collection for Repotrectinib PK Assessment during Phase 1a Dose Escalation Phase

PK Collection Number	Sample Number	Cycle	Day	Scheduled Time Points Relative to Previous Dose (hours)	Description
4	13	1	15	1 hr	Post-dose
4	14	1	15	2 hr	Post-dose
4	15	1	15	4 hr	Post-dose
4	16	1	15	6 hr	Post-dose
4	17	1	15	8 hr	Post-dose
4	18	1	16	24 hr	Pre-dose
5	19	2	1	0 hr[a]	Pre-dose
5	20	2	1	4 hr	Post-dose
6	21	3	1	0 hr[a]	Pre-dose
6	22	3	1	4 hr	Post-dose
7	23	4	1	0 hr[a]	Pre-dose
7	24	4	1	4 hr	Post-dose
	1001+ [b]	NA	NA	Unscheduled[b]	Unspecified

Abbreviations: BID, twice daily; CRF, case report form; NA, not applicable; PK, pharmacokinetic
All measurement times are relative to dose of repotrectinib unless otherwise specified. For BID dose, the times are relative to the first dose of repotrectinib in the day. Samples obtained within 10% of the nominal time (eg, within 6 minutes of the 60-minute sample) from dosing will not be considered as a protocol deviation, as long as the exact time of the sample collection is noted on the source document and data collection tool (eg, CRF).

[a] Take PK sample immediately prior to the administration of repotrectinib

[b] Sample numbers for any unscheduled blood collection for repotrectinib will start with 1001

7.7.1.2 Blood for PK Analysis of Repotrectinib (Phase 1b Food Effect)

The Phase 1b Food Effect was performed according to Section 3.3. Each subject served as his/her own control.

For the determination of repotrectinib during the Phase 1b Dose Escalation portion, 5.0 mL of whole blood was drawn for each time point. For subjects treated in the Food Effect phase, 2 full PK profiles will be collected during the PK lead-in period Day -7 and Cycle 1 Day 1 and abbreviated PK profiles on Cycle 1 Day 15 and on Day 1 of Cycles 2-4. There is an additional pre-dose PK draw on Cycle 1 Day 8. The third dose will be given at Cycle 1 Day 4 after blood

sampling at 72-hour time point. A total of approximately 135 mL blood will be collected for each subject during Phase 1b Dose Escalation phase (Table 18 and Table 19).

Table 18: Time Points of Blood Collection for Repotrectinib PK Assessment during Phase 1b Food Effect Study (FED Followed by FASTED)

PK Collection Number	Sample Number	Cycle	Day	Scheduled Time Points Relative to Previous Dose (hours)	Description
10	1	PK lead-in	-7	0 hr[a]	Pre-dose (Fed)
10	2	PK lead-in	-7	1 hr	Post-dose (Fed)
10	3	PK lead-in	-7	2 hr	Post-dose (Fed)
10	4	PK lead-in	-7	4 hr	Post-dose (Fed)
10	5	PK lead-in	-7	6 hr	Post-dose (Fed)
10	6	PK lead-in	-7	8 hr	Post-dose (Fed)
20	7	PK lead-in	-6	24 hr	Post-dose (Fed)
30	8	PK lead-in	-5	48 hr	Post-dose (Fed)
40	9	PK lead-in	-4	72 hr	Post-dose (Fed)
50	10	1	1	0 hr[a]	Pre-dose (Fasted)
50	11	1	1	1 hr	Post-dose (Fasted)
50	12	1	1	2 hr	Post-dose (Fasted)
50	13	1	1	4 hr	Post-dose (Fasted)
50	14	1	1	6 hr	Post-dose (Fasted)
50	15	1	1	8 hr	Post-dose (Fasted)
60	16	1	2	24 hr	Cycle 1 Day 1 post-dose (Fasted)
70	17	1	3	48 hr	Cycle 1 Day 1 post-dose (Fasted)
80	18	1	4	72 hr	Cycle 1 Day 1 post-dose (Fasted)
90	19	1	8	0 hr[a]	Pre-dose
100	20	1	15	0 hr[a]	Pre-dose
100	21	1	15	4 hr	Post-dose
110	22	2	1	0 hr[a]	Pre-dose
110	23	2	1	4 hr	Post-dose

Table 18: Time Points of Blood Collection for Repotrectinib PK Assessment during Phase 1b Food Effect Study (FED Followed by FASTED)

PK Collection Number	Sample Number	Cycle	Day	Scheduled Time Points Relative to Previous Dose (hours)	Description
120	24	3	1	0 hr[a]	Pre-dose
120	25	3	1	4 hr	Post-dose
130	26	4	1	0 hr[a]	Pre-dose
130	27	4	1	4 hr	Post-dose
	1001+ [b]	NA	NA	Unscheduled[b]	Unspecified

Abbreviations: BID, twice daily; CRF, case report form; NA, not applicable; PK, pharmacokinetic

All measurement times are relative to dose of repotrectinib unless otherwise specified. Samples obtained within 10% of the nominal time (eg, within 6 minutes of the 60-minute sample) from dosing will not be considered as a protocol deviation, as long as the exact time of the sample collection is noted on the source document and data collection tool (eg, CRF).

[a] Take PK sample immediately prior to the administration of repotrectinib

[b] Sample numbers for any unscheduled blood collection for repotrectinib will start with 1001

Table 19: Time Points of Blood Collection for Repotrectinib PK Assessment during Phase 1b Food Effect Study (FASTED Followed by FED)

PK Collection Number	Sample Number	Cycle	Day	Scheduled Time Points Relative to Previous Dose (hours)	Description
10	1	PK lead-in	-7	0 hr[a]	Pre-dose (Fasted)
10	2	PK lead-in	-7	1 hr	Post-dose (Fasted)
10	3	PK lead-in	-7	2 hr	Post-dose (Fasted)
10	4	PK lead-in	-7	4 hr	Post-dose (Fasted)
10	5	PK lead-in	-7	6 hr	Post-dose (Fasted)
10	6	PK lead-in	-7	8 hr	Post-dose (Fasted)
20	7	PK lead-in	-6	24 hr	Post-dose (Fasted)
30	8	PK lead-in	-5	48 hr	Post-dose (Fasted)
40	9	PK lead-in	-4	72 hr	Post-dose (Fasted)

Table 19: Time Points of Blood Collection for Repotrectinib PK Assessment during Phase 1b Food Effect Study (FASTED Followed by FED)

PK Collection Number	Sample Number	Cycle	Day	Scheduled Time Points Relative to Previous Dose (hours)	Description
50	10	1	1	0 hr[a]	Pre-dose (Fed)
50	11	1	1	1 hr	Post-dose (Fed)
50	12	1	1	2 hr	Post-dose (Fed)
50	13	1	1	4 hr	Post-dose (Fed)
50	14	1	1	6 hr	Post-dose (Fed)
50	15	1	1	8 hr	Post-dose (Fed)
60	16	1	2	24 hr	Cycle 1 Day 1 post-dose (Fed)
70	17	1	3	48 hr	Cycle 1 Day 1 post-dose (Fed)
80	18	1	4	72 hr	Cycle 1 Day 1 post-dose (Fed)
90	19	1	8	0 hr[a]	Pre-dose
100	20	1	15	0 hr[a]	Pre-dose
100	21	1	15	4 hr	Post-dose
110	22	2	1	0 hr[a]	Pre-dose
110	23	2	1	4 hr	Post-dose
120	24	3	1	0 hr[a]	Pre-dose
120	25	3	1	4 hr	Post-dose
130	26	4	1	0 hr[a]	Pre-dose
130	27	4	1	4 hr	Post-dose
	1001+ [b]	NA	NA	Unscheduled[b]	Unspecified

Abbreviations: BID, twice daily; CRF, case report form; NA, not applicable; PK, pharmacokinetic

All measurement times are relative to dose of repotrectinib unless otherwise specified. Samples obtained within 10% of the nominal time (eg, within 6 minutes of the 60-minute sample) from dosing will not be considered as a protocol deviation, as long as the exact time of the sample collection is noted on the source document and data collection tool (eg, CRF).

[a] Take PK sample immediately prior to the administration of repotrectinib

[b] Sample numbers for any unscheduled blood collection for repotrectinib will start with 1001

7.7.1.3 Blood for PK Analysis of Repotrectinib (Phase 1c Dose Escalation)

For the determination of repotrectinib during the Phase 1c Dose Escalation portion, 5.0 mL of whole blood will be drawn for each time point. For subjects treated in the escalation Phase 1c, 2 full PK profiles will be collected during Cycle 1 Day 1 and Cycle 1 Day 15 and abbreviated PK profiles on Day 1 of Cycles 2-4. There is an additional pre-dose PK draw on Cycle 1 Day 8. A total of approximately 105 mL of blood will be collected for each subject during the Phase 1c Dose Escalation phase (Table 20).

Table 20: Time Points of Blood Collection for Repotrectinib PK Assessment during Phase 1c Dose Escalation Phase

PK Collection Number	Sample Number	Cycle	Day	Scheduled Time Points Relative to Previous Dose (hours)	Description
1	1	1	1	0 hr[a]	Pre-dose
1	2	1	1	1 hr	Post-dose
1	3	1	1	2 hr	Post-dose
1	4	1	1	4 hr	Post-dose
1	5	1	1	6 hr	Post-dose
1	6	1	1	8 hr	Post-dose
2	7	1	2	24 hr	Post-dose of Cycle 1 Day 1 Dose
2	8	1	8	0 hr[a]	Pre-dose
3	9	1	15	0 hr[a]	Pre-dose
3	10	1	15	1 hr	Post-dose
3	11	1	15	2 hr	Post-dose
3	12	1	15	4 hr	Post-dose
3	13	1	15	6 hr	Post-dose
3	14	1	15	8 hr	Post-dose
3	15	1	16	24 hr	Post-dose of Cycle 1 Day 15 dose
4	16	2	1	0 hr[a]	Pre-dose
4	17	2	1	4 hr	Post-dose
5	18	3	1	0 hr[a]	Pre-dose

Table 20: Time Points of Blood Collection for Repotrectinib PK Assessment during Phase 1c Dose Escalation Phase

PK Collection Number	Sample Number	Cycle	Day	Scheduled Time Points Relative to Previous Dose (hours)	Description
5	19	3	1	4 hr	Post-dose
6	20	4	1	0 hr[a]	Pre-dose
6	21	4	1	4 hr	Post-dose
	1001+ [b]	NA	NA	Unscheduled[b]	Unspecified

Abbreviations: BID, twice daily; CRF, case report form; NA, not applicable; PK, pharmacokinetic

All measurement times are relative to dose of repotrectinib unless otherwise specified. Samples obtained within 10% of the nominal time (eg, within 6 minutes of the 60-minute sample) from dosing will not be considered as a protocol deviation, as long as the exact time of the sample collection is noted on the source document and data collection tool (eg, CRF).

[a] Take PK sample immediately prior to the administration of repotrectinib

[b] Sample numbers for any unscheduled blood collection for repotrectinib will start with 1001

7.7.1.4 Blood for PK Analysis of Midazolam and Repotrectinib (Midazolam DDI Sub-study)

For the determination of midazolam PK during the midazolam interaction sub-study at the RP2D, 4.0 mL of whole blood will be drawn on Day -2 at the specified time points following the midazolam dose. Blood samples will be collected for the two full PK profiles of midazolam on Cycle 1 Day -2 and Cycle 1 Day 22. Repotrectinib full PK will also be assessed on Cycle 1 Day 1 and 22 and additional abbreviated PK samples will be collected on Cycle 1 Day 15 and Day 1 of Cycles 2, 3, and 4. The blood samples to be collected for the midazolam and repotrectinib PK profile are listed in Appendix 6.

7.7.1.5 Blood for PK Analysis of Repotrectinib (Phase 2)

For adult subjects (age 18 years and older) enrolled in the Phase 2 Study, 5 abbreviated PK profiles will be collected on Cycle 1 Day 1, Cycle 1 Day 15, and Cycles 2-4 Day 1 (Table 21). For adolescent subjects age 12-17 years enrolled in the Phase 2 Study, PK sample collections are specified in Table 22. At each time point, 4.0 mL of whole blood will be drawn. A total of approximately 40 mL blood will be collected for each adult subject and 56 mL for adolescent subjects enrolled in the dose expansion phase.

Table 21: Time Points of Blood Collection of Repotrectinib PK Assessment during Phase 2 (Adult Subjects age 18 Years and Older)

PK Collection Number	Sample Number	Cycle	Day	Scheduled Time Points Relative to Previous Dose (hours)	Description
101	201	1	1	0 hr[a]	Pre-dose
101	202	1	1	4 hr	Post-dose
102	203	1	15	0 hr[a]	Pre-dose
102	204	1	15	4 hr	Post-dose
103	205	2	1	0 hr[a]	Pre-dose
103	206	2	1	4 hr	Post-dose
104	207	3	1	0 hr[a]	Pre-dose
104	208	3	1	4 hr	Post-dose
105	209	4	1	0 hr[a]	Pre-dose
105	210	4	1	4 hr	Post-dose
	2001+[b]	NA	NA	Unscheduled[b]	Unspecified

Abbreviations: BID, twice daily; CRF, case report form; NA, not applicable; PK, pharmacokinetic

All measurement times are relative to dose of repotrectinib unless otherwise specified. Samples obtained within 10% of the nominal time (eg, within 6 minutes of the 60-minute sample) from dosing will not be considered as a protocol deviation, as long as the exact time of the sample collection is noted on the source document and data collection tool (eg, CRF).

[a] Take PK sample immediately prior to the administration of repotrectinib

[b] Sample numbers for any unscheduled blood collection for repotrectinib will start with 1001

Table 22: Time Points of Blood Collection of Repotrectinib PK Assessment during Phase 2 (Adolescent Subjects Age 12-17 Years)

PK Collection Number	Sample Number	Cycle	Day	Scheduled Time Points Relative to Previous Dose (hours)	Description
101	201	1	1	0 hr[a]	Pre-dose
101	202	1	1	1 hr	Post-dose
101	203	1	1	2 hr	Post-dose
101	204	1	1	4 hr	Post-dose
101	205	1	1	6 hr	Post-dose

Table 22: Time Points of Blood Collection of Repotrectinib PK Assessment during Phase 2 (Adolescent Subjects Age 12-17 Years)

PK Collection Number	Sample Number	Cycle	Day	Scheduled Time Points Relative to Previous Dose (hours)	Description
101	206	1	2	24 hr	Pre-dose
102	207	1	15	0 hr[a]	Pre-dose
102	208	1	15	4 hr	Post-dose
103	209	2	1	0 hr[a]	Pre-dose
103	210	2	1	4 hr	Post-dose
104	211	3	1	0 hr[a]	Pre-dose
104	212	3	1	4 hr	Post-dose
105	213	4	1	0 hr[a]	Pre-dose
105	214	4	1	4 hr	Post-dose
	2001+[b]	NA	NA	Unscheduled[b]	Unspecified

Abbreviations: BID, twice daily; CRF, case report form; NA, not applicable; PK, pharmacokinetic

All measurement times are relative to dose of repotrectinib unless otherwise specified. Samples obtained within 10% of the nominal time (eg, within 6 minutes of the 60-minute sample) from dosing will not be considered as a protocol deviation, as long as the exact time of the sample collection is noted on the source document and data collection tool (eg, CRF).

[a] Take PK sample immediately prior to the administration of repotrectinib

[b] Sample numbers for any unscheduled blood collection for repotrectinib will start with 1001

PK sampling schedule may be modified based on emerging PK data. In addition to samples collected at the scheduled times, an additional blood sample should be collected from subjects experiencing unexpected and/or serious AEs and the date and time documented in the CRF.

All efforts will be made to obtain the PK samples at the scheduled nominal time relative to dosing. However, samples obtained within 10% of the nominal time (eg, within 6 minutes of a 60-minute sample, within 12 minutes of a 120-minute samples) will be considered protocol compliant, and the exact time of the sample collection noted on the CRF. If a scheduled blood sample collection cannot be completed for any reason, the missed sample time may be re-scheduled with agreement of clinical Investigators, subject, and the Sponsor Medical Monitor.

PK samples will be assayed for repotrectinib using a validated analytical method in compliance with designated bioanalytical Contract Research Organization SOPs. Details regarding the collection, processing, storage, and shipping of the blood samples will be provided in the Study Manual.

7.8 Pharmacodynamic (PD) Assessment

7.8.1 On-treatment Tumor Biopsy

One optional fresh tumor tissue re-biopsy is recommended between Cycle 1 Day 8 and Cycle 1 Day 15 during the Phase 1b Food Effect. Specimens will be sent to the Sponsor-designated central laboratories for the PD study. Subjects enrolled in the Phase 1a Dose Escalation part of the study will not undergo this optional fresh tumor tissue re-biopsy.

In Phase 2, liquid biopsies will be collected during screening prior to the first dose of repotrectinib and at the Cycle 3 Day 1 visit to study the MAF modulation of genomic alterations and their association with early response or intrinsic resistance.

7.9 Patient-Reported Outcomes (PRO)

For NSCLC subjects enrolled into Phase 2, at screening, prior to the first dose of repotrectinib on Cycle 1 Day 1, pre-dose on Day 1 of each subsequent treatment cycle thereafter, and at the End of Treatment, subjects will be required to complete the following self-administered quality of life (QOL) instruments: the European Organization for Research and Treatment of Cancer (EORTC) Core Quality of Life Questionnaire (QLQ-C30) (APPENDIX 4) and EORTC-Lung Cancer Quality of Life Questionnaire (QLQ-LC13) (APPENDIX 5) to assess health status.

Non-NSCLC subjects will only be required to complete the EORTC-QLQ-C30 module (APPENDIX 4).

8 STUDY ASSESSMENTS BY VISIT

8.1 Phase 1a Dose Escalation

For country-specific requirements, please refer to APPENDIX 8.

8.1.1 Screening Phase 1a Dose Escalation (within 28 days of the first dose of repotrectinib)

- Obtain Informed Consent
- Eligibility checklist (see complete list in Section 5.1 and Section 5.3)
- Perform molecular testing as per Section 5.1 and Table 23
  - Molecular testing to determine eligibility can be performed in advance with no time limit (eg, the subject can be tested while being treated on another anticancer therapy)
  - Source documents (molecular pathology report detailing the specific test that detect the *ALK*, *ROS1*, or *NTRK* rearrangement) should be submitted for the Sponsor approval of eligibility
  - Submitted archival tissues to Sponsor's designated central laboratory to confirm the specific molecular alteration
  - An optional tumor tissue re-biopsy (for subjects who have failed prior TKIs) during the screening period to investigate resistance mechanism(s) is recommended but not mandatory
- Obtain detailed treatment history
  - Number of prior TKIs

- Duration of treatment of each prior TKI
- Investigator assessment of best response to each prior TKI
- Number of prior chemotherapy regimens
- Duration of treatment of each chemotherapy regimen
- Investigator assessment of best response to each prior chemotherapy regimen
- Number of prior immunotherapy regimens
- Duration of treatment of each immunotherapy regimen
- Investigator assessment of best response to each prior immunotherapy regimen
- For subjects with prior treated brain metastases, record the method of radiation: whole brain radiation, stereotactic radiosurgery, or both. Record also the dates of the radiation treatment(s), total dose delivered to each course of treatment, and date of last radiation to the start of repotrectinib treatment

- Obtain detailed medical history
- Perform a complete physical examination
- Perform and record vital signs, height, body weight, pain level (0-10), and ECOG PS
- Review concomitant medications
- Female subjects with childbearing potential: Perform a serum pregnancy test
- Collect blood and urine for clinical laboratory assessments and tumor markers
- Collect blood for ccfDNA
- Obtain echocardiogram or MUGA for baseline LVEF assessment (same measurement should be used throughout the study)
- Perform and record triplicate ECGs:
  - **All subjects**: Triplicate 12-lead ECGs obtain approximately 2 minutes apart
- Obtain cardiac troponin-I or troponin-T test
- Obtain baseline tumor assessment including CT chest, abdomen, and pelvis, MRI of the brain and bone scan (if applicable)
- Subject registration

8.1.2 Lead-in PK Cycle 0 Day -3 (Phase 1a Dose Escalation)

- Perform a complete physical examination
- Perform and record vital signs, body weight, pain level (0-10), and ECOG PS
- Review medical history
- Review concomitant medications
- Female subjects with childbearing potential: Perform a serum pregnancy test
- Collect blood and urine for clinical laboratory assessments
- All subjects: Collect a baseline pre-dose PK sample
- Perform and record pre-dose ECGs:

  - **All subjects**: Triplicate 12-lead ECGs approximately 2 minutes apart
- Administer first dose of repotrectinib on an empty stomach (1 hour before dosing and 2 hours after dosing)
- All subjects: Collect PK samples at 1, 2, and 4 hours (± 5 minutes), 6 and 8 hours (± 15 minutes), 24 hours (± 1 hour), and 48 hours (± 2 hours) post-dose. Prior to blood sample collection, record triplicate 12-lead ECGs approximately 2 minutes apart.
- Record any adverse events

8.1.3 Cycle 1 Day 1 (Phase 1a Dose Escalation)

- Perform a complete physical examination
- Perform and record vital signs, body weight, pain level (0-10), and ECOG PS
- Review medical history
- Review concomitant medications
- Record any adverse events
- Collect blood and urine for clinical laboratory assessments
- **All subjects**: Collect a baseline pre-dose PK sample
- Perform and record pre-dose ECGs:
  - **All subjects**: triplicate 12-lead ECGs approximately 2 minutes apart
- Administer repotrectinib on an empty stomach (1 hour before dosing and 2 hours after dosing)
- **All Subjects**: At 4 hours (± 15 minutes) post-dose, record triplicate 12-lead ECGs approximately 2 minutes apart and collect a corresponding PK blood sample
- Dispense study medication for the next cycle (21 days) of treatment

8.1.4 Cycle 1 Day 8 (Phase 1a Dose Escalation)

- Perform a complete physical examination
- Perform and record vital signs, body weight, pain level (0-10), and ECOG PS
- Review medical history
- Assess study drug compliance
- Review concomitant medications
- Record any adverse events
- Collect blood and urine for clinical laboratory assessments
- **All subjects**: Collect a baseline pre-dose PK sample
- Perform and record pre-dose ECGs:
  - **All subjects**: triplicate 12-lead ECGs approximately 2 minutes apart
- Administer repotrectinib on an empty stomach (1 hour before dosing and 2 hours after dosing)

8.1.5 Cycle 1 Day 15 (Phase 1a Dose Escalation)

- Perform a complete physical examination
- Perform and record vital signs, body weight, pain level (0-10), and ECOG PS
- Review medical history
- Assess study drug compliance
- Review concomitant medications
- Record any adverse events
- Collect blood and urine for clinical laboratory assessments
- **All subjects**: Collect a baseline pre-dose PK sample
- Perform and record pre-dose ECGs:
  - **All subjects**: Triplicate 12-lead ECGs approximately 2 minutes apart
- Administer repotrectinib on an empty stomach (1 hour before dosing and 2 hours after dosing)
- **All subjects**: Collect PK samples at 1, 2, and 4 hours (± 5 minutes), 6 hours, 8 hours (± 15 minutes), and 24 hours (± 1 hour) post-dose. ***Prior to each blood sample collection, record triplicate 12-lead ECGs approximately 2 minutes apart***.

8.1.6 Cycle 2 Day 1 (Phase 1a Dose Escalation)

- Perform a complete physical examination
- Perform and record vital signs, body weight, pain level (0-10), and ECOG PS
- Review medical history
- Assess study drug compliance
- Review concomitant medications
- Record any adverse events
- Female subjects with childbearing potential: Perform a serum pregnancy test
- Collect blood and urine for clinical laboratory assessments
- **All subjects**: Collect a baseline pre-dose PK sample
- Perform and record pre-dose ECGs:
  - **All subjects**: triplicate 12-lead ECGs approximately 2 minutes apart
- Administer repotrectinib on an empty stomach (1 hour before dosing and 2 hours after dosing)
- **All subjects**: At 4 hours (± 15 minutes) post-dose, record triplicate 12-lead ECGs approximately 2 minutes apart and collect a corresponding PK blood sample
- Dispense study medication for the next cycle (28 days) of treatment

8.1.7 Cycle 2 Day 15 (Phase 1a Dose Escalation)

- Perform a complete physical examination
- Perform and record vital signs, body weight, pain level (0-10), and ECOG PS
- Review medical history

- Assess study drug compliance
- Review concomitant medications
- Record any adverse events
- Collect blood for clinical laboratory assessments

8.1.8 Cycle 3 Day 1 (Phase 1a Dose Escalation)

- Perform a complete physical examination
- Perform and record vital signs, body weight, pain level (0-10), and ECOG PS
- Review medical history
- Assess study drug compliance
- Review concomitant medications
- Record any adverse events
- Female subjects with childbearing potential: Perform a serum pregnancy test
- Collect blood and urine for clinical laboratory assessments
- Assess radiographic response to repotrectinib
- Schedule the next scans to confirm the responses when a PR or CR is observed (ideally confirmation scan should be scheduled at the end of the next cycle ie, 28 days)
- **All subjects**: Collect a baseline pre-dose PK sample
- Perform and record pre-dose ECGs:
  - **All subjects**: triplicate 12-lead ECGs approximately 2 minutes apart
- Administer repotrectinib on an empty stomach (1 hour before dosing and 2 hours after dosing)
- **All subjects**: At 4 hours (± 15 minutes) post-dose, record triplicate 12-lead ECGs approximately 2 minutes apart and collect a corresponding PK blood sample
- Dispense study medication for the next cycle (28 days) of treatment

8.1.9 Cycle 4 Day 1 (Phase 1a Dose Escalation)

- Perform a complete physical examination
- Perform and record vital signs, body weight, pain level (0-10), and ECOG PS
- Review medical history
- Assess study drug compliance
- Review concomitant medications
- Record any adverse events
- Female subjects with childbearing potential: Perform a serum pregnancy test
- Obtain echocardiogram or MUGA for LVEF assessment (and beginning of every 3 cycles, Cycle 4, 7, 10, 13)
- Collect blood and urine for clinical laboratory assessments
- Assess radiographic response to repotrectinib when a PR or CR is observed at Cycle 3 Day 1 evaluation

- **All subjects**: Collect a baseline pre-dose PK sample
- Perform and record pre-dose ECGs:
  - **All subjects**: triplicate 12-lead ECGs approximately 2 minutes apart
- Administer repotrectinib on an empty stomach (1 hour before dosing and 2 hours after dosing)
- **All subjects**: At 4 hours (± 15 minutes) post-dose, record triplicate 12-lead ECGs approximately 2 minutes apart and collect a corresponding PK blood sample
- Dispense study medication for the next cycle (28 days) of treatment

8.1.10 Cycle 5 and Every 4 Weeks Thereafter at Each Cycle Day 1 Visit (Phase 1a Dose Escalation)

- Perform a complete physical examination
- Perform and record vital signs, body weight, pain level (0-10), and ECOG PS
- Review medical history
- Assess study drug compliance
- Review concomitant medications
- Record any adverse events
- Female subjects with childbearing potential: Perform a serum pregnancy test
- Collect blood and urine for clinical laboratory assessments
- Assess radiographic response to study drug
- Schedule the next scans to confirm the responses when a PR or CR is observed (ideally confirmation scan should be scheduled at the end of the next cycle ie, 28 days)
- Obtain echocardiogram or MUGA for LVEF assessment (starting at the beginning of Cycle 7 and continuing every 3 cycles, eg, Cycles 10, 13 and so on, and at the EOT visit)
- Dispense study medication to last until the next required visit

8.1.11 End of Treatment (Phase 1a Dose Escalation) (within 7 days post last dose of repotrectinib and after decision to end treatment)

- Female subjects with childbearing potential: Perform a serum pregnancy test
- Collect blood and urine for clinical laboratory assessments
- Collect blood for ccfDNA
- Perform and record triplicate ECGs:
  - **All subjects**: Triplicate 12-lead ECGs obtain approximately 2 minutes apart
- Obtain echocardiogram or MUGA for LVEF assessment
- Assess study drug compliance (optional if performed at the time of the last dose)
- The following assessments are optional if performed within the past week:
  - Review concomitant medications

- Record any adverse events
- Review medical history
- Perform complete physical examination
- Perform and record vital signs, body weight, pain level (0-10), and ECOG PS
- Perform tumor imaging, submit scans for BICR within 1 week, and assess locally as per RECIST version 1.1 (optional if performed within the past 4 weeks)

8.1.12 Safety Follow-up (Phase 1a Dose Escalation) (approximately 28 days post the last dose of repotrectinib)

- Review concomitant medications
- Record any adverse events
- Review medical history
- Perform complete physical examination
- Perform and record vital signs, body weight, pain level (0-10), and ECOG PS, as clinically indicated
- Collect blood and urine for clinical laboratory assessments, as clinically indicated

8.1.13 Survival Follow-up (Phase 1a Dose Escalation)

- For subjects discontinuing the study treatment due to documented radiographic progression, obtain survival status *via* phone call or medical chart review, including information about subsequent anticancer therapies (including best response) every 3 months until death, loss of follow-up, or withdrawal of consent, whichever comes first.
- For subjects discontinuing the study treatment prior to documented radiographic progression, tumor assessments should continue on schedule approximately every 2 cycles or at the current scan interval at the time of treatment discontinuation until radiographic evidence of disease progression, the start of a subsequent anticancer therapy, or decision to no longer treat (eg, supportive care only), whichever is first. At that time, survival status (and subsequent anticancer therapy information, including best response, if appropriate) will be collected every 3 months until death, loss of follow-up, or withdrawal of consent, whichever comes first.

8.2 Phase 1b Food Effect

For country-specific requirements, please refer to APPENDIX 8.

8.2.1 Screening Phase 1b Food Effect (within 28 days of the first dose of repotrectinib)

- Obtain Informed Consent
- Eligibility checklist (See complete list in Section 5.1 and Section 5.3)
- Perform molecular testing as per Section 5.1 and Table 24
  - Molecular testing to determine eligibility can be performed in advance with no time limit (eg, the subject can be tested while being treated on another anticancer therapy)

  - Source documents (molecular pathology report detailing the specific test that detect the *ALK*, *ROS1*, or *NTRK* rearrangement) should be submitted for the Sponsor approval of eligibility
  - Submitted archival tissues to Sponsor's designated central laboratory to confirm the specific molecular alteration
  - An optional tumor tissue re-biopsy (for subjects who have failed prior TKIs) during the screening period to investigate resistance mechanism(s) is recommended but not mandatory
- Obtain detailed treatment history
  - Number of prior TKIs
  - Duration of treatment of each prior TKI
  - Investigator assessment of best response to each prior TKI
  - Number of prior chemotherapy regimens
  - Duration of treatment of each chemotherapy regimen
  - Investigator assessment of best response to each prior chemotherapy regimen
  - Number of prior immunotherapy regimens
  - Duration of treatment of each immunotherapy regimen
  - Investigator assessment of best response to each prior immunotherapy regimen
  - For subjects with prior treated brain metastases, record the method of radiation: whole brain radiation, stereotactic radiosurgery, or both. Record also the dates of the radiation treatment(s), total dose delivered to each completed course of treatment, and date of last radiation to the start of repotrectinib treatment
- Obtain detailed medical history
- Perform a complete physical examination
- Perform and record vital signs, height, body weight, pain level (0-10), and ECOG PS
- Review concomitant medications
- Female subjects with childbearing potential: Perform a serum pregnancy test
- Collect blood and urine for clinical laboratory assessments and tumor markers
- Collect blood for ccfDNA
- Perform and record triplicate ECGs:
  - All **subjects**: Triplicate 12-lead ECGs obtain approximately 2 minutes apart
- Obtain echocardiogram or MUGA for baseline LVEF assessment
- Obtain cardiac troponin-I or troponin-T test
- Obtain baseline tumor assessment including CT chest, abdomen and pelvis, MRI of the brain and bone scan (if applicable)
- Subject registration

8.2.2 Lead-in PK Cycle 0 Day -7 (Phase 1b Food Effect): applicable to Cohorts 1-3 only

- **Fasted Treatments**: repotrectinib should be administered with 8 oz (240 mL) of water on an empty stomach. No food and beverages should be allowed 1 hour before dosing and 2 hours after dosing. Water can be allowed as desired except for one hour before and after drug administration.
- **Fed Treatments**: Following an overnight fast of at least 10 hours, subjects should start the recommended meal 30 minutes prior to administration of the drug product. Study subjects should eat this meal in 30 minutes or less; however, repotrectinib should be administered 30 minutes after start of the meal. Repotrectinib should be administered with 8 oz (240 mL) of water. No food should be allowed for at least 2 hours post-dose. Water can be allowed as desired except for one hour before and after drug administration. Subjects should receive standardized meals scheduled at the same time [Guidance for Industry Food Effect Bioavailability and Fed Bioequivalence Studies. http://www.fda.gov/downloads/RegulatoryInformation/Guidances/UCM126833.pdf.].
- For subjects starting with the **Fed** state, a high-fat (approximately 50 percent of total caloric content of the meal) and high-calorie (approximately 800 to 1000 calories) meal is recommended as a test meal. This test meal should derive approximately 150, 250, and 500-600 calories from protein, carbohydrate, and fat, respectively.
  - An example test meal would be 2 eggs fried in butter, 2 strips of bacon, 2 slices of toast with butter, 4 ounces of hash brown potatoes and 8 ounces of whole milk. Substitutions in this test meal can be made as long as the meal provides a similar number of calories from protein, carbohydrate, and fat and has comparable meal volume and viscosity. The caloric breakdown of the test meal should be provided in the CRF.
- Subjects assigned the Fed treatment on PK Lead-in Cycle will follow the Fasted treatment procedure on Cycle 1 Day 1. Subjects assigned the Fasted treatment on PK Lead-in Cycle will follow the Fed treatment procedure on Cycle 1 Day 1.
- Review medical history
- Perform a complete physical examination
- Perform and record vital signs, body weight, pain level (0-10), and ECOG PS
- Review concomitant medications
- Record any adverse events
- Female subjects with childbearing potential: Perform a serum pregnancy test
- Collect blood and urine for clinical laboratory assessments and tumor markers
- All **subjects**: Collect a baseline pre-dose PK sample
- Perform and record pre-dose ECGs:
  - **All subjects**: Triplicate 12-lead ECGs approximately 2 minutes apart
- Administer first dose of repotrectinib
- **All subjects**: Collect PK samples at 1, 2, and 4 hours (±5 minutes), 6 and 8 hours (± 15 minutes), 24 hours (± 1 hour), 48 hours (± 2 hours), and 72 hours (± 2 hours) post-dose.

Prior to blood sample collection, record triplicate 12-lead ECGs approximately 2 minutes apart.

8.2.3 Cycle 1 Day 1 (Phase 1b Food Effect): applicable to Cohorts 1-3 only

- **Fasted Treatments:** repotrectinib should be administered with 8 oz (240 mL) of water on an empty stomach. No food and beverages should be allowed 1 hour before dosing and 2 hours after dosing. Water can be allowed as desired except for 1 hour before and after drug administration. Subject should receive standardized meals scheduled at the same time in each period of the study.
- **Fed Treatments:** Following an overnight fast of at least 10 hours, subjects should start the recommended meal 30 minutes prior to administration of the drug product. Study subjects should eat this meal in 30 minutes or less; however, repotrectinib should be administered 30 minutes after start of the meal. Repotrectinib should be administered with 8 oz (240 mL) of water. No food should be allowed for at least 2 hours post-dose. Water can be allowed as desired except for 1 hour before and after drug administration. Subjects should receive standardized meals scheduled at the same time in each period of the study [Guidance for Industry Food Effect Bioavailability and Fed Bioequivalence Studies. http://www.fda.gov/downloads/Regulatory Information/Guidances/UCM126833.pdf.].
- For subjects starting with the **Fed** state, a high-fat (approximately 50% of total caloric content of the meal) and high-calorie (approximately 800 to 1000 calories) meal is recommended as a test meal. This test meal should derive approximately 150, 250, and 500-600 calories from protein, carbohydrate, and fat, respectively.
  - An example test meal would be 2 eggs fried in butter, 2 strips of bacon, 2 slices of toast with butter, 4 oz of hash brown potatoes and 8 oz of whole-fat milk. Substitutions in this test meal can be made as long as the meal provides a similar number of calories from protein, carbohydrate, and fat and has comparable meal volume and viscosity. The caloric breakdown of the test meal should be provided in the CRF.
- Subjects assigned the Fed treatment on PK Lead-in Cycle will follow the Fasted treatment procedure on Cycle 1 Day 1. Subjects assigned the Fasted treatment on PK Lead-in Cycle will follow the Fed treatment procedure on Cycle 1 Day 1.
- Perform a complete physical examination
- Perform and record vital signs, body weight, pain level (0-10), and ECOG PS
- Review medical history
- Review concomitant medications
- Record any adverse events
- Collect blood and urine for clinical laboratory assessments
- **All subjects**: Collect a baseline pre-dose PK sample
- Perform and record pre-dose ECGs:
  - **All subjects**: triplicate 12-lead ECGs approximately 2 minutes apart
- Administer repotrectinib according to either Fasted or Fed instructions.

- **All subjects**: Collect PK samples at 1, 2, and 4 hours (± 5 minutes), 6 and 8 hours (± 15 minutes), 24 hours (± 1 hour), 48 hours (± 2 hours), and 72 hours (± 2 hours) post-dose. ***Prior to blood sample collection, record triplicate 12-lead ECGs approximately 2 minutes apart.***
- The third dose will be given at Cycle 1 Day 4 after blood sampling.
- Dispense study medication for the remainder of cycle (18 days) of treatment to begin on Cycle 1 Day 4 (there is no dosing on Cycle 1 Day 2 and Day 3).

8.2.4 Cycle 1 Day 8 (Phase 1b Food Effect): applicable to Cohorts 1-3 only

- Perform a complete physical examination
- Perform and record vital signs, body weight, pain level (0-10), and ECOG PS
- Review medical history
- Assess study drug compliance
- Review concomitant medications
- Record any adverse events
- Collect blood and urine for clinical laboratory assessments
- **All subjects**: Collect a baseline pre-dose PK sample
- Perform and record pre-dose ECGs:
  - **All subjects**: triplicate 12-lead ECGs approximately 2 minutes apart
- Administer repotrectinib on an empty stomach (1 hour before dosing and 2 hours after dosing)
- One optional fresh tumor biopsy is recommended between Cycle 1 Day 8 and Cycle 1 Day 15

8.2.5 Cycle 1 Day 15 (Phase 1b Food Effect): applicable to Cohorts 1-3 only

- Perform a complete physical examination
- Perform and record vital signs, body weight, pain level (0-10), and ECOG PS
- Review medical history
- Assess study drug compliance
- Review concomitant medications
- Record any adverse events
- Collect blood and urine for clinical laboratory assessments
- **All subjects**: Collect a baseline pre-dose PK sample
- Perform and record pre-dose ECGs:
  - **All subjects**: Triplicate 12-lead ECGs approximately 2 minutes apart
- Administer repotrectinib on an empty stomach (1 hour before dosing and 2 hours after dosing)
- **All subjects**: At 4 hours (± 15 minutes) post-dose, record triplicate 12-lead ECGs approximately 2 minutes apart and collect a corresponding PK blood sample

8.2.6 Cycle 2 Day 1 (Phase 1b Food Effect): applicable to Cohorts 1-3 only

- Perform a complete physical examination
- Perform and record vital signs, body weight, pain level (0-10), and ECOG PS
- Review medical history
- Assess study drug compliance
- Review concomitant medications
- Record any adverse events
- Female subjects with childbearing potential: Perform a serum pregnancy test
- Collect blood and urine for clinical laboratory assessments
- All subjects: Collect a baseline pre-dose PK sample
- Perform and record pre-dose ECGs:
  - **All subjects**: triplicate 12-lead ECGs approximately 2 minutes apart
- Administer repotrectinib on an empty stomach (1 hour before dosing and 2 hours after dosing)
- **All subjects**: At 4 hours (± 15 minutes) post-dose, record triplicate 12-lead ECGs approximately 2 minutes apart and collect a corresponding PK blood sample
- Dispense study medication for the next cycle (28 days) of treatment

8.2.7 Cycle 2 Day 15 (Phase 1b Food Effect): applicable to Cohorts 1-3 only

- Perform a complete physical examination
- Perform and record vital signs, body weight, pain level (0-10), and ECOG PS
- Review medical history
- Assess study drug compliance
- Review concomitant medications
- Record any adverse events
- Collect blood for clinical laboratory assessments and tumor markers

8.2.8 Cycle 3 Day 1 (Phase 1b Food Effect): applicable to Cohorts 1-3 only

- Perform a complete physical examination
- Perform and record vital signs, body weight, pain level (0-10), and ECOG PS
- Review medical history
- Assess study drug compliance
- Review concomitant medications
- Record any adverse events
- Female subjects with childbearing potential: Perform a serum pregnancy test
- Collect blood and urine for clinical laboratory assessments
- Assess radiographic response to repotrectinib

- Schedule the next scans to confirm the responses when a PR or CR is observed (ideally confirmation scan should be scheduled at the end of the next cycle ie, 28 days)
- **All subjects**: Collect a baseline pre-dose PK sample
- Perform and record pre-dose ECGs:
  - **All subjects**: Triplicate 12-lead ECGs approximately 2 minutes apart
- Administer repotrectinib on an empty stomach (1 hour before dosing and 2 hours after dosing)
- **All subjects**: At 4 hours (+/- 15 minutes) post-dose, record triplicate 12-lead ECGs approximately 2 minutes apart and collect a corresponding PK blood sample
- Dispense study medication for the next cycle (28 days) of treatment

8.2.9 Cycle 4 Day 1 (Phase 1b Food Effect): applicable to Cohorts 1-3 only

- Perform a complete physical examination
- Perform and record vital signs, body weight, pain level (0-10), and ECOG PS
- Review medical history
- Assess study drug compliance
- Review concomitant medications
- Record any adverse events
- Female subjects with childbearing potential: Perform a serum pregnancy test
- Obtain echocardiogram or MUGA for LVEF assessment (and beginning of every 3 cycles, Cycle 4, 7, 10, 13)
- Collect blood and urine for clinical laboratory assessments
- Assess radiographic response to repotrectinib when a PR or CR is observed at Cycle 3 Day 1 evaluation
- **All subjects**: Collect a baseline pre-dose PK sample
- Perform and record pre-dose ECGs:
  - **All subjects**: Triplicate 12-lead ECGs approximately 2 minutes apart
- Administer repotrectinib on an empty stomach (1 hour before dosing and 2 hours after dosing)
- **All subjects**: At 4 hours (± 15 minutes) post-dose, record triplicate 12-lead ECGs approximately 2 minutes apart and collect a corresponding PK blood sample
- Dispense study medication for the next cycle (28 days) of treatment

8.2.10 Cycle 5 and Every 4 Weeks Thereafter at Each Cycle Day 1 Visit (Phase 1b Food Effect): applicable to Cohorts 1-3 only

- Perform a complete physical examination
- Perform and record vital signs, body weight, pain level (0-10), and ECOG PS
- Review medical history
- Assess study drug compliance

- Review concomitant medications
- Record any adverse events
- Female subjects with childbearing potential: Perform a serum pregnancy test
- Collect blood and urine for clinical laboratory assessments and tumor markers
- Assess radiographic response to study drug
- Schedule the next scans to confirm the responses when a PR or CR is observed (ideally confirmation scan should be scheduled at the end of the next cycle ie, 28 days)
- Obtain echocardiogram or MUGA for LVEF assessment (starting at the beginning of Cycle 7 and continuing every 3 cycles, eg, Cycle 10, 13 and so on, and at the EOT visit)
- Dispense study medication to last until the next required visit

8.2.11 End of Treatment (Phase 1b Food Effect) (within 7 days post last dose of repotrectinib and after decision to end treatment)

- Perform tumor biopsy (if clinically feasible and subject has consented to the biopsy) and send tissue to Sponsor's designated central laboratory
- Collect blood for ccfDNA
- Female subjects with childbearing potential: Perform a serum pregnancy test
- Collect blood and urine for clinical laboratory assessments
- Perform and record triplicate ECGs:
- **All subjects**: Triplicate 12-lead ECGs obtain approximately 2 minutes apart
- Obtain echocardiogram or MUGA for LVEF assessment
- Assess study drug compliance (optional if performed at the time of the last dose)
- The following assessments are optional if performed within the past week:
  - Review concomitant medications
  - Record any adverse events
  - Perform complete physical examination
  - Perform and record vital signs, body weight, pain level (0-10), and ECOG PS
  - Perform tumor imaging, submit scans for BICR within 1 week, and assess locally as per RECIST version 1.1 (optional if performed within the past 4 weeks)

8.2.12 Safety Follow-up (Phase 1b Food Effect) (approximately 28 days post the last dose of repotrectinib)

- Review concomitant medications
- Record any adverse events
- Review medical history
- Perform complete physical examination
- Perform and record vital signs, body weight, pain level (0-10), and ECOG PS, as clinically indicated

- Collect blood and urine for clinical laboratory assessments, as clinically indicated

8.2.13 Survival follow-up (Phase 1b Food Effect)

- For subjects discontinuing the study treatment due to documented radiographic progression, obtain survival status via phone call or medical chart review, including information about subsequent anticancer therapies (including best response) every 3 months until death, loss of follow-up, or withdrawal of consent, whichever comes first.
- For subjects discontinuing the study treatment prior to documented radiographic progression, tumor assessments should continue on schedule approximately every 2 cycles or at the current scan interval at the time of treatment discontinuation until radiographic evidence of disease progression, the start of a subsequent anticancer therapy, or decision to no longer treat (eg, supportive care only), whichever is first. At that time, survival status (and subsequent anticancer therapy information, including best response, if appropriate) will be collected every 3 months until death, loss of follow-up, or withdrawal of consent, whichever comes first.

8.3 Phase 1c Study (Cohort 7-9)

For country-specific requirements, please refer to APPENDIX 8.

8.3.1 Screening Phase 1c Dose Escalation (within 28 days of the first dose of repotrectinib)

- Obtain Informed Consent
- Eligibility checklist (see complete list in Section 5.1 and Section 5.3)
- Perform molecular testing as per Section 5.1 and Table 25
  - Molecular testing to determine eligibility can be performed in advance with no time limit (eg, the subject can be tested while being treated on another anticancer therapy)
  - Source documents (molecular pathology report detailing the specific test that detect the *ALK*, *ROS1*, or *NTRK* rearrangement) should be submitted for the Sponsor approval of eligibility (if multiple reports are available, submit them all to ensure a clear understanding of the history of tumor progression)
  - Submitted archival tissues to Sponsor's designated central laboratory to retrospectively confirm the specific molecular alteration
  - An optional tumor tissue re-biopsy (for subjects who have failed prior TKIs) during the screening period to investigate resistance mechanism(s) is recommended but not mandatory
- Obtain detailed treatment history
  - Number of prior TKIs
  - Duration of treatment of each prior TKI
  - Investigator assessment of best response to each prior TKI
  - Number of prior chemotherapy regimens
  - Duration of treatment of each chemotherapy regimen
  - Investigator assessment of best response to each prior chemotherapy regimen
  - Number of prior immunotherapy regimens

- Duration of treatment of each immunotherapy regimen
- Investigator assessment of best response to each prior immunotherapy regimen
- For subjects with prior treated brain metastases, record the method of radiation: whole brain radiation, stereotactic radiosurgery, or both. Record also the dates of the radiation treatment(s), total dose delivered to each course of treatment, and date of last radiation to the start of repotrectinib treatment

- Obtain detailed medical history
- Perform a complete physical examination
- Perform and record vital signs, height, body weight, pain level (0-10), and ECOG PS
- Review concomitant medications
- Female subjects with childbearing potential: Perform a serum pregnancy test
- Collect blood and urine for clinical laboratory assessments and tumor markers
- Collect blood for ccfDNA
- Obtain echocardiogram or MUGA for baseline LVEF assessment
- Perform and record triplicate ECGs:
  - **All subjects**: Triplicate 12-lead ECGs obtain approximately 2 minutes apart
- Obtain cardiac troponin-I or troponin-T test
- Obtain baseline tumor assessment including CT chest, abdomen, and pelvis, MRI of the brain and bone scan (if applicable)
- Subject registration

8.3.2 Cycle 1 Day 1 (Phase 1c Dose Escalation)

- Perform a complete physical examination
- Perform and record vital signs, body weight, pain level (0-10), and ECOG PS
- Review medical history
- Review concomitant medications
- Female subjects with childbearing potential: Perform a serum pregnancy test
- Collect blood and urine for clinical laboratory assessments
- **All subjects**: Collect a baseline pre-dose PK sample. Perform and record pre-dose ECGs:
  - **All subjects**: triplicate 12-lead ECGs approximately 2 minutes apart
- Administer repotrectinib with at least 8 oz. of water and with food or within one hour after food intake
- **All Subjects:** Collect PK samples at 1, 2, 4, 6, 8, and 24 hours post-dose (the 24-hour sample will be collected as the pre-dose collection before the first dose of C1D2). ***Prior to blood sample collection, record triplicate 12-lead ECGs approximately 2 minutes apart***
- Record any adverse events
- Dispense study medication for the next cycle (28 days) of treatment

8.3.3 Cycle 1 Day 8 (Phase 1c Dose Escalation)

- Perform a complete physical examination
- Perform and record vital signs, body weight, pain level (0-10), and ECOG PS
- Review medical history
- Assess study drug compliance
- Review concomitant medications
- Record any adverse events
- Collect blood and urine for clinical laboratory assessments
- **All subjects**: Collect a baseline pre-dose PK
- Perform and record pre-dose ECGs:
  - **All subjects**: triplicate 12-lead ECGs approximately 2 minutes apart
- Administer repotrectinib with at least 8 oz. of water and with food or within one hour after food intake

8.3.4 Cycle 1 Day 15 (Phase 1c Dose Escalation)

- Perform a complete physical examination
- Perform and record vital signs, body weight, pain level (0-10), and ECOG PS
- Review medical history
- Assess study drug compliance
- Review concomitant medications
- Record any adverse events
- Collect blood and urine for clinical laboratory assessments
- **All subjects**: Collect a baseline pre-dose PK sample
- Perform and record pre-dose ECGs:
  - All subjects: Triplicate 12-lead ECGs approximately 2 minutes apart
- Administer repotrectinib with at least 8 oz. of water and with food or within one hour of food intake
- Collect PK samples at 1, 2, 4, 6, 8, and 24 hours post-dose (the 24-hour sample will be collected as the pre-dose collection before the first dose of C1D2). ***Prior to blood sample collection, record triplicate 12-lead ECGs approximately 2 minutes apart***

8.3.5 Cycle 1 Day 22 (Phase 1c Dose Escalation)

- Perform a complete physical examination
- Perform and record vital signs, body weight, pain level (0-10), and ECOG PS
- Review medical history
- Assess study drug compliance
- Review concomitant medications

- Record any adverse events
- Collect blood and urine for clinical laboratory assessments

8.3.6 Cycle 2 Day 1 (Phase 1c Dose Escalation)

- Perform a complete physical examination
- Perform and record vital signs, body weight, pain level (0-10), and ECOG PS
- Review medical history
- Assess study drug compliance
- Review concomitant medications
- Record any adverse events
- Female subjects with childbearing potential: Perform a serum pregnancy test
- Collect blood and urine for clinical laboratory assessments
- **All subjects**: Collect a baseline pre-dose and a 4 -hour post-dose PK sample
- Perform and record pre-dose ECGs:
  - **All subjects**: triplicate 12-lead ECGs approximately 2 minutes apart
- Administer repotrectinib with at least 8 oz. of water and with food or within one hour after food intake
- **All subjects**: At 4 hours (± 15 minutes) post-dose, record triplicate 12-lead ECGs approximately 2 minutes apart and collect a corresponding PK blood sample
- Dispense study medication for the next cycle (28 days) of treatment

8.3.7 Cycle 2 Day 15 (Phase 1c Dose Escalation)

- Perform a complete physical examination
- Perform and record vital signs, body weight, pain level (0-10), and ECOG PS
- Review medical history
- Assess study drug compliance
- Review concomitant medications
- Record any adverse events
- Collect blood for clinical laboratory assessments

8.3.8 Cycle 3 Day 1 (Phase 1c Dose Escalation)

- Perform a complete physical examination
- Perform and record vital signs, body weight, pain level (0-10), and ECOG PS
- Review medical history
- Assess study drug compliance
- Review concomitant medications
- Record any adverse events

- Female subjects with childbearing potential: Perform a serum pregnancy test
- Collect blood and urine for clinical laboratory assessments
- Collect blood for ccfDNA
- Assess radiographic response to repotrectinib
- Schedule the next scans to confirm the responses when a PR or CR is observed (ideally confirmation scan should be scheduled at the end of the next cycle ie, 28 days)
- **All subjects**: Collect a baseline pre-dose PK sample
- Perform and record pre-dose ECGs:
  - **All subjects**: triplicate 12-lead ECGs approximately 2 minutes apart
- Administer repotrectinib with at least 8 oz of water and with food or within one hour after food intake
- **All subjects**: At 4 hours (± 15 minutes) post-dose, record triplicate 12-lead ECGs approximately 2 minutes apart and collect a corresponding PK blood sample
- Dispense study medication for the next cycle (28 days) of treatment

8.3.9 Cycle 4 Day 1 (Phase 1c Dose Escalation)

- Perform a complete physical examination
- Perform and record vital signs, body weight, pain level (0-10), and ECOG PS
- Review medical history
- Assess study drug compliance
- Review concomitant medications
- Record any adverse events
- Female subjects with childbearing potential: Perform a serum pregnancy test
- Obtain echocardiogram or MUGA for LVEF assessment (and beginning of every 3 cycles, Cycle 4, 7, 10, 13)
- Collect blood and urine for clinical laboratory assessments
- If patient is confirmed to have progressed, collect a blood sample (ccfDNA)
- Assess radiographic response to repotrectinib when a PR or CR is observed at Cycle 3 Day 1 evaluation
- **All subjects**: Collect a baseline pre-dose PK sample
- Perform and record pre-dose ECGs:
  - **All subjects**: triplicate 12-lead ECGs approximately 2 minutes apart
- Administer repotrectinib with at least 8 oz of water and with food or within one hour after food intake
- **All subjects**: At 4 hours (± 15 minutes) post-dose, record triplicate 12-lead ECGs approximately 2 minutes apart and collect a corresponding PK blood sample
- Dispense study medication for the next cycle (28 days) of treatment

8.3.10 Cycle 5 and Every 4 Weeks Thereafter at Each Cycle Day 1 Visit (Phase 1c Dose Escalation)

- Perform a complete physical examination
- Perform and record vital signs, body weight, pain level (0-10), and ECOG PS
- Review medical history
- Assess study drug compliance
- Review concomitant medications
- Record any adverse events
- Female subjects with childbearing potential: Perform a serum pregnancy test
- Collect blood and urine for clinical laboratory assessments
- If patient is confirmed to have progressed, collect a blood sample (ccfDNA)
- Assess radiographic response to study drug
- Schedule the next scans to confirm the responses when a PR or CR is observed (ideally confirmation scan should be scheduled at the end of the next cycle ie, 28 days)
- Obtain echocardiogram or MUGA for LVEF assessment (starting at the beginning of Cycle 7 and continuing every 3 cycles, eg, Cycle 10, 13, and so on, and at the EOT visit)
- Dispense study medication to last until the next required visit

8.3.11 End of Treatment (Phase 1c Dose Escalation) (within 7 days post last dose of repotrectinib and after decision to end treatment)

- Female subjects with childbearing potential: Perform a serum pregnancy test
- Collect blood and urine for clinical laboratory assessments
- Collect blood for ccfDNA, if EOT is earlier than Cycle 3 Day 1 or was not collected at progression
- Perform and record triplicate ECGs:
  - **All subjects**: Triplicate 12-lead ECGs obtain approximately 2 minutes apart
- Obtain echocardiogram or MUGA for LVEF assessment
- Assess study drug compliance (optional if performed at the time of the last dose)
- The following assessments are optional if performed within the past week:
  - Review concomitant medications
  - Record any adverse events
  - Review medical history
  - Perform complete physical examination
  - Perform and record vital signs, body weight, pain level (0-10), and ECOG PS
  - Perform tumor imaging, submit scans for BICR within 1 week, and assess locally as per RECIST version 1.1 (optional if performed within the past 4 weeks)

8.3.12 Safety Follow-up (Phase 1c Dose Escalation) (approximately 28 days post the last dose of repotrectinib)

- Review concomitant medications
- Female subjects with childbearing potential: Perform a serum pregnancy test
- Record any adverse events
- Review medical history
- Perform complete physical examination
- Perform and record vital signs, body weight, pain level (0-10), and ECOG PS, as clinically indicated
- Collect blood and urine for clinical laboratory assessments, as clinically indicated

8.3.13 Survival Follow-up (Phase 1c Dose Escalation)

- For subjects discontinuing the study treatment due to documented radiographic progression, obtain survival status via phone call or medical chart review, including information about subsequent anticancer therapies (including best response) every 3 months until death, loss of follow-up, or withdrawal of consent, whichever comes first.
- For subjects discontinuing the study treatment prior to documented radiographic progression, tumor assessments should continue on schedule approximately every 2 cycles or at the current scan interval at the time of treatment discontinuation until radiographic evidence of disease progression, the start of a subsequent anticancer therapy, or decision to no longer treat (eg, supportive care only), whichever is first. At that time, survival status (and subsequent anticancer therapy information, including best response, if appropriate) will be collected every 3 months until death, loss of follow-up, or withdrawal of consent, whichever comes first.

8.4 Phase 2 Study

For country-specific requirements, please refer to APPENDIX 8.

8.4.1 Screening Phase 2 Study

- Obtain Informed Consent
- Eligibility checklist (see complete list in Section 5.2 and Section 5.3)
- Submit adequate tumor tissue sample for prospective or retrospective molecular testing for tumor molecular alteration as per Section 5.2 and Table 26.
  - Source documents of the historical molecular testing (molecular pathology report detailing the specific test that was used to detect the *ROS1* or *NTRK*1-3 gene fusion, should be submitted for the Sponsor approval of eligibility)
  - Submit adequate archival or fresh tissue to Sponsor’s designated central laboratory as described in Section 5.2 (Inclusion Criteria 2) and Section 7.3 to prospectively confirm the specific gene fusion. A fresh biopsy is required if no adequate previously taken and stored tumor sample is available and recommended (for subjects who failed prior TKIs) to investigate resistance mechanism(s).

- Obtain detailed treatment history
  - Number of prior TKIs
  - Duration of treatment of each prior TKI
  - Investigator assessment of best response to each prior TKI
  - Number of prior chemotherapy regimens
  - Duration of treatment of each chemotherapy regimen
  - Investigator assessment of best response to each prior chemotherapy regimen
  - Number of prior immunotherapy regimens
  - Duration of treatment of each immunotherapy regimen
  - Investigator assessment of best response to each prior immunotherapy regimen
  - For subjects with prior treated brain metastases, record the method of radiation: whole brain radiation, stereotactic radiosurgery, or both. Record also the dates of the radiation treatment(s), total dose each completed course of treatment delivered, and date of last radiation to the start of repotrectinib treatment
  - Prior surgery
- Obtain detailed medical history
- Perform a complete physical examination including neurological exam
- Perform and record vital signs, height, body weight, pain level (0-10), and performance scores of either Lansky (< 16 years), Karnofsky (≥ 16 to <18 years) or ECOG PS (≥18 years)
- Review concomitant medications
- Female subjects with childbearing potential: Perform a serum pregnancy test
- Collect blood and urine for clinical laboratory assessments to be sent to Central Lab
- Collect blood for ccfDNA
- Perform and record triplicate ECGs:
  - **All subjects**: Triplicate 12-lead ECGs obtain approximately 2 minutes apart
- Obtain echocardiogram or MUGA for baseline LVEF assessment (same methodology should be utilized throughout)
- Obtain cardiac troponin-I or troponin-T test
- Obtain baseline tumor assessment including CT chest, abdomen and pelvis, gadolinium enhanced MRI of the brain and bone scan (if applicable). If a CT scan, MRI of the brain or bone scan has been performed within 30 days prior to Cycle 1 Day 1 the scan can be used for Screening
- Administer QOL questionnaires:
  - EORTC-QLQ-C30 (all subjects)
  - EORTC-QLQ-LC13 (NSCLC subjects only)
- Subject registration and assignment to one of the 6 expansion cohorts

8.4.2 Re-Screening

- Obtain Informed Consent
- Eligibility checklist (see complete list in Section 5.2 and Section 5.3)
- Obtain detailed treatment history
  - Number of prior TKIs
  - Duration of treatment of each prior TKI
  - Investigator assessment of best response to each prior TKI
  - Number of prior chemotherapy regimens
  - Duration of treatment of each chemotherapy regimen
  - Investigator assessment of best response to each prior chemotherapy regimen
  - Number of prior immunotherapy regimens
  - Duration of treatment of each immunotherapy regimen
  - Investigator assessment of best response to each prior immunotherapy regimen
  - For subjects with prior treated brain metastases, record the method of radiation: whole brain radiation, stereotactic radiosurgery, or both. Record also the dates of the radiation treatment(s), total dose each completed course of treatment delivered, and date of last radiation to the start of repotrectinib treatment
  - Prior surgery
- Obtain detailed medical history
- Perform a complete physical examination, including neurological exam
- Perform and record vital signs, height, body weight, pain level (0-10), and performance scores of either Lansky (< 16 years), Karnofsky (≥ 16 to <18 years), or ECOG PS (≥18 years)
- Review concomitant medications
- Female subjects with childbearing potential: Perform a serum pregnancy test
- Collect blood and urine for clinical laboratory assessments to be sent to Central Lab
- Collect blood for ccfDNA
- Perform and record triplicate ECGs:
  - **All subjects**: Triplicate 12-lead ECGs obtain approximately 2 minutes apart
- Obtain echocardiogram or MUGA for baseline LVEF assessment (same methodology should be utilized throughout)
- Obtain cardiac troponin-I or troponin-T test
- Obtain baseline tumor assessment including CT chest abdomen and pelvis, MRI of the brain and bone scan (if applicable). If a CT scan, MRI of the brain or bone scan has been performed within 30 days prior to Cycle 1 Day 1 the scan can be used for Screening
- Administer QOL questionnaires:
  - EORTC-QLQ-C30 (all subjects)
  - EORTC-QLQ-LC13 (NSCLC subjects only)
- Subject registration and assignment to one of the 6 expansion cohorts

8.4.3 Cycle 1 Day 1 (Phase 2 Study)

- Administer QOL questionnaires:
  - EORTC-QLQ-C30 (all subjects)
  - EORTC-QLQ-LC13 (NSCLC subjects only)
- Review concomitant medications
- Record any adverse events
- Perform ophthalmologic tests with a window of -7 days.
- Perform a complete physical examination including neurological exam
- Perform and record vital signs, body weight, pain level (0-10), and performance scores of either Lansky (< 16 years), Karnofsky (≥ 16 to <18 years) or ECOG PS (≥18 years)
- Female subjects with childbearing potential: Perform a serum pregnancy test (*optional if performed within the past 7 days*)
- Collect blood and urine for clinical laboratory assessments (***optional if performed within the past 7 days***) to be sent to Central Lab
- Perform and record pre-dose ECGs:
  - **All subjects**: triplicate 12-lead ECGs approximately 2 minutes apart
- **All subjects**: Collect a baseline pre-dose PK sample
- Administer repotrectinib with at least 8 oz of water and with or without food.
- **Adolescent subjects age 12-17 years:** Collect PK blood samples at 1, 2, 4, 6 and 24 hours post-dose; record triplicate 12-lead ECGs approximately 2 minutes apart at 4 hours post-dose
- **Adult subjects age 18 years and older:** Collect PK blood sample at 4 hours post-dose and record triplicate 12-lead ECGs approximately 2 minutes apart at 4 hours post-dose
- Dispense repotrectinib

8.4.4 Cycle 1 Day 8 (Phase 2 Study)

- Perform a complete physical examination including neurological exam
- Perform and record vital signs, body weight, pain level (0-10), and performance scores of either Lansky (< 16 years), Karnofsky (≥ 16 to <18 years), or ECOG PS(≥18 years)
- Assess study drug compliance
- Review concomitant medications
- Record any adverse events
- Collect blood and urine for clinical laboratory assessments to be sent to Central Lab

8.4.5 Cycle 1 Day 15 (Phase 2 Study)

- Perform a complete physical examination including neurological exam
- Perform and record vital signs, body weight, pain level (0-10), and performance scores of either Lansky (< 16 years), Karnofsky (≥ 16 to <18 years), or ECOG PS(≥18 years)
- Assess study drug compliance

- Review concomitant medications
- Record any adverse events
- Collect blood and urine for clinical laboratory assessments to be sent to Central Lab
- Perform and record pre-dose ECGs:
  - **All subjects**: triplicate 12-lead ECGs approximately 2 minutes apart
- All Subjects: Collect a baseline pre-dose PK sample
- Administer repotrectinib with at least 8 oz of water and with or without food
- At 4 hours post-dose, record triplicate 12-lead ECGs approximately 2 minutes apart and collect a corresponding PK blood sample
- Investigator Assessment for repotrectinib dosing: Continue 160 mg QD or begin 160 BID
- Dispense repotrectinib

8.4.6 Cycle 1 Day 22 (Phase 2 Study)

- Perform a complete physical examination including neurological exam
- Perform and record vital signs, body weight, pain level (0-10), and performance scores of either Lansky (< 16 years), Karnofsky (≥ 16 to <18 years), or ECOG PS (≥18 years)
- Assess study drug compliance
- Review concomitant medications
- Record any adverse events
- Collect blood and urine for clinical laboratory assessments to be sent to Central Lab

8.4.7 Cycle 2 Day 1 (Phase 2 Study)

- Administer QOL questionnaires:
  - EORTC-QLQ-C30 (all subjects)
  - EORTC-QLQ-LC13 (NSCLC subjects only)
- Perform a complete physical examination including neurological exam
- Perform and record vital signs, body weight, pain level (0-10), and performance scores of either Lansky (< 16 years), Karnofsky (≥ 16 to <18 years), or ECOG PS (≥18 years)
- Assess study drug compliance
- Review concomitant medications
- Record any adverse events
- **Female subjects with childbearing potential**: Perform a serum pregnancy test (a urine pregnancy test may also be collected if the results of the serum pregnancy test are not available before dosing)
- Collect blood and urine for clinical laboratory assessments to be sent to Central Lab
- Perform and record pre-dose ECGs:
  - **All subjects**: triplicate 12-lead ECGs approximately 2 minutes apart

- Collect a baseline pre-dose PK sample
- Administer repotrectinib with at least 8 oz of water and with or without food
- At 4 hours post-dose, record triplicate 12-lead ECGs approximately 2 minutes apart and collect a corresponding PK blood sample
- Dispense repotrectinib for the next cycle (28 days) of treatment

8.4.8 Cycle 2 Day 15 (Phase 2 Study)

- Perform a complete physical examination including neurological exam
- Perform and record vital signs, body weight, pain level (0-10), and performance scores of either Lansky (< 16 years), Karnofsky (≥ 16 to <18 years), or ECOG PS (≥18 years)
- Assess study drug compliance
- Review concomitant medications
- Record any adverse events
- Collect blood for clinical laboratory assessments to be sent to Central Lab

8.4.9 Cycle 3 Day 1 (Phase 2 Study)

- Administer QoL questionnaires:
  - EORTC-QLQ-C30 (all subjects)
  - EORTC-QLQ-LC13 (NSCLC subjects only)
- Perform a complete physical examination including neurological exam
- Perform ophthalmologic tests
- Perform and record vital signs, body weight, pain level (0-10), and performance scores of either Lansky (< 16 years), Karnofsky (≥ 16 to <18 years), or ECOG PS (≥18 years)
- Assess study drug compliance
- Review concomitant medications
- Record any adverse events
- **Female subjects with childbearing potential**: Perform a serum pregnancy test (a urine pregnancy test may also be collected if the results of the serum pregnancy test are not available before dosing)
- Collect blood and urine for clinical laboratory assessments to be sent to Central Lab
- Collect blood for ccfDNA
- Assess radiographic response to study drug
- Schedule the next scans to confirm the responses ≥ 4 weeks later (ideally confirmation scan should be scheduled at end of the next cycle, i.e., 28 days later) when a PR or CR is observed
- Perform and record pre-dose ECGs:
  - **All subjects**: triplicate 12-lead ECGs approximately 2 minutes apart
- Collect a baseline pre-dose PK sample

- Administer repotrectinib with at least 8 oz of water and with or without food
- At 4 hours post-dose, record triplicate 12-lead ECGs approximately 2 minutes apart and collect a corresponding PK blood sample
- Dispense repotrectinib for the next cycle (28 days) of treatment

8.4.10 Cycle 4 Day 1 (Phase 2 Study)

- Administer quality of life questionnaires:
  - QLQ-C30 (all subjects)
  - QLQ-LC13 (NSCLC subjects only)
- Perform a complete physical examination including neurological exam
- Perform and record vital signs, body weight, pain level (0-10), and performance scores of either Lansky (< 16 years), Karnofsky (≥ 16 to <18 years) or ECOG PS (≥18 years)
- Assess study drug compliance
- Review concomitant medications
- Record any adverse events
- **Female subjects with childbearing potential**: Perform a serum pregnancy test (a urine pregnancy test may also be collected if the results of the serum pregnancy test are not available before dosing)
- Obtain echocardiogram or MUGA for LVEF assessment (and beginning of every 3 cycles, Cycle 4, 7, 10, 13)
- Collect blood and urine for clinical laboratory assessments to be sent to Central Lab
- Collect blood for ccfDNA either at progression or at the EOT
- Assess radiographic response to repotrectinib when a PR or CR is observed at Cycle 3 Day 1 evaluation
- Perform and record pre-dose ECGs:
  - **All subjects**: triplicate 12-lead ECGs approximately 2 minutes apart
- Collect a baseline pre-dose PK sample
- Administer repotrectinib with at least 8 oz of water and with or without food
- At 4 hours post-dose, record triplicate 12-lead ECGs approximately 2 minutes apart and collect a corresponding PK blood sample
- Dispense repotrectinib for the next cycle (28 days) of treatment

8.4.11 Cycle 5 and Every 4 Weeks Thereafter at Each Cycle Day 1 Visit (Phase 2 Study)

- Administer quality of life questionnaires:
  - QLQ-C30 (all subjects)
  - QLQ-LC13 (NSCLC subjects only)
- Perform a complete physical examination including neurological exam

- Perform and record vital signs, body weight, pain level (0-10), and performance scores of either Lansky (< 16 years), Karnofsky (≥ 16 to <18 years) or ECOG PS (≥18 years)
- Assess study drug compliance
- Review concomitant medications
- Record any adverse events
- **Female subjects with childbearing potential**: Perform a serum pregnancy test (a urine pregnancy test may also be collected if the results of the serum pregnancy test are not available before dosing)
- Collect blood for ccfDNA either at progression or at the EOT (ccfDNA must be collected within 10 days from either progression or at EOT)
- Assess radiographic response to study drug every 2 cycles from Cycle 5 Day 1 to Cycle 19 Day 1, and every 3 cycles (± 7 days) up to Cycle 37 and then every 4 cycles (± 7 days) thereafter until documented progression of disease. Subject responses will be confirmed ≥ 4 weeks later (ideally confirmation scan should be scheduled at end of the next cycle, i.e., 28 days later) when a PR or CR is observed.
- Obtain echocardiogram or MUGA for LVEF assessment (starting at the beginning of Cycle 7 and continuing every 3 cycles, eg, Cycle 10, 13, and so on, and at the EOT visit)
- Collect blood and urine for clinical laboratory assessments to be sent to Central Lab
- Dispense repotrectinib to last until the next required visit

For subjects who are on study treatment for > 1 year: at Investigator's discretion, may have decreased study visits to align with scan and/or Echo/MUGA dates. Please reference Table 26.

8.4.12 End of Treatment (Phase 2 Study) (within 7 days post last dose of repotrectinib and after decision to end treatment)

- Collect blood for ccfDNA if not collected previously at progression
- Female subjects with childbearing potential: Perform a serum or urine pregnancy test.
- Collect blood and urine for clinical laboratory assessments to be sent to Central Lab
- Perform and record triplicate ECGs:
  - **All subjects**: triplicate 12-lead ECGs approximately 2 minutes apart
- Obtain echocardiogram or MUGA for LVEF assessment
- Administer quality of life questionnaires (optional if performed within the past 2 weeks)
  - QLQ-C30 (all subjects)
  - QLQ-LC13 (NSCLC subjects only)
- Assess study drug compliance (optional if performed at the time of the last dose)
- Perform ophthalmologic tests
- The following assessments are optional if performed within the past week:
  - Review concomitant medications
  - Record any adverse events

- Perform complete physical examination including neurological exam
- Perform and record vital signs, body weight, pain level (0-10), and performance scores of either Lansky (<16 years), Karnofsky (≥16 to <18 years) or ECOG PS (≥18 years)
- Perform tumor imaging, submit scans for BICR as soon as possible (preferably within 1 week), and assess locally as per RECIST version 1.1 (optional if performed within the past 4 weeks)

8.4.13 Safety Follow-up (Phase 2 Study) (approximately 28 days post the last dose of repotrectinib)

- Review concomitant medications
- Female subjects with childbearing potential: Perform a serum or urine pregnancy test
- Record any adverse events
- Perform complete physical examination including neurological exam
- Perform and record vital signs, body weight, pain level (0-10), and performance scores of either Lansky (<16 years), Karnofsky (≥16 to <18 years) or ECOG PS (≥18 years), as clinically indicated
- Collect blood and urine for clinical laboratory assessments, as clinically indicated

8.4.14 Survival Follow-up (Phase 2 Study)

- For subjects discontinuing the study treatment due to documented radiographic progression, obtain survival status via phone call or medical chart review, including information about subsequent anticancer therapies (including best response) every 3 months from the Safety Follow-up visit (Section 8.4.13) until death, loss of follow-up, or withdrawal of consent, whichever comes first.
- For subjects discontinuing the study treatment prior to documented radiographic progression, tumor assessments should continue on schedule approximately every 2 cycles or at the current scan interval at the time of treatment discontinuation until radiographic evidence of disease progression, the start of a subsequent anticancer therapy, or decision to no longer treat (eg, supportive care only), whichever is first. At that time, survival status (and subsequent anticancer therapy information, including best response, if appropriate) will be collected every 3 months from the Safety Follow-up visit (Section 8.4.13) until death, loss of follow-up, or withdrawal of consent, whichever comes first.

8.5 Study Calendars

Table 23: Study Calendar for Phase 1a Dose Escalation Portion of TPX-0005-01

	**Screening**	**Lead-in PK/Cycle 0 (3 days)**	**Cycle 1 (21 days)**			**Cycle 2 (28 days)**		**Cycle 3 (28 days)**	**Cycle 4 (Response Confirmation *)**	**Cycle 5 and Beyond ****	**End of Treatment**	**Safety Follow-up**	**Long-Term Follow-up**
**Protocol Activity**	**Days -28 to -1**	**Day -3**	**Day 1**	**Day 8**	**Day 15**	**Day 1**	**Day 15**	**Day 1**	**Day 1**	**Day 1**			
Visit Window[1]	NA			±1	±1	±2	±2	±2	±2	±2	±7	+7	
Informed Consent[2]	X												
Tumor Molecular Alteration[3]	X												
Tumor Treatment History[4]	X												
Medical History	X	X	X	X	X	X	X	X	X	X	X	X	
Physical Examination	X	X	X	X	X	X	X	X	X	X	X	X	
ECOG Performance Status	X	X	X	X	X	X	X	X	X	X	X	X	
Height	X												
Weight	X	X	X	X	X	X	X	X	X	X	X	X	
Vital Signs[5]	X	X	X	X	X	X	X	X	X	X	X	X	
Dispense repotrectinib			X			X		X	X	X			
repotrectinib Compliance				X	X	X	X	X	X	X	X		

Table 23: Study Calendar for Phase 1a Dose Escalation Portion of TPX-0005-01

	Screening	Lead-in PK/Cycle 0 (3 days)	Cycle 1 (21 days)			Cycle 2 (28 days)		Cycle 3 (28 days)	Cycle 4 (Response Confirmation *)	Cycle 5 and Beyond **	End of Treatment	Safety Follow-up	Long-Term Follow-up
**Protocol Activity**	**Days -28 to -1**	**Day -3**	**Day 1**	**Day 8**	**Day 15**	**Day 1**	**Day 15**	**Day 1**	**Day 1**	**Day 1**			
Laboratory													
Complete Blood Count with Differential[6]	X	X	X	X	X	X	X	X	X	X	X	X	
Reticulocyte Count (% or absolute)	X	X	X			X		X	X	X	X	X	
Complete Metabolic Panel[7]	X	X	X	X	X	X	X	X	X	X	X	X	
Coagulation[8]	X	X	X	X	X	X		X	X	X	X	X	
Endocrine (for male subjects only)[9]	X	X				X		X	X	X	X	X	
Pregnancy Test (serum)[10]	X	X				X		X	X	X	X	X	
Urine Analysis[11]	X	X	X	X	X	X		X	X	X	X	X	
Blood Specimens for ccfDNA Enrichment[12]	X										X		
Cardiac Safety Monitoring													
Triplicate 12-lead ECG[13]	X	X	X	X	X	X		X	X		X		

Table 23: Study Calendar for Phase 1a Dose Escalation Portion of TPX-0005-01

	**Screening**	**Lead-in PK/Cycle 0 (3 days)**	**Cycle 1 (21 days)**			**Cycle 2 (28 days)**		**Cycle 3 (28 days)**	**Cycle 4 (Response Confirmation *)**	**Cycle 5 and Beyond ****	**End of Treatment**	**Safety Follow-up**	**Long-Term Follow-up**
**Protocol Activity**	**Days -28 to -1**	**Day -3**	**Day 1**	**Day 8**	**Day 15**	**Day 1**	**Day 15**	**Day 1**	**Day 1**	**Day 1**			
Echocardiogram/ MUGA for LVEF Assessment[14]	X							X	X	X	X		
Cardiac Troponin-I/T[15]	X												
Pharmacokinetics													
Plasma for Full PK[16]		X			X								
Plasma for Abbreviated PK[17]			X	X		X		X	X				
CSF (Optional)[18]						X							
Tumor Assessment													
CT or MRI (chest/abdomen/pelvis)[19]	X							X	X	X	X		
MRI of Brain[20]	X							X	X	X	X		
Bone Scan[21]	X							X	X	X	X		

Table 23: Study Calendar for Phase 1a Dose Escalation Portion of TPX-0005-01

	**Screening**	**Lead-in PK/Cycle 0 (3 days)**	**Cycle 1 (21 days)**			**Cycle 2 (28 days)**		**Cycle 3 (28 days)**	**Cycle 4 (Response Confirmation *)**	**Cycle 5 and Beyond** **	**End of Treatment**	**Safety Follow-up**	**Long-Term Follow-up**
**Protocol Activity**	**Days -28 to -1**	**Day -3**	**Day 1**	**Day 8**	**Day 15**	**Day 1**	**Day 15**	**Day 1**	**Day 1**	**Day 1**			
Other Clinical Assessments													
Adverse Events		X	X	X	X	X	X	X	X	X	X	X	
Concomitant Medications[22]	X	X	X	X	X	X	X	X	X	X	X	X	
Survival Follow-Up[23]								X					X

Abbreviations: AE, adverse event; BID, twice daily; ccfDNA, circulating cell-free deoxyribonucleic acid; CNS, central nervous system; CR, complete response; CRF, case report form; CSF, cerebrospinal fluid; CT, computerized tomography; CTCAE, Common Terminology Criteria for adverse event; ECG, electrocardiogram; ECHO, echocardiogram; ECOG, Eastern Cooperative Oncology Group; EOT, end of treatment; FFPE, formalin-fixed paraffin-embedded; HPF, high power field; LVEF, left ventricular ejection fraction; MRI, magnetic resonance imaging; MUGA, multigated acquisition; QTc, QT interval corrected for heart rate; PK, pharmacokinetic; PR, partial response; TKI, tyrosine kinase inhibitor

* When PRs or CRs are observed, the responses will be confirmed ≥ 4 weeks later (ideally confirmation scan should be scheduled at end of the next cycle ie, 28 days) after the initial documentation of response by the Investigator.

1 **Visit Window**: The 28-day screening period starts on the day informed consent is signed by the subject. The safety visit should be within 28 days from the last dose of repotrectinib.

2 **Informed Consent**: Must be obtained before undergoing any protocol-specific procedures.

3 **Tumor Molecular Alteration**: Confirmation of *ALK+, ROS1+*, or *NTRK+* rearrangement (submit molecular pathology report-source document) and submit archival tumor tissue (Section 5.1).

4 **Tumor Treatment History**: Documentation of disease progression on prior TKI, duration of treatment with each prior TKI, prior chemotherapy regimens and duration of each chemotherapy regimens, prior immunotherapy regimens and duration of each immunotherapy regimens (if applicable), prior radiation to brain (if brain metastases present, and methods of radiation: whole brain radiation, stereotactic radiosurgery).

5 **Vital Signs**: Body temperature, blood pressure, heart rate, respiratory rate, pain level (0-10).

6 **Complete Blood Count with Differential**: White blood cell count, hemoglobin, platelet, absolute neutrophils, absolute lymphocytes, absolute monocytes, absolute eosinophils, absolute basophils, and absolute or % reticulocytes. On dosing days, assessments will be performed PRIOR to the dose of study medication being given.

7 **Complete Chemistry Panel**: Sodium, potassium, chloride, bicarbonate or CO_2, blood urea nitrogen, creatinine, magnesium, phosphorus, calcium, uric acid, total protein, albumin, glucose, lactate dehydrogenase, aspartate aminotransferase, alanine aminotransferase, total bilirubin, alkaline phosphatase, amylase, lipase, and creatine phosphokinase. On dosing days, assessments will be performed PRIOR to the dose of study medication being given.

8 **Coagulation**: Prothrombin time/international normalized ratio, partial thromboplastin time. On dosing days, assessments will be performed PRIOR to the dose of study medication being given.

9 **Endocrine (for male subjects only)**: Total testosterone, sex hormone-binding globulin, follicle-stimulating hormone, luteinizing hormone, prolactin. These laboratories should be drawn between 8 am to 11 am in the morning.

10 **Serum Pregnancy Test**: For female subjects of childbearing potential, a serum pregnancy test, with sensitivity of at least 25 mIU/mL, will be performed on 2 occasions prior to starting study therapy: once at the start of screening and once at the baseline visit, immediately before investigational product administration. Only subjects with negative serum pregnancy tests are allowed to enroll. Serum pregnancy test will also be performed at the beginning of each cycle of visit.

11 **Urine Analysis**: Urinalysis includes the analysis of protein, glucose, ketones, blood, and specific gravity. A microscopic (white blood cells/HPF, red blood cells/HPF, and any additional findings) exam need only be performed if the urinalysis result is abnormal. More frequent and complete examinations may be performed at the Investigator's discretion if medically indicated; results should be recorded on unscheduled visit CRFs.

12 **Blood Specimens for ccfDNA Enrichment (20 mL blood specimen)**: Blood will be collected across 2 10 ml Streck Cell-Free DNA Blood Collection Tubes (for ccfDNA analysis) prior to the first dose of repotrectinib and at EOT for exploratory ccfDNA molecular profiling analyses. Details for handling of these specimens including processing, storage, and shipment will be provided in the Study Manual.

13 **Triplicate 12-lead ECGs**: At each time point, 3 consecutive 12-lead ECGs will be performed approximately 2 minutes apart to determine mean QTc interval. Triplicate 12-lead ECGs will all be time matched with PK with the exception of during the screening period and at the EOT. Triplicate ECGs will be collected as follows:

a) At Screening.
b) Day -3 (Lead-in/Cycle 0) after single-dose administration at pre-dose (0 hour), at 1, 2, 4, 6, 8, 24, and 48 hours post-dose.
c) Cycle 1 Day 1, at pre-dose (0 hour) and 4 hours post-dose.
d) Cycle 1 Day 8, at pre-dose (0 hour).
e) Cycle 1 Day 15 after single-dose administration at pre-dose (0 hour), at 1, 2, 4, 6, 8, and 24 hours post-dose.
f) Cycles 2-4, Day 1 at pre-dose (0 hour) and 4 hours post-dose.

In addition to these time points, ECGs should be repeated as clinically indicated. Additional ECG time points may be included based on the emerging data. Interpretation of the tracing will be made by a central ECG laboratory. Each ECG tracing should be labeled with the study number, subject initials, subject number, date, and kept in the source documents at the study site. Only clinically significant abnormalities will be recorded in the AE CRF page. Clinically significant abnormalities at screening/baseline should be recorded on the relevant medical history/current medical conditions CRF page. Clinically significant findings must be discussed with the Sponsor Medical Monitor prior to enrolling the subject in the study.

[14] **Echocardiography or MUGA for LVEF Assessments:** Echocardiography to be performed at screening, and after every 3 cycles of treatment (± 7 days) (Cycles 4, 7, 10, 13 and so on), and at the EOT visit (± 7 days).

[15] **Cardiac Troponin-I or Troponin T**: Cardiac troponin-I or Troponin T will be done at baseline then repeated and assessed whenever the ECG morphology demonstrates possible myocardial ischemia or infarction. If troponin CTCAE grade ≥ 3 is seen, this parameter must be repeated twice a week until resolution to CTCAE grade ≤ 1.

[16] **Plasma for Full PK**: Timing for full PK: Lead-in PK pre-dose (time 0), 1, 2, 4, 6, 8, 24, and 48 hours post-dose. Cycle 1 Day 15 pre-dose (time 0), 1, 2, 4, 6, 8, and 24 hours post-dose. For BID dose, Cycle 1 Day15 24 -hour post-dose is relative to the first dose of the day.

[17] **Plasma for Abbreviated PK**: Timing for abbreviated PK: at pre-dose (0 hour) and 4 hours post-dose on Cycle 1 Day 1 and Cycles 2-4 Day 1, and at pre-dose (0 hour) on Cycle 1 Day 8.

[18] **CSF Collection (optional)**: If a subject undergoes a lumbar puncture, a sample of CSF should be collected for exploratory analysis of TPX 0005 concentration, if possible. If a CSF sample is collected, a blood sample for PK analysis should also be collected at approximately the same time as the CSF sample.

[19] **CT or MRI Chest/Abdomen/Pelvis Tumor Assessment**: Tumor assessments will include all known or suspected disease sites. For all tumor assessments, the method of assessment that was used at baseline should be the same method used throughout the study. For subjects who are without documented disease progression, CT or MRI scans to be done at every 2 cycles (7 weeks after starting treatment for the first on treatment assessment and then every 2 cycles up to Cycle 18 and then every 3 cycles up to Cycle 36 and then every 4 cycles thereafter until documented progression of disease). Subject responses will be confirmed ≥ 4 weeks later (ideally confirmation scan should be scheduled at end of the next cycle ie, 28 days) after the initial documentation of response by the Investigator. For subjects who have documented disease progression but are continuing repotrectinib post-RECIST progression, CT or MRI scans are to be done according to local institutional standard of care. For subjects discontinuing the study treatment prior to documented radiographic progression, tumor assessments should continue on schedule approximately every 2 cycles or at the current scan interval at the time of treatment discontinuation until radiographic evidence of disease progression, the start of a subsequent anticancer therapy, withdrawal of consent, or decision to no longer treat (eg, supportive care only), whichever is first. The end of treatment scan is optional ONLY if performed within the past 4 weeks. Every effort should be made to maintain the assessment scheduling relative to Cycle 1 Day 1 especially if there are dosing cycle interruptions due to toxicities. For all subjects, copies of radiologic images must be available for independent central radiology review as determined by the Sponsor Medical Monitor.

[20] **MRI of Brain**: Gadolinium contrast-enhanced MRI must be used for assessment of CNS lesions at baseline with contingent slices of 1 mm for lesions 5 to 10 mm in size, 5 mm for lesions greater than 10 mm. For the Phase 1b food effect portion of the study, only subjects with documented baseline CNS metastases will be assessed by MRI of the brain at every 2 cycles (after 7 weeks for the first assessment and then every 2 cycles up to Cycle 18 and then every 3 cycles up to Cycle 36 and then every 4 cycles thereafter until documented progression of disease). Subject responses will be confirmed ≥ 4 weeks later after (ideally confirmation scan should be scheduled at end of the next cycle ie, 28 days) the initial documentation of response by the Investigator. For subjects who have documented disease progression but are still continuing repotrectinib post-RECIST progression, MRI scans of the brain are to be done according to local institutional standard of care. Subject responses will be confirmed ≥ 4 weeks later (ideally confirmation scan should be scheduled at end of the next cycle ie, 28 days) after the initial documentation of response by the Investigator. For subjects discontinuing the study treatment prior to documented radiographic progression, tumor assessments should continue on schedule approximately every 2 cycles or at the current scan interval at the time of treatment discontinuation until radiographic evidence of disease progression, the start of a subsequent anticancer therapy, withdrawal of consent, or decision to no longer treat (eg, supportive care only), whichever is first. The end of treatment scan is optional ONLY if performed within the past 4 weeks.

[21] **Bone Scans**: Bone scans (or bone MRI if preferred by Investigator) will be performed at baseline if bone metastases are suspected and repeated every 3 cycles while on study only if evidence of bone metastases is observed at baseline. Subject responses will be confirmed ≥ 4 weeks later (ideally confirmation scan

should be scheduled at end of the next cycle ie, 28 days) after the initial documentation of response by the Investigator. For subjects discontinuing the study treatment prior to documented radiographic progression, tumor assessments should continue on schedule approximately every 2 cycles or at the current scan interval at the time of treatment discontinuation until radiographic evidence of disease progression, the start of a subsequent anticancer therapy, withdrawal of consent, or decision to no longer treat (eg, supportive care only), whichever is first. The end of treatment scan is optional ONLY if performed within the past 4 weeks.

[22] **Concomitant Medications and Non-Drug Supportive Interventions**: All concomitant medications and non-drug supportive interventions should be recorded in the CRF.

[23] **Survival Follow-Up**: For subjects discontinuing the study treatment due to documented radiographic progression, obtain survival status via phone call or medical chart review, including information about subsequent anticancer therapies (including best response) every 3 months until death, loss of follow-up, or withdrawal of consent, whichever comes first.

[**] Patients on treatment after 1 year: Patients on treatment greater than 1 year (≥ 12 cycles), at the Investigator's discretion, may have decreased study visits to align with scan and/or ECHO/MUGA dates. Please note the following protocol requirements:

- Tumor assessments (scans) are to be performed at Screening (30 days prior to Cycle 1 Day 1), C3D1 (± 7 days), every 2 cycles (± 7 days) up to Cycle 19, every 3 cycles (± 7 days) up to Cycle 37, and then every 4 cycles (± 7 days) thereafter until documented progression of disease.
- ECHO or MUGA for LVEF assessment are to be performed at screening and after every 3 cycles of treatment (± 7 days) (Cycles 4, 7, 10, 13 and so on), and at the EOT visit (± 7 days).
- As the frequency of the scans and ECHO/MUGA do not align beyond Cycle 12 of treatment, once a subject is able to begin a reduced visit schedule, in order to ensure proper safety assessments are being conducted for all subjects, the following is provided as guidance on mandatory and optional visits.
  - Mandatory Visits : Cycle 13, Cycle 15, Cycle 16, Cycle 17, Cycle 19, Cycle 22, Cycle 25, Cycle 28, Cycle 31, Cycle 34, Cycle 37, Cycle 40, Cycle 41, Cycle 43, Cycle 45, Cycle 46, Cycle 49, Cycle 52, Cycle 53, Cycle 55, Cycle 57, Cycle 58, Cycle 61, Cycle 64, Cycle 65, Cycle 67, Cycle 69, Cycle 70, Cycle 73, Cycle 76, Cycle 77, Cycle 79, Cycle 81
  - Optional Visits : Cycle 12, Cycle 14, Cycle 18, Cycle 20, Cycle 21, Cycle 23, Cycle 24, Cycle 26, Cycle 27, Cycle 29, Cycle 30, Cycle 32, Cycle 33, Cycle 35, Cycle 36, Cycle 38, Cycle 39, Cycle 42, Cycle 44, Cycle 47, Cycle 48, Cycle 50, Cycle 51, Cycle 54, Cycle 56, Cycle 59, Cycle 60, Cycle 62, Cycle 63, Cycle 66, Cycle 68, Cycle 71, Cycle 72, Cycle 74, Cycle 75, Cycle 78, Cycle 80

Table 24: Study Calendar for Phase 1b Food Effect Portion of TPX-0005-01 (Applicable for Cohorts 1-3 ONLY)

	**Screening**	**Lead-in PK/Cycle 0 (7 days)**	**Cycle 1 (21 days)**			**Cycle 2 (28 days)**		**Cycle 3 (28 days)**	**Cycle 4 (Response Confirmation*)**	**Cycle 5 and Beyond******	**End of Treatment**	**Safety Follow-up**	**Long-Term Follow-up**
**Protocol Activity**	**Days -28 to -1**	**Day -7**	**Day 1**	**Day 8**	**Day 15**	**Day 1**	**Day 15**	**Day 1**	**Day 1**	**Day 1**			
Visit Window[1]	NA			±1	±1	±2	±2	±2	±2	±2	±7	+7	
Informed Consent[2]	X												
Tumor Molecular Alteration[3]	X												
Tumor Treatment History[4]	X												
Medical History	X	X	X	X	X	X	X	X	X	X	X	X	
Physical Examination	X	X	X	X	X	X	X	X	X	X	X	X	
ECOG Performance Status	X	X	X	X	X	X	X	X	X	X	X	X	
Height	X												
Weight	X	X	X	X	X	X	X	X	X	X	X	X	
Vital Signs[5]	X	X	X	X	X	X	X	X	X	X	X	X	
Dispense repotrectinib			X			X		X	X	X			
repotrectinib Compliance				X	X	X	X	X	X	X	X		

Table 24: Study Calendar for Phase 1b Food Effect Portion of TPX-0005-01 (Applicable for Cohorts 1-3 ONLY)

	**Screening**	**Lead-in PK/Cycle 0 (7 days)**	**Cycle 1 (21 days)**			**Cycle 2 (28 days)**		**Cycle 3 (28 days)**	**Cycle 4 (Response Confirmation*)**	**Cycle 5 and Beyond****	**End of Treatment**	**Safety Follow-up**	**Long-Term Follow-up**
**Protocol Activity**	**Days -28 to -1**	**Day -7**	**Day 1**	**Day 8**	**Day 15**	**Day 1**	**Day 15**	**Day 1**	**Day 1**	**Day 1**			
Laboratory													
Complete Blood Count with Differential[6]	X	X	X	X	X	X	X	X	X	X	X	X	
Reticulocyte Count (% or absolute)	X	X	X			X		X	X	X	X	X	
Complete Metabolic Panel[7]	X	X	X	X	X	X	X	X	X	X	X	X	
Coagulation[8]	X	X	X	X	X	X		X	X	X	X	X	
Endocrine (for male subjects only)[9]	X		X			X		X	X	X	X	X	
Pregnancy Test (serum)[10]	X	X				X		X	X	X	X	X	
Urine Analysis[11]	X	X	X	X	X	X		X	X	X	X	X	
Blood Specimens for ccfDNA Enrichment[12]	X										X		
Cardiac Safety Monitoring													
Triplicate 12-lead ECG[13]	X	X	X	X	X	X		X	X		X		

Table 24: Study Calendar for Phase 1b Food Effect Portion of TPX-0005-01 (Applicable for Cohorts 1-3 ONLY)

	**Screening**	**Lead-in PK/Cycle 0 (7 days)**	**Cycle 1 (21 days)**			**Cycle 2 (28 days)**		**Cycle 3 (28 days)**	**Cycle 4 (Response Confirmation*)**	**Cycle 5 and Beyond****[**]**	**End of Treatment**	**Safety Follow-up**	**Long-Term Follow-up**
**Protocol Activity**	**Days -28 to -1**	**Day -7**	**Day 1**	**Day 8**	**Day 15**	**Day 1**	**Day 15**	**Day 1**	**Day 1**	**Day 1**			
Echocardiogram/MUGA for LVEF Assessment[14]	X							X	X	X	X		
Cardiac Troponin-I/T[15]	X												
Pharmacokinetics													
Plasma for Full PK[16]		X	X										
Plasma for Abbreviated PK[17]				X	X	X		X	X				
CSF (optional)[18]								X					
Tumor Assessment													
CT or MRI (Chest/Abdomen/Pelvis)[19]	X							X	X	X	X		
MRI of Brain[20]	X							X	X	X	X		
Bone Scan[21]	X							X	X	X	X		

Table 24: Study Calendar for Phase 1b Food Effect Portion of TPX-0005-01 (Applicable for Cohorts 1-3 ONLY)

	Screening	Lead-in PK/Cycle 0 (7 days)	Cycle 1 (21 days)			Cycle 2 (28 days)		Cycle 3 (28 days)	Cycle 4 (Response Confirmation*)	Cycle 5 and Beyond**	End of Treatment	Safety Follow-up	Long-Term Follow-up
**Protocol Activity**	**Days -28 to -1**	**Day -7**	**Day 1**	**Day 8**	**Day 15**	**Day 1**	**Day 15**	**Day 1**	**Day 1**	**Day 1**			
Other Clinical Assessments													
Adverse Events		X	X	X	X	X	X	X	X	X	X	X	
PD Assessment[22]				X									
Concomitant Medications[23]	X	X	X	X	X	X	X	X	X	X	X	X	
Survival Follow-Up[24]													X

Abbreviations: AE, adverse event; ccfDNA, circulating cell-free deoxyribonucleic acid; CNS, central nervous system; CR, complete response; CRF, case report form; CSF, cerebrospinal fluid; CT, computerized tomography; CTCAE, Common Terminology Criteria for adverse event; ECG, electrocardiogram; ECOG, Eastern Cooperative Oncology Group; EOT, end of treatment; FFPE, formalin-fixed paraffin-embedded; HPF, high power field; LVEF, left ventricular ejection fraction; MRI, magnetic resonance imaging; MUGA, multigated acquisition; QTc, QT interval corrected for heart rate; PD, pharmacodynamic; PK, pharmacokinetic; PR, partial response; TKI, tyrosine kinase inhibitor

* When PRs or CRs are observed, the responses will be confirmed ≥ 4 weeks later (ideally confirmation scan should be scheduled at end of the next cycle ie, 28 days) after the initial documentation of response by the Investigator.

1 **Visit Window**: The 28-day screening period starts on the day informed consent is signed by the subject. The safety visit should be within 28 days from the last dose of repotrectinib.

2 **Informed Consent**: Must be obtained before undergoing any protocol-specific procedures.

3 **Tumor Molecular Alteration**: Confirmation of *ALK+, ROS1+, or NTRK+* rearrangement (submit molecular pathology report-source document) and submit archival tumor tissue (Section 5.1).

4 **Tumor Treatment History**: Documentation of disease progression on prior TKI, duration of treatment with each prior TKI, prior chemotherapy regimens and duration of each chemotherapy regimens, prior immunotherapy regimens and duration of each immunotherapy regimens (if applicable), prior radiation to brain (if brain metastases present, and methods of radiation: whole brain radiation, stereotactic radiosurgery).

5 **Vital Signs**: Body temperature, blood pressure, heart rate, respiratory rate, pain level (0-10).

6 **Complete Blood Count with Differential**: White blood cell count, hemoglobin, platelet, absolute neutrophils, absolute lymphocytes, absolute monocytes, absolute eosinophils, absolute basophils, and absolute or % reticulocytes. On dosing days, assessments will be performed PRIOR to the dose of study medication being given.

7 **Complete Chemistry Panel**: Sodium, potassium, chloride, bicarbonate or CO2, blood urea nitrogen, creatinine, magnesium, phosphorus, calcium, uric acid, total protein, albumin, glucose, lactate dehydrogenase, aspartate aminotransferase, alanine aminotransferase, total bilirubin, alkaline phosphatase, amylase, lipase, and creatine phosphokinase. On dosing days, assessments will be performed PRIOR to the dose of study medication being given.

8 **Coagulation**: Prothrombin time/international normalized ratio, partial thromboplastin time. On dosing days, assessments will be performed PRIOR to the dose of study medication being given.

9 **Endocrine (for male subjects only)**: Total testosterone, sex hormone-binding globulin, follicle-stimulating hormone, luteinizing hormone, prolactin. These laboratories should be drawn between 8 am to 11 am in the morning.

10 **Serum Pregnancy Test**: For female subjects of childbearing potential, a serum pregnancy test, with sensitivity of at least 25 mIU/mL, will be performed on 2 occasions prior to starting study therapy: once at the start of screening and once at the baseline visit, immediately before investigational product administration. Only subjects with negative serum pregnancy tests are allowed to enroll. Serum pregnancy test will also be performed at the beginning of each cycle of visit.

11 **Urine Analysis**: Urinalysis includes the analysis of protein, glucose, ketones, blood, and specific gravity. A microscopic (white blood cells/high power field [HPF], red blood cells/HPF, and any additional findings) exam need only be performed if the urinalysis result is abnormal. More frequent and complete examinations may be performed at the Investigator’s discretion if medically indicated; results should be recorded on unscheduled visit CRFs.

12 **Blood Specimens for ccfDNA Enrichment (20 mL blood specimen)**: Blood will be collected across 2 10-ml Streck Cell-Free DNA Blood Collection Tubes (for ccfDNA analysis) prior to the first dose of repotrectinib and at the EOT for ccfDNA enumeration and exploratory molecular profiling analyses. Details for handling of these specimens including processing, storage, and shipment will be provided in the Study Manual.

13 **Triplicate 12-lead ECGs**: At each time point, 3 consecutive 12-lead ECGs will be performed approximately 2 minutes apart to determine mean QTc interval. Triplicate 12-lead ECGs will all be time matched with PK with the exception of during the screening period and at EOT. Triplicate ECGs will be collected as follows:

g) At Screening.

h) Day -7 (Lead-in/Cycle 0) after single-dose administration at pre-dose (0 hour), at 1, 2, 4, 6, 8, 24, 48, and 72 hours post-dose.

i) Cycle 1 Day 1, after single-dose administration at pre-dose (0 hour), at 1, 2, 4, 6, 8, 24, 48, and 72 hours post-dose.

j) Cycle 1 Day 8, at pre-dose (0 hour).

k) Cycle 1 Day 15, Cycles 2-4, Day 1 at pre-dose (0 hour) and 4 hours post-dose.

In addition to these time points, ECGs should be repeated as clinically indicated. Additional ECG time points may be included based on the emerging data. Interpretation of the tracing will be made by a central ECG laboratory. Each ECG tracing should be labeled with the study number, subject initials, subject

number, date, and kept in the source documents at the study site. Only clinically significant abnormalities will be recorded in the AE CRF page. Clinically significant abnormalities at screening/baseline should be recorded on the relevant medical history/current medical conditions CRF page. Clinically significant findings must be discussed with the Sponsor Medical Monitor prior to enrolling the subject in the study.

[14] **Echocardiography/MUGA for LVEF Assessments:** Echocardiography to be performed at screening, and after every 3 cycles of treatment (± 7 days) (Cycles 4, 7, 10, 13 and so on), and at the EOT visit (± 7 days).

[15] **Cardiac Troponin-I or Troponin T**: Cardiac troponin-I or Troponin T will be done at baseline then repeated and assessed whenever the ECG morphology demonstrates possible myocardial ischemia or infarction. If troponin CTCAE grade ≥ 3 is seen, this parameter must be repeated twice a week until resolution to CTCAE grade ≤ 1.

[16] **Plasma for Full PK**: Timing for full PK: Lead-in PK pre-dose (0 hour), 1, 2, 4, 6, 8, 24, 48, and 72 hours post-dose. Cycle 1 Day 1 pre-dose (time 0), 1, 2, 4, 6, 8, 24, 48, and 72 hours post-dose.

[17] **Plasma for Abbreviated PK**: Timing for abbreviated PK: at pre-dose (0 hour) and 4 hours post-dose on Cycle 1 Day 15 and Cycles 2-4 Day 1, and pre-dose (0 hour) on Cycle 1 Day 8.

[18] **CSF Collection (optional)**: If a subject undergoes a lumbar puncture, a sample of CSF should be collected for exploratory analysis of repotrectinib concentration, if possible. If a CSF sample is collected, a blood sample for PK analysis should also be collected at approximately the same time as the CSF sample.

[19] **CT or MRI Chest/Abdomen/Pelvis Tumor Assessment:** Tumor assessments will include all known or suspected disease sites. For all tumor assessments, the method of assessment that was used at baseline should be the same method used throughout the study. For subjects who are without documented disease progression, CT or MRI scans to be done at every 2 cycles (7 weeks after starting treatment for the first on treatment assessment and then every 2 cycles up to Cycle 18 and then every 3 cycles up to Cycle 36 and then every 4 cycles thereafter until documented progression of disease). Subject responses will be confirmed ≥ 4 weeks later (ideally confirmation scan should be scheduled at end of the next cycle ie, 28 days) after the initial documentation of response by the Investigator. For subjects who have documented disease progression but are continuing repotrectinib post-RECIST progression, CT or MRI scans are to be done according to local institutional standard of care. For subjects discontinuing the study treatment prior to documented radiographic progression, tumor assessments should continue on schedule approximately every 2 cycles or at the current scan interval at the time of treatment discontinuation until radiographic evidence of disease progression, the start of a subsequent anticancer therapy, withdrawal of consent, or decision to no longer treat (eg, supportive care only), whichever is first. The end of treatment scan is optional ONLY if performed within the past 4 weeks. Every effort should be made to maintain the assessment scheduling relative to Cycle 1 Day 1 especially if there are dosing cycle interruptions due to toxicities. For all subjects, copies of radiologic images must be available for independent central radiology review as determined by the Sponsor Medical Monitor.

[20] **MRI of Brain**: Gadolinium contrast-enhanced MRI must be used for assessment of CNS lesions at baseline with contingent slices of 1 mm for lesions 5 to 10 mm in size, 5 mm for lesions greater than 10 mm. For the Phase 1b food effect portion of the study, only subjects with documented baseline CNS metastases will be assessed by MRI of the brain at every 2 cycles (after 7 weeks for the first assessment and then every 2 cycles up to Cycle 18 and then every 3 cycles up to Cycle 36 and then every 4 cycles thereafter until documented progression of disease). Subject responses will be confirmed ≥ 4 weeks later after (ideally confirmation scan should be scheduled at end of the next cycle ie, 28 days) the initial documentation of response by the Investigator. For subjects who have documented disease progression but are still continuing repotrectinib post-RECIST progression, MRI scans of the brain are to be done according to local institutional standard of care. Subject responses will be confirmed ≥ 4 weeks later (ideally confirmation scan should be scheduled at end of the next cycle ie, 28 days) after the initial documentation of response by the Investigator. For subjects discontinuing the study treatment prior to documented radiographic progression, tumor assessments should continue on schedule approximately every 2 cycles or at the current scan interval at the time of treatment discontinuation

until radiographic evidence of disease progression, the start of a subsequent anticancer therapy, withdrawal of consent, or decision to no longer treat (eg, supportive care only), whichever is first. The end of treatment scan is optional ONLY if performed within the past 4 weeks.

21 **Bone Scans**: Bone scans (or bone MRI if preferred by Investigator) will be performed at baseline if bone metastases are suspected and repeated every 3 cycles while on study only if evidence of bone metastases is observed at baseline. Subject responses will be confirmed ≥ 4 weeks later (ideally confirmation scan should be scheduled at end of the next cycle ie, 28 days) after the initial documentation of response by the Investigator. For subjects discontinuing the study treatment prior to documented radiographic progression, tumor assessments should continue on schedule approximately every 2 cycles or at the current scan interval at the time of treatment discontinuation until radiographic evidence of disease progression, the start of a subsequent anticancer therapy, withdrawal of consent, or decision to no longer treat (eg, supportive care only), whichever is first. The end of treatment scan is optional ONLY if performed within the past 4 weeks.

22 **PD** Assessment: One optional fresh tumor tissue re-biopsy is recommended between Cycle 1 Day 8 and Cycle 1 Day 15 to assess the PD effect of TPX 0005. The biopsy should obtain enough amount of tumor to generate at least 10 (preferably 15) unbaked, 5-micron thick unstained slides containing FFPE tumor tissue. Specimens will be sent to the Sponsor-designated central laboratories for PD study.

23 **Concomitant Medications and Non-Drug Supportive Interventions**: All concomitant medications and non-drug supportive interventions should be recorded in the CRF.

24 **Survival Follow-Up**: For subjects discontinuing the study treatment due to documented radiographic progression, obtain survival status via phone call or medical chart review, including information about subsequent anticancer therapies (including best response) every 3 months until death, loss of follow-up, or withdrawal of consent, whichever comes first.

** Patients on treatment after 1 year: Patients on treatment greater than 1 year (≥ 12 cycles), at the Investigator's discretion, may have decreased study visits to aligning with scan and/or Echo/MUGA dates. Please note the following protocol requirements:

- Tumor assessments (scans) are to be performed at Screening (30 days prior to Cycle 1 Day 1), C3D1 (± 7 days), every 2 cycles (± 7 days) up to Cycle 19, every 3 cycles (± 7 days) up to Cycle 37, and then every 4 cycles (± 7 days) thereafter until documented progression of disease.
- ECHO or MUGA for LVEF assessment are to be performed at screening and after every 3 cycles of treatment (± 7 days) (Cycles 4, 7, 10, 13 and so on), and at the EOT visit (± 7 days).
- As the frequency of the scans and ECHO/MUGA do not align beyond Cycle 12 of treatment, once a subject is able to begin a reduced visit schedule, in order to ensure proper safety assessments are being conducted for all subjects, the following is provided as guidance on mandatory and optional visits.
  - Mandatory Visits : Cycle 13, Cycle 15, Cycle 16, Cycle 17, Cycle 19, Cycle 22, Cycle 25, Cycle 28, Cycle 31, Cycle 34, Cycle 37, Cycle 40, Cycle 41, Cycle 43, Cycle 45, Cycle 46, Cycle 49, Cycle 52, Cycle 53, Cycle 55, Cycle 57, Cycle 58, Cycle 61, Cycle 64, Cycle 65, Cycle 67, Cycle 69, Cycle 70, Cycle 73, Cycle 76, Cycle 77, Cycle 79, Cycle 81
  - Optional Visits : Cycle 12, Cycle 14, Cycle 18, Cycle 20, Cycle 21, Cycle 23, Cycle 24, Cycle 26, Cycle 27, Cycle 29, Cycle 30, Cycle 32, Cycle 33, Cycle 35, Cycle 36, Cycle 38, Cycle 39, Cycle 42, Cycle 44, Cycle 47, Cycle 48, Cycle 50, Cycle 51, Cycle 54, Cycle 56, Cycle 59, Cycle 60, Cycle 62, Cycle 63, Cycle 66, Cycle 68, Cycle 71, Cycle 72, Cycle 74, Cycle 75, Cycle 78, Cycle 80

Table 25: Study Calendar for Phase 1c Study of TPX-0005-01

	Screening	Cycle 1 28 days				Cycle 2 28 days		Cycle 3 28 days	Cycle 4 (Response confirmation*)	Cycle 5 and Beyond **	EOT	Safety Follow-up	Long-Term Follow-up
**Protocol Activity**	**Days -28 to -1**	**Day 1**	**Day 8**	**Day 15**	**Day 22**	**Day 1**	**Day 15**	**Day 1**	**Day 1**	**Day 1**			
Visit Window[1]	NA		±1	±1	±1	±2	±2	±2	±2	±2	±7	+7	
Informed Consent[2]	X												
Tumor Molecular Alteration[3]	X												
Tumor Treatment History[4]	X												
Medical History	X	X	X	X	X	X	X	X	X	X	X	X	
Physical Examination	X	X	X	X	X	X	X	X	X	X	X	X	
ECOG Performance Status	X	X	X	X	X	X	X	X	X	X	X	X	
Height	X												
Weight	X	X	X	X	X	X	X	X	X	X	X	X	
Vital Signs[5]	X	X	X	X	X	X	X	X	X	X	X	X	
Dispense repotrectinib		X				X		X	X	X			
Repotrectinib Compliance			X	X	X	X	X	X	X	X	X		

Approved v2.0 930210036 2.0

Table 25: Study Calendar for Phase 1c Study of TPX-0005-01

	Screening	Cycle 1 28 days				Cycle 2 28 days		Cycle 3 28 days	Cycle 4 (Response confirmation*)	Cycle 5 and Beyond **	EOT	Safety Follow-up	Long-Term Follow-up
**Protocol Activity**	**Days -28 to -1**	**Day 1**	**Day 8**	**Day 15**	**Day 22**	**Day 1**	**Day 15**	**Day 1**	**Day 1**	**Day 1**			
Laboratory													
Complete Blood Count with Differential[6]	X	X	X	X	X	X	X	X	X	X	X	X	
Reticulocyte Count (% or absolute)	X	X				X		X	X	X	X	X	
Complete Metabolic Panel[7]	X	X	X	X		X	X	X	X	X	X	X	
Coagulation[8]	X	X	X	X	X	X		X	X	X	X	X	
Endocrine (for male subjects only)[9]	X	X				X		X	X	X	X	X	
Pregnancy Test (serum)[10]	X	X				X		X	X	X	X	X	
Urine Analysis[11]	X	X	X	X		X		X	X	X	X	X	
Blood Specimens for ccfDNA Enrichment[12]	X							X	X		X		
Cardiac Safety Monitoring													
Triplicate 12-lead ECG[13]	X	X	X	X		X		X	X		X		

Table 25: Study Calendar for Phase 1c Study of TPX-0005-01

	**Screening**	**Cycle 1 28 days**				**Cycle 2 28 days**		**Cycle 3 28 days**	**Cycle 4 (Response confirmation*)**	**Cycle 5 and Beyond ****	**EOT**	**Safety Follow-up**	**Long-Term Follow-up**
**Protocol Activity**	**Days -28 to -1**	**Day 1**	**Day 8**	**Day 15**	**Day 22**	**Day 1**	**Day 15**	**Day 1**	**Day 1**	**Day 1**			
Echocardiogram/MUGA for LVEF Assessment[14]	X							X	X	X	X		
Cardiac Troponin-I/T[15]	X												
Pharmacokinetics													
Plasma for Full repotrectinib PK[16]		X		X									
Plasma for Abbreviated PK[17]			X			X		X	X				
CSF (Optional)[18]						X							
Tumor Assessment													
CT or MRI (chest/abdomen/pelvis)[19]	X							X	X	X	X		
MRI of Brain[20]	X							X	X	X	X		
Bone Scan[21]	X												

Table 25: Study Calendar for Phase 1c Study of TPX-0005-01

	**Screening**	**Cycle 1 28 days**				**Cycle 2 28 days**		**Cycle 3 28 days**	**Cycle 4 (Response confirmation*)**	**Cycle 5 and Beyond** **	**EOT**	**Safety Follow-up**	**Long-Term Follow-up**
**Protocol Activity**	**Days -28 to -1**	**Day 1**	**Day 8**	**Day 15**	**Day 22**	**Day 1**	**Day 15**	**Day 1**	**Day 1**	**Day 1**			
Other Clinical Assessments													
Adverse Events		X	X	X	X	X	X	X	X	X	X	X	
Concomitant Medications[22]	X	X	X	X	X	X	X	X	X	X	X	X	
Survival Follow-Up[23]													X

Abbreviations: AE, adverse event; BID, twice daily; ccfDNA, circulating cell-free deoxyribonucleic acid; CNS, central nervous system; CR, complete response; CRF, case report form; CSF, cerebrospinal fluid; CT, computerized tomography; CTCAE, Common Terminology Criteria for adverse event; ECHO, echocardiogram; ECG, electrocardiogram; ECOG, Eastern Cooperative Oncology Group; EOT, end of treatment; FFPE, formalin-fixed paraffin-embedded; HPF, high power field; LVEF, left ventricular ejection fraction; MRI, magnetic resonance imaging; MUGA, multigated acquisition; QTc, QT interval corrected for heart rate; PK, pharmacokinetic; PR, partial response; TKI, tyrosine kinase inhibitor

* When PRs or CRs are observed, the responses will be confirmed ≥ 4 weeks later (ideally confirmation scan should be scheduled at end of the next cycle ie, 28 days) after the initial documentation of response by the Investigator.

1 **Visit Window**: The 28-day screening period starts on the day informed consent is signed by the subject. The safety visit should be within 28 days from the last dose of repotrectinib.

2 **Informed Consent**: Must be obtained before undergoing any protocol-specific procedures.

3 **Tumor Molecular Alteration**: Confirmation of *ALK+, ROS1+*, or *NTRK+* rearrangement (submit molecular pathology report-source document) and submit archival tumor tissue (Section 5.1).

4 **Tumor Treatment History**: Documentation of disease progression on prior TKI, duration of treatment with each prior TKI, prior chemotherapy regimens and duration of each chemotherapy regimens, prior immunotherapy regimens and duration of each immunotherapy regimens (if applicable), prior radiation to brain (if brain metastases present, and methods of radiation: whole brain radiation, stereotactic radiosurgery).

5 **Vital Signs**: Body temperature, blood pressure, heart rate, respiratory rate, pain level (0-10).

6 **Complete Blood Count with Differential**: White blood cell count, hemoglobin, platelet, absolute neutrophils, absolute lymphocytes, absolute monocytes, absolute eosinophils, absolute basophils, and absolute or % reticulocytes. On dosing days, assessments will be performed PRIOR to the dose of study medication being given.

7 **Complete Chemistry Panel**: Sodium, potassium, chloride, bicarbonate or CO2, blood urea nitrogen, creatinine, magnesium, phosphorus, calcium, uric acid, total protein, albumin, glucose, lactate dehydrogenase, aspartate aminotransferase, alanine aminotransferase, total bilirubin, alkaline phosphatase, amylase, lipase, and creatine phosphokinase. On dosing days, assessments will be performed PRIOR to the dose of study medication being given.

8 **Coagulation**: Prothrombin time/international normalized ratio, partial thromboplastin time. On dosing days, assessments will be performed PRIOR to the dose of study medication being given.

9 **Endocrine (for male subjects only)**: Total testosterone, sex hormone-binding globulin, follicle-stimulating hormone, luteinizing hormone, prolactin. These laboratories should be drawn between 8 am to 11 am in the morning.

10 **Serum Pregnancy Test**: For female subjects of childbearing potential, a serum pregnancy test, with sensitivity of at least 25 mIU/mL, will be performed on 2 occasions prior to starting study therapy: once at the start of screening and once at the baseline visit, immediately before investigational product administration. Only subjects with negative serum pregnancy tests are allowed to enroll. Serum pregnancy test will also be performed at the beginning of each cycle of visit.

11 **Urine Analysis**: Urinalysis includes the analysis of protein, glucose, ketones, blood, and specific gravity. A microscopic (white blood cells/HPF, red blood cells/HPF, and any additional findings) exam need only be performed if the urinalysis result is abnormal. More frequent and complete examinations may be performed at the Investigator's discretion if medically indicated; results should be recorded on unscheduled visit CRFs.

12 **Blood Specimens for ccfDNA Enrichment (20 mL blood specimen)**: Blood will be collected in two 10 ml Streck Cell-Free DNA Blood Collection Tubes prior to the first dose of repotrectinib, at the Cycle 3 Day 1 visit, and either at the time of tumor progression or EOT (whichever is earlier) for exploratory ccfDNA molecular profiling analyses. Details for handling of these specimens including processing, storage, and shipment will be provided in the Study Manual. For the Cycle 4, Day 1 visit, collect a blood sample (ccfDNA) if patient is confirmed to have progressed.

13 **Triplicate 12-lead ECGs**: At each time point, 3 consecutive 12-lead ECGs will be performed approximately 2 minutes apart to determine mean QTc interval. Triplicate 12-lead ECGs will all be time matched with PK with the exception of during the screening period and at the EOT. Triplicate ECGs will be collected as follows:

l) At Screening.

m) Cycle 1 Day 1, at pre-dose (0 hour), at 1, 2, 4, 6, 8, and 24 hours post-dose.

n) Cycle 1 Day 8, at pre-dose (0 hour).

o) Cycle 1 Day 15 after single-dose administration at pre-dose (0 hour), at 1, 2, 4, 6, 8, and 24 hours post-dose.

p) Cycles 2-4, Day 1 at pre-dose (0 hour) and 4 hours post-dose.

In addition to these time points, ECGs should be repeated as clinically indicated. Additional ECG time points may be included based on the emerging data. Interpretation of the tracing will be made by a central ECG laboratory. Each ECG tracing should be labeled with the study number, subject initials, subject number, date, and kept in the source documents at the study site. Only clinically significant abnormalities will be recorded in the AE CRF page. Clinically significant abnormalities at screening/baseline should be recorded on the relevant medical history/current medical conditions CRF page. Clinically significant findings must be discussed with the Sponsor Medical Monitor prior to enrolling the subject in the study.

[14] **Echocardiography or MUGA for LVEF Assessments**: Echocardiography to be performed at screening, and after every 3 cycles of treatment (± 7 days) (Cycles 4, 7, 10, 13 and so on), and at the EOT visit (± 7 days). **Cardiac Troponin-I or Troponin T**: Cardiac troponin-I or troponin T will be done at baseline then repeated and assessed whenever the ECG morphology demonstrates possible myocardial ischemia or infarction. If troponin CTCAE grade ≥ 3 is seen, this parameter must be repeated twice a week until resolution to CTCAE grade ≤ 1.

[15] **Cardiac Troponin-I or Troponin T:** Cardiac troponin-I or troponin T will be done at baseline then repeated and assessed whenever the ECG morphology demonstrates possible myocardial ischemia or infarction. If troponin CTCAE grade ≥ 3 is seen, this parameter must be repeated twice a week until resolution to CTCAE grade ≤ 1.

[16] **Plasma for Full repotrectinib PK**: Timing for full PK: Cycle 1 Day 1 PK pre-dose (time 0), 1, 2, 4, 6, 8- and 24 -hours post-dose. Cycle 1 Day 15 pre-dose (time 0), 1, 2, 4, 6, 8 and 24 hours post-dose.

[17] **Plasma for Abbreviated PK**: Timing for abbreviated PK: at pre-dose (0 hour) and 4 hours post-dose on Cycles 2-4 Day 1, and at pre-dose (0 hour) on Cycle 1 Day 8.

[18] **CSF Collection (optional):** If a subject undergoes a lumbar puncture, a sample of CSF should be collected for exploratory analysis of repotrectinib concentration, if possible. If a CSF sample is collected, a blood sample for PK analysis should also be collected at approximately the same time as the CSF sample.

[19] **CT or MRI Chest/Abdomen/Pelvis Tumor Assessment:** Tumor assessments will include all known or suspected disease sites. For all tumor assessments, the method of assessment that was used at baseline should be the same method used throughout the study. For subjects who are without documented disease progression, CT or MRI scans to be done at every 2 cycles (7 weeks after starting treatment for the first on treatment assessment and then every 2 cycles up to Cycle 18 and then every 3 cycles up to Cycle 36 and then every 4 cycles thereafter until documented progression of disease). Subject responses will be confirmed ≥ 4 weeks later (ideally confirmation scan should be scheduled at end of the next cycle ie, 28 days) after the initial documentation of response by the Investigator. For subjects who have documented disease progression but are continuing repotrectinib post-RECIST progression, CT or MRI scans are to be done according to local institutional standard of care. For subjects discontinuing the study treatment prior to documented radiographic progression, tumor assessments should continue on schedule approximately every 2 cycles or at the current scan interval at the time of treatment discontinuation until radiographic evidence of disease progression, the start of a subsequent anticancer therapy, withdrawal of consent, or decision to no longer treat (eg, supportive care only), whichever is first. The end of treatment scan is optional ONLY if performed within the past 4 weeks. Every effort should be made to maintain the assessment scheduling relative to Cycle 1 Day 1 especially if there are dosing cycle interruptions due to toxicities. For all subjects, copies of radiologic images must be available for independent central radiology review as determined by the Sponsor Medical Monitor.

[20] **MRI of Brain:** Gadolinium contrast-enhanced MRI must be used for assessment of CNS lesions at baseline with contingent slices of 1 mm for lesions 5 to 10 mm in size, 5 mm for lesions greater than 10 mm. For the Phase 1b food effect portion of the study, only subjects with documented baseline CNS metastases will be assessed by MRI of the brain at every 2 cycles (after 7 weeks for the first assessment and then every 2 cycles up to Cycle 18 and then every 3 cycles up to Cycle 36 and then every 4 cycles thereafter until documented progression of disease). Subject responses will be confirmed ≥ 4 weeks later after (ideally confirmation scan should be scheduled at end of the next cycle ie, 28 days) the initial documentation of response by the Investigator. For subjects who have documented disease progression but are still continuing repotrectinib post-RECIST progression, MRI scans of the brain are to be done according to local institutional standard of care. Subject responses will be confirmed ≥ 4 weeks later (ideally confirmation scan should be scheduled at end of the next cycle ie, 28 days) after the initial documentation of response by the Investigator. For subjects discontinuing the study treatment prior to documented radiographic progression, tumor assessments should continue on schedule approximately every 2 cycles or at the current scan interval at the time of treatment discontinuation until radiographic evidence of disease progression, the start of a subsequent anticancer therapy, withdrawal of consent, or decision to no longer treat (eg, supportive care only), whichever is first. The end of treatment scan is optional ONLY if performed within the past 4 weeks.

[21] **Bone Scans**: Bone scans (or bone MRI if preferred by Investigator) will be performed at baseline if bone metastases are suspected. These scans are only repeated for subjects having a baseline scan, to confirm the absence of bone metastases in case of a CR. In addition, bone scans can be performed if clinically indicated per Investigator's discretion. Subject responses will be confirmed ≥ 4 weeks later (ideally confirmation scan should be scheduled at end of the next cycle ie, 28 days) after the initial documentation of response by the Investigator.

[22] **Concomitant Medications and Non-Drug Supportive Interventions**: All concomitant medications and non-drug supportive interventions should be recorded in the CRF.

[23] **Survival Follow-Up**: For subjects discontinuing the study treatment due to documented radiographic progression, obtain survival status via phone call or medical chart review, including information about subsequent anticancer therapies (including best response) every 3 months until death, loss of follow-up, or withdrawal of consent, whichever comes first.

[**] Patients on treatment after 1 year: Patients on treatment greater than 1 year (≥ 12 cycles), at the Investigator's discretion, may have decreased study visits to align with scan and/or Echo/MUGA dates. Please note the following protocol requirements:

- Tumor assessments (scans) are to be performed at Screening (30 days prior to Cycle 1 Day 1), C3D1 (± 7 days), every 2 cycles (± 7 days) up to Cycle 19, every 3 cycles (± 7 days) up to Cycle 37, and then every 4 cycles (± 7 days) thereafter until documented progression of disease.
- ECHO or MUGA for LVEF assessment are to be performed at screening and after every 3 cycles of treatment (± 7 days) (Cycles 4, 7, 10, 13 and so on), and at the EOT visit (± 7 days).
- As the frequency of the scans and ECHO/MUGA do not align beyond Cycle 12 of treatment, once a subject is able to begin a reduced visit schedule, in order to ensure proper safety assessments are being conducted for all subjects, the following is provided as guidance on mandatory and optional visits.
  - Mandatory Visits : Cycle 13, Cycle 15, Cycle 16, Cycle 17, Cycle 19, Cycle 22, Cycle 25, Cycle 28, Cycle 31, Cycle 34, Cycle 37, Cycle 40, Cycle 41, Cycle 43, Cycle 45, Cycle 46, Cycle 49, Cycle 52, Cycle 53, Cycle 55, Cycle 57, Cycle 58, Cycle 61, Cycle 64, Cycle 65, Cycle 67, Cycle 69, Cycle 70, Cycle 73, Cycle 76, Cycle 77, Cycle 79, Cycle 81
  - Optional Visits : Cycle 12, Cycle 14, Cycle 18, Cycle 20, Cycle 21, Cycle 23, Cycle 24, Cycle 26, Cycle 27, Cycle 29, Cycle 30, Cycle 32, Cycle 33, Cycle 35, Cycle 36, Cycle 38, Cycle 39, Cycle 42, Cycle 44, Cycle 47, Cycle 48, Cycle 50, Cycle 51, Cycle 54, Cycle 56, Cycle 59, Cycle 60, Cycle 62, Cycle 63, Cycle 66, Cycle 68, Cycle 71, Cycle 72, Cycle 74, Cycle 75, Cycle 78, Cycle 80

Approved v2.0 930210036 2.0

Table 26: Study Calendar for Phase 2 Study of TPX-0005-01

	Screening	Cycle 1 (28 days)				Cycle 2 (28 days)		Cycle 3 (28 days)	Cycle 4 (28 days)	Cycle 5 and beyond	>1 Year of Treatment[¥]	End of Treatment[**]	Safety Follow-Up[≠]	Long-Term Follow-Up
**Protocol Activity**	**Days -28 to -1**	**Day 1**	**Day 8**	**Day 15**	**Day 22**	**Day 1**	**Day 15**	**Day 1**	**Day 1**	**Day 1**	**Day 1**			
Visit Window[1]	NA		±1	±1	±1	±2	±2	±2	±2	±2		±7	+7	
Informed Consent[2]	X													
Pre-screening molecular testing Informed Consent	No time restriction													
Tumor Molecular Alteration[3]	X													
Tumor Treatment History[4]	X													
Medical History	X													
Physical Examination	X	X	X	X	X	X	X	X	X	X	X	X	X	
Ophthalmologic Tests[5]		X						X			X	X		
Lansky (< 16 years) OR Karnofsky (≥ 16 to <18 years) OR ECOG PS (≥18 years) Performance Status	X	X	X	X	X	X	X	X	X	X	X	X	X	
Height	X													

Table 26: Study Calendar for Phase 2 Study of TPX-0005-01

	**Screening**	**Cycle 1 (28 days)**				**Cycle 2 (28 days)**		**Cycle 3 (28 days)**	**Cycle 4 (28 days)**	**Cycle 5 and beyond**	**>1 Year of Treatment[¥]**	**End of Treatment[**]**	**Safety Follow-Up[≠]**	**Long-Term Follow-Up**
**Protocol Activity**	**Days -28 to -1**	**Day 1**	**Day 8**	**Day 15**	**Day 22**	**Day 1**	**Day 15**	**Day 1**	**Day 1**	**Day 1**	**Day 1**			
Weight	X	X	X	X	X	X	X	X	X	X	X	X	X	
Vital Signs[6]	X	X	X	X	X	X	X	X	X	X	X	X	X	
Repotrectinib administration in clinic[7]		X		X		X		X	X					
Dispense repotrectinib		X		X		X		X	X	X	X			
Repotrectinib Compliance			X	X	X	X	X	X	X	X	X	X		
Laboratory														
Complete Blood Count with Differential[8]	X	X	X	X	X	X	X	X	X	X	X	X	X	
Reticulocyte Count (% or absolute)	X	X				X		X	X	X	X	X	X	
Complete Chemistry Panel [9]	X	X	X	X	X	X	X	X	X	X	X	X	X	
Coagulation[10]	X	X		X		X		X	X	X	X	X	X	

Table 26: Study Calendar for Phase 2 Study of TPX-0005-01

	Screening	Cycle 1 (28 days)				Cycle 2 (28 days)		Cycle 3 (28 days)	Cycle 4 (28 days)	Cycle 5 and beyond	>1 Year of Treatment[¥]	End of Treatment[**]	Safety Follow-Up[≠]	Long-Term Follow-Up
**Protocol Activity**	**Days -28 to -1**	**Day 1**	**Day 8**	**Day 15**	**Day 22**	**Day 1**	**Day 15**	**Day 1**	**Day 1**	**Day 1**	**Day 1**			
Endocrine (for male subjects only)[11]	X	X				X		X	X	X	X	X	X	
Pregnancy Test (serum)[12]	X	X				X		X	X	X	X	X	X	
Urine Analysis[13]	X	X	X	X	X	X		X	X	X	X	X	X	
Blood Specimens for ccfDNA Enrichment[14]	X							X				X		
Cardiac Safety Monitoring														
Triplicate 12-lead ECG[15]	X	X		X		X		X	X			X		
Echocardiogram/MUGA for LVEF Assessment[16]	X								X	X	X	X		
Cardiac Troponin-I/T[17]	X													
Pharmacokinetics														

Table 26: Study Calendar for Phase 2 Study of TPX-0005-01

	Screening	Cycle 1 (28 days)				Cycle 2 (28 days)		Cycle 3 (28 days)	Cycle 4 (28 days)	Cycle 5 and beyond	>1 Year of Treatment[¥]	End of Treatment[**]	Safety Follow-Up[≠]	Long-Term Follow-Up
**Protocol Activity**	**Days -28 to -1**	**Day 1**	**Day 8**	**Day 15**	**Day 22**	**Day 1**	**Day 15**	**Day 1**	**Day 1**	**Day 1**	**Day 1**			
Plasma for PK[18]		X		X		X		X	X					
Tumor Assessment														
CT or MRI (Chest/Abdomen/Pelvis)[19]	X							X		X	X	X		
Confirmatory CT or MRI (Chest/Abdomen/Pelvis) and Brain MRI*									X	X				
MRI of Brain[20]	X							X		X	X	X		
Bone Scan[21]	X													
Other Clinical Assessments														
Adverse Events		X	X	X	X	X	X	X	X	X	X	X	X	
EORTC-QLQ-C30 and EORTC-QLQ-LC-13[22]	X	X				X		X	X	X	X	X		
Concomitant Medications[23]	X	X	X	X	X	X	X	X	X	X	X	X	X	

Table 26: Study Calendar for Phase 2 Study of TPX-0005-01

	Screening	Cycle 1 (28 days)				Cycle 2 (28 days)		Cycle 3 (28 days)	Cycle 4 (28 days)	Cycle 5 and beyond	>1 Year of Treatment[¥]	End of Treatment[**]	Safety Follow-Up[≠]	Long-Term Follow-Up
**Protocol Activity**	**Days -28 to -1**	**Day 1**	**Day 8**	**Day 15**	**Day 22**	**Day 1**	**Day 15**	**Day 1**	**Day 1**	**Day 1**	**Day 1**			
Survival Follow-Up[24]														X

Abbreviations: AE, adverse event; BID, twice daily; C, cycle; ccfDNA, circulating cell-free deoxyribonucleic acid; CNS, central nervous system; CR, complete response; CRF, case report form; CSF, cerebrospinal fluid; CT, computerized tomography; CTCAE, Common Terminology Criteria for adverse event; D, day; ECHO, echocardiogram; ECG, electrocardiogram; ECOG, Eastern Cooperative Oncology Group; EOT, end of treatment; FFPE, formalin-fixed paraffin-embedded; HPF, high power field; LVEF, left ventricular ejection fraction; MRI, magnetic resonance imaging; MUGA, multigated acquisition; NGS, next-generation sequencing; qPCR, quantitative PCR; QTc, QT interval corrected for heart rate; PK, pharmacokinetic; PR, partial response; TKI, tyrosine kinase inhibitor

[1] **Visit Window**: The 28-day screening period starts on the day informed consent is signed by the subject. The safety visit should be within 28 days from the last dose of repotrectinib. All study visits (visit date calculations) are based off of the Cycle 1 Day 1 visit date, regardless of dose interruptions; and evaluations are to be performed according to the schedule.

[2] **Informed Consent**: Must be obtained before undergoing any protocol-specific procedures.

[3] **Tumor Molecular Alteration**: If an NGS or qPCR was used for local testing (submit molecular pathology report-source document), the fusion status will be retrospectively confirmed using adequate tumor tissue by a central diagnostic laboratory test selected by the Sponsor. If a FISH test was used for local testing (submit molecular pathology report-source document), prospective confirmation of a *ROS1*+ or *NTRK*+ gene fusion by a central diagnostic laboratory test selected by the Sponsor is required PRIOR to enrollment. Pre-screening molecular testing Informed Consent can be used for tissue testing for molecular alterations required for enrollment.

[4] **Tumor Treatment History**: Documentation of disease progression on prior TKI, duration of treatment with each prior TKI, prior chemotherapy regimens and duration of each chemotherapy regimens, prior immunotherapy regimens and duration of each immunotherapy regimens (if applicable), prior radiation to brain (if brain metastases present, and methods of radiation: whole brain radiation, stereotactic radiosurgery).

[5] **Ophthalmologic Tests:** Seven Ophthalmologic tests are mandatory and will be performed at Cycle 1 Day 1 (C1D1) with a window of -7 days (baseline), Cycle 1 Cycle 3 Day 1 (C3D1, ±2 days), annually thereafter while on treatment (±7 days), at the end of treatment (±7 days) and as clinically indicated.

[6] **Vital Signs**: Body temperature, blood pressure, heart rate, respiratory rate, pain level (0-10)

[7] **Repotrectinib Administration in Clinic:** During Cycle 1 Day 1, Cycle 1 Day 15, and Cycles 2-4 Day 1 repotrectinib morning dose should be administered in clinic due to pre-dose PK collection and ECG timepoints, as per protocol Sections: 7.7.1.5 and 8.4.

8 **Complete Blood Count with Differential**: White blood cell count, hemoglobin, platelet, absolute neutrophils, absolute lymphocytes, absolute monocytes, absolute eosinophils, absolute basophils, and absolute or % reticulocytes. On dosing days, assessments will be performed PRIOR to the dose of study medication being given.

9 **Complete Chemistry Panel**: Sodium, potassium, chloride, bicarbonate or CO2, creatine phosphokinase, blood urea nitrogen, creatinine, magnesium, phosphorus, total calcium, uric acid, total protein, albumin, glucose, lactate dehydrogenase, aspartate aminotransferase, alanine aminotransferase, total bilirubin, alkaline phosphatase, amylase, and lipase. On dosing days, assessments will be performed PRIOR to the dose of study medication being given.

10 **Coagulation**: Prothrombin time/international normalized ratio, partial thromboplastin time. On dosing days, assessments will be performed PRIOR to the dose of study medication being given.

11 **Endocrine (for male subjects only)**: Total testosterone, sex hormone-binding globulin, follicle-stimulating hormone, luteinizing hormone, prolactin. These laboratories should be drawn between 8 am to 11 am in the morning.

12 **Serum Pregnancy Test**: For female subjects of childbearing potential, a serum pregnancy test, with sensitivity of at least 25 mIU/mL, will be performed on 2 occasions prior to starting study therapy: once at the start of screening and once at the baseline visit, immediately before study drug administration. Only subjects with negative serum pregnancy tests are allowed to enroll. A pregnancy test at C1D1 is optional if performed within the past 7 days. Serum pregnancy test is mandatory at the beginning of each cycle; however, if the results of the serum pregnancy test are not available during the clinic visits (eg, C2D1, C3D1, C4D1 etc., EOT visit and safety follow-up visit), a urine pregnancy test may be performed.

13 **Urine Analysis**: Urinalysis includes the analysis of protein, glucose, ketones, blood, and specific gravity. A microscopic (WBC/HPF, RBC/HPF, and any additional findings) exam need only be performed if the urinalysis result is abnormal. More frequent and complete examinations may be performed at the Investigator's discretion if medically indicated; results should be recorded on unscheduled visit CRFs.

14 **Blood Specimens for ccfDNA Enrichment (20 mL blood specimen)**: Blood will be collected in two 10 ml Streck Cell-Free DNA Blood Collection Tubes prior to the first dose of repotrectinib, at the Cycle 3 Day 1 visit, and either at the time of tumor progression or EOT (whichever is earlier) for exploratory ccfDNA molecular profiling analyses. Details for handling of these specimens including processing, storage, and shipment will be provided in the Study Manual. For the Cycle 4 Day 1 visit, and beyond, collect a blood sample (ccfDNA) if patient is confirmed to have progressed.

15 **Triplicate 12-lead ECGs**: At each time point, 3 consecutive 12-lead ECGs will be performed approximately 2 minutes apart to determine mean QTc interval. Triplicate 12-lead ECGs will all be time matched with PK with the exception of during the screening period and at EOT. Triplicate ECGs will be collected for ALL subjects as follows:

q) At Screening,

r) Cycle 1 Day 1, at pre-dose (0 hour) and 4 hours post-dose,

s) Cycle 1 Day 15 at pre-dose (0 hour) and 4 hours post-dose,

t) Cycles 2-4, Day 1 at pre-dose (0 hour) and 4 hours post-dose,

u) End of treatment.

In addition to these time points, ECGs should be repeated as clinically indicated. Additional ECG time points may be included based on the emerging data. Interpretation of the tracing will be made by a central ECG laboratory. Each ECG tracing should be labeled with the study number, subject initials, subject number, date, and kept in the source documents at the study site. Only clinically significant abnormalities will be recorded in the AE CRF page. Clinically

significant abnormalities at screening/baseline should be recorded on the relevant medical history/current medical conditions CRF page. Clinically significant findings must be discussed with the Sponsor Medical Monitor prior to enrolling the subject in the study.

16 **Echocardiography or MUGA for LVEF Assessments**: Echocardiography to be performed at screening, and after every 3 cycles of treatment (± 7 days) (Cycles 4, 7, 10, 13 and so on), and at the EOT visit (± 7 days).

17 **Cardiac Troponin-I or Troponin T**: Cardiac troponin-I or Troponin T will be done at baseline then repeated and assessed whenever the ECG morphology demonstrates possible myocardial ischemia or infarction. Troponin levels should be monitored according to Investigator's medical judgment. If troponin CTCAE grade ≥ 3 is seen, this parameter must be repeated twice a week until resolution to CTCAE grade ≤ 1.

18 **Plasma for PK**: For adult subjects age 18 years and older: at pre-dose (0 hour) and 4 hours post-dose on Cycle 1 Day 1, Cycle 1 Day 15, and Cycles 2-4 Day 1. For adolescent subjects age 12-17 years, serial PK samples will be collected on Cycle 1 Day 1: at pre-dose (0 hour), 1, 2, 4, 6 and 24 hours post-dose. In addition, pre-dose and 4 hours post-dose PK samples will be collected on Cycle 1 Day 15, and Cycles 2-4 Day 1.

19 **CT or MRI Chest/Abdomen/Pelvis Tumor Assessment**: Tumor assessments will include all known or suspected disease sites. For all tumor assessments, the method of assessment that was used at baseline should be the same method used throughout the study. For subjects who are without documented disease progression, CT or MRI scans to be done at every 2 cycles (± 7 days) at Screening (30 days prior to Cycle 1 Day 1), C3D1 (± 7 days), every 2 cycles (± 7 days) up to Cycle 19 and then every 3 cycles (± 7 days) up to Cycle 37 and then every 4 cycles (± 7 days) thereafter until documented progression of disease. Subject responses will be confirmed ≥ 4 weeks after the initial documentation of response by the Investigator. For subjects who have documented disease progression but are continuing repotrectinib post-RECIST progression, CT or MRI scans are to be done according to local institutional standard of care. For subjects discontinuing the study treatment prior to documented radiographic progression, tumor assessments should continue on schedule approximately every 2 cycles or at the current scan interval at the time of treatment discontinuation until radiographic evidence of disease progression, the start of a subsequent anticancer therapy, withdrawal of consent, or decision to no longer treat (eg, supportive care only), whichever is first. The end of treatment scan is optional ONLY if performed within the past 4 weeks. Disease progression must be confirmed by BICR before treatment discontinuation and whenever possible the Investigator should consult the Sponsor Medical Monitor prior to treatment discontinuation for any reason other than radiologic disease progression by BICR evaluation. Every effort should be made to maintain the assessment scheduling relative to Cycle 1 Day 1 especially if there are dosing cycle interruptions due to toxicities. For all subjects, copies of radiologic images must be available for independent central radiology review as determined by the Sponsor Medical Monitor.

20 **MRI of Brain**: Gadolinium contrast-enhanced MRI must be used for assessment of CNS lesions at baseline (30 days prior to Cycle 1 Day 1). For subjects who are without documented disease progression, MRI scans are to be done at every 2 cycles (± 7 days): Screening, C3D1, C5D1, C7D1 and then every 2 cycles up to Cycle 19 and then every 3 cycles up to Cycle 37 and then every 4 cycles thereafter until documented progression of disease. Subject responses will be confirmed ≥ 4 weeks later after the initial documentation of response by the Investigator. For subjects who have documented disease progression but are still continuing repotrectinib post-RECIST progression, MRI scans of the brain are to be done according to local institutional standard of care. Disease progression must be confirmed by BICR before treatment discontinuation and whenever possible the Investigator should consult the Sponsor Medical Monitor prior to treatment discontinuation for any reason other than radiologic disease progression by BICR evaluation. For subjects discontinuing the study treatment prior to documented radiographic progression, tumor assessments should continue on schedule approximately every 2 cycles or at the current scan interval at the time of treatment discontinuation until radiographic evidence of disease progression, the start of a subsequent anticancer therapy, withdrawal of consent, or decision to no longer treat (eg, supportive care only), whichever is first. The end of treatment scan is optional ONLY if performed within the past 4 weeks.

21 **Bone Scans**: Bone scans (or bone MRI if preferred by Investigator) will be performed at baseline (30 days prior to Cycle 1 Day 1), if bone metastases are suspected. In addition, bone scans can be performed if clinically indicated per Investigator's discretion. These scans are only repeated for subjects having a baseline scan, to confirm the absence of bone metastases in case of a Complete Response (CR).

22 **EORTC-QLQ-C30 and QLQ-LC13**: Subjects must complete all EORTC-QLQ-C30 and EORTC-QLQ-LC13 self-assessment questionnaires in the clinic at the specified time points prior to dosing. At Cycle 1 Day 1, site staff (eg, site coordinators) should instruct subjects that the assessment should be completed without help from friends or family members and also recommend that this assessment be completed in the morning. All scheduled assessments of the EORTC-QLQ-C30 and EORTC-QLQ-LC13 cannot be taken home and must be completed in the clinic prior to any other study or medical procedure.

23 **Concomitant Medications and Non-Drug Supportive Interventions**: All concomitant medications and non-drug supportive interventions should be recorded in the CRF.

24 **Survival Follow-Up**: For subjects discontinuing the study treatment due to documented radiographic progression, obtain survival status via phone call or medical chart review, including information about subsequent anticancer therapies (including best response) every 3 months from the Safety Follow-up visit until death, loss of follow-up, or withdrawal of consent, whichever comes first.

*When PRs or CRs are observed, the responses will be confirmed ≥ 4 weeks later (ideally confirmation scans should be scheduled at end of the next cycle ie, 28 days) after the initial documentation of response.

**All effort should be made to obtain end of treatment scan

≠Safety Follow-Up: Subjects should be evaluated in clinic approximately 28 days after the last dose of study drug. Physical examination (including Lansky (< 16 years) or Karnofsky (≥ 16 to <18 years) or ECOG PS (≥18 years) and vitals) and clinical laboratory assessments should be performed as clinically indicated. Adverse events should be followed until all serious or study drug-related toxicities have resolved or are deemed “chronic” or “stable,” whichever is later.

¥ Patients on treatment after 1 year: Patients on treatment greater than 1 year (≥ 12 cycles), at the Investigator’s discretion, may have decreased study visits to align with scan and/or Echo/MUGA dates. Please note the following protocol requirements:

- Tumor assessments (scans) are to be performed at Screening (30 days prior to Cycle 1 Day 1), C3D1 (± 7 days), every 2 cycles (± 7 days) up to Cycle 19, every 3 cycles (± 7 days) up to Cycle 37, and then every 4 cycles (± 7 days) thereafter until documented progression of disease.
- ECHO or MUGA for LVEF assessment are to be performed at screening and after every 3 cycles of treatment (± 7 days) (Cycles 4, 7, 10, 13 and so on), and at the EOT visit (± 7 days).
- As the frequency of the scans and ECHO/MUGA do not align beyond Cycle 12 of treatment, once a subject is able to begin a reduced visit schedule, in order to ensure proper safety assessments are being conducted for all subjects, the following is provided as guidance on mandatory and optional visits.
  - Mandatory Visits : Cycle 13, Cycle 15, Cycle 16, Cycle 17, Cycle 19, Cycle 22, Cycle 25, Cycle 28, Cycle 31, Cycle 34, Cycle 37, Cycle 40, Cycle 41, Cycle 43, Cycle 45, Cycle 46, Cycle 49, Cycle 52, Cycle 53, Cycle 55, Cycle 57, Cycle 58, Cycle 61, Cycle 64, Cycle 65, Cycle 67, Cycle 69, Cycle 70, Cycle 73, Cycle 76, Cycle 77, Cycle 79, Cycle 81
  - Optional Visits : Cycle 12, Cycle 14, Cycle 18, Cycle 20, Cycle 21, Cycle 23, Cycle 24, Cycle 26, Cycle 27, Cycle 29, Cycle 30, Cycle 32, Cycle 33, Cycle 35, Cycle 36, Cycle 38, Cycle 39, Cycle 42, Cycle 44, Cycle 47, Cycle 48, Cycle 50, Cycle 51, Cycle 54, Cycle 56, Cycle 59, Cycle 60, Cycle 62, Cycle 63, Cycle 66, Cycle 68, Cycle 71, Cycle 72, Cycle 74, Cycle 75, Cycle 78, Cycle 80

Approved v2.0 930210036 2.0

9 SAFETY ASSESSMENTS

Safety assessments will consist of monitoring and recording AEs, including serious adverse events (SAEs), measurement of protocol-specified clinical laboratory assessments and vital signs, and other protocol-specified tests that are deemed critical to the safety evaluation of the study.

Certain types of events require immediate reporting to the Sponsor, as outlined in Section 9.3.

For country-specific requirements, please refer to APPENDIX 8.

9.1 Adverse Events

According to the ICH guideline for Good Clinical Practice, an adverse event is any untoward medical occurrence in a clinical investigation subject administered a medicinal product, regardless of causal attribution. An adverse event can therefore be any of the following:

- Any unfavorable and unintended sign (including any deterioration in a laboratory value or other clinical test [eg, ECG, X-ray] that is associated with symptoms or leads to a change in study treatment or concomitant treatment or discontinuation from study drug), symptom, or disease temporally associated with the use of a medicinal product, whether or not considered related to the medicinal product
- Any new disease or exacerbation of an existing disease (a worsening in the character, frequency, or severity of a known condition), except as described in Section 9.2.5.9
- Recurrence of an intermittent medical condition (eg, headache) not present at baseline
- Any deterioration in a laboratory value or other clinical test (eg, ECG, X-ray) that is associated with symptoms or leads to a change in study treatment or concomitant treatment or discontinuation from study drug
- Adverse events that are related to a protocol-mandated intervention, including those that occur prior to assignment of study treatment (eg, screening invasive procedures such as biopsies)

9.1.1 Serious Adverse Events (Immediately Reportable to the Sponsor)

An SAE is any adverse event that meets any of the following criteria:

- Fatal (ie, the adverse event actually causes or leads to death)
- Life-threatening (ie, the adverse event, in the view of the Investigator, places the subject at immediate risk of death)
- This does not include any adverse event that, had it occurred in a more severe form or was allowed to continue, might have caused death
- Requires or prolongs in-patient hospitalization (see Section 9.2.5.10)
- Results in persistent or significant disability/incapacity (ie, the adverse event results in substantial disruption of the subject's ability to conduct normal life functions)
- Congenital anomaly/birth defect in a neonate/infant born to a mother exposed to study drug
- Significant medical event in the Investigator's judgment (eg, may jeopardize the subject or may require medical/surgical intervention to prevent one of the outcomes listed above)

The terms "severe" and "serious" are **not** synonymous. Severity refers to the intensity of an adverse event (eg, rated as mild, moderate, or severe, or according to CTCAE; see Section 9.2.3); the event itself may be of relatively minor medical significance (such as severe headache without any further findings).

Severity and seriousness need to be independently assessed for each adverse event recorded on the CRF.

Serious adverse events (SAEs) are required to be reported by the Investigator to the Sponsor Medical Monitor immediately (ie, no more than 24 hours after learning of the event; see Section 9.3 for reporting instructions).

9.2 Methods and Timing for Capturing and Assessing Safety Parameters

The Investigator is responsible for ensuring that all AEs are recorded on the Adverse Event CRF and reported to the Sponsor.

For each adverse event recorded on the Adverse Event CRF, the Investigator will make an assessment of seriousness (see Section 9.1.1 for seriousness criteria), severity (see Section 9.2.3), and causality (see Section 9.2.4).

9.2.1 Adverse Event Reporting Period

For country-specific requirements, please refer to APPENDIX 8.

Investigators will seek information on AEs at each subject contact. All AEs, whether reported by the subject or noted by study personnel, will be recorded in the subject's medical record and on the Adverse Event CRF.

After informed consent for the clinical trial has been obtained but prior to initiation of study drug, only SAEs related to protocol-mandated assessments should be reported.

After initiation of study drug, all AEs, regardless of relationship to study drug, will be reported until at least 28 days after the last dose of study treatment. The investigator must report any SAE that occurs after these periods and that is believed to be related to a study intervention or protocol-specified procedure (eg, a follow-up skin biopsy).

9.2.2 Eliciting Adverse Event Information

A consistent methodology of non-directive questioning should be adopted for eliciting adverse event information at all subject evaluation time points. Examples of non-directive questions include the following:

"How have you been feeling since your last clinic visit?"

"Have you noticed any new or changed health problems since you were last here?"

9.2.3 Assessment of Severity of Adverse Events

The adverse event severity grading scale for the CTCAE v4.03 will be used for assessing adverse event severity. Table 27 will be used for assessing severity for AEs that are not specifically listed in the CTCAE.

Table 27: Grading of Severity of Adverse Events

Grade	Severity
1	Mild; asymptomatic or mild symptoms; clinical or diagnostic observations only; or intervention not indicated
2	Moderate; minimal, local, or non-invasive intervention indicated; or limiting age-appropriate instrumental activities of daily living [a]
3	Severe or medically significant, but not immediately life-threatening; hospitalization or prolongation of hospitalization indicated; disabling; or limiting self-care activities of daily living [b, c]
4	Life-threatening consequences or urgent intervention indicated [d]
5	Death related to adverse event [d]

[a] Instrumental activities of daily living refer to preparing meals, shopping for groceries or clothes, using the telephone, managing money, etc.

[b] Examples of self-care activities of daily living include bathing, dressing and undressing, feeding oneself, using the toilet, and taking medications, as performed by subjects who are not bedridden.

[c] If an event is assessed as a "significant medical event," it must be reported as a SAE per the definition of SAE.

[d] Grade 4 and 5 events must be reported as SAEs per the definition of SAE.

9.2.4 Assessment of Causality of Adverse Events

Investigators should use their knowledge of the subject, the circumstances surrounding the event, and an evaluation of any potential alternative causes to determine whether or not an adverse event is considered to be related to the study drug, indicating "yes" or "no" accordingly. The following guidance should be taken into consideration (see also Table 28):

- Temporal relationship of event onset to the initiation of study drug
- Course of the event, considering especially the effects of dose reduction, discontinuation of study drug, or reintroduction of study drug (as applicable)
- Known association of the event with the study drug or with similar treatments
- Known association of the event with the disease under study
- Presence of risk factors in the subject or use of concomitant medications known to increase the occurrence of the event
- Presence of non-treatment-related factors that are known to be associated with the occurrence of the event

Table 28: Causation Attribution

Is the adverse event suspected to be caused by the study drug on the basis of facts, evidence, science-based rationales, and clinical judgment?	
YES	There is a plausible temporal relationship between the onset of the adverse event and administration of the study drug, and the adverse event cannot be readily explained by the subject's clinical state, intercurrent illness, or concomitant therapies; and/or the adverse event follows a known pattern of response to the study drug; and/or the adverse event abates or resolves upon discontinuation of the study drug or dose reduction and, if applicable, reappears upon re-challenge.
NO	Evidence exists that the adverse event has an etiology other than the study drug (eg, preexisting medical condition, underlying disease, intercurrent illness, or concomitant medication); and/or the adverse event has no plausible temporal relationship to administration of the study drug (eg, cancer diagnosed 2 days after first dose of study drug).

9.2.5 Procedures for Recording Adverse Events

Investigators should use correct medical terminology/concepts when recording AEs on the Adverse Event CRF. Avoid colloquialisms and abbreviations. Only one adverse event term should be recorded in the event field on the Adverse Event CRF.

9.2.5.1 Diagnosis versus Signs and Symptoms

A diagnosis (if known) should be recorded on the Adverse Event CRF rather than individual signs and symptoms (eg, record only liver failure or hepatitis rather than jaundice, asterixis, and elevated transaminases). However, if a constellation of signs and/or symptoms cannot be medically characterized as a single diagnosis or syndrome at the time of reporting, each individual event should be recorded on the Adverse Event CRF. If a diagnosis is subsequently established, all previously reported AEs based on signs and symptoms should be nullified and replaced by one adverse event report based on the single diagnosis, with a starting date that corresponds to the starting date of the first symptom of the eventual diagnosis.

9.2.5.2 Adverse Events That Are Secondary to Other Events

In general, AEs that are secondary to other events (eg, cascade events or clinical sequelae) should be identified by their primary cause, with the exception of severe or serious secondary events. A medically significant secondary adverse event that is separated in time from the initiating event should be recorded as an independent event on the Adverse Event CRF. For example:

- If vomiting results in mild dehydration with no additional treatment in a healthy adult, then only vomiting should be reported on the CRF
- If vomiting results in severe dehydration, then both events should be reported separately on the CRF
- If a severe gastrointestinal hemorrhage leads to renal failure, then both events should be reported separately on the CRF
- If dizziness leads to a fall and consequent fracture, then all 3 events should be reported separately on the CRF

- If neutropenia is accompanied by an infection, then both events should be reported separately on the CRF

All AEs should be recorded separately on the Adverse Event CRF if it is unclear as to whether the events are associated.

9.2.5.3 Persistent or Recurrent Adverse Events

A persistent adverse event is one that extends continuously, without resolution, across cycles. When this occurs, the initial severity (intensity or grade) of the event will be recorded at the time the event is first reported. If a persistent adverse event becomes more or less severe, the initial event should be closed and a new event with the new severity should be recorded on the Adverse Event CRF. Similarly, if the event becomes serious, the initial event should be closed and a new event recorded on the Adverse Event CRF, completing all data fields related to SAEs. The SAE should be reported to the Sponsor Medical Monitor immediately (ie, no more than 24 hours after learning that the event became serious).

A recurrent adverse event is one that resolves between treatment cycles and subsequently recurs. Each recurrence of an adverse event should be recorded as a separate event on the Adverse Event CRF.

9.2.5.4 Abnormal Laboratory Values

Not every laboratory abnormality qualifies as an adverse event. A laboratory test result must be reported as an adverse event if it meets any of the following criteria:

- Accompanied by clinical symptoms
- Results in a change in study treatment (eg, dosage modification, treatment interruption, or treatment discontinuation)
- Results in a medical intervention (eg, potassium supplementation for hypokalemia) or a change in concomitant therapy
- Results in more frequent follow-up assessments or further diagnostic investigation
- Clinically significant in the Investigator's judgment

It is the Investigator's responsibility to review all laboratory findings. Medical and scientific judgment should be exercised in deciding whether an isolated laboratory abnormality should be classified as an adverse event.

If a clinically significant laboratory abnormality is a sign of a disease or syndrome (eg, increased alkaline phosphatase and bilirubin at 5 × ULN associated with cholestasis), only the diagnosis (ie, cholestasis) should be recorded on the Adverse Event CRF.

If a clinically significant laboratory abnormality is not a sign of a disease or syndrome, the abnormality itself should be recorded on the Adverse Event CRF, along with a descriptor indicating if the test result is above or below the normal range (eg, "elevated potassium," as opposed to "abnormal potassium"). If the laboratory abnormality can be characterized by a precise

clinical term per standard definitions, the clinical term should be recorded as the adverse event. For example, an elevated serum potassium level of 7.0 mEq/L should be recorded as "hyperkalemia."

Observations of the same clinically significant laboratory abnormality from visit to visit should not be repeatedly recorded on the Adverse Event CRF, unless the etiology changes. The initial severity of the event should be recorded, and the severity or seriousness should be updated as described in Section 9.2.5.3.

9.2.5.5 *Abnormal Vital Sign Values*

Not every vital sign abnormality qualifies as an adverse event. A vital sign result must be reported as an adverse event if it meets any of the following criteria:

- Accompanied by clinical symptoms
- Results in a change in study treatment (eg, dosage modification, treatment interruption, or treatment discontinuation)
- Results in a medical intervention or a change in concomitant therapy
- Clinically significant in the Investigator's judgment

It is the Investigator's responsibility to review all vital sign findings. Medical and scientific judgment should be exercised in deciding whether an isolated vital sign abnormality should be classified as an adverse event.

If a clinically significant vital sign abnormality is a sign of a disease or syndrome (eg, high blood pressure), only the diagnosis (ie, hypertension) should be recorded on the Adverse Event CRF.

Observations of the same clinically significant vital sign abnormality from visit to visit should not be repeatedly recorded on the Adverse Event CRF, unless the etiology changes. The initial severity of the event should be recorded, and the severity or seriousness should be updated as described in Section 9.2.5.3.

9.2.5.6 *Abnormal Liver Function Tests*

Abnormal values in aspartate transaminase (AST) and/or alanine transaminase (ALT) levels concurrent with abnormal elevations in total bilirubin level that meet the criteria outlined below in the absence of other causes of liver injury are considered potential cases of drug-induced liver injury (potential Hy's Law cases) and should always be considered important medical events.

The threshold of laboratory abnormalities for a potential case of drug-induced liver injury depends on the subject's individual baseline values and underlying conditions. Subjects who present with the following laboratory abnormalities should be evaluated further to definitively determine the etiology of the abnormal laboratory values:

- Subjects with AST or ALT and total bilirubin baseline values within the normal range who subsequently present with AST or ALT values > 3× ULN concurrent with a total bilirubin value > 2 × ULN with no evidence of hemolysis and an alkaline phosphatase value ≤ 2 × ULN or not available.

- For subjects with preexisting ALT OR AST OR total bilirubin values above the ULN, the following threshold values should be used in the definition mentioned above:
  - For subjects with preexisting AST or ALT baseline values above the normal range, AST or ALT value ≥ 2 times the baseline values and ≥ 3 × ULN, or ≥ 8 × ULN (whichever is smaller).
- Concurrent with
  - For subjects with preexisting values of total bilirubin above the normal range: Total bilirubin increased from baseline by an amount of ≥ 1 × ULN or if the value reaches ≥ 3 × ULN (whichever is smaller).

The subject should return to the investigational site and be evaluated as soon as possible, preferably within 48 hours from awareness of the abnormal results. This evaluation should include laboratory tests, detailed history and physical assessment. The possibility of hepatic neoplasia (primary or secondary) should be considered. In addition to repeating measurements of AST and ALT, laboratory tests should include albumin, creatine kinase, total bilirubin, direct and indirect bilirubin, gamma-glutamyl transferase, prothrombin time (PT)/international normalized ratio (INR), and alkaline phosphatase. A detailed history, including relevant information, such as review of ethanol, acetaminophen, recreational drug and supplement consumption, family history, occupational exposure, sexual history, travel history, history of contact with a jaundiced person, surgery, blood transfusion, history of liver or allergic disease, and work exposure, should be collected. Further testing for acute hepatitis A, B, or C infection and liver imaging (eg, biliary tract) may be warranted. All cases confirmed on repeat testing as meeting the laboratory criteria defined above, with no other cause for liver function test (LFT) abnormalities identified at the time should be considered potential Hy's Law cases irrespective of availability of all the results of the investigations performed to determine etiology of the abnormal LFTs. Such potential Hy's Law cases should be reported as SAEs.

9.2.5.7 Deaths

In Phase 1, all deaths, regardless of the causality will be considered an SAE and will be reported as such if they occur within 28-day safety period.

In Phase 2, death due to disease progression will not be considered an SAE.

All deaths, regardless of relationship to study drug, must be recorded on the Death CRF and on the Adverse Event CRF only if it is considered related to an adverse event.

Death should be considered an outcome and not a distinct event. The event or condition that caused or contributed to the fatal outcome should be recorded as the single medical concept on the Adverse Event CRF. Generally, only one such event should be reported. The term "**sudden death**" should be used only for the occurrence of an abrupt and unexpected death due to presumed cardiac causes in a subject with or without preexisting heart disease, within 1 hour after the onset of acute symptoms or, in the case of an unwitnessed death, within 24 hours after the subject was last seen alive and stable. If the cause of death is unknown and cannot be ascertained at the time of reporting, "**unexplained death**" should be recorded on the Adverse Event CRF. If the cause of

death later becomes available (eg, after autopsy), "unexplained death" should be replaced by the established cause of death.

9.2.5.8 Preexisting Medical Conditions

A preexisting medical condition is one that is present at the screening visit for this study. Such conditions should be recorded on the Medical History CRF.

A preexisting medical condition should be recorded as an adverse event only if the frequency, severity, or character of the condition worsens during the study. When recording such events on the Adverse Event CRF, it is important to convey the concept that the preexisting condition has changed by including applicable descriptors (eg, "worsening headaches").

9.2.5.9 Lack of Efficacy or Worsening of the Malignancy under Study.

Events that are clearly consistent with the expected pattern of progression of the underlying disease should not be recorded as AEs. These data will be captured as efficacy assessment data only. In most cases, the expected pattern of progression will be reflected as objective radiographic progression according to RECIST Version 1.1 criteria. In rare cases, the determination of disease progression will be based predominantly on symptomatic deterioration. However, every effort should be made to document progression through use of objective criteria. If there is any uncertainty as to whether an event is due to disease progression, it should be reported as an adverse event.

9.2.5.10 Hospitalization or Prolonged Hospitalization

Any adverse event that results in hospitalization (ie, in-patient admission to a hospital) or prolonged hospitalization should be documented and reported as a SAE, except as outlined below.

The following hospitalization scenarios are not considered to be adverse events:

- Hospitalization for respite care or social admissions (eg, lack of housing, economic inadequacy, family circumstances)
- Hospitalization solely for coordination of care, including hospice arrangements
- Hospitalization due solely to progression of the underlying cancer
- Hospitalization that was necessary solely because of subject requirement for outpatient care outside of normal outpatient clinic operating hours
- Hospitalization for same day surgeries (as outpatient/same day/ambulatory procedures)
- Planned hospitalization required by the protocol (eg, for study drug administration or insertion of access device for study drug administration)
- Hospitalization for a preexisting condition, provided that the hospitalization was planned prior to the study or was scheduled during the study when elective surgery became necessary because of the expected normal progression of the disease and the subject has not experienced an adverse event

9.2.5.11 Occupational Exposure

An occupational exposure occurs when during the performance of job duties, a person (whether a healthcare professional or otherwise) has unplanned direct contact with the product, which may or may not lead to the occurrence of an adverse event.

An occupational exposure is reported to safety within 24 hours of Investigator's awareness, using the SAE Report form, regardless of whether there is an associated AE/SAE. Since the information does not pertain to a subject enrolled in the study, the information is not reported on a CRF, however a copy of the completed SAE Report form is maintained in the study master file.

9.2.5.12 Adverse Events Associated with an Overdose or Incorrect Administration of Study Drug

An overdose is the accidental or intentional use of a drug in an amount higher than the dose being studied. An overdose or incorrect administration of study treatment is not itself an adverse event, but it may result in an adverse event. All AEs associated with an overdose or incorrect administration of study drug should be recorded on the Adverse Event CRF. If the associated adverse event fulfills serious criteria, the event should be reported to the Sponsor Medical Monitor immediately (ie, no more than 24 hours after learning of the event).

9.3 Immediate Reporting Requirements from Investigator to the Sponsor

Certain events require immediate reporting to allow the Sponsor to take appropriate measures to address potential new risks in a clinical trial. The Investigator must report such events to the Sponsor immediately; under no circumstances should reporting take place more than 24 hours after the Investigator learns of the event. The following is a list of events that the Investigator must report to the Sponsor within 24 hours after learning of the event, regardless of relationship to study drug:

- SAEs
- Pregnancies

The Investigator must report new significant follow-up information for these events to the Sponsor immediately (ie, no more than 24 hours after becoming aware of the information). New significant information includes the following:

- New signs or symptoms or a change in the diagnosis
- Significant new diagnostic test results
- Change in causality based on new information
- Change in the event's outcome, including recovery
- Additional narrative information on the clinical course of the event

Investigators must also comply with local requirements for reporting SAEs to the local health authority and IRB/EC.

9.3.1 SAE Reporting

For country-specific requirements, please refer to APPENDIX 8.

After informed consent has been obtained but prior to initiation of study drug, only SAEs related to protocol-mandated procedures should be reported.

After initiation of study drug, SAEs will be reported until at least 28 days after the last dose of study drug.

Investigators should record all case details that can be gathered immediately (ie, within 24 hours after learning of the event) on the SAE report form and submit the report via fax or email (please refer to the Study Manual).

9.3.2 Pregnancy

This section is not applicable for women not of childbearing potential.

If, following initiation of the study intervention, it is discovered that a participant is pregnant or may have been pregnant at the time of study intervention exposure, including at least 2 months after study intervention administration, the investigator must immediately notify the Sponsor Medical Monitor/designee of this event and complete and forward a Pregnancy Surveillance Form to the Sponsor designee within 24 hours of awareness of the event and in accordance with SAE reporting procedures described in Section 9.3.1.

In all cases, the study intervention will be discontinued in an appropriate manner (eg, dose tapering if necessary for participant safety).

Follow-up information regarding the course of the pregnancy, including perinatal and neonatal outcome and, where applicable, offspring information, must be reported on the Pregnancy Surveillance Form. Protocol-required procedures for study discontinuation and follow-up must be performed.

Any pregnancy that occurs in a female partner of a male participant at the time of study intervention exposure, including at least 4 months after study intervention administration, should be reported to the Sponsor or designee. For the Sponsor or designee to collect any pregnancy surveillance information from the female partner, the female partner must sign an ICF for disclosure of this information. Information on this pregnancy will be collected and recorded on the Pregnancy Surveillance Form.

If any sexual activity involving penile intercourse (eg, vaginal, anal, oral) has occurred between a male participant and a pregnant partner(s) without the use of a latex condom during and for at least 4 months after study intervention administration, the information should be reported to the Sponsor or designee, even if the male participant has undergone a successful vasectomy.

9.4 Follow-Up of Subjects After Adverse Events

9.4.1 Investigator Follow-Up

The Investigator should follow each adverse event until the event has resolved to baseline grade or better, the event is assessed as stable by the Investigator, the subject is lost to follow-up, or the

subject withdraws consent. Every effort should be made to follow all SAEs considered to be related to study drug or trial-related procedures until a final outcome can be reported.

During the study period, resolution of AEs (with dates) should be documented on the Adverse Event CRF and in the subject's medical record to facilitate source data verification.

All pregnancies reported during the study should be followed until pregnancy outcome.

9.4.2 Sponsor Follow-Up

For SAEs and pregnancies, the Sponsor or a designee may follow-up by telephone, fax, electronic mail, and/or a monitoring visit to obtain additional case details and outcome information (eg, from hospital discharge summaries, consultant reports, autopsy reports) in order to perform an independent medical assessment of the reported case.

9.5 Post-Study Adverse Events

For country-specific requirements, please refer to APPENDIX 8.

The Sponsor should be notified if the Investigator becomes aware of any SAE that occurs after the end of the adverse event reporting period (defined as 28 days after the last dose of study drug), if the event is believed to be related to the study drug.

The Investigator should report these events directly to the Sponsor or its designee using the SAE report form.

9.6 Expedited Reporting to Health Authorities, Investigators, Institutional Review Boards, and Ethics Committees

The Sponsor will promptly evaluate all SAEs against cumulative product experience to identify and expeditiously communicate possible new safety findings to Investigators, IRBs, Ecs, and applicable health authorities based on applicable legislation.

To determine reporting requirements for single adverse event cases, the Sponsor will assess the expectedness of these events using the Investigator's Brochure.

The Sponsor will compare the severity of each event and the cumulative event frequency reported for the study with the severity and frequency reported in the applicable reference document.

Reporting requirements will also be based on the Investigator's assessment of causality and seriousness, with allowance for upgrading by the Sponsor as needed.

10 SUBJECT END OF TREATMENT

A subject may be discontinued from study treatment at any time if the subject, the Investigator, or the Sponsor Medical Monitor determine that it is not in the subject's best interest to continue on study. The following is a list of possible reasons for early discontinuation of study treatment:

- Disease progression (unless there is reasonable evidence of clinical benefit to justify continuation on treatment – which must be previously discussed with the Sponsor)
  - Subjects should remain on study treatment until there is BICR-confirmed radiographic progression

  - Subject may be allowed to continue repotrectinib beyond RECIST-defined progression if, in the clinical judgment of the Investigator, the subject continues to derive clinical benefit from repotrectinib (with approval from the Sponsor)
  - There is no limit to the number of cycles of treatment with repotrectinib
- Any adverse event that cannot be adequately managed with dose modifications, including dose interruption > 28 days (unless there is reasonable evidence of clinical benefit to justify continuation on the protocol – which must be previously discussed with the Sponsor)
- Protocol violation requiring discontinuation of study treatment
- Subject is not compliant with study procedures
- Lost to follow-up
- Subject withdrawal of consent for further treatment
- Sponsor's early termination of study. Reasons for terminating the study may include, but are not limited to, the following:
  - All enrolled subjects have discontinued study treatment
  - The incidence or severity of AEs in this or other studies indicates a potential health hazard to subjects
  - Subject enrollment is unsatisfactory

Data to be collected for the EOT visit are described in Section 8.5.

Subjects will be followed for at least 28 calendar days after the last dose of study drug. If a subject is withdrawn from treatment due to an adverse event, the subject will be followed until the adverse event has resolved or stabilized as per Section 9.4.

11 PROTOCOL DEVIATIONS

A protocol deviation occurs when the subject or Investigator fails to adhere to significant protocol requirements affecting the inclusion, exclusion, subject safety, or primary endpoint criteria. Protocol deviations for this study include, but are not limited to, the following:

- Failure to meet inclusion/exclusion criteria (no waivers will be granted to meet the eligibility criteria)
- Dose modifications (eg, wrong treatment or incorrect dose) that are not within the protocol specifications
- Use of a prohibited concomitant medication
- Any other deviation that presents significant risk or safety concerns to the subject (e.g, pregnancy on study)

Failure to comply with Good Clinical Practice (GCP) guidelines will also result in a protocol deviation. The Sponsor, in consultation with the Investigator, will determine if a protocol deviation should result in withdrawal of a subject.

12 DATA MONITORING COMMITTEE

For the Phase 1 portion of the study, safety will be overseen by the CSC comprised of all Investigators and the Medical Monitor of the Study.

An independent Data Monitoring Committee (DMC) will be established to ensure the overall integrity and conduct of the Phase 2 Dose Expansion portion of the study.

The DMC will review the progress of the study and cumulative clinical data on a periodic basis (eg, approximately 4 review meetings per year).

Following each review meeting, the DMC will recommend to the Sponsor whether to continue the trial unchanged, modify the conduct of the study, terminate a study cohort early, or terminate the study early with the final decision by the Sponsor. Rules for early termination, modifications, and/or continuation of the study, as well as how those recommendations will be made to the Sponsor or Health Authorities will be outlined in a separate DMC Charter.

The DMC will be composed of 3 external members (2 physicians and 1 biostatistician) not associated with the conduct of the study. Sponsor's biostatistician and data management team (or their designee) will prepare and provide study data to the DMC. Complete details regarding the composition and governance of the DMC will be outlined in the DMC Charter. Periodic adverse event data review will also be performed by designated members of Sponsor's study team or their designee. Any safety issues of concern identified by the study team will be promptly reported to the DMC as described in the DMC Charter.

For IEC/IRB information, refer to Section 15.2.

13 STATISTICAL METHODS AND CONSIDERATIONS

Detailed methodology for summary and statistical analyses of the data collected in this trial will be documented in a Statistical Analysis Plan (SAP), which will be maintained by the Sponsor or their designee. There will be two SAPs for this protocol; one for Phase 1 and one for Phase 2. The Phase 2 SAP may include analyses where eligible Phase 1 subjects are pooled with the Phase 2 subjects. These documents may modify the initial analysis plans outlined in the protocol; however, any major modifications of the primary endpoint and/or its analysis will also be reflected in a protocol amendment.

13.1 Analysis Populations (Sets)

All the analyses will be performed by cohort, except when it is necessary to pool them together. Data for all subjects will be assessed to determine if subjects meet the criteria for inclusion in each analysis population (set) prior to locking the database.

13.1.1 Full Analysis Set

The Full Analysis Set (FAS) consists of all subjects who receive at least 1 full or partial dose of repotrectinib. This will be the same as the safety analysis set. Subjects will be classified according to the assigned treatment (dose cohort for Phase 1 and study cohort for Phase 2).

For Phase 1, The FAS set will be used for the summary of subject dispositions, demographics, baseline characteristics, and utilized for the safety analysis.

For Phase 2, the FAS set will be used for the summary of subject dispositions, demographics, baseline characteristics, and utilized for the safety and primary efficacy analysis.

Subjects who were screened but never started treatment will not be in the FAS and will be listed in the subject data listing as a screen failure including their reason for screen failure. Screening failures will not be included in any of the summary tables.

13.1.2 Safety Analysis Set

The safety analysis set includes all enrolled subjects who receive at least 1 dose of repotrectinib. This will be the same as the Full Analysis Set. Subjects will be classified according to assigned treatment (dose cohort for Phase 1 and study cohort for Phase 2). Each subject will be classified into and analyzed consistently within one treatment group.

13.1.3 Per Protocol Analysis Set (Phase 1)

- Evaluable for MTD: All enrolled subjects who receive at least 75% of the planned repotrectinib doses in the first cycle. Subjects who receive less than 75% of the planned repotrectinib doses in the first cycle due to DLT are also considered evaluable for MTD.
- Evaluable for Response: All enrolled subjects who receive study treatment, have a baseline tumor assessment with documentation of measurable disease, have at least 1 on study tumor assessment, and have no major protocol violations including inclusion/exclusion criteria or prohibited concomitant medication while on study, will be considered evaluable for response. In addition, if a subject is enrolled without central lab confirmation of the specific gene alteration, and then is subsequently found not to have the specific gene alteration by central review, this subject will not be included in the efficacy evaluable (evaluable for response) population.
- Evaluable for CNS Metastasis Response: A subset of the evaluable for response analysis set will be used to include only those subjects with measurable brain metastasis at baseline for tumor assessment set with CNS metastases at study entry.

13.1.4 PK Concentration Analysis Set

13.1.4.1 Repotrectinib

The PK concentration analysis set for repotrectinib is defined as all enrolled subjects who receive at least 1 dose of repotrectinib and have at least 1 concentration of repotrectinib.

13.1.4.2 Midazolam (MDZ DDI sub-study only)

The midazolam PK concentration analysis set is defined as all patients treated with midazolam and who have at least 1 concentration of midazolam.

13.1.5 PK Parameter Analysis Set

13.1.5.1 Repotrectinib

The repotrectinib PK parameter analysis set is defined as all enrolled subjects who receive at least 1 dose of repotrectinib and have sufficient information to estimate at least 1 of the PK parameters of interest for repotrectinib.

13.1.5.2 Midazolam (MDZ DDI sub-study only)

The midazolam PK parameter analysis set is defined as all enrolled patients have received at least 1 dose of midazolam and have sufficient information to estimate at least 1 midazolam PK parameters of interest (C_{max} or AUC).

13.2 Efficacy Analyses

Efficacy data for primary and secondary endpoints will be based on the radiologic assessments evaluated by the BICR. Data from the Investigator will be analyzed as supportive.

13.2.1 Analysis of Primary Endpoint

13.2.1.1 Analysis of Primary Endpoint (Phase 1)

The primary endpoint for the Phase 1 study is to determine the MTD and RP2D of repotrectinib.

13.2.1.2 Analysis of Primary Endpoint (Phase 2)

The primary endpoint for the Phase 2 study is ORR, as assessed by BICR using RECIST 1.1, in each subject population expansion cohort of solid tumors that harbor a *ROS1*, *NTRK1*, *NTRK2*, or *NTRK3* gene rearrangement.

The ORR will be defined as the proportion of subjects with a confirmed complete response (CR) or partial response (PR). A confirmed response is a response that persists on a repeat-imaging performed at least 4 weeks after initial documentation of response. Subjects with a confirmed objective response (CR or PR) will be referred to as responders. Non-responders will include subjects without a confirmed objective response, SD, or PD.

The ORR will be reported as the proportion of responders along with the corresponding 2-sided 95% Clopper-Pearson exact CI.

Subgroup analyses of primary endpoint ORR may be performed by demographic and baseline risk factors in each expansion cohort if data permit. Below lists are the major subgroups to be considered:

- Age: ≥65 vs. <65 years old
- Sex: Male vs. Female
- Race: Caucasian vs. non-Caucasian
- Performance status
- Brain metastases at baseline: Yes vs. No
- Prior TKI : Crizotinib vs. Non-Crizotinib (Cohort 2)
- Prior chemotherapy: Yes vs. No
- Prior lines of systemic therapies: ≤ 2 vs. > 2

13.2.2 Analysis of Secondary Endpoints

13.2.2.1 Analysis of Secondary Endpoints (Phase 1)

The secondary endpoint for the phase 1 study is ORR, as assessed by BICR using RECIST version 1.1, in each subject population expansion cohort of solid tumors that harbor an *ALK*, *ROS1*, *NTRK1*, *NTRK2*, or *NTRK3* gene rearrangement.

The ORR will be defined as the proportion of subjects with confirmed CR or PR; a confirmed response is a response that persists on repeat-imaging ≥ 4 weeks after initial documentation of response. Such subjects with a confirmed objective response (CR or PR) will be referred to as responders. Non-responders will include the following:

- Subjects without a confirmed objective response
- Subjects without a post-baseline tumor assessment
- Subjects who receive at least 1 dose of repotrectinib and who discontinue for any reason prior to undergoing 1 post-baseline response evaluation

The ORR will be reported as the proportion of responders along with the corresponding 2-sided 95% Clopper-Pearson exact CI.

13.2.2.2 Analysis of Secondary Endpoints (Phase 2)

Duration of Response

The DOR will be defined from the first date of objective response (either CR or PR) to first documentation of radiographic disease progression or death, as assessed by RECIST Version 1.1. The DOR will be censored at the last tumor assessment date for subjects without disease progression.

The DOR will only be calculated for the subgroup of subjects with a confirmed objective tumor response. The DOR will be summarized in the populations of subjects with a confirmed CR or PR using the Kaplan-Meier method and will be displayed graphically where appropriate. The median event time (if appropriate) and 2-sided 95% CI for the median will be provided. The number and % of subjects with actual DOR ≥ 6, 12, and 18 months will also be tabulated.

Time to Response

The TTR will be defined as the time from the first dose of repotrectinib to the first documentation of objective response (either CR or PR), as assessed by RECIST Version 1.1. Descriptive statistics (n, mean, median, standard deviation, minimum, and maximum) will be presented for TTR.

Clinical Benefit Rate

The CBR will be defined as the proportion of subjects with CR, PR, or SD. Stable disease refers to a condition where the tumor is neither increasing nor decreasing in extent or severity for at least 6 weeks after the first dose of repotrectinib, as assessed by RECIST Version 1.1. The CBR and its 95% CI will be estimated using Clopper-Pearson exact confidence interval.

Intracranial Objective Response Rate

Intracranial ORR is defined as the percent of subjects with PR/CR based on the assessment of intracranial target lesions, non-target lesions, and new lesions in subjects with measurable CNS metastasis at baseline. The IC-ORR and its 95% CI will be estimated using the similar methods as for ORR. The modified RECIST Version 1.1 criteria may be utilized to assess IC-ORR.

For Phase 1, Intracranial ORR will be calculated pooling the subjects from all of the dose escalation cohorts in the per protocol analysis set, evaluable for CNS metastasis response subset.

CNS Progression-Free Survival

CNS progression-free survival (CNS-PFS) will be defined as the time from the first dose of repotrectinib to first evidence of radiographic CNS disease progression or death due to any cause (whichever occurs first) + 1 day. Modified RECIST Version 1.1 criteria may be utilized to assess CNS-PFS. Censoring rules will apply as for PFS per below.

Progression-Free Survival

Progression-free survival (PFS) will be defined as the time from the first dose of repotrectinib to first documentation of radiographic disease progression by BICR using RECIST Version 1.1 or death due to any cause (whichever occurs first).

For subjects discontinuing study treatment prior to documented radiographic progression, tumor assessments should continue on schedule approximately every 2 cycles or at the current scan interval at the time of treatment discontinuation until radiographic evidence of disease progression, the start of a subsequent anticancer therapy, withdrawal of consent, or decision to no longer treat (eg, supportive care only), whichever is first.

If subjects have at least 1 on study disease assessment, PFS data will be censored on the date of the last evaluable tumor assessment documenting absence of progressive disease for subjects:

- Subjects without progression or death will be censored on the date of the last tumor assessment (or, if no tumor assessment was performed after the baseline visit, at the date of first dose of study drug + 1 day).
- Subjects who start a subsequent anticancer therapy in the absence of PD or death will be censored at the last evaluable assessment prior to the start of the new anti-tumor treatment.
- For those with 2 or more missing consecutive radiologic assessments, it will be censored at the last radiologic assessment prior to the missing assessments.

Estimates of the PFS curves using the Kaplan-Meier method will be presented. This method will be applied to derive the median event time and it's 95% CI as well as the 1-year and 18-month survival probabilities with related 95% Cis.

Overall Survival

OS is defined as the time from the first dose of repotrectinib to the date of death due to any cause. OS final analysis will occur approximately five years after the last patient initiates treatment. For subjects still alive at the time of analysis, the OS time will be censored on the last date the subjects

were known to be alive. For subjects who are lost to follow-up, OS will be censored at the last date the subject is known as alive before being lost to follow-up.

Estimates of the OS curves using the Kaplan-Meier method will be presented. This method will be applied to derive the median event time and a 95% CI for the median as well as the 1-year and 18-month survival probabilities with related 95% CIs.

13.3 Statistical Analysis Plan (SAP) and Sample Size Justification

13.3.1 Phase 1 Dose Escalation and Food Effect Cohorts

13.3.1.1 Phase 1a Dose Escalation

For the Dose Escalation phase of the study, cohorts of 3-6 evaluable subjects will be required. The total number of subjects will depend upon the number of dose escalations necessary.

Six total dose levels were evaluated (40 mg, 80 mg, 160 mg, 240 mg, 320 mg, and 400 mg/day). If MTD is not reached at the highest dose level, then assuming 6 subjects at each of the 6 dose levels and expansion to 12 subjects was permitted at the potential RP2D, a maximum of 42 subjects are anticipated to be enrolled.

13.3.1.2 Phase 1b Food Effect Study

The food effect study commenced at the 40 mg QD dose level where a subject experienced a PR as per Investigator. Six subjects were enrolled at that dose level and subsequent dose levels. An additional 6 subjects enrolled into the food effect study at the RP2D which was assumed to be 160 mg QD. Thus, the minimum number of subjects to enroll into the food effect study will be 12 subjects.

13.3.1.3 Phase 1c Dose Escalation with Food

For the Phase 1c dose escalation with food phase of the study, cohorts of 3-6 evaluable subjects will be required. The total number of subjects will depend upon the number of DLTs (if any), and if preliminary efficacy is seen within that cohort.

Up to 5 total dose levels will be evaluated (120 mg QD, 160 mg QD, 160 mg QD for 7 days followed by 160 mg BID, 120 mg QD for 7 days followed by 120 mg BID and 120 mg QD for 7 days followed by 240 mg QD). Assuming a maximum of 6 subjects at each of the 5 dose levels, a maximum of 30 subjects are anticipated to be enrolled.

13.3.2 Phase 2 Study

The primary objective for each of the six Phase 2 expansion cohorts is to determine the objective response rate (ORR) by BICR and their corresponding exact 95% confidence intervals. Confidence intervals will be used for inference purposes.

Below are the sample size justifications for each of the 6 cohorts.

13.3.2.1 EXP-1 ROS1 TKI-Naïve ROS1+ NSCLC

Fifty-five ROS1 TKI-naïve eligible *ROS1*+ NSCLC subjects will be enrolled into the ROS1i-naïve *ROS1*+ NSCLC subject expansion cohort. For the ROS1i-naïve expansion cohort, if the ORR is 66% or less, then it is assumed that repotrectinib is not adequately effective. Using Table 29, if

44 out of 55 subjects have a confirmed objective response (ORR = 80%; 95% CI: 67.0 – 89.6) where the lower limit of the 95% CI is > 66%, repotrectinib is considered to be superior than the currently approved therapy in this subject expansion cohort, crizotinib (Xalkori® USPI, 2019).

Table 29: Estimated ORRs and 95% Confidence Intervals Using Sample Size N = 55 for EXP-1

# Responses n/N	Overall Response Rate (95% CI)
28/55	50.9% (37.1 – 64.7)
33/55	60% (45.9 – 73.0)
37/55	67.3% (53.3 – 79.3)
39/55	70.9% (57.1 – 82.4)
**44/55**	**80% (67.0 – 89.6)**
50/55	90.9% (80.1 – 97.0)

After enrollment of 55 subjects specified above for the primary analysis, an additional 55 subjects are to be enrolled for a total of 110 subjects in EXP-1. If 88 out of 110 subjects have a confirmed objective response, the ORR (95% CI) will be 80% (71.3, 87.0) which will rule out an ORR ≤ 66% with at least 90% statistical power at the one-sided alpha level of 0.025 if the true ORR is 80%.

13.3.2.2 EXP-2 1 Prior ROS1 TKI AND 1 Platinum-based Chemotherapy ROS1+ NSCLC

Sixty *ROS1* TKI-pretreated eligible *ROS1*+ NSCLC subjects will be enrolled into the cohort with 1 prior ROS1 TKI. For this cohort, if the ORR is 23% or less, then it is assumed that repotrectinib is not effective. Using Table 30, if 21 subjects out of 60 subjects have a confirmed objective response (ORR = 35 %; 95% CI: 23.1 – 48.4) where the lower limit of the 95% CI is > 23%, repotrectinib is considered to be superior than the currently approved chemotherapy in the second line setting for NSCLC in this subject expansion cohort including the combination of docetaxel + ramucirumab (Cyramza® USPI, 2020) which has an ORR of 23% (95% CI: 20 – 26).

Table 30: Estimated ORRs and 95% Confidence Intervals Using Sample Size N = 60 for EXP-2

Responses n/N	Overall Response Rate (95% CI)
**21/60**	**35.0% (23.1 – 48.4)**
25/60	41.7% (29.1 – 55.1)
30/60	50.0% (36.8 63.2)

After enrollment of 60 subjects in the EXP-2 cohort as specified above for the primary analysis, an additional 60 subjects are to be enrolled for a total of 120 subjects in EXP-2.

13.3.2.3 EXP-3 2 Prior ROS1 TKI and NO Chemotherapy or Immunotherapy ROS1+ NSCLC

Forty *ROS1* TKI-pretreated ROS1+ eligible NSCLC subjects will be enrolled into the cohort with 2 prior ROS1 TKIs and no prior chemotherapy or immunotherapy. For this cohort, if the ORR is 10% or less, then it is assumed that repotrectinib is not effective. Using Table 31, if 10 subjects out of 40 subjects have a confirmed objective response (ORR = 25.0%; 95% CI: 12.7 – 41.2) where the lower limit of 95% CI >10%, repotrectinib will be considered efficacious in this cohort.

Table 31: Estimated ORRs and 95% Confidence Intervals Using Sample Size N = 40 for EXP-3

# Responses n/N	Overall Response Rate (95% CI)
8/40	20.0% (9.1 – 35.7)
9/40	22.5% (10.8 – 38.5)
**10/40**	**25.0% (12.7 – 41.2)**

After enrollment of 40 subjects in the EXP-3 cohort as specified above for the primary analysis, an additional 40 subjects are to be enrolled for a total of 80 subjects in EXP-3.

13.3.2.4 EXP-4 1 Prior ROS1 TKI and NO Chemotherapy or Immunotherapy ROS1+ NSCLC

Sixty *ROS1*+ NSCLC subjects pretreated with 1 prior ROS1 TKI and NO previous treatment with chemotherapy or immunotherapy will be enrolled into this cohort. Assuming a target ORR of 50%, with a sample size of 60, the 95% CI will be 36.8% - 63.2% with the lower bound greater than 35%, which shows a superiority to first-line chemotherapy in NSCLC. The ORR from platinum-based doublets or in combination with bevacizumab was in the range 25 – 35% (Abraxane® USPI, 2019; Alimta® USPI, 2019; Gemzar® USPI, 2019; Taxotere® USPI, 2019; Avastin USPI, 2009).

Table 32: Estimated ORRs and 95% Confidence Intervals Using Sample Size N = 60 for EXP-4

Responses n/N	Overall Response Rate (95% CI)
21/60	35.0% (23.1 – 48.4)
25/60	41.7% (29.1 – 55.1)
**30/60**	**50.0% (36.8 – 63.2)**
35/60	58.3% (44.9 – 70.9)

After enrollment of 60 subjects in the EXP-4 cohort as specified above for the primary analysis, an additional 60 subjects are to be enrolled for a total of 120 subjects in EXP-4.

As indicated above, EXP-4 was originally designed to show superiority to first-line chemotherapy in NSCLC with the target ORR of 50%. However, first-line chemotherapy in NSCLC does not seem to be the most appropriate comparator in this setting. There are currently no approved therapies indicated for ROS1+ advanced NSCLC patients who have progressed after first-line ROS1 TKI treatment (i.e., Crizotinib or Entrectinib). Platinum based chemotherapy is recommended for subjects who progress following treatment with a ROS1 TKI (NCCN 2022; Planchard 2019). Efficacy data for platinum-based chemotherapy in the second-line setting following first-line TKIs are limited. In one retrospective study of 21 patients with ROS1+ tumors that received 2L+ pemetrexed-based chemotherapy, the ORR was reported as 24% (5 out of 21 patients) (Zhang 2016). If the ORR of 38% is observed, a sample size of 55 is required to show superiority to chemotherapy following first-line ROS1 TKI, where the 95% CI of ORR will be 25.4% - 52.3% with the lower bound greater than 24%.

13.3.2.5 EXP-5 TRK TKI-Naïve NTRK+ Solid Tumors

Fifty-five TRK TKI-naïve *NTRK+* (*NTRK1, NTRK2,* and *NTRK3*) eligible solid tumor subjects will be enrolled into this cohort. For the TRK-naïve expansion cohort, if the ORR is 35% or less, then it is not considered as effective. Using Table 33, if 27 subjects out of 55 subjects have a confirmed objective response (ORR = 49.1%; 95% CI: 35.4 – 62.9) where the lower limit of the 95% CI is > 35%, repotrectinib is considered to be efficacious in this subject expansion cohort.

Table 33: Estimated ORRs and 95% Confidence Intervals Using Sample Size N = 55 for EXP-5

# Responses n/N	Overall Response Rate (95% CI)
20/55	36.4% (23.8 – 50.4)
22/55	40% (27.0 – 54.1)
**27/55**	**49.1% (35.4 – 62.9)**
28/55	50.9% (37.1 – 64.7)
33/55	60% (45.9 – 73.0)

After enrollment of 55 subjects in the EXP-5 cohort as specified above for the primary analysis, an additional 25 subjects are to be enrolled for a total of approximately 80 subjects in EXP-5.

13.3.2.6 EXP-6 TRK TKI-Pretreated NTRK+ Advanced Solid Tumors

Forty TRK TKI-pretreated *NTRK+* eligible solid tumor subjects will be enrolled into the cohort with 1 or 2 prior TRKi TKIs treatments. For this cohort, if the ORR is 10% or less, then it is assumed that repotrectinib is not effective. Using Table 34, if 9 out of 40 subjects have a confirmed

objective response (ORR = 22.5%; 95% CI: 10.8 – 38.5) where the lower limit of 95% CI >10%, repotrectinib will be considered efficacious in this cohort.

Table 34: Estimated ORRs and 95% Confidence Intervals using Sample Size N = 40 for EXP-6

# Responses n/N	Overall Response Rate (95% CI)
8/40	20.0% (9.1 – 35.7)
**9/40**	**22.5% (10.8 – 38.5)**
10/40	25.0% (12.7 – 41.2)

After enrollment of 40 subjects in the EXP-6 cohort as specified above for the primary analysis, an additional 80 subjects are to be enrolled for a total of approximately 120 subjects in EXP-6.

13.4 Safety Analyses

Safety analysis will be performed using the safety analysis set for each cohort, as well as for all cohorts combined.

13.4.1 Extent of Exposure

Exposure analysis will be based on the actual dose administered for the study drug. Duration of treatment will be calculated as last dose date minus first dose date plus 1 day. Duration of treatment, and total daily dose amount will be summarized using descriptive statistics. The number of subjects treated by cycles, and the number of subjects with dose reduction/interruptions will be summarized.

13.4.2 Adverse Events

Adverse events will be graded according to the CTCAE v4.03 and coded to preferred term and system organ class (SOC) using the most recent version of MedDRA.

All AEs reported during the AE reporting period (inclusive of the 28-day post last dose of study drug period) will be considered as TEAEs.

For each subject population expansion cohort, incidence rates will be summarized with frequency and percentage by MedDRA SOC and preferred term, with all subjects treated in that particular expansion cohort as the denominator, unless otherwise specified. In addition, AE incidence rates will also be summarized by severity and relationship to study drug. Treatment-related AEs are those judged by the Investigator to be at least possibly related to the study drug. Subjects with multiple occurrences of events will only be counted once at the maximum severity to study drug for each preferred team, SOC, and overall. Deaths that occur within 28 days after the last dose of study drug are defined as on study deaths and reported as SAEs within the required reporting period for Phase1. In Phase 2, death due to disease progression will not be considered an SAE.

Summary tables and individual subject listings will be prepared as per the SAP.

13.4.3 Clinical Laboratory Results

Normal ranges will be used to identify values that are outside the normal ranges and abnormal laboratory results will be graded according to the CTCAE v4.03. Descriptive statistics will be provided for each test result and for the change from baseline by visit for subjects who have data collected at that visit.

A shift summary of baseline grade by maximum post-baseline CTCAE grade will be presented, as appropriate. For each laboratory parameter, the baseline laboratory value will be defined as the last laboratory value collected on or prior to the date of the first dose of study drug. Subjects with a missing baseline or all post-baseline results will be reflected as missing in the shift table.

Subjects who develop toxicities of CTCAE grade ≥ 3 will be summarized. Laboratory test results not having CTCAE grade will also be summarized. Parameters that have criteria available for both low and high values (eg, hypercalcemia versus hypocalcemia) will be summarized for both criteria. Subjects will only be counted once for each criterion.

13.4.4 Vital Signs

Each vital sign (body temperature, blood pressure [systolic and diastolic], respiration rate, and heart rate) will be summarized and presented by study visit. Subjects with clinically significant abnormalities as compared to baseline will be listed.

13.4.5 Concomitant Medications/Treatment

All medications and/or treatments received during the protocol treatment period will be considered as concomitant medications and/or concomitant treatments and will be coded by the World Health Organization Drug Dictionary. Concomitant medications will also be summarized by Anatomical Therapeutic Chemical (ATC) level 2 and generic drug names. Subjects who received concomitant medications and/or treatments will be listed.

13.4.6 ECG

The analysis of ECG results will be based on Safety Population subjects with baseline and on treatment ECG data. ECG collected prior to the first day of dosing will be considered the baseline ECG.

ECG measurements (an average of the triplicate measurements) will be used for the statistical analysis and all data presentations. Any data obtained from ECGs repeated for safety reasons after the nominal time points will not be averaged along with the preceding triplicates. Interval measurements from repeated ECGs will be included in the outlier analysis as individual values obtained at unscheduled time points.

QT intervals will be corrected for heart rate (QTc) using standard correction factors (ie, Bazett's, Fridericia's, and possibly a study-specific factor). The adequacy of the correction method will be assessed graphically (plots of QT and QTc versus RR) and supplementary transformations may be considered, as appropriate. Data will be summarized and listed for QT, PR, QRS, QTcF, and QTcB by cycle, day, and dose. Individual QTc (all evaluated corrections) intervals will be listed by time and dose. Descriptive statistics (n, mean, median, standard deviation, minimum, and

maximum) will be used to summarize the absolute QTc value and changes from baseline in QTc after treatment by cycle, day, dose, and by time point. For each subject and by treatment, the maximum change from baseline will be calculated as well as the maximum post-baseline value across time points. Outlier analysis of the QTc data will be conducted and summarized as follows:

- The number of subjects with maximum change from baseline in QTcF (< 30, 30-60, and > 60 msec)
- The number of subjects with maximum post-dose (post-baseline) QTcF (< 450, 450 to < 480, 480 to ≤ 500, and > 500 msec).

Shift tables will be provided for baseline vs worst on study QTc (one or more correction method will be used) using maximum CTCAE grade (V4.03), as well as tables of ECG abnormality at baseline (yes, no, not done: [n, %]). Subjects experiencing clinically relevant morphological ECG changes will be summarized (including frequency and percentage). The effect of drug concentrations on QTc change from baseline will be explored graphically. Additional concentration-QTc analyses may be performed. Data may be pooled with other study results and/or explored further with PK/PD models.

13.4.7 Left Ventricular Ejection Fraction (LVEF)

For subjects with MUGA scans or echocardiograms, individual LVEF proportion (%) and its changes from baseline will be summarized by time point. The number of subjects and the percentage whose maximum decrease from baseline in LVEF is ≥ 20% will be calculated.

13.4.8 Ophthalmologic Assessment

Ophthalmologic assessments will be summarized using descriptive statistics by visit, change from baseline by visit, and changes from baseline to worst post baseline result, depending on the format of the data collected for each assessment. Specific objective ophthalmologic findings from each assessment will be listed.

13.5 Analysis of Pharmacokinetics

13.5.1 Single- and Multiple-Dose Repotrectinib Pharmacokinetic Analysis

Repotrectinib plasma concentration-time data will be analyzed using non-compartmental methods to estimate the following PK parameters in individual patients: maximum plasma concentration (C_{max}), time to maximum plasma concentration (T_{max}), area under the plasma concentration-versus-time curve (AUC_{last}). If data permit or if considered appropriate, area under the plasma concentration-versus-time curve to infinity (AUC_{inf}), terminal elimination half-life ($t_{1/2}$), apparent plasma clearance (CL/F), apparent volume of distribution (V_z/F), and accumulation ratio (R_{acc}) will be estimated. The single- and multiple-dose PK parameters will be summarized descriptively by dose, cycle, and day.

Repotrectinib concentrations will be summarized descriptively (n, mean, SD, CV, median, minimum, maximum, geometric mean and its associated CV) by dose, cycle, day and nominal time. Individual, mean and median profiles of the concentration-time data will be plotted by dose,

cycle and day (single dose, and multiple-dose) using nominal time. Median and mean profiles will be presented on both linear and log-linear scales.

Dose normalized AUC_{inf}, (AUC_{τ} at steady-state), AUC_{last}, and C_{max} will be plotted against dose (using a logarithmic scale) by cycle and day. These plots will include individual patient values and the geometric means for each dose. These plots will be used to help understand the relationship between PK exposure and dose.

The observed accumulation ratio will be summarized descriptively. It will be analyzed after natural log transformation using a one-way analysis of variance with a single term for dose. The means and 90% CIs obtained from the model will be back-transformed to provide means and 90% CIs for the accumulation and linearity ratios for each dose.

Trough concentrations will be plotted for each dose using a box-whisker plot by cycle and day within cycle in order to assess the attainment of steady-state.

13.5.1.1 Effect of Food on Repotrectinib Pharmacokinetics

To evaluate the food effect, repotrectinib plasma concentration-time data will be compared on Day -7 to Cycle 1 Day 1. Non-compartmental methods will be used to estimate PK parameters. Natural log transformed C_{max}, AUC_{last}, and AUC_{inf} (if data permit) values will be analyzed using a mixed effects model with sequence and treatment (fed, fasted) as fixed effects and patient within sequence as a random effect. Estimates of the adjusted mean differences (Fed-Fasted) and corresponding 90% CIs will be calculated from the model. The adjusted mean differences and 90% CIs for the differences will be exponentiated to provide estimates of the ratios of the adjusted geometric means (Fed/Fasted) and 90% CIs for the ratios.

13.5.1.2 Effect of Repotrectinib on MDZ Pharmacokinetics

Plasma concentration-time data of MDZ in the absence and presence of repotrectinib will be analyzed using non-compartmental methods to estimate the following PK parameters in individual patients: C_{max}, T_{max}, AUC_{last}, and if data permit, AUC_{inf}, $t_{1/2}$, CL/F and V/F.

The PK parameters C_{max}, AUC_{last}, and AUC_{inf} (if data permit) will be utilized to estimate the effect of multiple doses of repotrectinib on MDZ PK.

13.5.1.3 Population Pharmacokinetic Analysis or PK/PD Modeling

Pharmacokinetic and pharmacodynamic data from this study may be analyzed using modeling approaches (population PK and PK/PD modeling). The results of these analyses, if performed, may be reported separately.

13.6 Subject-Reported Outcomes (PROs)

Subject-reported outcomes (PROs) will be administered during the Phase 2 portion of the study.

PRO is an umbrella term referring to all outcomes and symptoms that are directly reported by the subject. PROs have become a significant endpoint when evaluating effectiveness of treatments in clinical trials.

Subjects will complete the self-administered quality of life (QOL) instruments listed below in Table 35 every 4 weeks: The subject should complete the questionnaires at the scheduled clinic visit at baseline (screening) and then every 4 weeks. The subject should also complete a questionnaire at progression and their treatment discontinuation visit. If any scheduled PRO assessment is not completed, the reason for non-completion should be recorded.

Table 35: Subject-Reported Outcome QOL Instruments

Instruments[1]	Definition
EORTC QLQ-C30 (APPENDIX 4)	Core Quality of Life Questionnaire:<br>• Comprises 30 questions assessing global QOL, functioning, and symptoms of both multi-item and single-item measures<br>• Developed to assess the quality of life of cancer subjects (Aaronson 1993)<br>• Has been translated and validated into 81 languages and is used in more than 3,000 studies worldwide<br>• (http://groups.eortc.be/qol/eortc-qlq-c30)
EORTC QLQ-LC13 (APPENDIX 5)	Lung Cancer Module (NSCLC subjects only)<br>• Comprises 13 questions assessing lung cancer-specific symptoms (Bergman 1994)

1 Developed by European Organization for Research and Treatment of Cancer (EORTC)

The following PROs will be administered: European Organization for Research and Treatment of Cancer (EORTC) QLQ-C30 and QLQ-LC-13 (for subjects with either *ROS1*-, or *NTRK*-rearranged NSCLC).

13.6.1 Administration of PROs

PROs will only be administered during the Phase 2 Dose Expansion portion of the study.

Questionnaires will be administered. The subject should complete the questionnaires at the scheduled clinic visit at screening, and every 4 weeks relative to the first dose of multiple dosing, discontinuation, and progression as specified in the Study Calendars (Table 26).

PROs will be filled out prior to any other site activities and encounters with physician. The subjects will be instructed to complete the PRO independently. The site will have a designated quiet space for subjects to use when completing the assessments. Each center should allocate responsibility for PRO assessment to a specified individual (eg, a research nurse).

It is important that the value and relevance of QOL data are explained carefully to participating subjects so that they are motivated to comply with data collection. The appointed individual should also stress that the information is confidential. Therefore, if the subject has any medical problems she should discuss them with the doctor or research nurse separately from their QoL assessment.

The instructions for completion of questionnaires are:

- It must be completed before any investigations or discussions about the status of the subject's disease with the clinic staff.

- The subject must complete it themselves without any intervention from family, friends, center staff, etc.
- The only exception to this is if the subject is blind or illiterate. In this case the questionnaire may be read to the subject verbatim; however, the reader must not aid in the interpretation of questions or in the selection of answers.
- Only one answer to every question should be checked.
- Study center personnel should not review the responses to the questionnaire with the subject or with any other center staff.

Following completion, the appointed individual may quickly scan the questionnaire visually for completeness and should confirm verbally with the subject that the questionnaire has been completed fully.

13.6.2 EORTC-QLQ-C30

The EORTC-QLQ-C30 was developed by the EORTC Quality of Life Group 1993. It consists of 30 items and measures cancer subjects' functioning (QoL) and symptoms (Aaronson 1993).

Note that the term item refers to a question. For example, "Do you have any trouble doing strenuous activities, like carrying a heavy shopping bag or a suitcase?" refers to question 1 of the EORTC-QLQ-C30 questionnaire.

The following variables are to be calculated from the QLQ-C30 questionnaires:

- Global health status: Global health status
- Function scales: Physical functioning, Role functioning, Emotional functioning, Cognitive functioning, and Social functioning
- Symptoms: Fatigue, Nausea and vomiting, and Pain
- Single items: Dyspnea, Insomnia, Appetite loss, Constipation, Diarrhea, and Financial difficulties

Each of these variables is described as a domain in the EORTC scoring manual. The Global health status, Functional scales, and Symptom variables are multi-item domains (consist of more than 1 question). The single-item domains consist of only 1 question.

There are 28 questions whose options are: Not at all (score is 1), A little (score is 2), Quite a bit (score is 3), and Very much (score is 4). Question 29 and 30, which are for measuring global health status, are scored from 1 (very poor) to 7 (excellent).

Each of the multi-item domains includes a different set of items; no item occurs in more than one domain. Note that:

- A computed high score for a functional domain represents a high/healthy level of functioning, whereas a high raw score for a functional item represents a low/poor level of functioning;
- A computed/raw high score for the global health status/QOL represents a high QOL;

- A computed/raw high score for a symptom domain/item represents a high level of symptomatology/problems.

Note: No items are to be reversed for scoring.

The principle for scoring these scales is the same in all cases:

15) Estimate the average of the items that contribute to the scale; this is the raw score (RS).

Use a linear transformation to standardize the raw score, so that scores range from 0 to 100; a higher score represents a higher ("better") level of functioning, or a higher ("worse") level of symptoms.

13.6.2.1 Technical Summary

In practical items, if terms I1, I2… In are included in a scale, the procedure is as follows:

Raw score: calculate the raw score, Raw score = RS = (I1+ I2+…+In)/n

13.6.2.2 Linear Transformation

Apply the linear transformation to 0-100 to obtain the score S.

Functional scales: S = {1 – ((RS – 1) /range)}*100
Symptom scales/items: S = {(RS – 1) /range}*100
Global health score /QOL: S = {(RS – 1) /range}*100

Range is difference between the maximum possible value of RS and minimum possible value. The QLQ-C30 has been designed so that all items in any scale take the same range of values.

Examples:

Emotional functioning, Raw score = (Q21+ Q22+ Q23+ Q24)/4

EF score = {1 – ((RS – 1)/3)}*100

13.6.2.3 Missing Items for QLQ-C30

If at least half of the items (ie, 3 of 6 items, or 3 of 5 items) from the domain have been answered, assume that the missing items have values equal to the average of those items that are present for that respondent. This is equivalent to the raw score being taken as the mean of the non-missing item values. As a result, none of the single-item measures can be imputed.

Example:

Emotional Functioning if Q23 is missing (ie, 3 items are not missing)

Raw/Score = (Q21+Q22+Q24)/3
EF Score = {1 – ((RS – 1)/3)}*100

13.6.3 EORTC-QLQ-LC13

The EORTC-QLQ-LC13 is a complementary module measuring lung cancer-associated symptoms and side effects from conventional chemotherapy and radiotherapy (Bergman 1994). The EORTC-

QLQ-LC13 incorporates 1 multi-item domain to assess dyspnea, and a series of single items assessing pain, coughing, sore mouth, dysphagia, peripheral neuropathy, alopecia, and hemoptysis.

There are 12 questions whose options are: Not at all (score is 1), A little (score is 2), Quite a bit (score is 3), and Very much (score is 4).

A low score for a domain represents a high/healthy level.

Scoring procedure: The scoring approach for the EORTC-QLQ-LC13 is identical in principle to that for the symptom domains/single items of the EORTC-QLQ-C30.

Note: Imputation will be performed for EORTC-QLQ-LC13 for Dyspnea only if question 5 is not missing (eg, if RS for dyspnea will be Q3 + Q5 if Q4 is missing, then RS for dyspnea will be Q4 + Q5)

If Q5 is missing then dyspnea will not be derived, however Q3 and Q4 will be summarized separately.

13.6.4 QOL Compliance Rates

Summary measures of overall compliance and compliance over time will be derived for EORTC-QLQ-C30 and EORTC-QLQ-LC13 scores. These will be based upon:

- Received forms = number of EORTC-QLQ-C30 and EORTC-QLQ-LC13 forms received back plus the number not received back where the reason was 'Subject too heavily affected by symptoms of disease under investigation'
- Expected forms = number of subjects still on treatment at the specified assessment time excluding subjects in countries with no available translation
- Evaluable forms = EORTC-QLQ-C30 and EORTC-QLQ-LC13 forms with at least 1 domain that can be determined

Thus, the overall compliance rate is defined as number of subjects with an evaluable baseline and at least 1 evaluable follow-up form (as defined above), divided by the number of subjects expected to have completed at least a baseline EORTC-QLQ-C30 and EORTC-QLQ-LC13 form.

Compliance over time will be calculated separately for each visit, including baseline, as the number of subjects with an evaluable form at the time point (as defined above), divided by number of subjects still expected to complete forms. Similarly, the evaluability rate over time will be calculated separately for each visit, including baseline, as the number of evaluable forms (per definition above), divided by the number of received forms.

13.6.5 PRO Endpoints

All QOL instruments will be reported on a 0 to 100 scale.

For the EORTC-QLQ-C30 and EORTC-QLQ-LC13, respectively, each domain and item will be linearly transformed to standardize the raw score to a range from 0 to 100, with 100 representing the best possible function/QOL and highest symptom severity.

The following analysis variables will be summarized for all QOL instruments, domains, and single items:

- Mean change from baseline, by cycle and across cycle
- Proportion of subjects with improved, stable, and worsened outcomes, by cycle and across cycle

A 10-point change from baseline in an item or domain is established to be clinically meaningful (Osoba 1998). Hence, for functioning domains and global QOL, a subject will be deemed:

- Improved, if the change from baseline is a 10-points or greater increase
- Worsened, if the change from baseline is 10-point or greater decrement
- Stable otherwise

For analysis of symptom domains and single items, the opposite is true:

- Improved, if the change from baseline is a 10-points or greater decrement
- Worsened, if the change from baseline is 10-point or greater increase
- Stable otherwise

13.6.6 PRO Analyses

For each subject population expansion cohort and using the PRO-evaluable population, the number and percentage of subjects of the total number eligible at each cycle who completed each questionnaire will be summarized per time point. The questionnaire domains and items will be scored according to each questionnaire's respective scoring algorithm. Analyses will include the mean change from baseline scores across cycles and the proportion of subjects with improved, stable, or worsened outcomes.

13.6.6.1 Change from Baseline Scores

Descriptive statistics (including 95% Cis) of raw and change from baseline scores will be performed by cycle and across cycle for the EORTC-QLQ-C30 and EORTC-QLQ-LC13 items. In addition, results will be depicted graphically.

13.6.6.2 Proportion of Subjects Improved, Remained Stable, or Worsened

The proportion of subjects who improved, remained stable, or worsened will be reported by cycle and across cycle.

13.7 Interim Analysis

In Phase 2, interim analyses for cohorts EXP-2 and EXP-4 are planned when 50% (n=30) of the subjects in each cohort have been treated and followed for response for at least their first 2 post-baseline scans.

See Section 9 on safety and efficacy monitoring.

14 DATA COLLECTION, RETENTION AND MONITORING

14.1 Data Collection Instruments

The Investigator will prepare and maintain adequate and accurate source documents designed to record all observations and other pertinent data for each subject treated with the study drug.

Study personnel at each site will enter data from source documents corresponding to a subject's visit into the protocol-specific electronic CRF when the information corresponding to that visit is available. Subjects will not be identified by name in the study database or on any study documents to be collected by the Sponsor (or designee), but will be identified by a site number, subject number, and/or subject initials, as per local regulations.

If a correction is required for an CRF, the time and date stamps track the person entering or updating CRF data and creates an electronic audit trail.

The Investigator is responsible for reviewing all information collected on subjects enrolled in this study for completeness and accuracy. A copy of the CRF will remain at the Investigator's site at the completion of the study.

14.2 Data Management Procedures

The data will be entered into a validated database. The Sponsor- or designee-designated data management group will be responsible for data processing, in accordance with procedural documentation. Database lock will occur once quality assurance procedures have been completed.

All procedures for the handling and analysis of data will be conducted using good computing practices meeting FDA guidelines for the handling and analysis of data for clinical trials.

14.3 Data Quality Control and Reporting

After data have been entered into the study database, a system of computerized data validation checks will be implemented and applied to the database on a regular basis. Queries are entered, tracked, and resolved through the electronic data capture (EDC) system directly. The study database will be updated in accordance with the resolved queries. All changes to the study database will be documented.

14.4 Data Archival

The database is safeguarded against unauthorized access by established security procedures; appropriate backup copies of the database and related software files will be maintained. Databases are backed up by the database administrator in conjunction with any updates or changes to the database.

At critical junctures of the protocol (eg, production of interim and final reports), data for analysis are locked and cleaned per established procedures.

14.5 Availability and Retention of Investigational Records

To enable evaluations and/or audits from regulatory authorities or the Sponsor, the Investigator agrees to keep records, including the identity of all participating subjects (sufficient information to link records [eg, CRFs and hospital records]), all original signed informed consent forms, copies

of all CRFs, safety reporting forms, source documents, and detailed records of treatment disposition, and adequate documentation of relevant correspondence (eg, letters, meeting minutes, telephone calls reports). The records should be retained by the Investigator according to ICH, local regulations, or as specified in the Clinical Trial Agreement, whichever is longer; but at a minimum, all study documentation must be retained for 2 years after the last marketing application approval in an ICH region or after at least 2 years have elapsed since formal discontinuation of clinical development of repotrectinib.

If the Investigator becomes unable for any reason to continue to retain study records for the required period (eg, retirement, relocation), then the Sponsor should be prospectively notified. The study records must be transferred to a designee acceptable to the Sponsor, such as another Investigator, another institution, or to the Sponsor itself. The Investigator must obtain the Sponsor's written permission before disposing of any records, even if retention requirements have been met.

The Investigator agrees to comply with all applicable laws and regulations related to the privacy and protection of patient health information.

14.6 Monitoring

For country-specific requirements, please refer to APPENDIX 8.

Monitoring visits will be conducted by authorized representatives of the Sponsor according to the US Code of Federal Regulations (CFR) Title 21 Parts 50, 56, and 312 and ICH Guidelines for GCP (E6)[1], as well as any applicable local, regional, and country laws. By signing this protocol, the Investigator grants permission to the Sponsor (or designee), the US FDA, other regulatory agencies, IRB/ECs, and respective national or local health authorities to conduct on-site monitoring and/or auditing of all appropriate study documentation.

14.7 Subject Confidentiality

In order to maintain subject confidentiality, only a site number and subject number will identify all study subjects on CRFs and other documentation submitted to the Sponsor. Additional subject confidentiality issues (if applicable) are covered in the Clinical Trial Agreement.

The Investigator must ensure that the study is performed in accordance with the applicable data protection laws, including Regulation (EU) 2016/679 of the European Parliament and of the Council of 27 April 2016 on the protection of natural persons with regard to the processing of personal data and on the free movement of such data, and repealing Directive 95/46/EC (General Data Protection Regulation or GDPR). The Investigator must ensure that the subject's anonymity is maintained and keep in strict confidence documents not de-identified (eg, signed informed consent form). The Investigator must also provide all reasonable assistance to the Sponsor in the exercise of their duties as Data Controller (as defined by the GDPR) for the study.

[1] ICH E6(R1) Tripartite Guideline. Guideline for Good Clinical Practice. 10 June 1996.

15 ADMINISTRATIVE, ETHICAL, REGULATORY CONSIDERATIONS

This study will be conducted in accordance with the US Food and Drug Administration (FDA) regulations, the International Council for Harmonisation (ICH) E6 Guideline for Good Clinical Practice (GCP), and applicable local, state, and federal laws, as well as other applicable country laws.

To maintain confidentiality, all laboratory specimens, evaluation forms, reports, and other records will be identified by a coded number. All study records will be kept in a locked file cabinet and code sheets linking a subject's name to a subject identification number will be stored separately in another locked file cabinet. Clinical information will not be released without written permission of the subject, except as necessary for inspection by the FDA or other regulatory agencies, IRB/ECs, and respective national or local health authorities. The Investigator must also comply with all applicable privacy regulations (eg, Health Insurance Portability and Accountability Act of 1996).

15.1 Protocol Amendments

Any amendment to the protocol will be written by the Sponsor. Protocol amendments cannot be implemented without submission to the appropriate health authorities for review and written IRB/EC approval except as necessary to eliminate immediate safety hazards to subjects. A protocol amendment intended to eliminate an apparent immediate hazard to subjects may be implemented immediately, provided the IRB/ECs are notified within 5 working days.

15.2 Institutional Review Boards/Ethic Committees

In accordance with 21CFR part 50 and 56, the Investigator agrees to obtain IRB/IEC approval of all appropriate material, including a copy of the protocol, ICF(s), Investigator's Brochure, and subject recruitment materials/processes (eg, advertisements, subject dosing diary), prior to the start of the study and/or prior to its use on the study.

Informed consent will be obtained in accordance with ICH GCP, US Code of Federal Regulations for Protection of Human Subjects (21 CFR 50.25[a, b], CFR 50.27, and CFR Part 56, Subpart A), the Health Insurance Portability and Accountability Act (if applicable), and local regulations.

Either the local institution's or Sponsor's designated central laboratory molecular testing consent form can be used to perform molecular testing in order to determine enrollment eligibility.

For all subjects, the Sponsor's clinical trial informed consent form will be used.

The Sponsor or its designee must review and approve any proposed deviations from the sample ICFs or any alternate consent forms proposed by the site before IRB/EC submission. Subjects must be re-consented to the most current version of the consent forms during their participation in the study. The final IRB/EC-approved consent forms must be provided to the Sponsor for regulatory purposes.

The consent forms must be signed by the subject or the subject's legal representative before his/her participation in the study. The case history for each subject shall document the informed consent process and that written informed consent was obtained prior to participation in the study. A copy

of each signed consent form must be provided to the subject or the subject's legal representative. If applicable, it will be provided in a certified translation of the local language.

All signed and dated consent forms must remain in each subject's study file and must be available for verification by study monitors at any time.

The informed consent form should be revised whenever there are changes to procedures outlined in the informed consent or when new information becomes available that may affect the willingness of the subject to participate.

For any updated or revised consent forms, the case history for each subject shall document the informed consent process and that written informed consent was obtained for the updated/revised consent form for continued participation in the study. The final revised IRB/EC-approved informed consent form must be provided to the Sponsor for regulatory purposes.

15.3 Reporting of Safety Issues and Serious Breaches of the Protocol or ICH GCP

In the event of any prohibition or restriction imposed (ie, clinical hold) by an applicable Competent Authority in any area of the World, or if the Investigator is aware of any new information that might influence the evaluation of the benefits and risks of the IP, the Sponsor should be informed immediately.

In addition, the Investigator will inform the Sponsor immediately of any urgent safety measures taken by the Investigator to protect the study subjects against any immediate hazard, and of any serious breaches of this protocol or of ICH GCP of which the Investigator becomes aware.

15.4 Sponsor Discontinuation Criteria

Premature termination of this study may occur because of a regulatory authority decision, change in opinion of the IRB/EC, drug safety problems, or at the discretion of the Sponsor. In addition, the Sponsor retains the right to discontinue development of repotrectinib at any time.

If a study is prematurely terminated or discontinued, then the Sponsor will promptly notify the Investigator. After notification, the Investigator must notify the respective IRB/EC, and contact all participating subjects and the hospital pharmacy (if applicable) within a 4-week time period. As directed by the Sponsor, all study materials must be collected and all CRFs completed to the greatest extent possible.

15.5 Post-Trial Access

At the conclusion of the study, participants who continue to demonstrate clinical benefit may be eligible to receive Sponsor-supplied study treatment. Study treatment may be provided through another mechanism at the discretion of the Sponsor. The Sponsor reserves the right to terminate access to the Sponsor-supplied study treatment if any of the following occur: a) the study is terminated due to safety concerns; b) the development of repotrectinib is terminated for other reasons, including but not limited to lack of efficacy and/or not meeting the study objectives; c) the participant can obtain medication from a government sponsored or private health program. In all cases, the Sponsor will follow local regulations.

15.6 Publications

Publication of study results is discussed in the Clinical Trial Agreement. Details regarding production of manuscripts and conference presentations will adhere to the International Committee of Medical Journal Editors (ICMJE) requirements for authorship and contributorship.

http://www.icmje.org/recommendations/browse/roles-and-responsibilities/defining-the-roleof-authors-and-contributors.html

16 REFERENCES

Aaronson NK, Ahmedzai S, Bergman B, et al. The European Organization for Research and Treatment of Cancer QLQ-C30: A Quality-of-Life Instrument for Use in International Clinical Trials in Oncology. JNCI: Journal of the National Cancer Institute. 1993;85(5):365-76. doi: 10.1093/jnci/85.5.365.

Abraxane® [paclitaxel] Package Insert, Los Angeles, CA, Abraxis BioScience, LLC, 2019.

Aisner DL, Nguyen TT, Paskulin DD, et al. ROS1 and ALK fusions in colorectal cancer, with evidence of intratumoral heterogeneity for molecular drivers. Mol Cancer Res. 2014;12(1):111-8. doi: 10.1158/1541-7786.MCR-13-0479-T.

Alimta® [pemetrexed disodium] Package Insert, Indianapolis, IN; Eli Lilly and Company, 2019.

Ardini E, Bosotti R, Borgia AL, et al. The TPM3-NTRK1 rearrangement is a recurring event in colorectal carcinoma and is associated with tumor sensitivity to TRKA kinase inhibition. Molecular oncology. 2014;8(8):1495-507. doi: 10.1016/j.molonc.2014.06.001.

Avastin® [bevacizumab] Package Insert, South San Francisco, CA, Genentech, Inc., 2020.

Awad MM, Katayama R, McTigue M, et al. Acquired resistance to crizotinib from a mutation in CD74-ROS1. N Engl J Med. 2013;368(25):2395-401. doi: 10.1056/NEJMoa1215530.

Balko JM, Schwarz LJ, Luo N, et al. Triple-negative breast cancers with amplification of JAK2 at the 9p24 locus demonstrate JAK2-specific dependence. Science translational medicine. 2016;8(334):334ra53-ra53. doi: 10.1126/scitranslmed.aad3001.

Barbacid M. Structural and functional properties of the TRK family of neurotrophin receptors. Ann N Y Acad Sci. 1995;766:442-58.

Barlesi F, Mazieres J, Merlio JP, et al. Routine molecular profiling of patients with advanced non-small-cell lung cancer: results of a 1-year nationwide programme of the French Cooperative Thoracic Intergroup (IFCT). Lancet. 2016;387(10026):1415-26. doi: 10.1016/S0140-6736(16)00004-0.

Bergethon K, Shaw AT, Ou SH, et al. ROS1 rearrangements define a unique molecular class of lung cancers. J Clin Oncol. 2012;30(8):863-70. doi: 10.1200/JCO.2011.35.6345.

Bergman B, Aaronson NK, Ahmedzai S, et al. The EORTC QLQ-LC13: a modular supplement to the EORTC Core Quality of Life Questionnaire (QLQ-C30) for use in lung cancer clinical trials. EORTC Study Group on Quality of Life. Eur J Cancer. 1994;30A(5):635-42.

Blume-Jensen P, Hunter T. Oncogenic kinase signalling. Nature. 2001;411(6835):355-65. doi: 10.1038/35077225.

Brenca M, Rossi S, Polano M, et al. Transcriptome sequencing identifies ETV6-NTRK3 as a gene fusion involved in GIST. J Pathol. 2016;238(4):543-9. doi: 10.1002/path.4677.

Bromann PA, Korkaya H, Courtneidge SA. The interplay between Src family kinases and receptor tyrosine kinases. Oncogene. 2004;23(48):7957-68. doi: 10.1038/sj.onc.1208079.

Buchert M, Burns CJ, Ernst M. Targeting JAK kinase in solid tumors: emerging opportunities and challenges. Oncogene. 2016;35(8):939-51. doi: 10.1038/onc.2015.150.

Castellana B, Aasen T, Moreno-Bueno G, et al. Interplay between YB-1 and IL-6 promotes the metastatic phenotype in breast cancer cells. Oncotarget. 2015;6(35):38239-56. doi: 10.18632/oncotarget.5664.

Charest A, Lane K, McMahon K, et al. Fusion of FIG to the receptor tyrosine kinase ROS in a glioblastoma with an interstitial del(6)(q21q21). Genes Chromosomes Cancer. 2003;37(1):58-71. doi: 10.1002/gcc.10207.

Costa DB, Shaw AT, Ou SH, et al. Clinical Experience With Crizotinib in Patients With Advanced ALK-Rearranged Non-Small-Cell Lung Cancer and Brain Metastases. J Clin Oncol. 2015;33(17):1881-8. doi: 10.1200/JCO.2014.59.0539.

Créancier L, Vandenberghe I, Gomes B, et al. Chromosomal rearrangements involving the NTRK1 gene in colorectal carcinoma. Cancer Lett. 2015;365(1):107-11. doi: 10.1016/j.canlet.2015.05.013.

Crescenzo R, Abate F, Lasorsa E, et al. Convergent mutations and kinase fusions lead to oncogenic STAT3 activation in anaplastic large cell lymphoma. Cancer Cell. 2015;27(4):516-32. doi: 10.1016/j.ccell.2015.03.006.

Crystal AS, Shaw AT, Sequist LV, et al. Patient-derived models of acquired resistance can identify effective drug combinations for cancer. Science (New York, NY). 2014;346(6216):1480-6. doi: 10.1126/science.1254721.

Cyramza® [package insert]. Indianapolis, IN; Eli Lilly and Company, 2020.

Debelenko LV, Raimondi SC, Daw N, et al. Renal cell carcinoma with novel VCL-ALK fusion: new representative of ALK-associated tumor spectrum. Mod Pathol. 2011;24(3):430-42. doi: 10.1038/modpathol.2010.213.

Demeure MJ, Aziz M, Rosenberg R, et al. Whole-genome sequencing of an aggressive BRAF wild-type papillary thyroid cancer identified EML4-ALK translocation as a therapeutic target. World J Surg. 2014;38(6):1296-305. doi: 10.1007/s00268-014-2485-3.

Doebele RC, Davis LE, Vaishnavi A, et al. An Oncogenic NTRK Fusion in a Patient with Soft-Tissue Sarcoma with Response to the Tropomyosin-Related Kinase Inhibitor LOXO-101. Cancer Discov. 2015;5(10):1049-57. doi: 10.1158/2159-8290.CD-15-0443.

Drilon A, Somwar R, Wagner JP, et al. A Novel Crizotinib-Resistant Solvent-Front Mutation Responsive to Cabozantinib Therapy in a Patient with ROS1-Rearranged Lung Cancer. Clin Cancer Res. 2016a;22(10):2351-8. doi: 10.1158/1078-0432.CCR-15-2013.

Drilon A, Li G, Dogan S, et al. What hides behind the MASC: clinical response and acquired resistance to entrectinib after ETV6-NTRK3 identification in a mammary analogue secretory carcinoma (MASC). Ann Oncol. 2016b;27(5):920-6. doi: 10.1093/annonc/mdw042.

Drilon A, Laetsch TW, Kummar S, et al. Efficacy of Larotrectinib in TRK Fusion-Positive Cancers in Adults and Children. N Engl J Med. 2018;378(8):731-9. doi: 10.1056/NEJMoa1714448.

Eisenhauer EA, Therasse P, Bogaerts J, Schwartz LH, Sargent D, Ford R, Dancey J, Arbuck S, Gwyther S, Mooney M, Rubinstein L, Shankar L, Dodd L, Kaplan R, Lacombe D, Verweij J. New response evaluation criteria in solid tumours: revised RECIST guideline (version 1.1). Eur J Cancer. 2009 Jan;45(2):228-47. doi: 10.1016/j.ejca.2008.10.026. PMID: 19097774.

Farago AF, Le LP, Zheng Z, Muzikansky A, Drilon A, Patel M, Bauer TM, Liu SV, Ou SH, Jackman D, Costa DB, Multani PS, Li GG, Hornby Z, Chow-Maneval E, Luo D, Lim JE, Iafrate AJ, Shaw AT. Durable Clinical Response

to Entrectinib in NTRK1-Rearranged Non-Small Cell Lung Cancer. J Thorac Oncol. 2015;10(12):1670-4. doi: 10.1097/01.JTO.0000473485.38553.f0.

Formisano L, Nappi L, Rosa R, et al. Epidermal growth factor-receptor activation modulates Src-dependent resistance to lapatinib in breast cancer models. Breast Cancer Res. 2014;16(3):R45. doi: 10.1186/bcr3650.

Frattini V, Trifonov V, Chan JM, et al. The integrated landscape of driver genomic alterations in glioblastoma. Nat Genet. 2013;45(10):1141-9. doi: 10.1038/ng.2734.

Gainor JF, Dardaei L, Yoda S, et al. Molecular Mechanisms of Resistance to First- and Second-Generation ALK Inhibitors in ALK-Rearranged Lung Cancer. Cancer Discov. 2016;6(10):1118-33. doi: 10.1158/2159-8290.CD-16-0596.

Gao SP, Chang Q, Mao N, et al. JAK2 inhibition sensitizes resistant EGFR-mutant lung adenocarcinoma to tyrosine kinase inhibitors. Sci Signal. 2016;9(421):ra33. doi: 10.1126/scisignal.aac8460.

Gemzar® [gemcitabine hydrochloride] Package Insert, Indianapolis, IN, Eli Lilly and Company, 2019.

Giacomini CP, Sun S, Varma S, et al. Breakpoint analysis of transcriptional and genomic profiles uncovers novel gene fusions spanning multiple human cancer types. PLoS Genet. 2013;9(4):e1003464. doi: 10.1371/journal.pgen.1003464.

Girotti MR, Pedersen M, Sanchez-Laorden B, et al. Inhibiting EGF receptor or SRC family kinase signaling overcomes BRAF inhibitor resistance in melanoma. Cancer Discov. 2013;3(2):158-67. doi: 10.1158/2159-8290.CD-12-0386.

Goto K, Yang JC, Kim DW, et al. Phase II study of crizotinib in East Asian patients (pts) with ROS1-positive advanced non-small cell lung cancer (NSCLC). J Clin Oncol. 2016;34(suppl):Abstract 9022.

Greco A, Miranda C, Pierotti MA. Rearrangements of NTRK1 gene in papillary thyroid carcinoma. Mol Cell Endocrinol. 2010;321(1):44-9. doi: 10.1016/j.mce.2009.10.009.

Gu TL, Deng X, Huang F, et al. Survey of tyrosine kinase signaling reveals ROS kinase fusions in human cholangiocarcinoma. PLoS One. 2011;6(1):e15640. doi: 10.1371/journal.pone.0015640.

ICH S10 Tripartite Guideline. Photosafety Evaluation of Pharmaceuticals. Step 4 version. 13 November 2013

Isozaki H, Ichihara E, Takigawa N, et al. Non-Small Cell Lung Cancer Cells Acquire Resistance to the ALK Inhibitor Alectinib by Activating Alternative Receptor Tyrosine Kinases. Cancer Res. 2016;76(6):1506-16. doi: 10.1158/0008-5472.CAN-15-1010.

Ji JH, Oh YL, Hong M, et al. Identification of Driving ALK Fusion Genes and Genomic Landscape of Medullary Thyroid Cancer. PLoS Genet. 2015;11(8):e1005467. doi: 10.1371/journal.pgen.1005467.

Jokinen E, Laurila N, Koivunen P, et al. Combining targeted drugs to overcome and prevent resistance of solid cancers with some stem-like cell features. Oncotarget. 2014;5(19):9295-07. doi: 10.18632/oncotarget.2424.

Kanda R, Kawahara A, Watari K, et al. Erlotinib resistance in lung cancer cells mediated by integrin beta1/Src/Akt-driven bypass signaling. Cancer Res. 2013;73(20):6243-53. doi: 10.1158/0008-5472.CAN-12-4502.

Kang Y, Hu W, Ivan C, et al. Role of focal adhesion kinase in regulating YB-1-mediated paclitaxel resistance in ovarian cancer. J Natl Cancer Inst. 2013;105(19):1485-95. doi: 10.1093/jnci/djt210.

Kaplan DR, Hempstead BL, Martin-Zanca D, et al. The trk proto-oncogene product: a signal transducing receptor for nerve growth factor. Science. 1991;252(5005):554-8.

Kazandjian D, Blumenthal GM, Luo L, et al. Benefit-Risk Summary of Crizotinib for the Treatment of Patients With ROS1 Alteration-Positive, Metastatic Non-Small Cell Lung Cancer. Oncologist. 2016;21(8):974-80. doi: 10.1634/theoncologist.2016-0101.

Kazandjian D, Blumenthal GM, Chen HY, et al. FDA approval summary: crizotinib for the treatment of metastatic non-small cell lung cancer with anaplastic lymphoma kinase rearrangements. Oncologist. 2014;19(10):e5-11. doi: 10.1634/theoncologist.2014-0241.

Khozin S, Blumenthal GM, Zhang L, et al. FDA approval: ceritinib for the treatment of metastatic anaplastic lymphoma kinase-positive non-small cell lung cancer. Clin Cancer Res. 2015;21(11):2436-9. doi: 10.1158/1078-0432.CCR-14-3157.

Kim D-W, Tiseo M, Ahn MJ, et al. Brigatinib (BRG) in patients (pts) with crizotinib (CRZ)-refractory ALK+ non-small cell lung cancer (NSCLC): First report of efficacy and safety from a pivotal randomized phase (ph) 2 trial (ALTA). J Clin Oncol. 2016a;34(suppl):Abstract 9007.

Kim DW, Mehra R, Tan DSW, et al. Activity and safety of ceritinib in patients with ALK-rearranged non-small-cell lung cancer (ASCEND-1): updated results from the multicentre, open-label, phase 1 trial. Lancet Oncol. 2016b;17(4):452-63. doi: 10.1016/S1470-2045(15)00614-2.

Kim J, Lee Y, Cho HJ, et al. NTRK1 fusion in glioblastoma multiforme. PLoS One. 2014;9(3):e91940. doi: 10.1371/journal.pone.0091940.

Klein R, Jing SQ, Nanduri V, et al. The trk proto-oncogene encodes a receptor for nerve growth factor. Cell. 1991;65(1):189-97.

Knezevich SR, Garnett MJ, Pysher TJ, et al. ETV6-NTRK3 Gene Fusions and Trisomy 11 Establish a Histogenetic Link between Mesoblastic Nephroma and Congenital Fibrosarcoma. Cancer Research. 1998;58(22):5046-8.

Koivunen JP, Mermel C, Zejnullahu K, et al. EML4-ALK fusion gene and efficacy of an ALK kinase inhibitor in lung cancer. Clin Cancer Res. 2008;14(13):4275-83. doi: 10.1158/1078-0432.CCR-08-0168.

Kris MG, Johnson BE, Berry LD, et al. Using multiplexed assays of oncogenic drivers in lung cancers to select targeted drugs. JAMA. 2014;311(19):1998-2006. doi: 10.1001/jama.2014.3741.

Lamballe F, Klein R, Barbacid M. trkC, a new member of the trk family of tyrosine protein kinases, is a receptor for neurotrophin-3. Cell. 1991;66(5):967-79.Lasham A, Print CG, Woolley AG, et al. YB-1: oncoprotein, prognostic marker and therapeutic target? Biochem J. 2013;449(1):11-23. doi: 10.1042/BJ20121323.

Lee J, Borboa AK, Chun HB, et al. Conditional deletion of the focal adhesion kinase FAK alters remodeling of the blood-brain barrier in glioma. Cancer Res. 2010;70(24):10131-40. doi: 10.1158/0008-5472.CAN-10-2740.

Lee J, Lee SE, Kang SY, et al. Identification of ROS1 rearrangement in gastric adenocarcinoma. Cancer. 2013;119(9):1627-35. doi: 10.1002/cncr.27967.

Lee J, Kim HC, Hong JY, et al. Detection of novel and potentially actionable anaplastic lymphoma kinase (ALK) rearrangement in colorectal adenocarcinoma by immunohistochemistry screening. Oncotarget. 2015;6(27):24320-32. doi: 10.18632/oncotarget.4462.

Lin E, Li L, Guan Y, et al. Exon array profiling detects EML4-ALK fusion in breast, colorectal, and non-small cell lung cancers. Mol Cancer Res. 2009;7(9):1466-76. doi: 10.1158/1541-7786.MCR-08-0522.

Lipson D, Capelletti M, Yelensky R, et al. Identification of new ALK and RET gene fusions from colorectal and lung cancer biopsies. Nat Med. 2012;18(3):382-4. doi: 10.1038/nm.2673.

Lovly CM, Gupta A, Lipson D, et al. Inflammatory myofibroblastic tumors harbor multiple potentially actionable kinase fusions. Cancer Discov. 2014;4(8):889-95. doi: 10.1158/2159-8290.CD-14-0377.

Lu S, Mok T, Lu Y, et al. Phase 3 study of first-line crizotinib vs pemetrexed–cisplatin/carboplatin in East Asian patients with ALK+ advanced non-squamous non-small cell lung cancer. J Clin Oncol. 2016;34(suppl):Abstract 9058. doi: 10.1200/JCO.2016.34.15_suppl.9058.

Malik SM, Maher VE, Bijwaard KE, et al. U.S. Food and Drug Administration approval: crizotinib for treatment of advanced or metastatic non-small cell lung cancer that is anaplastic lymphoma kinase positive. Clin Cancer Res. 2014;20(8):2029-34. doi: 10.1158/1078-0432.Ccr-13-3077.

Mariño-Enriquez A, Ou WB, Weldon CB, et al. ALK rearrangement in sickle cell trait-associated renal medullary carcinoma. Genes Chromosomes Cancer. 2011;50(3):146-53. doi: 10.1002/gcc.20839.

Martin-Zanca D, Hughes SH, Barbacid M. A human oncogene formed by the fusion of truncated tropomyosin and protein tyrosine kinase sequences. Nature. 1986;319(6056):743-8. doi: 10.1038/319743a0.

National Comprehensive Cancer Network. 2022. *Non-small Cell Lung Cancer*

Osoba D, Zee B. Completion rates in health-related quality-of-life assessment: approach of the National Cancer Institute of Canada Clinical Trials Group. Stat Med. 1998;17(5-7):603-12.

Ou SH, Tan J, Yen Y, et al. ROS1 as a 'druggable' receptor tyrosine kinase: lessons learned from inhibiting the ALK pathway. Expert Rev Anticancer Ther. 2012;12(4):447-56. doi: 10.1586/era.12.17.

Ou SH, Janne PA, Bartlett CH, et al. Clinical benefit of continuing ALK inhibition with crizotinib beyond initial disease progression in patients with advanced ALK-positive NSCLC. Ann Oncol. 2014;25(2):415-22. doi: 10.1093/annonc/mdt572.

Ou SH, Ahn JS, De Petris L, et al. Alectinib in Crizotinib-Refractory ALK-Rearranged Non-Small-Cell Lung Cancer: A Phase II Global Study. J Clin Oncol. 2016;34(7):661-8. doi: 10.1200/jco.2016.34.4_suppl.661.

Ou SH, Zhu VW. CNS metastasis in ROS1+ NSCLC: An urgent call to action, to understand, and to overcome. Lung Cancer. 2019;130:201-207. doi:10.1016/j.lungcan.2019.02.025

Park K, Tan EH, O'Byrne K, et al. Afatinib versus gefitinib as first-line treatment of patients with EGFR mutation-positive non-small-cell lung cancer (LUX-Lung 7): a phase 2B, open-label, randomised controlled trial. Lancet Oncol. 2016;17(5):577-89. doi: 10.1016/s1470-2045(16)30033-x.

Perot G, Soubeyran I, Ribeiro A, et al. Identification of a recurrent STRN/ALK fusion in thyroid carcinomas. PLoS One. 2014;9(1):e87170. doi: 10.1371/journal.pone.0087170.

Planchard D, Popat S, Kerr K, Novello S, Smit EF, Faivre-Finn C, Mok TS, Reck M, Van Schil PE, Hellmann MD, Peters S; ESMO Guidelines Committee. Metastatic non-small cell lung cancer: ESMO Clinical Practice Guidelines for diagnosis, treatment and follow-up. Ann Oncol. 2018 Oct 1;29(Suppl 4):iv192-iv237. doi: 10.1093/annonc/mdy275. Erratum in: Ann Oncol. 2019 May;30(5):863-870. PMID: 30285222.

Rami-Porta R, Asamura H, Travis WD, Rusch VW. Lung cancer - major changes in the American Joint Committee on Cancer eighth edition cancer staging manual. CA Cancer J Clin. 2017;67(2):138-155. doi:10.3322/caac.21390.

Rikova K, Guo A, Zeng Q, et al. Global survey of phosphotyrosine signaling identifies oncogenic kinases in lung cancer. Cell. 2007;131(6):1190-203. doi: 10.1016/j.cell.2007.11.025.

Rosell R, Carcereny E, Gervais R, et al. Erlotinib versus standard chemotherapy as first-line treatment for European patients with advanced EGFR mutation-positive non-small-cell lung cancer (EURTAC): a multicentre, open-label, randomised phase 3 trial. Lancet Oncol. 2012;13(3):239-46. doi: 10.1016/s1470-2045(11)70393-x.

Ross JS, Wang K, Gay L, et al. New routes to targeted therapy of intrahepatic cholangiocarcinomas revealed by next-generation sequencing. Oncologist. 2014;19(3):235-42. doi: 10.1634/theoncologist.2013-0352.

Rozlytrek[TM] [Entrectinib]. Package Insert. San Francisco, CA Genentech Inc., 2019.

Russo M, Misale S, Wei G, et al. Acquired Resistance to the TRK Inhibitor Entrectinib in Colorectal Cancer. Cancer Discov. 2016;6(1):36-44. doi: 10.1158/2159-8290.CD-15-0940.

Sartore-Bianchi A, Ardini E, Bosotti R, et al. Sensitivity to Entrectinib Associated With a Novel LMNA-NTRK1 Gene Fusion in Metastatic Colorectal Cancer. J Natl Cancer Inst. 2016;108(1). doi: 10.1093/jnci/djv306.

Schuler M, Wu YL, Hirsh V, et al. First-Line Afatinib versus Chemotherapy in Patients with Non-Small Cell Lung Cancer and Common Epidermal Growth Factor Receptor Gene Mutations and Brain Metastases. J Thorac Oncol. 2016;11(3):380-90. doi: 10.1016/j.jtho.2015.11.014.

Seguin L, Desgrosellier JS, Weis SM, et al. Integrins and cancer: regulators of cancer stemness, metastasis, and drug resistance. Trends Cell Biol. 2015;25(4):234-40. doi: 10.1016/j.tcb.2014.12.006.

Serrels A, Lund T, Serrels B, et al. Nuclear FAK controls chemokine transcription, Tregs, and evasion of anti-tumor immunity. Cell. 2015;163(1):160-73. doi: 10.1016/j.cell.2015.09.001.

Shaw AT, Hsu PP, Awad MM, et al. Tyrosine kinase gene rearrangements in epithelial malignancies. Nat Rev Cancer. 2013a;13(11):772-87. doi: 10.1038/nrc3612.

Shaw AT, Gandhi L, Gadgeel S, et al. Alectinib in ALK-positive, crizotinib-resistant, non-small-cell lung cancer: a single-group, multicentre, phase 2 trial. Lancet Oncol. 2016a;17(2):234-42. doi: 10.1016/S1470-2045(15)00488-X.

Shaw AT, Kim DW, Mehra R, et al. Ceritinib in ALK-rearranged non-small-cell lung cancer. N Engl J Med. 2014a;370(13):1189-97. doi: 10.1056/NEJMoa1311107.

Shaw AT, Ou SH, Bang YJ, et al. Crizotinib in ROS1-rearranged non-small-cell lung cancer. N Engl J Med. 2014b;371(21):1963-71. doi: 10.1056/NEJMoa1406766.

Shaw AT, Kim DW, Nakagawa K, et al. Crizotinib versus chemotherapy in advanced ALK-positive lung cancer. N Engl J Med. 2013b;368(25):2385-94. doi: 10.1056/NEJMoa1214886.

Shaw AT, Friboulet L, Leshchiner I, et al. Resensitization to Crizotinib by the Lorlatinib ALK Resistance Mutation L1198F. N Engl J Med. 2016b;374(1):54-61. doi: 10.1056/NEJMoa1508887.

Simon R. Optimal two-stage designs for phase II clinical trials. Control Clin Trials. 1989;10(1):1-10.

Skálová A, Vanecek T, Sima R, et al. Mammary analogue secretory carcinoma of salivary glands, containing the ETV6-NTRK3 fusion gene: a hitherto undescribed salivary gland tumor entity. Am J Surg Pathol. 2010;34(5):599-608. doi: 10.1097/PAS.0b013e3181d9efcc.

Soda M, Choi YL, Enomoto M, et al. Identification of the transforming EML4-ALK fusion gene in non-small-cell lung cancer. Nature. 2007;448(7153):561-6. doi: 10.1038/nature05945.

Solomon BJ, Cappuzzo F, Felip E, et al. Intracranial Efficacy of Crizotinib Versus Chemotherapy in Patients With Advanced ALK-Positive Non-Small-Cell Lung Cancer: Results From PROFILE 1014. J Clin Oncol. 2016;34(24):2858-65. doi: 10.1200/JCO.2015.63.5888.

Solomon BJ, Mok T, Kim DW, et al. First-line crizotinib versus chemotherapy in ALK-positive lung cancer. N Engl J Med. 2014;371(23):2167-77. doi: 10.1056/NEJMoa1408440.

Approved v2.0 930210036 2.0

Song A, Kim TM, Kim DW, et al. Molecular Changes Associated with Acquired Resistance to Crizotinib in ROS1-Rearranged Non-Small Cell Lung Cancer. Clin Cancer Res. 2015;21(10):2379-87. doi: 10.1158/1078-0432.CCR-14-1350.

Squinto SP, Stitt TN, Aldrich TH, et al. trkB encodes a functional receptor for brain-derived neurotrophic factor and neurotrophin-3 but not nerve growth factor. Cell. 1991;65(5):885-93.

Stransky N, Cerami E, Schalm S, et al. The landscape of kinase fusions in cancer. Nat Commun. 2014;5:4846. doi: 10.1038/ncomms5846.

Stratford AL, Habibi G, Astanehe A, et al. Epidermal growth factor receptor (EGFR) is transcriptionally induced by the Y-box binding protein-1 (YB-1) and can be inhibited with Iressa in basal-like breast cancer, providing a potential target for therapy. Breast Cancer Res. 2007;9(5):R61. doi: 10.1186/bcr1767.

Taxotere® [docetaxel] Package Insert, Bridgewater, NJ; sanofi-aventis U.S. LLC, 2019.

Thakur R, Trivedi R, Rastogi N, et al. Inhibition of STAT3, FAK and Src mediated signaling reduces cancer stem cell load, tumorigenic potential and metastasis in breast cancer. Sci Rep. 2015;5:10194. doi: 10.1038/srep10194.

Tognon C, Knezevich SR, Huntsman D, Ret al. Expression of the ETV6-NTRK3 gene fusion as a primary event in human secretory breast carcinoma. Cancer Cell. 2002;2(5):367-76.

Torre LA, Bray F, Siegel RL, et al. Global cancer statistics, 2012. CA Cancer J Clin. 2015;65(2):87-108. doi: 10.3322/caac.21262.

Vaishnavi A, Le AT, Doebele RC. TRKing down an old oncogene in a new era of targeted therapy. Cancer Discov. 2015;5(1):25-34. doi: 10.1158/2159-8290.CD-14-0765.

Vaishnavi A, Capelletti M, Le AT, et al. Oncogenic and drug-sensitive NTRK1 rearrangements in lung cancer. Nat Med. 2013;19(11):1469-72. doi: 10.1038/nm.3352.

Vitrakvi [Larotrectinib sulfate] Package Insert. Whippany, NJ. Bayer Healthcare. 2022.

Voena C, Varesio LM, Zhang L, et al. Oncogenic ALK regulates EMT in non-small cell lung carcinoma through repression of the epithelial splicing regulatory protein 1. Oncotarget. 2016;7(22):33316-30. doi: 10.18632/oncotarget.8955.

Wiesner T, He J, Yelensky R, et al. Kinase fusions are frequent in Spitz tumours and spitzoid melanomas. Nat Commun. 2014;5:3116. doi: 10.1038/ncomms4116.

Wilson C, Nicholes K, Bustos D, et al. Overcoming EMT-associated resistance to anti-cancer drugs via Src/FAK pathway inhibition. Oncotarget. 2014;5(17):7328-41. doi: 10.18632/oncotarget.2397.

Wu G, Diaz AK, Paugh BS, et al. The genomic landscape of diffuse intrinsic pontine glioma and pediatric non-brainstem high-grade glioma. Nat Genet. 2014;46(5):444-50. doi: 10.1038/ng.2938.

Xalkori® [package insert]. Pfizer, 2019.

Yamamoto H, Yoshida A, Taguchi K, et al. ALK, ROS1 and NTRK3 gene rearrangements in inflammatory myofibroblastic tumours. Histopathology. 2016;69(1):72-83. doi: 10.1111/his.12910.

Ying J, Lin C, Wu J, et al. Anaplastic Lymphoma Kinase Rearrangement in Digestive Tract Cancer: Implication for Targeted Therapy in Chinese Population. PLoS One. 2015;10(12):e0144731. doi: 10.1371/journal.pone.0144731.

Yoshida R, Okumura S, Sasaki T, et al. Src mediates acquired resistance to ALK inhibitor in ALK-rearranged non-small cell lung cancers. Cancer Res. 2016;76((14 Suppl)):Abstract nr 2121.

Zhang L, Jiang T, Zhao C, Li W, Li X, Zhao S, Liu X, Jia Y, Yang H, Ren S, Zhou C. Efficacy of crizotinib and pemetrexed-based chemotherapy in Chinese NSCLC patients with ROS1 rearrangement. Oncotarget. 2016 Nov 15;7(46):75145-75154. doi: 10.18632/oncotarget.12612. PMID: 27738334; PMCID: PMC5342729.

Zhang S, Yu D. Targeting Src family kinases in anti-cancer therapies: turning promise into triumph. Trends in pharmacological sciences. 2012;33(3):122-8. doi: 10.1016/j.tips.2011.11.002.

Zhang S, Huang W-C, Zhang L, et al. SRC family kinases as novel therapeutic targets to treat breast cancer brain metastases. Cancer Research. 2013;73(18):5764-74. doi: 10.1158/0008-5472.CAN-12-1803.

Zhang S, Huang WC, Li P, et al. Combating trastuzumab resistance by targeting SRC, a common node downstream of multiple resistance pathways. Nat Med. 2011;17(4):461-9. doi: 10.1038/nm.2309.

Zheng Z, Liebers M, Zhelyazkova B, et al. Anchored multiplex PCR for targeted next-generation sequencing. Nat Med. 2014;20(12):1479-84. doi: 10.1038/nm.3729.

APPENDIX 1 CONTRACEPTIVE GUIDELINES

All Phases: WOMEN OF CHILDBEARING POTENTIAL DEFINITIONS AND METHODS OF CONTRACEPTION

This appendix provides general information and definitions related to Women of Childbearing Potential (WOCBP) and methods of contraception that can be applied to most clinical trials. For information specific to this study regarding acceptable contraception requirements for female and male participants, refer to Sections 5.1 and 5.2 of the protocol. Only the contraception methods as described in Sections 5.1 and 5.2 of the protocol are acceptable for this study.

DEFINITIONS

Women of Childbearing Potential (WOCBP)

A woman is considered fertile following menarche and until becoming postmenopausal unless permanently sterile. Permanent sterilization methods include hysterectomy, bilateral salpingectomy, and bilateral oophorectomy.

Women in the following categories are not considered WOCBP:

- Premenarchal
- Pre-menopausal female with 1 of the following:
  - Documented hysterectomy
  - Documented bilateral salpingectomy
  - Documented bilateral oophorectomy
- Note: Documentation can come from the site personnel's review of the participant's medical records, medical examination, or medical history interview.
- Post-menopausal female
  - A post-menopausal state is defined as 12 months of amenorrhea in a woman over the age of 45 years in the absence of other biological or physiological causes. In addition, females under the age of 55 years must have a serum follicle-stimulating hormone (FSH) level > 40 mIU/mL to confirm menopause.

Note: Females treated with hormone replacement therapy (HRT) are likely to have artificially suppressed FSH levels and may require a washout period to obtain a physiologic FSH level. The duration of the washout period is a function of the type of HRT used. Suggested guidelines for the duration of the washout periods for HRT types are presented below. Investigators should use their judgment in checking serum FSH levels.

- 1-week minimum for vaginal hormonal products (rings, creams, gels)
- 4-week minimum for transdermal products
- 8-week minimum for oral products

Other parenteral products may require washout periods as long as 6 months. If the serum FSH level is > 40 mIU/mL at any time during the washout period, the woman can be considered postmenopausal.

METHODS OF CONTRACEPTION

Local laws and regulations may require use of alternative and/or additional contraception methods.

**Highly Effective Contraceptive Methods That Are <u>User Dependent</u>**<br>*Failure rate of < 1% per year when used consistently and correctly.*[a]
• Combined (estrogen- and progestogen-containing) hormonal contraception associated with inhibition of ovulation and/or implantation. (This method of contraception can only be used by WOCBP participants in studies in which hormonal contraception is permitted by the study protocol.)[b]<br>– Oral (birth control pills)<br>– Intravaginal (rings)<br>– Transdermal<br>• Combined (estrogen- and progestogen-containing) hormonal contraception must begin at least 7 days prior to initiation of study therapy.
• Progestogen-only hormonal contraception associated with inhibition of ovulation. (This method of contraception can only be used by WOCBP participants in studies in which hormonal contraception is permitted by the study protocol.)[b]<br>– Oral<br>– Injectable<br>• Progestogen-only hormonal contraception must begin at least 7 days prior to initiation of study therapy
**Highly Effective Methods That Are User Independent**
• Implantable progestogen-only hormonal contraception associated with inhibition of ovulation and/or implantation. (This method of contraception can only be used by WOCBP participants in studies in which hormonal contraception is permitted by the study protocol.)[b]<br>• Intrauterine device.<br>• Intrauterine system (IUS). (This method of contraception can only be used by WOCBP participants in studies in which hormonal contraception is permitted by the study protocol.)[b,c]<br>• Bilateral tubal occlusion
• Vasectomized partner.<br>• Having a vasectomized partner is a highly effective contraception method provided that the partner is the sole male sexual partner of the WOCBP and the absence of sperm has been confirmed. If not, an additional highly effective method of contraception should be used.<br>• Male participants will be required to always use a latex or other synthetic condom during any sexual activity (eg, vaginal, anal, oral) with WOCBP, even if the participants have undergone a successful vasectomy or if their partner is pregnant or breastfeeding.
• Sexual abstinence.

Sexual abstinence is considered a highly effective method only if defined as refraining from heterosexual intercourse during the entire period of risk associated with the study intervention. The reliability of sexual abstinence needs to be evaluated in relation to the duration of the study and the preferred and usual lifestyle of the participant.

- Continuous abstinence must begin at least 30 days prior to initiation of study therapy.
- It is not necessary to use any other method of contraception when complete abstinence is elected.
- WOCBP participants who choose complete abstinence must continue to have pregnancy tests, as specified in the protocol.
- Acceptable alternate methods of highly effective contraception must be discussed in the event that the WOCBP participant chooses to forego complete abstinence.
- Periodic abstinence (including, but not limited to, calendar, symptothermal, post-ovulation methods), withdrawal (coitus interruptus), spermicides only, and lactation amenorrhea method (LAM) are not acceptable methods of contraception for this study

Less Than Highly Effective Contraceptive Methods That Are User Dependent

Failure rate of > 1% per year when used consistently and correctly.

- Male or female condom with or without spermicide. Male and female condoms cannot be used simultaneously.
- Diaphragm with spermicide.
- Cervical cap with spermicide.
- Vaginal sponge with spermicide.
- Progestogen-only oral hormonal contraception, where inhibition of ovulation is not the primary mechanism of action. (This method of contraception cannot be used by WOCBP participants in studies in which hormonal contraception is prohibited.)

Unacceptable Methods of Contraception

- Periodic abstinence (calendar, symptothermal, post-ovulation methods)
- Withdrawal (coitus interruptus)
- Spermicide only
- LAM

a Typical use failure rates may differ from failure rates when contraceptive methods are used consistently and correctly. Use should be consistent with local regulations regarding the use of contraceptive methods for participants in clinical studies.

b Due to a potential loss of effectiveness of hormonal contraceptives caused by interaction with study intervention, if WOCBP use hormonal contraceptives (including oral hormonal contraceptives), they must use either another form of non-hormonal highly effective contraception or a reliable barrier method.

c IUSs are acceptable methods of contraception in the absence of definitive drug interaction studies when hormone exposures from intrauterine devices do not alter contraception effectiveness.

COLLECTION OF PREGNANCY INFORMATION

Guidance for collection of pregnancy information and outcome of pregnancy on the Pregnancy Surveillance Form is provided in Section 9.3.1 and Section 9.3.2.

APPENDIX 2 PHASE 1 REQUIRED LABORATORY ASSESSMENTS

Phase 1 Dose Escalation					
**Hematology**	**Chemistry[a]**	**Coagulation**	**Urinalysis**	**Pregnancy Test**	**Hypogonadism blood sampling [b]**
Hemoglobin and Hematocrit	Alanine aminotransferase	Prothrombin time or international normalized ratio	Urine analysis for urine protein	Serum from female subjects of childbearing potential	Total testosterone
Platelets	Aspartate aminotransferase	Partial thromboplastin time	Urine analysis for urine blood		Sex hormone-binding globulin
White blood cell count	Alkaline phosphatase		Urine analysis for urine glucose		Luteinizing hormone
Absolute neutrophils	Sodium		Urine analysis for urine ketones		Follicle-stimulating hormone
Absolute lymphocytes	Potassium		Specific gravity		Prolactin
Absolute monocytes	Magnesium				
Absolute eosinophils	Bicarbonate or CO2				
Absolute basophils	Creatine phosphokinase				
Absolute or % reticulocytes	Lactate Dehydrogenase				
	Total bilirubin				
	Blood urea nitrogen				
	Creatinine				
	Uric acid				
	Glucose (non-fasted)				
	Total Protein				
	Albumin				
	Phosphorus				

Phase 1 Dose Escalation					
**Hematology**	**Chemistry[a]**	**Coagulation**	**Urinalysis**	**Pregnancy Test**	**Hypogonadism blood sampling [b]**
	Calcium				
	Total calcium				
	Chloride				
	Cardiac troponin				
	Serum amylase				
	Serum lipase				

[a] For Hy's Law cases, in addition to repeating AST and ALT, laboratory tests should include albumin, creatine phosphokinase (CPK), total bilirubin, direct and indirect bilirubin, gamma-glutamyl transferase, prothrombin time (PT)/INR, and alkaline phosphatase

[b] Hypogonadism blood sampling between 08:00-11:00 in MALE subjects only

APPENDIX 3 PHASE 2 REQUIRED LABORATORY ASSESSMENTS

Phase 2 Dose Escalation					
**Hematology**	**Chemistry[a]**	**Coagulation**	**Urinalysis**	**Pregnancy Test**	**Hypogonadism blood sampling [b]**
Hemoglobin	Alanine aminotransferase	PT or INR	Urine analysis for urine protein	Serum from female subjects of childbearing potential	Total testosterone (TT)
Platelets	Aspartate aminotransferase	PTT	Urine analysis for urine blood		Sex hormone-binding globulin
White blood cell count	Alkaline phosphatase		Urine analysis for urine glucose		Luteinizing hormone
Absolute neutrophils	Sodium		Urine analysis for urine ketones		Follicle-stimulating hormone (FSH)
Absolute lymphocytes	Potassium		Specific gravity		Prolactin
Absolute monocytes	Magnesium				
Absolute eosinophils	Creatine phosphokinase				
Absolute basophils	Chloride				
Absolute or % reticulocytes	Lactate Dehydrogenase				
	Total bilirubin				
	BUN or urea				
	Creatinine				
	Uric acid				
	Glucose (non-fasted)				
	Total Protein				
	Albumin				
	Phosphorus				

Phase 2 Dose Escalation					
**Hematology**	**Chemistry[a]**	**Coagulation**	**Urinalysis**	**Pregnancy Test**	**Hypogonadism blood sampling [b]**
	Calcium				
	Total calcium				
	Bicarbonate or CO2				
	Cardiac troponin				
	Serum amylase				
	Serum lipase				

[a] For Hy's Law cases, in addition to repeating AST and ALT, laboratory tests should include albumin, creatine phosphokinase (CPK), total bilirubin, direct and indirect bilirubin, gamma-glutamyl transferase, prothrombin time (PT)/INR, and alkaline phosphatase

[b] Hypogonadism blood sampling between 08:00-11:00 in MALE subjects only

APPENDIX 4 SUBJECT-REPORTED OUTCOMES: EORTC-QLQ-C30

We are interested in some things about you and your health. Please answer all of the questions yourself by circling the number that best applies to you. There are no "right" or "wrong" answers. The information that you provide will remain strictly confidential. Note: If not allowed per local regulations, keep initials blank and provide only birth year.

Please fill in your initials:

Your birthdate (Month, Day, Year):

Today's Date (Month, Day Year):

During the past week:	Not at All	A Little	Quite a Bit	Very Much
1. Do you have any trouble doing strenuous activities, like carrying a heavy shopping bag or suitcase?	1	2	3	4
2. Do you have any trouble taking a <u>long</u> walk?	1	2	3	4
3. Do you have any trouble taking a <u>short</u> walk outside of the house?	1	2	3	4
4. Do you need to stay in bed or a chair during the day?	1	2	3	4
5. Do you need help with eating, dressing, washing yourself or using the toilet?	1	2	3	4
**During the Past week:**				
6. Were you limited in doing either your work or other daily activities?	1	2	3	4
7. Were you limited in pursuing your hobbies of other leisure time activities?	1	2	3	4
8. Were you short of breath?	1	2	3	4
9. Have you had pain?	1	2	3	4
10. Did you need to rest?	1	2	3	4
11. Have you had trouble sleeping?	1	2	3	4
12. Have you felt weak?	1	2	3	4
13. Have you lacked appetite?	1	2	3	4
14. Have you felt nauseated?	1	2	3	4
15. Have you vomited?	1	2	3	4
16. Have you been constipated?	1	2	3	4

17. Have you had diarrhea?	1	2	3	4
18. Were you tired?	1	2	3	4
19. Did pain interfere with your daily activities?	1	2	3	4
20. Have you had difficulty in concentrating on things, like reading a newspaper or watching television?	1	2	3	4
21. Did you feel tense?	1	2	3	4
22. Did you worry?	1	2	3	4
23. Did you feel irritable?	1	2	3	4
24. Did you feel depressed?	1	2	3	4
25. Have you had difficulty remembering things?	1	2	3	4
26. Has your physical condition or medical treatment interfered with your family life?	1	2	3	4
27. Has your physical condition or medical treatment interfered with your social life?	1	2	3	4
28. Has your physical condition or medical treatment caused you financial difficulties?	1	2	3	4

For the following questions, please circle the number between 1 and 7 that best applies to you

29. How would you rate your overall health during the past week?

1	2	3	4	5	6	7
Very Poor						Excellent

30. How would you rate your overall quality of life during the past week?

1	2	3	4	5	6	7
Very Poor						Excellent

APPENDIX 5 SUBJECT-REPORTED OUTCOMES: EORTC-QLQ-LC13

Subjects sometimes report that they have the following symptoms or problems. Please indicate the extent to which you have experiences these symptoms or problems <u>during the past week</u>. Please answer by circling the number that best applies to you.

**During the past week:**	**Not at All**	**A Little**	**Quite a Bit**	**Very Much**
1. How much did you cough?	1	2	3	4
2. Did you cough up blood?	1	2	3	4
3. Were you short of breath when you rested?	1	2	3	4
4. Were you short of breath when you walked?	1	2	3	4
5. Were you short of breath when you climbed stairs?	1	2	3	4
6. Have you had a sore mouth or tongue?	1	2	3	4
7. Have you had trouble swallowing?	1	2	3	4
8. Have you had tingling hands or feet?	1	2	3	4
9. Have you had hair loss?	1	2	3	4
10. Have you had pain in your chest?	1	2	3	4
11. Have you had pain in your arm or shoulder?	1	2	3	4
12. Have you had pain in other parts of your body?	1	2	3	4
If yes, where ______________________				
13. Did you take any medicine for pain?				
**1 No** **2 Yes**				
If yes, how much did it help?	1	2	3	4

APPENDIX 6 TABLE OF MEDICATIONS THAT ARE STRONG CYP3A INHIBITORS OR INDUCERS OF CYP3A OR CAUSE QTC PROLONGATION

Strong CYP3A inhibitors	Strong CYP3A inducers
boceprevir	apalutamide
clarithromycin [a]	carbamazepine
cobicistat [a]	enzalutamide
danoprevir and ritonavir [b]	mitotane
elvitegravir and ritonavir [b]	phenytoin
grapefruit juice [c]	rifampin
idelalisib	St John's wort [d]
indinavir and ritonavir [b]	
itraconazole [a]	
ketoconazole	
lopinavir and ritonavir [a, b]	
nefazodone	
nelfinavir [a]	
paritaprevir and ritonavir and (ombitasvir and/or dasabuvir) [b]	
posaconazole	
ritonavir [a, b]	
saquinavir and ritonavir [a, b]	
telaprevir [a]	
telithromycin	
tipranavir and ritonavir [a, b]	
troleandomycin	

**Strong CYP3A inhibitors**		**Strong CYP3A inducers**	
voriconazole			
**Medications that cause QTc prolongation**			
Amiodarone	Citalopram	Ibutilide	Quinidine
Arsenic trioxide	Disopyramide	Levomethadyl	Sotalol
Astemizole	Dofetilide	Mesoridazine	Sparfloxacin
Azithromycin	Domperidone	Methadone	Terfenadine
Bepridil	Droperidol	Moxifloxacin	Thioridazine
Clarithromycin	Erythromycin	Pentamidine	Vavdetanib
Chlorpromazine	Flecainide	Pimozide	
Chloroquine	Halofantrine	Probucol	
Cisapride	Haloperidol	Procainamide	

[a] Inhibitor of P-gp (defined as those increasing AUC of digoxin to ≥ 1.25-fold)

[b] Ritonavir is usually given in combination with other anti-HIV or anti-HCV drugs in clinical practice. Caution should be used when extrapolating the observed effect of ritonavir alone to the effect of combination regimens on CYP3A activities.

[c] The effect of grapefruit juice varies widely among brands and is concentration-, dose-, and preparation-dependent. Studies have shown that it can be classified as a “strong CYP3A inhibitor” when a certain preparation was used (eg, high-dose, double strength) or as a “moderate CYP3A inhibitor” when another preparation was used (eg, low dose, single strength).

[d] The effect of St. John’s wort varies widely and is preparation-dependent.

APPENDIX 7 MIDAZOLAM DRUG-DRUG INTERACTION SUB-STUDY

7.1 Midazolam DDI Sub-study Objectives:

To evaluate the potential effect of repotrectinib on CYP3A induction, a midazolam DDI sub-study will be conducted in 6 PK evaluable subjects after RP2D determination. All subjects participating in the DDI assessment periods of this sub-study will be administered a single 5 mg oral dose of MDZ **alone** on Day -2 under fasted condition (no food 8 hours before through 2 hours after dosing of MDZ), and then starting on Cycle 1 Day 1, subjects will begin daily treatment of 160 mg repotrectinib QD followed by 160 mg BID on Cycle 1 Day 15. On Cycle 1 Day 22, another single dose of 5 mg MDZ will be administered under fasted condition (no food 8 hours before through 2 hours after dosing of MDZ) concurrently with repotrectinib (morning dose). Serial plasma samples will be collected for up to 24 hours following both MDZ dosing periods to assess full MDZ and its metabolite, 1'-OH-MDZ PK profiles. A comparison of MDZ and 1'-OH-MDZ PK profiles on Cycle 1 Day -2 and Cycle 1 Day 22 will then allow an assessment of the potential effect of repotrectinib on CYP3A induction. Repotrectinib full PK will be assessed using serial plasma samples collected on Cycle 1 Day 1 and Day 22, and abbreviated PK samples will be collected on Cycle 1 Day 15, and on Day 1 of Cycles 2, 3 and 4. Once the DDI assessment periods end, patients will continue the study treatment until progression of disease as determined by the Investigator, unacceptable toxicity, or consent withdrawal. Repotrectinib can be taken without regard to food between Cycle 1 Day 1 to Cycle 1 Day 21, as well as after completion of the DDI assessment.

If a subject fails to escalate to 160 mg BID on Cycle 1 Day 15, a second MDZ dose will not be administered, and this subject will be replaced.

7.1.1 Inclusion Criteria for Midazolam DDI Sub-study

Please refer to Section 5.1 for inclusion criteria.

7.1.2 Exclusion criteria for Midazolam DDI Sub-Study:

Please refer to Section 5.3 for exclusion criteria.

7.1.3 Midazolam Administration:

Please refer to Section 6.1.3.2 for administration instruction.

7.2 Concomitant Medications for Patients participating in the Midazolam DDI Sub-study

In addition to the strong CYP3A inhibitors or inducers listed in APPENDIX 6, subjects should not be taking any moderate inhibitors or inducers of CYP3A (moderate CYP3A inhibitors eg: erythromycin, verapamil, atazanavir, fluconazole, darunavir, diltiazem, delavirdine, aprepitant, imatinib, tofisopam, ciprofloxacin, cimetidine; moderate CYP3A inducers eg: bosentan, efavirenz, etravirine, modafinil) within 2 weeks of the lead-in midazolam dosing and until the DDI assessment portions is completed on Cycle 1 Day 23. Please refer to the midazolam product package insert for complete information.

7.3 Blood for PK Analysis of Midazolam/Repotrectinib (Midazolam DDI Sub-Study)

For the determination of midazolam and repotrectinib PK during the midazolam DDI sub-study, serial blood samples of 4.0 mL each will be collected after MDZ dosing on Cycle 1 Day -2 and Cycle 1 Day 22 at the specified time points. Repotrectinib full PK will also be collected on Cycle 1 Day 1 and Day 22 and additional abbreviated PK samples will be collected. A total of approximately 148 mL of blood will be collected from each subject participating in the midazolam DDI sub-study over a 12-week period (Table 36 and Table 37)

Table 36: Time Points of Blood Collection for Midazolam Pharmacokinectic Assessment

PK Collection Number	Sample Number	Cycle	Day	Scheduled Time Points Relative to Previous Dose (hours)	Description
1	1	1	-2	0 hr[a]	Pre-dose
1	2	1	-2	0.5 hr	Post-dose
1	3	1	-2	1 hr	Post-dose
1	4	1	-2	2 hr	Post-dose
1	5	1	-2	4 hr	Post-dose
1	6	1	-2	6 hr	Post-dose
1	7	1	-2	8 hr	Post-dose
1	8	1	-1	24 hr	Post-dose
2	9	1	22	0 hr[a]	Pre-dose
2	10	1	22	0.5 hr	Post-dose
2	11	1	22	1 hr	Post-dose
2	12	1	22	2 hr	Post-dose
2	13	1	22	4 hr	Post-dose
2	14	1	22	6 hr	Post-dose
2	15	1	22	8 hr	Post-dose
2	16	1	23	24 hr	Post-dose

All measurement times are relative to dose of midazolam unless otherwise specified. Samples obtained within 10% of the nominal time (eg, within 6 minutes of the 60-minute sample) from dosing will not be considered as a protocol deviation, as long as the exact time of the sample collection is noted on the source document and data collection tool (eg, CRF).

[a] Take PK sample immediately prior to the administration of midazolam

Table 37: Time Points of Blood Collection for Repotrectinib PK Assessment (Midazolam DDI Sub-study)

PK Collection Number	Sample Number	Cycle	Day	Scheduled Time Points Relative to Previous Dose (hours)	Description
1	1	1	1	0 hr[a]	Pre-dose
1	2	1	1	1 hr	Post-dose
1	3	1	1	2 hr	Post-dose
1	4	1	1	4 hr	Post-dose
1	5	1	1	6 hr	Post-dose
1	6	1	1	8 hr	Post-dose
1	7	1	2	24 hr	Post-dose
2	8	1	15	0 hr[a]	Pre-dose
3	9	1	22	0 hr[a]	Pre-dose
3	10	1	22	1 hr	Post-dose
3	11	1	22	2 hr	Post-dose
3	12	1	22	4 hr	Post-dose
3	13	1	22	6 hr	Post-dose
3	14	1	22	8 hr	Post-dose
3	15	1	23	24 hr	Post-dose
4	16	2	1	0 hr[a]	Pre-dose
4	17	2	1	4 hr	Post-dose
5	18	3	1	0 hr[a]	Pre-dose
5	19	3	1	4 hr	Post-dose
6	20	4	1	0 hr[a]	Pre-dose
6	21	4	1	4 hr	Post-dose

All measurement times are relative to dose of midazolam unless otherwise specified. Samples obtained within 10% of the nominal time (eg, within 6 minutes of the 60-minute sample) from dosing will not be considered as a protocol deviation, as long as the exact time of the sample collection is noted on the source document and data collection tool (eg, CRF).

[a] Take PK sample immediately prior to the administration of midazolam

7.4 Midazolam DDI Sub-study Assessments

7.4.1 Screening Midazolam DDI sub-study (within 28 days of the first dose of midazolam)

- Obtain Informed Consent
- Eligibility checklist (see complete list in Section 5.1 and Section 5.3)
- Perform molecular testing as per Section 5.1 and Table 38:

- Molecular testing to determine eligibility can be performed in advance with no time limit (eg, the subject can be tested while being treated on another anticancer therapy)
- Source documents (molecular pathology report detailing the specific test that detect the *ALK*, *ROS1*, or *NTRK* rearrangement) should be submitted for the Sponsor approval of eligibility (if multiple reports are available, submit them all to ensure a clear understanding of the history of tumor progression)
- Submitted archival tissues to Sponsor's designated central laboratory to retrospectively confirm the specific molecular alteration
- An optional tumor tissue re-biopsy (for subjects who have failed prior TKIs) during the screening period to investigate resistance mechanism(s) is recommended but not mandatory

- Obtain detailed treatment history
  - Number of prior TKIs
  - Duration of treatment of each prior TKI
  - Investigator assessment of best response to each prior TKI
  - Number of prior chemotherapy regimens
  - Duration of treatment of each chemotherapy regimen
  - Investigator assessment of best response to each prior chemotherapy regimen
  - Number of prior immunotherapy regimens
  - Duration of treatment of each immunotherapy regimen
  - Investigator assessment of best response to each prior immunotherapy regimen
  - For subjects with prior treated brain metastases, record the method of radiation: whole brain radiation, stereotactic radiosurgery, or both. Record also the dates of the radiation treatment(s), total dose delivered to each course of treatment, and date of last radiation to the start of repotrectinib treatment
- Obtain detailed medical history
- Perform a complete physical examination
- Perform and record vital signs, height, body weight, pain level (0-10), and ECOG PS
- Review concomitant medications
- Female subjects with childbearing potential: Perform a serum pregnancy test
- Collect blood and urine for clinical laboratory assessments and tumor markers
- Collect blood for ccfDNA
- Obtain echocardiogram or MUGA for baseline LVEF assessment
- Perform and record triplicate ECGs:
  - **All subjects**: Triplicate 12-lead ECGs obtain approximately 2 minutes apart
- Obtain cardiac troponin-I or troponin-T test
- Obtain baseline tumor assessment including CT chest, abdomen, and pelvis, MRI of the brain and bone scan (if applicable)
- Subject registration

7.4.2 Cycle 1 Day -2

- Subjects to come to the clinic visit overnight fasting (8 hours) prior to dosing of midazolam
- Perform complete physical examination
- Perform and record vital signs, body weight, pain level (0-10), and ECOG PS
- Collect a midazolam pre-dose PK sample
- Administer oral 5 mg dose of midazolam in the AM. No food will be allowed within 2 hours of midazolam dosing
- Collect midazolam PK samples at 0.5, 1, 2, 4, 6, and 8 hours post-dose
- Record any adverse events

7.4.3 Cycle 1 Day 1

- Perform a complete physical examination
- Perform and record vital signs, body weight, pain level (0-10), and ECOG PS
- Review medical history
- Review concomitant medications
- Female subjects with childbearing potential: Perform a serum pregnancy test
- Collect blood and urine for clinical laboratory assessments
- Perform and record pre-dose ECGs:
  - **All subjects**: triplicate 12-lead ECGs approximately 2 minutes apart
- Collect a repotrectinib pre-dose PK sample
- Administer repotrectinib
- Collect repotrectinib PK samples at 1, 2, 4, 6, 8, and 24 hours post-dose. Prior PK sample collection, record triplicate 12-lead ECGs approximately 2 minutes apart
- Record any adverse events
- Dispense study medication for the next cycle (28 days) of treatment

7.4.4 Cycle 1 Day 8

- Perform a complete physical examination
- Perform and record vital signs, body weight, pain level (0-10), and ECOG PS
- Review medical history
- Assess study drug compliance
- Review concomitant medications
- Record any adverse events
- Collect blood and urine for clinical laboratory assessments
- Administer repotrectinib

7.4.5 Cycle 1 Day 15

- Perform a complete physical examination
- Perform and record vital signs, body weight, pain level (0-10), and ECOG PS
- Review medical history
- Assess study drug compliance
- Review concomitant medications
- Record any adverse events
- Collect blood and urine for clinical laboratory assessments
- Perform and record pre-dose ECGs:
  - **All subjects:** Triplicate 12-lead ECGs approximately 2 minutes apart
- Collect a repotrectinib pre-dose PK sample
- Administer repotrectinib

7.4.6 Cycle 1 Day 22

- Subjects to come to the clinic visit overnight fasting (8 hours) prior to dosing of midazolam
- Perform a complete physical examination
- Perform and record vital signs, body weight, pain level (0-10), and ECOG PS
- Review medical history
- Assess study drug compliance
- Review concomitant medications
- Record any adverse events
- Collect blood and urine for clinical laboratory assessments
- Perform and record pre-dose ECGs:
  - **All subjects:** Triplicate 12-lead ECGs approximately 2 minutes apart
- Collect a repotrectinib pre-dose PK sample
- Collect a midazolam pre-dose PK sample
- Administer repotrectinib
- Administer 5 mg dose of midazolam immediately following administration of repotrectinib. No food will be allowed within 2 hours of midazolam dosing
- Collect repotrectinib PK samples at 1, 2, 4, 6, 8, and 24 hours post-dose. Prior to PK collection, record triplicate 12-lead ECGs approximately 2 minutes apart
- Collect midazolam PK samples at 0.5, 1, 2, 4, 6, 8, and 24 hours post-dose. Prior to PK sample collection, record triplicate 12-lead ECGs approximately 2 minutes apart

7.4.7 Cycle 2 Day 1

- Perform a complete physical examination
- Perform and record vital signs, body weight, pain level (0-10), and ECOG PS

- Review medical history
- Assess study drug compliance
- Review concomitant medications
- Record any adverse events
- Female **subjects with childbearing potential**: Perform a serum pregnancy test
- Collect blood and urine for clinical laboratory assessments
- Perform and record pre-dose ECGs:
  - **All subjects:** triplicate 12-lead ECGs approximately 2 minutes apart
- Collect a baseline pre-dose PK sample
- Administer repotrectinib
- At 4 hours post-dose, record triplicate 12-lead ECGs approximately 2 minutes apart
- Collect a corresponding 4-hour PK blood sample. Prior to PK collection, record triplicate 12-lead ECGs approximately 2 minutes apart
- Dispense study medication for the next cycle (28 days) of treatment

7.4.8 Cycle 2 Day 15

- Perform a complete physical examination
- Perform and record vital signs, body weight, pain level (0-10), and ECOG PS
- Review medical history
- Assess study drug compliance
- Review concomitant medications
- Record any adverse events
- Collect blood for clinical laboratory assessments

7.4.9 Cycle 3 Day 1

- Perform a complete physical examination
- Perform and record vital signs, body weight, pain level (0-10), and ECOG PS
- Review medical history
- Assess study drug compliance
- Review concomitant medications
- Record any adverse events
- Female subjects with childbearing potential: Perform a serum pregnancy test
- Collect blood and urine for clinical laboratory assessments
- Collect blood for ccfDNA
- Assess radiographic response to repotrectinib
- Schedule the next scans to confirm the responses when a PR or CR is observed (ideally confirmation scan should be scheduled at the end of the next cycle ie, 28 days)

- Collect a baseline pre-dose PK sample
- Perform and record pre-dose ECGs:
  - **All subjects:** triplicate 12-lead ECGs approximately 2 minutes apart
- Administer repotrectinib
- At 4 hours post-dose, record triplicate 12-lead ECGs approximately 2 minutes apart
- Collect a corresponding 4-hour PK blood sample. Prior to PK collection, record triplicate 12-lead ECGs approximately 2 minutes apart
- Dispense study medication for the next cycle (28 days) of treatment
- Obtain echocardiogram or MUGA for LVEF assessment

7.4.10 Cycle 4 Day 1

- Perform a complete physical examination
- Perform and record vital signs, body weight, pain level (0-10), and ECOG PS
- Review medical history
- Assess study drug compliance
- Review concomitant medications
- Record any adverse events
- Female subjects with childbearing potential: Perform a serum pregnancy test
- Obtain echocardiogram or MUGA for LVEF assessment (and beginning of every 3 cycles, Cycle 4, 7, 10, 13)
- Collect blood and urine for clinical laboratory assessments
- If patient is confirmed to have progressed, collect a blood sample (ccfDNA)
- Assess radiographic response to repotrectinib when a PR or CR is observed at Cycle 3 Day 1 evaluation
- Perform and record pre-dose ECGs:
  - **All subjects:** triplicate 12-lead ECGs approximately 2 minutes apart
- Collect a baseline pre-dose PK sample
- Administer repotrectinib
- At 4 hours post-dose, record triplicate 12-lead ECGs approximately 2 minutes apart
- Collect a corresponding 4-hour PK blood sample. Prior to PK collection, record triplicate 12-lead ECGs approximately 2 minutes apart
- Dispense study medication for the next cycle (28 days) of treatment

7.4.11 Cycle 5 and Every 4 Weeks Thereafter at Each Cycle Day 1 Visit

- Perform a complete physical examination
- Perform and record vital signs, body weight, pain level (0-10), and ECOG PS
- Review medical history

- Assess study drug compliance
- Review concomitant medications
- Record any adverse events
- Female subjects with childbearing potential: Perform a serum pregnancy test
- Collect blood and urine for clinical laboratory assessments
- If patient is confirmed to have progressed, collect a blood sample (ccfDNA)
- Assess radiographic response to study drug (at the beginning of every odd number of cycles)
- Schedule the next scans to confirm the responses when a PR or CR is observed (ideally confirmation scan should be scheduled at the end of the next cycle ie, 28 days)
- Obtain echocardiogram or MUGA for LVEF assessment (at the beginning of every 3 cycles, Cycle 7, 10, 13)
- Administer repotrectinib
- Dispense study medication for the next cycle (28 days) of treatment

7.4.12 End of Treatment (within 7 days post last dose of repotrectinib and after decision to end treatment)

- Female subjects with childbearing potential: Perform a serum pregnancy test
- Collect blood and urine for clinical laboratory assessments
- Collect blood for ccfDNA, if EOT is earlier than Cycle 3 Day 1 or was not collected at progression
- Perform and record triplicate ECGs:
  - **All subjects:** Triplicate 12-lead ECGs obtain approximately 2 minutes apart
- Obtain echocardiogram or MUGA for LVEF assessment
- Assess study drug compliance (optional if performed at the time of the last dose)
- The following assessments are optional if performed within the past week:
  - Review concomitant medications
  - Record any adverse events
  - Review medical history
  - Perform complete physical examination
  - Perform and record vital signs, body weight, pain level (0-10), and ECOG PS
  - Perform tumor imaging, submit scans for BICR within 1 week, and assess locally as per RECIST version 1.1 (optional if performed within the past 4 weeks)

7.4.13 Safety Follow-up (approximately 28 days post the last dose of repotrectinib)

- Review concomitant medications
- Female subjects with childbearing potential: Perform a serum pregnancy test

- Record any adverse events
- Review medical history
- Perform complete physical examination
- Perform and record vital signs, body weight, pain level (0-10), and ECOG PS, as clinically indicated
- Collect blood and urine for clinical laboratory assessments, as clinically indicated

7.4.14 Survival Follow-up

- For subjects discontinuing the study treatment due to documented radiographic progression, obtain survival status via phone call or medical chart review, including information about subsequent anticancer therapies (including best response) every 3 months until death, loss of follow-up, or withdrawal of consent, whichever comes first.
- For subjects discontinuing the study treatment prior to documented radiographic progression, tumor assessments should continue on schedule approximately every 2 cycles or at the current scan interval at the time of treatment discontinuation until radiographic evidence of disease progression, the start of a subsequent anticancer therapy, or decision to no longer treat (eg, supportive care only), whichever is first. At that time, survival status (and subsequent anticancer therapy information, including best response, if appropriate) will be collected every 3 months until death, loss of follow-up, or withdrawal of consent, whichever comes first.

Table 38: Study Calendar for Midazolam Drug-Drug Interaction Sub-study

	**Screening**		**Cycle 1 28 days**				**Cycle 2 28 days**		**Cycle 3 28 days**	**Cycle 4 28 days (Response confirmation*)**	**Cycle 5 and Beyond**	**End of Treatment**	**Safety Follow-up**	**Long-Term Follow-up**
**Protocol Activity**	**Days -28 to -1**	**Day -2**	**Day 1**	**Day 8**	**Day 15**	**Day 22**	**Day 1**	**Day 15**	**Day 1**	**Day 1**	**Day 1**			
Visit Window[1]	NA			±1	±1	±1	±2	±2	±2	±2	±2	±7	+7	
Informed Consent[2]	X													
Tumor Molecular Alteration[3]	X													
Tumor Treatment History[4]	X													
Medical History	X		X	X	X	X	X	X	X	X	X	X	X	
Physical Examination	X	X	X	X	X	X	X	X	X	X	X	X	X	
ECOG Performance Status	X	X	X	X	X	X	X	X	X	X	X	X	X	
Height	X													
Weight	X	X	X	X	X	X	X	X	X	X	X	X	X	
Vital Signs[5]	X	X	X	X	X	X	X	X	X	X	X	X	X	
Administer midazolam		X				X								

Table 38: Study Calendar for Midazolam Drug-Drug Interaction Sub-study

	Screening		Cycle 1 28 days				Cycle 2 28 days		Cycle 3 28 days	Cycle 4 28 days (Response confirmation*)	Cycle 5 and Beyond	End of Treatment	Safety Follow-up	Long-Term Follow-up
**Protocol Activity**	**Days -28 to -1**	**Day -2**	**Day 1**	**Day 8**	**Day 15**	**Day 22**	**Day 1**	**Day 15**	**Day 1**	**Day 1**	**Day 1**			
Administer repotrectinib			X	X	X	X	X		X	X	X			
Dispense repotrectinib			X				X		X	X	X			
Repotrectinib Compliance				X	X	X	X	X	X	X	X	X		
Laboratory														
Complete Blood Count with Differential[6]	X		X	X	X	X	X	X	X	X	X	X	X	
Reticulocyte Count (% or absolute)	X		X				X		X	X	X	X	X	
Complete Metabolic Panel[7]	X		X	X	X		X	X	X	X	X	X	X	
Coagulation[8]	X		X	X	X	X	X		X	X	X	X	X	
Endocrine (for male subjects only)[9]	X		X				X		X	X	X	X	X	
Pregnancy Test (serum)[10]	X		X				X		X	X	X	X	X	

Table 38: Study Calendar for Midazolam Drug-Drug Interaction Sub-study

	**Screening**		**Cycle 1 28 days**				**Cycle 2 28 days**		**Cycle 3 28 days**	**Cycle 4 28 days (Response confirmation*)**	**Cycle 5 and Beyond**	**End of Treatment**	**Safety Follow-up**	**Long-Term Follow-up**
**Protocol Activity**	**Days -28 to -1**	**Day -2**	**Day 1**	**Day 8**	**Day 15**	**Day 22**	**Day 1**	**Day 15**	**Day 1**	**Day 1**	**Day 1**			
Urine Analysis[11]	X		X	X	X	X	X		X	X	X	X	X	
Blood Specimens for ccfDNA Enrichment[12]	X								X	X	X	X		
Cardiac Safety Monitoring														
Triplicate 12-lead ECG[13]	X		X		X	X	X		X	X		X		
Echocardiogram/MUGA for LVEF Assessment[14]	X								X	X	X	X		
Cardiac Troponin-I/T[15]	X													
Pharmacokinetics														
Plasma for Full repotrectinib PK[16]			X			X								
Plasma for full midazolam PK[17]		X				X								

Table 38: Study Calendar for Midazolam Drug-Drug Interaction Sub-study

	**Screening**		**Cycle 1 28 days**				**Cycle 2 28 days**		**Cycle 3 28 days**	**Cycle 4 28 days (Response confirmation*)**	**Cycle 5 and Beyond**	**End of Treatment**	**Safety Follow-up**	**Long-Term Follow-up**
**Protocol Activity**	**Days -28 to -1**	**Day -2**	**Day 1**	**Day 8**	**Day 15**	**Day 22**	**Day 1**	**Day 15**	**Day 1**	**Day 1**	**Day 1**			
Plasma for abbreviated repotrectinib PK[18]					X		X		X	X				
Tumor Assessment														
CT or MRI (chest/abdomen/pelvis)[19]	X								X	X	X	X		
MRI of Brain[20]	X								X	X	X	X		
Bone Scan[21]	X													
Other Clinical Assessments														
Adverse Events		X	X	X	X	X	X	X	X	X	X	X	X	
Concomitant Medications[22]	X		X	X	X	X	X	X	X	X	X	X	X	
Survival Follow-Up[23]														X

Abbreviations: AE, adverse event; BID, twice daily; ccfDNA, circulating cell-free deoxyribonucleic acid; CNS, central nervous system; CR, complete response; CRF, case report form; CSF, cerebrospinal fluid; CT, computerized tomography; CTCAE, Common Terminology Criteria for adverse event; ECG, electrocardiogram; ECHO, echocardiogram; ECOG, Eastern Cooperative Oncology Group; EOT, end of treatment; FFPE, formalin-fixed paraffin-embedded; HPF,

high power field; LVEF, left ventricular ejection fraction; MRI, magnetic resonance imaging; MUGA, multigated acquisition; QTc, QT interval corrected for heart rate; PK, pharmacokinetic; PR, partial response; TKI, tyrosine kinase inhibitor

[*]When PRs or CRs are observed, the responses will be confirmed ≥ 4 weeks later (ideally confirmation scan should be scheduled at end of the next cycle ie, 28 days) after the initial documentation of response by the Investigator.

[1] **Visit Window:** The 28-day screening period starts on the day informed consent is signed by the subject. The safety visit should be within 28 days from the last dose of repotrectinib.

[2] **Informed Consent**: Must be obtained before undergoing any protocol-specific procedures.

[3] **Tumor Molecular Alteration**: Confirmation of *ALK+, ROS1+*, or *NTRK+* rearrangement (submit molecular pathology report-source document) and submit archival tumor tissue (Section 5.1).

[4] **Tumor Treatment History**: Documentation of disease progression on prior TKI, duration of treatment with each prior TKI, prior chemotherapy regimens and duration of each chemotherapy regimens, prior immunotherapy regimens and duration of each immunotherapy regimens (if applicable), prior radiation to brain (if brain metastases present, and methods of radiation: whole brain radiation, stereotactic radiosurgery).

[5] **Vital Signs**: Body temperature, blood pressure, heart rate, respiratory rate, pain level (0-10).

[6] **Complete Blood Count with Differential**: White blood cell count, hemoglobin, platelet, absolute neutrophils, absolute lymphocytes, absolute monocytes, absolute eosinophils, absolute basophils, and absolute or % reticulocytes. On dosing days, assessments will be performed PRIOR to the dose of study medication being given.

[7] **Complete Chemistry Panel**: Sodium, potassium, chloride, bicarbonate or CO2, blood urea nitrogen, creatinine, magnesium, phosphorus, calcium, uric acid, total protein, albumin, glucose, lactate dehydrogenase, aspartate aminotransferase, alanine aminotransferase, total bilirubin, alkaline phosphatase. On dosing days, assessments will be performed PRIOR to the dose of study medication being given.

[8] **Coagulation**: Prothrombin time/international normalized ratio, partial thromboplastin time. On dosing days, assessments will be performed PRIOR to the dose of study medication being given.

[9] **Endocrine (for male subjects only)**: Total testosterone, sex hormone-binding globulin, follicle-stimulating hormone, luteinizing hormone, prolactin. These laboratories should be drawn between 8 am to 11 am in the morning.

[10] **Serum Pregnancy Test**: For female subjects of childbearing potential, a serum pregnancy test, with sensitivity of at least 25 mIU/mL, will be performed on 2 occasions prior to starting study therapy: once at the start of screening and once at the baseline visit, immediately before study drug administration. Only subjects with negative serum pregnancy tests are allowed to enroll. Serum pregnancy test will also be performed at the beginning of each cycle of visit.

[11] **Urine Analysis**: Urinalysis includes the analysis of protein, glucose, ketones, blood, and specific gravity. A microscopic (white blood cells/high power field [HPF], red blood cells/HPF, and any additional findings) exam need only be performed if the urinalysis result is abnormal. More frequent and complete examinations may be performed at the Investigator's discretion if medically indicated; results should be recorded on unscheduled visit CRFs.

[12] **Blood Specimens for ccfDNA Enrichment (20 mL blood specimen)**: Blood will be collected in two 10 ml Streck Cell-Free DNA Blood Collection Tubes prior to the first dose of repotrectinib, at the Cycle 3 Day 1 visit, and either at the time of tumor progression or EOT (whichever is earlier) for exploratory ccfDNA molecular profiling analyses. Details for handling of these specimens including processing, storage, and shipment will be provided in the Study Manual. For Cycle 5 and beyond, collect a blood sample (ccfDNA) if patient is confirmed to have progressed.

[13] **Triplicate 12-lead ECGs**: At each time point, 3 consecutive 12-lead ECGs will be performed approximately 2 minutes apart to determine mean QTc interval. Triplicate 12-lead ECGs will all be time matched with PK with the exception of during the screening period and at the EOT. Triplicate ECGs will be collected as follows:

a) At Screening.
b) Cycle 1 Day 1, at pre-dose (0 hour), at 1, 2, 4, 6, 8, and 24 hours post-dose.
c) Cycle 1 Day 15, at pre-dose (0 hour).
d) Cycle 1 Day 22 after single-dose administration at pre-dose (0 hour), at 1, 2, 4, 6, 8, and 24 hours post-dose.
e) Cycles 2-4, Day 1 at pre-dose (0 hour) and 4 hours post-dose.

In addition to these time points, ECGs should be repeated as clinically indicated. Additional ECG time points may be included based on the emerging data. Interpretation of the tracing will be made by a central ECG laboratory. Each ECG tracing should be labeled with the study number, subject initials, subject number, date, and kept in the source documents at the study site. Only clinically significant abnormalities will be recorded in the AE CRF page. Clinically significant abnormalities at screening/baseline should be recorded on the relevant medical history/current medical conditions CRF page. Clinically significant findings must be discussed with the Sponsor Medical Monitor prior to enrolling the subject in the study.

[14] **Echocardiography or MUGA for LVEF Assessments**: Echocardiography to be performed at screening, and after every 3 cycles of treatment (± 7 days) (Cycles 4, 7, 10, 13 and so on), and at the EOT visit (± 7 days). **Cardiac Troponin-I or Troponin T**: Cardiac troponin-I or troponin T will be done at baseline then repeated and assessed whenever the ECG morphology demonstrates possible myocardial ischemia or infarction. If troponin CTCAE grade ≥ 3 is seen, this parameter must be repeated twice a week until resolution to CTCAE grade ≤ 1.

[15] **Cardiac Troponin-I or Troponin T:** Cardiac troponin-I or troponin T will be done at baseline then repeated and assessed whenever the ECG morphology demonstrates possible myocardial ischemia or infarction. If troponin CTCAE grade ≥ 3 is seen, this parameter must be repeated twice a week until resolution to CTCAE grade ≤ 1.

[16] **Plasma for Full repotrectinib PK**: Timing for full PK: Cycle 1 Day 1 PK pre-dose (time 0), 1, 2, 4, 6, 8, and 24-hours post-dose. Cycle 1 Day 22 pre-dose (time 0), 1, 2, 4, 6, 8 and 24 hours post-dose. For BID dose, 24-hour post-dose is relative to the first dose of the day.

[17] **Plasma for Full midazolam PK**: Timing for full PK: Cycle 1 Day-2 PK pre-dose (time 0), 0.5, 1, 2, 4, 6, 8, and 24 hours post-dose. Cycle 1 Day 22 pre-dose (time 0), 0.5, 1, 2, 4, 6, 8, and 24 hours post-dose. For BID dose schedule, 24-hour post-dose is relative- to the first dose of the day.

[18] **Plasma for Abbreviated repotrectinib PK**: Timing for abbreviated PK: at pre-dose (0 hour) and 4 hours post-dose on Cycles 2-4 Day 1, and at pre-dose (0 hour) on Cycle 1 Day 15.

[19] **CT or MRI Chest/Abdomen/Pelvis Tumor Assessment:** Tumor assessments will include all known or suspected disease sites. For all tumor assessments, the method of assessment that was used at baseline should be the same method used throughout the study. All screening scans must be submitted for central review to confirm measurable disease before the subject is enrolled to the study. For subjects who are without documented disease progression, CT or MRI scans to be done at every 2 cycles (7 weeks after starting treatment for the first on treatment assessment and then every 2 cycles up to Cycle 18 and then every 3 cycles up to Cycle 36 and then every 4 cycles thereafter until documented progression of disease). Subject responses will be confirmed ≥ 4 weeks later (ideally confirmation scan should be scheduled at end of the next cycle ie, 28 days) after the initial documentation of response by the Investigator. For subjects who have documented disease progression but are continuing repotrectinib post-RECIST progression, CT or MRI scans are to be done according to local institutional standard of care. For subjects discontinuing the study treatment prior to documented radiographic progression, tumor assessments should continue on schedule approximately every 2 cycles or at the current scan interval at the time of treatment discontinuation until radiographic evidence of disease progression, the start

of a subsequent anticancer therapy, withdrawal of consent, or decision to no longer treat (eg, supportive care only), whichever is first. The end of treatment scan is optional ONLY if performed within the past 4 weeks. Every effort should be made to maintain the assessment scheduling relative to Cycle 1 Day 1 especially if there are dosing cycle interruptions due to toxicities. For all subjects, copies of radiologic images must be available for independent central radiology review as determined by the Sponsor Medical Monitor.

[20] **MRI of Brain:** Gadolinium contrast-enhanced MRI must be used for assessment of CNS lesions at baseline with contingent slices of 1 mm for lesions 5 to 10 mm in size, 5 mm for lesions greater than 10 mm. For the Phase 1b food effect portion of the study, only subjects with documented baseline CNS metastases will be assessed by MRI of the brain at every 2 cycles (after 7 weeks for the first assessment and then every 2 cycles up to Cycle 18 and then every 3 cycles up to Cycle 36 and then every 4 cycles thereafter until documented progression of disease). Subject responses will be confirmed ≥ 4 weeks later after (ideally confirmation scan should be scheduled at end of the next cycle ie, 28 days) the initial documentation of response by the Investigator. For subjects who have documented disease progression but are still continuing repotrectinib post-RECIST progression, MRI scans of the brain are to be done according to local institutional standard of care. Subject responses will be confirmed ≥ 4 weeks later (ideally confirmation scan should be scheduled at end of the next cycle ie, 28 days) after the initial documentation of response by the Investigator. For subjects discontinuing the study treatment prior to documented radiographic progression, tumor assessments should continue on schedule approximately every 2 cycles or at the current scan interval at the time of treatment discontinuation until radiographic evidence of disease progression, the start of a subsequent anticancer therapy, withdrawal of consent, or decision to no longer treat (eg, supportive care only), whichever is first. The end of treatment scan is optional ONLY if performed within the past 4 weeks.

[21] **Bone Scans**: Bone scans (or bone MRI if preferred by Investigator) will be performed at baseline if bone metastases are suspected. These scans are only repeated for subjects having a baseline scan, to confirm the absence of bone metastases in case of a Complete Response (CR). In addition, bone scans can be performed if clinically indicated per Investigator's discretion. Subject responses will be confirmed ≥ 4 weeks later (ideally confirmation scan should be scheduled at end of the next cycle ie 28 days) after the initial documentation of response by the Investigator.

[22] **Concomitant Medications and Non-Drug Supportive Interventions**: All concomitant medications and non-drug supportive interventions should be recorded in the CRF.

[23] **Survival Follow-Up**: For subjects discontinuing the study treatment due to documented radiographic progression, obtain survival status via phone call or medical chart review, including information about subsequent anticancer therapies (including best response) every 3 months until death, loss of follow-up, or withdrawal of consent, whichever comes first.

APPENDIX 8 COUNTRY SPECIFIC REQUIRMENTS / DIFFERENCES

The countries listed below have the following country-specific changes to the text provided above. These changes apply to the listed country only, and to none of the other countries listed below (e.g., the additional weight criteria for inclusion for Canada applies to subjects in Canada only, and not to subjects in Germany).

Country / Location Requirement	Section Number	Country-specific Language or Differences
Canada	Protocol Synopsis and Section 5.2, Inclusion Criteria #4 for Phase 2	Additional weight criteria for inclusion:<br>4. Age ≥12 and >50kg (or as required by local regulations).
France	Protocol Synopsis and Section 5.2, Inclusion Criteria #7 for Phase 2, EXP-5<br><br>Section 3.5.2, EXP-5	Prior lines of chemotherapy or immunotherapy are allowed. Disease progression on prior systemic therapy is required unless no appropriate therapeutic alternative exists.
France	Protocol Synopsis and Section 5.2, Inclusion Criteria #8 for Phase 2, EXP-5	Required wash-out time that is related to prior therapies before starting repotrectinib treatment:<br>• If the immediate prior treatment was a ROS1 or TRK TKI: Approximately 5 half-lives must have elapsed since completion of treatment with the last TKI for subjects enrolling into the pretreated expansion cohorts (EXP-2, -3, -4, and -6). All side effects from prior treatments with a ROS1 or TRK TKI must have resolved to grade ≤ 1 prior to starting treatment with repotrectinib.<br>• Approximately 5 half-lives must have elapsed after discontinuation of prior systemic chemotherapy (or at least 42 days for prior nitrosoureas and mitomycin C) and all side effects from prior treatments must have resolved to grade ≤1 with the exception of alopecia.<br>• Approximately 5 half-lives must have elapsed after discontinuation of prior immunotherapy and all immune-related side effects from prior immunotherapy must have resolved to grade ≤1.

Country / Location Requirement	Section Number	Country-specific Language or Differences
France	Protocol Synopsis and Section 5.2 (Laboratory Table), Inclusion Criteria #10 for Phase 2	Baseline laboratory values fulfilling the following requirements:<br>In addition to calcium, magnesium and potassium, subject's phosphate level should also be within normal of CTCAE grade ≤1 with or without supplementation
France	Appendix 1	Additional contraception related guidance:<br>Before starting treatment, subjects may wish to consider cryopreservation of semen or harvested eggs.
France	Section 6.2.4	Additional Considerations for Dose Modification in Phase 2:<br><br>Dyspnoea, Hypoxia, Pleural Effusion<br>Subjects who present with worsening of respiratory symptoms such as dyspnoea, cough, pyrexia, hypoxia, and/or pleural effusion should be promptly investigated for interstitial lung disease (ILD)/pneumonitis or other causes such as infections, pulmonary embolism or complications associated with disease progression. Immediately withhold repotrectinib in subjects diagnosed with ILD/pneumonitis and permanently discontinue if no other causes of ILD/pneumonitis have been identified. For all other causes, treat per institutional standard of care and follow the dose management guidance for Non-hematologic Adverse Events including ILD/pneumonitis. Please refer to Table 14 Dose Modification Guidance for Adverse Events.<br><br>Hypophosphataemia<br>Serum calcium and phosphate concentrations are monitored during therapy. Treatment of the underlying disorder and oral phosphate replacement are usually adequate in asymptomatic subjects. Consider phosphate replacement therapy per institutional standard of care. For dose management for grade 3

Country / Location Requirement	Section Number	Country-specific Language or Differences
		phosphate withhold repotrectinib until toxicity is ≤grade 1 or returns to baseline following supplementation then reduce by 1 dose level to resume treatment. Refer to Table 14 for Dose Modifications for Adverse Events (Non-Hematologic Adverse Events).<br><br>Stroke, Cerebrovascular Accident, Hemiparesis<br>During the study subjects will be routinely monitored through frequent physical exams and serial blood collection for hematology and chemistry. Subjects with risk factors for a stroke or a cerebrovascular accident, such as smoking, hypertension, dyslipidemia, diabetes should be closely monitored and guided to modify risk factors through lifestyle changes to help delay or prevent subsequent strokes. Stroke prevention strategies based on the subject's risk factors may include procedures (eg, carotid endarterectomy, stent placement), antiplatelet therapy, and anticoagulation. If a stroke is suspected, immediate neuroimaging is required to differentiate hemorrhagic from ischemic stroke and to detect signs of increased intracranial pressure. Specific acute treatments vary by type of stroke and should follow institutional standard of care. Dose management for repotrectinib for stroke or cerebrovascular events should follow guidance provided in Table 14 Dose Modifications for Adverse Events (Non- Hematologic Adverse Events).
Germany	Protocol Synopsis and Section 5.2, Inclusion Criteria # 3, 4, and 5	Eastern Cooperative Oncology Group (ECOG) PS 0–1. Subjects who are unable to walk because of paralysis or tumor pain, but who are in a wheelchair, will be considered ambulatory for the purpose of assessing the performance score.<br>Age ≥18 (or as required by local regulations).<br>Willing and able to provide written institutional review board (IRB)/institutional ethics committee-approved Informed Consent.

Country / Location Requirement	Section Number	Country-specific Language or Differences
Germany	Protocol Synopsis, section 8.1, 8.2, 8.3, 8.4, Table 23-26	Safety follow-up visit:<br>Safety follow-up visit will be conducted within 30 days of last dose of repotrectinib.
Germany	Sections 7.1.7, 8.4.1 to 8.4.15	Study procedures:<br>As only subjects age ≥18 is allowed in Germany, wherever applicable, study procedures related to Lansky and Karnofsky performance score and PK sample collection in adolescents is NOT applicable.
Germany	Synopsis - Exclusion Criteria for Phase 1 and Phase 2 and Section 5.3	Additional exclusion criteria:<br>Subjects with R0-resectable cancer (i.e., no identifiable tumor remaining, negative surgical margins after surgery)
Germany	Section 9.2.1	AE reporting period:<br>After initiation of study drug, all AEs, regardless of relationship to study drug, will be reported until at least 30 days after the last dose of study treatment.
Germany	Section 9.2.1, 9.3.1, and 9.5	SAE reporting period:<br>After initiation of study drug, SAEs will be reported until end of the study, regardless of the relationship to the study drug.
Germany	Section 14.6	Additional Clarification for Monitoring:<br>In the event that on-site Source Data Verification (SDV) is impossible due to exceptional circumstances, Premier Research may perform remote SDV activities under the following guidelines:<br>CRAs and key site staff members will be trained on the Remote SDV Plan in advance of any activities being conducted.<br>Remote SDV and other monitoring activities will be customized based on site resources and capabilities. Premier Clinical Research Associates (CRAs) must review the subject informed consent form (ICF) prior to performing any Remote SDV activities. While viewing any source documentation remotely,

Country / Location Requirement	Section Number	Country-specific Language or Differences
		CRA should ensure physical controls (i.e., not using computer in public location, high traffic areas, etc.) are in place to prevent inadvertent sharing of confidential data. All CRAs will be required to document activities according to the approved Clinical Monitoring Plan (CMP).<br>The methods for remote SDV are listed below in order of project preference, based on site ability to implement:<br>• Remote SDV – via Electronic Medical Records (EMR) access<br>• Remote SDV – via outside party managed External Access System (EAS) – managed by the site<br>• Remote SDV – via Premier Managed eTMF/External Access System (EAS) – BOX.com™<br>• Remote SDV – via outside party managed External Access System (EAS) – managed by the client or third-party vendor<br>• Remote SDV – via email/fax<br>• Remote SDV – via video conference.
Germany	Appendix 1	The sole use of barrier methods (also as a double barrier method) is not considered highly effective contraceptive method and should not be used by WOCBP. If WOCBP chooses to use hormonal contraceptives (including oral hormonal contraceptives), they then must use either another form of non-hormonal highly effective contraception or a reliable barrier method.

APPENDIX 9 SUMMARY OF CHANGES

OVERALL RATIONALE FOR PROTOCOL AMENDMENT 15:

Summary of Changes for Version 15.0 to Version 16.0. This Summary of Changes details the substantive changes to protocol TPX-0005-01.

The protocol is being modified to include conducting baseline and post - baseline ophthalmologic assessments to evaluate risk factors, manifestations, and outcomes associated with potential ocular toxicity with repotrectinib per health authority request.

Minor changes for editorial clarifications, and for administrative corrections of typographic errors, reformatting, abbreviations or section/table re-numbering are not included in this summary.

SUMMARY OF CHANGES FOR PROTOCOL AMENDMENT 15		
**Section Number & Title**	**Description of Change**	**Brief Rationale**
Section 5.2: Inclusion Criteria for Phase 2	Modified the text for platelets, hemoglobin, and creatinine clearance requirements.	For consistency with text in the synopsis, and to specify requirements.
Section 7.1.12: Ophthalmologic Examination<br>Section 13.4.8: Ophthalmologic Assessment	Added subsections.	To incorporate Health Authority request to include ophthalmologic examination.
Section 8.4.3: Cycle 1 Day 1 (Phase 2 Study)<br>Section 8.4.9: Cycle 3 Day 1 (Phase 2 Study)<br>Section 8.4.12: End of Treatment (Phase 2 Study)	Added ophthalmologic exam.	To incorporate the addition of ophthalmologic exam.

SUMMARY OF CHANGES FOR PROTOCOL AMENDMENT 15		
**Section Number & Title**	**Description of Change**	**Brief Rationale**
(within 7 days post last dose of repotrectinib and after decision to end treatment) Table 26: Study Calendar for Phase 2 Study of TPX-0005-01		
Table 26: Study Calendar for Phase 2 Study of TPX-0005-01	Removed pharmacodynamic (PD) assessment, and its footnote.	PD biopsy is not collected during C1D8 (Cycle 1, Day8) and C1D15 (Cycle 1, Day 15).
All	Minor formatting and typographical corrections.	Minor, therefore have not been summarized.

INTEGRATED STATISTICAL ANALYSIS PLAN – NTRK

Analysis of the Anti-Tumor Activity of Repotrectinib in Patients with *NTRK*-positive Advanced or Metastatic Solid Tumors and Safety of Repotrectinib in Patients with Advanced or Metastatic NSCLC or Other Solid Tumors

Protocols Included:	TPX-0005-01 Phase 1 (TRIDENT-1)<br>TPX-0005-01 Phase 2 (TRIDENT-1)<br>TPX-0005-07 (CARE)
SAP Version:	1.0 for Subjects with Solid Tumors that are NTRK+
Date:	24 February 2023
Compound:	TPX-0005
Compound Name:	Repotrectinib
United States (US) Investigational New Drug (IND) Number:	130465
European Clinical Trial Database (EudraCT) Number:	2016-003616-13
Turning Point Therapeutics (a wholly owned subsidiary of Bristol Myers Squibb Company):	Turning Point Therapeutics, Inc.<br>10628 Science Center Drive, Suite 200<br>San Diego, CA 92121 USA

Approved v1.0 930213273 1.0

TURNING POINT THERAPEUTICS APPROVAL

Integrated SAP Approval:	Repotrectinib (TPX-0005)<br>Integrated Statistical Analysis Plan (SAP) for efficacy and safety for NTRK+ Subjects
Version (Date):	1.0 (24 February 2023)
SAP Title:	Analysis of the Anti-Tumor Activity of Repotrectinib in Patients with NTRK-positive Advanced or Metastatic Solid Tumors and Safety of Repotrectinib in Patients with Advanced or Metastatic NSCLC or Other Solid Tumors

[redacted]

Date: 24-Feb-2023 | 11:22 EST

[redacted]

Pharmapace, Inc.

[redacted]

Date: 24-Feb-2023 | 15:46 EST

[redacted]

Premier Research

[redacted]

Date: 24-Feb-2023 | 11:34 EST

[redacted]

Premier Research

[redacted]

Date: 24-Feb-2023 | 08:19 PST

[redacted]

Turning Point Therapeutics

Approved v2.0 930213273 1.0

TABLE OF CONTENTS

TURNING POINT THERAPEUTICS APPROVAL 2
TABLE OF CONTENTS 3
LIST OF TABLES 5
LIST OF FIGURES 6
LIST OF ABBREVIATIONS AND DEFINITION OF TERMS 7
1. INTRODUCTION 9
2. OBJECTIVES AND ENDPOINTS 10
2.1. Efficacy Endpoints 10
2.2. Safety Endpoints 10
3. STUDY OVERVIEW 11
3.1. *ROS1 or NTRK* Expansion Cohorts 14
3.1.1. *ROS1*+ NSCLC Expansion Cohorts 14
3.1.2. *NTRK*+ Advanced Solid Malignancy Expansion Cohorts 14
4. ANALYSIS SETS 17
4.1. Efficacy Analysis Set 18
4.2. Safety Analysis Set 19
5. STATISTICAL ANALYSIS METHODS AND CONSIDERATIONS 20
5.1. Sample Size Determinations 20
5.1.1. EXP-5 TRK TKI-Naïve NTRK+ Solid Tumors 20
5.1.2. *NTRK*+ TKI-pretreated Solid Tumors 20
5.1.2.1. EXP-6 TRK TKI-Pretreated NTRK+ Advanced Solid Tumors 20
5.2. General Considerations 21
5.3. Disposition of Subjects 22
5.4. Protocol Violations and Deviations 24
5.5. Demographics and Baseline Characteristics 24
5.6. Efficacy Analysis 26
5.6.1. Analysis of Primary Efficacy Endpoints 26
5.6.2. Analysis of Secondary Endpoints 27
5.6.2.1. Duration of Response 27
5.6.2.2. Time to Response 28
5.6.2.3. Clinical Benefit Rate 28

Approved v2.0 930213273 1.0

5.6.2.4. Progression-Free Survival 28
5.6.2.5. Overall Survival 29
5.6.2.6. Intracranial Objective Response Rate 30
5.6.3. Subgroup Analysis 31
5.6.4. Exploratory Analysis 32
5.6.4.1 Subsequent Anti-Cancer Therapies 32
5.6.4.2 Analysis of Tumor Location and Progression 32
5.7 Safety Analyses 33
5.7.1 Extent of Exposure 33
5.7.2 Adverse Events 33
5.7.3 Clinical Laboratory Evaluations 36
5.7.4 Drug-induced Liver Injury 37
5.7.5 Performance Status and Vital Signs 38
5.7.6 Electrocardiograms 38
5.7.7 Left Ventricular Ejection Fraction 39
5.7.8 Death 39
5.7.9 Patient-Reported Outcomes (PRO) 39
6 ANALYSIS METHODS AND CONSIDERATIONS FOR CARE (TPX-0005-07) 40
6.1 Introduction 40
6.2 Analysis Population Sets 40
6.2.1 Full Analysis Set 40
6.2.2 NTRK Efficacy Evaluable Analysis Set 40
6.2.3 PK Analysis Sets 40
6.3 Disposition, Demographic and Baseline and Disease Characteristics 41
6.4 Efficacy Analysis 41
6.4.1 Analysis of Efficacy Endpoints 41
6.5 Safety Analysis 43
6.5.1 Extent of Exposure 43
6.5.2 Adverse Events 43
6.5.3 Clinical Laboratory Evaluations 44
6.5.4 Drug-induced Liver Injury 45
6.5.5 Karnofsky/Lansky Performance Status 45

Approved v1.0 930213273 1.0

6.5.6 Vital Signs 46
6.5.7 Prior and Concomitant Medications 47
6.5.8 Electrocardiograms (ECG) 47
6.5.9 Left Ventricular Ejection Fraction (LVEF) 47
6.6 Pharmacokinetic Analysis 48
6.6.1 Pharmacokinetic (PK) Summaries 48
7 REFERENCES 50

LIST OF TABLES

Table 1: Repotrectinib Studies in Cancer Subjects and Data for NDA Submission 12
Table 2: Estimated ORRs and 95% Confidence Intervals Using Sample Size N = 55 for EXP-5 20
Table 3: Estimated ORRs and 95% Confidence Intervals using Sample Size N = 40 for EXP-6 21
Table 4: Reason for Treatment and Study Discontinuation 23
Table 5: Outcome and Date Values for DOR Derivations 27
Table 6: Outcome and Date Values for PFS Derivations 29
Table 7: Outcome and Date Values for OS Derivations 30
Table 8: AE of Special Interest Derivations 36
Table 9: Vital Signs of Potential Clinical Importance 38
Table 10: Karnofsky and Lansky Performance Status Scale 46
Table 11: Vital Signs of Potential Clinical Importance 46

Approved v1.0 930213273 1.0

LIST OF FIGURES

Figure 1: Schema of TPX-0005-01 Phase 1 and Phase 2 Portions by Expansion Cohort 16
Figure 2: Integrated Safety and Efficacy Analysis Sets 17

Approved v1.0 930213273 1.0

LIST OF ABBREVIATIONS AND DEFINITION OF TERMS

The following abbreviations and specialist terms are used in this study document.

Abbreviation	Definition
AE	adverse events
AESI	adverse events of special interest
ALK	anaplastic lymphoma kinase
BICR	Blinded Independent Central Review
BID	twice daily
BOR	best overall response
CBR	clinical benefit rate
CI	confidence interval
CNS	central nervous system
CNS-PFS	central nervous system progression-free survival
cORR	confirmed objective response rate
CR	complete response
CRF	case report form
CSR	clinical study report
CTCAE	Common Terminology Criteria for Adverse Events
DDI	drug-drug interaction
DOR	duration of response
ECG	electrocardiogram
ECOG	Eastern Cooperative Oncology Group
EXP	expansion
FDA	Food and Drug Administration
FISH	fluorescence *in situ* hybridization
IC-ORR	intracranial objective response rate
LVEF	left ventricular ejection fraction
MedDRA	Medical Dictionary for Regulatory Affairs
MUGA	multigated acquisition
NCA	noncompartmental analysis
NDA	New Drug Application
NGS	next-generation sequencing
NSCLC	non-small cell lung cancer

Approved v2.0 930213273 1.0

Abbreviation	Definition
NTRK	neurotrophic receptor kinase
NTRK-1	neurotrophic receptor kinase 1
NTRK-2	neurotrophic receptor kinase 2
NTRK-3	neurotrophic receptor kinase 3
ORR	objective response rate
PD	progressive disease
PFS	progression-free survival
PK	pharmacokinetic
PR	partial response
PT	preferred term
QD	once daily
qPCR	quantitative polymerase chain reaction
QT	ECG interval measured from the onset of the QRS complex to the end of the T wave
QTc	QT interval corrected for heart rate
QTcB	QT interval corrected for heart rate using Bazett's formula
QTcF	QT interval corrected for heart rate using Fridericia's formula
RECIST	Response Evaluation Criteria in Solid Tumors
ROS1	receptor tyrosine kinase encoded by the *ROS1* gene
RP2D	recommended Phase 2 dose
SAE	serious adverse event
SAP	statistical analysis plan
SD	stable disease
SOC	system organ class
TEAE	treatment-emergent adverse event
TKI	tyrosine kinase inhibitor
TRK	tropomyosin receptor kinase
TTR	time to response

Approved v1.0 930213273 1.0

1. INTRODUCTION

This integrated statistical analysis plan (SAP) describes the objectives, endpoints, and detailed statistical analysis methods proposed for the analysis of efficacy and safety data integrated across the Phase 1 and Phase 2 portions of the TPX-0005-01 **(**TRIDENT-1) study. This integrated SAP serves as the primary source of analyses for a new drug application (NDA) submission to support approval for the treatment of adult subjects with advanced or metastatic solid tumors whose tumors are *NTRK+*. Individual SAPs are available for clinical study report (CSR) to support the protocol objectives of the Phase 1 and Phase 2 portions of TPX-0005-01, respectively, and are separate from this document. Additionally, another integrated SAP focused on ROS1+ NSCLC is available.

For this integrated analysis plan, subject data will be pooled from the Phase 1 and Phase 2 portions of TRIDENT-1 study. The integrated analysis will be the focus of this SAP in Section 3 through Section 5.

> **TPX-0005-01 (TRIDENT-1)** – A Phase 1/2, Open-Label, Multi-Center, First-in-Human Study of the Safety, Tolerability, Pharmacokinetics, and Anti-Tumor Activity of TPX-0005 in Patients with Advanced Solid Tumors Harboring *ALK*, *ROS1*, or *NTRK1-3* Rearrangements

Data from pediatric subjects will not be integrated with the data from TRIDENT-1, but will be included as supportive for safety and efficacy objectives from the CARE study. The separate analysis of the CARE data will be described in Section 6 .

> **TPX-0005-07 (CARE)** – A Phase 1/2, Open-label, Safety, Tolerability, Pharmacokinetics, and Anti-Tumor Activity of Repotrectinib in Pediatric and Young Adult Subjects with Advanced or Metastatic Malignancies Harboring *ALK*, *ROS1*, or *NTRK1-3* Alterations

Any major changes to this integrated SAP will be updated, as needed, through an amendment to this document.

Approved v2.0 930213273 1.0

2. OBJECTIVES AND ENDPOINTS

The objective of this integrated SAP is to provide the statistical analysis details to support the efficacy and safety claim for repotrectinib for the treatment of adult subjects with advanced or metastatic solid tumors whose tumors are *NTRK+*. The efficacy and safety endpoints for this integrated SAP are summarized below.

2.1. Efficacy Endpoints

In *NTRK+* subjects with advanced or metastatic solid tumors:

- Confirmed Objective Response Rate (cORR) as assessed by Blinded Independent Central Review (BICR).
- Duration of response (DOR), time to response (TTR), and clinical benefit rate (CBR) as assessed by BICR.
- Progression-free survival (PFS) as assessed by BICR.
- Intracranial objective response rate (IC-ORR) and Central Nervous System PFS (CNS-PFS) by BICR in subjects presenting with measurable brain metastases at baseline.
- cORR, DOR, TTR, CBR, and PFS as assessed by Investigator
- Overall Survival (OS)

2.2. Safety Endpoints

All safety parameters, including adverse events, clinical lab parameters, electrocardiogram results, performance status, and vital signs.

Approved v2.0 930213273 1.0

3. STUDY OVERVIEW

Repotrectinib is currently being evaluated as monotherapy in two ongoing Phase 1/2 studies in cancer subjects, TPX-0005-01 (TRIDENT-1) and TPX-0005-07.

The first-in-human Phase 1/2 clinical study (TRIDENT-1) is enrolling subjects with advanced solid tumors harboring *ROS1, NTRK1-3,* or *ALK* rearrangements to evaluate the safety, tolerability, pharmacokinetics (PK), and anti-tumor effect of repotrectinib. The Phase 1 portion of the study included a dose escalation evaluation (40 mg once daily [QD] to 200 mg twice daily [BID]) and a food-effect sub-study. Additionally, the protocol included a midazolam drug-drug interaction (DDI) sub-study at recommended Phase 2 dose (RP2D). The Phase 2 portion of the study includes treatment of subjects at the RP2D into 6 expansion cohorts based on different molecular characteristics. Enrollment has been completed in the dose escalation portion, food-effect portion, and the midazolam DDI sub-study of Phase 1. The Phase 2 portion of the study continues to enroll and treat subjects into the NTRK cohorts only. Screening for enrollment into the ROS1 cohorts was closed on 31 December 2022.

The second ongoing clinical trial of repotrectinib in monotherapy is a Phase 1/2 study in pediatric subjects and young adults (CARE) to evaluate the safety, tolerability, PK, and anti-tumor effects in subjects with advanced or metastatic malignancies harboring *ALK*, *ROS1*, or *NTRK1-3* alterations. NTRK1-3 alterations span a variety of tumor types and represent potentially targetable oncogenic drivers in pediatric patients. Currently there are no approved TKIs for pediatric patients with advanced or metastatic malignancies harboring NTRK1-3 fusions who have been pretreated with a TRK TKI. The unique polypharmacology profile of repotrectinib allows for not only inhibition of mutated TRKs, ALK, and ROS1 but also for combatting multiple resistance mechanisms simultaneously, making repotrectinib an attractive new treatment for patients with NTRK-driven malignancies. The number of pediatric subjects enrolled is expected to be small at the time of data cutoff for NDA, therefore data from the pediatric study (CARE (TPX-0005-07)) will not be pooled for this integrated summary of efficacy and safety. Instead, independent analysis will be presented for TPX-0005-07 to support the NDA submission in the pediatric population. This separate analysis of the CARE data is described in this document in Section 6.

Table 1 presents a summary of repotrectinib studies in adult cancer subjects and the available data for the NDA submission.

Approved v2.0 930213273 1.0

Table 1: Repotrectinib Studies in Cancer Subjects and Data for NDA Submission

Study	Phase/Indication	Study Design	# of Subjects Treated[a]	Datasets	Report
TRIDENT-1 Phase 1 and midazolam DDI substudy	Phase 1; adults with advanced solid tumors	Open-label, dose escalation (3+3), PK, efficacy, and safety study	103	CDISC (SDTM, ADaM)	CSR
TRIDENT-1 Phase 2	Phase 2; adults and youth with advanced solid tumors	Open-label, multi-cohort, global registrational, efficacy and safety study	416	CDISC (SDTM, ADaM)	CSR
CARE	Phase 1/2; pediatric subjects and young adults up to age 25 with solid tumors	Open-label, dose-escalation with dose expansion Phase 2 cohort, efficacy and safety study	25	CDISC (SDTM, ADaM)	Ongoing – CSR not yet available
ISS and ISE	Phase 1 and Phase 2 of TRIDENT-1	Integrated summary of safety and efficacy	519 for ISS<br>88 for ISE	CDISC (SDTM, ADaM)	Report

[a] Number of subjects treated is based on a data cut-off date of 19 December 2022.

The Phase 1 and Phase 2 portions of TPX-0005-01 (TRIDENT-1) study will be included as part of this integrated SAP. Due to each phase of this protocol having different objectives and entry criteria, the study designs are described separately.

The integrated efficacy analysis will focus on the *NTRK*+ solid tumor cohorts, while the integrated safety analysis will focus the overall safety population regardless of genetic rearrangement or tumor type.

TPX-0005-01 (TRIDENT-1) Phase 1 enrolled subjects whose tumors harbored *ROS1*, *NTRK1-3*, and *ALK* genetic alterations to explore finding the maximum tolerated dose and recommended Phase 2 dose (RP2D) of repotrectinib using a 3+3 design. In the Phase 1 portion, the initial RP2D cohort expansion subjects were dosed with repotrectinib at 160 mg QD for 7 days followed by the option to increase dose to 160 mg BID, if tolerated. The final RP2D used for the Phase 2 portion of the study was determined to be 160 mg QD for 14 days followed by the option to increase dose to 160 mg BID, if tolerated. Subjects entering the Phase 1 portion of the study were not required to have baseline measurable disease by BICR, specific tumor types, or to have been treated with a specific number of lines of anti-cancer therapy. There was no limit to the number of prior TKI, chemotherapy, or immunotherapy regimens. Additionally, with regard to genetic rearrangements, subjects were entered based on local test results and no central laboratory confirmation was required. To harmonize with the inclusion procedures in Phase 2,

Approved v2.0 930213273 1.0

subjects who entered Phase 1 based on a local test with *ROS1* or *NTRK* generic rearrangement by fluorescence *in situ* hybridization (FISH) will be required to have their genetic rearrangement retrospectively confirmed by central laboratory (Almac). Subjects who entered based on a local test by next generation sequencing (NGS) or quantitative polymerase chain reaction (qPCR) for *ROS1* or *NTRK* will not be required to have their rearrangement confirmed retrospectively. *ALK* gene mutation identified by local testing will not be retested by central laboratory. The Phase 1 portion of the protocol also includes a midazolam DDI sub-study. The subjects treated in this sub-study will be included in the integrated safety analysis but will not be included in the integrated efficacy analysis set. Enrollment into the Phase 1 portion of the study, including the midazolam DDI sub-study has been completed.

TPX-0005-01 (TRIDENT-1) Phase 2 evaluates the anti-cancer activity of single-agent repotrectinib at the RP2D in multiple cohorts of subjects with *ROS1+* and *NTRK1-3* advanced solid malignancies including 2 *NTRK+* cohorts specific for solid tumors. Subjects are accrued into 6 different expansion cohorts according to tumor of origin, prior anti-cancer treatment, and *ROS1*, or *NTRK* genetic rearrangement (see Figure 1 and Section 3.1). Subjects will be enrolled into each cohort based on results of genetic rearrangement status of *ROS1*, and *NTRK1-3* as determined by local or central results. Subject tested with NGS or qPCR test locally were enrolled without confirmation by central diagnostic testing. However, subjects tested with FISH locally were required to have prospective confirmation of a *ROS1+* or *NTRK+* gene fusion by central laboratory prior to enrollment. These subjects will be included as part of the integrated safety and efficacy analysis.

Subjects will continue to be enrolled and treated into the Phase 2 portion of the protocol for the NTRK cohorts (EXP-5 and EXP-6) until each expansion cohort is complete as specified in the study protocol. Screening for enrollment into the ROS1 cohorts was closed on 31 December 2022. (Figure 1).

The CARE study is a Phase 1/2, open-label, single-arm, multicenter, multicohort study to evaluate the safety, tolerability, PK, and efficacy of the ALK/ receptor tyrosine kinase encoded by the *ROS1* gene (ROS1)/neurotrophin receptor kinase /(NTRK) inhibitor, repotrectinib, in pediatric and young adult subjects with advanced or metastatic solid tumors, primary central nervous system (CNS) tumors, or anaplastic large cell lymphoma (ALCL) with *ALK*, *ROS1*, or *NTRK* alterations. Overall, the study is anticipated to enroll approximately 75 pediatric and young adult subjects.

CARE includes a Phase 1 dose escalation part and a Phase 2 part. Enrollment of subjects into Phase 1 and Phase 2 will proceed concurrently by age as follows:

- Subjects < 12 years old will initially be enrolled in the Phase 1 part to determine the pediatric RP2D for this age group; once the pediatric RP2D is determined, subjects < 12 years old may be enrolled into the Phase 2 part of the study.
- While the Phase 1 dose escalation is ongoing, subjects 12 to 25 years old may be directly enrolled into the Phase 2 part of the study.
- All subjects will receive repotrectinib orally in 28-day cycles. Treatment with repotrectinib may continue until either disease progression, subject withdrawal of consent, or unacceptable toxicity occurs. Subjects with BICR-confirmed progressive disease who are tolerating treatment and, in the opinion of the Investigator, are deriving

Approved v2.0 930213273 1.0

clinical benefit from continuing study treatment, may continue treatment with Sponsor approval.

3.1. *ROS1 or NTRK* Expansion Cohorts

Subjects with advanced solid tumors harboring *ROS1*, *NTRK1*, *NTRK2*, or *NTRK3* rearrangements will be assigned to 6 distinct expansion (EXP) cohorts based on criteria pertaining to their prior anti-cancer treatment history and type of solid tumor. For both the Phase 1 and Phase 2 portions of the TRIDENT-1 study, prior number of lines of anti-cancer therapy (e.g. tyrosine kinase inhibitor [TKI], chemotherapy, and immunotherapy) were determined by evaluating prior treatments collected on the case report form (CRF). These were used to categorize each subject into expansion cohorts according to the entry criteria specified for the Phase 2 portion of the study in protocol Version 11.0 (23 June 2021) for each expansion cohort. Note also that the cohort names (EXP-1 to EXP-6) in the Phase 2 portion of the study collected in the database via the IRT system are specific to each study protocol version due to changes in definition of each cohort through protocol amendments, thus the expansion cohort as specified on the CRF will not be used for the pooled analyses, and the derived expansion cohort based on prior lines of treatment will be used instead.

For the purpose of defining prior TKI therapy, the prespecified list of treatments for each gene rearrangement per the Phase 2 portion of the study in protocol Version 11.0 are included below:

- The ROS1 TKIs considered for subjects treated with one prior TKI treatment in EXP-2 and EXP-4 include: crizotinib, entrectinib, lorlatinib, and ceritinib. Subjects in EXP-1 should be naïve to a ROS1 TKI and should not have received any of these.
- The ROS1 TKIs considered for subjects treated with two prior TKI treatments in EXP-3 include: crizotinib, entrectinib, lorlatinib, ceritinib, brigatinib, ensartinib, and cabozantinib. Other prior ROS1 TKI agents that are not listed could be allowed after discussion with the Sponsor Medical Monitor.
- The TRK TKIs considered for subjects treated with any prior TKI treatment for EXP-6 include: entrectinib, larotrectinib, selitrectinib (LOXO-195), and cabozantinib. Subjects in EXP-5 should be naïve to a TRK inhibitor and should not have received any of these.

3.1.1. *ROS1*+ NSCLC Expansion Cohorts

There are 4 *ROS1*+ NSCLC expansion cohorts. The *ROS1*+ expansion cohorts will include either ROS1 TKI-naïve or ROS1 TKI pretreated subjects and are described in the protocol and the separate ROS1 NSCLC integrated SAP.

3.1.2. *NTRK*+ Advanced Solid Malignancy Expansion Cohorts

There are 2 *NTRK*+ advanced solid tumor expansion cohorts. Each of the *NTRK*+ expansion cohorts will enroll TRK TKI-naïve and TRK TKI-pretreated subjects. For the TRK TKI-naïve *NTRK*+ expansion cohort (EXP-5), there is no limit to the number of prior regimens of cytotoxic chemotherapy or immunotherapy.

Approved v2.0 930213273 1.0

For the TRK TKI-pretreated *NTRK*+ expansion cohort (EXP-6), there is no limit to the number of prior regimens of cytotoxic chemotherapy or immunotherapy and no more than 2 prior TRK inhibitors are allowed.

EXP-5: TRK TKI-naïve *NTRK*+ solid tumors (n=110)

No prior exposure to TRK TKI is allowed.

Any number of prior lines of chemotherapy or immunotherapy allowed. After enrollment of 55 subjects specified for the primary analysis, an additional 55 subjects are to be enrolled for a total of 110 subjects in EXP-5.

EXP-6: TRK TKI-pretreated *NTRK*+ solid tumors (n=80)

Disease progression or intolerant to 1 or 2 prior TRK TKIs.

TRK TKI used in prior lines of treatment are limited to: entrectinib, larotrectinib, selitrectinib (LOXO-195), and cabozantinib. Other prior TRK TKIs that are not listed may be allowed after discussion with the Sponsor Medical Monitor. Note: Any previous exposure of a TRK TKI is considered as one prior line of TKI treatment, e.g., if 2 different TRK TKIs are utilized or the same TRK TKI was used before and after a chemo- or other systemic therapy, it is considered as 2 prior TKIs and the subject would be eligible.

Any number of prior lines of chemotherapy or immunotherapy are allowed. After enrollment of 40 subjects specified for the primary analysis, an additional 40 subjects are to be enrolled for a total of 80 subjects in EXP-6.

Approved v1.0 930213273 1.0

Figure 1: Schema of TPX-0005-01 Phase 1 and Phase 2 Portions by Expansion Cohort

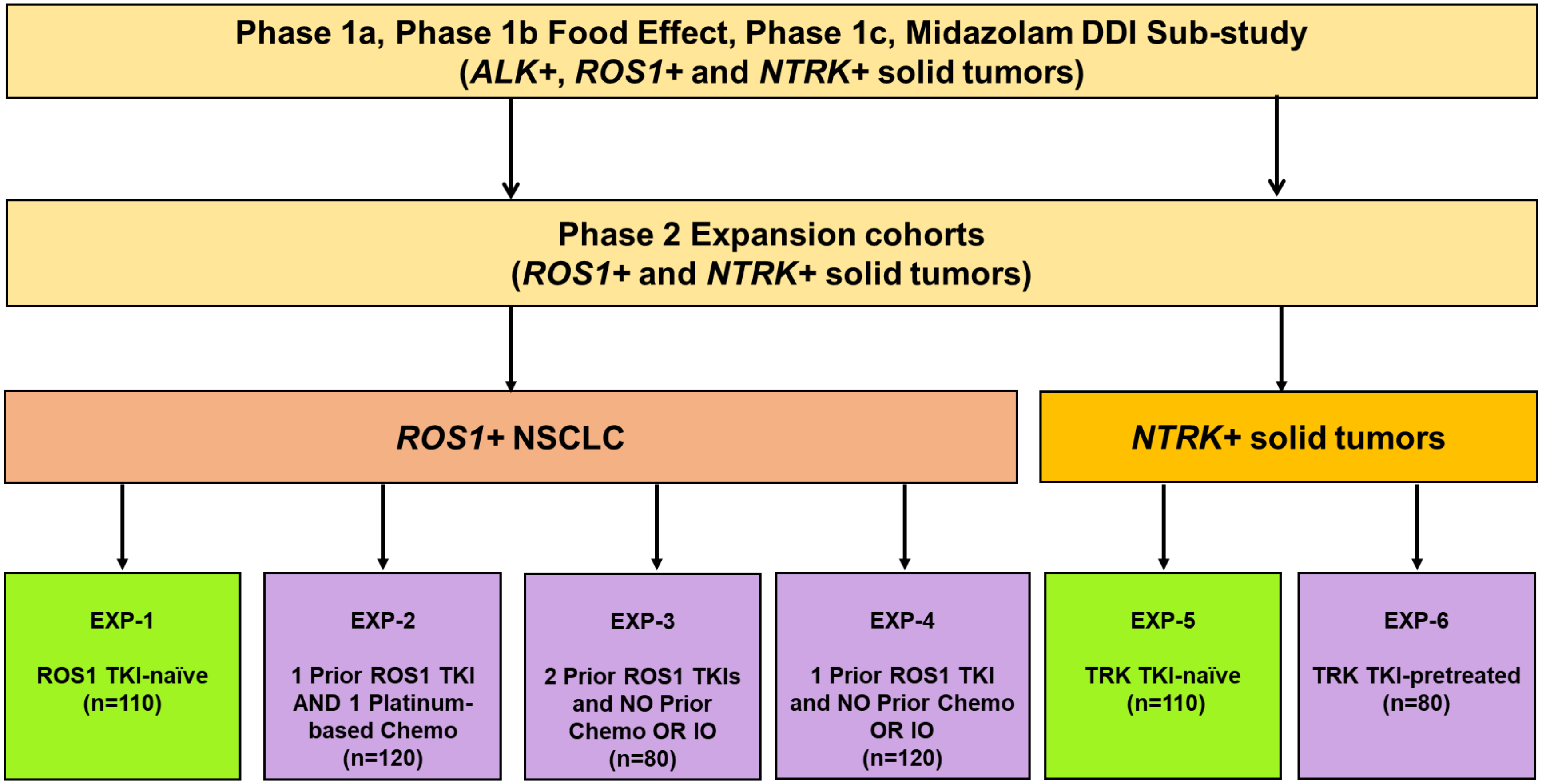

4. ANALYSIS SETS

The analysis sets will define the population of subjects for which each analysis will be performed to meet the integrated analysis objectives. Data for all subjects in the Phase 1 and Phase 2 portions of TRIDENT-1 will be assessed to determine if they meet the criteria for final inclusion into each analysis set prior to locking the study databases. A listing of subjects included in each safety and efficacy analysis set will be provided, along with the criteria they meet for each analysis set.

Figure 2 presents the primary analysis sets for efficacy and safety analyses to support the NDA submission for repotrectinib treatment in subjects with metastatic or locally advanced *NTRK+* solid tumors. In addition, there is an enrolled population that includes all subjects who are enrolled into either the Phase 1 or Phase 2 portion of TRIDENT-1 regardless of whether they receive treatment with repotrectinib. Subjects who are enrolled but do not receive repotrectinib treatment will not be part of the efficacy or safety analyses.

Figure 2: Integrated Safety and Efficacy Analysis Sets

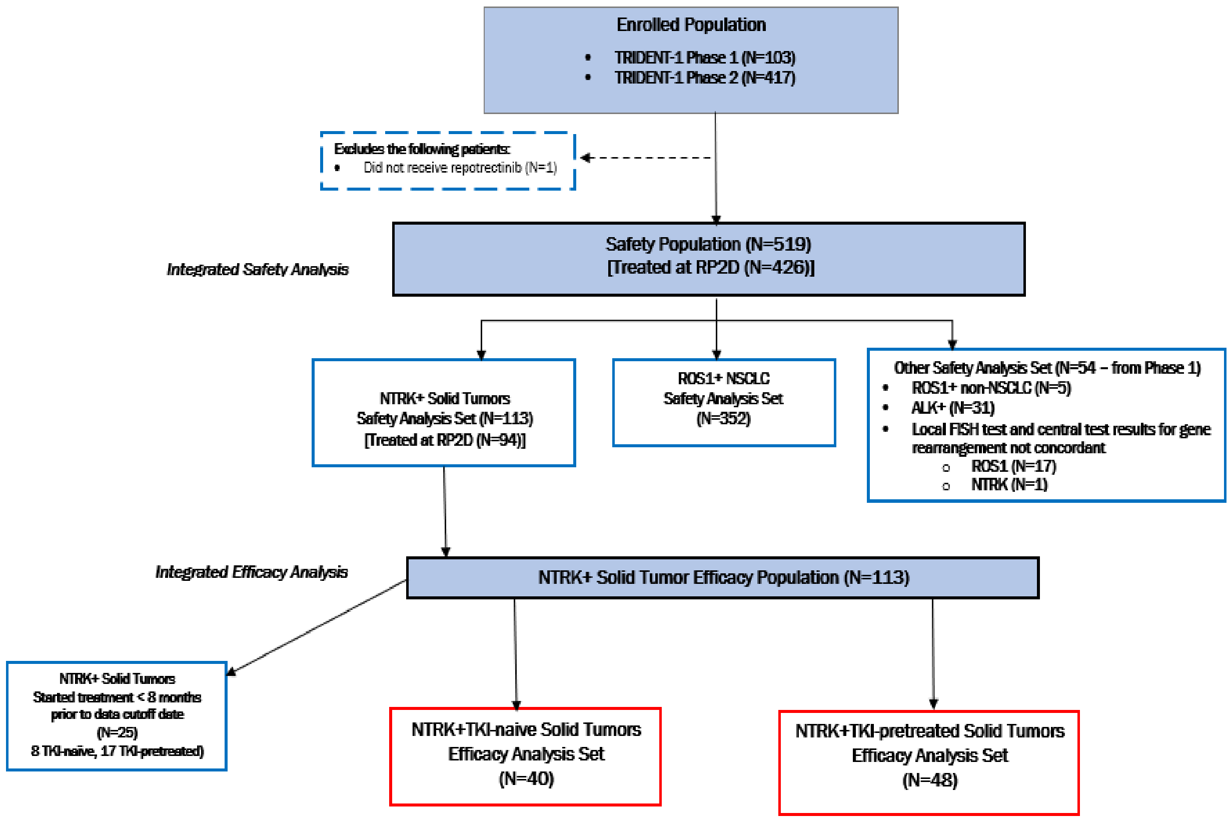

Approved v2.0 930213273 1.0

4.1. Efficacy Analysis Set

The Efficacy Analysis Set for the *NTRK*+ TKI-naïve solid tumor cohort will include all *NTRK*+ metastatic or locally advanced solid tumor subjects in the Phase 1 and Phase 2 portions of TRIDENT-1 study who meet the following criteria:

1. Confirmation by central laboratory (Almac) test for *NTRK* rearrangement per the requirement in each phase of TRIDENT-1 study protocol.
   - In Phase 1, central confirmation of gene fusion status was not required for enrollment. However, subjects with *NTRK*+ based on local FISH test will be retested retrospectively by central laboratory. Only subjects with confirmed *NTRK* positivity by central laboratory will be included in the integrated efficacy analysis, in addition to those *NTRK*+ subjects tested locally by NGS or qPCR.
   - In Phase 2, subjects entering with local results by FISH must have gene fusion status confirmed prospectively prior to enrollment.
2. No prior exposure to a NTRK TKI
3. Started treatment at least 8 months prior to data cutoff date (6 months of follow-up for tumor assessment after first post-baseline scan)

The Efficacy Analysis Set for the *NTRK*+ TKI-pretreated solid tumor cohort will include all *NTRK*+ metastatic or locally advanced solid tumor subjects in the Phase 1 and Phase 2 portions of TRIDENT-1 study who meet the following criteria:

1. Confirmation by the central laboratory test for *NTRK* rearrangement per the requirement in each phase of TRIDENT-1.
   - In Phase 1, central confirmation of gene fusion status was not required for enrollment. However, subjects with *NTRK*+ based on local FISH test will be retested retrospectively by central laboratory. Only subjects with confirmed *NTRK* positivity by central laboratory will be included in the integrated efficacy analysis, in addition to those *NTRK*+ subjects tested locally by NGS or qPCR.
   - In Phase 2, subjects entering with local results by FISH must have gene fusion status confirmed prospectively prior to enrollment.
2. Disease progression or intolerant to at least 1 prior line of a NTRK TKI treatment, including:entrectinib, larotrectinib, selitrectinib (LOXO-195), and cabozantinib. Other prior TRK TKIs that are not listed may be allowed after discussion with the Sponsor Medical Monitor.
3. Started treatment at least 8 months prior to data cutoff date (6 months of follow-up for tumor assessment after first post-baseline scan).

Subject disposition, demographics and baseline disease characteristics, important protocol deviations, treatment exposure, and efficacy will be summarized for the Efficacy Analysis Set *NTRK*+ TKI-naive solid tumor cohort and the *NTRK*+ TKI-pretreated solid tumor cohort.

Approved v2.0 930213273 1.0

4.2. Safety Analysis Set

The Safety Analysis Set will include all subjects who are enrolled and have received any dose of repotrectinib in either the Phase 1 or Phase 2 portion of TRIDENT-1.

As supportive data, the Safety Analysis Set will be categorized by the following three subgroups: *ROS1+* NSCLC subjects, *NTRK+* subjects with solid tumors, and any other treated subjects, including subjects with *ROS1+* non-NSCLC, *ALK+* gene fusions, and any gene fusions with discordant results between local FISH test and central laboratory test. Please note that subjects from Phase 1 with *ROS1+ or NTRK+* identified by local FISH testing will be retested retrospectively. Subjects with results that are not concordant or locally tested with ALK+ without confirmation will be included in the subgroup of other treated subjects.

Subject disposition, demographics and baseline disease characteristics, treatment exposure, and safety will be analyzed for the Safety Analysis Set and by the three subpopulations: *ROS1+* NSCLC subjects, *NTRK+* subjects with solid tumors, and any other treated subjects.

Approved v2.0 930213273 1.0

5. STATISTICAL ANALYSIS METHODS AND CONSIDERATIONS

5.1. Sample Size Determinations

5.1.1. EXP-5 TRK TKI-Naïve NTRK+ Solid Tumors

Fifty-five TRK TKI-naïve *NTRK+* (*NTRK1, NTRK2,* and *NTRK3*) eligible solid tumor subjects will be enrolled into this cohort. For the TRK-naïve expansion cohort, if the ORR is 35% or less, then it is not considered as effective. Using Table 2, if 27 subjects out of 55 subjects have a confirmed objective response (ORR = 49.1%; 95% CI: 35.4 – 62.9) where the lower limit of the 95% CI is > 35%, repotrectinib is considered to be efficacious in this subject expansion cohort.

Table 2: Estimated ORRs and 95% Confidence Intervals Using Sample Size N = 55 for EXP-5

# Responses n/N	Overall Response Rate (95% CI)
20/55	36.4% (23.8 – 50.4)
22/55	40% (27.0 – 54.1)
**27/55**	**49.1% (35.4 – 62.9)**
28/55	50.9% (37.1 – 64.7)
33/55	60% (45.9 – 73.0)

After enrollment of 55 subjects in the EXP-5 cohort as specified above for the primary analysis, an additional 55 subjects are to be enrolled for a total of 110 subjects in EXP-5.

5.1.2. *NTRK+* TKI-pretreated Solid Tumors

Subjects in Phase 1 of the study will be pooled according to the Phase 2 entry criteria of *NTRK+* TKI-pretreated NSCLC cohorts, as detailed in Section 4.1. In Phase 1, central confirmation of gene fusion status was not required for enrollment. However, subjects with NTRK positivity based on local FISH test will be retested retrospectively by Almac central laboratory. Only subjects with confirmed *NTRK* positivity by central laboratory will be included in the integrated efficacy analysis, in addition to those *NTRK+* subjects tested locally by NGS or qPCR. Subjects who are pretreated with at least one NTRK TKI will be included.

5.1.2.1. EXP-6 TRK TKI-Pretreated NTRK+ Advanced Solid Tumors

Forty TRK TKI-pretreated *NTRK+* eligible solid tumor subjects will be enrolled into the cohort with 1 or 2 prior TRKi TKIs treatments. For this cohort, if the ORR is 10% or less, then it is assumed that repotrectinib is not effective. Using Table 3, if 9 out of 40 subjects have a confirmed objective response (ORR = 22.5%; 95% CI: 10.8 – 38.5) where the lower limit of 95% CI >10%, repotrectinib will be considered efficacious in this cohort.

Approved v2.0 930213273 1.0

Table 3: Estimated ORRs and 95% Confidence Intervals using Sample Size N = 40 for EXP-6

# Responses n/N	Overall Response Rate (95% CI)
8/40	20.0% (9.1 – 35.7)
**9/40**	**22.5% (10.8 – 38.5)**
10/40	25.0% (12.7 – 41.2)

After enrollment of 40 subjects in the EXP-6 cohort as specified above for the primary analysis, an additional 40 subjects are to be enrolled for a total of 80 subjects in EXP-6.

5.2. General Considerations

- **Software**: Data listings, summaries, figures, and statistical analyses will be generated using SAS® Version 9 or above.
- **Missing Data**: All analyses and descriptive summaries will be based on the observed data. Unless otherwise specified missing data will not be imputed.
- **Partial dates:** Dates missing the day of the month, or both the day and month of the year will adhere to the following conventions:
  - If the onset date of an adverse event or start of a concurrent therapy is missing day and/or month components, the onset date will be set to:
    - The day of first dose of repotrectinib if it may have occurred in the same month as that of the first dose of repotrectinib, or if only the year is available, and it is within the same year as the first dose of repotrectinib
    - January 1 of the stated year, as long the year is after the first dose of repotrectinib and month and day are missing
    - The first of the month if the day is missing and non-missing components exclude the possibility that it occurred on the day of the first dose of repotrectinib
    - Completely missing dates will not be imputed; adverse events will be identified as treatment emergent
  - If the resolution date of an adverse event or end date of a concurrent therapy is missing day and/or month components, the date will be set to:
    - December 31 of the year of occurrence if month and day are missing
    - The last day of the stated month if day is missing
    - Based on the above, if the imputed date is later than the subject's discontinuation (end of study) date, and the event is not ongoing, the subject's discontinuation date will be used
  - Subsequent anticancer therapy, radiotherapy, and surgical procedures:
    - A method similar to that of partial dates for onset will be used, using documented progression date + 1 in place of the day of first dose of

Approved v2.0 930213273 1.0

repotrectinib (e.g. these dates should default to a date following progression if it is possible to have occurred in the same month or year)

- **Baseline Measurements**: The last measurement on or prior to the date of the first repotrectinib dose will serve as the baseline measurement. For evaluations with a time component, time of first dose of repotrectinib will be taken into account to ensure assessments evaluated prior to dosing are included in baseline value calculations. Values that are to be taken on the first dose date prior to dosing per the protocol will be considered in the baseline calculation only if a time is not collected. As for ECGs, the last average value of triplicate prior to first repotrectinib will be derived as baseline value.
- **Data Presentation**: Categorical variables will be summarized in frequency tables, with the counts and percentage of subjects in each category. Percentages given in the summary tables will be rounded and thus may not always add up to exactly 100 percent. For continuous variables, summary statistics will include N, mean, standard deviation, median, and minimum and maximum values (range).
- **Duration**: Duration is calculated as:
  - Duration (days): (End Date – Start Date + 1).
  - Duration (weeks): (End Date – Start Date + 1)/7
  - Duration (months)[1]: (End Date – Start Date + 1)/30.4375
  - Duration (years)[2]: (End Date – Start Date + 1)/365.25
- **Age:** Age will be computed from the date of birth to the date of informed consent. For countries where date of birth cannot be reported for regulatory reasons, age at the time of signing the ICF (study entry) collected on the CRF will be used for analysis.

5.3. Disposition of Subjects

A disposition table will summarize:

- Number of subjects enrolled
- Number of subjects treated with repotrectinib (at any dose)
- Number and percentage of subjects ongoing on treatment
- Number and percentage of subjects discontinued from study treatment and reasons for treatment discontinuation
- Number and percentage of subjects ongoing in long-term follow-up after discontinuation of study treatment
- Number and percentage of subjects discontinued from study and reasons for study discontinuation.

[1] Days in a month = Average number of days in a year/12.
[2] Average days in a year = 365.25, reflecting the Julian Year of three years with 365 days each and one leap year of 366 days.

Approved v2.0 930213273 1.0

The reason for treatment discontinuation and end-of-study will be summarized using the categories specified in the End of Treatment, and End-of Study CRF. When the set of reasons are different between the Phase 1 and Phase 2 study CRFs, they will be mapped and combined together as closely as possible to represent the original reason as displayed in below Table 4.

Table 4: Reason for Treatment and Study Discontinuation

TRIDENT-1 Phase 1	TRIDENT-1 Phase 2	ISS/ISE
**Reason for Treatment Discontinuation**		
Radiographic Disease Progression	Radiographic progression	Radiographic Disease Progression
Adverse Event	Adverse Event	Adverse Event
Investigator Decision<br>Clinical Disease Progression	Physician Decision	Physician Decision
Protocol Violation requiring discontinuation of study treatment<br>Subject is not compliant with study procedures		Protocol Deviation
Lost to Follow-Up	Lost to Follow-Up	Lost to Follow-Up
Withdrawal of Consent	Withdrawal of Consent	Withdrawal of Consent
TP Therapeutic's early termination of study	Sponsor early termination of study	Early Termination of Study
Death	Death	Death
**Reason for Study Discontinuation**		
Radiographic Disease Progression		Disease Progression
Adverse Event		Adverse Event
Investigator Decision<br>Clinical Disease Progression		Physician Decision
Protocol Violation requiring discontinuation of study treatment<br>Subject is not compliant with study procedures		Protocol Deviation
Lost to Follow-Up	Lost to Follow-Up	Lost to Follow-Up
Withdrawal of Consent	Withdrawal of Consent	Withdrawal of Consent
TP Therapeutic's early termination of study	Sponsor early termination of study	Early Termination of Study
Death	Death	Death

The number and percentage of subjects who form the Safety Analysis Set and Efficacy Analysis Set will be summarized. A listing of subjects included in each safety and efficacy analysis set will be provided, along with the criteria they meet for each analysis set.

Approved v1.0 930213273 1.0

Descriptive statistics (median, minimum, maximum) will be used to summarize the overall follow-up time in month for each subject, defined as: ((Data cutoff date - Date of first dose of repotrectinib) +1)/30.4375.

5.4. Protocol Violations and Deviations

Protocol violations and deviations will be recorded in an external spreadsheet maintained by the CRO and reviewed prior to database lock. Violations and deviations will be categorized into the following categories:

- Assessment - efficacy
- Assessment - safety
- Exclusion
- Inclusion
- Informed consent
- Lab/endpoint data
- Other
- Overdose/misuse
- Prohibited co-medication
- Study Drug
- Visit Window

Other protocol deviation categories may be identified during the study. Protocol deviations and violations will be categorized as important or non-important prior to each database lock.

Important deviations will be summarized by category for the Efficacy and Safety Analysis Sets.

5.5. Demographics and Baseline Characteristics

Demographics and baseline characteristics will be summarized based on CRF data for Efficacy Analysis Set and Safety Analysis Set. The following demographics and baseline characteristics will be presented:

Demographics

- Age (in years) as continuous variable
- Age (in years) categories: ≥12 to <18, ≥18 to <65, ≥65 to <75, ≥75
- Gender (male, female)
- Race (American Indian or Alaskan Native, Asian, White, Native Hawaiian or Pacific Islander, Black or African American, Other, Not allowed by local law)
- Region (US, Asia, Other)
    - Asian region includes subjects enrolled at sites in China, Hong Kong, Japan, Singapore, South Korea, and Taiwan.

Approved v2.0 930213273 1.0

- Ethnicity (Hispanic or Latino, Not Hispanic or Latino, Not allowed by local law)
- Height (in cm) as continuous variable
- Baseline weight (in kg) as continuous variable
- Body Mass Index at Baseline (in kg/m^2) as continuous variable
- Baseline Performance Status:
  - ECOG (0 or 1) - for ages ≥ 18 years

o Smoking Status, including timing (past and current) and type of tobacco use. Based smoking status, subjects will be categorized by smoking history into the following categories:

- never smoker
- former smoker
- current smoker

Disease characteristics

- Disease stage at diagnosis
- Disease stage at study entry
- Tumor type (breast cancer, colorectal cancer, cholangiocarcinoma, gallbladder cancer, gastric cancer, GIST, glioblastoma, head and neck cancer, malignant esophageal cancer, melanoma, mucoepidermoid carcinoma, neuroendocrine tumor, NSCLC, pancreatic cancer, peripheral nerve sheath tumor, prostate cancer, renal cancer, salivary gland cancer, sarcoma, thyroid cancer, uterine cancer, unknown tumor type)
- Histological classification
- Brain metastasis by Investigator and BICR (Yes/No)
  - Defined as having a target and/or non-target lesion in the brain selected at baseline for RECIST
- Time since diagnosis (years) as a continuous variable
- TNM stage at diagnosis
- TNM stage at study entry
- Prior surgery (Yes/No)
- Prior radiotherapy (Yes/No)
- Type of prior systemic therapy (TKI, Chemotherapy with/without Immunotherapy, Immunotherapy Alone, Other Targeted Therapy)
- Number of lines of prior systemic therapy (median and categorized as 0, 1, 2, ≥3)
- Number of lines of prior TKI therapy (median and categorized as 0, 1, 2, ≥3)

Approved v2.0 930213273 1.0

- Number of lines of prior chemotherapy with/without immunotherapy (median and categorized as 0, 1, 2, ≥3)
- Number of lines of prior immunotherapy alone (median and categorized as 0, 1, 2, ≥3)
- Prior use of platinum-based chemotherapy (Yes, No)
- Type of most recent prior systemic therapy (TKI, chemotherapy with/without Immunotherapy, Immunotherapy Alone, Other Targeted Therapy)
- Best response to most recent prior systemic therapy (CR, PR, SD, PD, Other)
- Reason for discontinuation of most recent prior systemic therapy (completed therapy, progressive disease, adverse event, other)
- Resistance Mutation (solvent front, gatekeeper, other, none)

5.6. Efficacy Analysis

Efficacy analysis will be performed using the Efficacy Analysis Set.

Primary endpoints will be based on the radiologic assessments evaluated by the BICR for Phase 1 and Phase 2 of the TRIDENT-1 protocol using Response Evaluation Criteria in Solid Tumors (RECIST) v1.1 (Eisenhauer 2009). Details of radiological assessments by BICR are included in the Independent Review Charters for Phase 1 and Phase 2 portion of TRIDENT-1 study respectively. Phase 2 scan data are assessed using a two-reader model with adjudication for disagreement between two readers, while Phase 1 scan data are assessed using a single reader model with overall oncology review. Response evaluations by Investigator using RECIST v1.1 will be analyzed as supportive.

Efficacy will be summarized for the two Efficacy Analysis Sets: *NTRK*+ TKI-naive solid tumor cohort and *NTRK*+ TKI-pretreated solid tumor cohort.

5.6.1. Analysis of Primary Efficacy Endpoints

The primary endpoint is cORR, as assessed by BICR using RECIST v1.1 (Eisenhauer 2009*)*. Note that the term cORR may be used interchangeably in this document with ORR due to it being understood that all best overall responses have been confirmed.

Best Overall Response (BOR) will be determined by BICR based on the Overall Response assessments at all timepoints in the following order: Complete Response (CR) > Partial Response (PR) > Stable Disease (SD) > Progressive Disease (PD) > Not Evaluable (NE)>Missing. If minimum time for SD (at least 6 weeks after the first dose of repotrectinib) at a timepoint has not been met, the SD at that timepoint will be considered NE in determining BOR. A confirmed CR or PR will be a response that persists on a repeat-imaging performed at least 4 weeks after initial documentation of response. For confirmation, it is not required that the second assessment of response immediately follow the initial response, and a subject may have intervening SDs, but cannot be identified to have progressed prior to response confirmation. Subjects with a confirmed objective response (i.e., CR or PR) will be referred to as responders. Otherwise, subjects will be considered non-responders, which include PD, SD, NE, and missing. BOR will be based on assessments collected after the first dose of study drug until disease

Approved v2.0 930213273 1.0

progression; assessments collected after the date of new cancer treatment will not be considered. BOR will be provided directly by the BICR as derived based on the BICR Charter; BOR based on Investigator assessments will be derived using the same rules as detailed in the BICR Charter. The ORR will be defined as the proportion of subjects with a confirmed CR or PR.

The ORR will be reported as the proportion of responders by RECIST v1.1 along with the corresponding 2-sided 95% Clopper-Pearson exact confidence interval (CI) (Clopper 1934).

Analyses of ORR will be presented using BICR assessments as the primary analysis and investigator assessment as a supportive analysis.

A waterfall plot of the maximum percentage of tumor size (sum of diameters) reduction in target lesions from baseline; measurements collected after the date of new cancer treatment will not be included in the display. The bars of the waterfall plot will be colored differently to represent each tumor type.

Individual subject data will also be displayed using swimmer plot showing timeline of events for each subject, including duration of study therapy, start of response (CR and/or PR), progression or ongoing at the time of analysis will be presented by cohort. The bars of the swimmer plot will be colored differently to represent each tumor type.

5.6.2. Analysis of Secondary Endpoints

5.6.2.1. Duration of Response

Duration of response (DOR) will be defined from the first date of objective response (either CR or PR) to first documentation of radiographic disease progression, as assessed by BICR per RECIST v1.1, or death due to any cause, whichever occurs first. DOR events and censoring will be based on the methods described in Table 5.

Table 5: Outcome and Date Values for DOR Derivations

Situation	Date of Event or Censoring	Outcome
No documented radiologic progression before start of new anticancer therapy or tumor-related surgery	Date of last tumor assessment with documented non-progression prior to start of new anticancer therapy or tumor-related surgery	Censored
Death or radiologic progression after two or more consecutive missed scheduled visits for tumor assessment	Date of last tumor assessment with documented non-progression	Censored
Documented radiologic progression before start of new anticancer therapy or tumor-related surgery	First date of tumor assessment with documented progression	Event
Death without documented radiologic progression within two tumor assessments window after last evaluable tumor assessment before start of new anticancer therapy or tumor-related surgery	Date of death	Event

Approved v1.0 930213273 1.0

DOR will only be calculated for the subgroup of subjects with a confirmed objective tumor response (PR or CR) and will be analyzed and presented graphically using the Kaplan-Meier method. If estimable, the median DOR and 95% CI for the median will be constructed based on a linear transformed CI for the survival function S(t). The first and third quartiles will also be summarized similarly.

Landmark analysis will be presented for the proportion subjects with observed DOR and 95% CI, using the Clopper-Pearson method, of at least 6, 9, 12, 18, and 24 months. The landmark analysis will be also presented with Kaplan-Meier survival probability and 95% CI will be derived based on Greenwood's formula for variance derivation and the linear transformation applied on the survival function S(t) at 6, 9, 12, 18, and 24 months. The principal Kaplan-Meier assessment of DOR, for the time points at 6, 9, and 12 months, will be based on the data cut when majority of responders would be at least 6 months post onset of first response. The principal Kaplan-Meier assessment of DOR, for the 18-month and 24-month time points, will be based on the data cut when the majority of responders would be at least 12 months post onset of response.

Supportive analyses for DOR will be presented based on investigator assessments.

5.6.2.2. Time to Response

Time to response (TTR) will be defined as the time from the first dose of repotrectinib to the first documentation of objective response (either CR or PR), as assessed by BICR per RECIST v1.1. TTR will only be calculated for the subgroup of subjects with a confirmed objective tumor response (PR or CR). Summary statistics (mean, SD, median, min, and max) will be presented for TTR.

Supportive analyses for TTR will be presented based on Investigator assessments.

5.6.2.3. Clinical Benefit Rate

The clinical benefit rate (CBR) will be defined as the proportion of subjects with a BOR of confirmed CR, confirmed PR, or SD. SD refers to a condition where the tumor has neither sufficient shrinkage to qualify for PR or CR nor sufficient increase to qualify for at least 6 weeks after the first dose of repotrectinib, as assessed by BICR per RECIST v1.1. The CBR and its exact 95% CI will be calculated using Clopper-Pearson method.

Supportive analyses for CBR will be presented based on Investigator assessments.

5.6.2.4. Progression-Free Survival

Progression-free survival will be defined as the time from the first dose of repotrectinib to first documentation of radiographic disease progression per RECIST v1.1 or death due to any cause (whichever occurs first). PFS will be based on BICR data, while PFS based on Investigator assessment will be supportive.

For subjects discontinuing study treatment prior to documented radiographic progression by BICR, tumor assessments should continue approximately every 2 cycles or at the current scan interval at the time of treatment discontinuation until radiographic evidence of disease progression by BICR, the start of a subsequent anticancer therapy, or withdrawal of consent, whichever is first.

Approved v1.0 930213273 1.0

PFS event and censoring derivations will be based on Table 6.

Table 6: Outcome and Date Values for PFS Derivations

Situation	Date of Event or Censoring	Outcome
No documented radiologic progression before start of new anticancer therapy, including tumor-related surgery	Date of last tumor assessment with documented non-progression prior to start of new anticancer therapy or tumor-related surgery	Censored
Death or radiologic progression after two or more consecutive missed scheduled visits for tumor assessment	Date of last tumor assessment with documented non-progression	Censored
Documented radiologic progression before start of new anticancer therapy or tumor-related surgery	First date of tumor assessment with documented progression	Event
Death without documented radiologic progression within two tumor assessments window after last evaluable tumor assessment before start of new anticancer therapy or tumor-related surgery	Date of death	Event
No evaluable baseline or post-baseline tumor assessment with no documented death as an event	Date of first dose of study treatment	Censored

PFS will be analyzed and presented graphically based on Kaplan-Meier methodology. The number of subjects with events and censored will be summarized. A 95% confidence interval constructed based on a linear transformed CI for the survival function S(t) using the Greenwood variance estimate will be provided for the median, the first quartile, and the third quartile of PFS. The minimum and maximum values within all subjects (either with events or censored) will be summarized.

The Kaplan-Meier survival probability and 95% CI using Greenwood variance estimate and the linear transformation applied on the survival function S(t) will be presented at 6, 9, 12, 18, and 24 months. The principal Kaplan-Meier assessment of PFS, for the time points at 6, 9, and 12 months, will be based on the data cut when majority of subjects would be at least 6 months post first dose of study drug. The principal Kaplan-Meier assessment of PFS for the 18-month and 24-month time points, will be based on the data cut when majority of patients would be at least 12 months post first dose of study drug.

Analyses for PFS will be presented based on BICR and Investigator assessments.

5.6.2.5. Overall Survival

Overall Survival (OS) is defined as the time from the first dose of repotrectinib to the date of death due to any cause. For subjects still alive at the time of analysis, the OS time will be censored on the last date the subjects were known to be alive. For subjects who are lost to follow up, OS will be censored at the last date the subject is known as alive before being lost to follow up.

Approved v2.0 930213273 1.0

OS event and censoring derivations will be based on Table 7.

Table 7: Outcome and Date Values for OS Derivations

Situation	Date of Event or Censoring	Outcome
Subject continues on treatment	Data cutoff date (defined as the date through which all visits will be included for analysis)	Censored
Subject off treatment, but not off study	Date of last survival follow-up or latest date in the visit domain (through data cutoff date) if survival follow-up visit has not yet occurred	Censored
Subject is off study and lost-to-follow up	Date last known alive from the survival follow-up CRF	Censored
Subject is off study due to reason other than lost-to-follow	End of study date	Censored
Death at any time	Date of death	Event

OS will be analyzed and presented graphically based on Kaplan-Meier methodology. The number of subjects with events and censored will be summarized as well. Minimum and maximum values of OS within all subjects (either with events or censored) will be calculated. OS will be analyzed and presented graphically based on Kaplan-Meier methodology. The median and 95% CI constructed based on a linear transformed CI for the survival function S(t) using the Greenwood variance estimate will be displayed; the first and third quartiles will be summarized similarly.

The Kaplan-Meier survival probability and 95% CI using Greenwood variance estimate and the linear transformation applied on the survival function S(t) will be presented at 12, 18 and 24 months.

5.6.2.6. Intracranial Objective Response Rate

Besides the overall tumor assessment for the whole body by BICR, subjects in Phase 2 with measurable brain metastasis at baseline will be evaluated by BICR for intracranial response per modified RECIST v1.1. Subjects in Phase 1 were not evaluated for intracranial response separately by BICR. Instead, the intracranial response and progression will be calculated based on the target, non-target, and new lesions from the brain using the data for overall tumor assessment for the whole body by BICR.

After tumor assessment for the whole body using RECIST v1.1, the BICR for Phase 2 will subsequently assess the intracranial response in subjects with brain metastases at baseline per BICR, and the modified RECIST v1.1 criteria will be applied. Up to 5 CNS target lesions can be selected within the brain. The target lesions in brain will have a minimum diameter of 5 mm as assessed using gadolinium contrast enhancement without any preference toward lesion(s) which have not undergone prior intervention. The same rules as in RECIST v1.1 will be applied to define responses and progression based on assessment of target lesions, non-target lesions, and new lesions within brain. A separate BICR charter based on modified RECIST criteria has been written for intracranial response assessment.

Approved v2.0 930213273 1.0

CNS activity by BICR was not evaluated separately from the rest of the body in Phase 1. However, the intracranial response and progression will be calculated for subjects who have lesions in the brain selected as target lesions for the whole-body tumor assessment by BICR. The BICR tumor assessments for target, non-target, and new lesions in the brain will be used to determine overall timepoint response, best overall response, duration of response, and progression-free survival.

Intracranial ORR is defined as the percent of subjects with a confirmed CR or PR achieved in intracranial lesions in subjects with measurable brain metastasis at baseline per BICR and will be provided along with the corresponding two-sided 95% Clopper-Pearson exact CIs (Clopper 1934).

Note that this analysis will be performed by BICR only and not by Investigator. Due to differences in the Phase 1 and Phase 2 evaluations of the modified RECIST criteria, these analyses will not be integrated across phases. These analyses are described in this plan but will be performed separately for each phase of the protocol and can be found in their individual clinical study reports.

5.6.3. Subgroup Analysis

Subgroup analyses of ORR, DOR, TTR, and CBR will be performed by demographic and baseline risk factors using the primary efficacy analysis population for the *NTRK*+ TKI-naive solid tumor cohort and the *NTRK*+ TKI-pretreated solid tumor cohort, respectively. Subgroup efficacy analysis will be conducted for the following subgroups:

- Age in years: ≥12 to <18, ≥18 to <65, and ≥65 to <75, ≥75
- Sex: Male and Female
- Race: American Indian or Alaskan Native, Asian, White, Native Hawaiian or Pacific Islander, Black or African American, Other, and Not allowed by local law
- Region: US, Asia, and Other
    - Asian region includes subjects enrolled at sites in China, Hong Kong, Japan, Singapore, South Korea, and Taiwan
    - Other includes Australia, Belgium, Canada, Denmark, France, Germany, Hungary, Italy, Netherlands, Poland, Spain, and United Kingdom.
- ECOG performance status: 0 and 1
- Solvent Front Mutation (for the *ROS1*+ TKI pretreated NSCLC cohorts)
- Tumor type (breast cancer, colorectal cancer, cholangiocarcinoma, gallbladder cancer, gastric cancer, GIST, glioblastoma, head and neck cancer, malignant esophageal cancer, melanoma, mucoepidermoid carcinoma, neuroendocrine tumor, NSCLC, pancreatic cancer, peripheral nerve sheath tumor, prostate cancer, renal cancer, salivary gland cancer, sarcoma, thyroid cancer, uterine cancer, unknown tumor type)
- Study Phase: Phase 1 vs. Phase 2
- Brain metastasis by Investigator and BICR

Approved v2.0 930213273 1.0

    - Defined as having a target and/or non-target lesion in the brain selected at baseline for RECIST
- Any prior treatment with larotrectinib: Yes (for analysis of ORR and DOR only in TKI-pretreated cohorts)
- Any prior treatment with entrectinib: Yes (for analysis of ORR and DOR only in TKI-pretreated cohorts)

Subgroup efficacy analysis for intracranial objective response only will be conducted for the following subgroup:

- Prior intervention (irradiation or surgery) on CNS lesions within 60 days prior to starting treatment with repotrectinib: yes vs. no

5.6.4. Exploratory Analysis

Exploratory analyses of efficacy endpoints are included below.

5.6.4.1 Subsequent Anti-Cancer Therapies

Subjects in the Efficacy Analysis Set who have ended treatment with repotrectinib and receive a subsequent anti-cancer therapy prior to their End of Study visit will be analyzed. The following will be analyzed for the first line of subsequent anti-cancer therapy received:

- Type of first subsequent anti-cancer therapy: TKI alone, TKI with chemotherapy, chemotherapy with/without immunotherapy; immunotherapy alone, other targeted therapy, other therapy, not reported, n (%)
- Time from end of treatment with repotrectinib to start of first subsequent anti-cancer therapy defined as (Start Date of first subsequent anti-cancer therapy – End of treatment date) +1: mean, standard deviation, median, minimum, maximum

5.6.4.2 Analysis of Tumor Location and Progression

The number of anatomical sites with target lesions at baseline (1, 2, 3, 4, 5) will be summarized by n (%), where anatomical sites are categorized through medical review to brain, lung, liver, adrenal, bone, lymph node or other. Additionally, the number of target lesions selected at baseline across all anatomical sites (1, 2, 3, 4, 5) will be summarized by n (%).

Subjects who progress on study in a target, non-target, and/or new lesion will have the first site of progression summarized, where anatomical sites are categorized by brain, lung, liver, adrenal, bone, lymph node and other through medical review. Time (in months) on treatment after progression is defined as ((End of treatment date – Date of first progression) +1)/30.4375.and for patients with treatment ongoing, it is derived as ((Data cutoff date – Date of first progression) +1)/30.4375. Descriptive statistics (mean, standard deviation, median, minimum, maximum) will be summarized for those subjects who stay on treatment after progression. Additionally, time on treatment after progression will be categorized as <1 month, ≥1 month, ≥3 months, ≥6 months, and ≥12 months and summarized by n (%) where subjects may be included in more than one category.

Approved v2.0 930213273 1.0

5.7 Safety Analyses

Safety analysis will be performed for the Safety Analysis Set and by the three subpopulations: *ROS1*+ NSCLC subjects, *NTRK*+ subjects with solid tumors, and other treated subjects. The presentation of primary safety analysis as agreed with the agency will be presented for the overall safety population of all patients treated with at least 1 dose of repotrectinib and two sub populations of patients treated at RP2D and patients treated at RP2D with NTRK+ solid tumors. A subset of the safety analyses may be performed by expansion cohort as defined in the Phase 2 portion of the protocol (see Section 3.1) rather than the three subpopulations specified above.

5.7.1 Extent of Exposure

Extent of exposure to repotrectinib will be assessed by summarizing the duration of treatment (months), cumulative dose on study (mg), average dose intensity (mg/day), relative dose intensity (%), and number of treatment cycles. The number of subjects treated at the RP2D of 160 mg QD for 14 days followed by 160 mg BID will be summarized. For subjects treated at the RP2D, the number subjects increasing dose from 160 mg QD to 160 mg BID at approximately Day 15, per protocol will be analyzed. Duration of treatment (months), cumulative dose (mg), average dose intensity (mg/day), and relative dose intensity (%) will be summarized using descriptive statistics. The number of subjects increasing dose from 160 mg QD to 160 mg BID, per protocol will be summarized categorically (n and %). Number of treatment cycles will be summarized by descriptive statistics and categorically (1, 2, 3, 4, 5, 6, > 6, > 12, > 15, > 18 cycles). Number of treatment cycles per subject will be calculated as the duration of treatment divided by the length of a cycle (28 days) and then increased to the next integer. For example, subjects on for 1.1 cycles would be increased to have been in 2 cycles. Therefore, subjects who receive any part of a cycle will be considered to have received that cycle.

Duration of treatment with repotrectinib (months) will be calculated as (last dose date - first dose date + 1)/30.4375. Subjects who are still on treatment at the time of data cutoff date will have the duration of treatment calculated using the data cutoff date as the last dose date.

Cumulative dose on study (mg) will be calculated as the actual total dose of repotrectinib taken on study.

Average dose intensity (mg/day) will be calculated as the cumulative dose (mg) divided by duration of treatment (days).

Relative dose intensity (%) will be calculated as (average dose intensity divided by expected dose intensity) x 100%, where expected dose intensity (mg/day) is defined as expected cumulative dose (mg) divided by duration of treatment.

5.7.2 Adverse Events

Adverse events will be graded according to the Common Terminology Criteria for Adverse Events (CTCAE) v4.03 and coded to preferred term and system organ class (SOC) using MedDRA version 25.0 (March 2022).

All adverse events (AEs) reported during the AE reporting period (inclusive AEs after the first dose of repotrectinib through the 28-day after receipt of the last dose of study drug) will be considered as treatment-emergent adverse events (TEAEs).

Approved v2.0 930213273 1.0

An overview summary of AE event types, including counts and percentages of subjects with any TEAEs, TEAEs leading to any dose modifications (dose reduction or drug interruption), TEAEs leading to dose reduction, TEAEs leading to drug interruption; TEAEs leading to treatment discontinuation; serious adverse events (SAEs); Grade ≥ 3 TEAEs; fatal TEAEs, and TEAEs of maximum CTCAE grade will be presented. This summary will be repeated for treatment-related events.

Incidence rates will be summarized based on reporting of frequencies and percentages by MedDRA SOC and preferred term. In addition, AE incidence rates will also be further summarized by maximum CTCAE grade. Subjects with multiple occurrences of events will only be counted once at the maximum CTCAE grade and strongest relationship at the maximum grade to study drug for each preferred team, SOC, and overall, for the specified event type. Treatment-related TEAEs will be summarized similarly. Treatment-related AEs are those judged by the Investigator to be at least possibly related to the study drug. Deaths that occur within 28 days after the last dose of study drug are defined as on-study deaths and reported as SAEs within the required reporting period unless they are due to disease progression in which they would not be reported as an SAE in Phase 2. In the Phase 1 portion of the study, death could be reported as an SAE even if it was due to disease progression.

Summaries by SOC and preferred term (PT) will include the following types:

- TEAEs;
- Treatment-related TEAEs
- Most common (≥ 10%) TEAEs (reported by PT only)
- Most common (≥ 10%) treatment-related TEAEs (reported by PT only)
- TEAEs leading to discontinuation of study drug
- Treatment-related TEAEs leading to discontinuation of study drug
- TEAEs leading to dose reduction
- TEAEs leading to drug interruption
- TEAEs leading to any dose modifications
- SAEs
- SAEs leading to discontinuation of study drug
- SAEs leading to dose reduction
- SAEs leading to drug interruption
- SAEs leading to any dose modifications
- Treatment-related SAEs
- Grade ≥ 3 TEAEs
- Grade ≥ 3 Treatment-related TEAEs
- TEAEs leading to death

Approved v1.0 930213273 1.0

- Treatment-related TEAEs leading to death

Subgroup analysis will be conducted for the following subgroups using the overall Safety Analysis Set for a subset of TEAE summaries, including the overall TEAE summary, and TEAE summary by SOC and PT for all TEAEs, Grade ≥ 3 TEAEs, SAEs, TEAEs leading to treatment discontinuation, and fatal TEAEs:

- Age (in years) categories: ≥12 to <18, ≥18 to <65, ≥65 to <75, and ≥75
- Sex: Male and Female
- Race: American Indian or Alaskan Native, Asian, White, Native Hawaiian or Pacific Islander, Black or African American, Other, and Not allowed by local law
- Dosing at 160 mg QD for 14 days followed by option to increase to 160 mg BID
  - This will include all subjects treated in Phase 2 and the midazolam substudy in Phase 1
- Brain metastasis by Investigator
  - Defined as having a target and/or non-target lesion in the brain selected at baseline for RECIST
- Region: US, Asia, and Other
  - Asian region includes subjects enrolled at sites in China, Hong Kong, Japan, Singapore, South Korea, and Taiwan.
  - Other includes Australia, Belgium, Canada, Denmark, France, Germany, Hungary, Italy, Netherlands, Poland, Spain, and United Kingdom.

Adverse Events of Special Interest (AESI)

Adverse events of special interest include medical concepts comprising composite preferred terms, which are assessed to determine clinical relevance in the target patient population. The AESI selection was multi-factorial, taking into consideration the frequent, severe, and serious adverse events observed in the clinical data, potential neurologic effects, associated with TRK inhibition and observed adverse events listed for tyrosine kinase inhibitors that are drugs in class for *ROS1*+ and *NTRK*+ tumors.

Neurologic Effects

Repotrectinib has shown to inhibit the neurotrophin receptor tyrosine kinases TRKA, TRKB, and TRKC in both biochemical and cellular potency assays. TRKs are known to play key roles in sensory neuron development and differentiation. Adverse events that have been attributed to TRK inhibition are associated with decreased proprioception and cerebellar dysfunction.

AESI include, Ataxia, Cognitive Disorders, Dizziness, Dysgeusia, Mood Disorders, Sleep Disorders, Paresthesia, Peripheral sensory neuropathy, Muscular Weakness, Hepatic Enzyme Elevations, Pneumonitis, QT prolongation, Skeletal Fractures, and Vision Disorders.

Others adverse events of interest may be added at the time of each data snapshot. Table 8 additionally provides a list of Medical Concepts defined by Higher-Level Terms (HLT) which will be used in the search strategy.

Approved v2.0 930213273 1.0

Summary tables will be analyzed by the Medical Concept and preferred terms identified within each group.

Table 8: AE of Special Interest Derivations

Medical Concept	MedDRA Terms Included
Paraesthesia	Higher Level Term: Paraesthesias and dysaesthesias
Dysgeusia	Higher Level Term: Sensory abnormalities NEC<br>Remove the Preferred Term: neuralgia
Peripheral sensory neuropathy	Higher Level Term: Peripheral neuropathies NEC<br>Plus the Preferred Term: neuralgia
Ataxia	Higher Level Term: Coordination and balance disturbances<br>Plus the Preferred Term: gait disturbance

Separate summaries of adverse events of special interest will include the following for TEAEs and repeated for treatment-related TEAEs (TRAEs). All sets will be repeated for the safety analysis set of subjects treated at the RP2D.:

- Overall summary of TEAEs by system and medical concept
- TEAEs leading to discontinuation of study drug
- TEAEs leading to dose reduction
- TEAEs leading to drug interruption
- TEAEs leading to drug modification
- Treatment-emergent SAEs
- TEAEs by maximum toxicity grade
- Grade ≥ 3 TEAEs
- Fatal TEAEs
- Time to first onset of each/ any TEAE within each medical concept (median and range)

Subgroup analyses for:

Age (in years) categories: ≥12 to <18, ≥18 to <65, ≥65 to <75, ≥75

Brain metastasis by Investigator:

- Defined as having a target and/or non-target lesion in the brain selected at baseline for RECIST

5.7.3 Clinical Laboratory Evaluations

Clinical laboratory parameters include Hematology, Coagulation, Chemistry.

Approved v2.0 930213273 1.0

Normal ranges will be used to identify values that are outside the normal ranges and abnormal gradable laboratory parameter results will be graded according to CTCAE v4.03. Descriptive statistics will be provided for each test result by visit and for the change from baseline by visit. Results collected at unscheduled visits will not be included in the by-visit analysis but will be included in shift tables that look across all visits.

Shift tables from baseline to worst post-baseline CTCAE grade will be presented, as appropriate, and will use laboratory results collected at planned and unscheduled visits. Baseline laboratory values will be defined described in Section 5.2. Particular analytes to be summarized in shift tables include:

- Hematology and coagulation: hemoglobin low, leukocyte count low, lymphocyte count high, lymphocyte count low, neutrophils count low, platelet count low, activated partial thromboplastin time.
- Chemistry: ALT high, Albumin low, ALK Phosphatase high, AST high, bilirubin high, calcium high, calcium low, creatinine high, GGT high, glucose high, glucose low, magnesium high, magnesium low, phosphate low, potassium high, potassium low, sodium high, sodium low, urate high

Graded lab data will also be analyzed for analytes where more than 20% of subjects had any worsening in grade from baseline, along with the percent of those subjects worsening to a Grade 3 or Grade 4.

Subjects who develop toxicities of CTCAE Grade ≥ 3 will be summarized and included in a listing.

In general, for CTCAE reporting, parameters that have criteria available for both low and high values (e.g., hypercalcemia versus hypocalcemia) will be summarized for both criteria. Subjects will only be counted once for each criterion.

In instances where local laboratory results were collected due to COVID-19 restrictions, specific lab normal ranges will be entered, units standardized, and CTCAE grades derived based on local lab ranges. The local lab data will be included in the shift table analysis but will not be included in the by-visit change from baseline analysis.

5.7.4 Drug-induced Liver Injury

A summary table will be provided for abnormal liver function tests to assess for drug-induced liver injury (DILI). The number and percentage of subjects having the following abnormal values on at least one visit will be provided:

- Alanine aminotransferase (ALT) > 3 x upper limit of normal (ULN), > 5x ULN, >10x ULN, >20x ULN
- Aspartate aminotransferase (AST) > 3x ULN, > 5x ULN, >10x ULN, >20x ULN
- Total bilirubin > 2x ULN
- Alkaline Phosphatase > 1.5x ULN

Number and percentage of subjects meeting the DILI laboratory criteria below will be identified by applying the following algorithm:

Approved v2.0 930213273 1.0

- (ALT or AST > 3x ULN) and Total Bilirubin > 2x ULN and Alkaline Phosphatase < 2x ULN. The DILI laboratory criteria will be evaluated for all laboratory values meeting the criteria at the same visit.

5.7.5 Performance Status and Vital Signs

Summary of ECOG performance status (0, 1, 2, 3, 4, 5) results at each scheduled time point will be presented for subjects over 18 years of age. Shift table from baseline to the worst change post-baseline will be tabulated for ECOG performance status. ECOG performance status will not be assessed for any subjects treated under the age of 18. Subjects treated who are 16 to < 18 years of age will be assessed using the Karnofsky Performance Status (scale from 0 to 100) and those who are 12 to < 16 years of age will be assessed using the Lansky Performance Status (scale 0 to 100). Analysis for these instruments will be similar to that for ECOG performance status. However, if the number of subjects treated in the 12 to 18 years age categories are limited, results over time, will be displayed in listings only.

Vital sign measurements and their post-baseline changes will be summarized at each scheduled timepoint.

An additional outlier analysis, presenting the number and percentage of subjects experiencing post-baseline changes of potential clinical importance (Table 9), will be presented.

Table 9: Vital Signs of Potential Clinical Importance

Vital sign	Criteria
Pulse (bpm)	Any post-baseline Pulse ≤ 50 and decrease from baseline ≥ 15<br>Any post- baseline Pulse > 100 and increase from baseline ≥ 15
Systolic blood pressure (mmHg)	Any post- baseline BP ≤ 90 and decrease from baseline ≥ 20<br>Any post- baseline BP ≥ 140 and increase from baseline ≥ 20
Diastolic blood pressure (mmHg)	Any post- baseline BP ≤ 50 and decrease from baseline ≥ 10<br>Any post- baseline BP ≥ 90 and increase from baseline ≥ 10
Temperature (C)	Any post- baseline T < 35.0 and decrease from baseline ≥ 1.1<br>Any post- baseline T > 38.0 and increase from baseline ≥ 1.1

5.7.6 Electrocardiograms

The analysis of electrocardiogram (ECG) results will be based on Safety Population subjects with baseline and on-treatment ECG data. Last average ECG value of triplicate measurements collected prior to the first day of dosing will be considered the baseline ECG. All ECG data are collected and analyzed through a central review vendor and they will provide a separate cardiac safety analysis report in addition to the analyses described below.

ECG measurements (an average of the triplicate measurements) will be used for the statistical analysis and all data presentations. Any data obtained from ECGs repeated for safety reasons after the nominal time points will not be averaged along with the preceding triplicates. Interval measurements from repeated ECGs will be included in the outlier analysis as individual values obtained at unscheduled time points.

Approved v1.0 930213273 1.0

QT intervals will be corrected for heart rate (QTc) using standard correction factors (i.e., Bazett's, Fridericia's). Actual value and change from baseline will be summarized for QT, PR, HR, RR, QRS, QTcF, and QTcB by visit and time point. For each subject, the maximum change from baseline will be calculated for all post-baseline time points. Outlier analysis of the QTcF data will be summarized as follows:

- The number of subjects with maximum change from baseline in QTcF (< 30, 30-60, and > 60 msec)
- The number of subjects with maximum post-dose (post-baseline) QTcF (< 450, 450 to < 480, 480 to ≤ 500, and > 500 msec).

ECG abnormality at baseline (yes, no, not done) and subjects experiencing clinically relevant morphological ECG changes will be included in listings at the individual protocol level.

5.7.7 Left Ventricular Ejection Fraction

For subjects with multigated acquisition (MUGA) scans or echocardiograms, individual left ventricular ejection fraction (LVEF) proportion (%) and its changes from baseline will be summarized by time point. The number and the percentage of subjects whose maximum decrease from baseline in LVEF is ≥ 20% will also be presented.

5.7.8 Death

A listing of all deaths will be presented by Safety Analysis Set and will include a column to identify if the death occurred within 28 days of the last dose of repotrectinib.

5.7.9 Patient-Reported Outcomes (PRO)

There will be a separate Statistical Analysis Plan and final report of results from the PRO instruments, European Organization for Research and Treatment of Cancer (EORTC) QLQ-C30 and QLQ-LC-13 (for subjects with either ROS1-, or NTRK-rearranged NSCLC) that were only administered in Phase 2.

Approved v2.0 930213273 1.0

6 ANALYSIS METHODS AND CONSIDERATIONS FOR CARE (TPX-0005-07)

6.1 Introduction

This section will describe the study objectives, study design, study evaluations, and detailed statistical analysis methods for the protocol TPX-0005-07 "A Phase 1/2, Open-Label Safety, Tolerability, Pharmacokinetics, and Anti-Tumor Activity Study of Repotrectinib in Pediatric and Young Adult Subjects with Advanced or Metastatic Malignancies Harboring ALK, ROS1 or NTRK1-3 Alterations". Any methods described here will supersede any analyses described in the protocol.

Prior to study completion, an interim database lock is planned for this study using a data cutoff date of 19 December 2022. The focus of this section of the document will be on analysis to be generated from the 28 February 2023 interim database lock. The purpose of this database lock is to provide analysis of pediatric subjects treated with repotrectinib to support a filing of application for marketing approval of repotrectinib specific to subjects with NTRK alterations. It is anticipated a separate, standalone analysis plan will be generated prior to study completion that addresses all study objectives specified in the protocol.

Unless specified otherwise, analysis methods described here will follow the considerations mentioned in Section 5.2 (General Considerations) of this document.

6.2 Analysis Population Sets

6.2.1 Full Analysis Set

The Full Analysis Set (FAS) consists of all subjects who are enrolled and have received repotrectinib.

The FAS will be used for the summary of demographics, baseline characteristics, prior treatment history and safety analyses.

Subjects who were screened but were not enrolled will not be in the FAS and will be listed and summarized as screen failures including their reason for screen failure and country.

Subjects who are enrolled but never treated will be included in listings.

6.2.2 NTRK Efficacy Evaluable Analysis Set

The NTRK Efficacy Evaluable Analysis Set will include all enrolled NTRK subjects who receive study treatment, have a baseline tumor assessment with documentation of measurable disease per central reviewer and have started treatment at least 8 months prior to the data cutoff date (had the opportunity to have 6 months of follow up from their first post-baseline scan).

6.2.3 PK Analysis Sets

The PK concentration analysis set will include all enrolled subjects who receive at least 1 dose of study drug and have at least one concentration of repotrectinib.

Approved v2.0 930213273 1.0

The PK parameter analysis set will include all enrolled subjects who receive at least 1 dose of study drug and have sufficient information to estimate at least one of the PK parameters of interest.

6.3 Disposition, Demographic and Baseline and Disease Characteristics

Separate summaries of Subject Disposition, Demographic and Baseline Characteristics and Disease Characteristics will be presented for the Full Analysis Set. In general, the summaries of Subject Disposition will follow the summaries described in Section 5.3 of this document and summaries of Demographic and Baseline Characteristics will follow the summaries described in Section 5.5 of this document. Items that will be different include the following:

- Data will be summarized by NTRK Subjects, Other Subjects and Overall. Other Subjects will include those subjects with ROS1 or ALK alterations.
- Age categories will be the following: (0-28 days, >28 days to < 2 years, 2 years to <12 years, 12 to < 18 years, 18-25 years)
- ECOG performance scores will be replaced with the following:
  - Karnofsky Performance Score (0 to 100 in increments of 10) for ages ≥16 to <18 years
  - Lansky Performance Score (0 to 100 in increments of 10) for ages < 16 years

Disease characteristics will include summaries of the following: Type of Cancer, Histological Classification, Time Since Diagnosis (years), Brain Metastasis, Stage at Study Entry, Stage at Diagnosis, Molecular Alteration Type.

A presentation of cancer treatments will also be summarized and include: Number of Lines of Prior Systemic Therapy, Number of Lines of Prior TKI, Prior Surgery, Prior Radiotherapy.

6.4 Efficacy Analysis

Efficacy data will be based on the NTRK Efficacy Evaluable Set using the radiologic assessments evaluated by the BICR. Response evaluations by Investigator will be analyzed as supportive, where appropriate. Efficacy data will be summarized by TKI-Naïve Subjects and TKI Pretreated Subjects. Summaries of subgroups by age (<12 years old, ≥12 years old) will be presented. If the number of subjects to be analyzed for any efficacy analysis becomes too small, a by-patient listing may be included instead of a summary table.

6.4.1 Analysis of Efficacy Endpoints

The primary endpoint for the Phase 1 study is to determine the MTD and the pediatric RP2D of repotrectinib.

The primary endpoint for the Phase 2 study is confirmed Overall Response Rate (ORR), as assessed by BICR using RECIST v1.1, in each subject population expansion cohort of advanced or metastatic malignancies that harbor a *ROS1*, *ALK*, *NTRK1-3* alterations. For subjects enrolled with malignancies that cannot be evaluated according to RECIST v1.1, the efficacy within this population will be evaluated separately using the appropriate criteria.

Approved v2.0 930213273 1.0

Subjects with primary CNS tumors will be evaluated using Response Assessment in Neuro-Oncology (RANO) criteria.

For analyses associated with the 28 February 2023 interim database lock, the primary endpoint will be ORR (across Phase 1 and Phase 2). Analyses will be based on the NTRK Efficacy Evaluable Set. Subjects with NTRK solid tumors will be pooled across phase, dose level and cohort and summarized as either TKI Naïve Subjects or TKI Pretreated Subjects.

Best Overall Response (BOR) will be determined by the highest qualitative value within a hierarchy of Overall Response assessments at each visit: Complete Response (CR) > Partial Response (PR) > Stable Disease (SD) > Progressive Disease (PD) > Not Evaluable (NE). If minimum time for SD (at least 6 weeks after the first dose of repotrectinib) has not been met prior to the first assessment, BOR will be dependent on subsequent evaluations. Subjects who do not have follow up dates after the first assessment of SD prior to the minimum requirement will be considered NE.

For subjects assessed using either the RECIST or RANO criteria, a confirmed response (CR or PR) will be a response that persists on a repeat-imaging performed at least 4 weeks after initial documentation of response. It is not required that the second assessment immediately follow the initial response, but a subject cannot be identified to have progressed prior to response confirmation. Subjects with a confirmed objective response (CR or PR) will be referred to as responders. Otherwise, subjects will be considered non-responders. Subjects who are unconfirmed CR or unconfirmed PR will be considered non-responders, unless subjects with unconfirmed CR already have a confirmed PR.

BOR will be based on assessments collected after the first dose of study drug until disease progression; assessments collected after the date of new cancer treatment will not be considered. BOR will be provided directly by the BICR and will be derived based on the BICR Charter; BOR based on investigator assessments will be derived as described above following the methods of the BICR Charter. BOR will be summarized based on the NTRK Efficacy Evaluable Set.

The ORR will be defined as the proportion of subjects with a confirmed complete response (CR) or partial response (PR). Subjects with a confirmed objective response (CR or PR) will be referred to as responders. Non-responders will include subjects without a confirmed objective response, SD, PD, NE, or missing.

The ORR will be reported as the proportion of responders by RECIST v1.1 or applicable disease-specific response criteria along with the corresponding 2-sided 95% Clopper-Pearson exact confidence interval (CI) (Clopper, 1934).

Analyses for ORR, BOR, and DOR will be presented for BICR and Investigator assessments.

A waterfall plot of the maximum percentage of tumor reduction in target lesions from baseline until disease progression may be provided; measurements collected after the date of new cancer treatment will not be included in the display.

A swimmer plot showing timeline of events for each subject, including duration of study therapy, start of response (CR and/or PR), progression or ongoing at the time of analysis may be presented by cohort.

Approved v2.0 930213273 1.0

DOR along with censoring flags will also be included in a listing. For details of derivation of DOR endpoint, including censoring rules, see Section 5.6 of this document.

6.5 Safety Analysis

Safety analysis will be performed using the Full Analysis Set for NTRK Subjects and Other Subjects, as well as overall.

6.5.1 Extent of Exposure

Extent of exposure with repotrectinib will be assessed by summaries of duration of treatment (months), number and percentage of subjects with each type of treatment modification, cumulative dose on study (mg) and number of treatment cycles started. Duration of treatment (months) will be summarized using descriptive statistics. Number of treatment cycles started will be summarized using counts of cycles and categories (> 6, > 12, > 15, > 18 cycles).

Duration of treatment with repotrectinib (months) will be calculated as (last dose date minus first dose date plus 1 day)/30.4375. Patients who are still on treatment at the time of data cutoff date will have duration of therapy calculated as ongoing through the data cutoff date.

Cumulative dose on study (mg) will be calculated as the actual total dose of repotrectinib taken on study.

Number of treatment cycles started per patient will be calculated as the duration of treatment divided by the length of a cycle (28 days). Patients who receive any part of a cycle will be considered to have received that cycle.

6.5.2 Adverse Events

Adverse events will be graded according to the NCI CTCAE v4.03 and coded to preferred term and system organ class (SOC) using the MedDRA version 25.0 (March 2022) or higher.

All AEs reported during the AE reporting period (inclusive AEs after the first dose of repotrectinib through the 28-day after receipt of the last dose of study drug) will be considered as treatment-emergent adverse events (TEAEs).

An overview summary of AE event types, including counts and percentages of participants with any TEAEs, TEAEs leading to any dose modifications (interruption of reduction), TEAEs leading to dose reduction, TEAEs leading to drug interruption; TEAEs leading to discontinuation of study drug; SAEs; Grade ≥ 3 TEAEs; and fatal TEAEs will be displayed. This summary will be repeated for treatment-related events. The overview will include a tabulation of counts and percentages for the incidence of maximum TEAE CTCAE grade; this will also be reported based on treatment-related TEAEs.

For each FAS population group (NTRK Subjects, Other Subjects and Overall), incidence rates will be summarized based on reporting of frequencies and percentages by MedDRA SOC and preferred term, with all subjects in each safety population group as the denominator, unless otherwise specified. In addition, AE incidence rates will also be further summarized by CTCAE grade. Subjects with multiple occurrences of events will only be counted once at the maximum CTCAE grade and strongest relationship to study drug for each preferred team, SOC, and overall for the specified event type. Treatment-related TEAEs will be summarized similarly. Treatment-

Approved v2.0 930213273 1.0

related adverse events (TRAEs) are those judged by the Investigator to be at least possibly related to the study drug. Deaths that occur within 28 days after the last dose of study drug are defined as on-study deaths and reported as SAEs within the required reporting period unless they are due to disease progression in which case they would not be reported as an SAE.

The following TEAEs will be summarized by SOC, PT, and maximum CTCAE grade:

- TEAEs
- TRAEs
- SAEs
- Grade ≥ 3 TEAEs
- TEAEs leading to discontinuation of study drug
- TEAEs leading to dose modifications (interruption or reduction)
- TEAEs leading to drug interruption
- TEAEs leading to dose reduction
- Fatal TEAEs

The following TEAEs will also be summarized by PT only:

- Most common (≥ 10%) TEAEs
- Most common (≥ 10%) TRAEs

Time to first onset of AEs (study day) will be determined and included in the listings along with action taken, and outcome. The listing of AEs by safety cohorts will be also provided.

Summaries of subgroups by age (<12 years old, ≥12 years old) will be presented for select summaries.

Adverse Events of Special Interest

Adverse events of special interest will be defined and analyzed for select tables as described in Section 5.7.2. Subgroup analysis will be performed only by age (<12 years old, ≥12 years old).

6.5.3 Clinical Laboratory Evaluations

Normal ranges will be used to identify values that are outside the normal ranges and abnormal laboratory results will be graded according to NCI CTCAE v4.03. Local results will be converted to standard international units based on the conversion factor and abnormal results will be graded according to NCI CTCAE v4.03.

Subjects who develop toxicities of CTCAE grade ≥ 3 will be included in a listing. Laboratory test results not having CTCAE grade will also be summarized.

Shift tables from baseline to worst post-baseline CTCAE grade will be presented, as appropriate, and will use laboratory results collected at planned and unscheduled visits. Baseline laboratory values will be defined described in Section 5.2. Particular analytes to be summarized in shift tables include:

Approved v2.0 930213273 1.0

- Hematology and coagulation: hemoglobin low, leukocyte count low, lymphocyte count high, lymphocyte count low, neutrophils count low, platelet count low, activated partial thromboplastin time.
- Chemistry: ALT high, Albumin low, ALK Phosphatase high, AST high, bilirubin high, calcium high, calcium low, creatinine high, GGT high, glucose high, glucose low, magnesium high, magnesium low, phosphate low, potassium high, potassium low, sodium high, sodium low, urate high

In general, for CTCAE reporting, parameters that have criteria available for both low and high values (e.g., hypercalcemia versus hypocalcemia) will be summarized for both criteria. Subjects will only be counted once for each criterion.

6.5.4 Drug-induced Liver Injury

A summary table will be provided for abnormal liver function tests to assess for drug-induced liver injury (DILI). The number and percentage of subjects having the following abnormal values on at least one visit will be provided:

- Alanine aminotransferase (ALT) > 3 x upper limit of normal (ULN), > 5x ULN, >10x ULN, >20x ULN
- Aspartate aminotransferase (AST) > 3x ULN, > 5x ULN, >10x ULN, >20x ULN
- Total bilirubin > 2x ULN
- Alkaline Phosphatase > 1.5x ULN

Number and percentage of subjects meeting the DILI laboratory criteria below will also be used to summarize subjects that meet Hy's Law criteria:

- (ALT or AST > 3x ULN) and Total Bilirubin > 2x ULN and Alkaline Phosphatase < 2x ULN.

The DILI criteria will be evaluated for all laboratory values meeting the criteria at the same visit.

6.5.5 Karnofsky/Lansky Performance Status

Subjects ages ≥ 16 to <18 years will be assessed for Karnofsky performance status (Table 10), and subjects ages < 16 years of age will be assessed for Lansky Performance Status.

Performance Status (KPS and LPS) results (0 to 100) at each scheduled visit will be presented in listings.

Approved v2.0 930213273 1.0

Table 10: Karnofsky and Lansky Performance Status Scale

Performance Status	Karnofsky Description	Lansky Description
100	Normal	Fully active, normal
90	Minor signs	Minor restrictions in strenuous physical activity
80	Normal with effort	Active, but tired more quickly
70	Cares for self	Greater restriction of plan and less time spent in play activity
60	Occasional assistance	Up and around, but active play minimal; keeps busy by being involved in quieter activities
50	Considerable assistance	Lying around much of the day, but gets dressed, no active playing, participates in all quiet play and activities
40	Disabled	Mainly in bed, participates in quiet activities
30	Severely	Bed bound, needing assistance even for quiet play
20	Very sick	Sleeping often, play entirely limited to very passive activities
10	Moribund	Doesn't play; doesn't get out of bed
0	Dead	Unresponsive

6.5.6 Vital Signs

Vital sign measurements and their post-baseline changes will be included in listings (body temperature, systolic blood pressure, diastolic blood pressure, heart rate and respiratory rate).

An outlier analysis, presenting the number and percentage of subjects experiencing post-baseline changes of potential clinical importance (Table 11), will be presented. Both scheduled and unscheduled visits will be considered for the outlier table.

Table 11: Vital Signs of Potential Clinical Importance

Vital sign	Criteria
Pulse (bpm)*	Any post-BL Pulse ≤threshold minimum for age and decrease from baseline ≥15<br>Any post-BL Pulse > threshold maximum for age and increase from baseline ≥ 15
Systolic blood pressure (mm Hg)	Any post- baseline BP =< 90 and decrease from baseline ≥ 20<br>Any post- baseline BP ≥ 140 and increase from baseline ≥ 20
Diastolic blood pressure (mm Hg)	Any post- baseline BP =< 50 and decrease from baseline ≥ 10<br>Any post- baseline BP ≥ 90 and increase from baseline ≥ 10
Temperature (F)	Any post- baseline T < 96 and decrease from baseline ≥ 2<br>Any post- baseline T > 101 and increase from baseline ≥ 2

*The minimum and maximum thresholds for pulse depend on age at the time of the visit according to the following criteria:

- Newborns 0 to <1 month old: 70 to 190 beats per minute
- Infants 1 to <12 months old: 80 to 160 beats per minute
- Children 1 to <3 years old: 80 to 130 beats per minute
- Children 3 to <5 years old: 80 to 120 beats per minute
- Children 5 to <7 years old: 75 to 115 beats per minute
- Children 7 to <10 years old: 70 to 110 beats per minute
- Children 10 years and older: 60 to 100 beats per minute

Approved v2.0 930213273 1.0

6.5.7 Prior and Concomitant Medications

All medications received during the protocol treatment period will be considered as concomitant medications and will be coded by the World Health Organization Drug Dictionary. Medications initiated and ended prior to the first dose date will be considered as prior medications. Concomitant medications will be summarized by ATC level 2 and generic drug names. Subjects who received prior and concomitant medications and/or treatments will be listed.

6.5.8 Electrocardiograms (ECG)

The analysis of ECG results will be based on FAS subjects with baseline and on-treatment ECG data. Last average ECG value of triplicate measurements collected prior to the first day of dosing will be considered the baseline ECG. All ECG data are collected and analyzed through a central review vendor, with the exception of any that may have been done locally due to COVID-19 restrictions. The local results, however, were sent to the central review vendor and analyzed centrally for consistency.

ECG measurements (an average of the triplicate measurements) will be used for the statistical analysis and all data presentations. Any data obtained from ECGs repeated for safety reasons after the nominal time points will not be averaged along with the preceding triplicates. Interval measurements from repeated ECGs will be included in the outlier analysis as individual values obtained at unscheduled time points.

QT intervals will be corrected for heart rate (QTc) using standard correction factors (i.e., Bazett's, Fridericia's, and possibly a study-specific factor). Data will be summarized and listed for ventricular heart rate (heart rate triplicate mean for summary table), QT, RR, PR, QRS, QTcF, and QTcB by cycle, day, and timepoint. Individual continuous ECG parameters as well as all triplicate averages will be listed. Interpretation by central read will also be presented in the listing.

For each subject, the maximum change from baseline of the QTcF will be calculated as well as the maximum post-baseline value of QTcF across all time points. Outlier analysis of the QTcF data will be conducted and summarized as follows:

- The number of subjects with maximum change from baseline in QTcF (< 30, 30-60, and > 60 msec)
- The number of subjects with maximum post-dose (post-baseline) QTcF (< 450, 450 to < 480, 480 to ≤ 500, and > 500 msec).

6.5.9 Left Ventricular Ejection Fraction (LVEF)

For subjects with MUGA scans or echocardiograms, individual LVEF proportion (%) and its changes from baseline will be included in listings. Considering both scheduled and unscheduled visits, the number of subjects and the percentage whose maximum decrease from baseline in LVEF is ≥ 20% will be calculated.

Approved v2.0 930213273 1.0

6.6 Pharmacokinetic Analysis

The plasma PK parameters listed below will be calculated, whenever possible, by non-compartmental analysis (NCA) methods for repotrectinib. These parameters will be calculated for Cycle 1 day 1 and Cycle 1 day 15 in Phase 1 and Phase 2 (only for subjects between 12 and 25 years of age in Phase 2). Non-compartmental analysis will also be conducted on repeat PK sampling, similar to the C1D15, if this should occur.

- Maximum plasma concentration (C_{max})
- Time to reach C_{max} (T_{max})
- AUC(0-24)

If data permit or if considered appropriate, area under the plasma concentration versus time curve from time zero to infinity (AUC_{inf}), terminal elimination half-life ($t_{½}$), apparent oral clearance (CL/F), apparent volume of distribution during terminal phase (V_z/F), and accumulation ratio (R_{acc}) will be estimated.

Repotrectinib concentrations will be summarized descriptively (n, mean, standard deviation, CV, median, minimum, maximum, geometric mean and its associated CV) by dose, cycle, day and nominal time. Individual, mean, and median profiles of the concentration-time data will be plotted by dose, cycle and day using nominal time except actual time for individual plots. Median and mean profiles will be presented on both linear and log-linear scales.

The PK concentration analysis set will be used for PK listings. The PK parameter analysis set will be used for summary statistics and statistical analyses of PK. Analysis will include all valid analyte data for repotrectinib and will be performed using actual times.

Individual PK parameters will be listed for each analyte, including any exclusions and reasons for exclusion. PK parameters will be summarized descriptively by dose, cycle, and day. Geometric means and geometric %CVs will be presented for C_{max}, AUC, CL/F, and V_z/F, and R_{acc}, if applicable. Median, minimum, and maximum values will be presented for T_{max}. Arithmetic means and SDs will be presented for $t_{½}$, if applicable.

In addition, the profiles of concentration-time data may be plotted by age groups, and PK parameters may be summarized descriptively by age groups.

6.6.1 Pharmacokinetic (PK) Summaries

In-text Tables

For in-text PK tables, %CV will be reported as integers. For other statistics except for SD, values of 100 or higher will be presented as integers, values of 10 - < 100 will be displayed to one decimal place, and values of 1 - < 10 will be displayed to two decimal places. Values less than one will be displayed to three decimal places. Ratios will also be displayed to three decimal places. SD will be reported to a precision of one decimal place more than the mean.

Handling of Non-Quantifiable Concentrations

For the summaries of plasma concentration-time data, concentrations that are less than the lower limit of quantification (LLOQ) should be displayed as "< LLOQ" in the listings and be treated as missing in summary tables and plots.

Approved v2.0 930213273 1.0

For the purpose of calculating PK parameters, predose concentrations that are less than LLOQ and concentrations prior to the first quantifiable concentration that are less than LLOQ will be set to zero, and all other concentrations less than LLOQ will be set to missing.

All available plasma concentration-time data and derived pharmacokinetic parameter values will be included in the PK data set and listed accordingly.

Treatment of Outliers

Individual plasma concentrations, if deemed to be anomalous, may be excluded from the analysis following a review of available documentation (e.g., bioanalytical report, clinical data). Any such exclusion will be clearly listed in the study report along with justification for exclusion.

Entire plasma concentration-time profiles for a subject may be excluded following review of available documentation (e.g., bioanalytical report, clinical data). Any such exclusion will be clearly listed in the study report along with justification for exclusion.

PK Exclusions

PK analysis, reporting, and exclusion criteria should follow the BMS PK Harmonization document, version 5.0. Specific guideline for exclusionary criteria for half-life and how other PK parameters are affected for exclusion are in the BMS PK Harmonization document.

Approved v1.0 930213273 1.0

7 REFERENCES

Clopper CJ, Pearson ES. The use of confidence or fiducial limits illustrated in the case of the binomial. Biometrika. 1934;26(4):404–413.

Eisenhauer EA, Therasse P, Bogaerts J, et al. New response evaluation criteria in solid tumours: revised RECIST guideline (version 1.1). Eur J Cancer. 2009;45(2):228-247. doi:10.1016/j.ejca.2008.10.026.

FDA Guidance for Industry. Clinical Trial Endpoints for the Approval of Cancer Drugs and Biologics. December 2018.

MedDRA, Introductory Guide MedDRA Version 25.0, March 2022.

NCI, Common Terminology Criteria for Adverse Events (CTCAE) Version 4.0 Published: May 28, 2009 (v4.03: June 14, 2010).

US FDA, XALKORI® Package Insert, 2010.

US FDA, ROZLYTREK® Package Insert, 2020.

US FDA, CYRAMZA® Package Insert, 2014.

Wen PY, Chang SM, Van den Bent MJ, et al. Response assessment in Neuro-oncology clinical trials. Journal of Clinical Oncology 2017 35:21, 2439-2449.

Approved v1.0 930213273 1.0
