# Supplementary material for: Repotrectinib in NTRK fusion–positive advanced solid tumors: a phase 1/2 trial
Source: Nat Med. 2026 Feb 4;32(2):682–9. doi: 10.1038/s41591-025-04079-7 (PMC12920079; doi:10.1038/s41591-025-04079-7)
Supplement: Supplementary file 2 — Reporting Summary [file 41591_2025_4079_MOESM2_ESM.pdf]

nature portfolio

Corresponding author(s): Benjamin Besse, MD, PhD

Last updated by author(s): Feb 10, 2025

Reporting Summary

Nature Portfolio wishes to improve the reproducibility of the work that we publish. This form provides structure for consistency and transparency in reporting. For further information on Nature Portfolio policies, see our Editorial Policies and the Editorial Policy Checklist.

Statistics

For all statistical analyses, confirm that the following items are present in the figure legend, table legend, main text, or Methods section.

n/a	Confirmed	
☐	☒	The exact sample size (n) for each experimental group/condition, given as a discrete number and unit of measurement
☒	☐	A statement on whether measurements were taken from distinct samples or whether the same sample was measured repeatedly
☒	☐	The statistical test(s) used AND whether they are one- or two-sided<br>*Only common tests should be described solely by name; describe more complex techniques in the Methods section.*
☒	☐	A description of all covariates tested
☒	☐	A description of any assumptions or corrections, such as tests of normality and adjustment for multiple comparisons
☒	☐	A full description of the statistical parameters including central tendency (e.g. means) or other basic estimates (e.g. regression coefficient) AND variation (e.g. standard deviation) or associated estimates of uncertainty (e.g. confidence intervals)
☒	☐	For null hypothesis testing, the test statistic (e.g. F, t, r) with confidence intervals, effect sizes, degrees of freedom and P value noted<br>*Give P values as exact values whenever suitable.*
☒	☐	For Bayesian analysis, information on the choice of priors and Markov chain Monte Carlo settings
☒	☐	For hierarchical and complex designs, identification of the appropriate level for tests and full reporting of outcomes
☒	☐	Estimates of effect sizes (e.g. Cohen's d, Pearson's r), indicating how they were calculated

Our web collection on statistics for biologists contains articles on many of the points above.

Software and code

Policy information about availability of computer code

Data collection	Data were collected from patients by study investigators and study staff at each study site. Clinical data from TRIDENT-1 were collected using Medidata Classic Rave 2023.2.0 from 152 sites across 19 countries. Patients enrolled in TRIDENT-1 from February 17, 2017, through October 15, 2023. The efficacy population included patients with NTRK+ solid tumors who started treatment with repotrectinib at any dose by February 15, 2023. The safety analysis population included patients with any tumor or fusion type who received at least one dose of repotrectinib.
Data analysis	Plots were generated and analyzed using SAS 9.4.

For manuscripts utilizing custom algorithms or software that are central to the research but not yet described in published literature, software must be made available to editors and reviewers. We strongly encourage code deposition in a community repository (e.g. GitHub). See the Nature Portfolio guidelines for submitting code & software for further information.

Data

Policy information about availability of data

All manuscripts must include a data availability statement. This statement should provide the following information, where applicable:

- Accession codes, unique identifiers, or web links for publicly available datasets
- A description of any restrictions on data availability
- For clinical datasets or third party data, please ensure that the statement adheres to our policy

Genomic data for this study are available at the European Genome-Phenome Archive (study, EGASXXX; dataset, EGADXXX). These and other data for this study may be requested in accordance with Bristol Myers Squibb's processes to ensure compliance with patient privacy and regulatory requirements. In-scope proposals for data requests are sent to and reviewed by an independent review committee (IRC) at the Duke Clinical Research Institute (DCRI) at Duke University. Review by an IRC is conducted to ensure that proposals requesting patient-level data receive a complete, consistent, and fair assessment, and they provide the final decision on the requests. The IRC consists of experts in three broadly defined areas of expertise, including clinical, statistical and bioethical/protection of human subjects. The IRC may also discuss the proposal with the study research team and additional experts if needed for the request. Proposals are evaluated based on scientific rationale and methodology, experience and relevant qualifications of the research team, presence of a robust statistical analysis plan, and publication plan. Plans for addressing potential conflicts of interest should be addressed. The researcher(s) will be expected to sign the Vivli Data Use Agreement prior to release of data, and the de-identified and/or anonymized datasets will be available within the Vivli Research environment upon agreement. The policy on data sharing for Bristol-Myers Squibb may be found at https://www.bms.com/researchers-and-partners/independent-research/data-sharing-request-process.html.

Human research participants

Policy information about studies involving human research participants and Sex and Gender in Research.

Reporting on sex and gender

Sex/gender was a pre-specified demographic variable in the study design for efficacy and safety analyses. Sex of patients in TRIDENT-1 was recorded by each site and is reported herein; gender was not recorded.

Population characteristics

Eligible patients had tumors harboring an NTRK fusion and were at least 18 years of age in phase 1 and at least 12 years of age in phase 2. The efficacy population included 51 TKI-naïve patients and 69 TKI-pretreated patients.

Median age was 61 years (range, 25–84) in the TKI-naïve cohort and 56 years (range, 18–81) in the TKI-pretreated cohort; approximately half were women (53% and 48%, respectively).

Among 18 NTRK+ tumor types reported, NSCLC was the most common at 53% and 25% in the TKI-naïve and TKI-pretreated cohorts, respectively, followed by thyroid cancer (12% and 10%), salivary gland cancer (10% and 17%), and soft tissue sarcoma (6% and 14%).

In the TKI-pretreated cohort, 26% of patients received 1 prior line of systemic therapy, 33% received 2 prior lines of systemic therapy, and 41% received 3 or more prior lines of systemic therapy.

Recruitment

Patients were recruited for the study by investigators at each site. Advertisements for patient recruitment were reviewed by study sponsor, institutional review boards, and ethics committees. As this is a single-arm study with no comparator arm, selection bias is less relevant.

Ethics oversight

The trial was conducted following US Food and Drug Administration regulations and the International Council for Harmonisation E6 guideline for Good Clinical Practice. Appropriate health authorities and institutional committees reviewed the protocol, and all patients provided written informed consent.

Note that full information on the approval of the study protocol must also be provided in the manuscript.

Field-specific reporting

Please select the one below that is the best fit for your research. If you are not sure, read the appropriate sections before making your selection.

☒ Life sciences ☐ Behavioural & social sciences ☐ Ecological, evolutionary & environmental sciences

For a reference copy of the document with all sections, see nature.com/documents/nr-reporting-summary-flat.pdf

Life sciences study design

All studies must disclose on these points even when the disclosure is negative.

Sample size

For the TRK TKI-naïve cohort, the planned sample size for the primary analysis was 55 patients. If the ORR was 35% or less, then it was not considered effective. If 27 out of 55 patients had a confirmed objective response (ORR = 49.1%; 95% CI, 35.4–62.9), where the lower limit of the 95% CI is > 35%, repotrectinib was considered to be efficacious in this cohort. After enrollment of 55 patients in the TKI-naïve cohort as specified above for the primary analysis, an additional 25 patients were to be enrolled for a total of approximately 80 patients in the TKI-naïve cohort.

For TRK TKI-pretreated cohort, the planned sample size for the primary analysis was 40 patients. Eligible patients with TRK TKI-pretreated NTRK+ solid tumors were enrolled into the cohort with 1 or 2 prior TRK TKI treatments. For this cohort, if the ORR was 10% or less, then it was assumed that repotrectinib was not effective. If 9 out of 40 patients had a confirmed objective response (ORR = 22.5%; 95% CI, 10.8–38.5), where the lower limit of 95% CI > 10%, repotrectinib was considered efficacious in this cohort. After enrollment of 40 patients in the TKI-pretreated cohort as specified above for the primary analysis, an additional 80 patients were to be enrolled for a total of 120 patients in this cohort.

The target sample size of 55 patients for the TKI-naïve cohort was not achieved within the reported cutoff date. We report herein patient populations of 51 and 69 for the TKI-naïve and TKI-pretreated cohorts, respectively, with at least 6 months of follow up for tumor assessment after first post-baseline scan, which is consistent with the registrational dataset agreed upon with regulatory agencies.

The safety population was determined by the number of patients who received any dose of repotrectinib in phase 1 or phase 2, regardless of tumor or fusion type, by October 15, 2023.

Data exclusions

For the efficacy population, patients with less than 6 months of follow-up for response were excluded from analysis. For the safety population, one patient was excluded due to not receiving a dose of study drug.

Replication

Data and analysis results reported in this study were checked for quality and accuracy. This clinical study has not yet been replicated. The findings reported herein could be prospectively validated in future trials.

Randomization

This was not a randomized controlled trial. Patients in the efficacy population were allocated to the TRK TKI-naïve cohort or TRK TKI-pretreated cohort based on treatment history.

Blinding

Investigators were not blinded during cohort allocation because this was done based on treatment history.

Reporting for specific materials, systems and methods

We require information from authors about some types of materials, experimental systems and methods used in many studies. Here, indicate whether each material, system or method listed is relevant to your study. If you are not sure if a list item applies to your research, read the appropriate section before selecting a response.

Materials & experimental systems

n/a	Involved in the study	
☒	☐	Antibodies
☒	☐	Eukaryotic cell lines
☒	☐	Palaeontology and archaeology
☒	☐	Animals and other organisms
☐	☒	Clinical data
☒	☐	Dual use research of concern

Methods

n/a	Involved in the study	
☒	☐	ChIP-seq
☒	☐	Flow cytometry
☒	☐	MRI-based neuroimaging

Clinical data

Policy information about clinical studies

All manuscripts should comply with the ICMJE guidelines for publication of clinical research and a completed CONSORT checklist must be included with all submissions.

Clinical trial registration

ClinicalTrials.gov: NCT03093116

Study protocol

The TRIDENT-1 trial protocol is available with this publication at Nature.com. It was also published in Drilon A, et al. N Engl J Med. 2024;390:118-131.
The following institutional review boards (IRBs) and committees reviewed the protocol: UC Irvine IRB, Memorial Sloan Kettering IRB, WCG IRB (previously WIRB), Dana-Farber Cancer Institute IRB, University of Michigan Medical School IRB, IRB of the Cleveland Clinic, Johns Hopkins Medicine IRB, Advarra IRB, UC San Diego Human Research Protections Program, NYU School of Medicine IRB, The University of Toledo - Human Research Protection Program - Cancer Biomedical IRB, The University of Chicago IRB, UT Southwestern Medical Center IRB, HealthPartners Institute IRB, MD Anderson Cancer Center Office of Protocol Research (OPR), University Health Network Research Ethics Board, HREBA Cancer Committee, William Osler Health System Research Ethics Board, Ottawa Health Science Network Research Ethics Board (OHSN-REB), UBC BC Cancer Research Ethics Board, Seoul National University Hospital, Yonsei Severence Hospital, Samsung Medical Center, Seoul St. Mary's Hospital IRB, Asan Medical Center IRB, Chungbuk National University Hospital, Konkuk University Medical Center IRB, Chonnam National University Hwasun Hospital, Peter MacCallum Cancer Centre, Ceim Instituto de Investigación Sanitaria La Fe, Comite de Protection des Personnes Sud Mediterranee III, Comitato Etico Territoriale Lombardia 4, London - Fulham Research Ethics Committee, Medisch Ethische Toetsingscommissie UMCG, Niezależna Komisja Bioetyczna do Spraw Badań Naukowych przy Gdańskim Uniwersytecie Medycznym, Ethikkommission der Medizinischen Fakultät der Universität zu Köln (Lead), Ethik-Kommission der Medizinischen Fakultät Heidelberg, Ethikkommission der Technischen Universität Dresden, Landesamt für Gesundheit und Soziales, (CEC) Ethics Committee Research UZ/KU Leuven, (LEC) Ethisch comité UZA/UA Antwerpen, De Videnskabsetiske Komiteer for Region Hovedstaden, Medical Research Council Ethics Committee for Clinical Pharmacology, Research Ethics Committee D of National Taiwan University Hospital, MacKay Memorial Hospital Institutional Review Board, National Cheng Kung University Hospital IRB, DSRB, IRB of the University of Hong Kong/Hospital Authority Hong Kong West

Cluster, Kowloon West Cluster Research Ethics Committee (KWC-REC), Joint Chinese University of Hong Kong - New Territories East Cluster Clinical Research Ethics Committee, and Hong Kong Oncology Study Group Research Ethics Committee.

Data collection

Data were collected from patients by study investigators and study staff at each study site. Clinical data from TRIDENT-1 were collected using Medidata Classic Rave 2023 2.0. A list of study investigators and sites were data were collected is provided in the Supplementary Information. All study sites are listed at the clinical trial registration website (https://www.clinicaltrials.gov/study/NCT03093116#contacts-and-locations). Patients enrolled in TRIDENT-1 from February 17, 2017, through October 15, 2023. The efficacy population included 120 patients with NTRK+ solid tumors who started treatment with repotrectinib at any dose by February 15, 2023, and with at least 6 months of follow up for tumor assessment after first post-baseline scan, which is consistent with the registrational dataset agreed upon with regulatory agencies. These 120 patients were allocated to the TRK TKI-naïve cohort (n = 51) or TRK TKI-pretreated cohort (n = 69) based on treatment history. The safety analysis population included 565 patients with any tumor or fusion type who received at least one dose of repotrectinib prior to data cutoff of October 15, 2023. The NTRK cohorts of TRIDENT-1 are still enrolling.

Outcomes

Phase 2 primary endpoint was confirmed objective response (complete response or partial response) as assessed by blinded independent central review (BICR) according to Response Evaluation Criteria in Solid Tumors (RECIST), version 1.1. Key secondary endpoints included duration of response, clinical benefit, progression-free survival, overall survival, intracranial response in patients with measurable brain metastasis at baseline, as assessed by BICR according to modified RECIST (mRECIST), version 1.1, safety as assessed with the Common Terminology Criteria for Adverse Events, version 4.03, and patient-reported outcomes as assessed with the European Organisation for Research and Treatment of Cancer Quality of Life Questionnaire–Core 30 (EORTC QLQ-C30). Exploratory endpoints included potential prognostic utility of genomic alterations, emergence of repotrectinib resistance mutations, and confirmed response by patient subgroup (demographic and baseline risk factors).
